# Hydroxy group-enabled highly regio- and stereo-selective hydrocarboxylation of alkynes†
†Electronic supplementary information (ESI) available. CCDC 1571775 and 1845002. For ESI and crystallographic data in CIF or other electronic format see DOI: 10.1039/c8sc05743e


**DOI:** 10.1039/c8sc05743e

**Published:** 2019-04-17

**Authors:** Chaofan Huang, Hui Qian, Wanli Zhang, Shengming Ma

**Affiliations:** a Research Center for Molecular Recognition and Synthesis , Department of Chemistry , Fudan University , 220 Handan Lu , Shanghai 200433 , P. R. China . Email: dxdzwl@sina.com; b State Key Laboratory of Organometallic Chemistry , Shanghai Institute of Organic Chemistry , Chinese Academy of Sciences , 345 Lingling Lu , Shanghai 200032 , P. R. China . Email: masm@sioc.ac.cn

## Abstract

Here we present an example of utilizing hydroxy groups for regioselectivity control in the addition reaction of alkynes—a highly efficient Pd-catalyzed *syn*-hydrocarboxylation of readily available 2-alkynylic alcohols with CO in the presence of alcohols with an unprecedented regioselectivity affording 3-hydroxy-2(*E*)-alkenoates. The role of the hydroxy group has been carefully studied. The synthetic potential of the products has also been demonstrated.

## Abstract 2

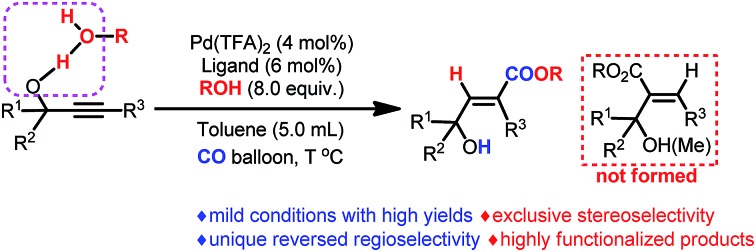
Hydrogen bonding-enabled highly regio- and stereo-selective hydrocarboxylation of alkynes has been successfully developed to afford 3-hydroxy-2(*E*)-alkenoates with up to 97% yield.

## Introduction

**Scheme 1:**
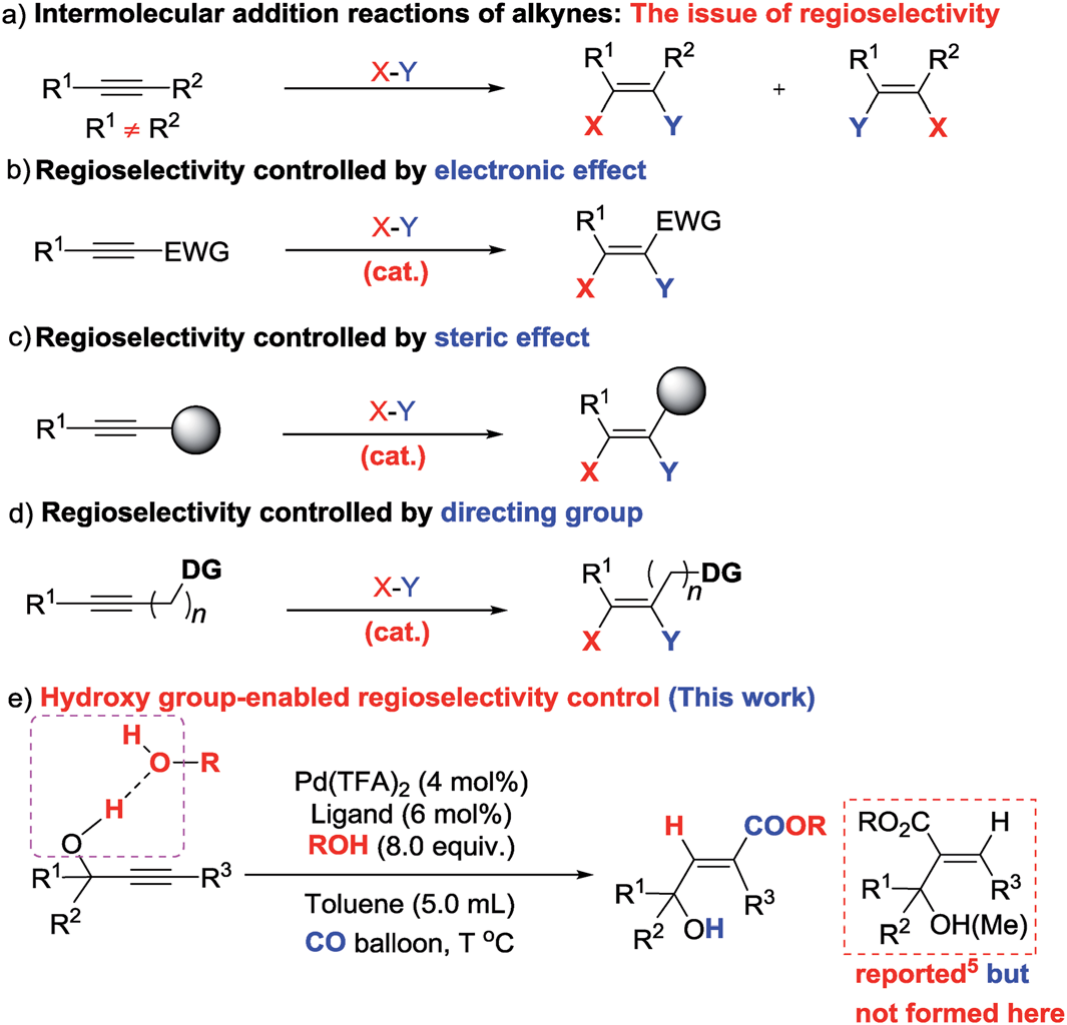
Addition reactions of alkynes—approaches for regioselectivity control (only *syn*-additions are shown for clarity).

As one of the most abundant and fundamental chemical feedstock, alkynes are widely applied in biochemistry, materials sciences, pharmacology, and medicine.^1^ Among many reactions, their addition reactions with another molecule, X–Y, perfectly suit the demand for green chemistry due to the 100% atom economy, thus leading to tremendous interest in this area due to the high importance of stereo-defined olefins.^2^ However, regioselectivity is the issue when it comes to non-symmetric alkynes (Scheme 1a). Electronic and steric effects help in solving this type of problem (Scheme 1b and c).^3^ Using a pre-installed directing group, such as carbonates, pyridyl groups, amides, alkenes, *etc.*, is another feasible way to control regioselectivity through coordination with metal catalysts (Scheme 1d).^4^,^5^ As we know hydrogen bonding interactions have been widely used in organocatalysis,^6^ and recent publications also demonstrate their capacity in regioselective addition reactions.^7^ Herein, we report our recent observation on hydroxy group-enabled regioselectivity control in highly stereoselective hydrocarboxylation of readily available 2-alkynylic alcohols affording highly functionalized 3-hydroxy-2(*E*)-alkenoates (Scheme 1e).

## Results and discussion

**Fig. 1:**
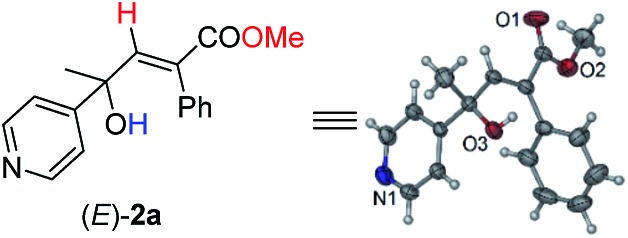
ORTEP representation of (*E*)-**2a**.

Initially, 1-phenyl-2-(pyridin-4-yl)but-3-yn-2-ol (**1a**) was treated with 8 equiv. of MeOH in the presence of 2 mol% [PdCl(π-allyl)]_2_, 6 mol% DPEphos, and 5 mol% (PhO)_2_POOH with a CO balloon. Surprisingly, the expected *syn*-hydrocarboxylation product (*E*)-**2a′**^5^ was not detected, while 66% of its regioisomeric product (*E*)-**2a** was exclusively formed unexpectedly together with 25% recovery of **1a** (Table 1, entry 1). The regio- and stereo-selectivity were further established by single-crystal X-ray diffraction analysis of (*E*)-**2a** (Fig. 1). Various palladium catalysts were then screened with no obvious improvement except for Pd(TFA)_2_, which afforded 82% yield of (*E*)-**2a** (Table 1, entries 2–4). Pd(0) pre-catalysts were also examined, affording (*E*)-**2a** with 57–82% yields (Table 1, entries 5–7). Among all the ligands examined, DPEphos was still the best (Table 1, entries 8–10). When the reaction was conducted at 60 °C, the yield of (*E*)-**2a** was improved to 90% (Table 1, entry 11). Besides, the reaction could also occur efficiently without the help of (PhO)_2_POOH (Table 1, entry 13).^8^

**Table 1:** Optimization of the reaction conditions

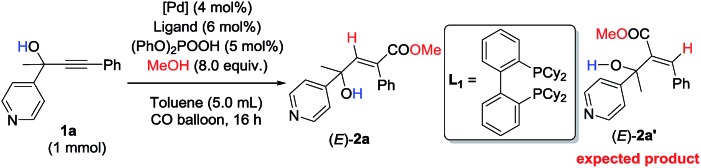					
Entry	[Pd]	Ligand	*T*/°C	(*E*)-**2a**^*a*^ (%)	Recovery^*a*^ (%)
1	[PdCl(π-allyl)]_2_	DPEphos	80	66	25
2	Pd(PPh_3_)_2_Cl_2_	DPEphos	80	0	100
3	Pd(TFA)_2_	DPEphos	80	82	6
4	Pd(OAc)_2_	DPEphos	80	70	4
5	Pd_2_(dba)_3_	DPEphos	80	57	34
6	Pd(*t*-Bu_2_-PPh)_2_	DPEphos	80	78	12
7	Pd(PPh_3_)_4_	DPEphos	80	82	15
8	Pd(TFA)_2_	BINAP	80	26	67
9	Pd(TFA)_2_	DPPB	80	33	43
10	Pd(TFA)_2_	**L_1_**	80	32	69
11	Pd(TFA)_2_	DPEphos	60	90	0
12	Pd(TFA)_2_	DPEphos	50	45	53
13^*b*^	Pd(TFA)_2_	DPEphos	60	89	0

^*a*^Yield and recovery were determined by ^1^H-NMR analysis using CH_2_Br_2_ as the internal standard.

^*b*^The reaction was carried out without (PhO)_2_POOH.

With the optimized conditions in hand and the importance of such 2-alkenoates, we set out to explore the scope of this reaction (Table 2). To our delight, this highly regioselective *syn*-hydrocarboxylation reaction delivered 3-hydroxy-2(*E*)-alkenoates as the sole product for various 2-alkynylic alcohols. Substitution of the pyridine ring at different positions made no difference (Table 2, entries 1–3). Quinolinyl-containing substrates were also compatible, efficiently furnishing (*E*)-**2d** in 71% yield (Table 2, entry 4). An electron-rich 3-aryl-substituted 2-alkynylic alcohol gave a higher yield (Table 2, entry 5). Compared with aromatic groups, 3-alkyl-substituted substrates **1f** and **1g** were less reactive and required a higher catalyst loading and temperature (Table 2, entries 6 and 7). Notably, the reaction could also be executed with more sterically hindered 2-alkynylic alcohols, delivering (*E*)-**2h** and (*E*)-**2i** in good yields (Table 2, entries 8 and 9). Interestingly, reaction with a 2-alkynylic alcohol with a *p*-nitrophenyl group instead of a pyridyl group also proceeded smoothly to give (*E*)-**2j** in 58% yield (Table 2, entry 10).

**Table 2:** Substrate scope-1

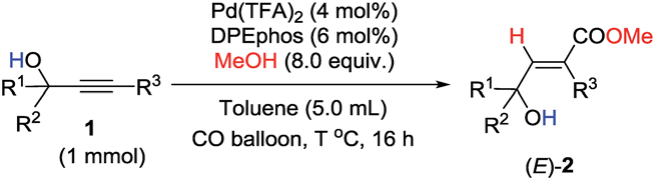					
Entry	R^1^	R^2^	R^3^	*T*/°C	(*E*)-**2** yield^*a*^/%
1	Me	4-pyridyl	Ph (**1a**)	60	82 (**2a**)
2	Me	3-Pyridyl	Ph (**1b**)	60	80 (**2b**)
3	Me	2-Pyridyl	Ph (**1c**)	60	62 (**2c**)
4	Me	3-Quinolinyl	Ph (**1d**)	60	71 (**2d**)
5	Me	4-Pyridyl	4-MeC_6_H_4_ (**1e**)	60	89 (**2e**)
6^*b*^	Me	4-Pyridyl	*n*-Bu (**1f**)	70	86 (**2f**)
7^*b*^	Me	4-Pyridyl	*n*-C_8_H_17_ (**1g**)	70	81 (**2g**)
8	Et	4-Pyridyl	Ph (**1h**)	60	88 (**2h**)
9^*c*^	Ph	4-Pyridyl	*n*-Bu (**1i**)	75	81 (**2i**)
10^*d*^	Me	4-O_2_NC_6_H_4_	*n*-C_6_H_13_ (**1j**)	70	58 (**2j**)

^*a*^Isolated yield.

^*b*^With 6 mol% Pd(TFA)_2_ and 9 mol% DPEphos.

^*c*^With 6 mol% Pd(TFA)_2_, 9 mol% DPEphos, and 6.0 mmol of MeOH for 24 h.

^*d*^With 4 mol% Pd(TFA)_2_, 8 mol% DPEphos, and 4.0 mmol of MeOH.

Unfortunately, this set of reaction conditions did not work very efficiently for 2-alkynylic alcohols with R^1^ and R^2^ both being alkyl groups—the reaction of **1k** resulted in the formation of the desired (*E*)-**2k** in only 35% yield (Table 3, entry 1). Lowering the temperature increased the yield up to 49% (Table 3, entry 2). Then, the ligand effect was re-investigated to address this issue. As shown in Table 3, mono-phosphine ligands were not efficient for the hydrocarboxylation (Table 3, entries 3 and 4). It is also worth noting that the efficiency strongly depends on the electronic properties and the backbone structure of the bisphosphine ligands. Compared to 2,2′-bis(dicyclohexyl-phosphino)-1,1′-biphenyl (**L_1_**), more rigid or more flexible backbone structures both made the reaction slower (Table 3, entries 5–7). Furthermore, a relatively electron-deficient ligand, BIPHEP, gave only 5% yield of the product with 95% recovery of **1k** (Table 3, entry 8). Finally, when the reaction was carried out with 4 equiv. of MeOH at 60 °C, (*E*)-**2k** could be obtained with the highest yield (Table 3, entry 9).

**Table 3:** Further optimization of the reaction conditions

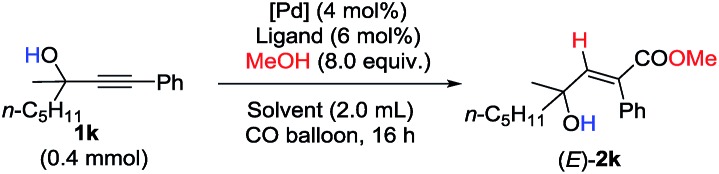					
Entry	Ligand	Solvent	*T*/°C	(*E*)-**2k**^*a*^ (%)	Recovery^*a*^ (%)
1^*b*^	DPEphos	Toluene	60	35	0
2^*b*^	DPEphos	Toluene	50	49	0
3^*c*^	Zheda-phos	Toluene	50	1	93
4^*c*^	Sphos	Toluene	50	11	88
5	BINAP	Toluene	50	40	44
6	DPPB	Toluene	50	17	66
7	**L_1_**	Toluene	50	58	42
8	BIPHEP	Toluene	50	5	95
9^*b*^ ^,^^*d*^	**L_1_**	Toluene	60	99 (95)	0
10^*b*^ ^,^^*d*^	**L_1_**	THF	60	34	66
11^*b*^ ^,^^*d*^	**L_1_**	1,2-DCE	60	30	70
12^*b*^ ^,^^*d*^	**L_1_**	CH_3_CN	60	8	92
13^*b*^ ^,^^*d*^	**L_1_**	DMF	60	5	90
14^*b*^ ^,^^*d*^	**L_1_**	DMSO	60	—	98

^*a*^Yield and recovery were determined by ^1^H-NMR analysis using CH_2_Br_2_ as the internal standard, and the isolated yield is shown in parentheses.

^*b*^The reaction was carried out on a 1 mmol scale with 5 mL of toluene.

^*c*^With 12 mol% mono-phosphine ligands.

^*d*^With 4.0 equiv. of MeOH for 24 h.

**Fig. 2:**
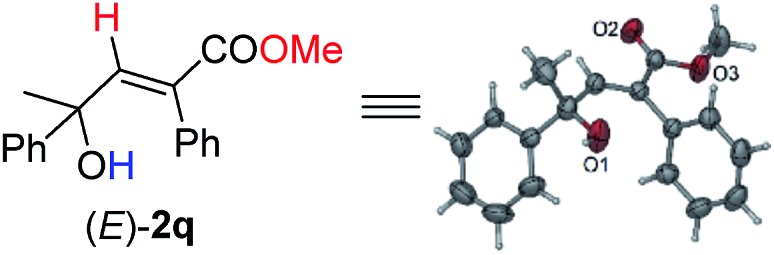
X-ray crystal structure of (*E*)-**2q**.

Under this set of new optimal reaction conditions, more examples of 2-alkynylic alcohols were examined. As shown in Table 4, R^2^ and R^3^ are both compatible with an alkyl or aryl group (Table 4, entries 1–9). The structure of (*E*)-**2q** was further confirmed by its single crystal X-ray diffraction analysis (Fig. 2). In addition, a 2-alkynylic alcohol with a four-membered cyclo-butyl ring also survived affording (*E*)-**2s** in 87% yield (Table 4, entry 10). Reaction with more sterically hindered 1,1-diphenylhept-2-yn-1-ol proceeded smoothly to give (*E*)-**2t** in 86% yield and 9% recovery of **1t** (Table 4, entry 11). It is noteworthy that secondary 2-alkynylic alcohols also afforded the target products in good to excellent yields with the same regio- and stereo-selectivity (Table 4, entries 12–15). Interestingly, even the C–Br bond could survive in this reaction (Table 4, entries 2, 3, and 15). The reaction could be easily executed on a gram scale, delivering (*E*)-**2l** in 89% yield (Table 4, entry 3).

**Table 4:** Substrate scope-2

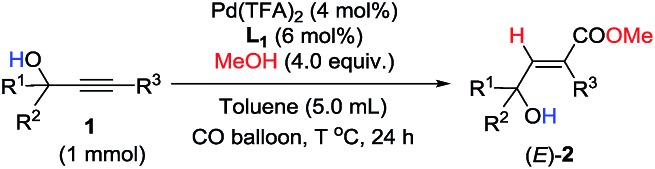					
Entry	R^1^	R^2^	R^3^	*T*/°C	(*E*)-**2** yield^*a*^/%
1	Me	*n*-C_5_H_11_	Ph (**1k**)	60	95 (**2k**)
2	Me	*n*-C_5_H_11_	4-BrC_6_H_4_ (**1l**)	60	92 (**2l**)
3^*b*^	Me	*n*-C_5_H_11_	4-BrC_6_H_4_ (**1l**)	60	89 (**2l**)
4^*c*^ ^,^^*d*^	Me	*n*-C_5_H_11_	*n*-Bu (**1m**)	80	66 (**2m**)
5^*e*^ ^,^^*f*^	Me	*n*-Pr	*n*-C_8_H_17_ (**1n**)	75	71 (**2n**)
6^*c*^ ^,^^*g*^	Me	(CH_2_)_2_CH=CH_2_	*n*-Pr (**1o**)	75	41 (**2o**)
7^*e*^ ^,^^*h*^	Me	*n*-Pr	(CH_2_)_4_Cl (**1p**)	75	60 (**2p**)
8	Me	Ph	Ph (**1q**)	70	93 (**2q**)
9^*c*^ ^,^^*i*^	Me	Ph	*n*-Bu (**1r**)	75	63 (**2r**)
10	–(CH_2_)_3_–		Ph (**1s**)	25	87 (**2s**)
11^*c*^ ^,^^*j*^	Ph	Ph	*n*-Bu (**1t**)	80	86 (**2t**)
12	H	*n*-C_11_H_23_	Ph (**1u**)	60	93 (**2u**)
13^*c*^	H	Ph	Ph (**1v**)	70	69 (**2v**)
14^*c*^	H	Ph	4-MeOC_6_H_4_ (**1w**)	70	69 (**2w**)
15	H	*n*-C_11_H_23_	4-BrC_6_H_4_ (**1x**)	60	87 (**2x**)

^*a*^Isolated yield.

^*b*^The reaction was carried out on a 4 mmol scale.

^*c*^With 5 mol% Pd(TFA)_2_, 10 mol% **L_1_,** and 5.0 mmol of MeOH.

^*d*^Reaction time 48 h; 19% recovery of **1m** was detected.

^*e*^With 6 mol% Pd(TFA)_2_, 12 mol% **L_1_,** and 5.0 mmol of MeOH.

^*f*^Reaction time 48 h; 27% recovery of **1n** was detected.

^*g*^36% recovery of **1o** was detected.

^*h*^Reaction time 48 h; 34% recovery of **1p** was detected.

^*i*^Reaction time 48 h; 21% recovery of **1r** was detected.

^*j*^Reaction time 32 h; 9% recovery of **1t** was detected.

In addition to methanol, some other alcohols were also examined. Ethanol and TMSCH_2_OH work well to obtain the target products in 95–97% yield (Table 5, entries 1 and 2). Sterically hindered i-PrOH is also tolerated with 76% yield (Table 5, entry 3). Phenol behaves worse, and only 30% yield was detected (Table 5, entry 4).^9^

**Table 5:** Substrate scope-3

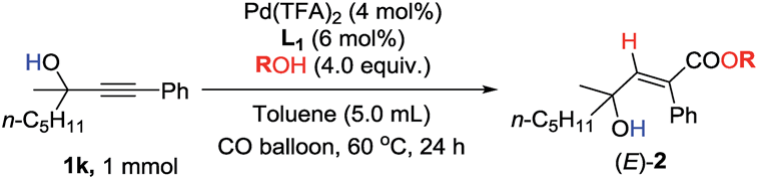		
Entry	ROH	Yield of (*E*)-**2**^*a*^/%
1	EtOH	95 (**2ka**)
2	TMSCH_2_OH	97 (**2kb**)
3^*b*^	i-PrOH	76 (**2kc**)
4^*c*^	PhOH	30 (**2kd**)

^*a*^Isolated yield.

^*b*^24% recovery of **1k** was determined by ^1^H-NMR analysis using CH_2_Br_2_ as the internal standard.

^*c*^70% recovery of **1k** was determined by ^1^H-NMR analysis using CH_2_Br_2_ as the internal standard.

Furthermore, as shown in Table 6, racemization of the chiral center in substrates (*S*)-**1** 10 was not observed—the reaction of optically active propargylic alcohols afforded optically active 3-hydroxy-2(*E*)-alkenoates with excellent ee values and high yields.

**Table 6:** Palladium-catalyzed hydrocarboxylation of chiral propargylic alcohols

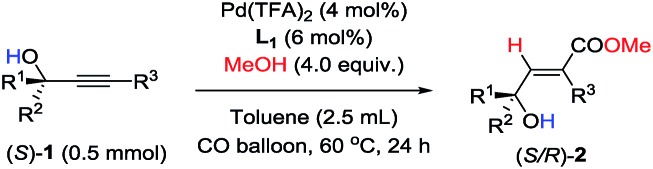					
Entry	(*S*)-**1**			**2** ^*a*^	
	R^1^	R^2^	R^3^	Yield/%	ee/%
1^*b*^ ^,^^*c*^	H	*n*-C_11_H_23_	Ph (**1u**, 98)	90 (**2u**)	99
2	H	*n*-C_11_H_23_	4-BrPh (**1x**, >99)	86 (**2x**)	99
3^*b*^ ^,^^*d*^	H	Ph	Ph (**1v**, >99)	68 (**2v**)	99
4^*b*^ ^,^^*d*^	H	Ph	4-MeOPh (**1w**, >99)	70 (**2w**)	99
5^*b*^	Me	*n*-C_5_H_11_	Ph (**1k**, 97)	89 (**2k**)	96

^*a*^Isolated yield; ee values were determined by chiral HPLC analysis.

^*b*^The reaction was carried out at 70 °C.

^*c*^With 4 mol% Pd(TFA)_2_ and 8 mol% **L_1_**.

^*d*^With 5 mol% Pd(TFA)_2_, 10 mol% **L_1_**, and 5.0 equiv. of MeOH.

**Scheme 2:**
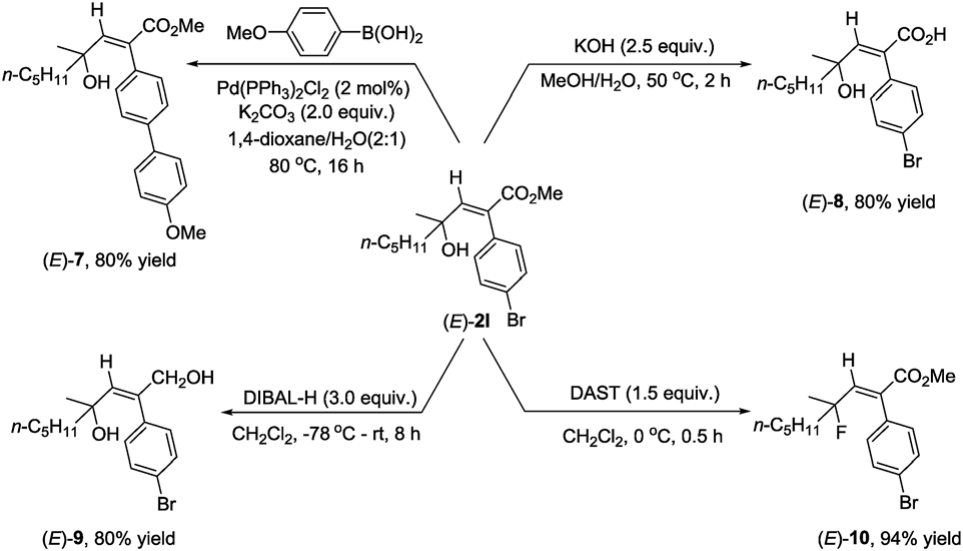
Synthetic applications of (*E*)-**2l**.

As we know that 2-alkenoates are important intermediates in organic synthesis, their synthetic potential has been further demonstrated for the synthesis of different stereo-defined functionalized olefins. Owing to the presence of the C–Br bond in (*E*)-**2l**, Suzuki coupling reactions could easily afford (*E*)-**7** in 80% yield.^11^ The ester unit could be hydrolyzed with KOH at 50 °C for 2 hours to afford the corresponding acid (*E*)-**8** in 80% yield,^12^ or reduced with DIBAL-H at –78 °C delivering the corresponding 1,4-diol (*E*)-**9** in 80% yield.^13^ Fluorination of the hydroxyl group could also be easily conducted with DAST to furnish (*E*)-**10** in 94% yield^14^ (Scheme 2).

**Scheme 3:**
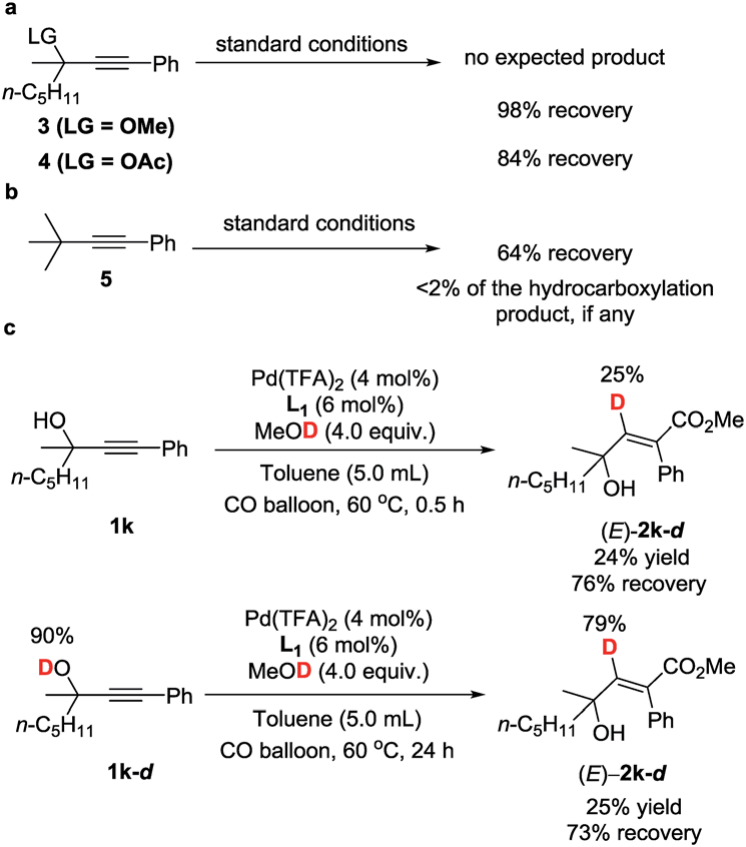
Mechanistic studies.

**Fig. 3:**
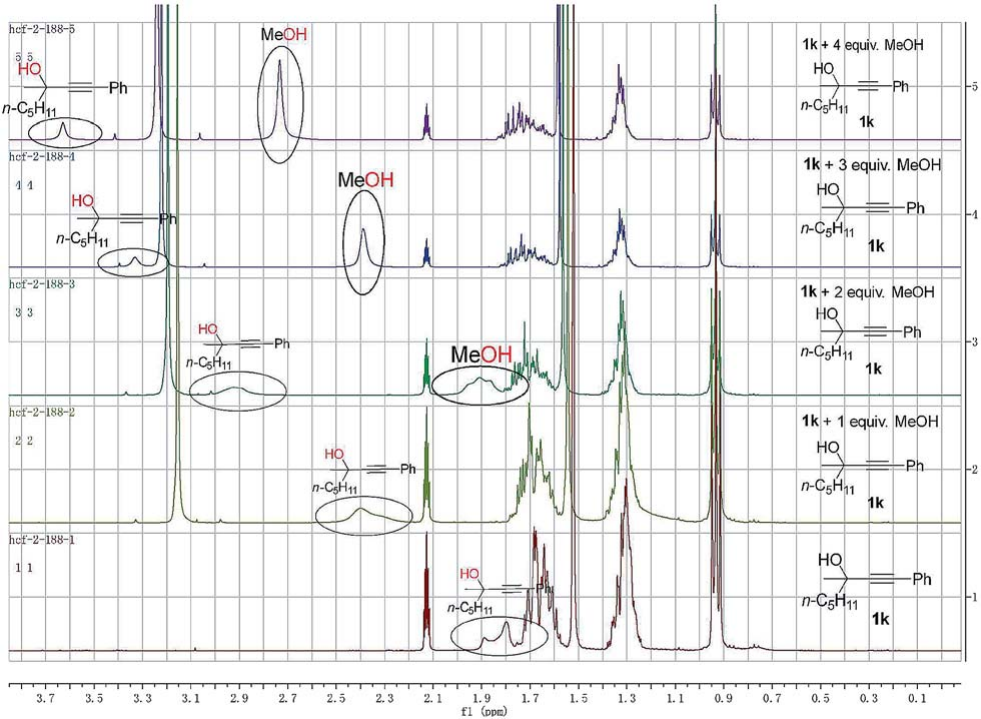
NMR investigation on hydrogen bonding.

To gain insight into the reaction mechanism and the effect of the hydroxyl group, a couple of control experiments were conducted (Scheme 3). No desired hydrocarboxylation products were obtained when propargylic methyl ether **3**, acetate **4**, or internal alkyne **5** was utilized (Scheme 3a and b), indicating that the hydrogen bonding originating from the free hydroxyl groups in propargylic alcohol and methanol might have played a critical role in this transformation. Isotopic labeling studies reinforce the notion that methanol was the hydrogen donor (Scheme 3c). We reasoned that the low D incorporation was caused by adventitious water in the reaction mixture. Furthermore, the ^1^H NMR signals of 1-phenyl-3-methyloctyn-3-ol **1k** were measured with respect to different amounts of MeOH and **1k**: an obvious shift of the hydroxy signal in **1k** and MeOH was observed, indicating hydrogen bonding between the two hydroxyl groups (Fig. 3).

**Fig. 4:**
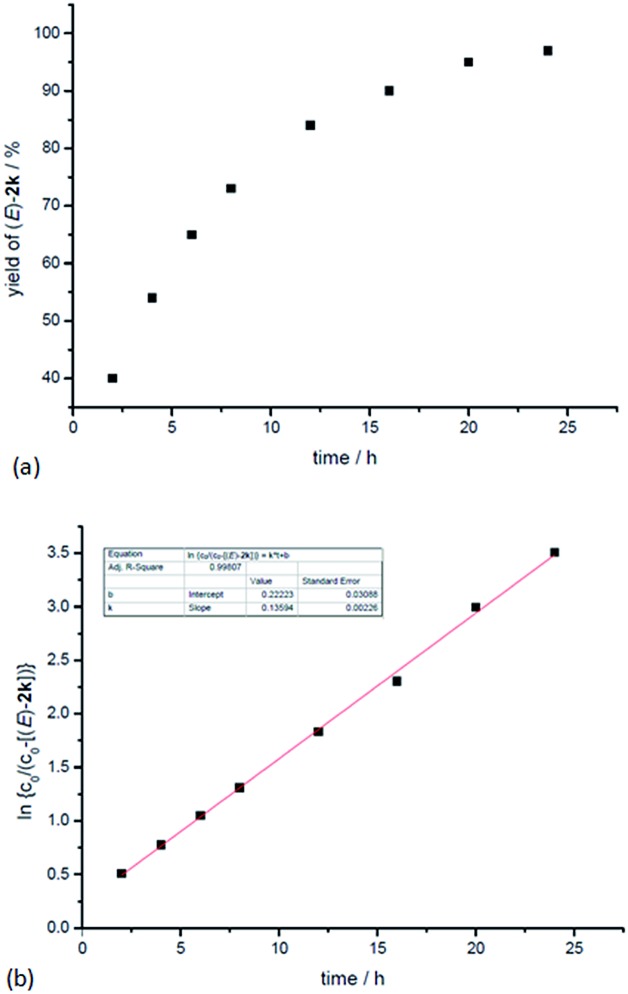
Determination of the reaction order of propargylic alcohol. (a) Yield of (*E*)-**2k***vs.* time. (b) ln{*c*_0_/(*c*_0_ – [(*E*)-**2k**])} *vs.* time (*R*-squared is the coefficient of determination).

In addition, kinetic studies were also carried out. Linear relationships were obtained for ln{*c*_0_/(*c*_0_ – [(*E*)-**2k**])} *vs.* reaction time (*c*_o_ is the initial concentration of **1k**), even with 10-fold excess of MeOH to ensure pseudo zero order in MeOH, indicating first-order dependence of the reaction rate with respect to propargylic alcohol (Fig. 4b). An experiment was also carried out to measure the rate of H/D-scrambling. By adding MeOD into the solution of **1k** in CDCl_3_ and then subjecting the mixture to ^1^H NMR analysis immediately, the H/D-exchange process was found to reach an equilibrium state within 3 minutes (for details, see the ESI†), which is much faster than the rate of this hydrocarboxylation reaction (Fig. 4a).

**Fig. 5:**
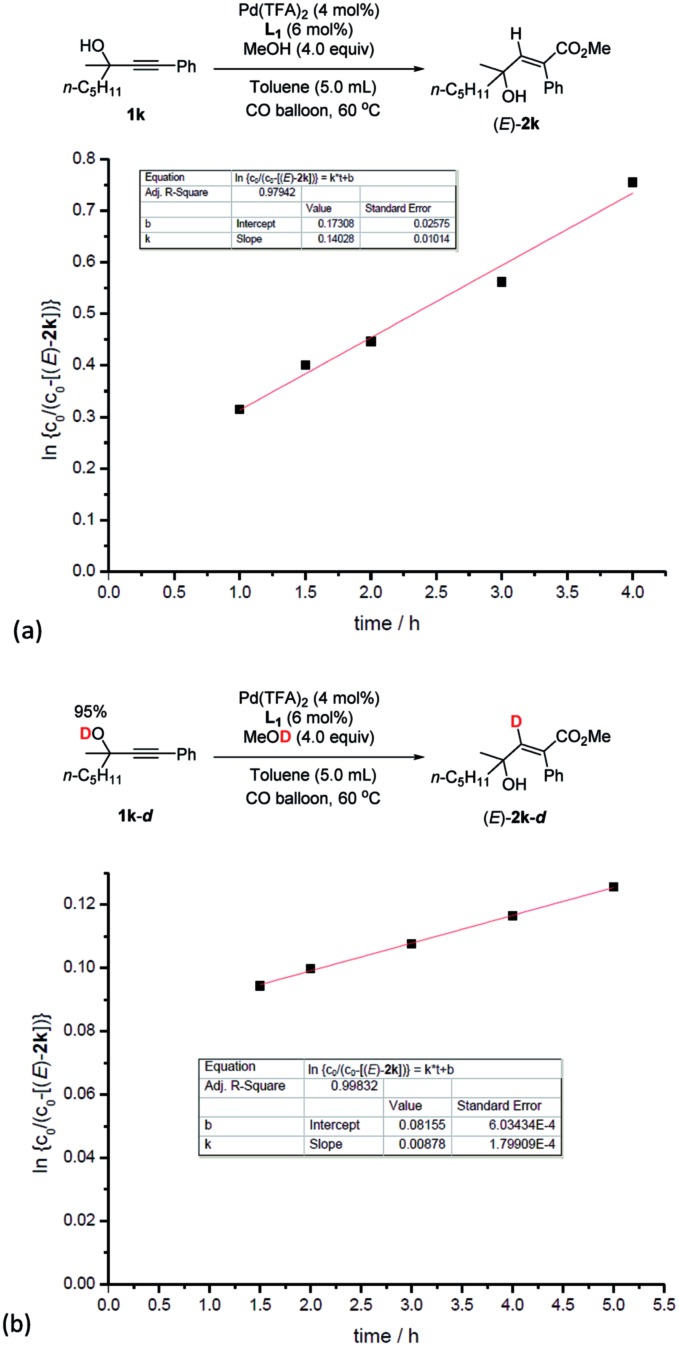
Kinetic isotope effect experiments. (a) Linear function fit for the reaction rate of **1k** to obtain *k*_H_. (b) Linear function fit for the reaction rate of **1k-*d*** to obtain *k*_D_. *k*_H_/*k*_D_ = 16.

Based on this, parallel reactions of **1k** and **1k**-***d*** in separate reaction vessels monitored by ^1^H NMR analysis of the reaction profile could help determine the value of *k*_H_ and *k*_D_, and then the KIE was calculated to be *k*_H_/*k*_D_ = 16 (Fig. 5), indicating the primary isotope effect of H/D.

**Fig. 6:**
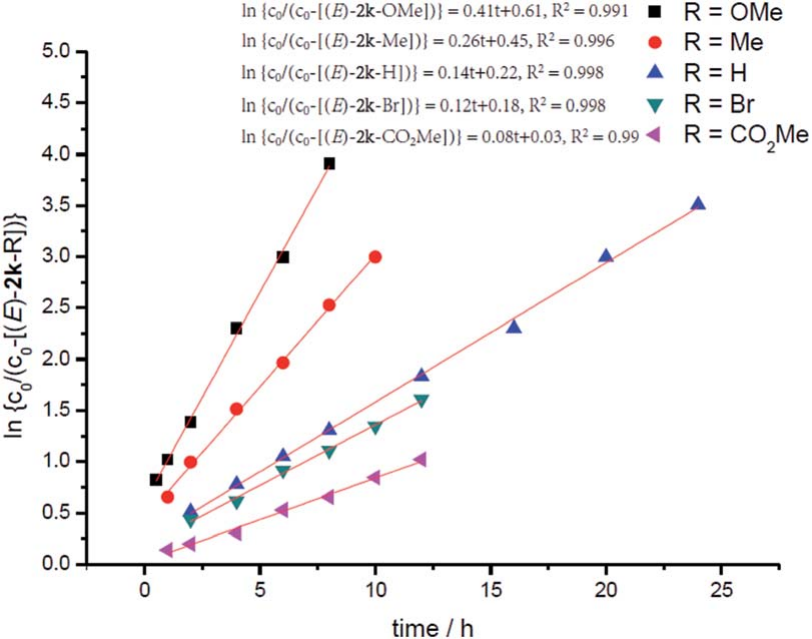
ln{*c*_0_/(*c*_0_ – [(*E*)-**2k**-*R*])} *vs.* time (*R*-squared is the coefficient of determination).

In order to further identify the rate-determining step, the electronic effect of substrates on the Pd–H insertion step was investigated (Table 7). Then, kinetic studies of the substrates with different substituents on the *para*-position of the phenyl ring such as Br, CO_2_Me, Me, and OMe were carried out. Linear relationships were obtained for ln{*c*_0_/(*c*_0_ – [(*E*)-**2k**])} *vs.* reaction time, and show significantly different reaction rates, that is, the more electron-rich the substituent is, the faster the reaction rate is (Fig. 6). These results also indicate that Pd–H insertion has a large effect on the reaction rate. However, we are still not able to exclude the oxidative addition of O–H with Pd as the rate-determining step.

**Table 7:** Electronic effect investigation

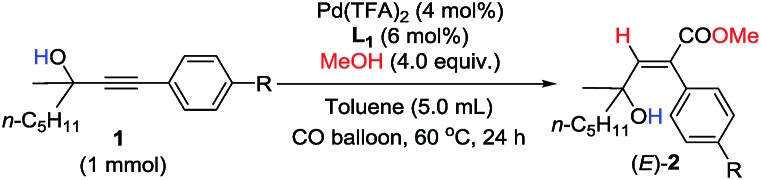		
Entry	R	Yield of (*E*)-**2**^*a*^/%
1	H (**1k**)	95 (**2k**)
2	Br (**1l**)	92 (**2l**)
3^*b*^	CO_2_Me (**1y**)	85 (**2y**)
4	Me (**1z-A**)	96 (**2z-A**)
5	OMe (**1z-B**)	97 (**2z-B**)

^*a*^Isolated yield.

^*b*^14% recovery of **1y** was detected.

In order to further unveil the mechanism, solvents without hydrogen bonding7*b* were also screened—lower yields were detected in comparison with the data for toluene. The stronger the polarity of the solvent is, the lower the yield would be, and nothing but a large amount of substrate recovery was observed when using DMSO, further supporting the irreplaceable role of hydrogen bonding in this transformation (Table 3, entries 10–14).

**Fig. 7:**
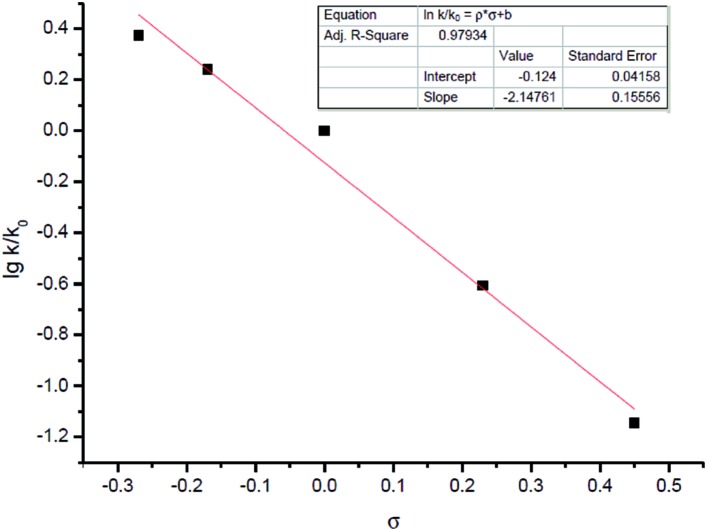
Hammett equation of phenols with varying acidities.

**Scheme 4:**
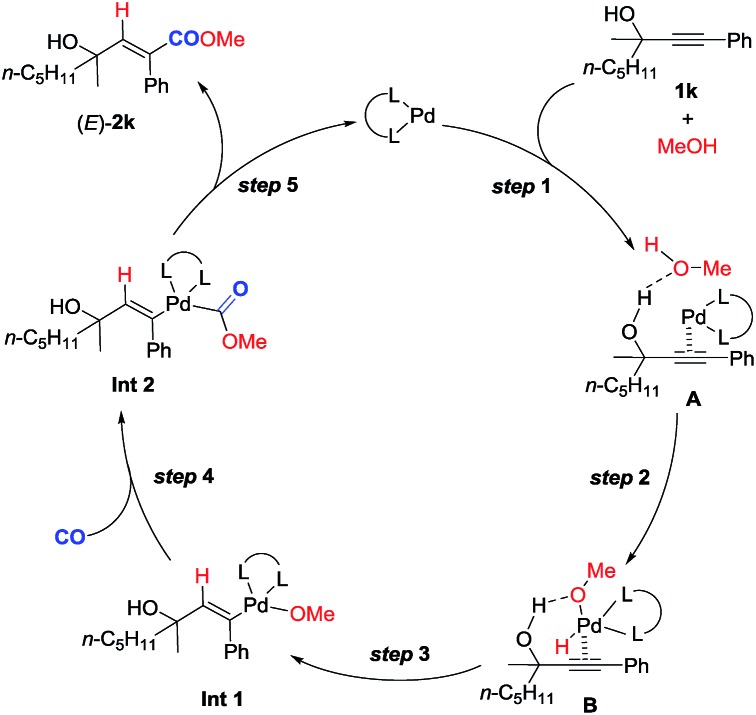
Plausible reaction mechanism.

Other than this, a Hammett study with phenols bearing various substituents has also been carried out (Table 8). The negative value for *ρ* points out that the rate-determining step favors phenols with electron-donating groups (Fig. 7).^15^ This seems reasonable to us because phenols with electron-donating groups would result in a higher electron density on the oxygen atom, thus leading to stronger hydrogen bonding with the hydrogen atom in the hydroxyl group and/or nucleophilicity (see *step* 3 in Scheme 4).

**Table 8:** Hammett study with phenols bearing various substituents^*a*^

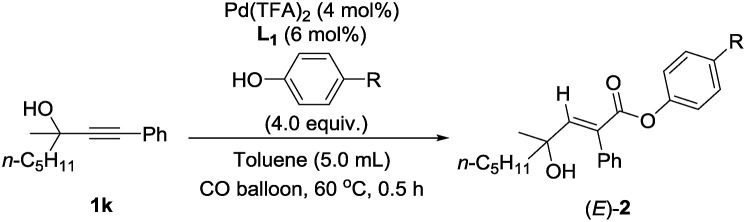			
Entry	R	*σ* (ref. 16)	Yield of (*E*)-**2**/%
1	H	0	11 (**2kd**)
2	MeO_2_C	0.45	3.5 (**2ke**)
3	Me	–0.17	14 (**2kf**)
4	MeO	–0.27	16 (**2kg**)
5	Cl	0.23	6 (**2kh**)

^*a*^Yield and recovery were determined by ^1^H-NMR analysis using CH_2_Br_2_ as the internal standard.

Based on these studies, a plausible mechanism is proposed (Scheme 4). Hydrogen bonding between the hydroxyl group of methanol and that of 2-alkynol combined with the coordination of the C–C triple bond to the Pd^0^ species would form complex **A**. Subsequent oxidative addition of the O–H bond in methanol with Pd^0^ in **A** affords complex **B**. Subsequent regioselective *syn*-hydropalladation of the C–C triple bond delivers the H atom to the sp carbon atom closer to the hydroxy group in **1k**, and then nucleophilic attack of CO by the methoxy anion generates **Int 2**. Reductive elimination would then furnish (*E*)-**2k** and regenerate the Pd^0^ species to finish the catalytic cycle. Of course, further studies are needed to fully verify this mechanism.

## Conclusions

In summary, we have developed hydroxy group-enabled highly regio- and stereo-selective hydrocarboxylation of 2-alkynylic alcohols, exploiting a previously unrecognized regioselectivity control strategy. The remarkable substrate scope, atom economy, and good to excellent yields make this reaction a facile synthetic method to produce highly functionalized 3-hydroxy-2(*E*)-alkenoates and the observed regioselectivity may arise from hydrogen bonding, which needs further investigation. Due to the versatility of the functionality in the products, the importance of the stereo-selective construction of C=C bonds, and the nature of regio-selectivity control, this method will be of high interest to organic and medicinal chemists. Further studies in this area are currently ongoing in our laboratory.

## Conflicts of interest

There are no conflicts to declare.

## Supplementary Material

Supplementary information

Crystal structure data
