# Supplementary material for: Hydroxy group-enabled highly regio- and stereo-selective hydrocarboxylation of alkynes
Source: Chem Sci. 2019 Apr 17;10(21):5505–12. doi: 10.1039/c8sc05743e (PMC6544123; doi:10.1039/c8sc05743e)
Supplement: Supplementary file 1 [file SC-010-C8SC05743E-s001.pdf]

Electronic Supplementary Material (ESI) for Chemical Science.
This journal is © The Royal Society of Chemistry 2019

Hydroxy Group-Enabled Highly Regio- and Stereo-selective Hydrocarboxylation of Alkynes

Chaofan Huang,[a] Hui Qian,[a] Wanli Zhang,[a*] and Shengming Ma[a,b*]

[a] Research Center for Molecular Recognition and Synthesis, Department of Chemistry, Fudan University, 220 Handan Lu, Shanghai 200433, P. R. China
[b] State Key Laboratory of Organometallic Chemistry, Shanghai Institute of Organic Chemistry, Chinese Academy of Sciences, 345 Lingling Lu, Shanghai 200032, P. R. China

General information	S2
Screening of palladium catalyst, ligand, reaction temperature and solvent	S3-S5
Experimental details and analytical data	S6-S41
Synthetic applications	S41-S44
Mechanistic studies	S44-S63
References	S64
^{1}H, ^{13}C, ^{19}F NMR, and HPLC spectra of the products	S64-S230

General Information. NMR spectra were taken with an Agilent-400 spectrometer (400 MHz for ^{1}H NMR, 100 MHz for ^{13}C NMR, 376 MHz for ^{19}F NMR) in $CDCl_3$ or d_6-DMSO. All ^{1}H NMR experiments were measured with tetramethylsilane (0 ppm) in $CDCl_3$ or the signal of residual DMSO (2.50 ppm) in d_6-DMSO as the internal reference; ^{13}C NMR experiments were measured in relative to the signal of $CDCl_3$ (77.0 ppm) or the signal of d_6-DMSO (39.52 ppm); ^{19}F NMR experiments were measured in relative to the signal of $CFCl_3$ (0 ppm) in $CDCl_3$. All reactions were carried out in flame-dried Schlenk tubes. $Pd(TFA)_2$ was purchased from J&K Chemicals, Meryer Chemical Technology CO., Ltd, and Macklin Biochemical Co., Ltd; DPEphos was purchased from Acros Organics or Energy Chemical; 2,2'-Bis(dicyclohexylphosphino)-1,1'-biphenyl was purchased from Strem Chemicals, Inc.; $(PhO)_2POOH$ was purchased from Energy Chemical and purified through stirring with 1 N HCl, extracting with dichloromethane, and removing solvents under vacuum; MeOH was purchased from Sinopharm Chemical Reagent Co., Ltd; ^{n}BuLi was purchased from Energy Chemical and Infinity Scientific (Beijing) Co. Ltd. Petroleum ether (b.p. 60-90 ^{o}C) was purchased from Shanghai Titan Scientific Co., Ltd. Toluene was dried over sodium wire with benzophenone as the indicator and distilled freshly before use. THF was dried over sodium wire. The reaction should be conducted in a hood working efficiently due to the toxicity of CO gas. All the temperatures are referred to the oil baths used. Recoveries of substrates were determined by ^{1}H NMR analysis using dibromomethane as the internal standard. The starting propargylic alcohols were synthesized according to the reported procedures. [1]

Table S1 Screening of palladium catalyst and ligand for substrate 1a [a]

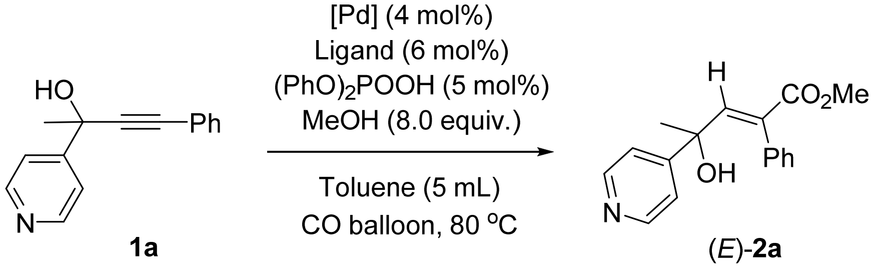

Entry	[Pd]	Ligand	NMR yield of (*E*)-**2a** (%) [b]	Recovery of **1a** (%) [b]
1	[PdCl(π-allyl)]$_2$	DPEphos	66	25
2	$Pd_2(dba)_3$	DPEphos	57	34
3	Pd(*t*-Bu$_2$-PPh)$_2$	DPEphos	78	12
4	$Pd(PPh_3)_4$	DPEphos	82	15
5	$Pd(PPh_3)_2Cl_2$	DPEphos	-	99
6	$Pd(TFA)_2$	DPEphos	82	6
7	$Pd(OAc)_2$	DPEphos	70	4
8	$PdCl_2$	DPEphos	2	86
9	$PdBr_2$	DPEphos	5	95
10	[PdCl(π-cinamyl)]$_2$	DPEphos	46	44
11	$Pd(acac)_2$	DPEphos	59	25
12	$Pd(TFA)_2$	BINAP	26	67
13	$Pd(TFA)_2$	DPPB	33	43
14	$Pd(TFA)_2$	**L$_1$**	32	69
15	$Pd(TFA)_2$	**L$_2$**	6	54

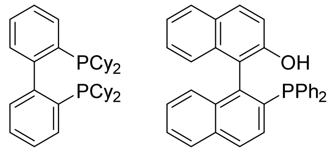

L$_1$ L$_2$

[a] **Condition A**: **1a** (1.0 mmol), [Pd] (4 mol%), ligand (6 mol%), $(PhO)_2POOH$ (5 mol%), MeOH (8.0 mmol), CO balloon, toluene (5 mL), 80 °C, 16 h. [b] Determined by ^{1}H NMR analysis using CH_2Br_2 as the internal standard.

Table S2 Screening of temperature [a]

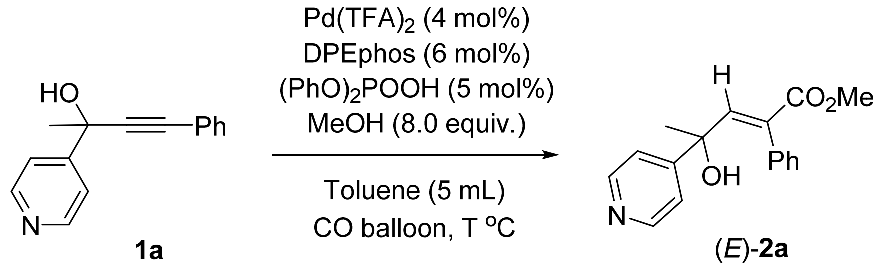

Entry	T / °C	NMR yield of (*E*)-**2a** (%) [b]	Recovery of **1a** (%) [b]
1	80	82	6
2	85	9	68
3	70	90	-
4	60	90	-
5	50	45	53
6 [c]	60	89	-

[a] **Condition A**: **1a** (1.0 mmol), $Pd(TFA)_2$ (4 mol%), DPEphos (6 mol%), $(PhO)_2POOH$ (5 mol%), MeOH (8.0 mmol), CO balloon, toluene (5 mL), 80 °C, 16 h. [b] Determined by ^1H NMR analysis using CH_2Br_2 as the internal standard. [c] The reaction was carried out without $(PhO)_2POOH$.

Table S3 Screening of palladium catalyst and ligand for substrate 1k [a]

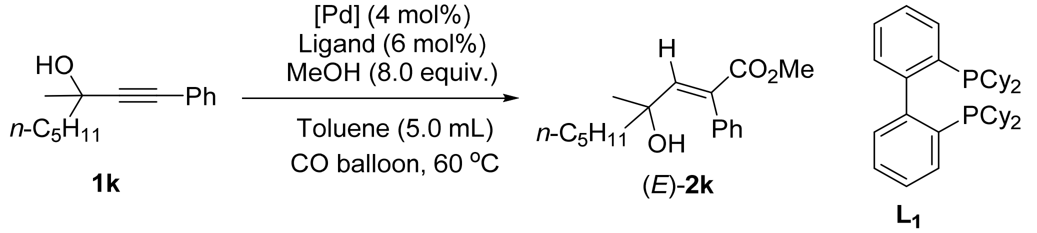

Entry	[Pd]	Ligand	T / °C	NMR yield of (*E*)-**2k** (%) [b]	Recovery of **1k** (%) [b]
1 [c]	$Pd(TFA)_2$	DPEphos	60	35	-
2 [c]	$Pd(TFA)_2$	DPEphos	50	49	-
3 [c]	$Pd(TFA)_2$	DPEphos	40	28	48
4	$PdCl_2$	DPEphos	50	-	93
5	$Pd(OAc)_2$	DPEphos	50	4	97
6	$Pd(PPh_3)_4$	DPEphos	50	-	96
7	$Pd(TFA)_2$	PPh_3	60	4	90
8	$Pd(TFA)_2$	Zhedaphos	50	1	100
9	$Pd(TFA)_2$	Sphos	50	11	88
10	$Pd(TFA)_2$	BINAP	50	40	44
11	$Pd(TFA)_2$	Xantphos	50	10	70
12	$Pd(TFA)_2$	DPPB	50	17	66
13	$Pd(TFA)_2$	DPPP	50	20	67
14	$Pd(TFA)_2$	DPPF	50	2	94
15	$Pd(TFA)_2$	$\mathbf{L_1}$	50	58	46
16	$Pd(TFA)_2$	BIPHEP	50	5	96
17 [c,d]	$Pd(TFA)_2$	$\mathbf{L_1}$	50	79	18
18 [c,d]	$Pd(TFA)_2$	$\mathbf{L_1}$	60	95	trace
19 [c,d,e]	$Pd(TFA)_2$	$\mathbf{L_1}$	60	77	trace
19 [c,d,f]	$Pd(TFA)_2$	$\mathbf{L_1}$	60	99(95)	-

[a] **Conditions B**: **1k** (0.4 mmol), [Pd] (4 mol%), ligand (6 mol%), MeOH (8.0 mmol), CO balloon, toluene (5.0 mL), 16 h. [b] Yield and recovery were determined by ^{1}H NMR analysis using CH_2Br_2 as the internal standard, and the isolated yield is shown in the parentheses. [c] The reaction scale was 1.0 mmol. [d] The reaction time was extended to 24 h. [e] The reaction was carried out with 16.0 mmol of MeOH. [f] The reaction was carried out with 4.0 mmol of MeOH.

Experimental details and analytical data

1. Synthesis of propargylic alcohols

(1) Preparation of 4-phenyl-2-(quinolin-3-yl)but-3-yn-2-ol (1d) [1] (hcf-1-185)

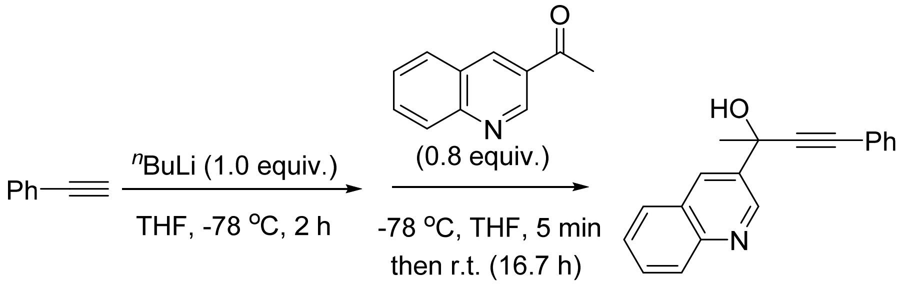

1d, 59%

Typical Procedure I: To an oven-dried flask (100 mL) were added phenylacetylene (2.80 mL, d = 0.93 g/mL, 2.6040 g, 25 mmol) and THF (50 mL). Then a solution of ^{n}BuLi (2.5 M in hexane, 10 mL, 25 mmol) was added dropwise at -78 °C over 5 min. under argon. After being stirred at -78 °C for 2 h, a solution of 1-(quinolin-3-yl)ethan-1-one (3.5298 g, 20 mmol) in THF (10 mL) was added dropwise over 5 min. The resulting mixture was warmed up to room temperature, stirred for 16.7 h, and quenched with a saturated aqueous solution of NH_4Cl (10 mL). After extraction with ethyl acetate (40 mL x 3), the organic layer was dried over anhydrous Na_2SO_4. After filtration and concentration under reduced pressure, the crude product was purified by column chromatography on silica gel to afford **1d** (3.3012 g, 59%) [eluent: dichloromethane/ethyl acetate = 20/1 (1050 mL), 10/1 (1100 mL), 1/1 (1000 mL)]: brown solid, m.p. 135.6-136.1 ℃ (petroleum ether/dichloromethane); **^{1}H NMR** (400 MHz, $CDCl_3$): δ = 9.24 (s, 1 H, Ar-H), 8.44 (s, 1 H, Ar-H), 8.12 (d, J = 8.0 Hz, 1 H, Ar-H), 7.80 (d, J = 6.4 Hz, 1 H, Ar-H), 7.73-7.59 (m, 1 H, Ar-H), 7.59-7.47 (m, 1 H, Ar-H), 7.47-7.38 (m, 2 H, Ar-H), 7.38-7.18 (m, 3 H, Ar-H), 4.48-3.88 (br, 1 H, OH), 1.95 (s, 3 H, CH_3); **^{13}C NMR** (100 MHz, $CDCl_3$): δ = 148.8, 147.0, 138.6, 131.7, 129.5, 128.7, 128.6, 128.3, 128.1, 127.4, 126.9, 122.1, 91.7, 85.6, 68.7, 33.2; **MS** (70 eV, EI) m/z (%): 274 (M^++1, 13.85), 273 (M^+, 60.93), 230 (100); **IR** (neat): ν = 3111, 2990, 2931, 2813, 2232, 1574, 1494, 1421, 1177, 1093, 1056 cm^{-1}; Anal. Calcd. for $C_{19}H_{15}NO$: C 83.49, H 5.53, N 5.12; found C 83.49, H

5.60, N 5.20.

(2) Preparation of 2-(pyridin-4-yl)-4-(*p*-tolyl)but-3-yn-2-ol (1e) [1] (hcf-1-146)

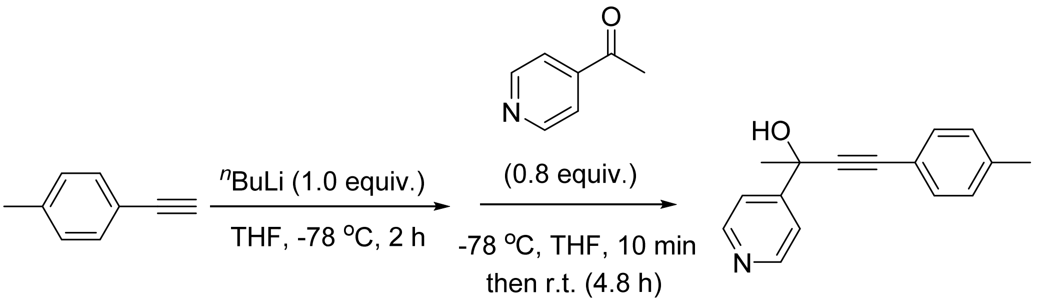

1e, 65%

Following Typical Procedure I, the reaction of 4-methylphenylacetylene (1.4825 g, 12.5 mmol)/THF (30 mL), ^{n}BuLi (2.5 M in hexane, 5 mL, 12.5 mmol), and 1-(pyridin-4-yl)ethan-1-one (1.11 mL, d = 1.095 g/mL, 1.2154 g, 10 mmol)/THF (30 mL) afforded **1e** (1.5424 g, 65%): [eluent: petroleum ether/ethyl acetate = 3/1 (1000 mL), 1/1 (1000 mL), then recrystallization (petroleum ether/ethyl acetate)]: white solid, m.p. 151.2-151.9 ℃ (petroleum ether/ethyl acetate); **^{1}H NMR** (400 MHz, $CDCl_3$): δ = 8.59-8.51 (m, 2 H, Ar-H), 7.65-7.57 (m, 2 H, Ar-H), 7.33 (d, *J* = 7.6 Hz, 2 H, Ar-H), 7.12 (d, *J* = 8.0 Hz, 2 H, Ar-H), 4.35-4.05 (br, 1 H, OH), 2.35 (s, 3 H, CH_3), 1.82 (s, 3 H, CH_3); **^{13}C NMR** (100 MHz, $CDCl_3$): δ = 155.2, 149.5, 138.9, 131.6, 129.1, 120.1, 119.0, 90.6, 85.4, 69.2, 33.1, 21.5; **MS** (70 eV, EI) *m/z* (%): 238 (M^++1, 3.00), 237 (M^+, 17.78), 222 (100); **IR** (neat): *ν* = 3154, 3036, 2976, 2918, 2857, 2235, 1600, 1422, 1142, 1001 cm^{-1}; Anal. Calcd. for $C_{16}H_{15}NO$: C 80.98, H 6.37, N 5.90; found C 80.85, H 6.38, N 5.82.

(3) Preparation of 2-(pyridin-4-yl)oct-3-yn-2-ol (1f) [1] (hcf-2-147)

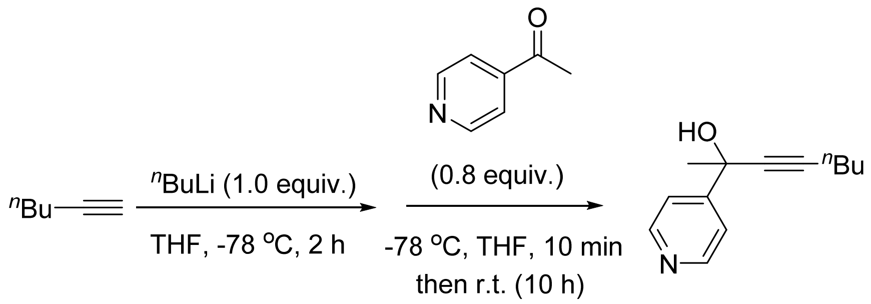

1f, 32%

Following Typical Procedure I, the reaction of 1-hexyne (1.48 mL, d = 0.715

g/mL, 1.0582 g, 12.5 mmol)/THF (30 mL), ^{n}BuLi (2.5 M in hexane, 5 mL, 12.5 mmol), and 1-(pyridin-4-yl)ethan-1-one (1.11 mL, d = 1.095 g/mL, 1.2045 g, 12.5 mmol)/THF (10 mL) afforded **1f** (0.6505 g, 32%) [eluent: petroleum ether/ethyl acetate = 3/1 (1000 mL), 1/1 (1000 mL), then recrystallization (petroleum ether/dichloromethane)]: yellow solid, m.p. 60.9-61.6 ℃ (petroleum ether/dichloromethane); **^{1}H NMR** (400 MHz, $CDCl_3$): δ = 8.51-8.38 (m, 2 H, Ar-H), 7.62-7.52 (m, 2 H, Ar-H), 5.69 (brs, 1 H, OH), 2.24 (t, J = 6.8 Hz, 2 H, CH_2), 1.71 (s, 3 H, CH_3), 1.57-1.29 (m, 4 H, 2 x CH_2), 0.89 (t, J = 7.0 Hz, 3 H, CH_3); **^{13}C NMR** (100 MHz, $CDCl_3$): δ = 155.8, 149.2, 120.2, 85.9, 82.8, 68.7, 33.4, 30.6, 21.9, 18.3, 13.5; **MS** (70 eV, EI) m/z (%): 203 (M^+, 1.90), 188 (100); **IR** (neat): ν = 3088, 3052, 2957, 2928, 2868, 2239, 1602, 1414, 1185, 1113, 1005 cm^{-1}; Anal. Calcd. for $C_{13}H_{17}NO$: C 76.81, H 8.43, N 6.89; found C 76.72, H 8.30, N 6.92.

(4) Preparation of 2-(pyridin-4-yl)dec-3-yn-2-ol (1g)[1] (hcf-2-11)

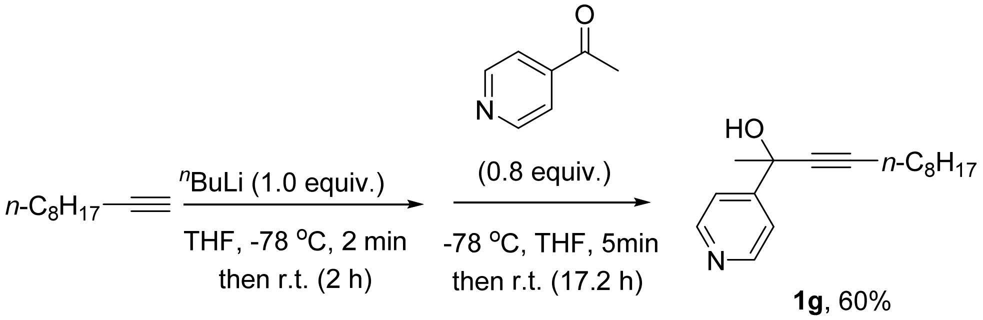

1g, 60%

Typical Procedure II: To an oven-dried flask (100 mL) were added 1-decyne (2.30 mL, d = 0.767 g/mL, 1.7641 g, 12.5 mmol) and THF (20 mL). Then a solution of ^{n}BuLi (2.5 M in hexane, 5 mL, 12.5 mmol) was added dropwise at -78 °C over 2 min. under argon. Then the reaction mixture was warmed up to room temperature. After stirring at room temperature for 2 h, the mixture was cooled to -78 °C, then a solution of 1-(quinolin-3-yl)ethan-1-one (3.5298 g, 20 mmol) in THF (10 mL) was added dropwise over 5 min. The resulting mixture was warmed up to room temperature, stirred for 17.2 h, and quenched with a saturated aqueous solution of NH_4Cl (10 mL). After extraction with ethyl acetate (40 mL x 3), the organic layer was dried over anhydrous Na_2SO_4. After filtration and concentration under reduced pressure, the crude product was purified by column chromatography on silica gel to afford **1g**

(1.5598 g, 60%) as an oil [eluent: dichloromethane/ethyl acetate = 5/1 (600 mL), 3/1 (400 mL), 1/1 (200 mL)]: **^{1}H NMR** (400 MHz, $CDCl_3$): δ = 8.55 (d, J = 6.0 Hz, 2 H, Ar-H), 7.54 (dd, J_1 = 4.8 Hz, J_2 = 1.60 Hz, 2 H, Ar-H), 3.29 (brs, 1 H, OH), 2.26 (t, J = 7.0 Hz, 2 H, CH_2), 1.71(s, 3 H, CH_3), 1.59-1.47 (m, 2 H, CH_2), 1.44-1.19 (m, 10 H, 5 x CH_2), 0.88 (t, J = 6.8 Hz, 3 H, CH_3); **^{13}C NMR** (100 MHz, $CDCl_3$): δ = 155.9, 149.2, 120.2, 86.0, 82.8, 68.6, 33.3, 31.7, 29.1, 29.0, 28.8, 28.5, 22.6, 18.6, 14.0; **MS** (70 eV, EI) *m/z* (%): 259 (M^+, 2.46), 244 (100); **IR** (neat): *v* = 3151, 2924, 2854, 2243, 1600, 1463, 1413, 1104, 1064, 1004 cm^{-1}; **HRMS** calcd for $C_{17}H_{25}NO$ [M^+]: 259.1936, found: 259.1934.

(5) Preparation of 1-phenyl-3-(pyridin-4-yl)pent-1-yn-3-ol (1h) [1] (hcf-1-164)

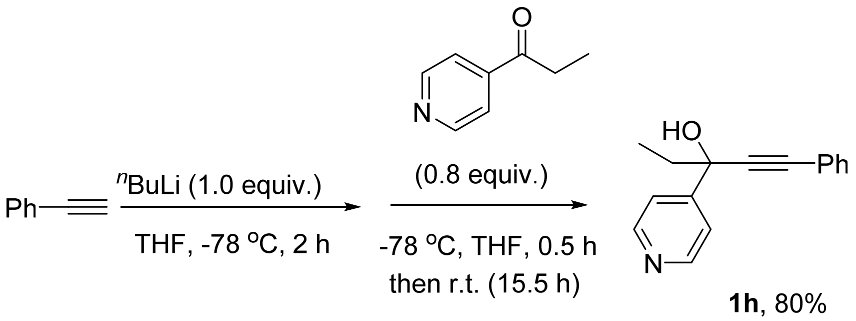

Following Typical Procedure I, the reaction of phenylacetylene (5.60 mL, d = 0.93 g/mL, 5.2080 g, 50 mmol)/THF (50 mL), ^{n}BuLi (2.5 M in hexane, 20 mL, 50 mmol), and 1-(pyridin-4-yl)propan-1-one (5.33 mL, d = 1.034 g/mL, 5.5112 g, 40 mmol)/THF (20 mL) afforded **1h** (7.6483 g, 80%) [recrystallization (petroleum ether/dichloromethane)]: brown solid: m.p. 108.6-110.1 ℃ (petroleum ether/dichloromethane); **^{1}H NMR** (400 MHz, $CDCl_3$): δ = 8.49 (d, J = 2.4 Hz, 2 H, Ar-H), 7.65-7.52 (m, 2 H, Ar-H), 7.48-7.38 (m, 2 H, Ar-H), 7.38-7.27 (m, 3 H, Ar-H), 5.08 (brs, 1 H, OH), 2.11-1.89 (m, 2 H, CH_2), 1.02 (t, J = 7.4 Hz, 3 H, CH_3); **^{13}C NMR** (100 MHz, $CDCl_3$): δ = 154.4, 149.1, 131.7, 128.6, 128.3, 122.2, 120.8, 90.4, 86.2, 72.8, 38.2, 8.8; **MS** (70 eV, EI) *m/z* (%): 237 (M^+, 1.61), 208 (100); **IR** (neat): *v* = 3128, 3056, 2969, 2931, 2872, 2223, 1600, 1453, 1429, 1130, 1070, 1002 cm^{-1}; Anal. Calcd. for $C_{16}H_{15}NO$: C 80.98, H 6.37, N 5.90; found C 80.92, H 6.51, N 6.09.

(6) Preparation of 1-phenyl-1-(pyridin-4-yl)hept-2-yn-1-ol (1i) [1] (hcf-1-188)

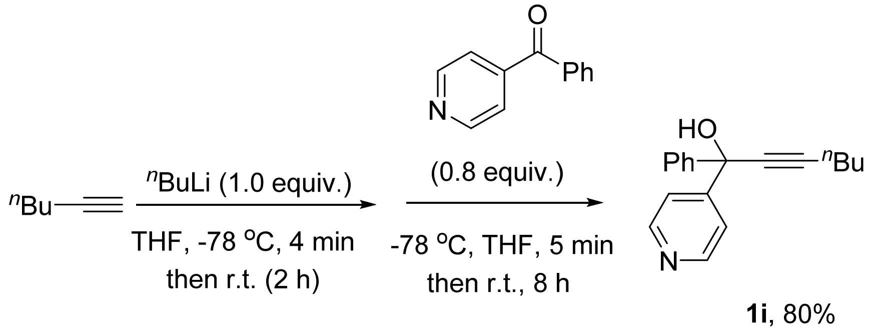

1i, 80%

Following Typical Procedure II, the reaction of 1-hexyne (2.96 mL, d = 0.715 g/mL, 2.1164 g, 25 mmol)/THF (50 mL), ^{n}BuLi (2.5 M in hexane, 10 mL, 25 mmol), and phenyl(pyridin-4-yl)methanone (3.7020 g, 20 mmol)/THF (10 mL) afforded **1i** (4.2988 g, 80%) [recrystallization (petroleum ether/ethyl acetate)]: white solid, m.p. 123.5-124.0 ℃ (petroleum ether/ethyl acetate); **^{1}H NMR** (400 MHz, $CDCl_3$): δ = 8.45 (d, *J* = 6.4 Hz, 2 H, Ar-H), 7.59 (d, *J* = 6.8 Hz, 2 H, Ar-H), 7.50 (d, *J* = 6.0 Hz, 2 H, Ar-H), 7.38-7.21 (m, 3 H, Ar-H), 4.18 (s, 1 H, OH), 2.32 (t, *J* = 7.0 Hz, 2 H, CH_2), 1.61-1.49 (m, 2 H, CH_2), 1.48-1.35 (m, 2 H, CH_2), 0.91(t, *J* = 7.2 Hz, 3 H, CH_3); **^{13}C NMR** (100 MHz, $CDCl_3$): δ = 155.0, 149.1, 144.5, 128.3, 127.8, 125.9, 120.8, 88.7, 82.0, 73.2, 30.5, 21.9, 18.5, 13.5; **MS** (70 eV, EI) *m/z* (%): 266 (M^++1, 2.87), 265 (M^+, 9.93), 208 (100); **IR** (neat): *v* = 3081, 3052, 2961, 2930, 2873, 2830, 2240, 1599, 1449, 1145, 1042, 1003 cm^{-1}; Anal. Calcd. for $C_{18}H_{19}NO$: C 81.47, H 7.22, N 5.28; found C 81.40, H 7.42, N 5.27.

(7) Preparation of 2-(4-nitrophenyl)dec-3-yn-2-ol (1j)[1] (hcf-2-52)

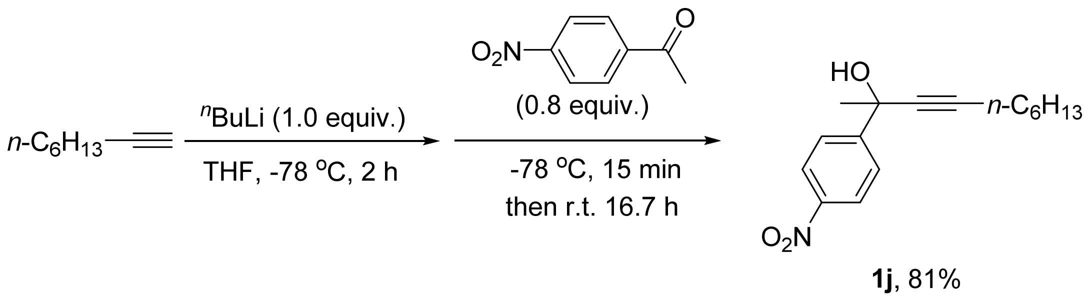

1j, 81%

Following Typical Procedure I, the reaction of 1-octyne (3.0 mL, d = 0.746 g/mL, 2.2380 g, 20 mmol)/THF (20 mL), ^{n}BuLi (2.5 M in hexane, 8.0 mL, 20 mmol), and 1-(4-nitrophenyl)ethan-1-one (2.6950 g, 16 mmol)/THF (10 mL) afforded **1j** (2.4635 g, 81%) as a yellow oil [eluent: petroleum ether 200 mL, petroleum ether

/ethyl acetate = 20/1 (800 mL)]: **^{1}H NMR** (400 MHz, $CDCl_3$): δ = 8.20 (d, *J* = 8.8 Hz, 2 H, Ar-H), 7.82 (d, *J* = 8.8 Hz, 2 H, Ar-H), 2.46 (s, 1 H, OH), 2.27 (t, *J* = 7.0 Hz, 2 H, CH_2), 1.75 (s, 3 H, CH_3), 1.59-1.49 (m, 2 H, CH_2), 1.45-1.24 (m, 6 H, 3 x CH_2), 0.90 (t, *J* = 6.8 Hz, 3 H, CH_3); **^{13}C NMR** (100 MHz, $CDCl_3$): δ = 153.3, 147.2, 126.0, 123.5, 86.9, 82.6, 69.6, 33.8, 31.2, 28.5, 28.4, 22.5, 18.6, 14.0; **MS** (ESI) *m/z*: 298 ($M+Na^+$), 258 $(M-OH)^+$; **IR** (neat): *v* = 3415, 2929, 2858, 2244, 1603, 1518, 1343, 1087, 1014 cm^{-1}; **HRMS** calcd for $C_{16}H_{22}NO_3$ [$M+H^+$]: 276.1594, found: 276.1593.

(8) Preparation of 1-(4-bromophenyl)-3-methyloct-1-yn-3-ol (1l)[1] (hcf-2-56)

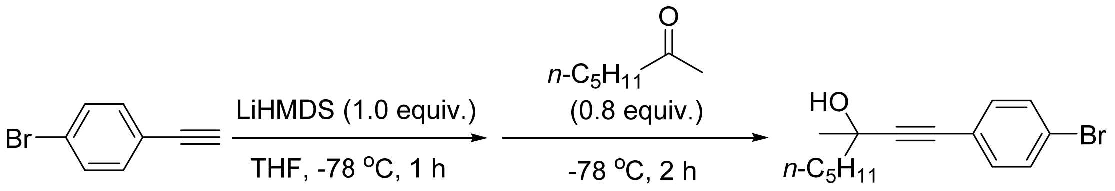

1l, 75%

Following Typical Procedure I, the reaction of 4-bromophenylacetylene (3.7341 g, 20 mmol)/THF (30 mL), LiHMDS (1.0 M in tetrahydrofuran, 20 mL, 20 mmol), and heptan-2-one (2.25 mL, d = 0.82 g/mL, 1.8450 g, 16 mmol)/THF (10 mL) afforded **1l** (3.5417 g, 75%) as a yellow oil [eluent: petroleum ether (200 mL), petroleum ether /ethyl acetate = 20/1 (1050 mL)]: **^{1}H NMR** (400 MHz, $CDCl_3$): δ = 7.43 (d, *J* = 8.4 Hz, 2 H, Ar-H), 7.27 (d, *J* = 8.4 Hz, 2 H, Ar-H), 2.03 (s, 1 H, OH), 1.80-1.67 (m, 2 H, CH_2), 1.63-1.48 (m, 5 H, CH_2 and CH_3), 1.43-1.27 (m, 4 H, 2 x CH_2), 0.91 (t, *J* = 6.8 Hz, 3 H, CH_3); **^{13}C NMR** (100 MHz, $CDCl_3$): δ = 133.0, 131.5, 122.4, 121.7, 94.1, 82.2, 68.6, 43.6, 31.8, 29.7, 24.4, 22.5, 14.0; **MS** (70 eV, EI) *m/z* (%): 296 (M^+ (^{81}Br), 4.75), 294 (M^+ (^{79}Br), 5.02), 223 (100); **IR** (neat): *v* = 3349, 2954, 2931, 2860, 1485, 1462, 1370, 1128, 1070, 1010 cm^{-1}; **HRMS** calcd for $C_{15}H_{19}{}^{79}BrO$ [M^+]: 294.0619, found: 294.0617.

(9) Preparation of 1-(methoxycarbonylphenyl)-3-methyloct-1-yn-3-ol (1y)[1] (hcf-3-138)

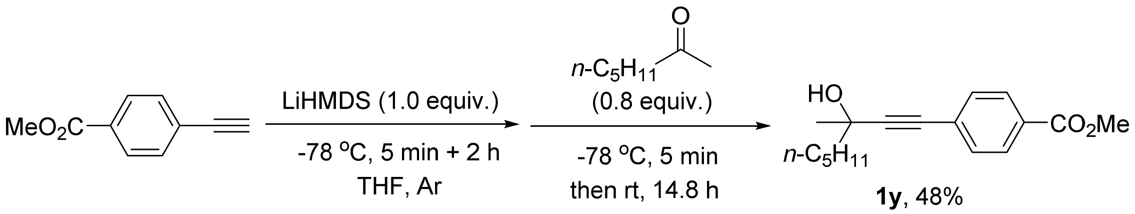

Following Typical Procedure I, the reaction of methyl 4-ethynylbenzoate (3.3029 g, 20 mmol)/THF (30 mL), LiHMDS (1.3 M in tetrahydrofuran, 15.4 mL, 20 mmol), and heptan-2-one (2.3 mL, d = 0.82 g/mL, 1.886 g, 16 mmol)/THF (10 mL) afforded **1y** (2.1314 g, 48%) as a yellow oil [eluent: petroleum ether/ethyl acetate = 50/1 (510 mL), 10/1 (1100 mL), 5/1 (600 mL)]: **^{1}H NMR** (400 MHz, $CDCl_3$): δ = 7.97 (d, *J* = 8.4 Hz, 2 H, Ar-H), 7.46 (d, *J* = 8.8 Hz, 2 H, Ar-H), 3.92 (s, 3 H, CO_2Me), 2.18-2.02 (m, 1 H, OH), 1.82-1.68 (m, 2 H, CH_2), 1.67-1.46 (m, 5 H, CH_2 and CH_3), 1.43-1.26 (m, 4 H, 2 x CH_2), 0.92 (t, *J* = 6.8 Hz, 3 H, CH_3); **^{13}C NMR** (100 MHz, $CDCl_3$): δ = 166.5, 131.6, 129.5, 129.4, 127.6, 96.1, 82.5, 68.7, 52.2, 43.6, 31.9, 29.7, 24.4, 22.5, 14.0; **MS** (70 eV, EI) *m/z* (%): 274 (M^+, 1.53), 203 (100); **IR** (neat): *ν* = 3436, 2953, 2934, 2870, 1724, , 1605, 1436, 1273, 1175, 1107 cm^{-1}; **HRMS** calcd for $C_{17}H_{22}O_3$ $[M]^+$: 274.1569, found: 274.1573.

(10) Preparation of 1-(4-methylphenyl)-3-methyloct-1-yn-3-ol (1z-A) [1] (hcf-3-139)

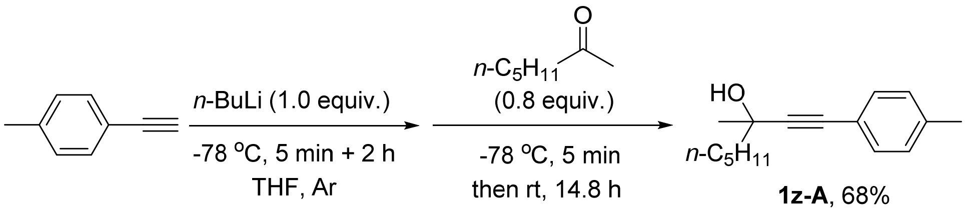

Following Typical Procedure I, the reaction of 4-methylphenylacetylene (2.6 mL, d = 0.916 g/mL, 2.3816 g, 20 mmol)/THF (30 mL), ^{n}BuLi (2.5 M in tetrahydrofuran, 8 mL, 20 mmol), and heptan-2-one (2.3 mL, d = 0.82 g/mL, 1.886 g, 16 mmol)/THF (10 mL) afforded **1z-A** (2.5351 g, 68%) as a yellow oil [eluent: petroleum ether/ethyl acetate = 50/1 (510 mL), 20/1 (1050 mL), 10/1 (550 mL)]: **^{1}H NMR** (400 MHz, $CDCl_3$): δ = 7.30 (d, *J* = 8.0 Hz, 2 H, Ar-H), 7.10 (d, *J* = 8.0 Hz, 2 H, Ar-H), 2.34 (s, 3 H, CH_3), 2.03-1.96 (m, 1 H, OH), 1.79-1.66 (m, 2 H, CH_2), 1.62-1.46 (m, 5 H, CH_2

and CH_3), 1.41-1.26 (m, 4 H, 2 x CH_2), 0.91 (t, J = 7.0 Hz, 3 H, CH_3); ^{13}**C NMR** (100 MHz, $CDCl_3$): δ = 138.3, 131.5, 129.0, 119.7, 92.3, 83.3, 68.7, 43.8, 31.9, 29.9, 24.4, 22.6, 21.4, 14.0; **MS** (70 eV, EI) *m/z* (%): 231 (M^++1, 1.62), 230 (M^+, 3.97), 159 (100); **IR** (neat): ν = 3357, 2954, 2932, 2861, 1509, 1456, 1370, 1129, 1040 cm^{-1}; **HRMS** calcd for $C_{16}H_{22}O$ $[M]^+$: 230.1665, found: 230.1665.

(11) Preparation of 1-(4-methoxyphenyl)-3-methyloct-1-yn-3-ol (1z-B) [1] (hcf-3-140)

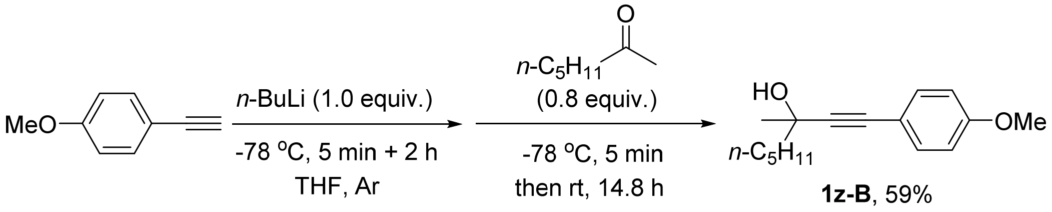

Following Typical Procedure I, the reaction of 4-methoxyphenylacetylene (2.6 mL, d = 1.019 g/mL, 2.6494 g, 20 mmol)/THF (30 mL), ^{n}BuLi (2.5 M in tetrahydrofuran, 8 mL, 20 mmol), and heptan-2-one (2.3 mL, d = 0.82 g/mL, 1.886 g, 16 mmol)/THF (10 mL) afforded **1z-B** (2.3524 g, 59%) as a yellow oil [eluent: petroleum ether/ethyl acetate = 50/1 (510 mL), 20/1 (1050 mL), 10/1 (550 mL)]: ^{1}**H NMR** (400 MHz, $CDCl_3$): δ = 7.35 (d, J = 8.8 Hz, 2 H, Ar-H), 6.82 (d, J = 8.8 Hz, 2 H, Ar-H), 3.80 (s, 3 H, OCH_3), 2.07-1.97 (m, 1 H, OH), 1.79-1.65 (m, 2 H, CH_2), 1.64-1.46 (m, 5 H, CH_2 and CH_3), 1.43-1.25 (m, 4 H, 2 x CH_2), 0.91 (t, J = 7.0 Hz, 3 H, CH_3); ^{13}**C NMR** (100 MHz, $CDCl_3$): δ = 159.5, 133.1, 114.9, 113.9, 91.6, 83.1, 68.7, 55.3, 43.8, 31.9, 29.9, 24.4, 22.6, 14.0; **MS** (70 eV, EI) *m/z* (%): 246 (M^+, 11.93), 175 (100); **IR** (neat): ν = 3393, 2955, 2933, 2870, 2227, 1606, 1509, 1465, 1288, 1245, 1031 cm^{-1}; **HRMS** calcd for $C_{16}H_{22}O_2$ $[M]^+$: 246.1614, found: 246.1621.

(12) Preparation of 4-methyltetradec-5-yn-4-ol (1n)[1] (zwl-11-164)

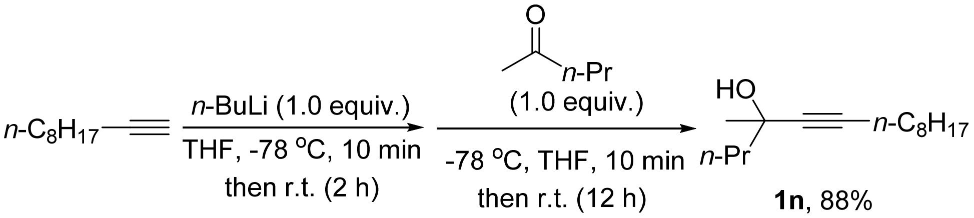

Following Typical Procedure II, the reaction of 1-decyne (4.50 mL, d = 0.766

g/mL, 3.4470 g, 25 mmol) in THF (30 mL), ^{n}BuLi (2.5 M in hexane, 10 mL, 25 mmol), and 2-pentanone (2.70 mL, d = 0.809 g/mL, 2.1843 g, 25 mmol) in THF (20 mL) afforded **1n** (4.1919 g, 88%) as an oil [eluent: petroleum ether/ethyl acetate = 10/1]: **^{1}H NMR** (400 MHz, $CDCl_3$): δ = 2.18 (t, *J* = 7.0 Hz, 2 H, CH_2), 1.97-1.85 (m, 1 H, OH), 1.65-1.57 (m, 2 H, CH_2), 1.57-1.42 (m, 7 H, CH_3 and 2 x CH_2), 1.42-1.20 (m, 10 H, 5 x CH_2), 0.96 (t, *J* = 7.2 Hz, 3 H, CH_3), 0.88 (t, *J* = 6.6 Hz, 3 H, CH_3); **^{13}C NMR** (100 MHz, $CDCl_3$): δ = 84.1, 83.7, 68.3, 46.2, 31.8, 30.1, 29.2, 29.0, 28.8, 28.7, 22.6, 18.6, 18.1, 14.2, 14.1; **MS** (ESI) *m/z*: 239 $(M+MeOH-OH)^+$, 225 $(M+H)^+$, 207 $(M-OH)^+$; **IR** (neat): *v* = 3375, 2926, 2856, 2236, 1461, 1370, 1131, 1051, 1012 cm^{-1}; **HRMS** calcd for $C_{15}H_{29}O$ $[M+H^+]$: 225.2213, found: 225.2212.

(13) Preparation of 5-methyldec-1-en-6-yn-5-ol (1o)[1] (zwl-11-166)

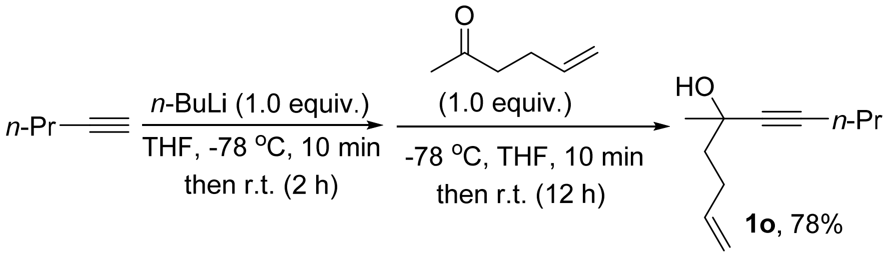

Following Typical Procedure II, the reaction of 1-pentyne (2.50 mL, d = 0.691 g/mL, 1.7275 g, 25 mmol) in THF (30 mL), ^{n}BuLi (2.5 M in hexane, 10 mL, 25 mmol), and 5-hexen-2-one (2.90 mL, d = 0.847 g/mL, 2.4563 g, 25 mmol) in THF (20 mL) afforded **1o** (3.2455 g, 78%) as an oil [eluent: petroleum ether/ethyl acetate = 10/1]: **^{1}H NMR** (400 MHz, $CDCl_3$): δ = 5.94-5.81 (m, 1 H, =CH), 5.07 (dd, J_1 = 17.2 Hz, J_2 = 1.6 Hz, 1 H, $=CH_2$), 4.97 (d, *J* = 10.4 Hz, 1 H, $=CH_2$), 2.39-2.20 (m, 2 H, CH_2), 2.18 (t, *J* = 7.0 Hz, 2 H, CH_2), 2.07-2.00 (m, 1 H, OH), 1.80-1.65 (m, 2 H, CH_2), 1.57-1.48 (m, 2 H, CH_2), 1.47 (s, 3 H, CH_3), 0.98 (t, *J* = 7.4 Hz, 3 H, CH_3); **^{13}C NMR** (100 MHz, $CDCl_3$): δ = 138.6, 114.6, 84.0, 83.8, 68.2, 42.9, 30.3, 29.3, 22.1, 20.5, 13.4; **MS** (ESI) *m/z*: 181 $(M+MeOH-OH)^+$, 149 $(M-OH)^+$; **IR** (neat): *v* = 3362, 2967, 2934, 2872, 2239, 1641, 1453, 1370, 1335, 1125, 1080 cm^{-1}; **HRMS** calcd for $C_{11}H_{19}O$ $[M+H^+]$: 167.1430, found: 167.1430.

(14) Preparation of 10-chloro-4-methyldec-5-yn-4-ol (1p)[1] (zwl-11-185)

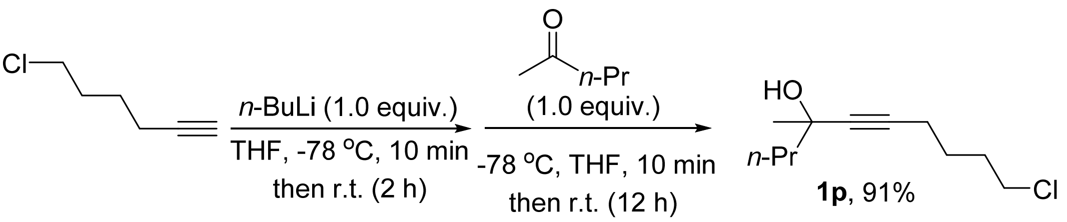

Following Typical Procedure II, the reaction of 6-chloro-1-hexyne (3.10 mL, d = 0.962 g/mL, 2.9822 g, 25 mmol) in THF (30 mL), ^{n}BuLi (2.5 M in hexane, 10 mL, 25 mmol), and 2-pentanone (2.70 mL, d = 0.809 g/mL, 2.1843 g, 25 mmol) in THF (20 mL) afforded **1p** (4.6781 g, 91%) as an oil [eluent: petroleum ether/ethyl acetate = 10/1]: **^{1}H NMR** (400 MHz, $CDCl_3$): δ = 3.57 (t, *J* = 6.6 Hz, 2 H, CH_2), 2.24 (t, *J* = 7.0 Hz, 2 H, CH_2), 2.16-1.98 (m, 1 H, OH), 1.95-1.81 (m, 2 H, CH_2), 1.72-1.38 (m, 9 H, CH_3 and 3 x CH_2), 0.95 (t, *J* = 7.2 Hz, 3 H, CH_3); **^{13}C NMR** (100 MHz, $CDCl_3$): δ = 84.8, 82.5, 68.1, 46.1, 44.4, 31.5, 30.0, 25.7, 18.0, 17.8, 14.1; **MS** (ESI) *m/z*: 219 $(M(^{37}Cl)+MeOH-OH)^+$, 217 $(M(^{35}Cl)+MeOH-OH)^+$; **IR** (neat): *ν* = 3419, 2957, 2934, 2872, 1455, 1369, 1333, 1316, 1131, 1051, 1011 cm^{-1}; **HRMS** calcd for $C_{11}H_{19}O^{35}Cl$ [M^+]: 202.1124, found: 202.1121.

(15) Preparation of 3-methyl-1-phenyltetradec-1-yn-3-ol (1u)[1] (hcf-2-71)

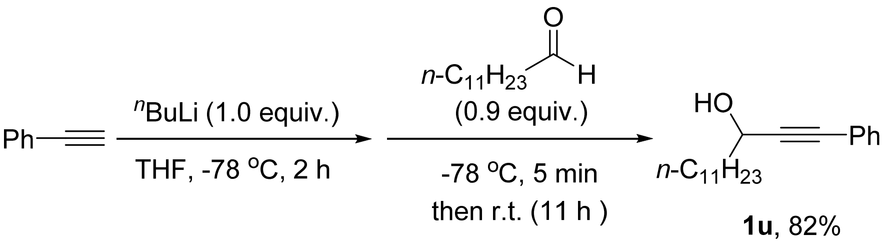

Following Typical Procedure I, the reaction of phenylacetylene (2.24 mL, d = 0.93 g/mL, 2.0832 g, 20 mmol)/THF (20 mL), ^{n}BuLi (2.5 M in hexane, 8.0 mL, 20 mmol), and dodecanal (4.1 mL, d = 0.831 g/mL, 3.4071 g, 18 mmol)/THF (10 mL) afforded **1u** (4.2117 g, 82%) as a yellow oil [eluent: petroleum ether/ethyl acetate = 20/1 (420 mL), 10/1 (880 mL)]: **^{1}H NMR** (400 MHz, $CDCl_3$): δ = 7.48-7.37 (m, 2 H, Ar-H), 7.35-7.25 (m, 3 H, Ar-H), 4.59 (q, *J* = 6.0 Hz, 1 H, CH), 2.23-2.10 (m, 1 H, OH), 1.86-1.71 (m, 2 H, CH_2), 1.58-1.42 (m, 2 H, CH_2), 1.42-1.18 (m, 16 H, 8 x CH_2), 0.88 (t, *J* = 7.0 Hz, 3 H, CH_3); **^{13}C NMR** (100 MHz, $CDCl_3$): δ = 131.6, 128.3, 128.2, 122.7, 90.3, 84.7, 62.9, 37.9, 31.9, 29.61, 29.60, 29.54, 29.51, 29.31, 29.26, 25.2, 22.6,

14.1; **MS** (EI 70 ev *m/z*) (%): 286 (M^+, 2.65), 131 (100); **IR** (neat): ν = 3349, 2956, 2916, 2847, 1597, 1487, 1464, 1126, 1058, 1037 cm^{-1}; **HRMS** calcd for $C_{20}H_{30}O$ $[M]^+$: 286.2297, found: 286.2298.

(16) Preparation of 1-(4-bromophenyl)tetradec-1-yn-3-ol (1x)[1] (hcf-2-109)

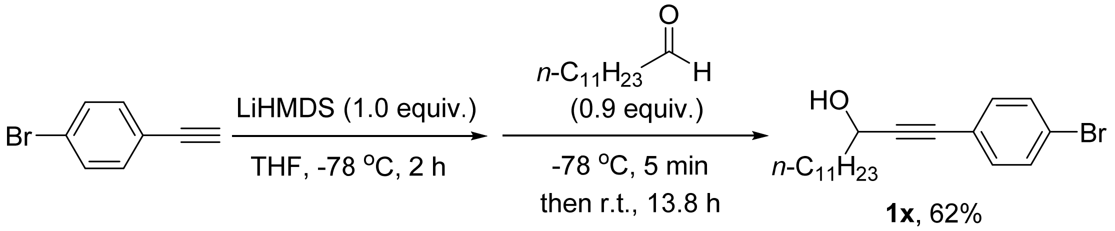

Following Typical Procedure I, the reaction of 4-bromophenylacetylene (3.7330 g, 20 mmol)/THF (20 mL), LiHMDS (1.0 M in tetrahydrofuran, 20 mL, 20 mmol), and dodecanal (4.1 mL, d = 0.831 g/mL, 3.4071 g, 18 mmol)/THF (10 mL) afforded **1x** (4.0665 g, 62%) [eluent: petroleum ether/ethyl ether/DCM = 50/1/1 (520 mL), 20/1/1 (550 mL), 10/1/1 (600 mL)]: white solid, m.p. 40.2-40.9 ℃ (petroleum ether/ethyl acetate); **^{1}H NMR** (400 MHz, $CDCl_3$): δ = 7.44 (d, *J* = 8.8 Hz, 2 H, Ar-H), 7.28 (d, *J* = 8.4 Hz, 2 H, Ar-H), 4.58 (q, *J* = 6.3 Hz, 1 H, CH), 1.87 (d, *J* = 5.2 Hz, 1 H, OH), 1.84-1.72 (m, 2 H, CH_2), 1.56-1.44 (m, 2 H, CH_2), 1.41-1.18 (m, 16 H, 8 x CH_2), 0.88 (t, *J* = 7.0 Hz, 3 H, CH_3); **^{13}C NMR** (100 MHz, $CDCl_3$): δ = 133.1, 131.5, 122.6, 121.6, 91.4, 83.7, 63.0, 37.8, 31.9, 29.62, 29.60, 29.54, 29.51, 29.3, 29.2, 25.2, 22.7, 14.1; **MS** (EI 70 ev *m/z*) (%): 366 (M^+ (^{81}Br), 5.85), 364 (M^+ (^{79}Br), 5.64), 211 (100); **IR** (neat): ν = 3364, 2951, 2914, 2847, 1670, 1484, 1465, 1257, 1057, 1012 cm^{-1}; Anal. Calcd. for $C_{20}H_{29}{}^{79}BrO$: C 65.75, H 8.00; found C 65.79, H 8.06.

2. Synthesis of optically active propargylic alcohols

(1) Preparation of (*S*)-3-methyl-1-phenyltetradec-1-yn-3-ol ((*S*)-1u)[2] (hcf-2-93)

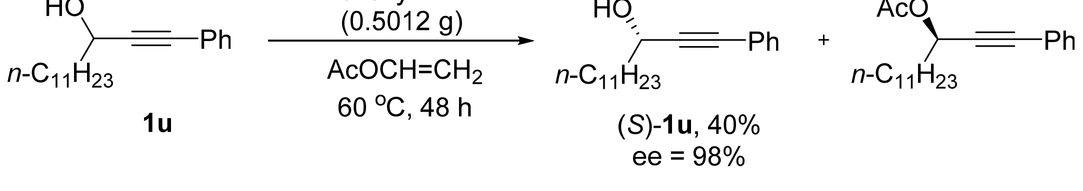

Typical Procedure III: To a flask (100 mL) were added **1u** (2.0295 g, 7 mmol) and vinyl acetate (25 mL). Then Novozym 435 (0.5012 g)/vinyl acetate (25 mL) was added. The resulting mixture was stirred at 60 °C for 48 h. After that, the resulting

mixture was filtered through a funnel and concentrated. The residue was purified by column chromatography on silica gel to afford (*S*)-**1u** (0.8118 g, 40%) as a yellow oil [eluent: petroleum ether/ethyl acetate = 50/1 (510 mL), 10/1 (550 mL)]: 98% ee (HPLC conditions: PA-2 column, hexane/*i*-PrOH = 95/5, 0.7 mL/min, λ = 214 nm, t_R (minor) = 8.5 min, t_R (major) = 9.2 min); $[\alpha]^{20}_D$ = +7.95 (*c* = 1.02, $CHCl_3$); **^{1}H NMR** (400 MHz, $CDCl_3$): δ = 7.48-7.38 (m, 2 H, Ar-H), 7.36-7.28 (m, 3 H, Ar-H), 4.60 (q, *J* = 6.3 Hz, 1 H, CH), 1.89-1.73 (m, 3 H, OH and CH_2), 1.60-1.45 (m, 2 H, CH_2), 1.42-1.18 (m, 16 H, 8 x CH_2), 0.88 (t, *J* = 7.0 Hz, 3 H, CH_3); **^{13}C NMR** (100 MHz, $CDCl_3$): δ = 131.7, 128.3, 128.2, 122.7, 90.2, 84.8, 63.0, 37.9, 31.9, 29.64, 29.61, 29.56, 29.5, 29.33, 29.28, 25.2, 22.7, 14.1; **MS** (EI 70 ev *m/z*) (%): 286 (M^+, 2.27), 131 (100); **IR** (neat): *v* = 3348, 2956, 2916, 2848, 1597, 1487, 1464, 1125, 1058, 1037 cm^{-1}; **HRMS** calcd for $C_{20}H_{30}O$ $[M]^+$: 286.2297, found: 286.2296.

(2) Preparation of (*S*)-1-(4-bromophenyl)tetradec-1-yn-3-ol ((*S*)-1x)[2] (hcf-2-111)

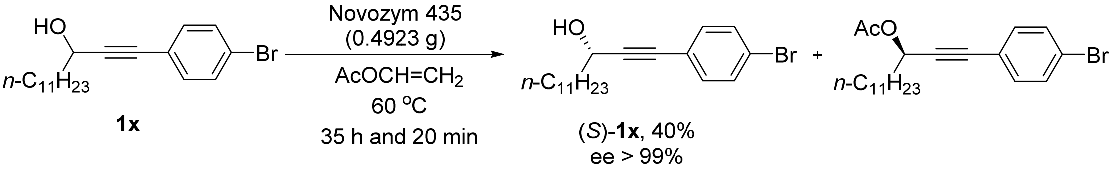

Following **Typical Procedure III**, the reaction of **1x** (2.1922 g, 6 mmol) and Novozym 435 (0.4923 g)/vinyl acetate (50 mL) afforded (*S*)-**1x** (0.8767 g, 40%) [eluent: petroleum ether/ethyl acetate = 50/1 (510 mL), 10/1 (550 mL)]: white solid, m.p. 40.0-40.8 ℃ (petroleum ether/ethyl acetate); >99% ee (HPLC conditions: PA-2 column, hexane/*i*-PrOH = 100/1, 0.7 mL/min, λ = 214 nm, t_R (major) = 38.8 min); $[\alpha]^{20}_D$ = +8.2 (*c* = 1.00, $CHCl_3$); **^{1}H NMR** (400 MHz, $CDCl_3$): δ = 7.43 (d, *J* = 8.8 Hz, 2 H, Ar-H), 7.28 (d, *J* = 8.4 Hz, 2 H, Ar-H), 4.57 (q, *J* = 6.1 Hz, 1 H, CH), 2.04-1.94 (m, 1 H, OH), 1.86-1.72 (m, 2 H, CH_2), 1.56-1.43 (m, 2 H, CH_2), 1.40-1.18 (m, 16 H, 8 x CH_2), 0.88 (t, *J* = 7.0 Hz, 3 H, CH_3); **^{13}C NMR** (100 MHz, $CDCl_3$): δ = 133.1, 131.5, 122.6, 121.6, 91.4, 83.7, 63.0, 37.8, 31.9, 29.63, 29.61, 29.54, 29.51, 29.3, 29.2, 25.2, 22.7, 14.1; **MS** (EI 70 ev *m/z*) (%): 366 (M^+(^{81}Br), 4.77), 364 (M^+(^{79}Br), 4.94), 211 (100); **IR** (neat): *v* = 3357, 2914, 2848, 1475, 1413, 1256, 1121, 1058 cm^{-1}; Anal. Calcd. for $C_{20}H_{29}{}^{79}BrO$: C 65.75, H 8.00; found C 65.69, H 8.04.

3. Preparation of 3-methyl-1-phenyloct-1-yn-3-yl methyl ether (3)[3] (hcf-2-148)

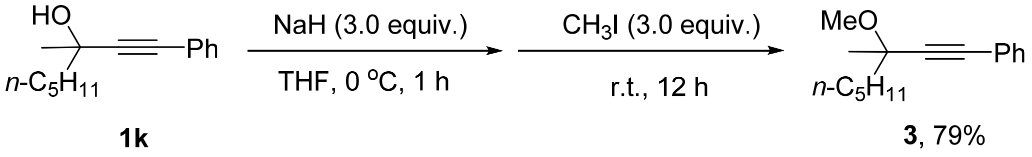

To a flame-dried Schlenk tube were added **1k** (1.7297 g, 8 mmol) and THF (10 mL). Then NaH (0.9613 g, 24 mmol) was added at 0 °C under argon. After stirring at 0 °C for 1 h, iodomethane (1.5 mL, d = 2.28 g/mL, 3.42 g, 24 mmol) was added. The resulting mixture was warmed up to room temperature, stirred for 12 h, and quenched with water (20 mL). After extraction with ethyl acetate (20 mL x 3), the organic layer was washed with brine (10 mL) and dried with anhydrous Na_2SO_4. After filtration and concentration under reduced pressure, the crude product was purified by column chromatography on silica gel to afford **3** (1.4516 g, 79%) as a colorless oil [eluent: petroleum ether 100 mL to petroleum ether/ethyl acetate = 100/1 (~810 mL)]: **^{1}H NMR** (400 MHz, $CDCl_3$): δ = 7.48-7.39 (m, 2 H, Ar-H), 7.36-7.26 (m, 3 H, Ar-H), 3.42 (s, 3 H, OMe), 1.83-1.66 (m, 2 H, CH_2), 1.65-1.42 (m, 5 H, CH_2 and CH_3), 1.41-1.23 (m, 4 H, 2 x CH_2), 0.91 (t, J = 6.8 Hz, 3 H, CH_3); **^{13}C NMR** (100 MHz, $CDCl_3$): δ = 131.7, 128.2, 128.1, 123.0, 90.5, 85.2, 74.0, 51.4, 41.3, 32.0, 25.6, 24.0, 22.6, 14.0; **MS** (EI 70 ev *m/z*) (%): 230 (M^+, 1.02), 159 (100); **IR** (neat): v = 2981, 2934, 2862, 2823, 1598, 1490, 1462, 1169, 1078 cm^{-1}; **HRMS** calcd for $C_{16}H_{22}O$ $[M]^+$: 230.1671, found: 230.1674.

4. Preparation of 3-methyl-1-phenyloct-1-yn-3-yl acetate (4)[4] (hcf-2-146)

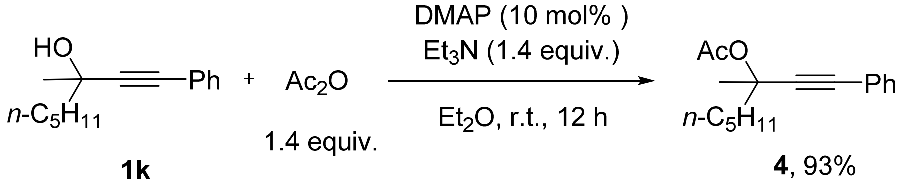

To a flask were added **1k** (1.0809 g, 5 mmol) and Et_2O (50 mL). Then acetic anhydride (0.66 mL, d = 1.08 g/mL, 0.7128 g, 7 mmol), trimethylamine (0.97 mL, d = 0.73 g/mL, 0.7081 g, 7 mmol), and DMAP (61.3 mg, 0.5 mmol) were added

sequentially. The resulting mixture was stirred at room temperature for 12 h and concentrated under reduced pressure. After that, the crude product was purified by column chromatography on silica gel to afford **4** (1.2071 g, 93%) as a colorless oil [eluent: petroleum ether 100 mL to petroleum ether/ ethyl acetate = 100/1 (~500 mL)]: **^{1}H NMR** (400 MHz, $CDCl_3$): δ = 7.48-7.39 (m, 2 H, Ar-H), 7.34-7.23 (m, 3 H, Ar-H), 2.10-1.96 (m, 4 H, one proton of CH_2 and CH_3), 1.94-1.81 (m, 1 H, one proton of CH_2), 1.74 (s, 3 H, CH_3), 1.64-1.44 (m, 2 H, CH_2), 1.42-1.28 (m, 4 H, 2 x CH_2), 0.91 (t, J = 7.0 Hz, 3 H, CH_3); **^{13}C NMR** (100 MHz, $CDCl_3$): δ = 169.3, 131.8, 128.2, 128.1, 122.7, 89.5, 84.9, 75.8, 41.5, 31.7, 26.5, 23.9, 22.5, 22.0, 14.0; **MS** (EI 70 ev *m/z*) (%): 258 (M^+, 1.02), 160 (100); **IR** (neat): v = 2954, 2932, 2864, 2240, 1742, 1490, 1367, 1232, 1149, 1122, 1012 cm^{-1}; **HRMS** calcd for $C_{17}H_{22}O_2$ $[M]^+$: 258.1620, found: 258.1622.

5. Synthesis of 3-hydroxyl-2(*E*)-alkenoates

(1) Preparation of methyl (*E*)-4-hydroxy-2-phenyl-4-(pyridin-4-yl)pent-2-enoate ((*E*)-2a) (hcf-1-197)

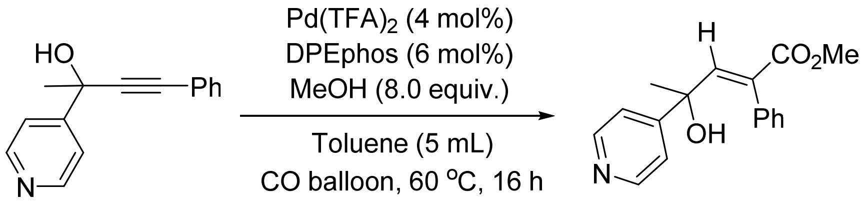

1a (*E*)-**2a**, 82%

Typical Procedure IV: To a flame-dried Schlenk tube were added $Pd(TFA)_2$ (13.6 mg, 0.04 mmol), DPEphos (32.3 mg, 0.06 mmol), **1a** (223.3 mg, 1 mmol), and MeOH (259.6 mg, 8.0 mmol)/toluene (5 mL) sequentially under argon. The resulting mixture was then frozen with a liquid nitrogen bath, degassed to remove the argon inside completely, and refilled with CO by a balloon of CO for three times. Then the resulting mixture was stirred at 60 ^oC with a balloon of CO for 16 h. After that, the resulting mixture was diluted with 3 mL of ethyl acetate, filtered through a short column of silica gel (3 cm) eluted with ethyl acetate (20 x 2 mL), and concentrated. The residue was purified by column chromatography on silica gel to afford impure

(E)-**2a** (241.3 mg) [eluent: dichloromethane/ethyl acetate = 5/1 (480 mL), 3/1 (200 mL), ethyl acetate (300 mL)], then recrystallized (petroleum ether/dichloromethane) to afford (E)-**2a** (232.1 mg, 82%): white solid, m.p. 112.9-113.5 ℃ (petroleum ether/dichloromethane); **^{1}H NMR** (400 MHz, $CDCl_3$): δ = 8.43 (d, J = 6.0 Hz, 2 H, Ar-H), 7.41 (s, 1 H, =CH), 7.33-7.17 (m, 5 H, Ar-H), 7.01-6.92 (m, 2 H, Ar-H), 3.71 (s, 3 H, CO_2Me), 3.17 (s, 1 H, OH), 1.59 (s, 3 H, CH_3); **^{13}C NMR** (100 MHz, $CDCl_3$): δ = 167.4, 155.9, 149.6, 147.4, 134.0, 133.4, 129.2, 128.3, 128.2, 120.0, 73.8, 52.6, 31.2; **MS** (ESI) m/z: 284 ($M+H^+$); **IR** (neat): ν = 3033, 2987, 2965, 2810, 2673, 1713, 1603, 1415, 1230, 1021 cm^{-1}; Anal. Calcd. for $C_{17}H_{17}NO_3$: C 72.07, H 6.05, N 4.94; found C 72.05, H 6.02, N 5.00.

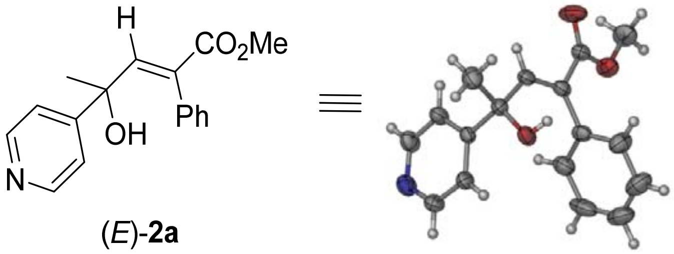

(E)-**2a**

Figure S1

(E)-**2a**: $C_{17}H_{17}NO_3$, MW = 283.32, monoclinic, space group $P2(1)/n$, final R indices [$I > 2\sigma(I)$], R_1 = 0.0539, wR_2 = 0.1472; R indices (all data), R_1 = 0.0765, wR_2 = 0.1585, a = 9.0342(9) Å, b = 14.3895(15) Å, c = 14.5074(15) Å, α = 90°, β = 101.707(3)°, γ = 90°, V = 1846.7(3) Å^3, T = 296(2) K, Z = 4, reflections collected/unique 3255/3255 [Rint = 0.0000], no. of observations [> $2\sigma(I)$] 2244, parameters: 190. Supplementary crystallographic data have been deposited at the Cambridge Crystallographic Data Centre via www.ccdc.cam.ac.uk/data_request/cif. CCDC-1571775

(2) Preparation of methyl (E)-4-hydroxy-2-phenyl-4-(pyridin-3-yl)pent-2-enoate ((E)-2b) (hcf-1-198)

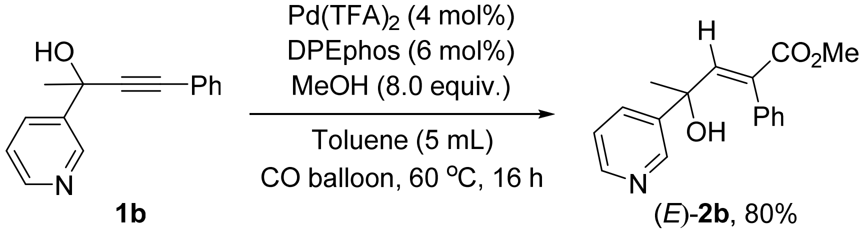

Following **Typical Procedure IV**, the reaction of $Pd(TFA)_2$ (13.6 mg, 0.04

mmol), DPEphos (32.3 mg, 0.06 mmol), **1b** (223.4 mg, 1 mmol), and MeOH (261.1 mg, 8 mmol)/toluene (5 mL) afforded (*E*)-**2b** (225.4 mg, 80%) [eluent: dichloromethane/ethyl acetate = 5/1 (480 mL), 3/1 (200 mL), ethyl acetate (300 mL)]: white solid, m.p. 108.5-109.1 ℃ (petroleum ether/ethyl acetate); **^{1}H NMR** (400 MHz, $CDCl_3$): δ = 8.53 (d, J = 2.0 Hz, 1 H, Ar-H), 8.41 (dd, J_1 = 4.8 Hz, J_2 = 1.6 Hz, 1 H, Ar-H), 7.61 (dt, J_1 = 8.0 Hz, J_2 = 1.9 Hz, 1 H, Ar-H), 7.44 (s, 1 H, =CH), 7.33-7.21 (m, 3 H, Ar-H), 7.21-7.15 (m, 1 H, Ar-H), 7.02-6.93 (m, 2 H, Ar-H), 3.71 (s, 3 H, CO_2Me), 2.79 (s, 1 H, OH), 1.64 (s, 3 H, CH_3); **^{13}C NMR** (100 MHz, $CDCl_3$): δ = 167.5, 148.1, 148.0, 146.9, 142.4, 134.1, 133.0, 132.7, 129.2, 128.14, 128.09, 123.1, 73.3, 52.5, 31.1; **MS** (ESI) *m/z*: 284 ($M+H^+$); **IR** (neat): ν = 3166, 2988, 2966, 2809, 1713, 1642, 1431, 1237, 1034, 1019 cm^{-1}; Anal. Calcd. for $C_{17}H_{17}NO_3$: C 72.07, H 6.05, N 4.94; found C 72.05, H 6.10, N 4.89.

(3) Preparation of methyl (*E*)-4-hydroxy-2-phenyl-4-(pyridin-2-yl)pent-2-enoate ((*E*)-2c) (hcf-2-2)

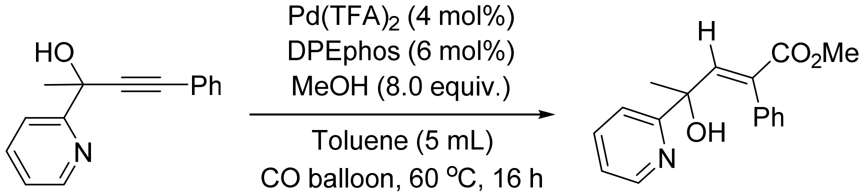

1c (*E*)-**2c**, 62%

Following **Typical Procedure IV**, the reaction of $Pd(TFA)_2$ (13.6 mg, 0.04 mmol), DPEPhos (32.3 mg, 0.06 mmol), **1c** (223.5 mg, 1 mmol), and MeOH (259.7 mg, 8 mmol)/toluene (5 mL) afforded (*E*)-**2c** (176.1 mg, 62%) as a yellow oil [eluent: dichloromethane 200 mL to dichloromethane/ethyl acetate = 30/1]: **^{1}H NMR** (400 MHz, $CDCl_3$): δ = 8.41 (d, J = 4.8 Hz, 1 H, Ar-H), 7.58 (td, J_1 = 7.8 Hz, J_2 = 1.6 Hz, 1 H, Ar-H), 7.39 (s, 1 H, =CH), 7.25-7.17 (m, 4 H, Ar-H), 7.13 (dd, J_1 = 7.0 Hz, J_2 = 5.0 Hz, 1 H, Ar-H), 7.02-6.90 (m, 2 H, Ar-H), 4.65 (br, 1 H, OH), 3.69 (s, 3 H, CO_2Me), 1.60 (s, 3 H, CH_3); **^{13}C NMR** (100 MHz, $CDCl_3$): δ = 168.0, 163.1, 148.5, 147.5, 136.8, 135.0, 133.1, 129.4, 127.5, 127.3, 122.1, 119.9, 73.9, 52.3, 29.7; **MS** (70 eV, EI) *m/z* (%): 284(M^++1, 6.07), 283 (M^+, 31.07), 162 (100); **IR** (neat): ν = 3400, 3056, 2980, 2950, 1713, 1589, 1433, 1241, 1195, 1072, 1019 cm^{-1}; **HRMS** calcd for

$C_{17}H_{17}NO_3$ $[M]^+$: 283.1208, found: 283.1212.

(4) Preparation of methyl (*E*)-4-hydroxy-2-phenyl-4-(quinolin-3-yl)pent-2-enoate ((*E*)-2d) (hcf-2-3)

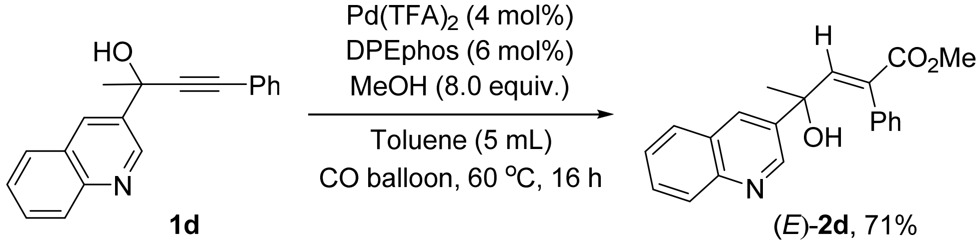

Following **Typical Procedure IV**, the reaction of $Pd(TFA)_2$ (13.6 mg, 0.04 mmol), DPEPhos (32.3 mg, 0.06 mmol), **1d** (273.3 mg, 1 mmol), and MeOH (260.1 mg, 8 mmol)/toluene (5 mL) afforded impure (*E*)-**2d** (243.4 mg) [eluent: dichloromethane/ethyl acetate = 40/1 (205 mL), 20/1 (420 mL), ethyl acetate (200 mL)], then recrystallized (petroleum ether/dichloromethane) to afford pure (*E*)-**2d** (236.7 mg, 71%): white solid, m.p. 137.7-138.5 ℃ (petroleum ether/dichloromethane); **^{1}H NMR** (400 MHz, $CDCl_3$): δ = 8.92 (d, J = 2.4 Hz, 1 H, Ar-H), 8.08 (d, J = 8.8 Hz, 1 H, Ar-H), 7.97 (d, J = 1.6 Hz, 1 H, =CH), 7.79-7.66 (m, 2 H, Ar-H), 7.59-7.49 (m, 2 H, Ar-H), 7.26-7.14 (m, 3 H, Ar-H), 7.02-6.93 (m, 2 H, Ar-H), 3.73 (s, 3 H, CO_2Me), 2.35-2.25 (m, 1 H, OH), 1.76 (s, 3 H, CH_3); **^{13}C NMR** (100 MHz, $CDCl_3$): δ = 167.5, 148.8, 147.9, 146.9, 139.4, 134.0, 133.3, 131.4, 129.4, 129.2, 128.9, 128.1, 127.9, 127.3, 126.8, 73.6, 52.5, 31.1; **MS** (70 eV, EI) *m/z* (%): 333 (M^+, 3.38), 230 (100); **IR** (neat): ν = 3174, 3055, 2992, 2948, 2922, 2851, 1709, 1433, 1254, 1198, 1113, 1036, 1021 cm^{-1}; Anal. Calcd. for $C_{21}H_{19}NO_3$: C 75.66, H 5.74, N 4.20; found C 75.31, H 5.78, N 4.19.

(5) Preparation of methyl (*E*)-4-hydroxy-4-(pyridin-4-yl)-2-(p-tolyl)pent-2-enoate ((*E*)-2e) (hcf-2-15)

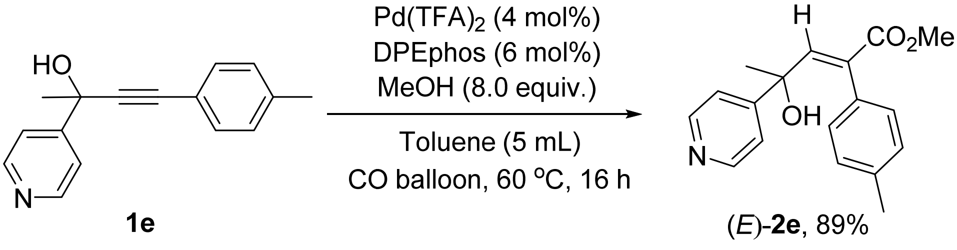

Following **Typical Procedure IV**, the reaction of $Pd(TFA)_2$ (13.6 mg, 0.04

mmol), DPEPhos (32.3 mg, 0.06 mmol), **1e** (237.2 mg, 1 mmol), and MeOH (258.1 mg, 8 mmol)/toluene (5 mL) afforded (*E*)-**2e** (264.6 mg, 89%) [eluent: dichloromethane/ethyl acetate = 5/1 (480 mL), 3/1 (200 mL), ethyl acetate (200 mL)]: white solid, m.p. 105.9-106.4 °C (petroleum ether/dichloromethane); **^{1}H NMR** (400 MHz, $CDCl_3$): δ = 8.49 (d, *J* = 6.0 Hz, 2 H, Ar-H), 7.40 (s, 1 H, =CH), 7.24 (d, *J* = 6.4 Hz, 2 H, Ar-H), 7.09 (d, *J* = 7.6 Hz, 2 H, Ar-H), 6.87 (d, *J* = 7.6 Hz, 2 H, Ar-H), 3.72 (s, 3 H, CO_2Me), 2.61 (s, 1 H, OH), 2.34 (s, 3 H, CH_3), 1.60 (s, 3 H, CH_3); **^{13}C NMR** (100 MHz, $CDCl_3$): δ = 167.6, 156.1, 149.4, 147.3, 138.1, 133.2, 130.8, 129.1, 128.9, 120.1, 73.7, 52.5, 31.1, 21.2; **MS** (70 eV, EI) *m/z* (%): 298 (M^++1, 2.12), 297 (M^+, 10.45), 194 (100); **IR** (neat): *ν* = 3121, 3030, 2974, 2956, 2860, 2814, 1708, 1601, 1413, 1260, 1222, 1172, 1018 cm^{-1}; Anal. Calcd. for $C_{18}H_{19}NO_3$: C 72.71, H 6.44, N 4.71; found C 72.89, H 6.33, N 4.61.

(6) Preparation of methyl (*E*)-2-(2-hydroxy-2-(pyridin-4-yl)propylidene)-hexanoate ((*E*)-2f) (hcf-2-4)

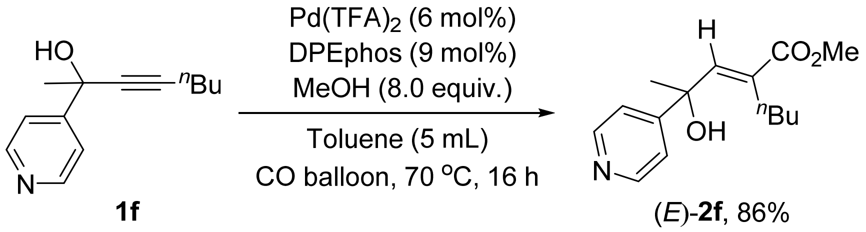

Following **Typical Procedure IV**, the reaction of $Pd(TFA)_2$ (20.4 mg, 0.06 mmol), DPEphos (48.5 mg, 0.09 mmol), **1f** (203.3 mg, 1 mmol), and MeOH (260.4 mg, 8 mmol)/toluene (5 mL) afforded impure (*E*)-2**f** (230.1 mg) [eluent: dichloromethane/ethyl acetate = 5/1 (480 mL), ethyl acetate 300 mL], then recrystallized (petroleum ether/dichloromethane) to afford pure (*E*)-**2f** (226.5 mg, 86%): white solid, m.p. 97.5-98.4 ℃ (petroleum ether/dichloromethane); **^{1}H NMR** (400 MHz, $CDCl_3$): δ = 8.55 (d, *J* = 5.6 Hz, 2 H, Ar-H), 7.44-7.35 (m, 2 H, Ar-H), 7.02 (s, 1 H, =CH), 3.75 (s, 3 H, CO_2Me), 2.81-2.48 (br, 1 H, OH), 2.32-2.20 (m, 2 H, CH_2), 1.72 (s, 3 H, CH_3), 1.28-0.98 (m, 4 H, 2 X CH_2), 0.77 (t, *J* = 7.2 Hz, 3 H, CH_3); **^{13}C NMR** (100 MHz, $CDCl_3$): δ = 168.6, 157.0, 149.1, 144.9, 136.2, 120.4, 72.8, 51.9, 32.3, 30.7, 27.2, 22.8, 13.6; **MS** (ESI) *m/z*: 264 ($M+H^+$); **IR** (neat): *ν* = 3163, 2962,

2931, 2861, 1708, 1599, 1432, 1409, 1267, 1173, 1080 cm^{-1}; Anal. Calcd. for $C_{15}H_{21}NO_3$: C 68.42, H 8.04, N 5.32; found C 68.35, H 7.96, N 5.20.

(7) Preparation of methyl (*E*)-2-(2-hydroxy-2-(pyridin-4-yl)propylidene)-decanoate ((*E*)-2g) (hcf-2-27)

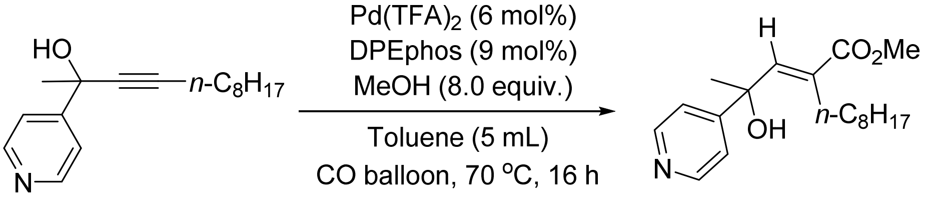

1g (*E*)-**2g**, 81%

Following **Typical Procedure IV**, the reaction of $Pd(TFA)_2$ (20.4 mg, 0.06 mmol), DPEphos (48.5 mg, 0.09 mmol), **1g** (259.6 mg, 1 mmol)/toluene (4.0 mL), and MeOH (259.7 mg, 8 mmol)/toluene (1.0 mL) afforded (*E*)-**2g** (258.9 mg, 81%) as a yellow oil [First run: eluent: dichloromethane/ethyl acetate = 5/1 (480 mL), 1/1 (200 mL) to ethyl acetate (100 mL) got the impure product. Second run: eluent: dichloromethane/ethyl acetate = 10/1 (440 mL), 5/1 (480 mL), 3/1 (200 mL), 1/1 (100 mL) got the pure (*E*)-**2g**]: **^{1}H NMR** (400 MHz, $CDCl_3$): δ = 8.54 (brs, 2 H, Ar-H), 7.40 (d, J = 4.8 Hz, 2 H, Ar-H), 7.01 (s, 1 H, =CH), 3.75 (s, 3 H, CO_2Me), 2.90-2.58 (m, 1 H, OH), 2.38-2.17 (m, 2 H, CH_2), 1.71 (s, 3 H, CH_3), 1.38-0.98 (m, 12 H, 6 X CH_2), 0.87 (t, J = 7.0 Hz, 3 H, CH_3); **^{13}C NMR** (100 MHz, $CDCl_3$): δ = 168.6, 157.2, 149.2, 144.6, 136.4, 120.5, 72.9, 51.9, 32.4, 31.7, 29.8, 29.14, 29.08, 28.7, 27.6, 22.6, 14.0; **MS** (70 eV, EI) *m/z* (%): 319 (M^+, 1.64), 276 (100); **IR** (neat): ν = 3148, 2920, 2852, 1719, 1601, 1434, 1246, 1224, 1195, 1086, 1065, 1003 cm^{-1}; **HRMS** calcd for $C_{19}H_{29}NO_3$ [M^+]: 319.2147, found: 319.2143.

(8) Preparation of methyl (*E*)-4-hydroxy-2-phenyl-4-(pyridin-4-yl)hex-2-enoate ((*E*)-2h) (hcf-1-199)

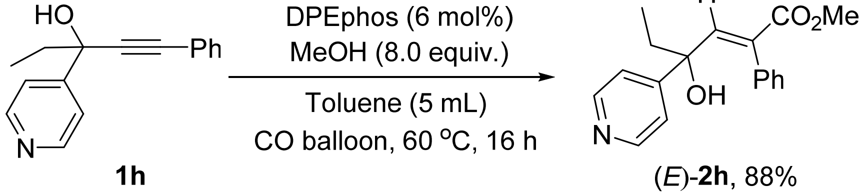

1h (*E*)-**2h**, 88%

Following **Typical Procedure IV**, the reaction of $Pd(TFA)_2$ (13.6 mg, 0.04

mmol), DPEphos (32.4 mg, 0.06 mmol), **1h** (237.1 mg, 1 mmol), and MeOH (260.1 mg, 8 mmol)/toluene (5 mL) afforded impure (*E*)-**2h** (265.1 mg) [eluent: dichloromethane/ethyl acetate = 5/1 (480 mL), ethyl acetate (300 mL)], then recrystallized (petroleum ether/dichloromethane) to afford pure (*E*)-**2h** (262.3 mg, 88%): white solid, m.p. 104.1-104.9 ℃ (petroleum ether/ethyl acetate); **^{1}H NMR** (400 MHz, $CDCl_3$): δ = 8.53 (d, J = 4.6 Hz, 2 H, Ar-H), 7.41 (s, 1 H, =CH), 7.37-7.25 (m, 3 H, Ar-H), 7.21 (dd, J_1 = 4.8 Hz, J_2 = 1.60 Hz, 2 H, Ar-H), 7.02-6.92 (m, 2 H, Ar-H), 3.72 (s, 3 H, CO_2Me), 2.03 (s, 1 H, OH), 2.01-1.80 (m, 2 H, CH_2), 0.83 (t, J = 7.4 Hz, 3 H, CH_3); **^{13}C NMR** (100 MHz, $CDCl_3$): δ = 167.4, 154.9, 149.6, 147.0, 134.0, 133.7, 129.2, 128.4, 128.3, 120.3, 76.4, 52.5, 36.6, 7.6; **MS** (ESI) *m/z*: 298 ($M+H^+$); **IR** (neat): ν = 3101, 3054, 2956, 2927, 2848, 1711, 1602, 1429, 1242, 1007 cm^{-1}; Anal. Calcd. for $C_{18}H_{19}NO_3$: C 72.71, H 6.44, N 4.71; found C 72.65, H 6.48, N 4.60.

(9) Preparation of methyl (*E*)-2-(2-hydroxy-2-phenyl-2-(pyridin-4-yl)ethylidene)-hexanoate ((*E*)-2i) (hcf-2-70)

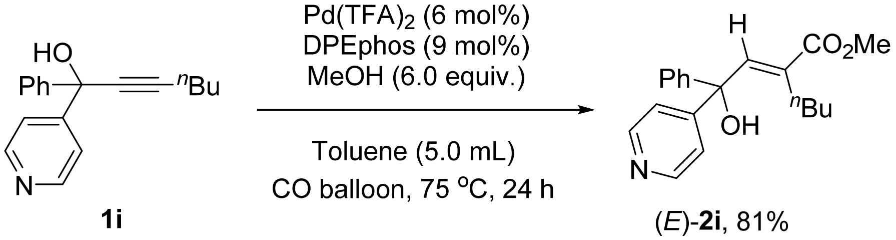

1i (*E*)-**2i**, 81%

Following **Typical Procedure IV**, the reaction of $Pd(TFA)_2$ (20.4 mg, 0.06 mmol), DPEPhos (48.5 mg, 0.09 mmol), **1i** (265.4 mg, 1 mmol), and MeOH (193.1 mg, 6 mmol)/toluene (5.0 mL) afforded (*E*)-**2i** (263.6 mg, 81%) as a white solid [eluent: dichloromethane (1000 mL) to dichloromethane/ethyl acetate = 5/1 (480 mL), 3/1 (200 mL)]: m.p. 92.5-93.0 °C (petroleum ether/dichloromethane); **^{1}H NMR** (400 MHz, $CDCl_3$): δ = 8.27 (d, J = 4.8 Hz, 2 H, Ar-H), 7.47-7.16 (m, 8 H, Ar-H and =CH), 5.33 (brs, 1 H, OH), 3.72 (s, 3 H, CO_2Me), 2.36 (t, J = 7.4 Hz, 2 H, CH_2), 1.24-1.02 (m, 4 H, 2 X CH_2), 0.73 (t, J = 6.8 Hz, 3 H, CH_3); **^{13}C NMR** (100 MHz, $CDCl_3$): δ = 168.6, 156.3, 148.9, 145.8, 143.8, 137.2, 128.4, 127.6, 126.2, 121.3, 77.4, 51.9, 30.7, 27.5, 22.8, 13.6; **MS** (EI 70 ev) *m/z* (%): 325 (M^+, 4.23), 187 (100); **IR** (neat): ν =

3086, 2948, 2860, 1715, 1597, 1436, 1262, 1247, 1208, 1129, 1021, 1003 cm^{-1}; **HRMS** calcd for $C_{20}H_{23}NO_3$ $[M]^+$: 325.1678, found: 325.1681.

(10) Preparation of methyl (*E*)-2-(2-hydroxy-2-(4-nitrophenyl)propylidene-octanoate ((*E*)-2j) (hcf-2-82)

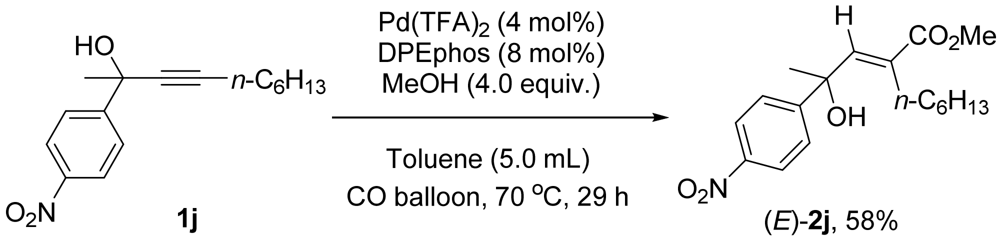

Following **Typical Procedure IV**, the reaction of $Pd(TFA)_2$ (13.6 mg, 0.04 mmol), DPEphos(43.1 mg, 0.08 mmol), **1j** (275.4 mg, 1 mmol)/toluene (4.0 mL), and MeOH (129.9 mg, 4 mmol)/toluene (1.0 mL) afforded (*E*)-**2j** (194.5 mg, 58%) as a white solid [eluent: petroleum ether/ethyl acetate = 20/1 (420 mL), 10/1 (1100 mL), 5/1 (240 mL)]: m.p. 90.1-90.5 °C (petroleum ether/ethyl acetate); **^{1}H NMR** (400 MHz, $CDCl_3$): δ = 8.20 (d, *J* = 8.8 Hz, 2 H, Ar-H), 7.66 (d, *J* = 9.2 Hz, 2 H, Ar-H), 7.06 (s, 1 H, =CH), 3.75 (s, 3 H, CO_2Me), 2.30-2.15 (m, 2 H, CH_2), 2.07 (s, 1 H, OH), 1.75 (s, 3 H, CH_3), 1.28-0.95 (m, 8 H, 4 x CH_2), 0.82 (t, *J* = 7.2 Hz, 3 H, CH_3); **^{13}C NMR** (100 MHz, $CDCl_3$): δ =168.5, 154.5, 146.8, 144.6, 136.8, 126.1, 123.5, 73.7, 52.1, 33.2, 31.4, 29.4, 28.6, 27.7, 22.4, 13.9; **MS** (EI 70 ev) *m/z* (%): 335 (M^+, 1.28), 292 (100); **IR** (neat): *v* = 3488, 3111, 3077, 2963, 2922, 2857, 1699, 1516, 1348, 1243, 1174, 1090 cm^{-1}; Anal. Calcd. for $C_{18}H_{25}NO_5$: C 64.46, H 7.51, N 4.18; found C 64.39, H 7.52, N 4.04.

(11) Preparation of methyl (*E*)-4-hydroxy-4-methyl-2-phenylnon-2-enoate ((*E*)-2k) (hcf-2-46)

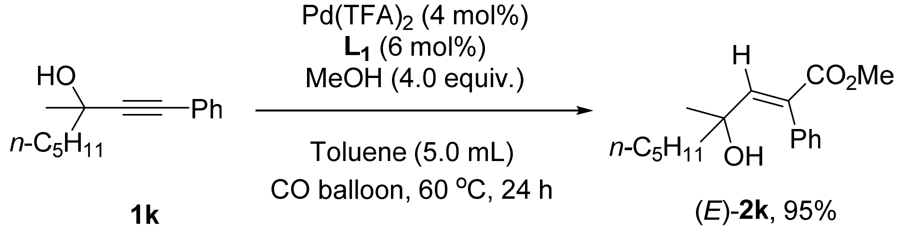

Following **Typical Procedure IV**, the reaction of $Pd(TFA)_2$ (13.6 mg, 0.04 mmol), $\mathbf{L_1}$ (32.8 mg, 0.06 mmol), **1k** (216.5 mg, 1 mmol)/toluene (4.0 mL), and MeOH (129.9 mg, 4 mmol)/toluene (1.0 mL) afforded (*E*)-**2k** (262.7 mg, 95%) as a yellow oil [eluent: petroleum ether/ethyl acetate = 20/1 (420 mL), 10/1 (440 mL)]: **^{1}H**

NMR (400 MHz, $CDCl_3$): δ = 7.41-7.30 (m, 3 H, Ar-H), 7.24-7.17 (m, 2 H, Ar-H), 7.08 (s, 1 H, =CH), 3.70 (s, 3 H, CO_2Me), 1.56-1.44 (m, 2 H, CH_2), 1.41-1.18 (m, 10 H, 3 x CH_2 and CH_3 and OH), 0.88 (t, J = 7.2 Hz, 3 H, CH_3); ^{13}**C NMR** (100 MHz, $CDCl_3$): δ = 167.9, 149.5, 135.1, 131.4, 129.4, 128.1, 127.9, 74.3, 52.3, 43.1, 32.1, 29.0, 23.6, 22.5, 14.0; **MS** (ESI) *m/z*: 299 $(M+Na)^+$, 259 $(M-OH)^+$; **IR** (neat): ν = 3486, 2953, 2931, 2860, 1716, 1637, 1435, 1238, 1197, 1020 cm^{-1}; **HRMS** calcd for $C_{17}H_{25}O_3$ $[M+H^+]$: 277.1798, found: 277.1795.

(12) Preparation of methyl (*E*)-2-(4-bromophenyl)-4-hydroxy-4-methylnon-2-enoate ((*E*)-2l) (hcf-2-60)

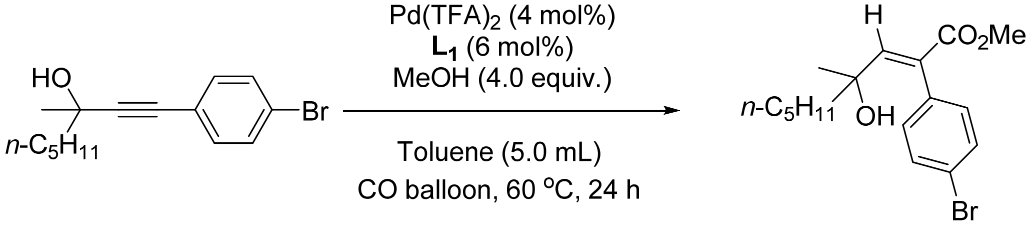

1l (*E*)-**2l**, 92%

Following **Typical Procedure IV**, the reaction of $Pd(TFA)_2$ (13.6 mg, 0.04 mmol), $\mathbf{L_1}$ (32.8 mg, 0.06 mmol), **1l** (295.1 mg, 1 mmol)/toluene (4.0 mL), and MeOH (131.1 mg, 4 mmol)/toluene (1.0 mL) afforded (*E*)-**2l** (326.7 mg, 92%) as a yellow oil [eluent: petroleum ether/ethyl acetate = 20/1 (420 mL), 10/1 (440 mL)]: ^{1}**H NMR** (400 MHz, $CDCl_3$): δ = 7.49 (d, J = 8.8 Hz, 2 H, Ar-H), 7.07 (d, J = 8.4 Hz, 2 H, Ar-H), 7.08 (s, 1 H, =CH), 3.71 (s, 3 H, CO_2Me), 1.58-1.45 (m, 2 H, CH_2), 1.44-1.10 (m, 10 H, 3 x CH_2 and CH_3 and OH), 0.88 (t, J = 7.2 Hz, 3 H, CH_3); ^{13}**C NMR** (100 MHz, $CDCl_3$): δ = 167.5, 150.1, 134.2, 131.20, 131.17, 130.5, 122.0, 74.3, 52.4, 43.1, 32.1, 28.9, 23.6, 22.5, 14.0; **MS** (ESI) *m/z*: 339 $(M(^{81}Br)-OH)^+$, 337 $(M(^{79}Br)-OH)^+$; **IR** (neat): ν = 3489, 2952, 2930, 2859, 1714, 1487, 1434, 1237, 1071, 1010 cm^{-1}; **HRMS** calcd for $C_{17}H_{24}{}^{79}BrO_3$ $[M+H^+]$: 355.0903, found: 355.0900.

Gram scale synthesis methyl (*E*)-2-(4-bromophenyl)-4-hydroxy-4-methylnon-2-enoate ((*E*)-2l) (hcf-2-97)

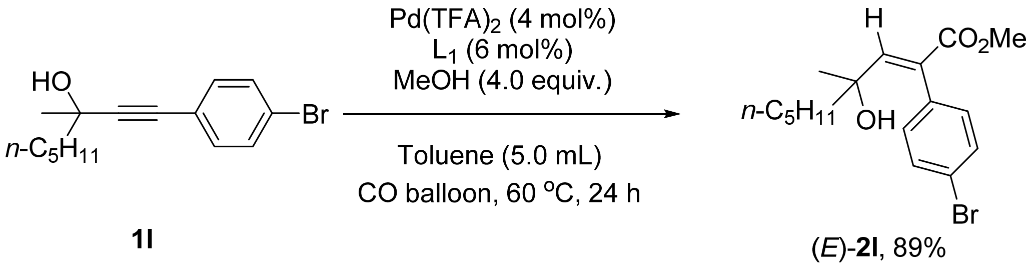

Following **Typical Procedure IV**, the reaction of Pd(TFA)$_2$ (54.4 mg, 0.16 mmol), **L$_1$** (131.2 mg, 0.24 mmol), **1l** (1.1811 g, 4 mmol)/toluene (16.0 mL), and MeOH (513.9 mg, 16 mmol)/toluene (4.0 mL) afforded (*E*)-**2l** (1.2650 g, 89%) as a yellow oil [eluent: petroleum ether/ethyl acetate = 50/1 (510 mL), 20/1 (525 mL), 5/1 (600 mL)]: **^{1}H NMR** (400 MHz, CDCl$_3$): δ = 7.49 (d, *J* = 8.4 Hz, 2 H, Ar-H), 7.076 (d, *J* = 8.4 Hz, 2 H, Ar-H), 7.078 (s, 1 H, =CH), 3.71 (s, 3 H, CO$_2$Me), 1.56-1.46 (m, 2 H, CH$_2$), 1.42-1.18 (m, 9 H, 3 x CH$_2$ and CH$_3$), 1.15 (s, 1 H, OH), 0.89 (t, *J* = 7.0 Hz, 3 H, CH$_3$); **^{13}C NMR** (100 MHz, CDCl$_3$): δ = 167.5, 150.1, 134.2, 131.2, 131.1, 130.4, 121.9, 74.3, 52.4, 43.1, 32.0, 28.9, 23.6, 22.5, 14.0.

(13) Preparation of methyl (*E*)-2-butyl-4-hydroxy-4-methylnon-2-enoate ((*E*)-2m) (hcf-2-92)

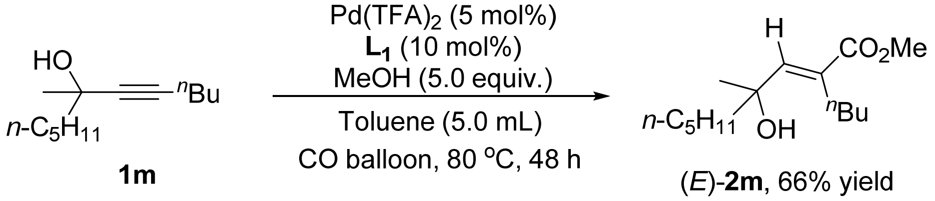

Following **Typical Procedure IV**, the reaction of Pd(TFA)$_2$ (17.0 mg, 0.05 mmol), **L$_1$** (54.7 mg, 0.10 mmol), **1m** (196.3 mg, 1 mmol)/toluene (4.0 mL), and MeOH (160.5 mg, 5 mmol)/toluene (1.0 mL) afforded (*E*)-**2m** (169.2 mg, 66%) (19% of **1m** remained based on ^{1}H NMR analysis of the crude product) as a yellow oil [eluent: petroleum ether/ethyl acetate = 50/1 (~410 mL), 20/1 (840 mL)]: **^{1}H NMR** (400 MHz, CDCl$_3$): δ = 6.66 (s, 1 H, =CH), 3.73 (s, 3 H, CO$_2$Me), 2.65-2.51 (m, 2 H, CH$_2$), 1.71-1.53 (m, 3 H, CH$_2$ and OH), 1.47-1.21 (m, 13 H, 5 x CH$_2$ and CH$_3$), 0.98-0.83 (m, 6 H, 2 x CH$_3$); **^{13}C NMR** (100 MHz, CDCl$_3$): δ = 169.1, 146.0, 133.3, 74.0, 51.8, 43.6, 32.3, 32.2, 29.0, 26.7, 23.6, 23.0, 22.5, 14.0, 13.9; **MS** (ESI) *m/z*: 239 (M-OH)$^+$; **IR** (neat): *v* = 3496, 2955, 2931, 2861, 1698, 1459, 1436, 1262, 1207,

1123 cm^{-1}; **HRMS** calcd for $C_{15}H_{27}O_2$ $[M-OH]^+$: 239.2006, found: 239.2005.

(14) Preparation of methyl (*E*)-2-octyl-4-hydroxy-4-methylhept-2-enoate ((*E*)-2n) (zwl-11-198)

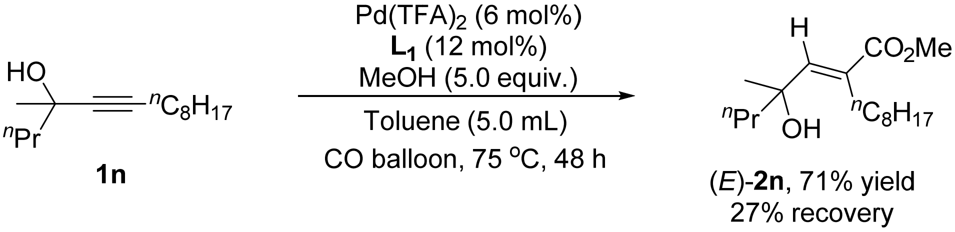

Following **Typical Procedure IV**, the reaction of $Pd(TFA)_2$ (20.6 mg, 0.06 mmol), $\mathbf{L_1}$ (65.7 mg, 0.12 mmol), **1n** (224.5 mg, 1 mmol)/toluene (4.0 mL), and MeOH (160.1 mg, 5 mmol)/toluene (1.0 mL) afforded (*E*)-**2n** (202.1 mg, 71%) (27% of **1n** remained based on ^{1}H NMR analysis of the crude product) as an oil [eluent: petroleum ether/ethyl ether/dichloromethane = 20/1/1]: ^{1}**H NMR** (400 MHz, $CDCl_3$): δ = 6.66 (s, 1 H, =CH), 3.73 (s, 3 H, CO_2Me), 2.63-2.50 (m, 2 H, CH_2), 1.68-1.52 (m, 3 H, CH_2 and OH), 1.50-1.17 (m, 17 H, 7 x CH_2 and CH_3), 0.93 (t, J = 7.4 Hz, 3 H, CH_3), 0.88 (t, J = 6.6 Hz, 3 H, CH_3); ^{13}**C NMR** (100 MHz, $CDCl_3$): δ = 169.1, 145.9, 133.4, 74.1, 51.8, 45.9, 31.8, 30.1, 30.0, 29.4, 29.2, 29.0, 27.0, 22.6, 17.3, 14.4, 14.1; **MS** (70 eV, EI) *m/z* (%): 285 (M^++1, 1.00), 284 (M^+, 1.94), 241 (100); **IR** (neat): ν = 3507, 2957, 2925, 2855, 1699, 1641, 1459, 1436, 1233, 1177, 1125 cm^{-1}; **HRMS** calcd for $C_{17}H_{32}O_3$ $[M]^+$: 284.2351, found: 284.2354.

(15) Preparation of methyl (*E*)-2-propyl-4-hydroxy-4-methylocta-2,7-dienoate ((*E*)-2o) (zwl-11-176)

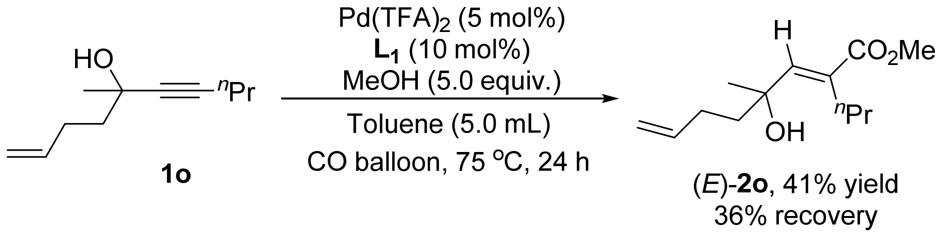

Following **Typical Procedure IV**, the reaction of $Pd(TFA)_2$ (17.2 mg, 0.05 mmol), $\mathbf{L_1}$ (54.8 mg, 0.10 mmol), **1o** (166.4 mg, 1 mmol)/toluene (4.0 mL), and MeOH (160.4 mg, 5 mmol)/toluene (1.0 mL) afforded (*E*)-**2o** (91.1 mg, 41%) (36% of **1o** remained based on ^{1}H NMR analysis of the crude product) as an oil [eluent:

petroleum ether/ethyl acetate = 20/1]: **^{1}H NMR** (400 MHz, $CDCl_3$): δ = 6.66 (s, 1 H, =CH), 5.92-5.78 (m, 1 H, =CH), 5.05 (d, *J* = 18.4 Hz, 1 H, one proton of =CH_2), 4.97 (d, *J* = 10.4 Hz, 1 H, one proton of =CH_2), 3.74 (s, 3 H, CO_2Me), 2.64-2.49 (m, 2 H, CH_2), 2.27-2.06 (m, 2 H, CH_2), 1.83-1.65 (m, 2 H, CH_2), 1.65-1.55 (m, 1 H, OH), 1.52-1.35 (m, 5 H, CH_2 and CH_3), 0.94 (t, *J* = 7.2 Hz, 3 H, CH_3); **^{13}C NMR** (100 MHz, $CDCl_3$): δ = 169.1, 145.7, 138.4, 133.4, 114.8, 74.0, 51.8, 42.4, 29.1, 28.9, 28.4, 23.2, 14.3; **MS** (ESI) *m/z*: 249 $(M+Na)^+$, 209 $(M-OH)^+$; **IR** (neat): *v* = 3498, 2958, 2932, 2872, 1698, 1641, 1436, 1268, 1219, 1171, 1121, 1060 cm^{-1}; **HRMS** calcd for $C_{13}H_{22}O_3Na$ $[M+Na]^+$: 249.1461, found: 249.1455.

(16) Preparation of methyl (*E*)-2-(4-chlorobutyl)-4-hydroxy-4-methylhept-2-enoate ((*E*)-2p) (zwl-11-196)

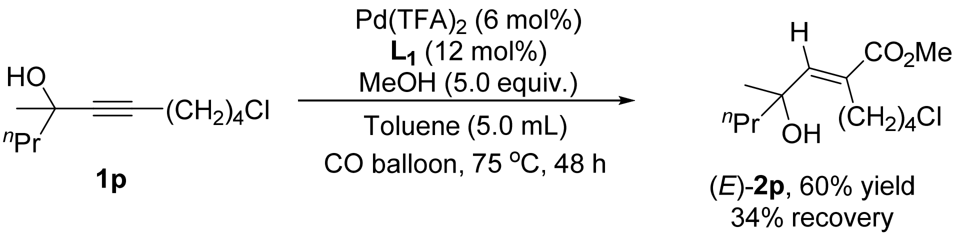

Following **Typical Procedure IV**, the reaction of $Pd(TFA)_2$ (20.5 mg, 0.06 mmol), **L_1** (65.7 mg, 0.12 mmol), **1p** (202.6 mg, 1 mmol)/toluene (4.0 mL), and MeOH (160.1 mg, 5 mmol)/toluene (1.0 mL) afforded (*E*)-**2p** (157.6 mg, 60%) (34% of **1p** remained based on ^{1}H NMR analysis of the crude product) as an oil [eluent: petroleum ether/ethyl ether/dichloromethane = 20/1/1]: **^{1}H NMR** (400 MHz, $CDCl_3$): δ = 6.67 (s, 1 H, =CH), 3.74 (s, 3 H, CO_2Me), 3.56 (t, *J* = 6.8 Hz, 2 H, CH_2), 2.70-2.55 (m, 2 H, CH_2), 1.88-1.78 (m, 2 H, CH_2), 1.70-1.20 (m, 10 H, OH and 3 x CH_2, and CH_3), 0.94 (t, *J* = 7.4 Hz, 3 H, CH_3); **^{13}C NMR** (100 MHz, $CDCl_3$): δ = 168.9, 146.5, 132.2, 74.0, 51.7, 45.9, 44.7, 32.5, 29.0, 27.1, 25.8, 17.1, 14.3; **MS** (ESI) *m/z*: 247 $(M(^{37}Cl)-OH)^+$, 245 $(M(^{35}Cl)-OH)^+$; **IR** (neat): *v* = 3495, 2957, 2872, 1697, 1642, 1436, 1274, 1237, 1190, 1094 cm^{-1}; **HRMS** calcd for $C_{13}H_{23}O_3{}^{35}Cl$ $[M]^+$: 262.1336, found: 262.1335.

(17) Preparation of methyl (*E*)-4-hydroxy-2,4-diphenylpent-2-enoate ((*E*)-2q) (hcf-2-55)

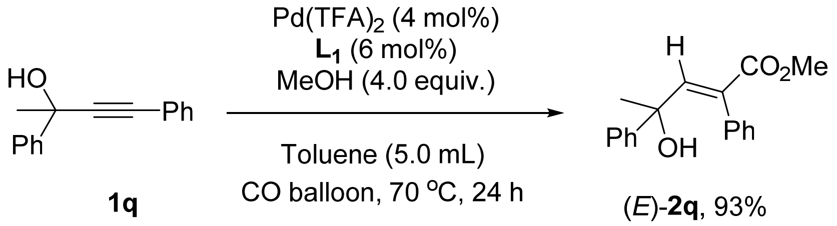

Following **Typical Procedure IV**, the reaction of $Pd(TFA)_2$ (13.6 mg, 0.04 mmol), $\mathbf{L_1}$ (32.8 mg, 0.06 mmol), **1q** (222.3 mg, 1 mmol), and MeOH (132.1 mg, 4 mmol)/toluene (5.0 mL) afforded (*E*)-**2q** (261.8 mg, 93%) as a white solid [eluent: petroleum ether (100 mL) to petroleum ether/ethyl acetate = 20/1 (~530 mL), 10/1 (440 mL)]: m.p. 64.5-65.0 °C (petroleum ether/dichloromethane); **^{1}H NMR** (400 MHz, $CDCl_3$): δ = 7.48 (s, 1 H, =CH), 7.39-7.18 (m, 8 H, Ar-H), 7.06-6.96 (m, 2 H, Ar-H), 3.70 (s, 3 H, CO_2Me), 1.99 (s, 1 H, OH), 1.60 (s, 3 H, CH_3); **^{13}C NMR** (100 MHz, $CDCl_3$): δ = 167.8, 149.1, 147.1, 134.6, 131.7, 129.4, 128.3, 128.1, 127.9, 127.1, 124.9, 74.8, 52.4, 31.2; **MS** (ESI) *m/z*: 300 $(M+NH_4)^+$, 265 $(M-OH)^+$; **IR** (neat): ν = 3505, 3057, 3010, 2986, 2954, 1693, 1639, 1435, 1251, 1211, 1149 cm^{-1}; Anal. Calcd. for $C_{18}H_{18}O_3$: C 76.57, H 6.43; found C 76.69, H 6.58.

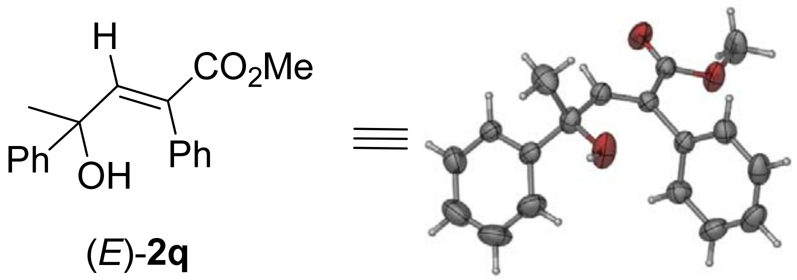

Figure S2

(*E*)-**2q**: $C_{18}H_{18}O_3$, MW = 282.32, orthorhombic, space group $P2_12_12_1$, final *R* indices $[I > 2\sigma(I)]$, $R_1 = 0.0278$, $wR_2 = 0.0691$; *R* indices (all data), $R_1 = 0.0288$, $wR_2 = 0.0696$, a = 7.95790(10) Å, b = 12.9023(2) Å, c = 14.8437(2) Å, $\alpha = 90^o$, $\beta = 90^o$, $\gamma = 90^o$, V = 1524.08(4) Å^3, T = 293(2) K, Z = 4, reflections collected/unique 15571/2710 [*R*int = 0.0285], no. of observations [> $2\sigma(I)$] 2617, parameters: 194. Supplementary crystallographic data have been deposited at the Cambridge Crystallographic Data Centre via www.ccdc.cam.ac.uk/data_request/cif. CCDC-1845002

(18) Preparation of methyl (*E*)-2-(2-hydroxy-2-phenylpropylidene)hexanoate ((*E*)-2r) (hcf-2-83)

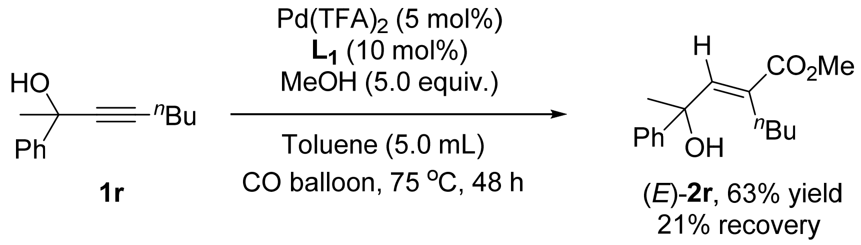

Following **Typical Procedure IV**, the reaction of Pd(TFA)$_2$ (17.0 mg, 0.05 mmol), **L$_1$** (54.7 mg, 0.10 mmol), **1r** (202.3 mg, 1 mmol)/toluene (4.0 mL), and MeOH (161.9 mg, 5 mmol)/toluene (1.0 mL) afforded (*E*)-**2r** (165.3 mg, 63%) (21% of **1r** remained based on ^{1}H NMR analysis of the crude product) as a yellow oil [eluent: petroleum ether/ethyl acetate = 50/1 (~410 mL), 20/1 (420 mL), 10/1 (220 mL)]: **^{1}H NMR** (400 MHz, CDCl$_3$): δ = 7.51-7.42 (m, 2 H, Ar-H), 7.39-7.29 (m, 2 H, Ar-H), 7.29-7.22 (m, 1 H, Ar-H), 7.07 (s, 1 H, =CH), 3.73 (s, 3 H, CO$_2$Me), 2.33-2.21 (m, 2 H, CH$_2$), 2.03 (s, 1 H, OH), 1.74 (s, 3 H, CH$_3$), 1.21-1.02 (m, 4 H, 2 x CH$_2$), 0.76 (t, J = 7.0 Hz, 3 H, CH$_3$); **^{13}C NMR** (100 MHz, CDCl$_3$): δ = 168.9, 147.0, 146.4, 134.6, 128.3, 127.1, 125.1, 74.3, 51.8, 31.8, 31.0, 27.2, 22.9, 13.7; **MS** (EI 70 ev) *m/z* (%): 262 (M$^+$, 1.80), 187 (100); **IR** (neat): v = 3483, 2955, 2930, 2870, 1697, 1437, 1263, 1209, 1140, 1066 cm^{-1}; **HRMS** calcd for $C_{16}H_{22}O_3$ [M]$^+$: 262.1569, found: 262.1566.

(19) Preparation of methyl (*E*)-3-(1-hydroxycyclobutyl)-2-phenylacrylate ((*E*)-2s) (hcf-2-87)

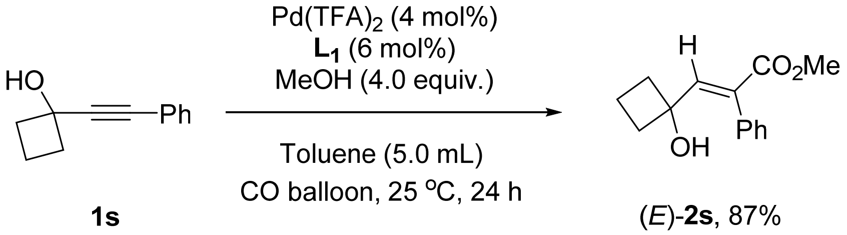

Following **Typical Procedure IV**, the reaction of Pd(TFA)$_2$ (13.6 mg, 0.04 mmol), **L$_1$** (32.8 mg, 0.06 mmol), **1s** (172.2 mg, 1 mmol), and MeOH (129.9 mg, 5 mmol)/toluene (5.0 mL) afforded (*E*)-**2s** (202.1 mg, 87%) as a yellow oil [eluent: petroleum ether/ethyl acetate = 50/1 (~200 mL), 20/1 (420 mL), 10/1 (440 mL)]: **^{1}H NMR** (400 MHz, CDCl$_3$): δ = 7.42-7.29 (m, 3 H, Ar-H), 7.32 (s, 1 H, =CH), 7.25-7.18 (m, 2 H, Ar-H), 3.74 (s, 3 H, CO$_2$Me), 2.28-2.16 (m, 2 H, CH$_2$), 2.14-2.01 (m, 2 H, CH$_2$), 1.94-1.78 (m, 2 H, one proton of CH$_2$ and OH), 1.76-1.62 (m, 1 H, one

proton of CH_2); **^{13}C NMR** (100 MHz, $CDCl_3$): δ = 167.9, 147.2, 134.9, 131.9, 129.5, 128.23, 128.16, 75.1, 52.4, 37.2, 13.0; **MS** (ESI) *m/z*: 250 ($M+NH_4^+$); **IR** (neat): ν = 2987, 2949, 1714, 1700, 1435, 1238, 1196, 1120 cm^{-1}; **HRMS** calcd for $C_{14}H_{20}NO_3$ [$M+NH_4^+$]: 250.1438, found: 250.1437.

(20) Preparation of methyl 4-hydroxy-4-phenyl-2-(*E*)-2-phenylpent-2-enoate ((*E*)-2t) (hcf-2-89)

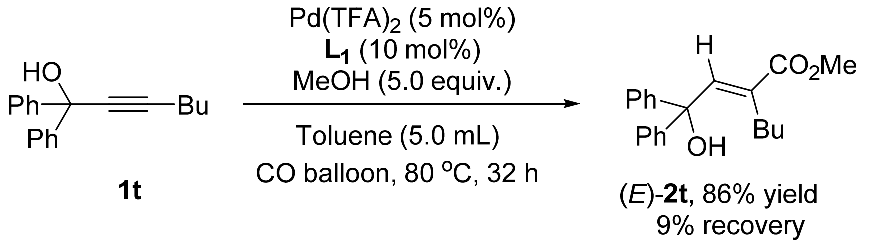

Following **Typical Procedure IV**, the reaction of $Pd(TFA)_2$ (17.0 mg, 0.05 mmol), **L_1** (54.7 mg, 0.10 mmol), **1t** (264.6 mg, 1 mmol)/toluene (4.0 mL), and MeOH (160.9 mg, 5 mmol)/toluene (1.0 mL) afforded (*E*)-**2t** (279.2 mg, 86%) (9% of **1t** remained based on ^{1}H NMR analysis of the crude product) as a yellow oil [eluent: petroleum ether/ethyl acetate = 20/1 (525 mL), 10/1 (220 mL)]: **^{1}H NMR** (400 MHz, $CDCl_3$): δ = 7.48-7.20 (m, 11 H, Ar-H and =CH), 3.74 (s, 3 H, CO_2Me), 2.46-2.31 (m, 3 H, CH_2 and OH), 1.30-1.08 (m, 4 H, 2 x CH_2), 0.78 (t, J = 7.0 Hz, 3 H, CH_3); **^{13}C NMR** (100 MHz, $CDCl_3$): δ = 168.9, 146.4, 144.8, 135.7, 128.3, 127.4, 126.5, 79.2, 51.9, 31.2, 27.5, 23.0, 13.8; **MS** (EI 70 ev) *m/z* (%): 324 (M^+, 2.66), 187 (100); **IR** (neat): ν = 3475, 2953, 2930, 2861, 1698, 1446, 1261, 1209, 1136 cm^{-1}; **HRMS** calcd for $C_{21}H_{24}O_3$ $[M]^+$: 324.1725, found: 324.1722.

(21) Preparation of methyl (*E*)-4-hydroxy-2-phenylpentadec-2-enoate ((*E*)-2u) (hcf-2-79)

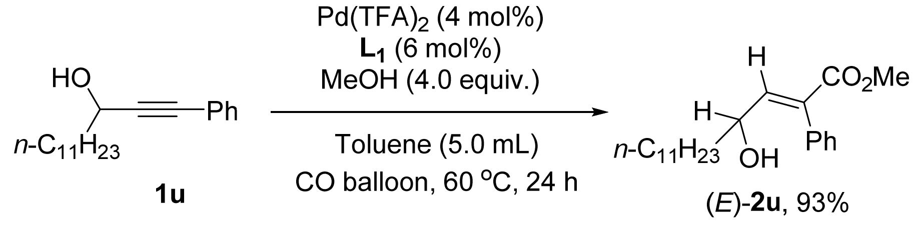

Following **Typical Procedure IV**, the reaction of $Pd(TFA)_2$ (13.6 mg, 0.04 mmol), **L_1** (32.8 mg, 0.06 mmol), **1u** (286.5 mg, 1 mmol)/toluene (4.0 mL), and MeOH (128.9 mg, 4 mmol)/toluene (1.0 mL) afforded (*E*)-**2u** (322.2 mg, 93%) as a yellow oil [eluent: petroleum ether/ethyl acetate = 20/1 (420 mL), 10/1 (440 mL), 5/1

(480 mL)]: **^{1}H NMR** (400 MHz, $CDCl_3$): δ = 7.41-7.31 (m, 3 H, Ar-H), 7.23-7.14 (m, 2 H, Ar-H), 6.92 (d, *J* = 9.6 Hz, 1 H, =CH), 4.18-4.09 (m, 1 H, CH), 3.75 (s, 3 H, CO_2Me), 1.71-1.44 (m, 3 H, CH_2 and OH), 1.36-1.11 (m, 18 H, 9 x CH_2), 0.88 (t, *J* = 7.0 Hz, 3 H, CH_3); **^{13}C NMR** (100 MHz, $CDCl_3$): δ = 167.5, 145.1, 134.6, 134.2, 129.4, 128.1, 127.8, 68.7, 52.3, 36.8, 31.9, 29.6, 29.5, 29.44, 29.36, 29.31, 25.0, 22.7, 14.1; **MS** (EI 70 ev) *m/z* (%): 346 (M^+, 1.71), 103 (100); **IR** (neat): *ν* = 3419, 2923, 2853, 1720, 1459, 1435, 1236, 1195, 1174, 1024 cm^{-1}; **HRMS** calcd for $C_{22}H_{34}O_3$ $[M]^+$: 346.2508, found: 346.2514.

(22) Preparation of methyl (*E*)-4-hydroxy-2,4-diphenylbut-2-enoate ((*E*)-2v) (hcf-2-125)

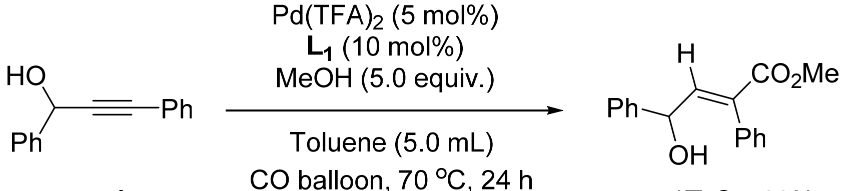

1v (*E*)-**2v**, 69%

Following **Typical procedure IV**, the reaction of $Pd(TFA)_2$ (17.0 mg, 0.05 mmol), $\mathbf{L_1}$ (54.7 mg, 0.10 mmol), **1v** (208.3 mg, 1 mmol)/toluene (4.0 mL), and MeOH (160.5 mg, 5 mmol)/toluene (1.0 mL) afforded (*E*)-**2v** (185.2 mg, 69%) as a yellow oil [eluent: petroleum ether/ethyl acetate = 10/1 (1100 mL), 5/1 (240 mL)]: **^{1}H NMR** (400 MHz, $CDCl_3$): δ = 7.50-7.24 (m, 8 H, Ar-H), 7.24-7.11 (m, 3 H, Ar-H and =CH), 5.19 (d, *J* = 9.2 Hz, 1 H, CH), 3.73 (s, 3 H, CO_2Me), 2.13 (brs, 1 H, OH); **^{13}C NMR** (100 MHz, $CDCl_3$): δ = 167.4, 143.5, 141.7, 134.3, 134.0, 129.5, 128.7, 128.1, 128.0, 126.3, 70.9, 52.3; **MS** (EI 70 ev) *m/z* (%): 269 (M^++1, 1.31), 268 (M^+, 7.23), 105 (100); **IR** (neat): *ν* = 3418, 1696, 1641, 1490, 1442, 1228 cm^{-1}; **HRMS** calcd for $C_{17}H_{16}O_3$ $[M]^+$: 268.1099, found: 268.1096.

(23) Preparation of methyl (*E*)-4-hydroxy-2-(4-methoxyphenyl)-4-phenylbut-2-enoate ((*E*)-2w) (hcf-2-126)

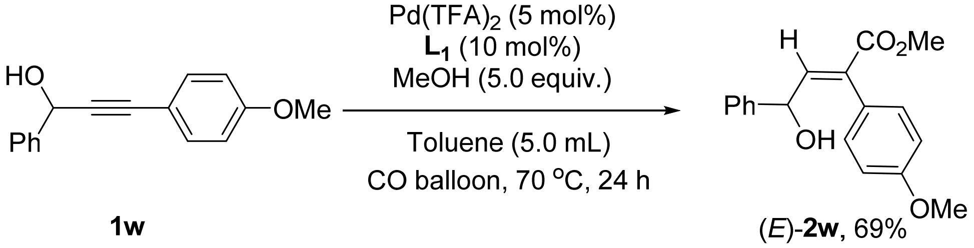

1w (*E*)-**2w**, 69%

Following **Typical procedure IV**, the reaction of $Pd(TFA)_2$ (17.0 mg, 0.05 mmol), $\mathbf{L_1}$ (54.7 mg, 0.10 mmol), **1w** (238.3 mg, 1 mmol)/toluene (4.0 mL), and MeOH (168.9 mg, 5 mmol)/toluene (1.0 mL) afforded (*E*)-**2w** (205.8 mg, 69%) as a yellow oil [eluent: petroleum ether/ethyl acetate = 10/1 (880 mL), 5/1 (600 mL)]: ^{1}**H NMR** (400 MHz, $CDCl_3$): δ = 7.40-7.19 (m, 5 H, Ar-H), 7.19-7.03 (m, 3 H, Ar-H and =CH), 6.90 (d, *J* = 8.8 Hz, 2 H, Ar-H), 5.19 (d, *J* = 9.2 Hz, 1 H, CH), 3.81 (s, 3 H, CO_2Me), 3.71 (s, 3 H, OMe), 2.55 (brs, 1 H, OH); ^{13}**C NMR** (100 MHz, $CDCl_3$): δ = 167.6, 159.3, 143.1, 141.9, 133.7, 130.8, 128.7, 128.1, 126.5, 126.3, 113.6, 71.0, 55.2, 52.3; **MS** (EI 70 ev) *m/z* (%): 299 (M^++1, 1.96), 298 (M^+, 9.60), 105 (100); **IR** (neat): *ν* = 3420, 3028, 2948, 2837, 1707, 1508, 1444, 1234, 1173, 1017 cm^{-1}; **HRMS** calcd for $C_{18}H_{18}O_4$ $[M]^+$: 298.1205, found: 298.1208.

(24) Preparation of methyl (*E*)-2-(4-bromophenyl)-4-hydroxypentadec-2-enoate ((*E*)-2x) (hcf-2-114)

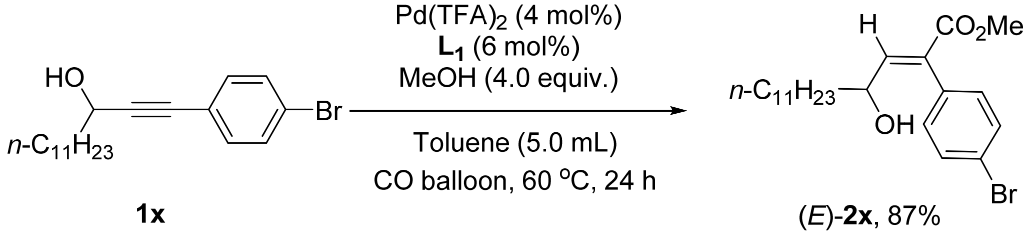

1x (*E*)-**2x**, 87%

Following **Typical Procedure IV**, the reaction of $Pd(TFA)_2$ (13.6 mg, 0.04 mmol), $\mathbf{L_1}$ (32.8 mg, 0.06 mmol), **1x** (365.4 mg, 1 mmol), and MeOH (128.7 mg, 4 mmol)/toluene (5.0 mL) afforded (*E*)-**2x** (370.1 mg, 87%) as a yellow oil [eluent: petroleum ether/ethyl acetate = 20/1 (420 mL), 10/1 (550 mL), 5/1 (240 mL)]: ^{1}**H NMR** (400 MHz, $CDCl_3$): δ = 7.50 (d, *J* = 8.0 Hz, 2 H, Ar-H), 7.07 (d, *J* = 8.4 Hz, 2 H, Ar-H), 6.93 (d, *J* = 9.6 Hz, 1 H, =CH), 4.10 (q, *J* = 7.5 Hz, 1 H, CH), 3.75 (s, 3 H, CO_2Me), 1.71-1.43 (m, 3 H, CH_2 and OH), 1.36-1.12 (m, 18 H, 9 x CH_2), 0.88 (t, *J* = 7.0 Hz, 3 H, CH_3); ^{13}**C NMR** (100 MHz, $CDCl_3$): δ = 167.0, 145.6, 133.4, 133.1, 131.3, 131.2, 122.1, 68.6, 52.4, 36.8, 31.9, 29.6, 29.5, 29.4, 29.35, 29.31, 25.0, 22.7, 14.1; **MS** (EI 70 ev) *m/z* (%): 426 ($M^+(^{81}Br)$, 1.43), 424 ($M^+(^{79}Br)$, 1.23), 43 (100); **IR** (neat): *ν* = 3427, 2922, 2852, 1720, 1487, 1435, 1236, 1071, 1032, 1010 cm^{-1}; **HRMS** calcd for $C_{22}H_{33}{}^{79}BrO_3$ $[M]^+$: 424.1613, found: 424.1617.

(25) Preparation of ethyl (*E*)-4-hydroxy-4-methyl-2-phenylnon-2-enoate ((*E*)-2ka) (hcf-2-76)

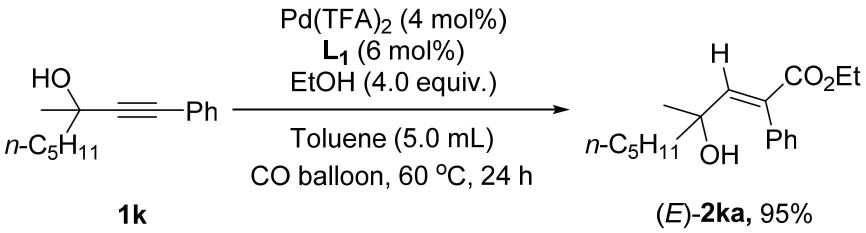

Following **Typical Procedure IV**, the reaction of $Pd(TFA)_2$ (13.6 mg, 0.04 mmol), $\mathbf{L_1}$ (32.8 mg, 0.06 mmol), **1k** (216.3 mg, 1 mmol)/toluene (4 mL), and EtOH (185.9 mg, 4 mmol)/toluene (1 mL) afforded (*E*)-**2ka** (275.8 mg, 95%) as yellow oil [eluent: petroleum ether/ethyl acetate = 20/1 (420 mL), 10/1 (440 mL)]: **^{1}H NMR** (400 MHz, $CDCl_3$): δ = 7.42-7.30 (m, 3 H, Ar-H), 7.24-7.17 (m, 2 H, Ar-H), 7.05 (s, 1 H, =CH), 4.17 (q, *J* = 7.1 Hz, 2 H, CO_2CH_2), 1.57-1.46 (m, 2 H, CH_2), 1.41-1.16 (m, 13 H, 3 x CH_2 and 2 x CH_3 and OH), 0.88 (t, *J* = 7.2 Hz, 3 H, CH_3); **^{13}C NMR** (100 MHz, $CDCl_3$): δ = 167.5, 149.0, 135.2, 131.7, 129.5, 128.1, 127.8, 74.3, 61.2, 43.2, 32.1, 29.0, 23.6, 22.6, 14.1, 14.0; **MS** (ESI) *m/z*: 313 $(M+Na)^+$, 308 $(M+NH_4)^+$, 291 $(M+H)^+$, 273 $(M-OH)^+$; **IR** (neat): ν = 3483, 2955, 2931, 2860, 1713, 1463, 1444, 1233, 1189, 1037, 1027 cm^{-1}; **HRMS** calcd for $C_{18}H_{27}O_3$ $[M+H]^+$: 291.1955, found: 291.1955.

(26) Preparation of (trimethylsilyl)methyl 4-hydroxy-4-methyl-2(*E*)-phenyl-non-2-enoate ((*E*)-2kb) (hcf-3-19)

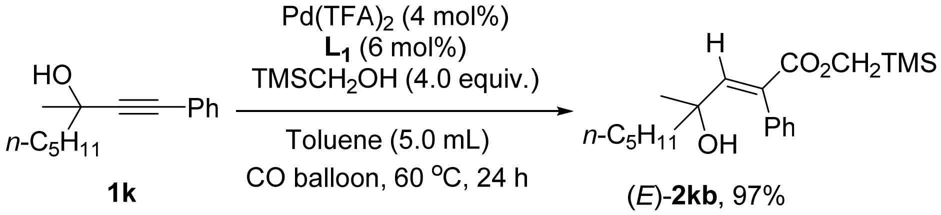

Following **Typical procedure IV**. The reaction of $Pd(TFA)_2$ (13.6 mg, 0.04 mmol), $\mathbf{L_1}$ (32.8 mg, 0.06 mmol), **1k** (216.1 mg, 1 mmol)/toluene (4 mL), and $TMSCH_2OH$ (417.1 mg, 4 mmol)/toluene (1 mL) afforded (*E*)-**2kb** (336.7 mg, 97%) as a yellow oil [eluent: petroleum ether/ethyl acetate = 25/1 (520 mL), 10/1 (440 mL)]: **^{1}H NMR** (400 MHz, $CDCl_3$): δ = 7.39-7.28 (m, 3 H, Ar-H), 7.22-7.15 (m, 2 H, Ar-H), 7.05 (s, 1 H, =CH), 3.76 (s, 2 H, OCH_2), 1.57-1.47 (m, 2 H, CH_2), 1.44-1.18 (m, 10 H, 3 x CH_2 and CH_3 and OH), 0.88 (t, *J* = 7.0 Hz, 3 H, CH_3), -0.08 (s, 9 H, 3 x CH_3); **^{13}C**

NMR (100 MHz, $CDCl_3$): δ = 168.3, 148.8, 135.5, 131.9, 129.4, 128.0, 127.7, 74.3, 58.3, 43.2, 32.1, 29.0, 23.6, 22.5, 14.0, -3.3; **MS** (EI 70 ev) *m/z* (%): 348 (M^+, 1.58), 173 (100); **IR** (neat): *v* = 3487, 2956, 2931, 2861, 1713, 1287, 1249, 1217, 1148 cm^{-1}; **HRMS** calcd for $C_{20}H_{32}O_3Si$ $[M]^+$: 348.2121, found: 348.2110.

(27) Preparation of isopropyl 4-hydroxy-4-methyl-2(*E*)-phenylnon-2-enoate ((*E*)-2kc) (hcf-3-20, 28)

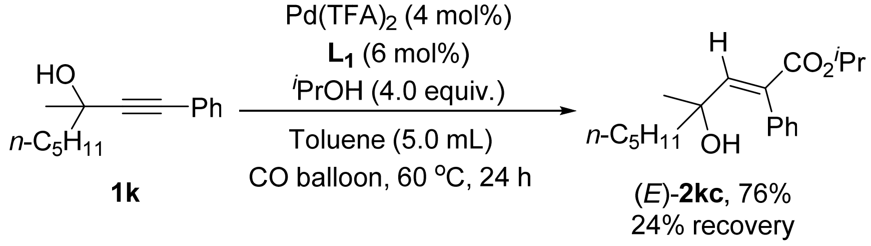

Following **Typical procedure IV**. The reaction of $Pd(TFA)_2$ (13.6 mg, 0.04 mmol), $\mathbf{L_1}$ (32.8 mg, 0.06 mmol), **1k** (216.5 mg, 1 mmol)/toluene (4 mL), and ^{i}PrOH (240.5 mg, 4 mmol)/toluene (1 mL) afforded (*E*)-**2kc** (231.1 mg, 76%) (24% of **1k** remained based on ^{1}H NMR analysis of the crude product) as a yellow oil [eluent: petroleum ether/ethyl acetate = 20/1 (840 mL), 10/1 (220 mL)]: **^{1}H NMR** (400 MHz, $CDCl_3$): δ = 7.40-7.27 (m, 3 H, Ar-H), 7.23-7.12 (m, 2 H, Ar-H), 7.01 (s, 1 H, =CH), 5.03 (hept, *J* = 6.2 Hz, 1 H, CH), 1.58-1.44 (m, 2 H, CH_2), 1.44-1.08 (m, 16 H, 3 x CH_2 and 3 x CH_3 and OH), 0.88 (t, *J* = 7.2 Hz, 3 H, CH_3); **^{13}C NMR** (100 MHz, $CDCl_3$): δ = 166.9, 148.6, 135.3, 132.1, 129.5, 128.0, 127.7, 74.2, 68.5, 43.1, 32.1, 29.0, 23.5, 22.5, 21.7, 14.0; **MS** (ESI) *m/z*: 327 $(M+Na)^+$, 287 $(M-OH)^+$; **IR** (neat): *v* = 3497, 2956, 2932, 2870, 1709, 1465, 1373, 1238, 1182, 1106, 1004 cm^{-1}; HRMS calcd for $C_{19}H_{28}O_3$ $[M]^+$: 304.2038, found: 304.2038.

(28) Preparation of phenyl (*E*)-4-hydroxy-4-methyl-2-phenylnon-2-enoate ((*E*)-2kd) (hcf-2-77)

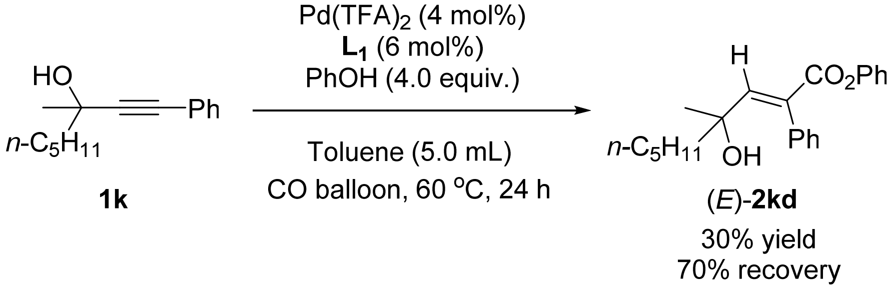

Following **Typical Procedure IV**, the reaction of Pd(TFA)$_2$ (13.6 mg, 0.04 mmol), **L$_1$** (32.8 mg, 0.06 mmol), **1k** (216.5 mg, 1 mmol)/toluene (5 mL), and PhOH (376.6 mg, 4 mmol) afforded (*E*)-**2kd** (101.6 mg, 30%) (70% of **1k** remained based on ^{1}H NMR analysis of the crude product) as a yellow oil [eluent: petroleum ether/ethyl acetate = 50/1(~200 mL), 20/1 (630 mL), 10/1 (220 mL)]: **^{1}H NMR** (400 MHz, CDCl$_3$): δ = 7.54-7.29 (m, 7 H, Ar-H), 7.27 (s, 1 H, =CH), 7.19 (t, *J* = 7.2 Hz, 1 H, Ar-H), 7.07 (d, *J* = 7.6 Hz, 2 H, Ar-H), 1.64-1.49 (m, 2 H, CH$_2$), 1.48-1.19 (m, 10 H, 3 x CH$_2$ and CH$_3$ and OH), 0.90 (t, *J* = 7.0 Hz, 3 H, CH$_3$); **^{13}C NMR** (100 MHz, CDCl$_3$): δ = 166.0, 151.2, 150.9, 134.7, 131.1, 129.5, 129.2, 128.2, 128.1, 125.7, 121.4, 74.4, 43.1, 32.1, 28.9, 23.6, 22.5, 14.0; **MS** (ESI) m/z: 361 $(M+Na)^+$, 339 $(M+H)^+$, 321 $(M-OH)^+$; **IR** (neat): *v* = 3471, 2930, 2858, 1724, 1485, 1237, 1164, 1054 cm^{-1}; **HRMS** calcd for $C_{22}H_{27}O_3$ $[M+H]^+$: 339.1955, found: 339.1977.

(29) Preparation of methyl (*S*, *E*)-4-hydroxy-2-phenylpentadec-2-enoate ((*S*, *E*)-2u) (hcf-2-96)

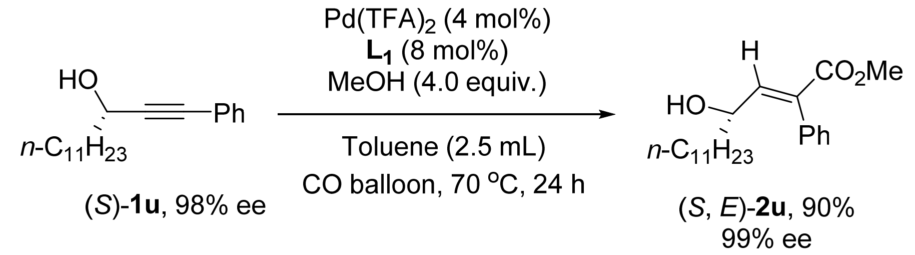

Following **Typical Procedure IV**, the reaction of Pd(TFA)$_2$ (6.8 mg, 0.02 mmol), **L$_1$** (21.9 mg, 0.04 mmol), (*S*)-**1u** (143.2 mg, 98% ee, 0.5 mmol)/toluene (2.0 mL), and MeOH (64.9 mg, 2 mmol)/toluene (0.5 mL) afforded (*S*, *E*)-**2u** (155.8 mg, 90%) as a yellow oil [eluent: petroleum ether/ethyl acetate = 20/1 (525 mL), 10/1 (550 mL), 5/1 (240 mL)]: 99% ee (HPLC conditions: PA-2 column, hexane/*i*-PrOH = 95/5, 1.0 mL/min, λ = 214 nm, t_R (major) = 16.4 min, t_R (minor) = 15.1 min); $[\alpha]^{20}_D$ = -20.9 (*c* = 1.02, CHCl$_3$); **^{1}H NMR** (400 MHz, CDCl$_3$): δ = 7.44-7.30 (m, 3 H, Ar-H), 7.24-7.13 (m, 2 H, Ar-H), 6.92 (d, *J* = 9.6 Hz, 1 H, =CH), 4.13 (q, *J* = 7.5 Hz, 1 H, CH), 3.75 (s, 3 H, CO$_2$Me), 1.76-1.44 (m, 3 H, CH$_2$ and OH), 1.41-1.11 (m, 18 H, 9 x CH$_2$), 0.88 (t, *J* = 6.8 Hz, 3 H, CH$_3$); **^{13}C NMR** (100 MHz, CDCl$_3$): δ = 167.5, 145.2, 134.5, 134.1, 129.4, 128.1, 127.8, 68.6, 52.3, 36.7, 31.9, 29.6, 29.5, 29.4, 29.34, 29.30, 25.0, 22.7,

14.1; **MS** (EI 70 ev) *m/z* (%): 346 (M^+, 1.23), 103 (100); **IR** (neat): *v* = 3434, 2922, 2853, 1720, 1462, 1435, 1236, 1195, 1175, 1024 cm^{-1}; **HRMS** calcd for $C_{22}H_{34}O_3$ $[M]^+$: 346.2508, found: 346.2510.

(30) Preparation of methyl (*S*, *E*)-2-(4-bromophenyl)-4-hydroxypentadec-2-enoate ((*S, E*)-2x) (hcf-2-118)

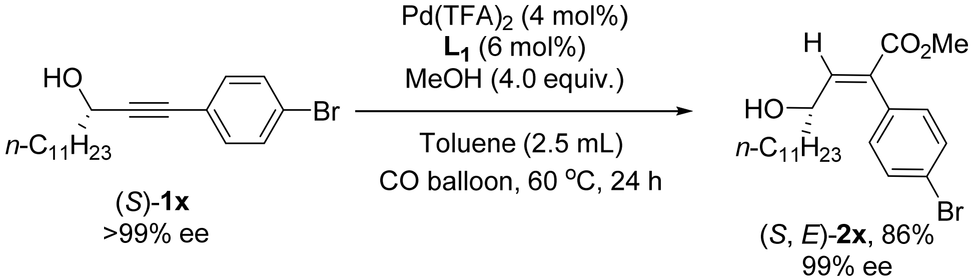

(*S*)-**1x**
>99% ee

(*S*, *E*)-**2x**, 86%
99% ee

Following **Typical Procedure IV**, the reaction of $Pd(TFA)_2$ (6.8 mg, 0.02 mmol), $\mathbf{L_1}$ (16.4 mg, 0.03 mmol), (*S*)-**1x** (182.7 mg, >99% ee, 0.5 mmol), and MeOH (64.9 mg, 2 mmol)/toluene (2.5 mL) afforded (*S*, *E*)-**2x** (182.9 mg, 86%) as a yellow oil [eluent: petroleum ether/ethyl acetate = 20/1 (525 mL), 10/1 (550 mL)]: 99% ee (HPLC conditions: AS-H column, hexane/*i*-PrOH = 97/3, 1.0 mL/min, λ = 214 nm, t_R (major) = 8.6 min, t_R (minor) = 7.4 min); $[\alpha]^{20}_D$ = -24.3 (*c* = 1.01, $CHCl_3$); **^{1}H NMR** (400 MHz, $CDCl_3$): δ = 7.49 (d, *J* = 8.4 Hz, 2 H, Ar-H), 7.06 (d, *J* = 8.4 Hz, 2 H, Ar-H), 6.92 (d, *J* = 9.6 Hz, 1 H, =CH), 4.08 (q, *J* = 7.5 Hz, 1 H, CH), 3.74 (s, 3 H, CO_2Me), 2.24 (brs, 1 H, OH), 1.67-1.03 (m, 20 H, 10 x CH_2), 0.88 (t, *J* = 6.8 Hz, 3 H, CH_3); **^{13}C NMR** (100 MHz, $CDCl_3$): δ = 167.0, 145.8, 133.4, 132.9, 131.2, 131.1, 122.0, 68.5, 52.3, 36.7, 31.8, 29.54, 29.53, 29.48, 29.4, 29.30, 29.26, 24.9, 22.6, 14.0; **MS** (EI 70 ev) *m/z* (%): 426 ($M^+(^{81}Br)$, 0.96), 424 ($M^+(^{79}Br)$, 1.12), 43 (100); **IR** (neat): *v* = 3428, 2922, 2852, 1720, 1487, 1435, 1236, 1071, 1032, 1010 cm^{-1}; **HRMS** calcd for $C_{22}H_{33}{}^{79}BrO_3$ $[M]^+$: 424.1613, found: 424.1615.

(31) Preparation of methyl (*R, E*)-4-hydroxy-2,4-diphenylbut-2-enoate ((*R, E*)-2v) (hcf-2-132)

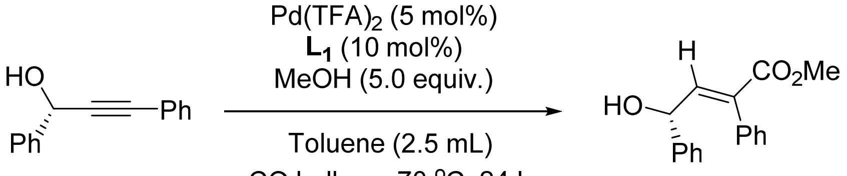

(*S*)-**1v**
>99% ee

(*R*, *E*)-**2v**, 68%
99% ee

Following **Typical Procedure IV**, the reaction of Pd(TFA)$_2$ (8.5 mg, 0.025 mmol), $\mathbf{L_1}$ (27.3 mg, 0.05 mmol), (*S*)-**1v** (104.2 mg, >99% ee, 0.5 mmol)/toluene (2.0 mL), and MeOH (80.9 mg, 2.5 mmol)/toluene (0.5 mL) afforded (*R*, *E*)-**2v** (91.3 mg, 68%) as a yellow oil [eluent: petroleum ether/ethyl acetate = 10/1 (550 mL), 5/1 (600 mL)]: 99% ee (HPLC conditions: PA-2 column, hexane/*i*-PrOH = 80/20, 0.7 mL/min, λ = 214 nm, t_R (major) = 14.0 min, t_R (minor) = 17.0 min); $[\alpha]^{20}_D$ = +157.6 (*c* = 1.01, $CHCl_3$); **^{1}H NMR** (400 MHz, $CDCl_3$): δ = 7.48-7.24 (m, 8 H, Ar-H), 7.24-7.11 (m, 3 H, Ar-H and =CH), 5.19 (d, *J* = 9.2 Hz, 1 H, CH), 3.73 (s, 3 H, CO_2Me), 2.13 (br, 1 H, OH); **^{13}C NMR** (100 MHz, $CDCl_3$): δ = 167.4, 143.4, 141.7, 134.3, 134.0, 129.5, 128.8, 128.2, 128.1, 126.3, 71.0, 52.3; **MS** (EI 70 ev) *m/z* (%): 269 (M^++1, 1.51), 268 (M^+, 7.65), 105 (100); **IR** (neat): ν = 3417, 1696, 1641, 1490, 1442, 1228 cm^{-1}; **HRMS** calcd for $C_{17}H_{16}O_3$ $[M]^+$: 268.1099, found: 268.1097.

(32) Preparation of methyl (*R*, *E*)-4-hydroxy-2-(4-methoxyphenyl)-4-phenylbut-2-enoate ((*R*, *E*)-2w) (hcf-2-152)

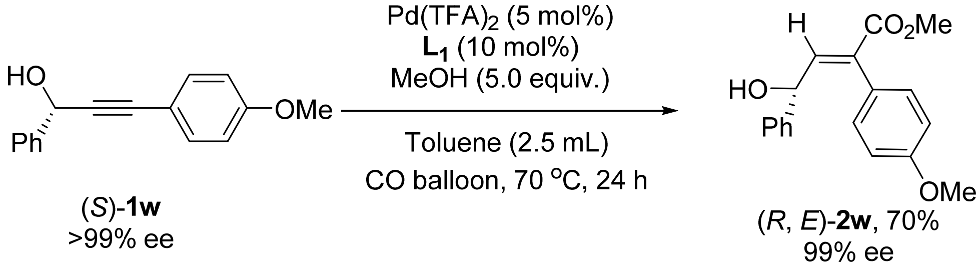

(*S*)-**1w**
>99% ee

(*R*, *E*)-**2w**, 70%
99% ee

Following **Typical Procedure IV**, the reaction of Pd(TFA)$_2$ (8.5 mg, 0.025 mmol), $\mathbf{L_1}$ (27.3 mg, 0.05 mmol), (*S*)-**1w** (119.1 mg, >99% ee, 0.5 mmol)/toluene (2.0 mL), and MeOH (81.1 mg, 2.5 mmol)/toluene (0.5 mL) afforded (*R*, *E*)-**2w** (104.4 mg, 70%) as a yellow oil [eluent: petroleum ether/ethyl acetate = 10/1 (440 mL), 5/1 (600 mL)]: 99% ee (HPLC conditions: PC-2 column, hexane/*i*-PrOH = 50/50, 0.8 mL/min, λ = 214 nm, t_R (major) = 7.9 min, t_R (minor) = 13.0 min); $[\alpha]^{21}_D$ = +196.5 (*c* = 0.80, $CHCl_3$); **^{1}H NMR** (400 MHz, $CDCl_3$): δ = 7.41-7.22 (m, 5 H, Ar-H), 7.20-7.06 (m, 3 H, Ar-H and =CH), 6.90 (d, *J* = 8.4 Hz, 2 H, Ar-H), 5.20 (d, *J* = 9.2 Hz, 1 H, CH), 3.81 (s, 3 H, CO_2Me), 3.71 (s, 3 H, OMe), 2.62-2.34 (m, 1 H, OH); **^{13}C NMR** (100 MHz, $CDCl_3$): δ = 167.6, 159.2, 143.2, 141.9, 133.5, 130.8, 128.7, 128.0, 126.4, 126.3, 113.5, 70.9, 55.2, 52.3; **MS** (EI 70 ev) *m/z* (%): 299 (M^++1, 1.90), 298 (M^+,

9.21), 105 (100); **IR** (neat): v = 3424, 3015, 2947, 2837, 1707, 1508, 1444, 1234, 1174, 1017 cm^{-1}; **HRMS** calcd for $C_{18}H_{18}O_4$ $[M]^+$: 298.1205, found: 298.1202.

(33) Preparation of methyl (*S*, *E*)-4-hydroxy-4-methyl-2-phenylnon-2-enoate ((*S*, *E*)-2k) (hcf-2-128)

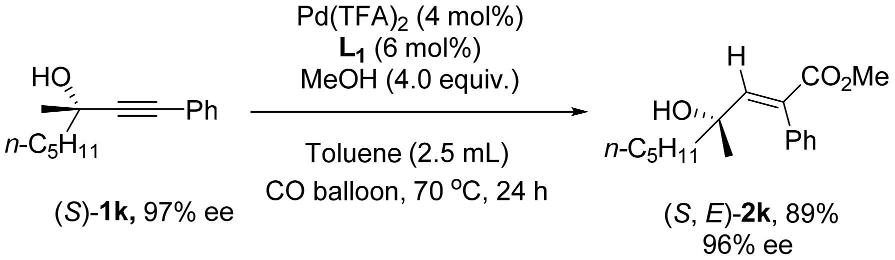

Following **Typical Procedure IV**, the reaction of $Pd(TFA)_2$ (6.8 mg, 0.02 mmol), $\mathbf{L_1}$ (16.4 mg, 0.03 mmol), (*S*)-**1k** (108.2 mg, 97% ee, 0.5 mmol)/toluene (2.0 mL), and MeOH (64.9 mg, 2 mmol)/toluene (0.5 mL) afforded (*S*, *E*)-**2k** (123.1 mg, 89%) as a yellow oil [eluent: petroleum ether/ethyl acetate = 20/1 (420 mL), 10/1 (440 mL)]: 96% ee (HPLC conditions: PA-2 column, hexane/*i*-PrOH = 95/5, 1.0 mL/min, λ = 214 nm, t_R (major) = 18.2 min, t_R (minor) = 13.9 min); $[\alpha]^{20}_D$ = +15.6 (c = 1.01, $CHCl_3$); **^{1}H NMR** (400 MHz, $CDCl_3$): δ = 7.42-7.30 (m, 3 H, Ar-H), 7.24-7.16 (m, 2 H, Ar-H), 7.08 (s, 1 H, =CH), 3.70 (s, 3 H, CO_2Me), 1.57-1.44 (m, 2 H, CH_2), 1.44-1.16 (m, 10 H, 3 x CH_2 and CH_3 and OH), 0.88 (t, J = 7.4 Hz, 3 H, CH_3); **^{13}C NMR** (100 MHz, $CDCl_3$): δ = 167.9, 149.6, 135.0, 131.3, 129.4, 128.1, 127.8, 74.2, 52.3, 43.1, 32.0, 28.9, 23.6, 22.5, 14.0; **MS** (ESI) m/z: 299 $(M+Na)^+$, 259 $(M-OH)^+$; **IR** (neat): v = 3495, 2953, 2931, 2860, 1716, 1458, 1435, 1237, 1196, 1020 cm^{-1}; **HRMS** calcd for $C_{17}H_{24}O_3$ $[M]^+$: 276.1725, found: 276.1722.

6. Synthetic applications

(1) Preparation of methyl (*E*)-4-hydroxy-2-(4'-methoxy-[1,1'-biphenyl]-4-yl)-4-methylnon-2-enoate ((*E*)-7)[5] (hcf-2-98)

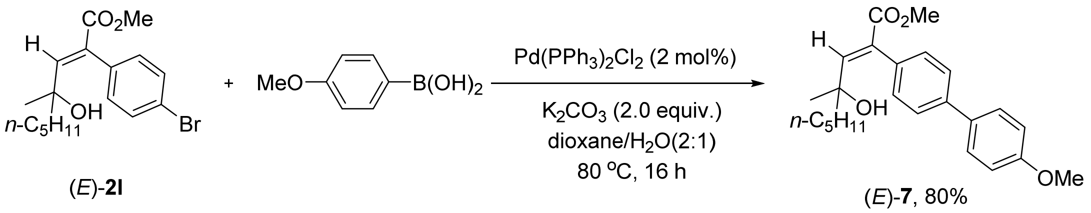

To a flame-dried Schlenk tube were added $Pd(PPh_3)_2Cl_2$ (7.0 mg, 0.01 mmol), (4-methoxyphenyl)boronic acid (116.3 mg, 0.75 mmol), potassium carbonate (138.2 mg, 1.0 mmol), (*E*)-**2l** (177.5 mg, 0.5 mmol)/1,4-doxane (2.5 mL), and H_2O (1.25 mL) sequentially under argon. The resulting mixture was stirred at 80 ^{o}C for 16 h and then diluted with 3 mL of ethyl acetate, filtered through a short column silica gel (3 cm) eluted with ethyl acetate (20 x 2 mL), and concentrated. The residue was purified by column chromatography on silica gel to afford (*E*)-**7** (152.8 mg, 80%) as a yellow oil [eluent: petroleum ether/ethyl acetate = 50/1 (510 mL), 20/1 (525 mL), 10/1 (550 mL), 5/1 (240 mL)]: ^{1}**H NMR** (400 MHz, $CDCl_3$): δ = 7.60-7.49 (m, 4 H, Ar-H), 7.31-7.20 (m, 2 H, Ar-H), 7.10 (s, 1 H, =CH), 6.98 (d, *J* = 8.8 Hz, 2 H, Ar-H), 3.85 (s, 3 H, CO_2Me), 3.73 (s, 3 H, OMe), 1.60-1.49 (m, 2 H, CH_2), 1.45 (s, 1 H, OH), 1.42-1.18 (m, 9 H, 3 x CH_2 and CH_3), 0.89 (t, *J* = 7.0 Hz, 3 H, CH_3); ^{13}**C NMR** (100 MHz, $CDCl_3$): δ = 168.0, 159.2, 149.7, 140.3, 133.2, 133.0, 131.1, 129.9, 128.1, 126.4, 114.2, 74.3, 55.3, 52.4, 43.2, 32.1, 29.0, 23.7, 22.6, 14.0; **MS** (EI 70 ev) *m/z* (%): 383 (M^++1, 6.42), 382 (M^+, 24.86), 279 (100); **IR** (neat): *v* = 3509, 2952, 2931, 2859, 1713, 1608, 1497, 1241, 1176, 1038 cm^{-1}; **HRMS** calcd for $C_{24}H_{30}O_4$ $[M]^+$: 382.2144, found: 382.2141.

(2) Preparation of (*E*)-2-(4-bromophenyl)-4-hydroxy-4-methylnon-2-enoic acid ((*E*)-8)[6] (hcf-2-130)

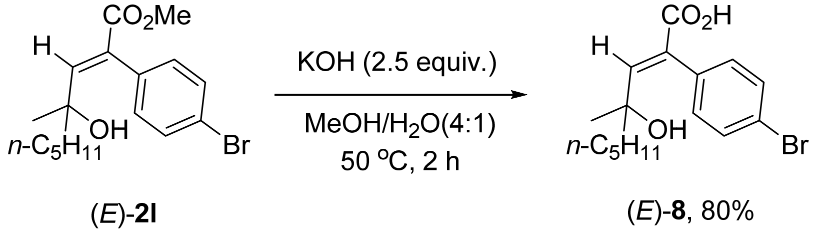

To a flame-dried Schlenk tube were added KOH (70.1 mg, 1.25 mmol), (*E*)-**2l** (177.6 mg, 0.5 mmol)/MeOH (1 mL), and H_2O (0.25 mL) sequentially. The Schlenk

tube was placed in an oil bath pre-heated at 50 °C and stirred for 2 h. To the mixture was added H_2O (5 mL), the organic layer was then washed with 3 N HCl (10 mL), extracted with ethyl acetate (5 mL x 3), washed with brine (10 mL), and dried over anhydrous Na_2SO_4. After filtration and concentration under reduced pressure, the crude product was purified by column chromatography on silica gel to afford (*E*)-**8** (136.5 mg, 80%) as a white solid [eluent: dichloromethane (200 mL) to dichloromethane/MeOH = 20:1]: m.p. 123.7-124.4 °C (petroleum ether/dichloromethane); **^{1}H NMR** (400 MHz, DMSO-D_6): δ = 12.53 (s, 1 H, COOH), 7.49 (d, *J* = 8.4 Hz, 2 H, Ar-H), 7.05 (d, *J* = 8.0 Hz, 2 H, Ar-H), 6.98 (s, 1 H, =CH), 4.50 (s, 1 H, OH), 1.38-1.06 (m, 8 H, 4 x CH_2), 1.00 (s, 3 H, CH_3), 0.83 (t, *J* = 7.4 Hz, 3 H, CH_3); **^{13}C NMR** (100 MHz, DMSO-D_6): δ = 168.4, 151.2, 135.9, 131.9, 130.5, 130.3, 120.3, 72.2, 42.5, 31.9, 28.6, 23.3, 22.2, 14.1; **MS** (ESI) m/z: 325 $(M(^{81}Br)-OH)^+$, 323 $(M(^{79}Br)-OH)^+$; **IR** (neat): *v* = 3387, 2961, 2926, 2870, 2855, 2584, 1693, 1630, 1488, 1232, 1148, 1009 cm^{-1}; Anal. Calcd. for $C_{16}H_{21}{}^{79}BrO_3$: C 56.32, H 6.20; found C 55.95, H 6.25.

(3) Preparation of (*E*)-2-(4-bromophenyl)-4-hydroxy-4-methylnon-2-enoic acid ((*E*)-9)[7] (hcf-2-134)

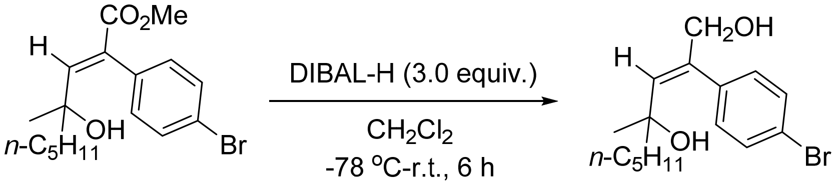

(*E*)-**2l** (*E*)-**9**, 80%

To a flame-dried Schlenk tube were added (*E*)-**2l** (177.6 mg, 0.5 mmol)/ dichloromethane (5 mL). Then the resulting mixture was stirred at -78 °C with a balloon of argon for 10 min. After that, DIBAL-H (1.0 M in toluene, 1.5 mL, 1.5 mmol, 3.0 equiv.) was added dropwise within 5 mins. After 3 hours, the resulting mixture was warmed up to room temperature and stirred at room temperature for 3h as monitored by TLC. A saturated aqueous solution of potassium sodium tartrate (10 mL) was added with a cooling bath of ice-water. The resulting mixture was then stirred at room temperature for 2 h. The organic layer was separated, and the aqueous layer was extracted with DCM (3 x 5 mL). The combined organic layer was washed

with brine (10 mL) and dried with anhydrous Na_2SO_4. After filtration and concentration under reduced pressure, the crude product was purified by column chromatography on silica gel to afford (*E*)-**9** (130.8 mg, 80%) as a yellow oil [eluent: petroleum ether/ethyl acetate = 10/1 (550 mL), 5/1 (600 mL), 3/1 (200 mL)]: **^{1}H NMR** (400 MHz, $CDCl_3$): δ = 7.48 (d, *J* = 8.4 Hz, 2 H, Ar-H), 7.09 (d, *J* = 8.0 Hz, 2 H, Ar-H), 5.78 (s, 1 H, =CH), 4.17 (d, *J* = 5.2 Hz, 2 H, $=CH_2$), 1.62-1.51 (m, 1 H, OH), 1.51-1.40 (m, 2 H, CH_2), 1.37-1.17 (m, 9 H, 3 x CH_2 and CH_3), 1.16 (s, 1 H, OH), 0.89 (t, *J* = 7.0 Hz, 3 H, CH_3); **^{13}C NMR** (100 MHz, DMSO-D_6): δ = 137.8, 137.5, 134.3, 131.4, 130.4, 121.5, 73.9, 68.9, 43.7, 32.2, 29.5, 23.7, 22.6, 14.1; **MS** (ESI) m/z: 351 $(M(^{81}Br)+Na)^+$, 349 $(M(^{79}Br)+Na)^+$, 293 $(M(^{81}Br)\text{-}OH\text{-}H_2O)^+$, 291 $(M(^{79}Br)\text{-}OH\text{-}H_2O)^+$; **IR** (neat): *v* = 3334, 2954, 2929, 2858, 1897, 1710, 1587, 1486, 1460, 1375, 1148, 1071, 1011 cm^{-1}; **HRMS** calcd for $C_{16}H_{23}{}^{79}BrO_2Na$ $[M+Na]^+$: 349.0774, found: 349.0762.

(4) Preparation of methyl 4-fluoro-4-methyl-2-(*E*)-2-(4-bromophenyl)-non-2-enoate ((*E*)-10)[8] (hcf-2-158)

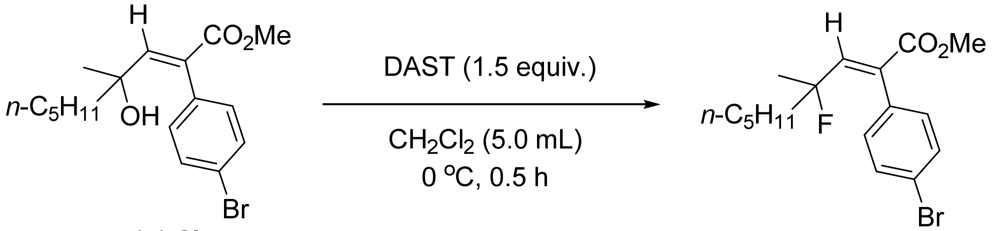

(*E*)-**2l** (*E*)-**10**, 94%

To a flame-dried Schlenk tube was added a solution of (*E*)-**2l** (177.9 mg, 0.5 mmol)/dichloromethane (5 mL) under argon. The resulting mixture was stirred at 0 °C for 10 min. The resulting mixture was added DAST (0.1 mL, d = 1.22 g/mL, 122.0 mg, 0.75 mmol) and stirred at 0 °C for 30 min. After that, the reaction mixture was poured into a cold sat. $NaHCO_3$ solution (30 mL) and extracted with CH_2Cl_2 (3 x 10 mL), washed with brine (10 mL), and dried over anhydrous Na_2SO_4. After filtration and concentration under reduced pressure, the crude product was purified by column chromatography on silica gel to afforded (*E*)-**10** (168.1 mg, 94%) as a yellow oil [eluent: petroleum ether/ethyl ether/dichloromethane = 150/1/1]: **^{1}H NMR** (400 MHz, $CDCl_3$): δ = 7.46 (d, *J* = 8.0 Hz, 2 H, Ar-H), 7.15-6.96 (m, 3 H,

Ar-H and =CH), 3.72 (s, 3 H, CO_2Me), 1.71-1.49 (m, 2 H, CH_2), 1.47-1.14 (m, 9 H, 3 x CH_2 and CH_3), 0.88 (t, J = 7.2 Hz, 3 H, CH_3); **^{13}C NMR** (100 MHz, $CDCl_3$): δ = 167.3, 145.8 (d, J = 23.5 Hz), 134.1, 131.7 (d, J = 4.6 Hz), 130.9 (d, J = 2.2 Hz), 130.7, 121.6, 96.2 (d, J = 173.8 Hz), 52.5, 40.6 (d, J = 22.8 Hz), 31.8, 26.1 (d, J = 24.2 Hz), 23.2 (d, J = 3.1 Hz), 22.5, 14.0; **^{19}F NMR** (376 MHz, CDCl3): δ = -143.1; **MS** (EI, 70 ev) *m/z* (%): 358 (M^+(^{81}Br), 45.13), 356 (M^+(^{79}Br), 45.11), 146 (100); **IR** (neat): ν = 2943, 2862, 1718, 1482, 1442, 1241, 1065, 1014 cm^{-1}; **HRMS** calcd for $C_{17}H_{22}{}^{79}BrFO_2$ $[M]^+$: 356.0787, found: 356.0781.

7. Mechanistic studies:

(1) Reaction of methyl ether 3 (hcf-2-154)

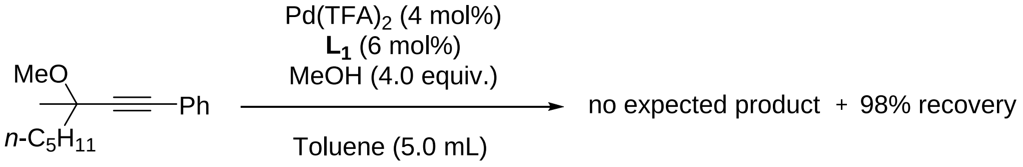

3

Following **Typical Procedure IV**, the reaction of $Pd(TFA)_2$ (13.6 mg, 0.04 mmol), $\mathbf{L_1}$ (32.8 mg, 0.06 mmol), **3** (230.4 mg, 1 mmol)/toluene (4.0 mL), and MeOH (130.0 mg, 4 mmol)/toluene (1.0 mL) was conducted for 24 h at 60 °C. Crude ^{1}H NMR showed that no expected product with 98% recovery of **3**.

(2) Reaction of acetate 4 (hcf-2-150)

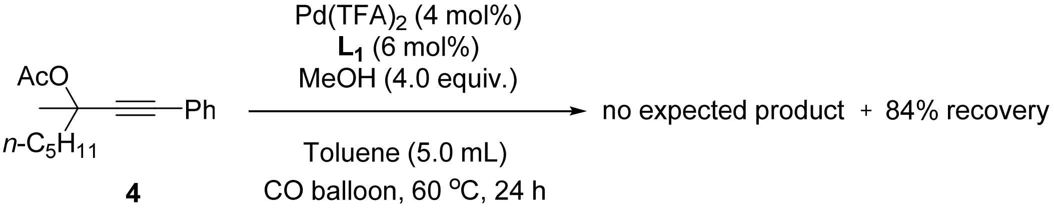

4

Following **Typical Procedure IV**, the reaction of $Pd(TFA)_2$ (13.6 mg, 0.04 mmol), $\mathbf{L_1}$ (32.8 mg, 0.06 mmol), **4** (258.6 mg, 1 mmol)/toluene (4.0 mL), and MeOH (129.7 mg, 4 mmol)/toluene (1.0 mL) was conducted for 24 h at 60 °C. Crude ^{1}H NMR showed that no expected product with 84% recovery of **4**.

(3) Reaction of internal alkyne 5 (hcf-2-176)

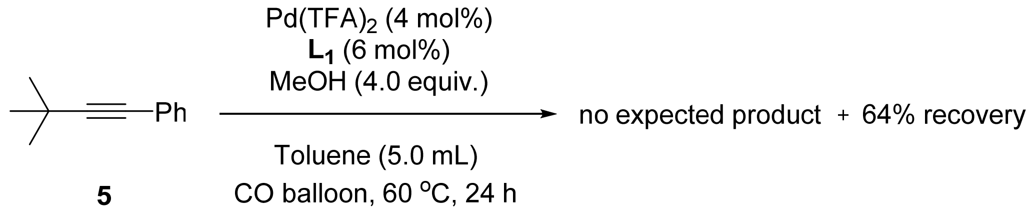

Following **Typical Procedure IV**, the reaction of $Pd(TFA)_2$ (13.6 mg, 0.04 mmol), $\mathbf{L_1}$ (32.8 mg, 0.06 mmol), **5** (158.1 mg, 1 mmol)/toluene (4.0 mL), and MeOH (130.5 mg, 4 mmol)/toluene (1.0 mL) was conducted for 24 h at 60 °C. Crude ^{1}H NMR showed that no expected product with 64% recovery of **5**.

(4) Preparation of 1k-*d* (hcf-3-169, hcf-3-93)

hcf-3-169:

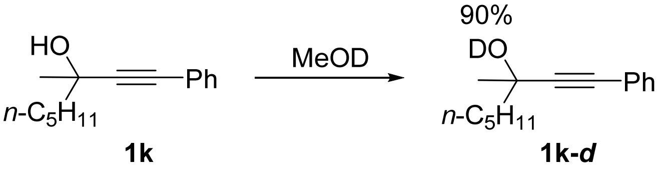

To a solution of **1k** (649.9 mg, 3 mmol) was added MeOD (1 mL) under argon. After shaking the flask at room temperature, the mixture was evaporated under vacuum directly using the vacuum line connected to argon for 30 min. Repeating the process five times afforded **1k-*d*** as an oil with 90% D incorporation: **^{1}H NMR** (400 MHz, $CDCl_3$): δ = 7.45-7.37 (m, 2 H, Ar-H), 7.34-7.27 (m, 3 H, Ar-H), 1.79-1.69 (m, 2 H, CH_2), 1.63-1.49 (m, 5 H, CH_2 and CH_3), 1.41-1.29 (m, 4 H, 2 x CH_2), 0.91 (t, J = 7.0 Hz, 3 H, CH_3). The following signal is discernible for **1k**: δ 2.02 (s, 1 H, OH).

hcf-3-93:

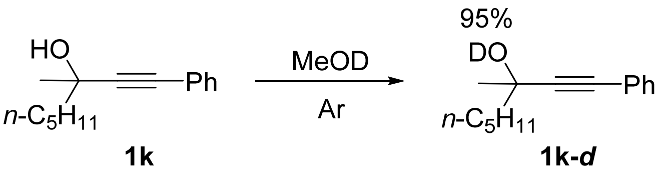

To a solution of **1k** (1.0831 g, 5 mmol) was added MeOD (2 mL) under argon. After shaking the flask at room temperature, the mixture was evaporated under vacuum directly using the vacuum line connected to argon for 30 min. Repeating the process five times afforded **1k-*d*** as an oil with 95% D incorporation: **^{1}H NMR** (400 MHz, $CDCl_3$): δ = 7.46-7.37 (m, 2 H, Ar-H), 7.34-7.27 (m, 3 H, Ar-H), 1.80-1.69 (m,

2 H, CH_2), 1.64-1.46 (m, 5 H, CH_2 and CH_3), 1.43-1.27 (m, 4 H, 2 x CH_2), 0.91 (t, J = 6.4 Hz, 3 H, CH_3). The following signal is discernible for **1k**: δ 2.06 (s, 1 H, OH).

(5) Reaction of 1k with MeOD (hcf-2-182B)

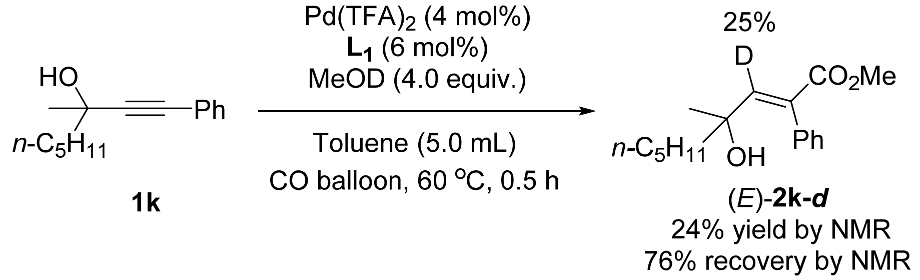

To a flame-dried Schlenk tube were added $Pd(TFA)_2$ (13.6 mg, 0.04 mmol), $\mathbf{L_1}$ (32.8 mg, 0.06 mmol), **1k** (216.2 mg, 1 mmol)/toluene (4 mL), and MeOD (132.7 mg, 4.0 mmol)/toluene (1 mL) sequentially under argon. The resulting mixture was then frozen with a liquid nitrogen bath, degassed to remove the argon inside completely, and refilled with CO by a balloon of CO for three times. Then the resulting mixture was stirred at 60 °C with a balloon of CO for 0.5 h. After that, the resulting mixture was diluted with 3 mL of ethyl acetate, filtered through a short column of silica gel (3 cm) eluted with ethyl acetate (20 x 2 mL), and concentrated to afford the crude product. (*E*)-**2k-*d*** (25% deuterium incorporation) was formed as determined by NMR analysis of the crude product by using CH_2Br_2 as the internal standard.

(6) Reaction of 1k-*d* with MeOD (hcf-3-170)

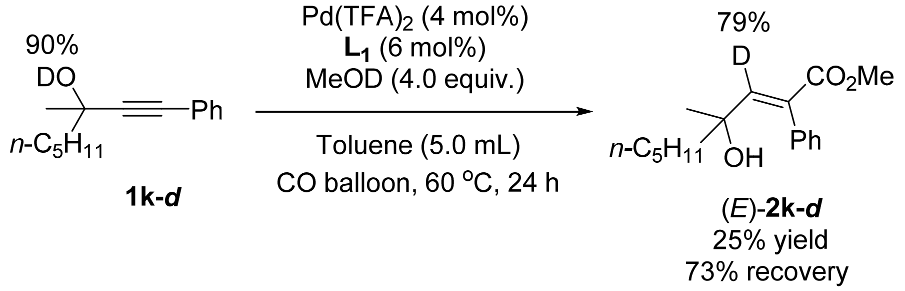

To a flame-dried Schlenk tube were added $Pd(TFA)_2$ (13.6 mg, 0.04 mmol), $\mathbf{L_1}$ (32.8 mg, 0.06 mmol), **1k-*d*** (217.1 mg, 1 mmol, 90% deuterium incorporation)/toluene (4 mL), MeOD (132.9 mg, 4.0 mmol)/toluene (1 mL) sequentially in a glove box. The resulting mixture was then frozen with a liquid nitrogen bath, degassed to remove the

nitrogen inside completely, and refilled with CO by a balloon of CO for three times. Then the resulting mixture was stirred at 60 ^{o}C with a balloon of CO for 24 h. After that, the resulting mixture was diluted with 3 mL of ethyl acetate, filtered through a short column of silica gel (3 cm) eluted with ethyl acetate (20 x 2 mL), and concentrated. The residue was purified by column chromatography on silica gel to afford (*E*)-**2k-*d*** (69.7 mg, 25%) as a yellow oil [eluent: petroleum ether/ethyl acetate = 20/1 (630 mL), 10/1 (220 mL)]: **^{1}H NMR** (400 MHz, $CDCl_3$): δ = 7.41-7.29 (m, 3 H, Ar-H), 7.24-7.14 (m, 2 H, Ar-H), 3.69 (s, 3 H, CO_2Me), 1.56-1.44 (m, 2 H, CH_2), 1.44-1.14 (m, 10 H, 3 x CH_2 and CH_3 and OH), 0.88 (t, J = 7.0 Hz, 3 H, CH_3), the following signal is discernible for (*E*)-**2k**: δ 7.08 (s, 1 H, =CH); **^{13}C NMR** (100 MHz, $CDCl_3$): δ = 167.9, 149.2 (J = 23.3 Hz), 135.0, 131.3, 129.4, 128.1, 127.8, 74.1, 52.3, 43.1, 32.0, 28.9, 23.6, 22.5, 14.0; the following signal is discernible for (*E*)-**2k**: 149.6, 135.05, 131.4, 74.2; **MS** (ESI) *m/z*: 300 $(M+Na)^+$, 260 $(M-OH)^+$; **IR** (neat): v = 3487, 2954, 2931, 2860, 1717, 1434, 1226, 1022 cm^{-1}; **HRMS** calcd for $C_{17}H_{23}DO_3Na$ $[M+Na]^+$: 300.1680, found: 300.1672.

(7) ^{1}H-NMR spectra of 1k with different amount of methanol (hcf-2-188)

1k (21.6 mg, 0.1 mmol) was dissolved in Tol-d_8 (0.5 mL), the ^{1}H-NMR spectrum was shown as hcf-2-188-1. Then, 1 equiv. of MeOH (4 μL, 0.1 mmol) was added to obtain ^{1}H-NMR spectra as hcf-2-188-2. This operation was repeated for three times to the same NMR tube, the spectra are hcf-2-188-3, hcf-2-188-4, and hcf-2-188-5. An obvious shift of the hydroxy signal was observed.

(8) ^{1}H-NMR spectra of methanol with different amount of 1k (hcf-2-188C)

Methanol (16 μL, 0.4 mmol) was dissolved in Tol-d_8 (0.5 mL), the ^{1}H-NMR spectrum was shown as hcf-2-188C-1. Then, 0.25 equiv. of **1k** (5.4 mg, 0.1 mmol) was added to obtain ^{1}H-NMR spectra as hcf-2-188C-5. This operation was repeated for three times to the same NMR tube, the spectra are hcf-2-188C-6, hcf-2-188C-7, and hcf-2-188C-8, an obvious shift of the hydroxy signal was observed.

(9) Kinetic Experiments (hcf-3-161)

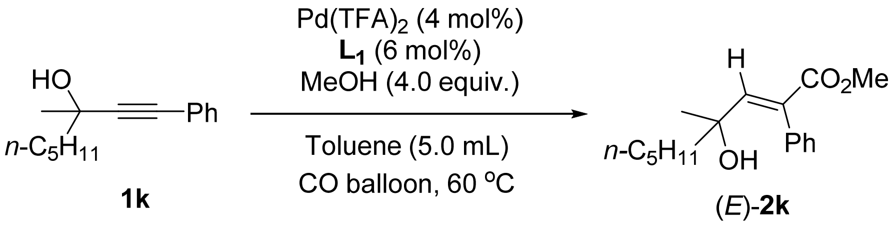

To a flame-dried Schlenk tube were added $Pd(TFA)_2$ (13.6 mg, 0.04 mmol), $\mathbf{L_1}$ (32.8 mg, 0.06 mmol), **1k** (216.3 mg, 1 mmol)/toluene (2 mL), MeOH (128.8 mg, 4.0 mmol)/toluene (2 mL), and diphenylmethane (the internal standard, 83.6 μL, d = 1.006 g/mL, 84.1 mg, 0.5 mmol)/toluene (1 mL) sequentially under argon. The resulting mixture was then frozen with a liquid nitrogen bath, degassed to remove the argon inside completely, and refilled with CO by a balloon of CO for three times. Then the resulting mixture was stirred at 60 ^{o}C with a balloon of CO and monitored at different time (see Table S4). The yields were determined by ^{1}H NMR measurement using diphenylmethane as the internal standard.

Table S4. For **1k**:

Time / h	Yield of (*E*)-**2k**
2	40
4	54
6	65
8	73
12	84
16	90
20	95
24	97

A linear relationship was obtained for ln $\{c_0/(c_0-[(E)\text{-}\mathbf{2k}])\}$ vs. reaction time, indicating a first-order dependence of reaction rate with respect to propargylic alcohol in the rate-determining step (Figures S3).

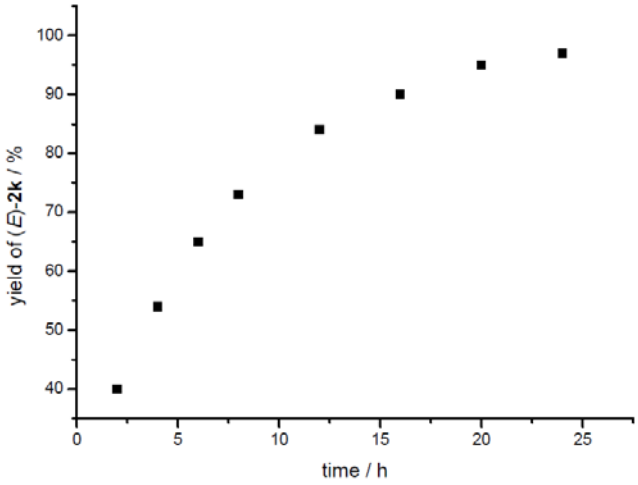

(a) yield of (*E*)-**2k** vs. time

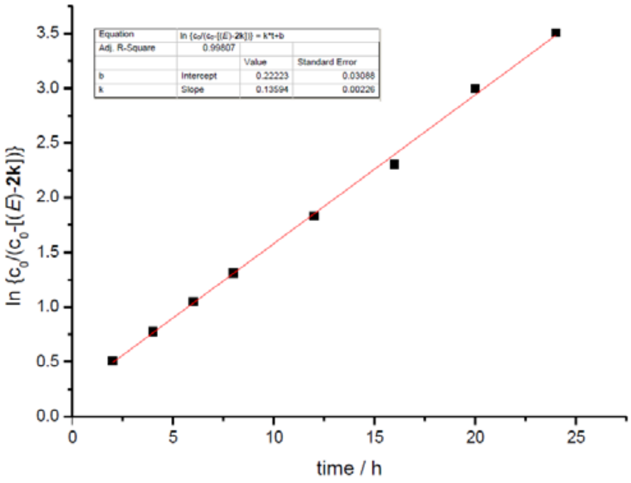

(b) ln {c_0/(c_0-[(*E*)-**2k**])} vs. time (R-square is the coefficient of determination)

Figure S3. Determination of the reaction order on propargylic alcohol

(10) Kinetic Experiments (hcf-3-162)

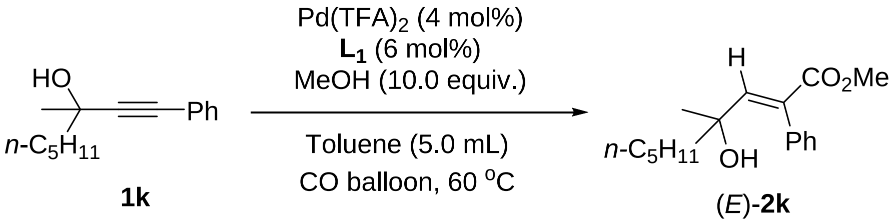

To a flame-dried Schlenk tube were added Pd(TFA)$_2$ (13.6 mg, 0.04 mmol), **L$_1$** (32.8 mg, 0.06 mmol), **1k** (216.3 mg, 1 mmol)/toluene (2 mL), MeOH (321.4 mg, 10.0 mmol)/toluene (2 mL), and diphenylmethane (the internal standard, 83.6 μL, d = 1.006 g/mL, 84.1 mg, 0.5 mmol)/toluene (1 mL) sequentially under argon. The resulting mixture was then frozen with a liquid nitrogen bath, degassed to remove the

argon inside completely, and refilled with CO by a balloon of CO for three times. Then the resulting mixture was stirred at 60 ^{o}C with a balloon of CO and monitored at different time (see Table S5). The yields were determined by ^{1}H NMR measurement using diphenylmethane as the internal standard.

Table S5. For **1k**:

Time / h	Yield of (*E*)-**2k**
2	67
4	80
6	86
8	90
12	95
16	98

A linear relationship was obtained for ln {c_0/(c_0-[(*E*)-**2k**])} vs. reaction time, indicating a first-order dependence of reaction rate with respect to propargylic alcohol in the rate-determining step (Figures S4).

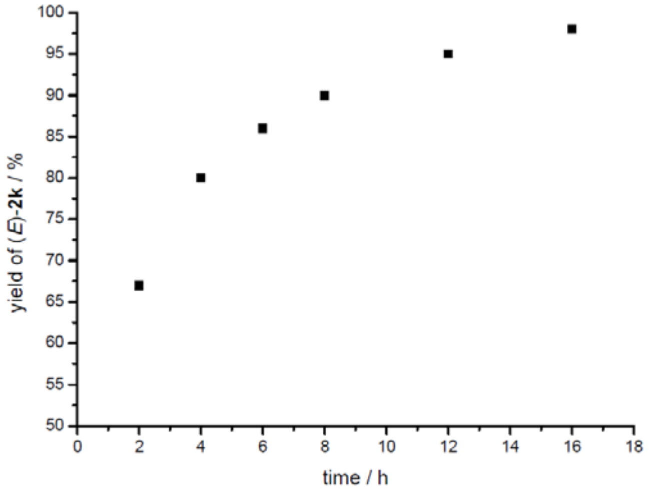

(a) yield of (*E*)-**2k** vs. time

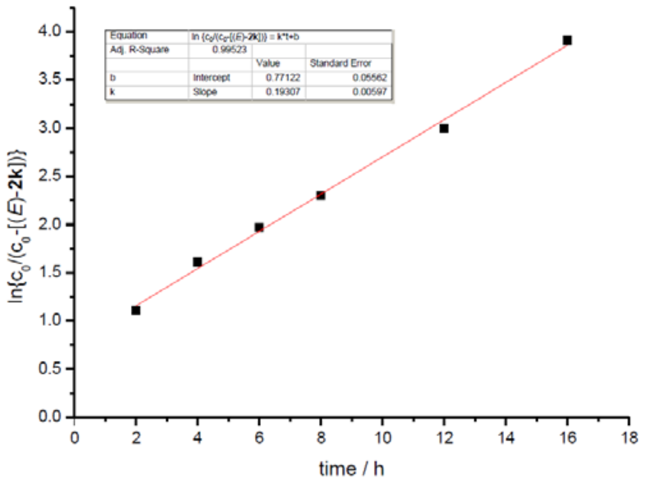

(b) ln {c_0/(c_0-[(*E*)-**2k**])} vs. time (R-square is the coefficient of determination)

Figure S4. Determination of the reaction order on propargylic alcohol-10.0-fold MeOH

(11) Measuring the rate of H/D-scrambling (hcf-3-109)

To a solution of 1k (8.2 mg, 0.038 mmol) in $CDCl_3$ (1 mL) was added MeOD (6.5 μL, 0.813 g/mL, 0.16 mmol), then the mixture was subjected to ^{1}H NMR analysis immediately, the H/D-exchange process was found to reach equilibrium state within 3 minutes.

(12) Kinetic Isotope Effect (KIE) Experiments (hcf-3-99, 100)

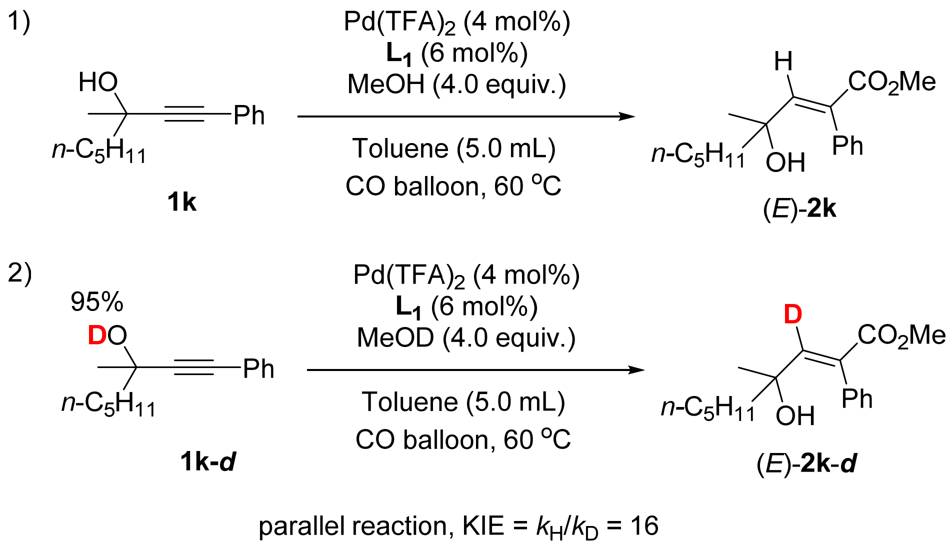

To a flame-dried Schlenk tube were added $Pd(TFA)_2$ (13.6 mg, 0.04 mmol), **L₁** (32.8 mg, 0.06 mmol), **1k** (216.3 mg, 1 mmol)/toluene (3 mL), MeOH (128.7 mg, 4.0 mmol)/toluene (1 mL), and diphenylmethane (the internal standard, 83.6 μL, d =

1.006 g/mL, 84.1 mg, 0.5 mmol)/toluene (1 mL) sequentially in a glove box. The resulting mixture was then frozen with a liquid nitrogen bath, degassed to remove the nitrogen inside completely, and refilled with CO by a balloon of CO for three times. Then the resulting mixture was stirred at 60 °C with a balloon of CO and monitored at different time (see Table S6). The yields were determined by ^{1}H NMR measurement using diphenylmethane as the internal standard.

Table S6. For **1k**:

Time/h	1	1.5	2	3	4
NMR yield of (*E*)-**2k**/%	27	32	36	43	53

Due to the nature of the experiment, plots to determine the KIE were taken for **1k** (Figure S5).

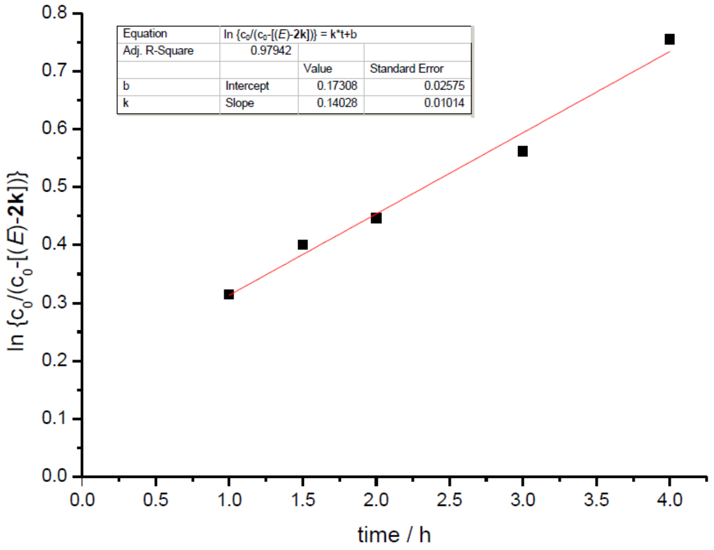

Figure S5. Linear function fit for reaction rate of **1k** to obtain k_H

To another dried Schlenk tube were added Pd(TFA)$_2$ (13.6 mg, 0.04 mmol), **L$_1$** (32.8 mg, 0.06 mmol), **1k-*d*** (217.2 mg, 1 mmol, 95% deuterium incorporation)/toluene (3 mL), MeOD (132.6 mg, 4.0 mmol)/toluene (1 mL), and diphenylmethane (the internal standard, 83.6 μL, d = 1.006 g/mL, 84.1 mg, 0.5 mmol)/toluene (1 mL) sequentially in a glove box. The resulting mixture was then frozen with a liquid nitrogen bath, degassed to remove the nitrogen inside completely, and refilled with CO by a balloon of CO for three times. Then the resulting mixture

was stirred at 60 °C with a balloon of CO and monitored at different time (see Table S7). The yields were determined by ^{1}H NMR measurement using diphenylmethane as the internal standard.

Table S7. For **1k-*d***:

Time/h	1.5	2	3	4	5
NMR yield of (*E*)-**2k-*d***/%	9.0	9.5	10.2	11	11.8

Due to the nature of the experiment, plots to determine the KIE were taken for **1k-*d*** (Figure S6).

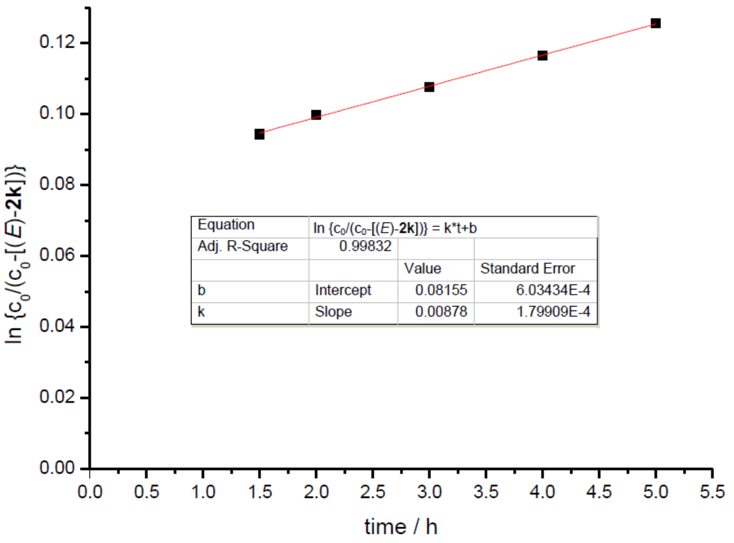

Figure S6. Linear function fit for reaction rate of **1k-*d*** to obtain k_D

Finally, the intermolecular isotope effect value is determined as 16.

$$k_H/k_D = (0.14028)/(0.00878) = 16$$

(13) Preparation of methyl (*E*)-2-(methoxycarbonylphenyl)-4-hydroxy-4-methylnon-2-enoate ((*E*)-2y) (hcf-3-155)

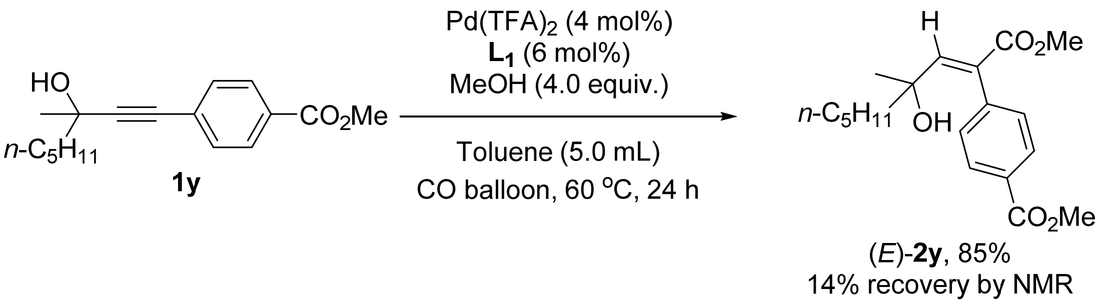

Following **Typical Procedure IV**, the reaction of Pd(TFA)$_2$ (13.6 mg, 0.04 mmol), **L$_1$** (32.8 mg, 0.06 mmol), **1y** (274.4 mg, 1 mmol)/toluene (4.0 mL), and

MeOH (128.8 mg, 4 mmol)/toluene (1.0 mL) afforded (*E*)-**2y** (282.9 mg, 85%) as a yellow oil [eluent: petroleum ether/ethyl acetate = 25/1 (520 mL), 10/1 (550 mL)]: **^{1}H NMR** (400 MHz, $CDCl_3$): δ = 8.03 (d, *J* = 8.0 Hz, 2 H, Ar-H), 7.28 (d, *J* = 8.0 Hz, 2 H, Ar-H), 7.10 (s, 1 H, =CH), 3.92 (s, 3 H, CO_2Me), 3.70 (s, 3 H, CO_2Me), 1.59-1.44 (m, 2 H, CH_2), 1.43-1.11 (m, 10 H, 3 x CH_2 and CH_3 and OH), 0.88 (t, *J* = 7.0 Hz, 3 H, CH_3); **^{13}C NMR** (100 MHz, $CDCl_3$): δ = 167.4, 166.7, 150.2, 140.6, 130.8, 129.6, 129.4, 129.1, 74.2, 52.4, 52.1, 43.1, 32.0, 28.8, 23.6, 22.5, 14.0; **MS** (70 eV, EI) *m/z* (%): 303 (M^+-OMe, 7.61), 231 (100); **IR** (neat): *v* = 3509, 2953, 2932, 2860, 1717, 1607, 1435, 1273, 1239, 1108, 1018 cm^{-1}; **HRMS** calcd for $C_{19}H_{26}O_5$ $[M]^+$: 334.1780, found: 334.1778.

(14) Preparation of methyl (*E*)-2-(4-methylphenyl)-4-hydroxy-4-methylnon-2-enoate ((*E*)-2z-A) (hcf-3-154)

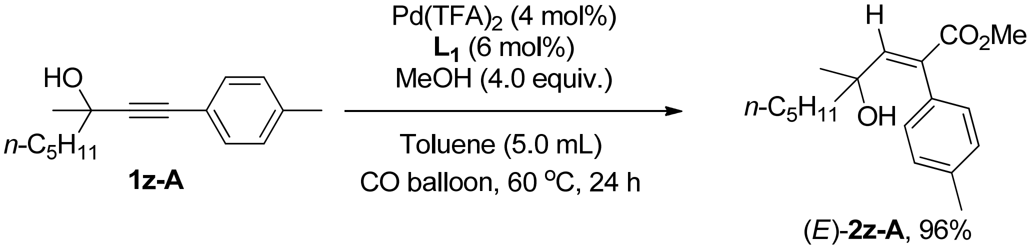

Following **Typical Procedure IV**, the reaction of $Pd(TFA)_2$ (13.6 mg, 0.04 mmol), **L_1** (32.8 mg, 0.06 mmol), **1z-A** (230.5 mg, 1 mmol)/toluene (4.0 mL), and MeOH (129.1 mg, 4 mmol)/toluene (1.0 mL) afforded (*E*)-**2z-A** (278.9 mg, 96%) as a yellow oil [eluent: petroleum ether/ethyl acetate = 20/1 (420 mL), 10/1 (440 mL)]: **^{1}H NMR** (400 MHz, $CDCl_3$): δ = 7.18 (d, *J* = 7.6 Hz, 2 H, Ar-H), 7.09 (d, *J* = 7.6 Hz, 2 H, Ar-H), 7.06 (s, 1 H, =CH), 3.70 (s, 3 H, CO_2Me), 2.36 (s, 3 H, CH_3), 1.57-1.46 (m, 2 H, CH_2), 1.46-1.40 (m, 1 H, OH), 1.40-1.17 (m, 9 H, 3 x CH_2 and CH_3), 0.88 (t, *J* = 7.2 Hz, 3 H, CH_3); **^{13}C NMR** (100 MHz, $CDCl_3$): δ = 168.1, 149.5, 137.7, 131.9, 131.3, 129.3, 128.9, 74.3, 52.3, 43.2, 32.1, 29.0, 23.6, 22.5, 21.2, 14.0; **MS** (70 eV, EI) *m/z* (%): 259 (M^+-OMe, 4.72), 187 (100); **IR** (neat): *v* = 3488, 2953, 2930, 2861, 1717, 1456, 1456, 1434, 1237, 1018 cm^{-1}; **HRMS** calcd for $C_{18}H_{26}O_3$ $[M]^+$: 290.1882, found: 290.1878.

(15) Preparation of methyl (*E*)-2-(4-methoxyphenyl)-4-hydroxy-4-methylnon-2-enoate ((*E*)-2z-B) (hcf-3-156)

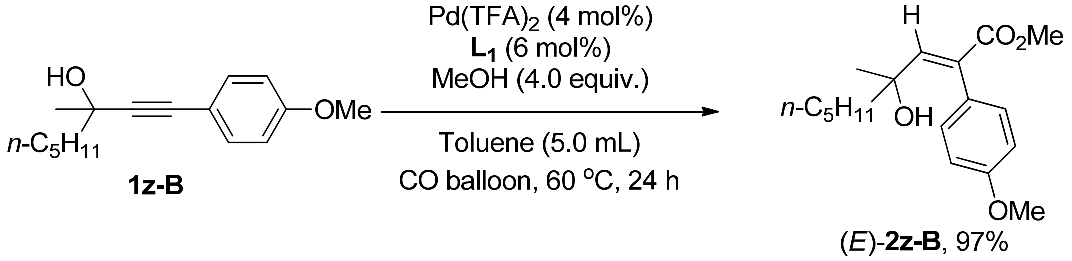

Following **Typical Procedure IV**, the reaction of $Pd(TFA)_2$ (13.6 mg, 0.04 mmol), $\mathbf{L_1}$ (32.8 mg, 0.06 mmol), **1z-B** (246.3 mg, 1 mmol)/toluene (4.0 mL), and MeOH (129.3 mg, 4 mmol)/toluene (1.0 mL) afforded (*E*)-**2z-B** (296.8 mg, 97%) as a yellow oil [eluent: petroleum ether/ethyl acetate = 25/1 (525 mL), 10/1 (550 mL)]: **^{1}H NMR** (400 MHz, $CDCl_3$): δ = 7.13 (d, *J* = 8.8 Hz, 2 H, Ar-H), 7.06 (s, 1 H, =CH), 6.90 (d, *J* = 8.8 Hz, 2 H, Ar-H), 3.81 (s, 3 H, OCH_3), 3.71 (s, 3 H, CO_2Me), 1.58-1.42 (m, 3 H, CH_2 and OH), 1.41-1.15 (m, 9 H, 3 x CH_2 and CH_3), 0.88 (t, *J* = 7.2 Hz, 3 H, CH_3); **^{13}C NMR** (100 MHz, $CDCl_3$): δ = 168.2, 159.2, 149.6, 131.0, 130.7, 126.7, 113.7, 74.3, 55.2, 52.3, 43.2, 32.1, 29.0, 23.6, 22.5, 14.0; **MS** (70 eV, EI) *m/z* (%): 306 (M^+, 4.39), 203 (100); **IR** (neat): *ν* = 3499, 2953, 2932, 2861, 1715, 1608, 1512, 1460, 1435, 1241, 1174 cm^{-1}; **HRMS** calcd for $C_{18}H_{26}O_4$ $[M]^+$: 306.1831, found: 306.1825.

(16) Electronic effect investigation (hcf-3-158, hcf-3-159, hcf-3-160, hcf-3-163)

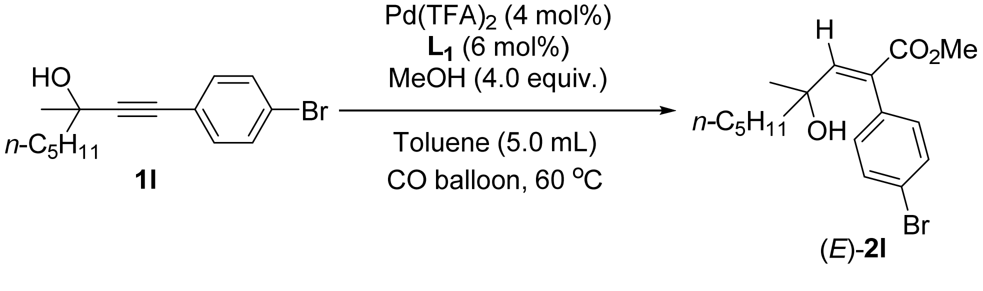

To a flame-dried Schlenk tube were added $Pd(TFA)_2$ (13.6 mg, 0.04 mmol), $\mathbf{L_1}$ (32.8 mg, 0.06 mmol), **1l** (295.3 mg, 1 mmol)/toluene (2 mL), MeOH (129.4 mg, 4.0 mmol)/toluene (2 mL), and diphenylmethane (the internal standard, 83.6 μL, d = 1.006 g/mL, 84.1 mg, 0.5 mmol)/toluene (1 mL) sequentially under argon. The resulting mixture was then frozen with a liquid nitrogen bath, degassed to remove the argon inside completely, and refilled with CO by a balloon of CO for three times. Then the resulting mixture was stirred at 60 °C with a balloon of CO and monitored at different time (see Table S8). The yields were determined by ^{1}H NMR measurement

using diphenylmethane as the internal standard.

Table S8. For **1l**:

Time / h	Yield of (*E*)-**2l**
2	35
4	46
6	60
8	67
10	74
12	80

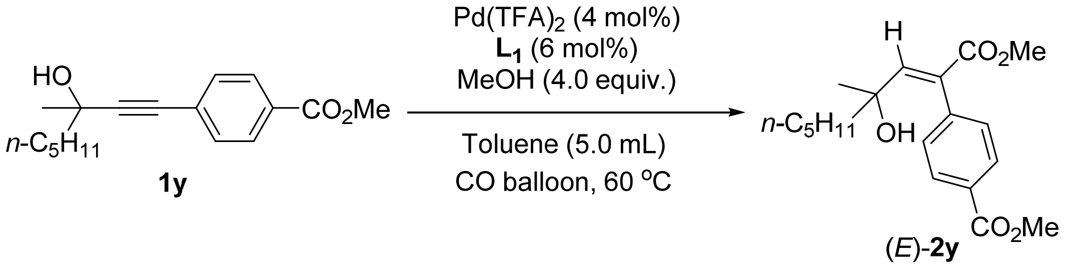

To a flame-dried Schlenk tube were added Pd(TFA)$_2$ (13.6 mg, 0.04 mmol), **L$_1$** (32.8 mg, 0.06 mmol), **1y** (274.4 mg, 1 mmol)/toluene (2 mL), MeOH (128.9 mg, 4.0 mmol)/toluene (2 mL), and diphenylmethane (the internal standard, 83.6 μL, d = 1.006 g/mL, 84.1 mg, 0.5 mmol)/toluene (1 mL) sequentially under argon. The resulting mixture was then frozen with a liquid nitrogen bath, degassed to remove the argon inside completely, and refilled with CO by a balloon of CO for three times. Then the resulting mixture was stirred at 60 ^{o}C with a balloon of CO and monitored at different time (see Table S9). The yields were determined by ^{1}H NMR measurement using diphenylmethane as the internal standard.

Table S9. For **1y**:

Time / h	Yield of (*E*)-**2y**
1	13
2	18
4	26
6	41
8	48
10	57
12	64

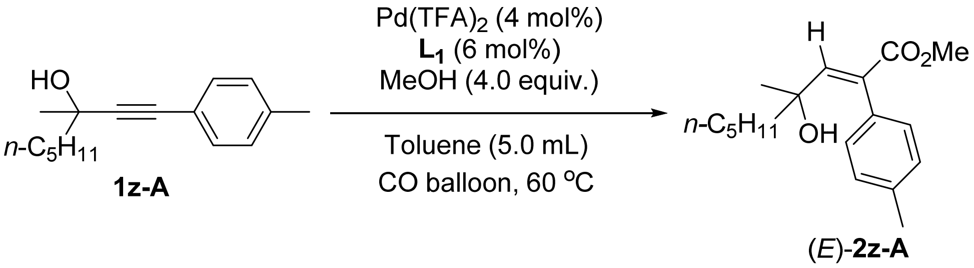

To a flame-dried Schlenk tube were added Pd(TFA)$_2$ (13.6 mg, 0.04 mmol), **L$_1$** (32.8 mg, 0.06 mmol), **1z-A** (230.4 mg, 1 mmol)/toluene (2 mL), MeOH (129.1 mg, 4.0 mmol)/toluene (2 mL), and diphenylmethane (the internal standard, 83.6 μL, d = 1.006 g/mL, 84.1 mg, 0.5 mmol)/toluene (1 mL) sequentially under argon. The resulting mixture was then frozen with a liquid nitrogen bath, degassed to remove the argon inside completely, and refilled with CO by a balloon of CO for three times. Then the resulting mixture was stirred at 60 ^{o}C with a balloon of CO and monitored at different time (see Table S10). The yields were determined by ^{1}H NMR measurement using diphenylmethane as the internal standard.

Table S10. For **1z-A**:

Time / h	Yield of (*E*)-**2z-A**
1	48
2	63
4	78
6	86
8	92
10	95

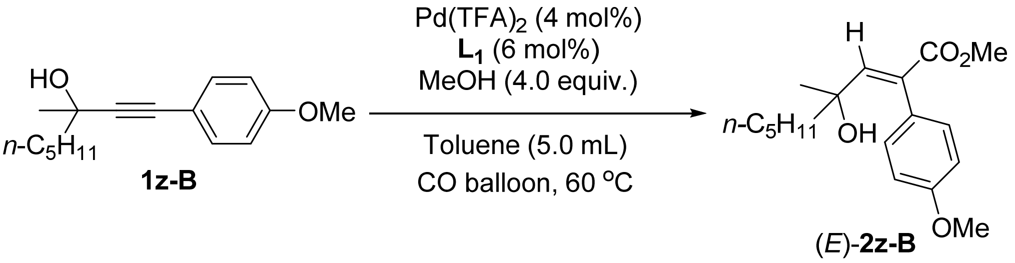

To a flame-dried Schlenk tube were added Pd(TFA)$_2$ (13.6 mg, 0.04 mmol), **L$_1$** (32.8 mg, 0.06 mmol), **1z-B** (246.4 mg, 1 mmol)/toluene (2 mL), MeOH (128.9 mg, 4.0 mmol)/toluene (2 mL), and diphenylmethane (the internal standard, 83.6 μL, d = 1.006 g/mL, 84.1 mg, 0.5 mmol)/toluene (1 mL) sequentially under argon. The resulting mixture was then frozen with a liquid nitrogen bath, degassed to remove the

argon inside completely, and refilled with CO by a balloon of CO for three times. Then the resulting mixture was stirred at 60 °C with a balloon of CO and monitored at different time (see Table S11). The yields were determined by ^{1}H NMR measurement using diphenylmethane as the internal standard.

Table S11. For **1z-B**:

Time / h	Yield of (*E*)-**2z-B**
0.5	56
1	64
2	75
4	90
6	95
8	98

Combing the above data, a linear relationship was obtained for ln $\{c_0/(c_0$-[(*E*)-**2k**-R])} vs. reaction time, indicating that the electronic effect has a significant effect on the rate-determining step (Figure S7).

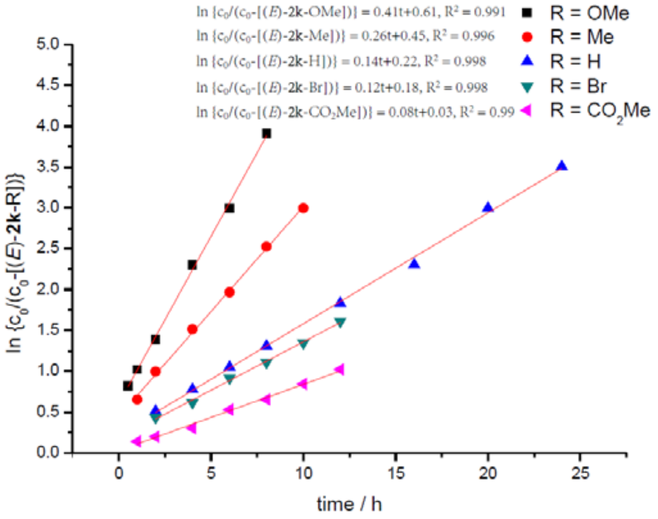

Figure S7. ln $\{c_0/(c_0$-[(*E*)-**2k**-R])} vs. time (R-square is the coefficient of determination)

(17) Preparation of 4-methoxycarbonylphenyl (*E*)-4-hydroxy-4-methyl-2-phenyl-non-2-enoate ((*E*)-2ke) (hcf-3-31)

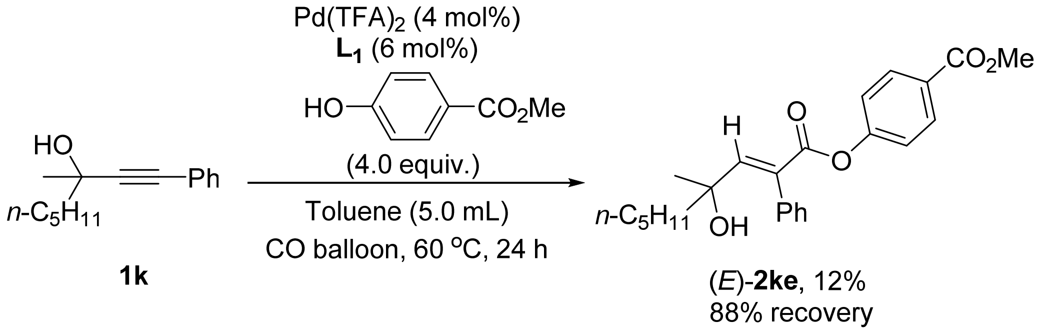

Following **Typical procedure IV**, the reaction of $Pd(TFA)_2$ (13.6 mg, 0.04 mmol), $\mathbf{L_1}$ (32.8 mg, 0.06 mmol), **1k** (216.3 mg, 1 mmol)/toluene (4 mL), and 4-methoxycarbonylphenol (608.9 mg, 4 mmol)/toluene (1 mL) afforded (*E*)-**2ke** (46.7 mg, 12%) (88% of **1k** remained based on ^{1}H NMR analysis of the crude product) as a yellow oil [eluent: petroleum ether/ethyl acetate = 50/1(~200 mL), 20/1 (630 mL), 10/1 (550 mL), 5/1 (240 mL)]: **^{1}H NMR** (400 MHz, $CDCl_3$): δ = 8.04 (d, J = 8.4 Hz, 2 H, Ar-H), 7.46-7.27 (m, 6 H, Ar-H and =CH), 7.16 (d, J = 8.8 Hz, 2 H, Ar-H), 3.89 (s, 3 H, CO_2Me), 1.61-1.50 (m, 2 H, CH_2), 1.50-1.18 (m, 10 H, 3 x CH_2 and CH_3 and OH), 0.90 (t, J = 7.0 Hz, 3 H, CH_3); **^{13}C NMR** (100 MHz, $CDCl_3$): δ = 166.3, 165.4, 154.6, 151.9, 134.4, 131.0, 130.8, 129.5, 128.3, 128.2, 127.6, 121.5, 74.4, 52.1, 43.1, 32.1, 28.9, 23.6, 22.5, 14.0; **MS** (ESI) m/z: 419 $(M+Na)^+$, 414 $(M+NH_4)^+$, 379 $(M-OH)^+$; **IR** (neat): v = 3516, 2959, 2940, 2862, 1717, 1436, 1278, 1186, 1160 cm^{-1}; **HRMS** calcd for $C_{24}H_{28}O_5Na$ $[M+Na]^+$: 419.1829, found: 419.1820.

(18) Preparation of *p*-tolyl (*E*)-4-hydroxy-4-methyl-2-phenylnon-2-enoate ((*E*)-2kf) (hcf-3-101, hcf-3-38)

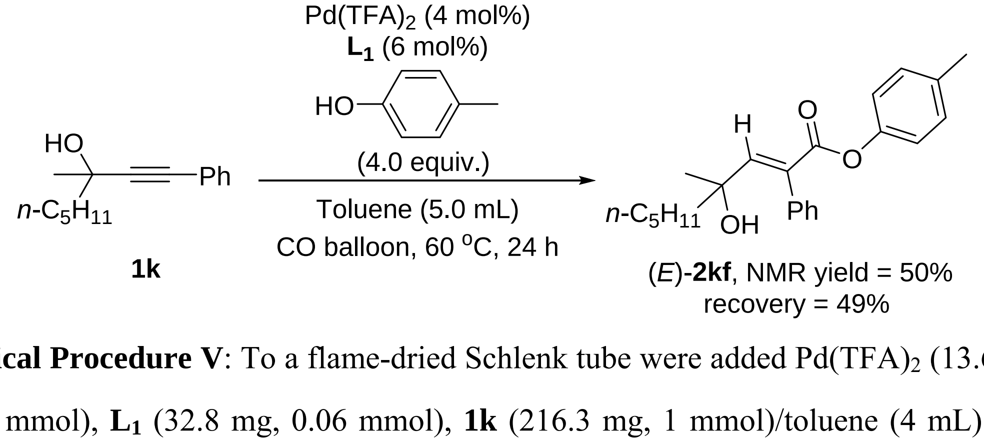

Typical Procedure V: To a flame-dried Schlenk tube were added $Pd(TFA)_2$ (13.6 mg, 0.04 mmol), $\mathbf{L_1}$ (32.8 mg, 0.06 mmol), **1k** (216.3 mg, 1 mmol)/toluene (4 mL), and *p*-cresol (432.8 mg, 4.0 mmol)/toluene (1 mL) sequentially under argon. The resulting

mixture was then frozen with a liquid nitrogen bath, degassed to remove the argon inside completely, and refilled with CO by a balloon of CO for three times. Then the resulting mixture was stirred at 60 °C with a balloon of CO for 24 h. After that, the resulting mixture was diluted with 3 mL of ethyl acetate, filtered through a short column of silica gel (3 cm) eluted with ethyl acetate (20 x 2 mL), and concentrated. The crude product was purified by column chromatography on silica gel to afford impure mixture (410.7 mg) [eluent: petroleum ether/ethyl acetate = 50/1 (~200 mL), 20/1 (1050 mL), 10/1 (440 mL)] as a solid containing (*E*)-**2kf** (162.1 mg, 0.46 mmol, 46%) and *p*-cresol (248.6 mg, 2.30 mmol) since the Rf values of *p*-cresol and product are almost the same [50% of (*E*)-**2kf** and 49% of **1k** were detected based on ^{1}H NMR analysis of the crude product]. The pure sample of (*E*)-**2kf** was prepared as a yellow oil by preparative thin layer chromatography (TLC, 20 x 20 cm^2) eluted with petroleum ether/ethyl acetate = 3/1: **^{1}H NMR** (400 MHz, $CDCl_3$): δ = 7.48-7.28 (m, 5 H, Ar-H), 7.25 (s, 1 H, =CH), 7.13 (d, *J* = 8.0 Hz, 2 H, Ar-H), 6.94 (d, *J* = 8.4 Hz, 2 H, Ar-H), 2.31 (s, 3 H, CH_3), 1.64-1.49 (m, 2 H, CH_2), 1.49-1.19 (m, 10 H, 3 x CH_2 and CH_3 and OH), 0.89 (t, *J* = 7.2 Hz, 3 H, CH_3); **^{13}C NMR** (100 MHz, $CDCl_3$): δ = 166.2, 151.0, 148.7, 135.3, 134.8, 131.2, 129.8, 129.6, 128.2, 128.0, 121.1, 74.4, 43.1, 32.1, 29.0, 23.6, 22.5, 20.8, 14.0; **MS** (EI 70 ev) *m/z* (%): 352 (M$^+$, 1.16), 43 (100); **IR** (neat): *v* = 3502, 2956, 2931, 2861, 1731, 1507, 1213, 1192, 1167 cm^{-1}; **HRMS** calcd for $C_{23}H_{28}O_3$ [M]$^+$: 352.2038, found: 352.2042.

(19) Preparation of 4-methoxyphenyl (*E*)-4-hydroxy-4-methyl-2-phenylnon-2-enoate ((*E*)-2kg) (hcf-3-102, hcf-3-52)

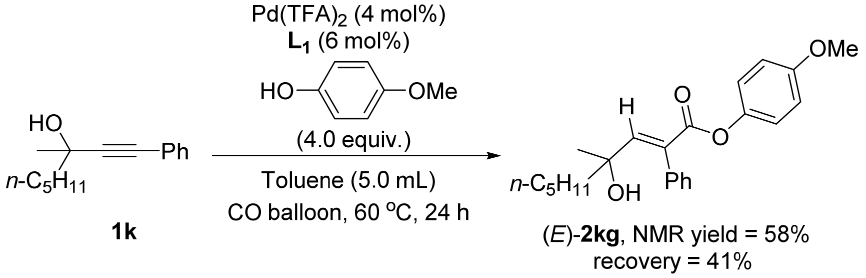

Following **Typical procedure V**, the reaction of Pd(TFA)$_2$ (13.6 mg, 0.04 mmol), **L$_1$** (32.8 mg, 0.06 mmol), **1k** (216.3 mg, 1 mmol)/toluene (3 mL), and 4-methoxyphenol (496.9 mg, 4 mmol)/toluene (2 mL) afforded a solid (574.5 mg)

[eluent: petroleum ether/ethyl acetate = 50/1 (~200 mL), 20/1 (1050 mL), 10/1 (440 mL)] containing (*E*)-**2kg** (206.2 mg, 0.56 mmol, 56%) and 4-methoxyphenol (368.3mg, 2.97 mmol) since the Rf values of 4-methoxyphenol and product are almost the same [58% of (*E*)-**2kg** and 41% of **1k** were detected based on ^{1}H NMR analysis of the crude product]. The pure sample of (*E*)-**2kg** was prepared as a yellow oil by preparative thin layer chromatography (TLC, 20 x 20 cm^2) eluted with petroleum ether/ethyl acetate = 3/1: **^{1}H NMR** (400 MHz, $CDCl_3$): δ = 7.51-7.28 (m, 5 H, Ar-H), 7.25 (s, 1 H, =CH), 6.98 (d, *J* = 8.8 Hz, 2 H, Ar-H), 6.84 (d, *J* = 8.8 Hz, 2 H, Ar-H), 3.76 (s, 3 H, OMe), 1.66-1.49 (m, 2 H, CH_2), 1.49-1.19 (m, 10 H, 3 x CH_2 and CH_3 and OH), 0.89 (t, *J* = 7.0 Hz, 3 H, CH_3); **^{13}C NMR** (100 MHz, $CDCl_3$): δ = 166.3, 157.1, 151.0, 144.5, 134.8, 131.2, 129.5, 128.2, 128.0, 122.2, 114.3, 74.4, 55.5, 43.1, 32.1, 28.9, 23.6, 22.5, 14.0; **MS** (EI 70 ev) *m/z* (%): 368 (M^+, 6.06), 43 (100); **IR** (neat): *v* = 3487, 2948, 2937, 2869, 2844, 1724, 1509, 1238, 1194, 1172, 1158 cm^{-1}; **HRMS** calcd for $C_{23}H_{28}O_4$ $[M]^+$: 368.1988, found: 368.1993.

(20) Preparation of 4-chlorophenyl (*E*)-4-hydroxy-4-methyl-2-phenylnon-2-enoate ((*E*)-2kh) (hcf-3-103, hcf-3-53)

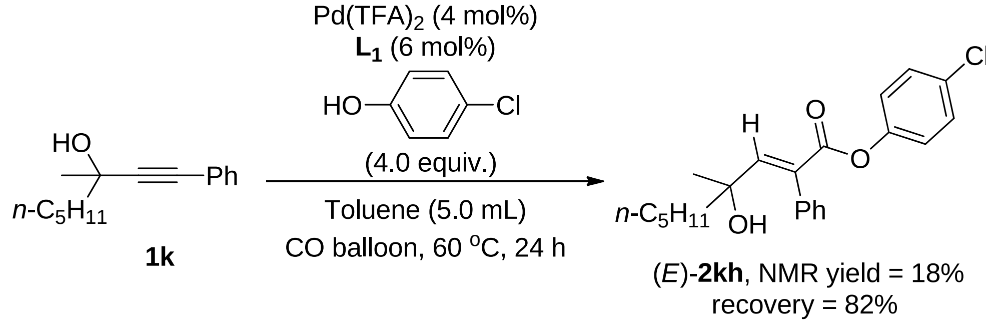

Following **Typical procedure V**, the reaction of $Pd(TFA)_2$ (13.6 mg, 0.04 mmol), **L_1** (32.8 mg, 0.06 mmol), **1k** (216.4 mg, 1 mmol)/toluene (3 mL), and 4-chlorophenol (514.5 mg, 4 mmol)/toluene (2 mL) afforded a solid (189.3 mg) [eluent: petroleum ether/ethyl acetate = 50/1 (~200 mL), 20/1 (1050 mL), 10/1 (440 mL)] containing (*E*)-**2kh** (63.4 mg, 0.17 mmol, 17%) and 4-chlorophenol (125.9mg, 0.98 mmol) since the Rf values of 4-chlorophenol and product are almost the same [18% of (*E*)-**2kh** and 82% of **1k** were detected based on ^{1}H NMR analysis of the crude product]. The pure sample of (*E*)-**2kh** was prepared as a yellow oil by preparative thin layer

chromatography (TLC, 20 x 20 cm^2) eluted with petroleum ether/ethyl acetate = 3/1: **^{1}H NMR** (400 MHz, $CDCl_3$): δ = 7.47-7.22 (m, 8 H, Ar-H and =CH), 7.02 (d, J = 8.8 Hz, 2 H, Ar-H), 1.64-1.49 (m, 2 H, CH_2), 1.49-1.19 (m, 10 H, 3 x CH_2 and CH_3 and OH), 0.89 (t, J = 7.2 Hz, 3 H, CH_3); **^{13}C NMR** (100 MHz, $CDCl_3$): δ = 165.7, 151.7, 149.4, 134.5, 131.1, 130.9, 129.5, 129.3, 128.3, 128.2, 122.9, 74.4, 43.1, 32.1, 28.9, 23.6, 22.5, 14.0; **MS** (ESI) m/z: 397 $(M(^{37}Cl)+Na)^+$, 395 $(M(^{35}Cl)+Na)^+$, 357 $(M(^{37}Cl)-OH)^+$, 355 $(M(^{35}Cl)-OH)^+$; **IR** (neat): v = 3437, 2958, 2931, 2871, 1732, 1486, 1196, 1164, 1090, 1014 cm^{-1}; **HRMS** calcd for $C_{22}H_{25}{}^{35}ClO_3Na$ $[M+Na]^+$: 395.1384, found: 395.1377.

The negative value for ρ points out the reaction favors phenols with electron-donating groups (Table 12, Figure S8).

Table 12. Hammett study with phenols bearing various substituents

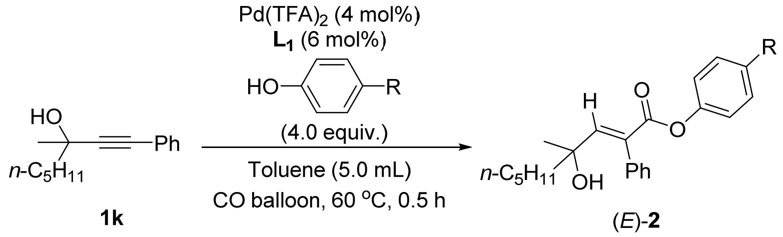

entry	R	Yield/%
1	H	11
2	MeO_2C	3.5
3	Me	14
4	MeO	16
5	Cl	6

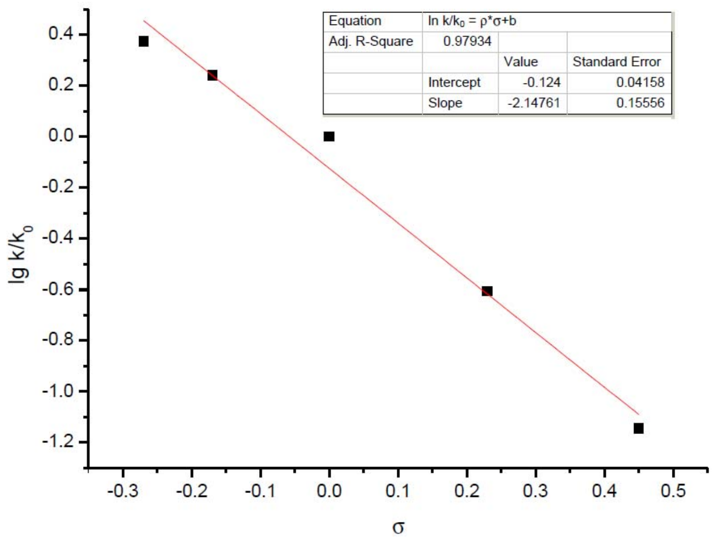

Figure S8. Hammett equation of phenols with varying acidity

References:

1. W. Zhang, C. Huang, Y. Yuan, S. Ma, *Chem. Commun.* **2017**, *53*, 12430.

2. Xu, D.; Li, Z.; Ma, S. *Tetrahedron Lett*. **2003**, *44*, 6343.

3. Kim N.; Widenhoefer, R. A. *Angew. Chem., Int. Ed.* **2018**, *57*, 4722.

4. Dai, J.; Duan, X.; Zhou, J.; Fu, C.; Ma, S. *Chin. J. Chem*. **2018**, *36*, 387.

5. Eleya, N.; Mahal, A.; Hein, M.; Villinger, A.; Langer, P. *Adv. Synth. Catal.* **2011**, *353*, 2761.

6. Tang, X.; Huang, X.; Cao, T.; Han, Y.; Jiang, X.; Lin, W.; Tang, Y.; Zhang, J.; Yu, Q.; Fu, C.; Ma, S. *Org. Chem. Front.* **2015**, *2*, 688.

7. Rajaram, S.; Ramulu, U.; Aravind, S. Babu, K. S. *Helv. Chim. Acta*. **2015**, *98*, 650.

8. Grée, D.; Vallerie, L.; Grée, R. *J. Org. Chem.* **2001**, *66*, 2374.

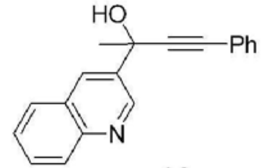

1d

hcf-1-185-H
Sep 1 2017
SOLVENT: cdcl3
NA = 8
F1 = 399.749542 MHz
F2 = 100.526031 MHz

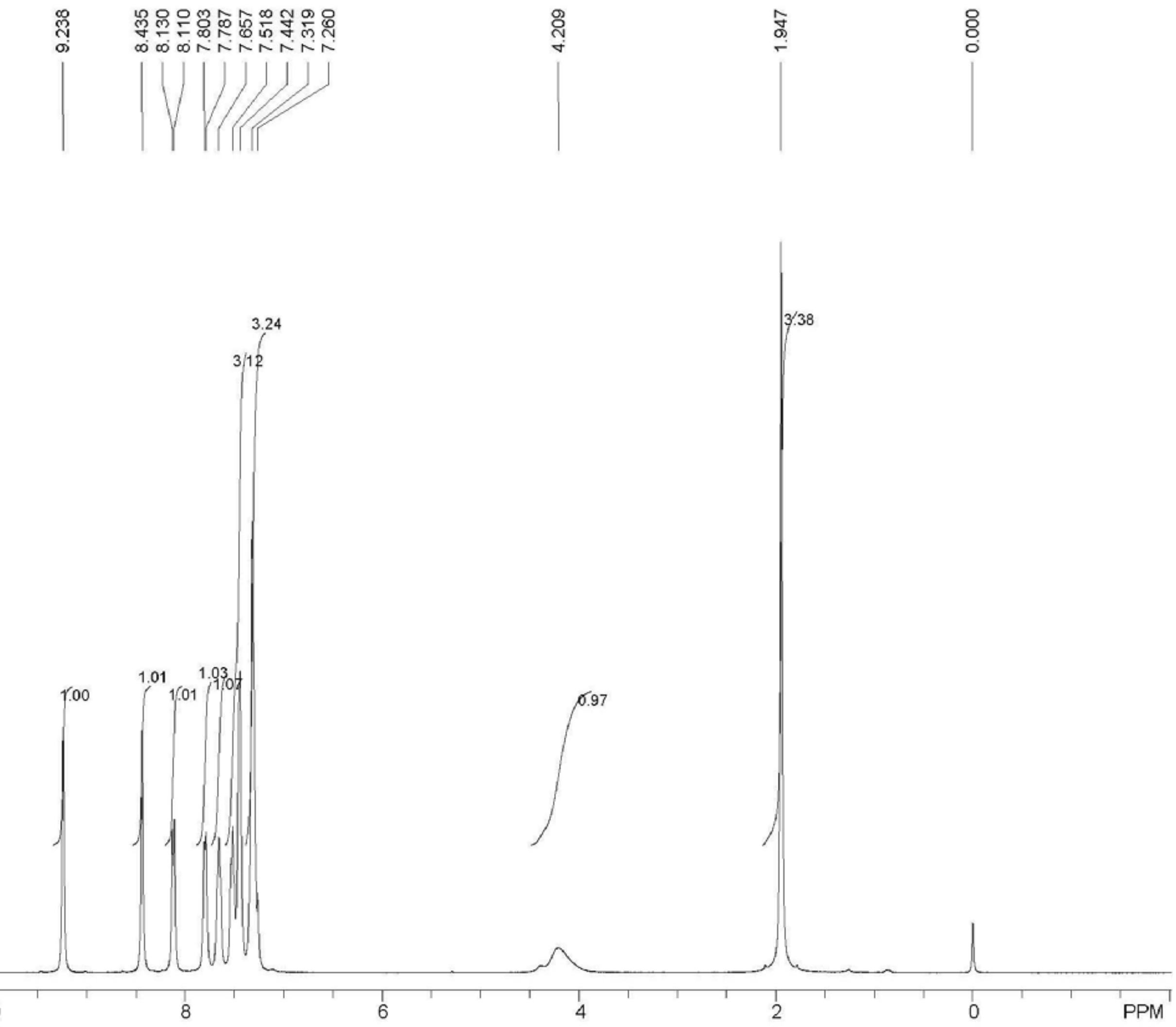

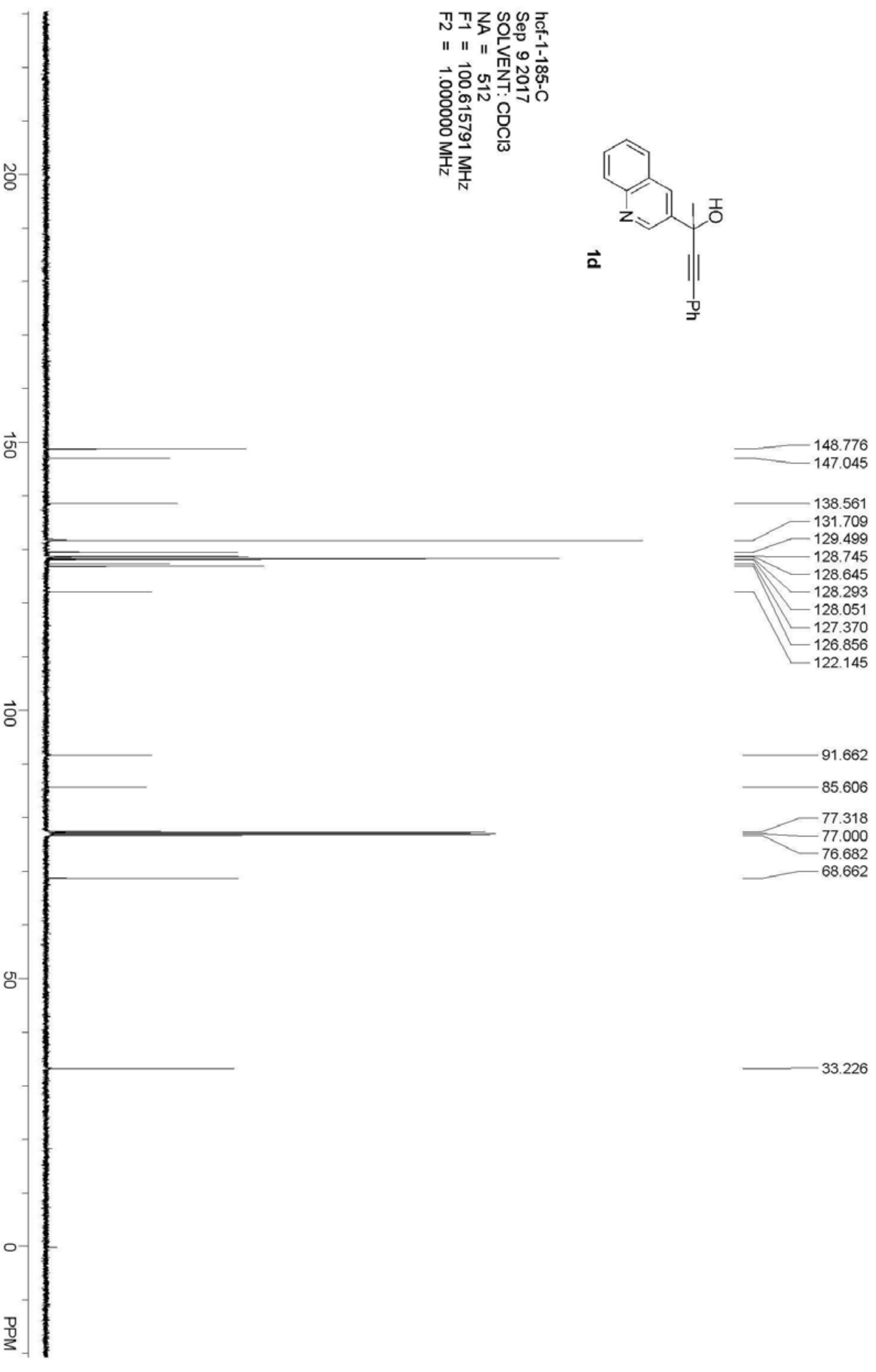
hcf-1-185-C
Sep 9 2017
SOLVENT: CDCl3
NA = 512
F1 = 100.615791 MHz
F2 = 1.000000 MHz
HO
N
Ph
1d
148.776
147.045
138.561
131.709
129.499
128.745
128.645
128.293
128.051
127.370
126.856
122.145
91.662
85.606
77.318
77.000
76.682
68.662
33.226
200
150
100
50
0
PPM

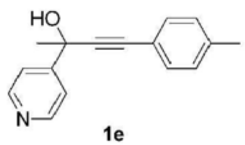

1e

hcf-1-146-H
Mar 16 2017
SOLVENT: cdcl3
NA = 8
F1 = 399.749542 MHz
F2 = 100.526031 MHz

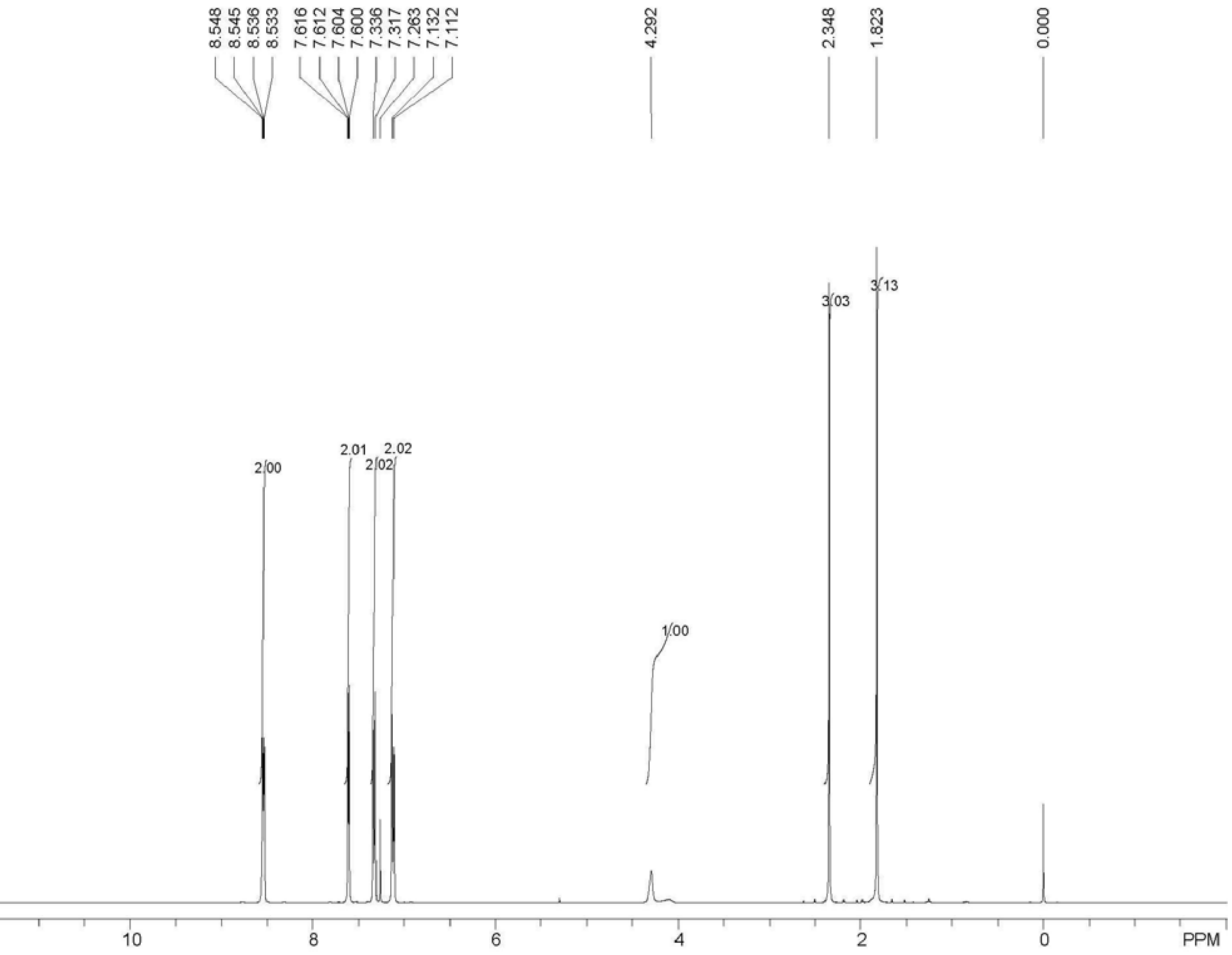

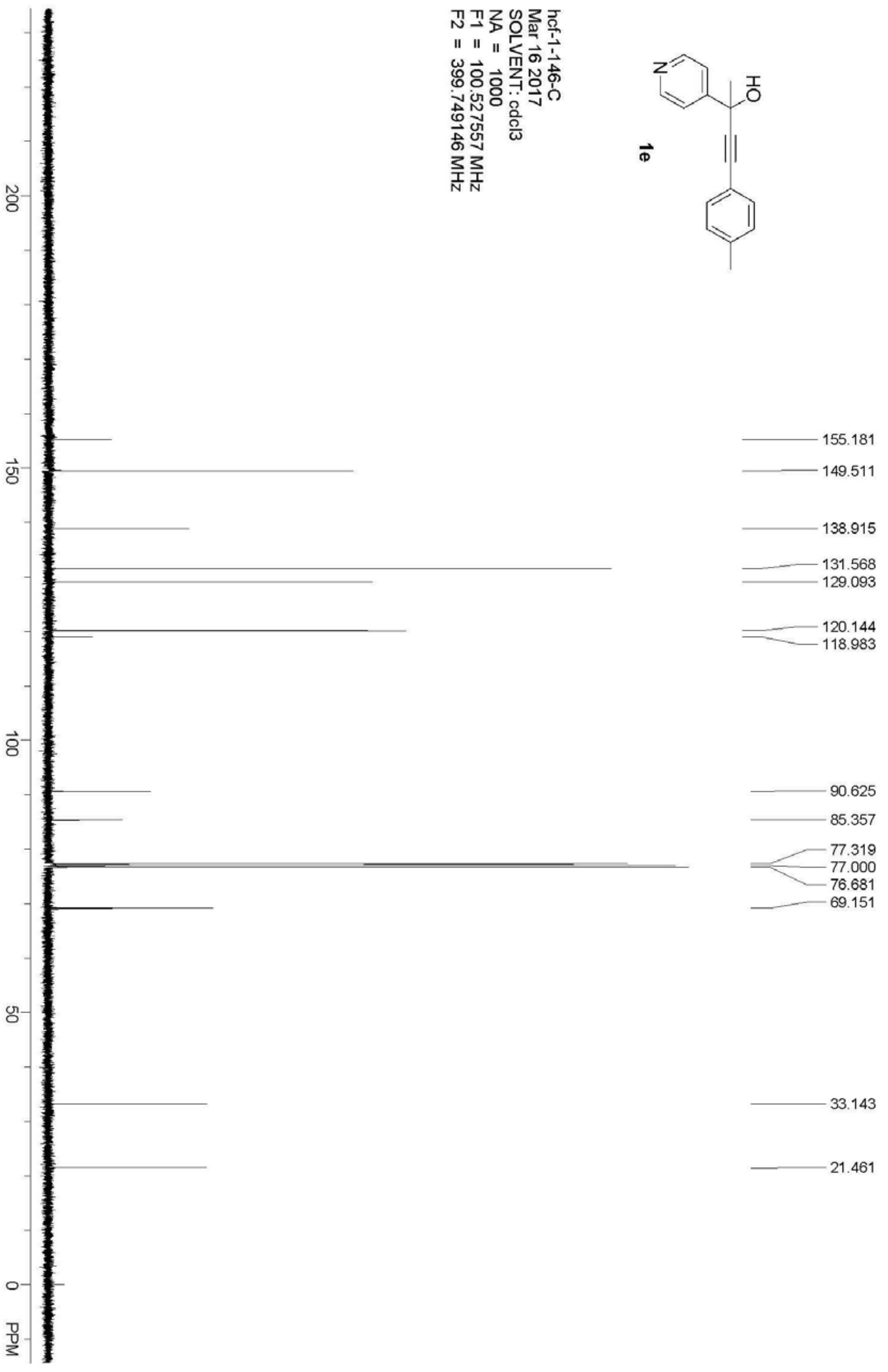

HO
N
1e
hcf-1-146-C
Mar 16 2017
SOLVENT: cdcl3
NA = 1000
F1 = 100.527557 MHz
F2 = 399.749146 MHz
155.181
149.511
138.915
131.568
129.093
120.144
118.983
90.625
85.357
77.319
77.000
76.681
69.151
33.143
21.461
200
150
100
50
0
PPM

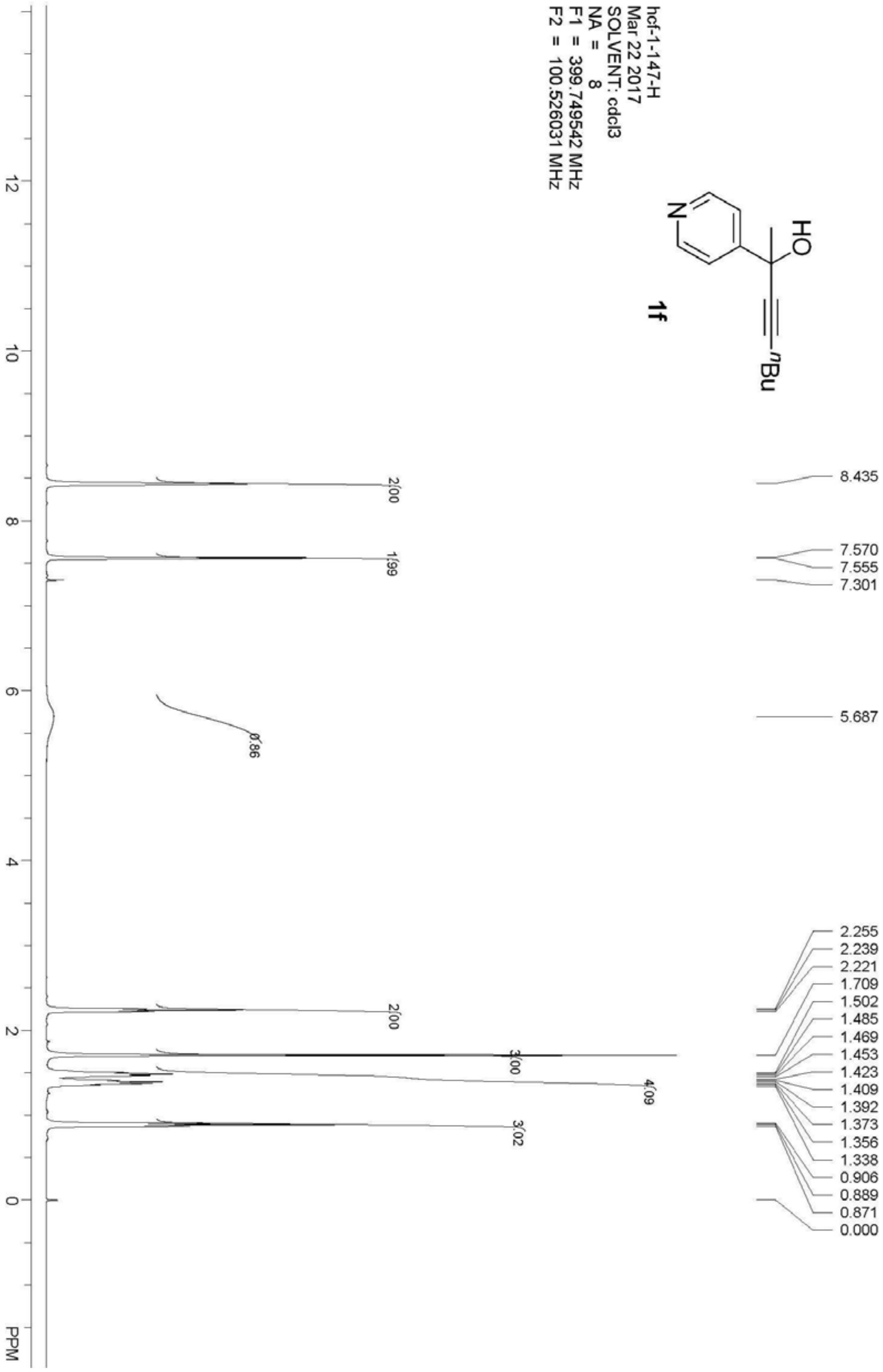
hcf-1-147-H
Mar 22 2017
SOLVENT: cdcl3
NA = 8
F1 = 399.749542 MHz
F2 = 100.526031 MHz
HO
N
ⁿBu
1f
8.435
7.570
7.555
7.301
5.687
2.255
2.239
2.221
1.709
1.502
1.485
1.469
1.453
1.423
1.409
1.392
1.373
1.356
1.338
0.906
0.889
0.871
0.000
2.00
1.99
0.86
2.00
3.00
4.09
3.02
PPM

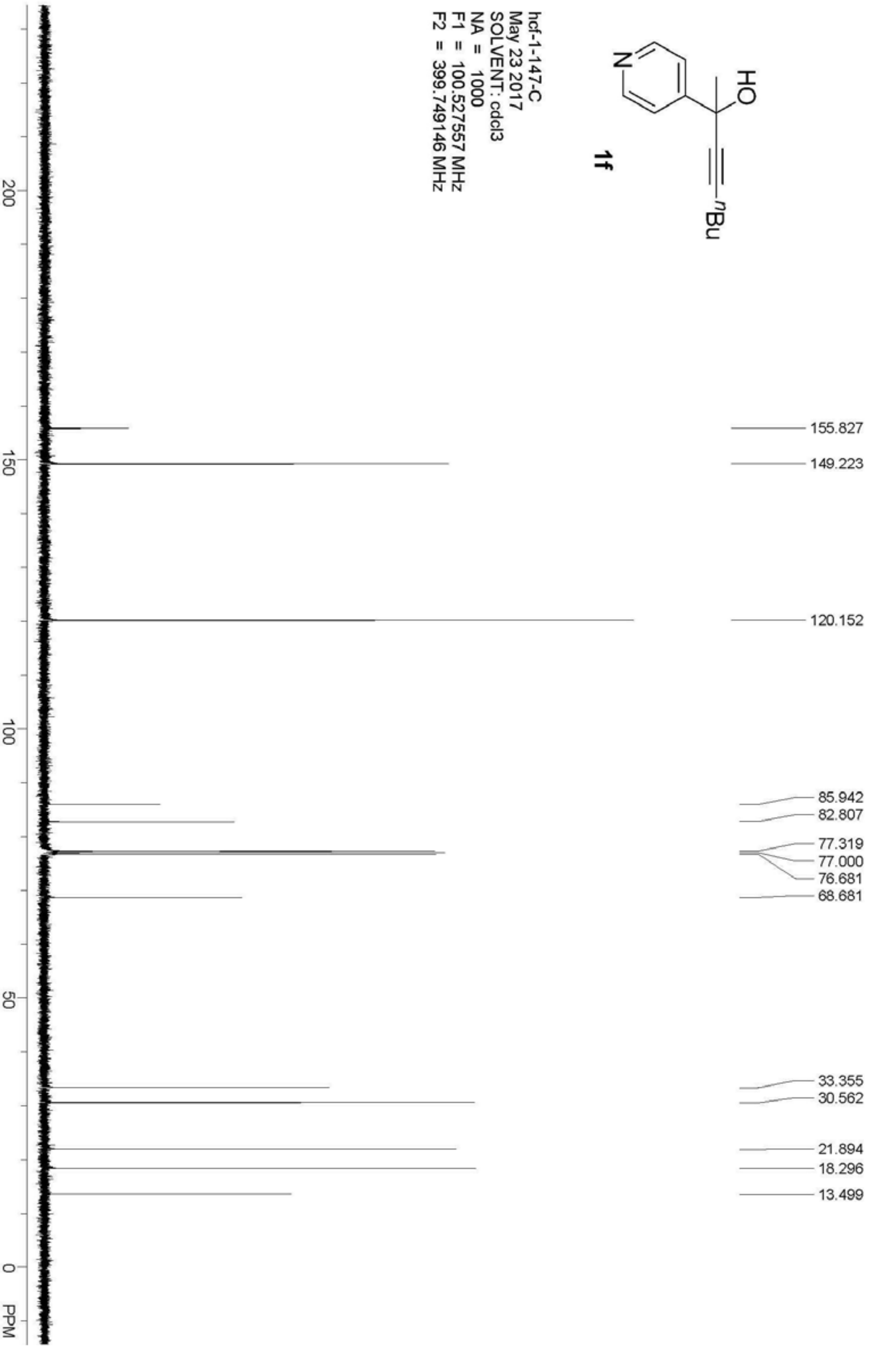
hcf-1-147-C
May 23 2017
SOLVENT: cdcl3
NA = 1000
F1 = 100.527557 MHz
F2 = 399.749146 MHz
1f
HO
N
nBu
155.827
149.223
120.152
85.942
82.807
77.319
77.000
76.681
68.681
33.355
30.562
21.894
18.296
13.499
200
150
100
50
0
PPM

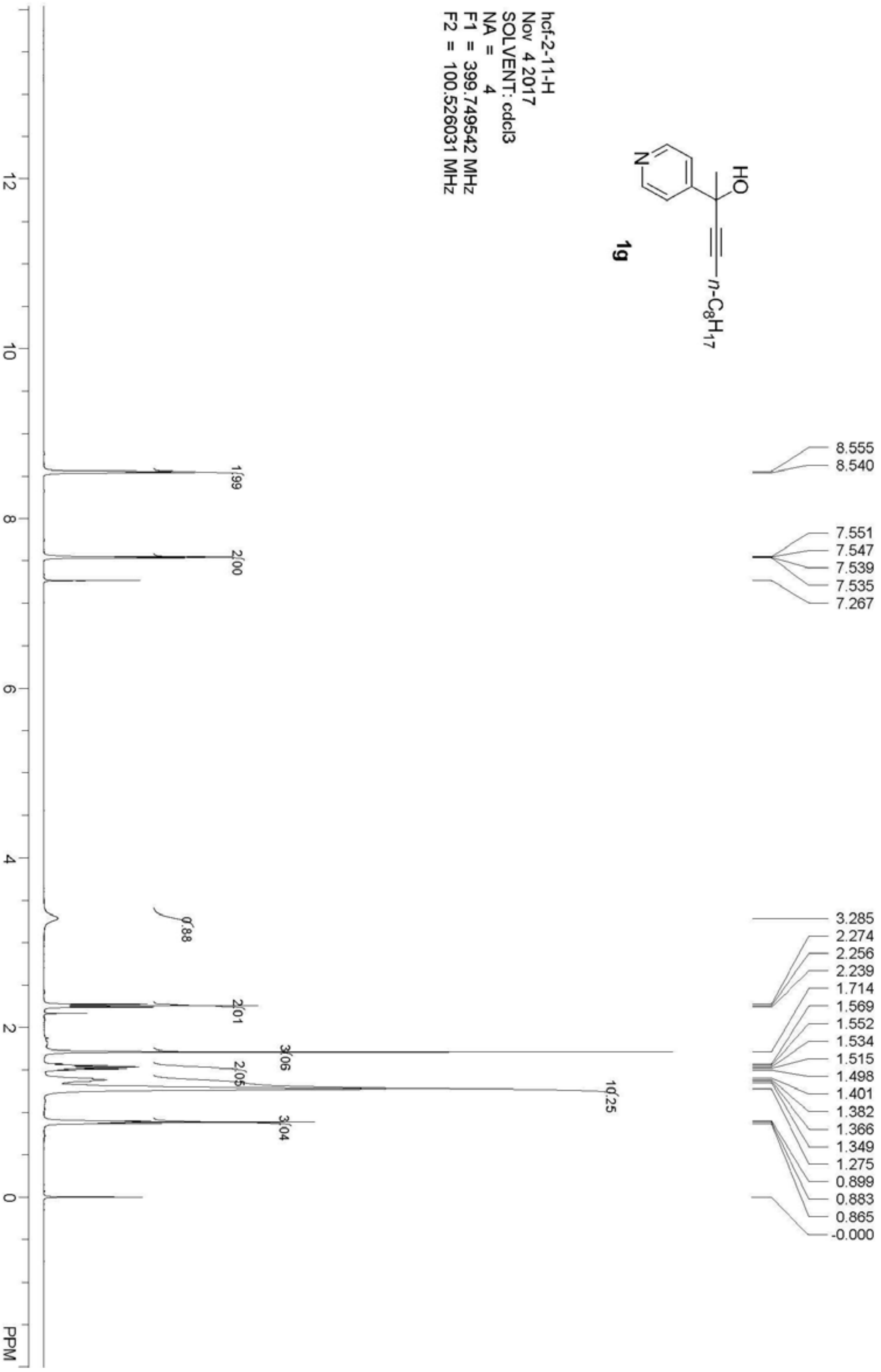

hcf-2-11-H
Nov 4 2017
SOLVENT: cdcl3
NA = 4
F1 = 399.749542 MHz
F2 = 100.526031 MHz
HO
N
n-C8H17
1g
8.555
8.540
7.551
7.547
7.539
7.535
7.267
3.285
2.274
2.256
2.239
1.714
1.569
1.552
1.534
1.515
1.498
1.401
1.382
1.366
1.349
1.275
0.899
0.883
0.865
-0.000
1.99
2.00
0.88
2.01
3.06
2.05
10.25
3.04
12
10
8
6
4
2
0
PPM

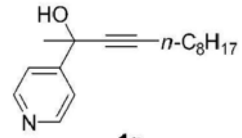

1g

hcf-2-11-C
Oct 1 2017
SOLVENT: CDCl3
NA = 512
F1 = 100.615791 MHz
F2 = 1.000000 MHz

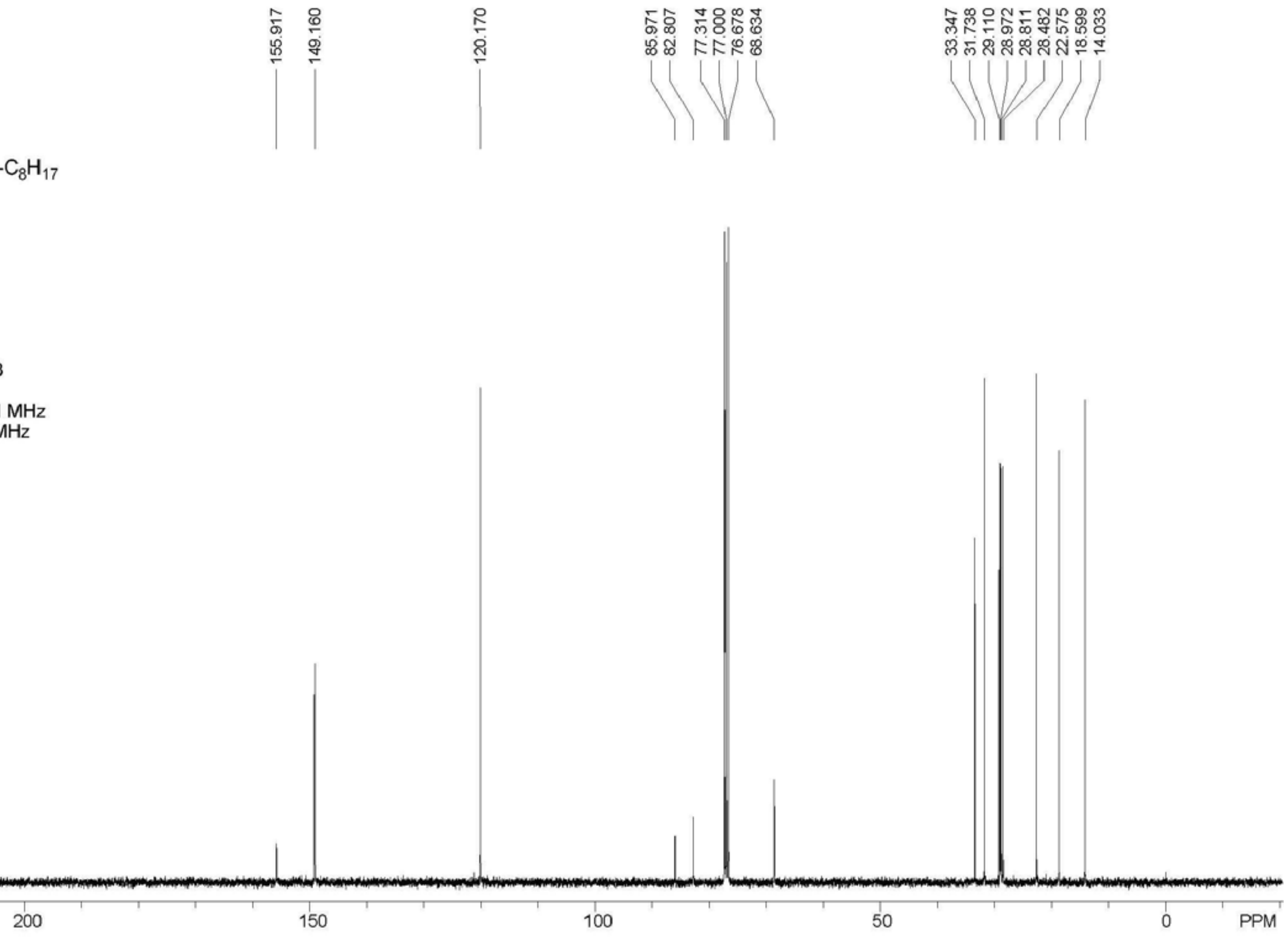

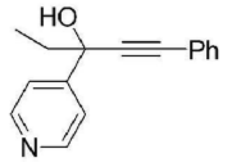
HO
Ph
N

1h

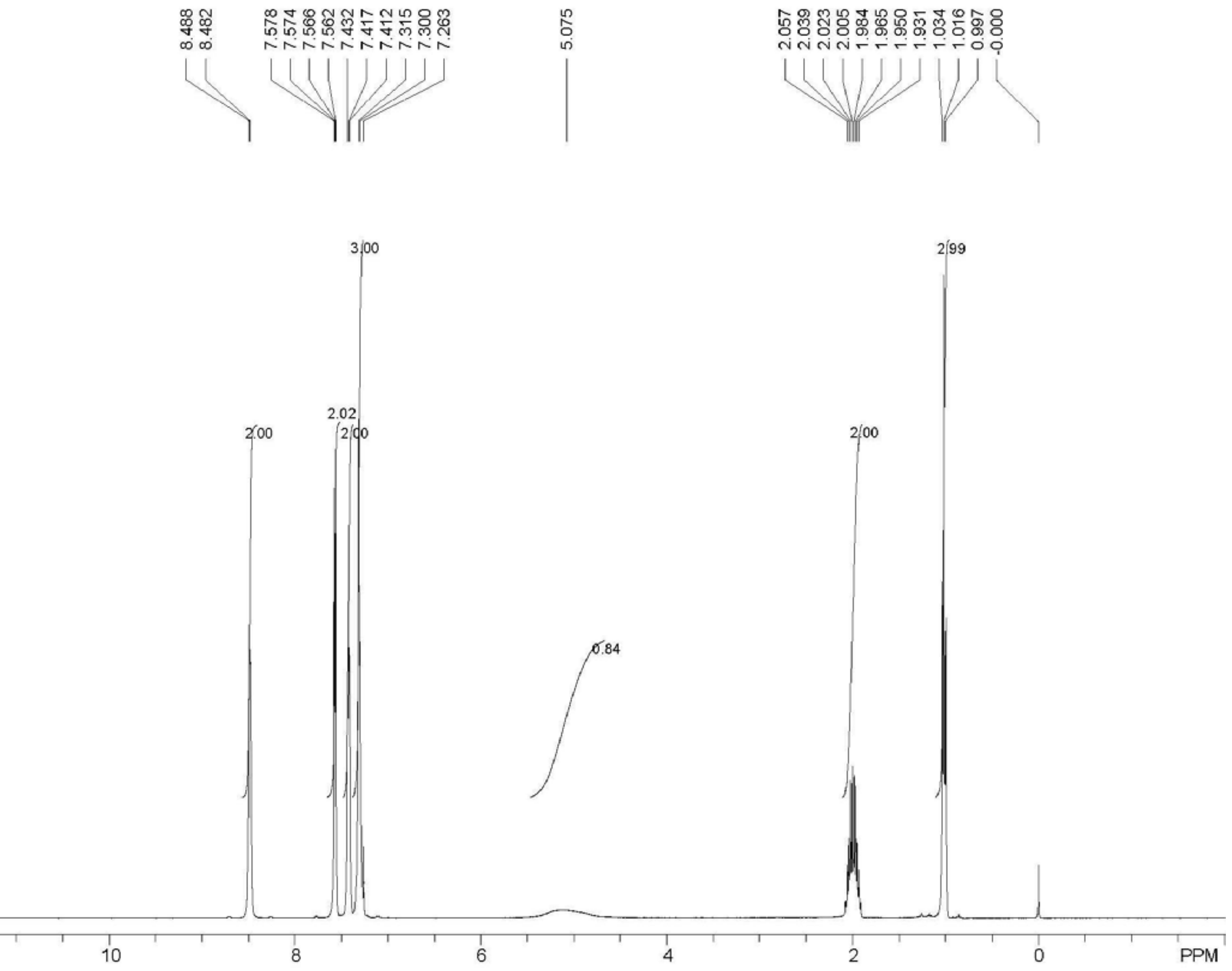
8.488
8.482
7.578
7.574
7.566
7.562
7.432
7.417
7.412
7.315
7.300
7.263
5.075
2.057
2.039
2.023
2.005
1.984
1.965
1.950
1.931
1.034
1.016
0.997
-0.000
hcf-1-164-H
May 31 2017
SOLVENT: cdcl3
NA = 4
F1 = 399.749542 MHz
F2 = 100.526031 MHz
2.00
2.02
2.00
3.00
0.84
2.00
2.99
12
10
8
6
4
2
0
PPM

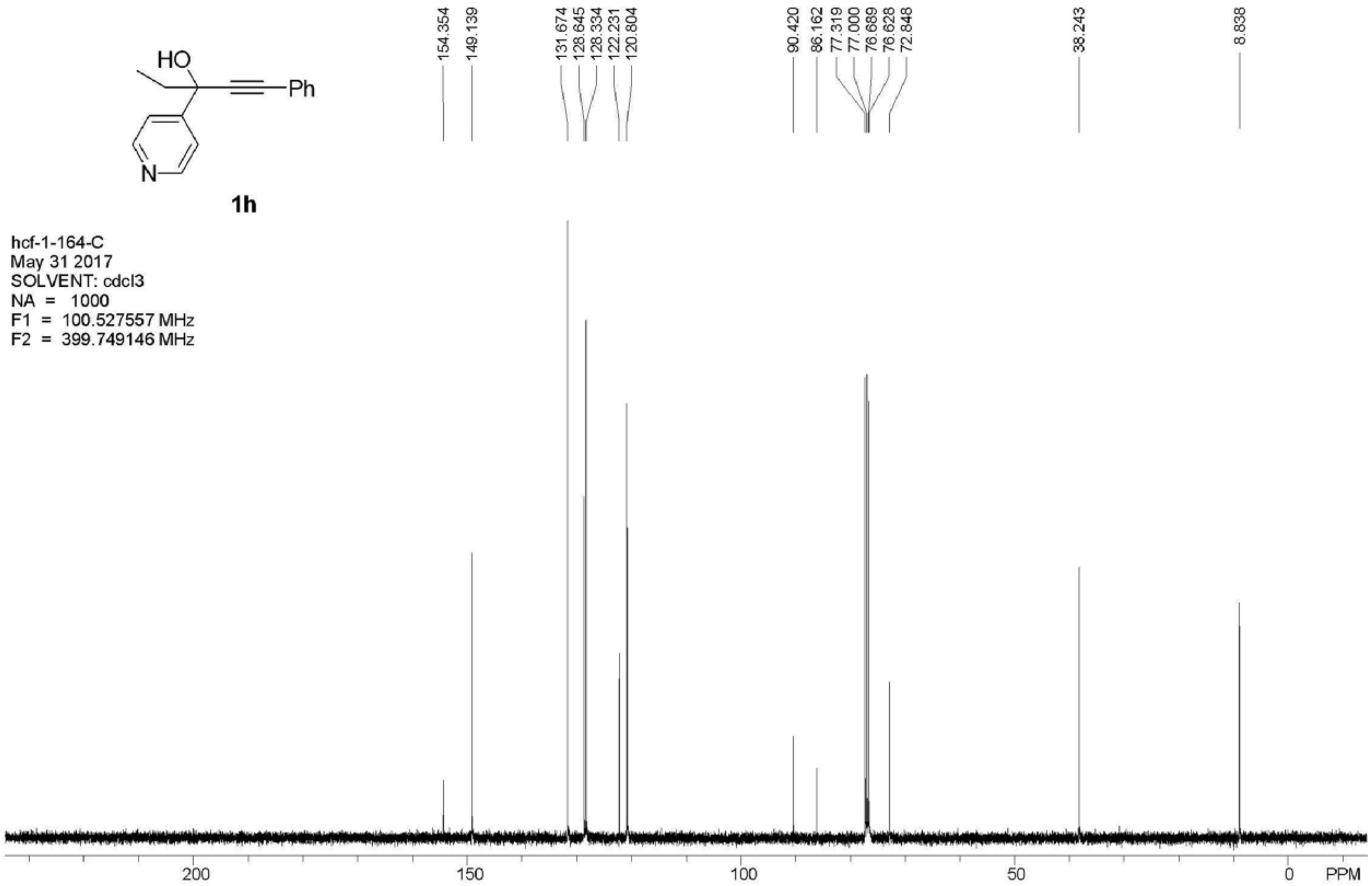
HO
Ph
N
1h
hcf-1-164-C
May 31 2017
SOLVENT: cdcl3
NA = 1000
F1 = 100.527557 MHz
F2 = 399.749146 MHz
154.354
149.139
131.674
128.645
128.334
122.231
120.804
90.420
86.162
77.319
77.000
76.689
76.628
72.848
38.243
8.838
200
150
100
50
0
PPM

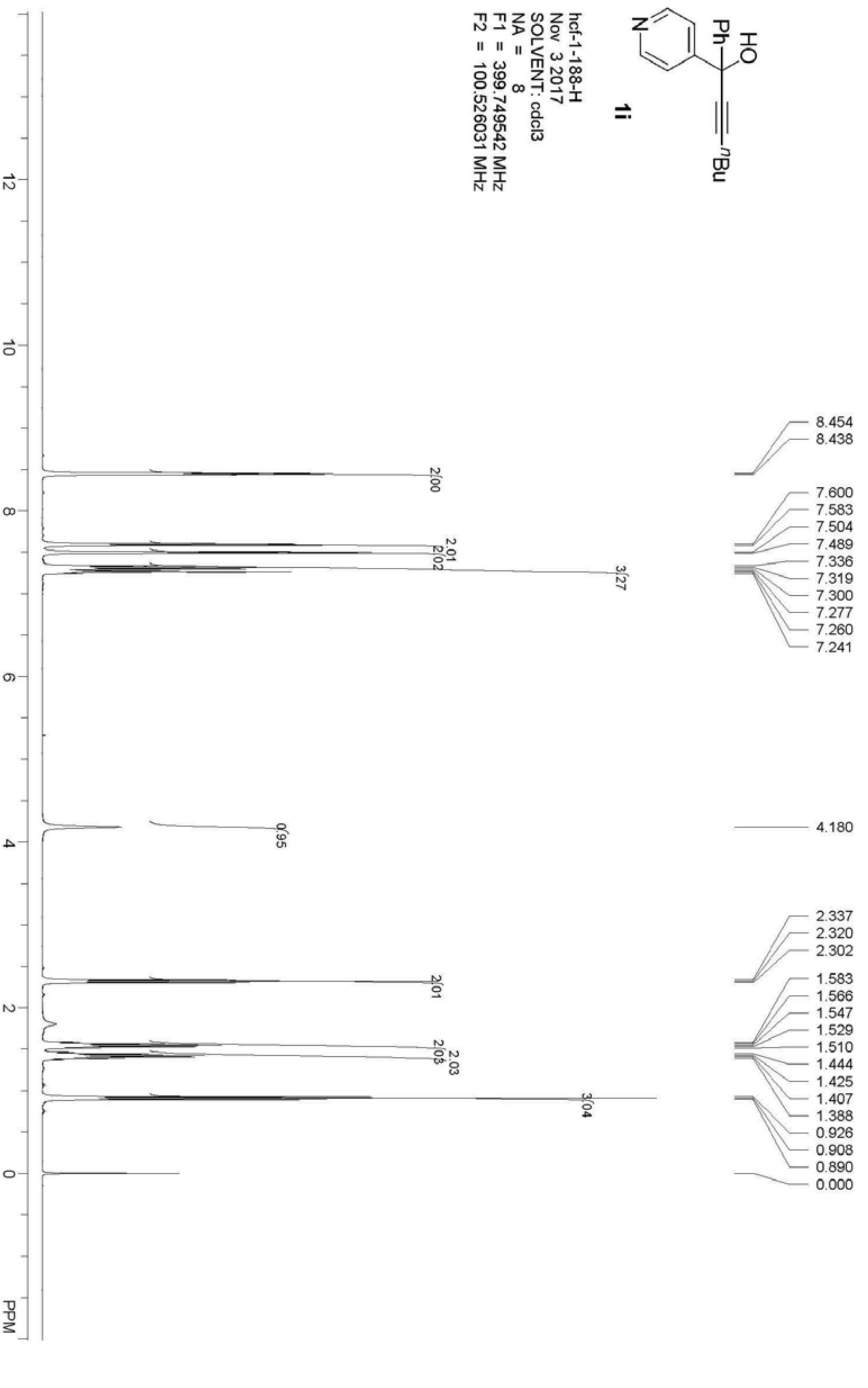
HO
Ph
nBu
N
1i
hcf-1-188-H
Nov 3 2017
SOLVENT: cdcl3
NA = 8
F1 = 399.749542 MHz
F2 = 100.526031 MHz
8.454
8.438
7.600
7.583
7.504
7.489
7.336
7.319
7.300
7.277
7.260
7.241
4.180
2.337
2.320
2.302
1.583
1.566
1.547
1.529
1.510
1.444
1.425
1.407
1.388
0.926
0.908
0.890
0.000
2.00
2.01
2.02
3.27
0.95
2.01
2.03
2.03
3.04
12
10
8
6
4
2
0
PPM

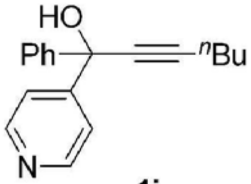

1i

hcf-1-188-C
Sep 1 2017
SOLVENT: cdcl3
NA = 1000
F1 = 100.527557 MHz
F2 = 399.749146 MHz

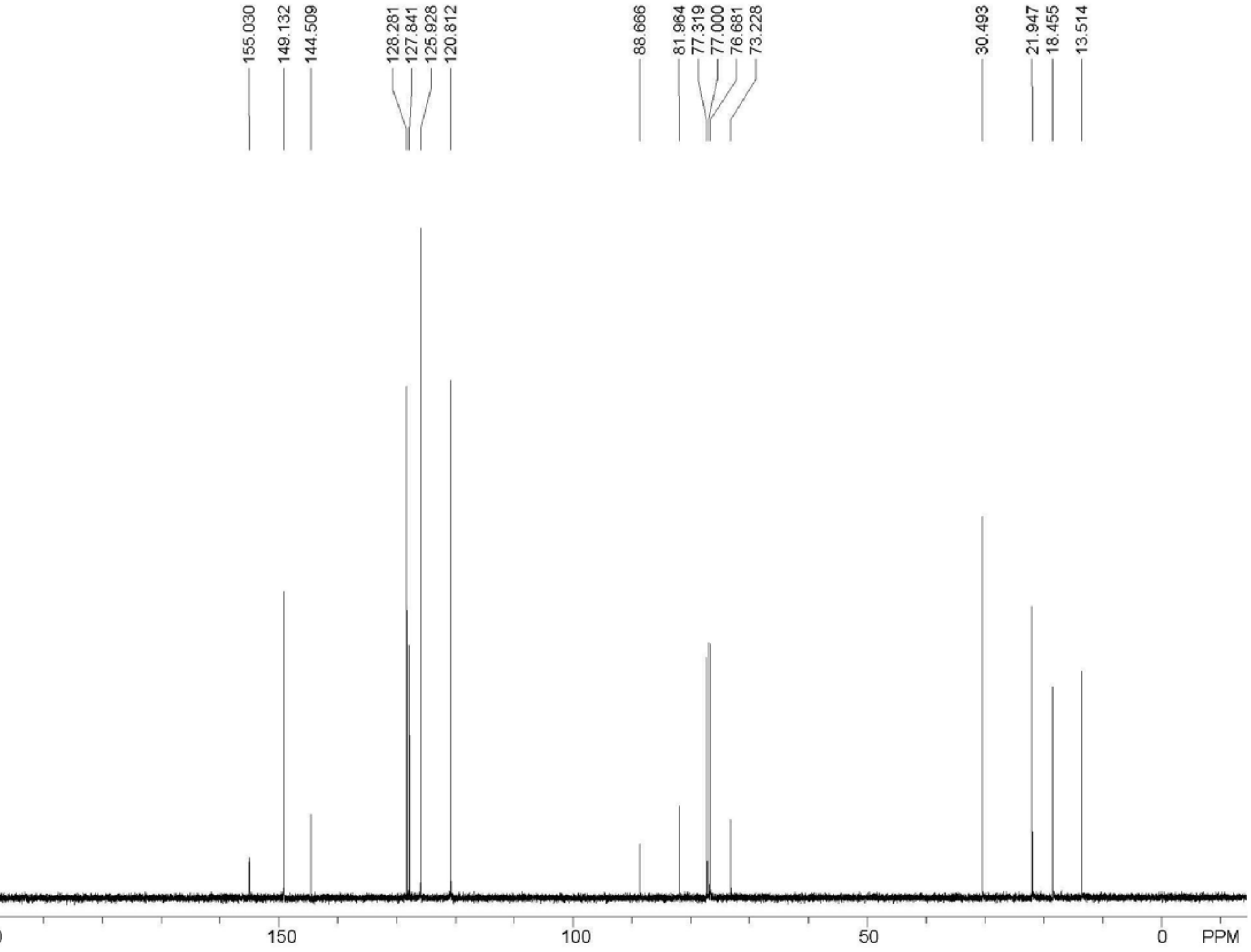

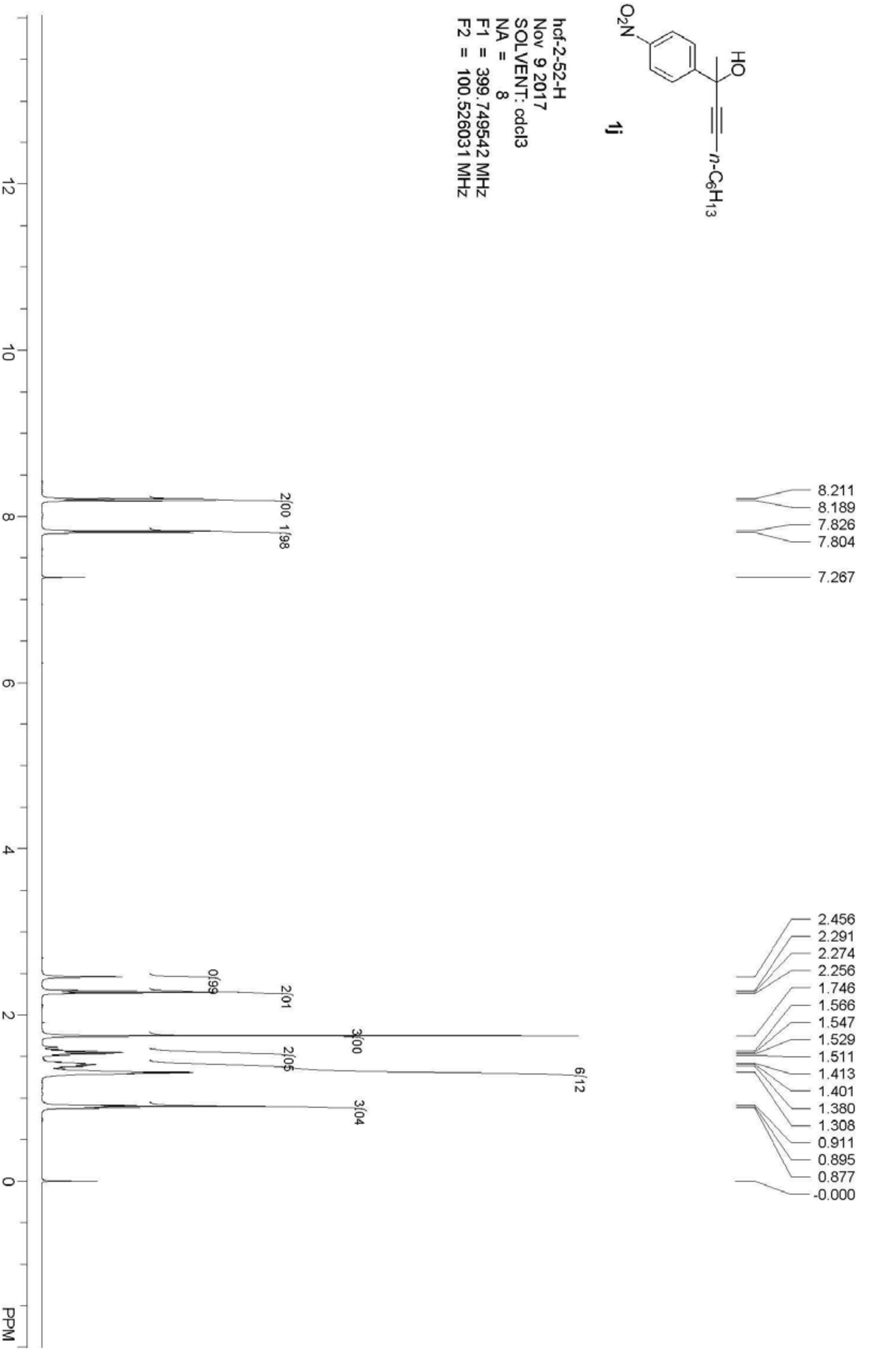

HO
O2N
n-C6H13
1j
hcf-2-52-H
Nov 9 2017
SOLVENT: cdcl3
NA = 8
F1 = 399.749542 MHz
F2 = 100.526031 MHz
8.211
8.189
7.826
7.804
7.267
2.456
2.291
2.274
2.256
1.746
1.566
1.547
1.529
1.511
1.413
1.401
1.380
1.308
0.911
0.895
0.877
-0.000
2.00
1.98
0.99
2.01
3.00
2.05
6.12
3.04
12
10
8
6
4
2
0
PPM

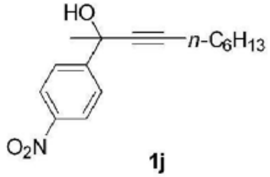

HO
n-C_6H_{13}
O_2N

1j

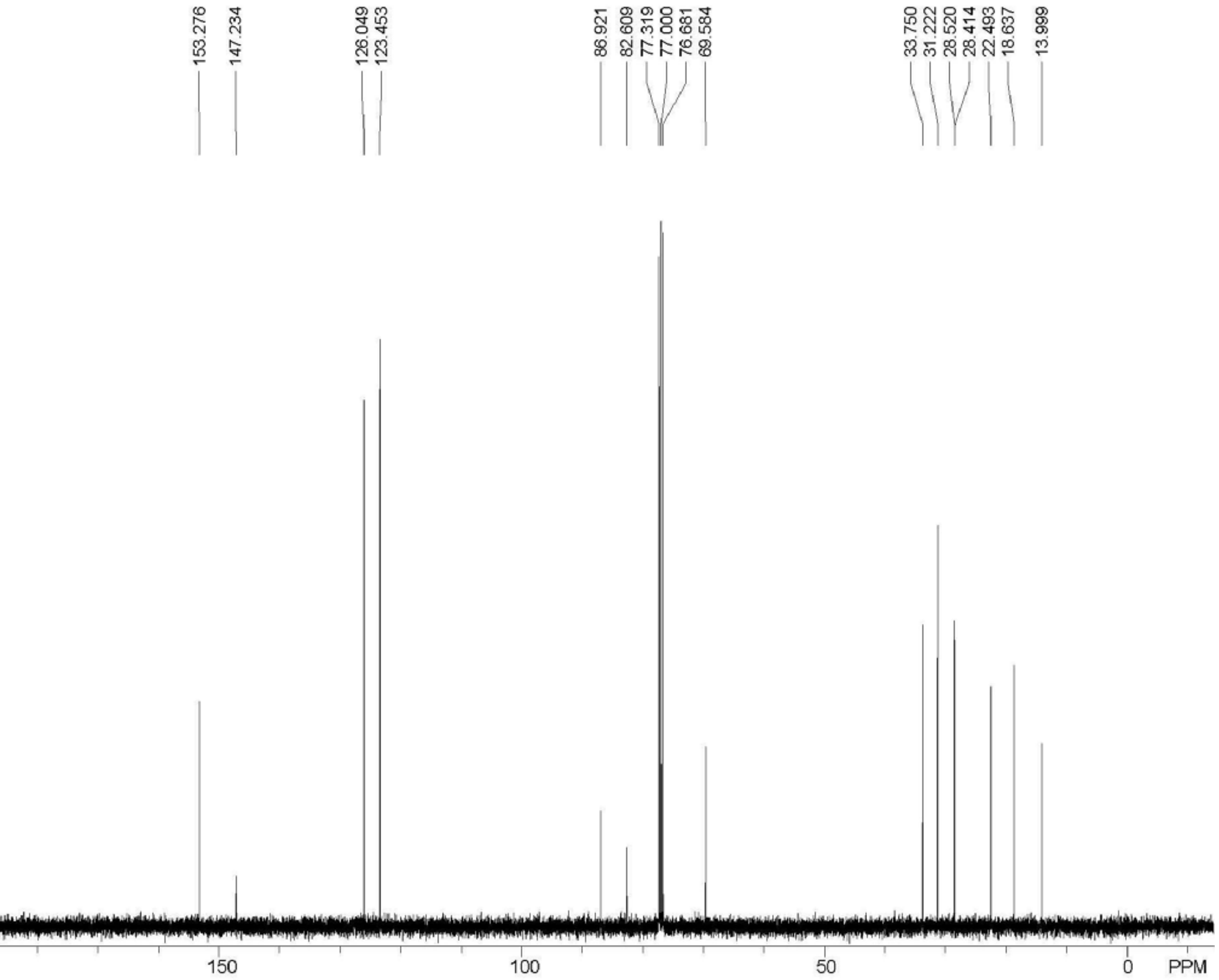

153.276
147.234
126.049
123.453
86.921
82.609
77.319
77.000
76.681
69.584
33.750
31.222
28.520
28.414
22.493
18.637
13.999
hcf-2-52-C
Nov 9 2017
SOLVENT: cdcl3
NA = 512
F1 = 100.527557 MHz
F2 = 399.749146 MHz
200
150
100
50
0
PPM

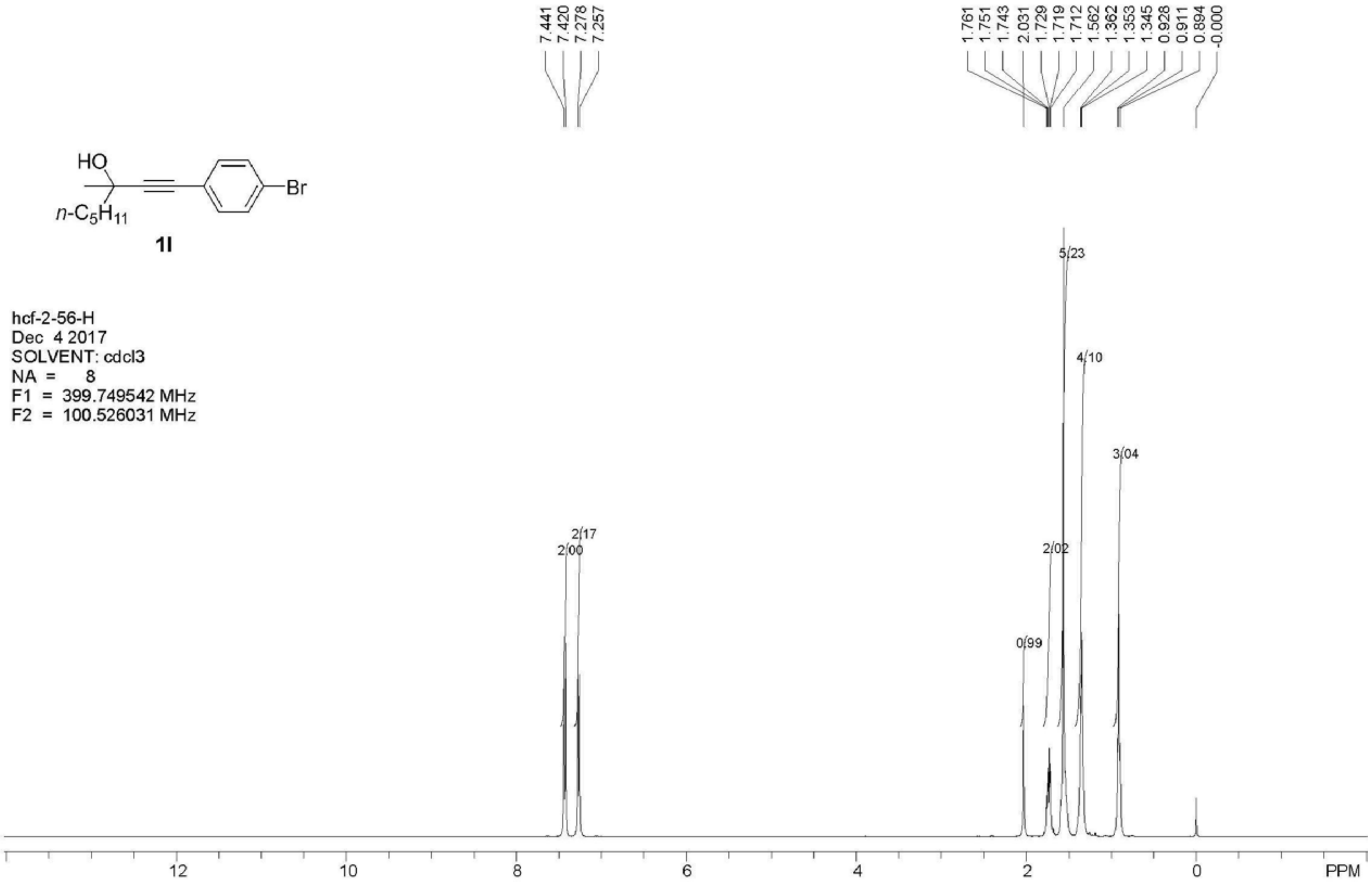
HO
n-C_5H_{11}
Br
1l
hcf-2-56-H
Dec 4 2017
SOLVENT: cdcl3
NA = 8
F1 = 399.749542 MHz
F2 = 100.526031 MHz
7.441
7.420
7.278
7.257
1.761
1.751
1.743
2.031
1.729
1.719
1.712
1.562
1.362
1.353
1.345
0.928
0.911
0.894
-0.000
2.00
2.17
0.99
2.02
5.23
4.10
3.04
12
10
8
6
4
2
0
PPM

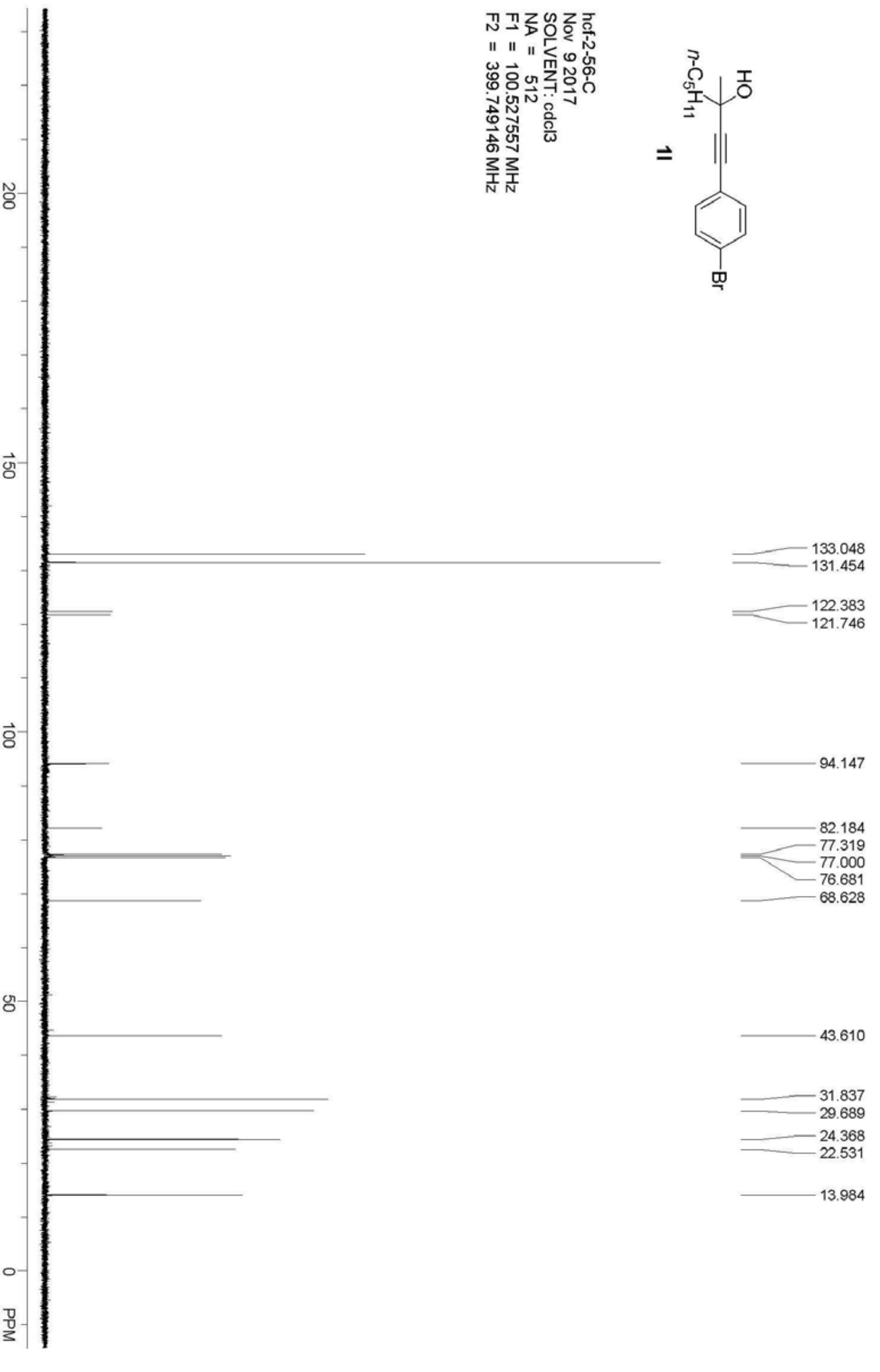
HO
n-C5H11
1l
Br
hcf-2-56-C
Nov 9 2017
SOLVENT: cdcl3
NA = 512
F1 = 100.527557 MHz
F2 = 399.749146 MHz
133.048
131.454
122.383
121.746
94.147
82.184
77.319
77.000
76.681
68.628
43.610
31.837
29.689
24.368
22.531
13.984
200
150
100
50
0
PPM

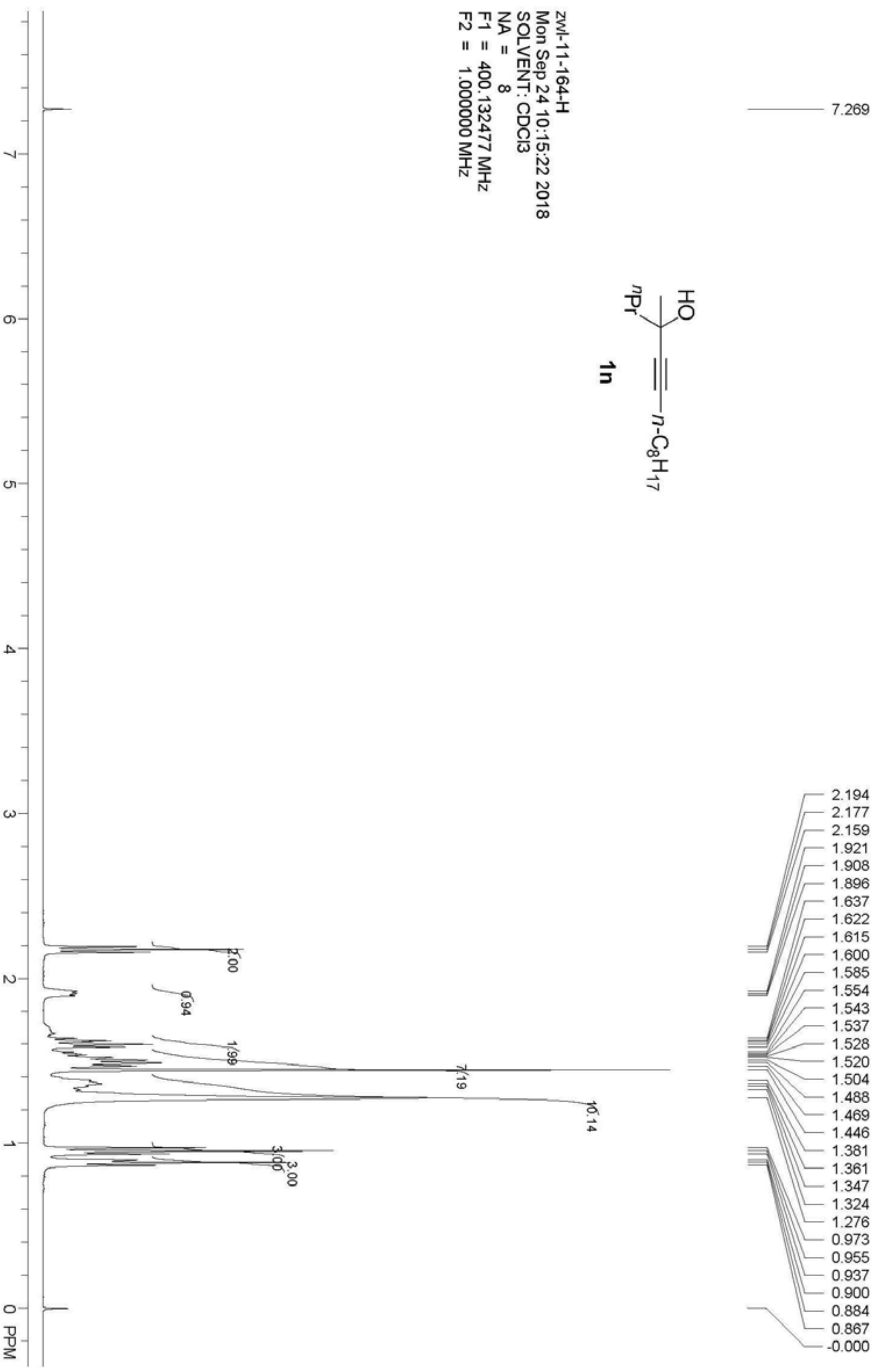
zwl-11-164-H
Mon Sep 24 10:15:22 2018
SOLVENT: CDCl3
NA = 8
F1 = 400.132477 MHz
F2 = 1.000000 MHz
HO
nPr
n-C8H17
1n
7.269
2.194
2.177
2.159
1.921
1.908
1.896
1.637
1.622
1.615
1.600
1.585
1.554
1.543
1.537
1.528
1.520
1.504
1.488
1.469
1.446
1.381
1.361
1.347
1.324
1.276
0.973
0.955
0.937
0.900
0.884
0.867
-0.000
2.00
0.94
1.99
7.19
10.14
3.00
3.00
7
6
5
4
3
2
1
0 PPM

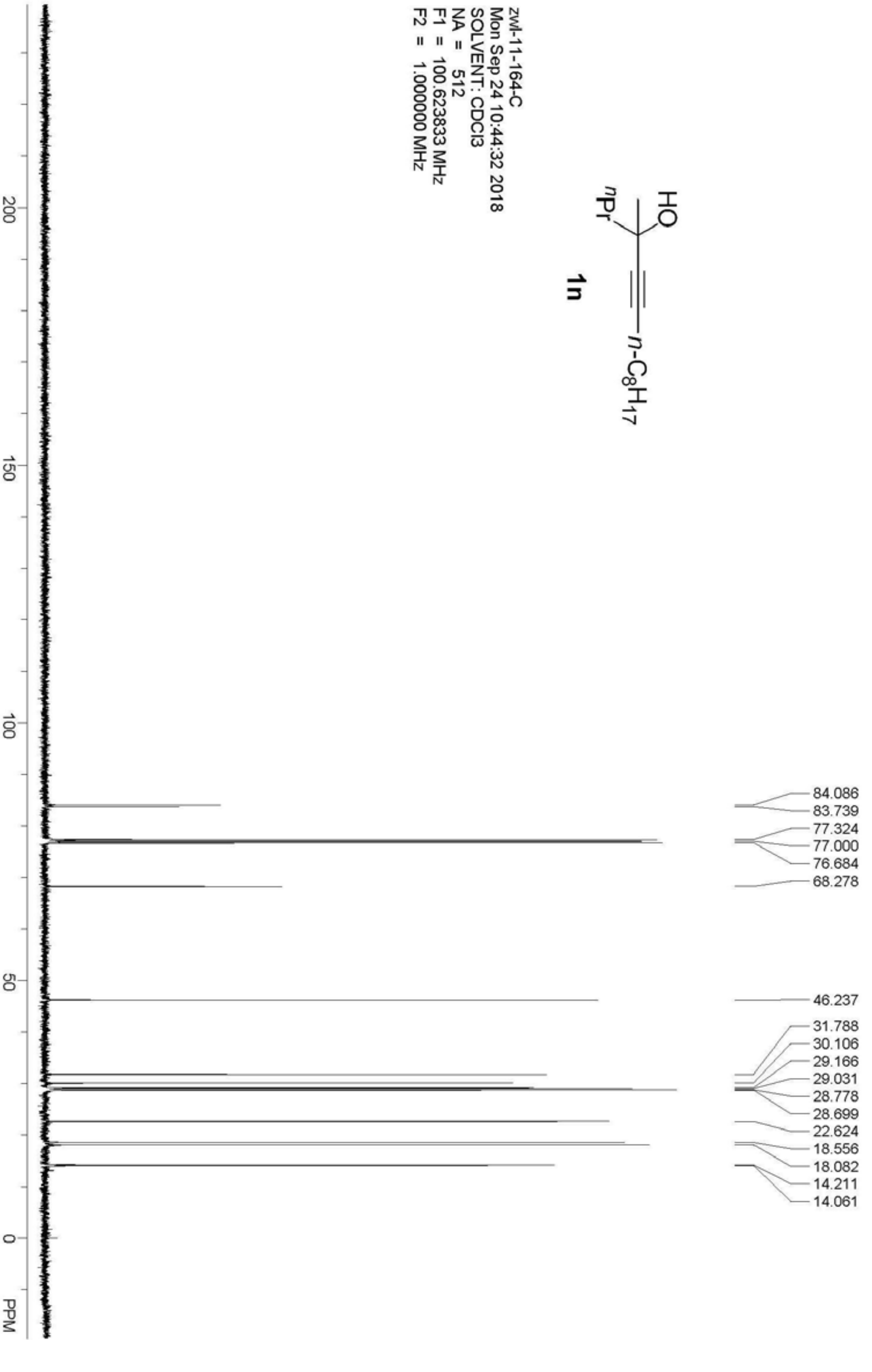
zwl-11-164-C
Mon Sep 24 10:44:32 2018
SOLVENT: CDCl3
NA = 512
F1 = 100.623833 MHz
F2 = 1.000000 MHz
HO
nPr
n-C8H17
1n
84.086
83.739
77.324
77.000
76.684
68.278
46.237
31.788
30.106
29.166
29.031
28.778
28.699
22.624
18.556
18.082
14.211
14.061
200
150
100
50
0
PPM

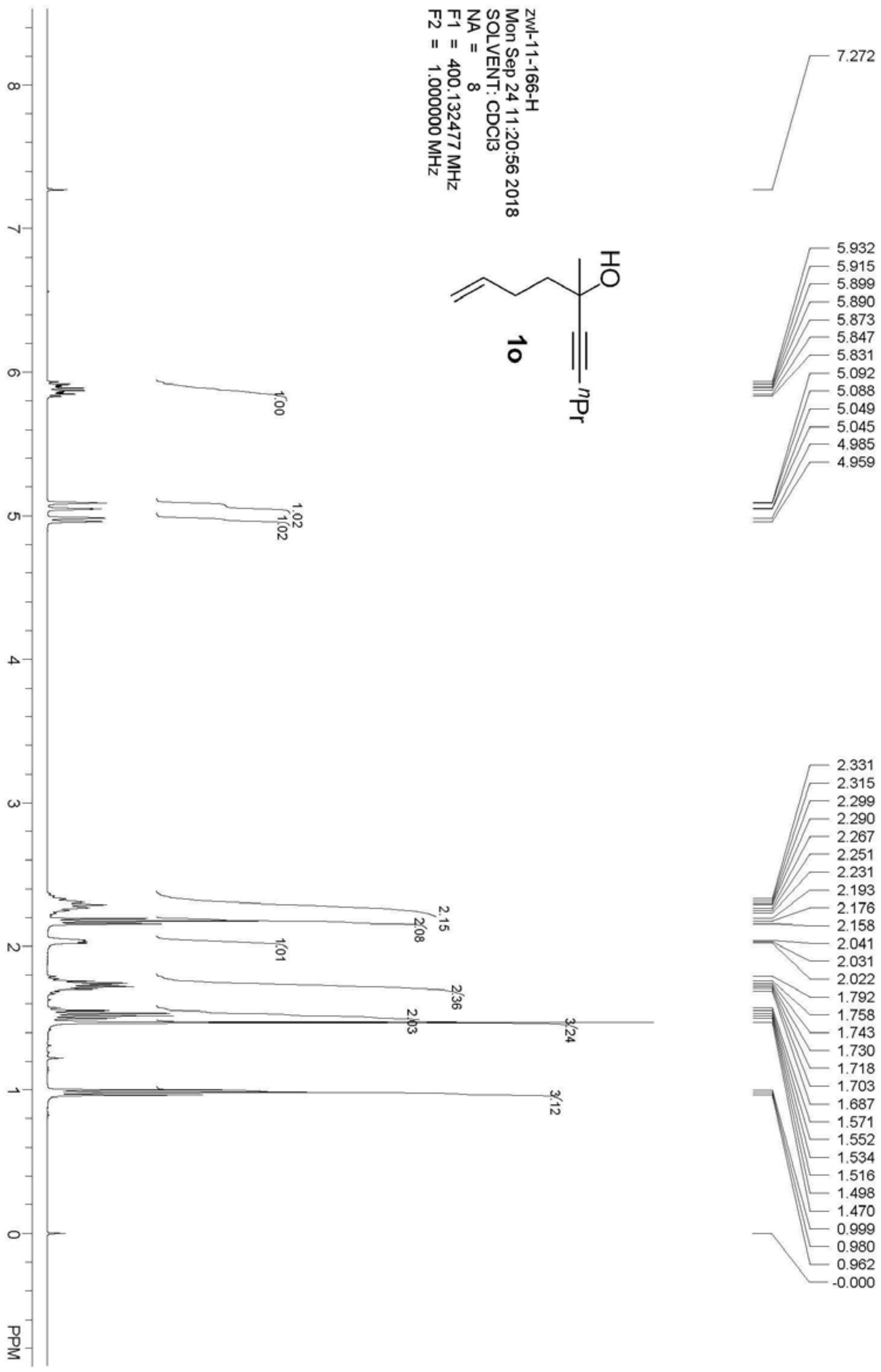
zwl-11-166-H
Mon Sep 24 11:20:56 2018
SOLVENT: CDCl3
NA = 8
F1 = 400.132477 MHz
F2 = 1.000000 MHz
HO
nPr
1o
7.272
5.932 5.915 5.899 5.890 5.873 5.847 5.831 5.092 5.088 5.049 5.045 4.985 4.959
2.331 2.315 2.299 2.290 2.267 2.251 2.231 2.193 2.176 2.158 2.041 2.031 2.022 1.792 1.758 1.743 1.730 1.718 1.703 1.687 1.571 1.552 1.534 1.516 1.498 1.470 0.999 0.980 0.962 -0.000
1.00 1.02 1.02 2.15 2.08 1.01 2.36 2.03 3.24 3.12
PPM

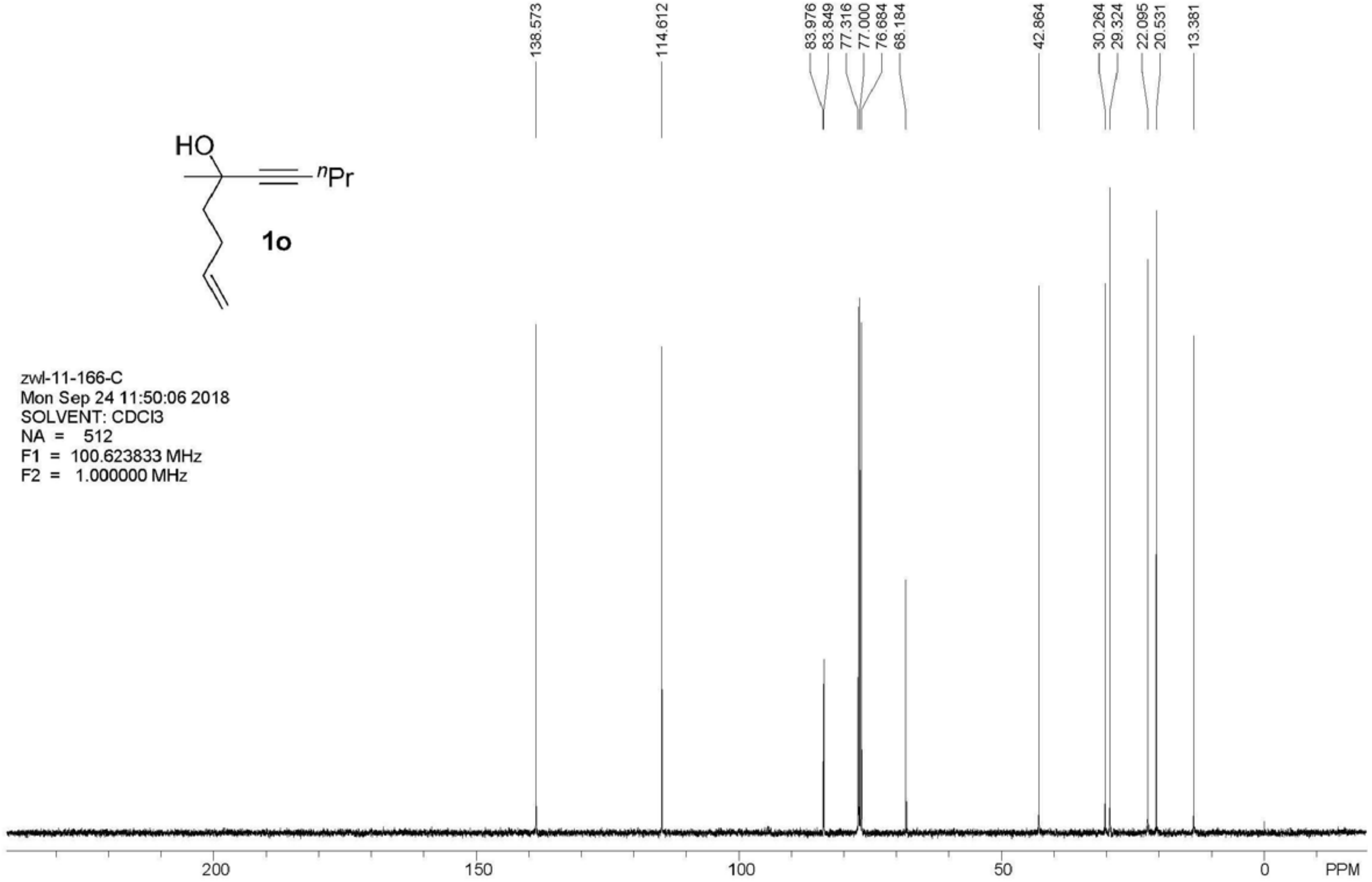

zwl-11-166-C
Mon Sep 24 11:50:06 2018
SOLVENT: CDCl3
NA = 512
F1 = 100.623833 MHz
F2 = 1.000000 MHz

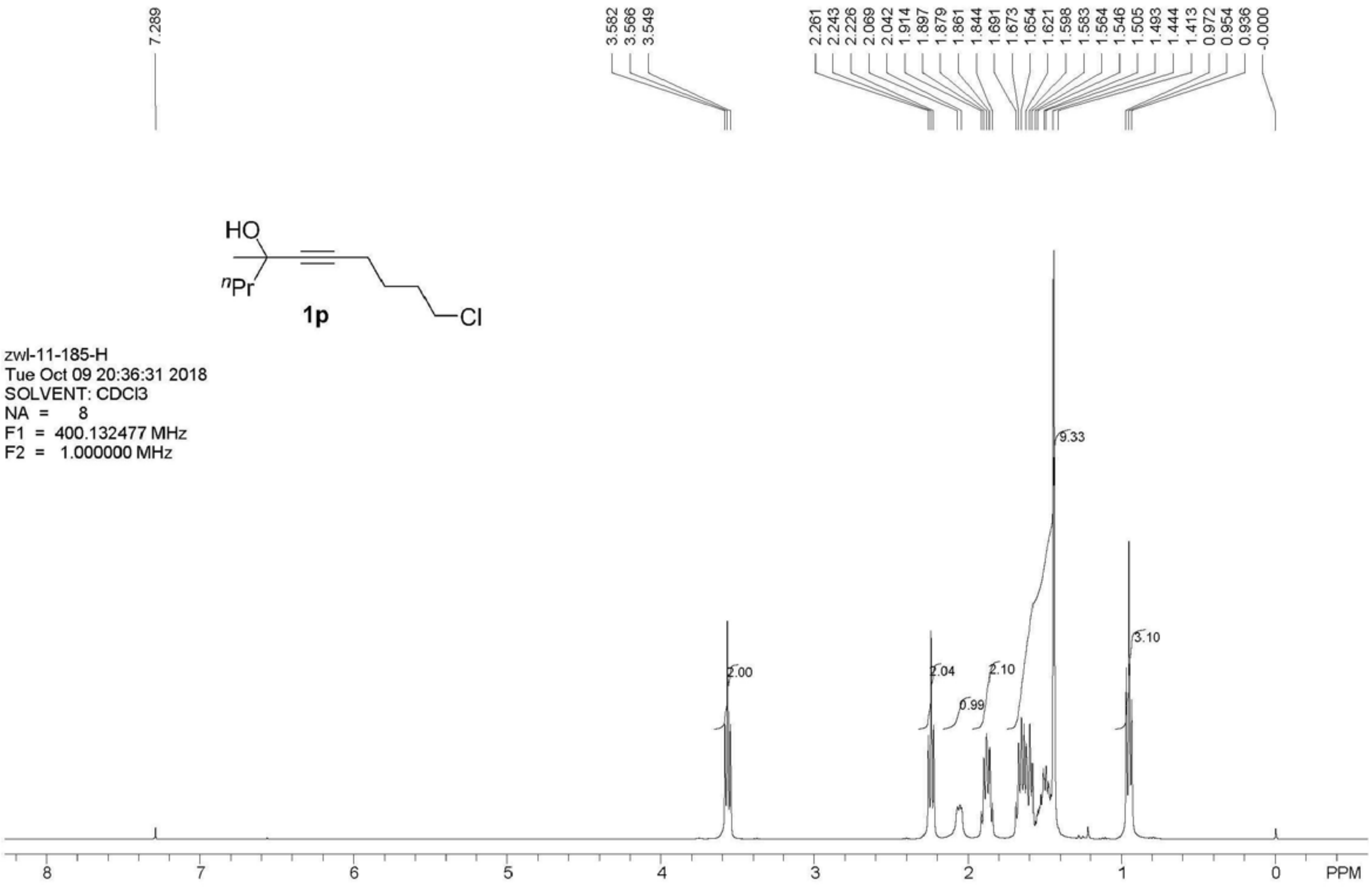
HO
nPr
1p
Cl
zwl-11-185-H
Tue Oct 09 20:36:31 2018
SOLVENT: CDCl3
NA = 8
F1 = 400.132477 MHz
F2 = 1.000000 MHz
7.289
3.582
3.566
3.549
2.261
2.243
2.226
2.069
2.042
1.914
1.897
1.879
1.861
1.844
1.691
1.673
1.654
1.621
1.598
1.583
1.564
1.546
1.505
1.493
1.444
1.413
0.972
0.954
0.936
-0.000
2.00
2.04
0.99
2.10
9.33
3.10
8
7
6
5
4
3
2
1
0
PPM

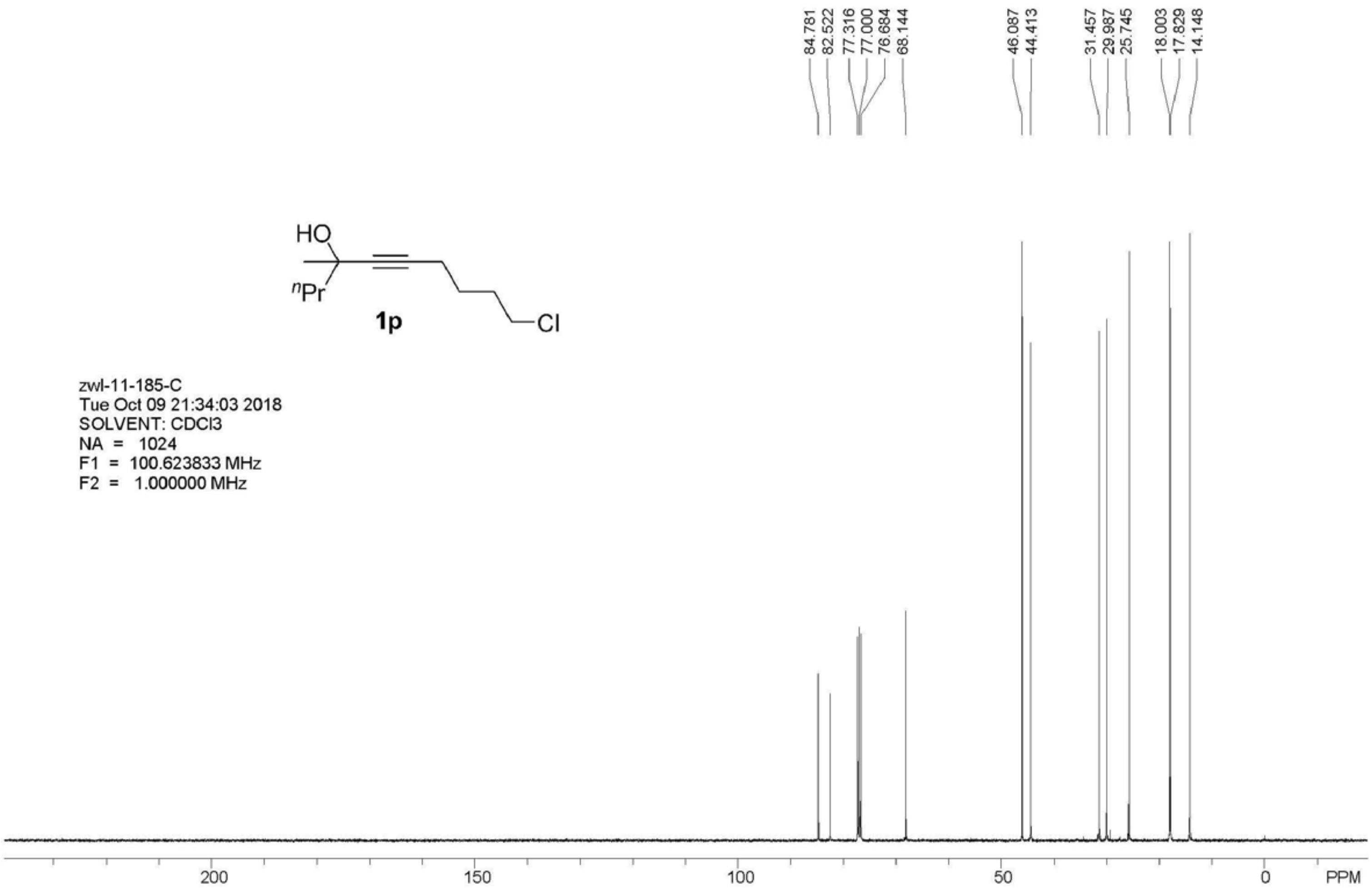
84.781
82.522
77.316
77.000
76.684
68.144
46.087
44.413
31.457
29.987
25.745
18.003
17.829
14.148
HO
ⁿPr
1p
Cl
zwl-11-185-C
Tue Oct 09 21:34:03 2018
SOLVENT: CDCl3
NA = 1024
F1 = 100.623833 MHz
F2 = 1.000000 MHz
200
150
100
50
0
PPM

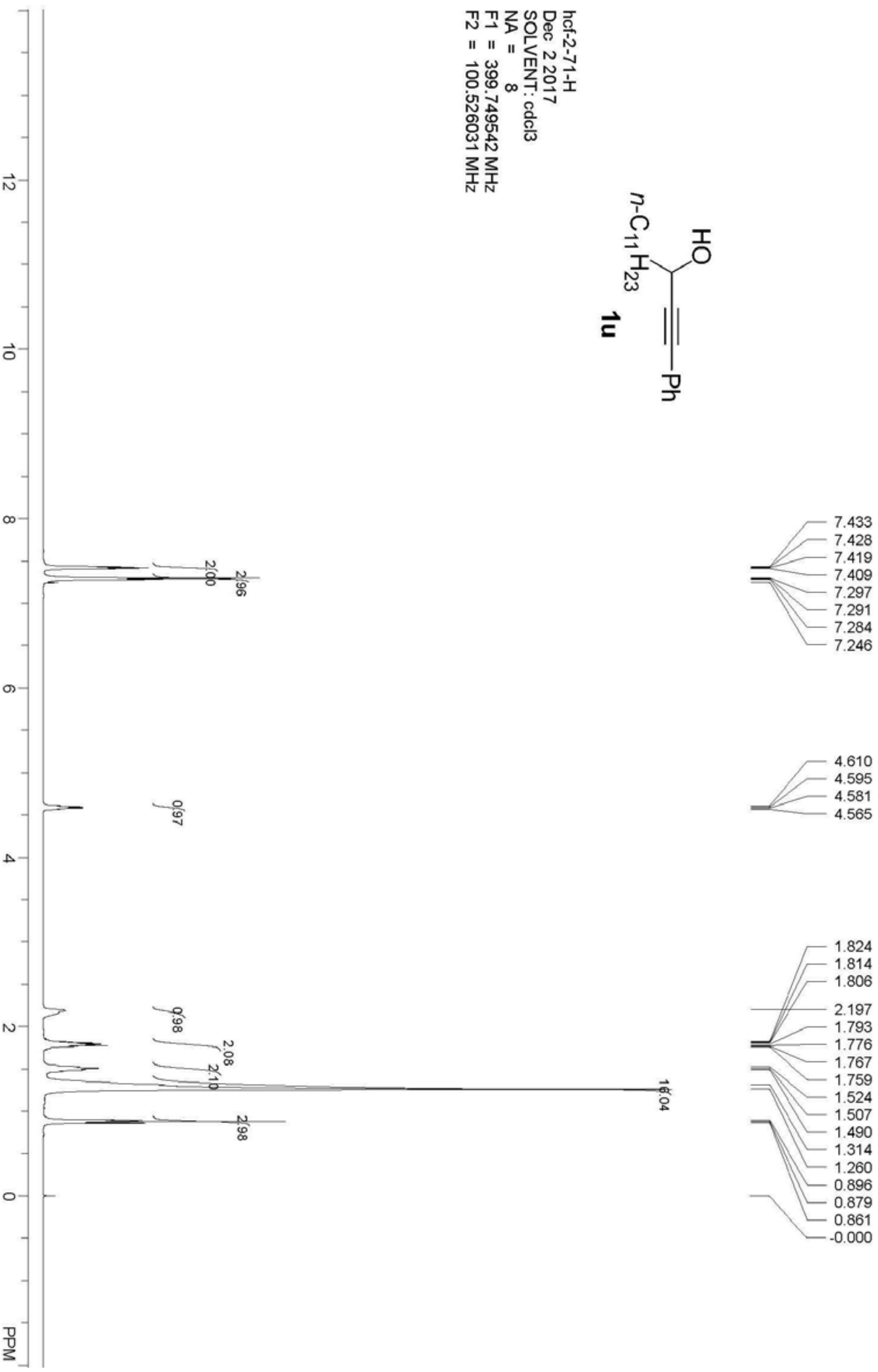
hcf-2-71-H
Dec 2 2017
SOLVENT: cdcl3
NA = 8
F1 = 399.749542 MHz
F2 = 100.526031 MHz
HO
n-C11H23
Ph
1u
7.433
7.428
7.419
7.409
7.297
7.291
7.284
7.246
4.610
4.595
4.581
4.565
1.824
1.814
1.806
2.197
1.793
1.776
1.767
1.759
1.524
1.507
1.490
1.314
1.260
0.896
0.879
0.861
-0.000
2.00
2.96
0.97
0.98
2.08
2.10
16.04
2.98
12
10
8
6
4
2
0
PPM

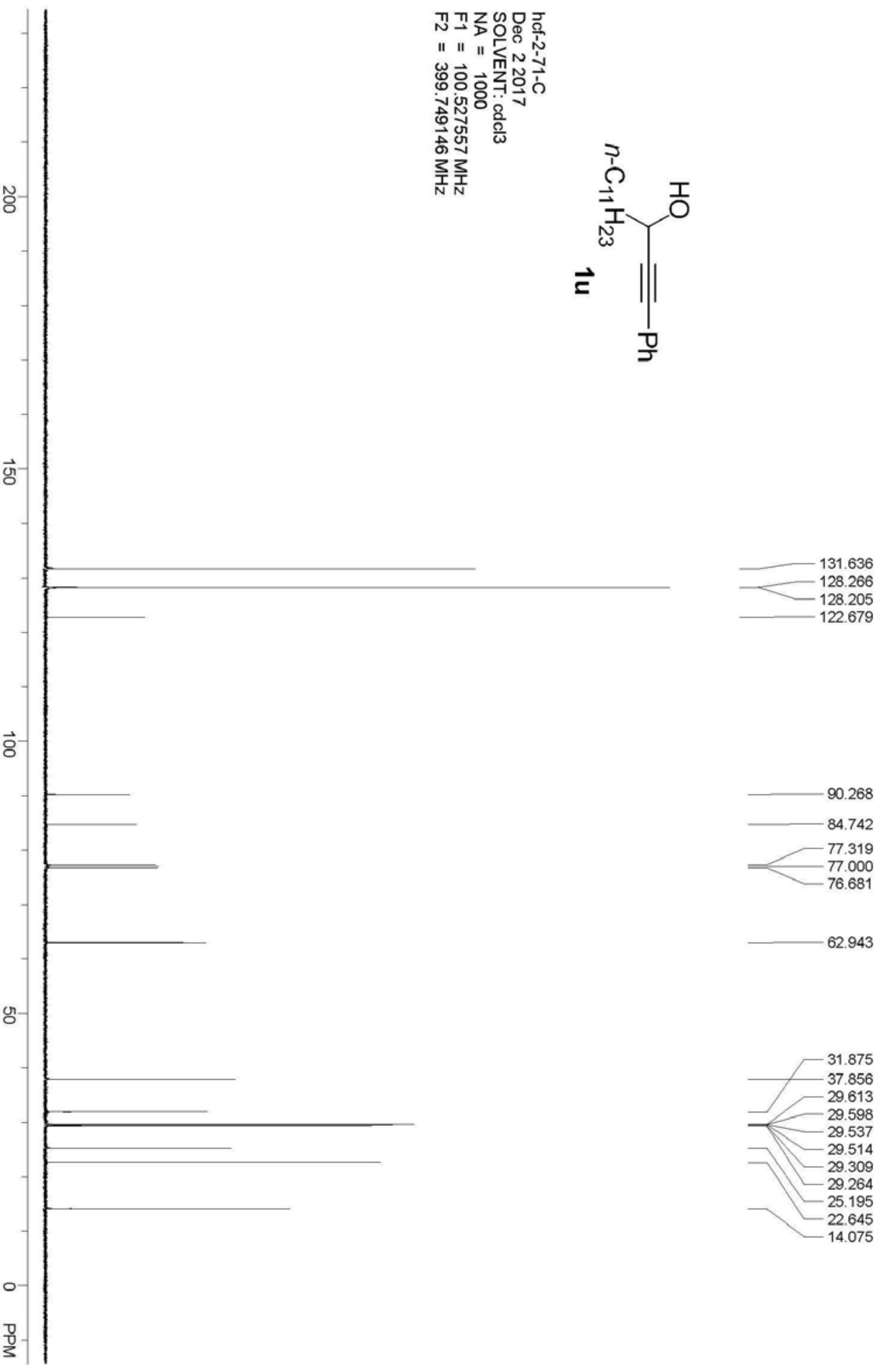
hcf-2-71-C
Dec 2 2017
SOLVENT: cdcl3
NA = 1000
F1 = 100.527557 MHz
F2 = 399.749146 MHz
HO
n-C11H23
Ph
1u
131.636
128.266
128.205
122.679
90.268
84.742
77.319
77.000
76.681
62.943
31.875
37.856
29.613
29.598
29.537
29.514
29.309
29.264
25.195
22.645
14.075
200
150
100
50
0
PPM

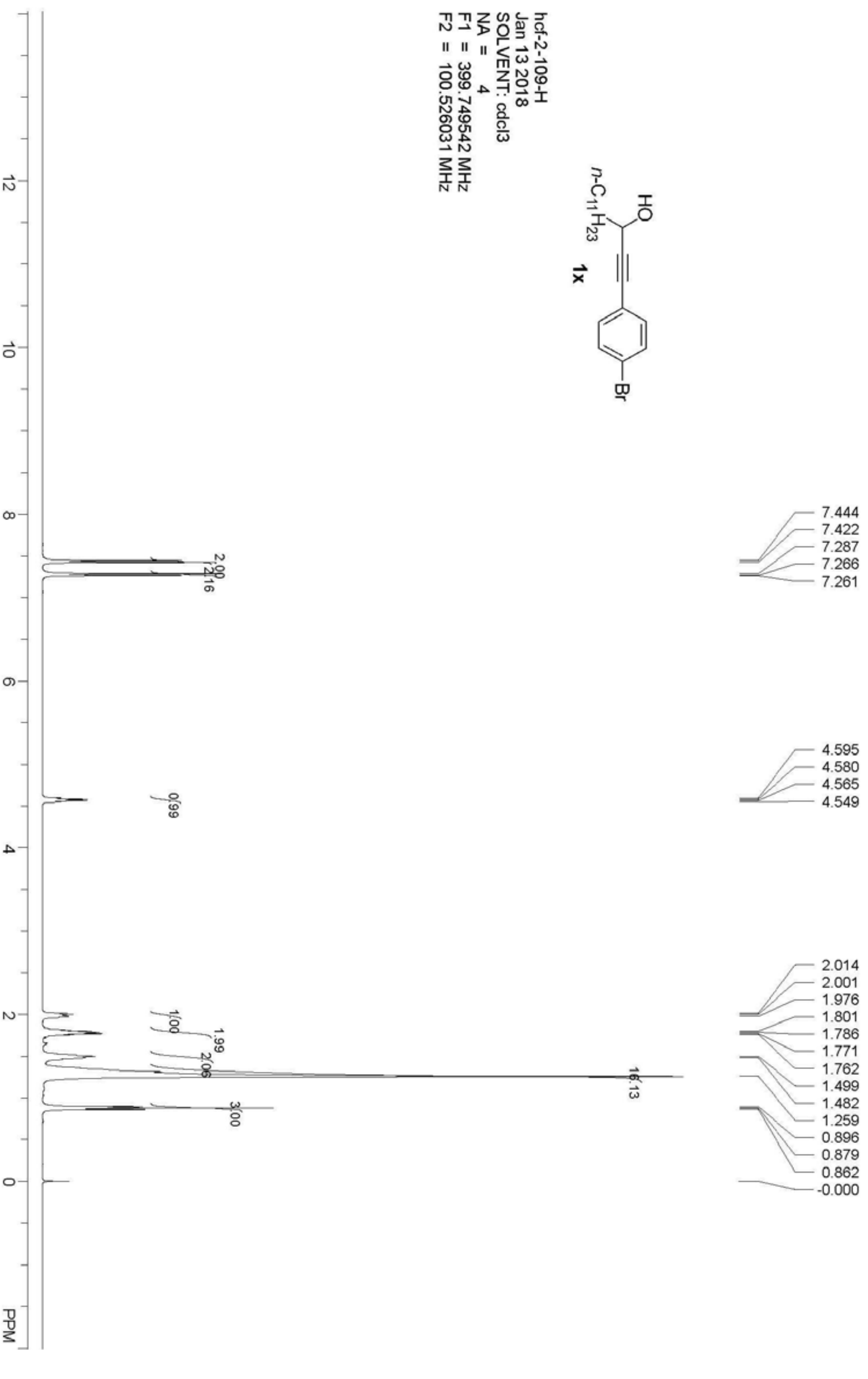
hcf-2-109-H
Jan 13 2018
SOLVENT: cdcl3
NA = 4
F1 = 399.749542 MHz
F2 = 100.526031 MHz
HO
n-C11H23
Br
1x
7.444
7.422
7.287
7.266
7.261
4.595
4.580
4.565
4.549
2.014
2.001
1.976
1.801
1.786
1.771
1.762
1.499
1.482
1.259
0.896
0.879
0.862
-0.000
2.00
2.16
0.99
1.00
1.99
2.06
16.13
3.00
12
10
8
6
4
2
0
PPM

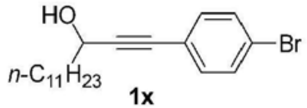

1x

hcf-2-109-C
Jan 13 2018
SOLVENT: cdcl3
NA = 512
F1 = 100.527557 MHz
F2 = 399.749146 MHz

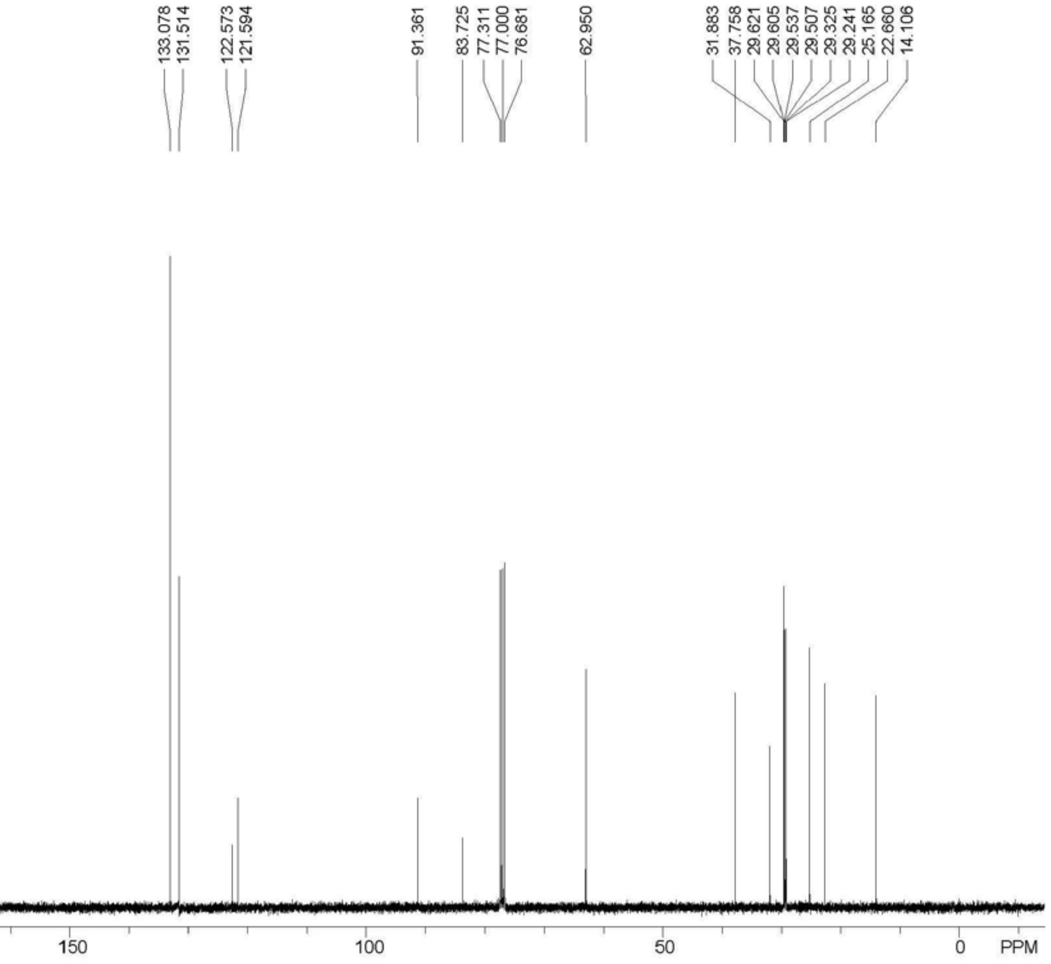

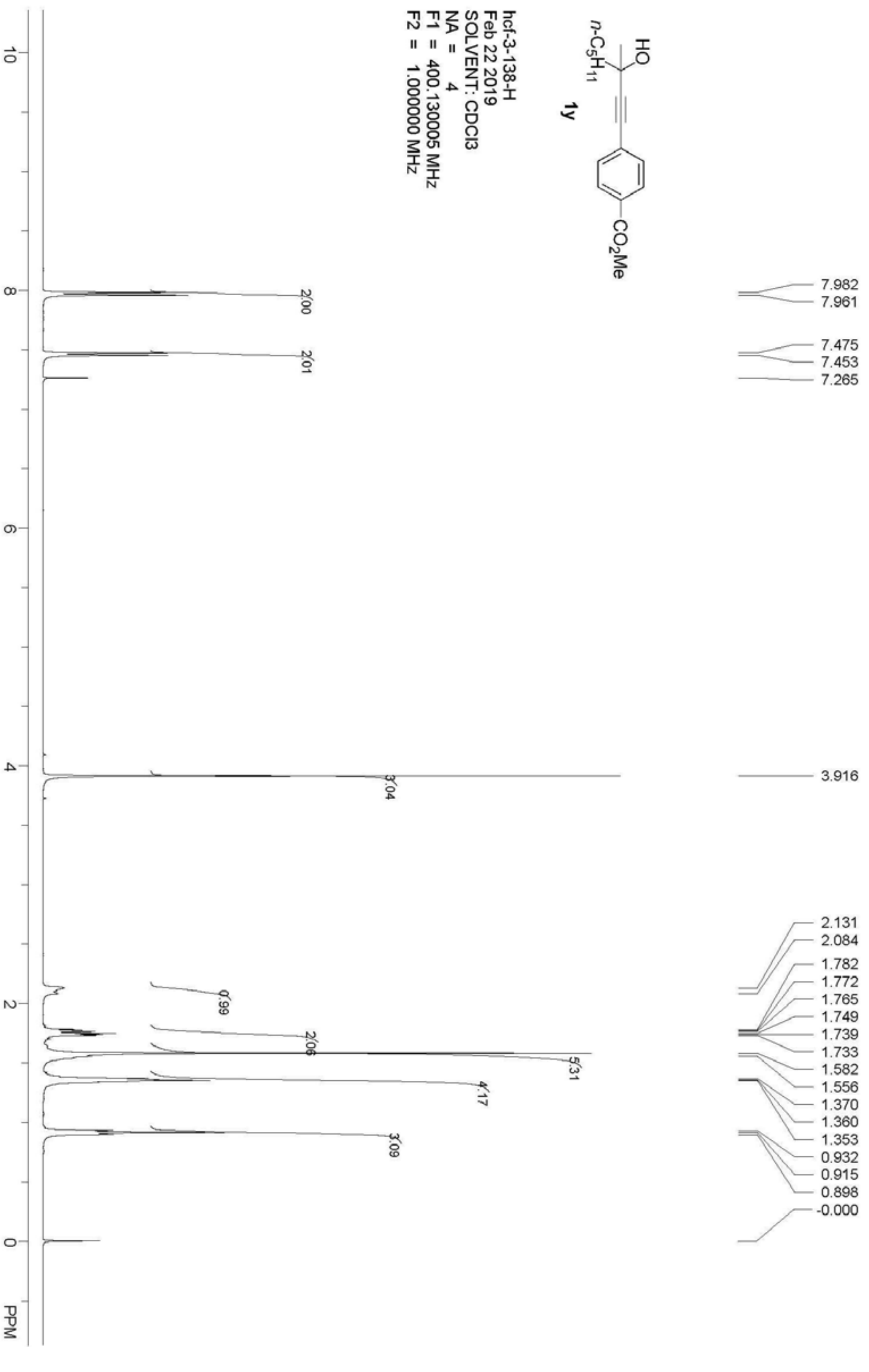
HO
n-C5H11
1y
CO2Me
hcf-3-138-H
Feb 22 2019
SOLVENT: CDCl3
NA = 4
F1 = 400.130005 MHz
F2 = 1.000000 MHz
2.00
2.01
3.04
0.99
2.06
5.31
4.17
3.09
7.982
7.961
7.475
7.453
7.265
3.916
2.131
2.084
1.782
1.772
1.765
1.749
1.739
1.733
1.582
1.556
1.370
1.360
1.353
0.932
0.915
0.898
-0.000
10
8
6
4
2
0
PPM

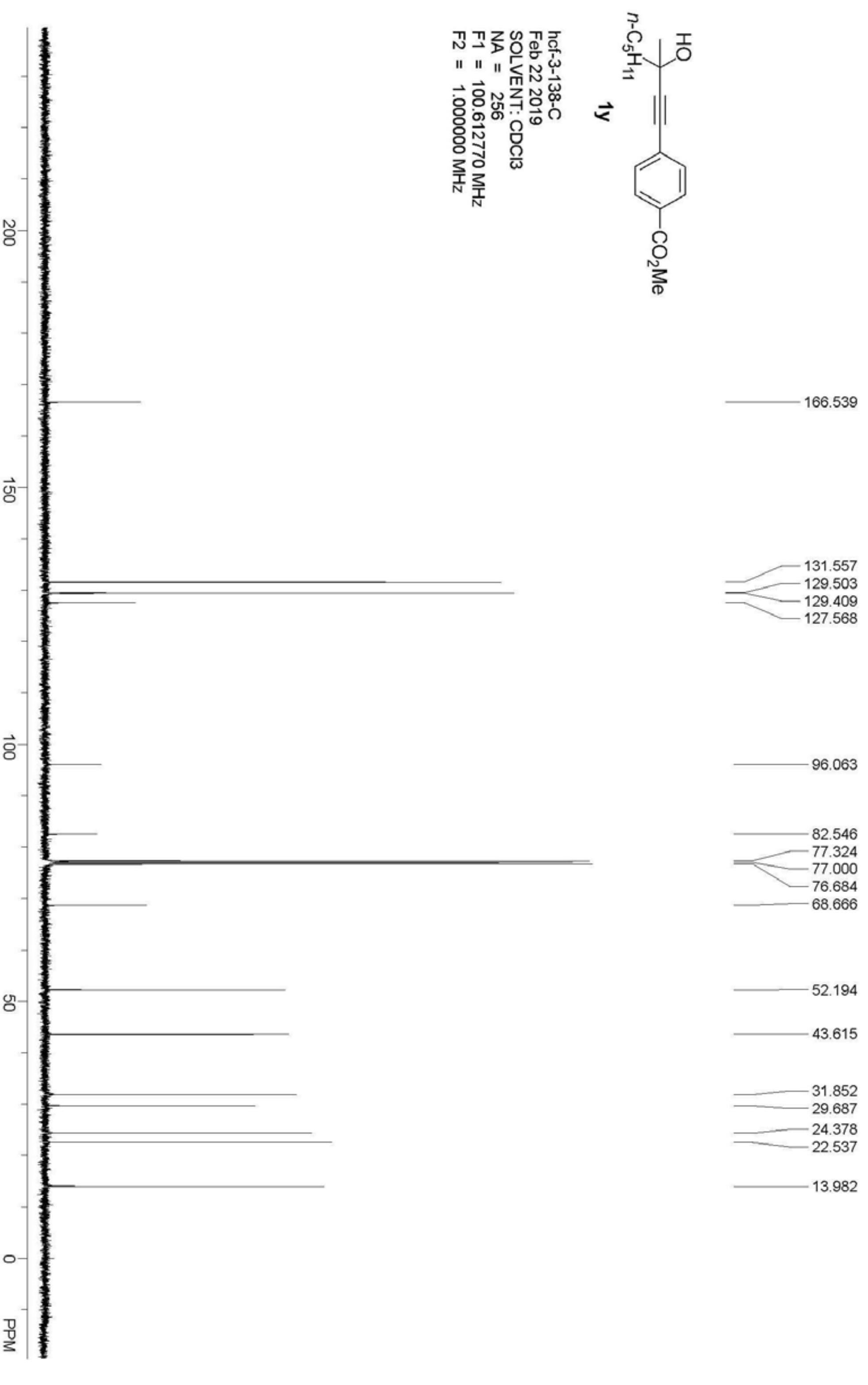
HO
n-C5H11
CO2Me
1y
hcf-3-138-C
Feb 22 2019
SOLVENT: CDCl3
NA = 256
F1 = 100.612770 MHz
F2 = 1.000000 MHz
166.539
131.557
129.503
129.409
127.568
96.063
82.546
77.324
77.000
76.684
68.666
52.194
43.615
31.852
29.687
24.378
22.537
13.982
200
150
100
50
0
PPM

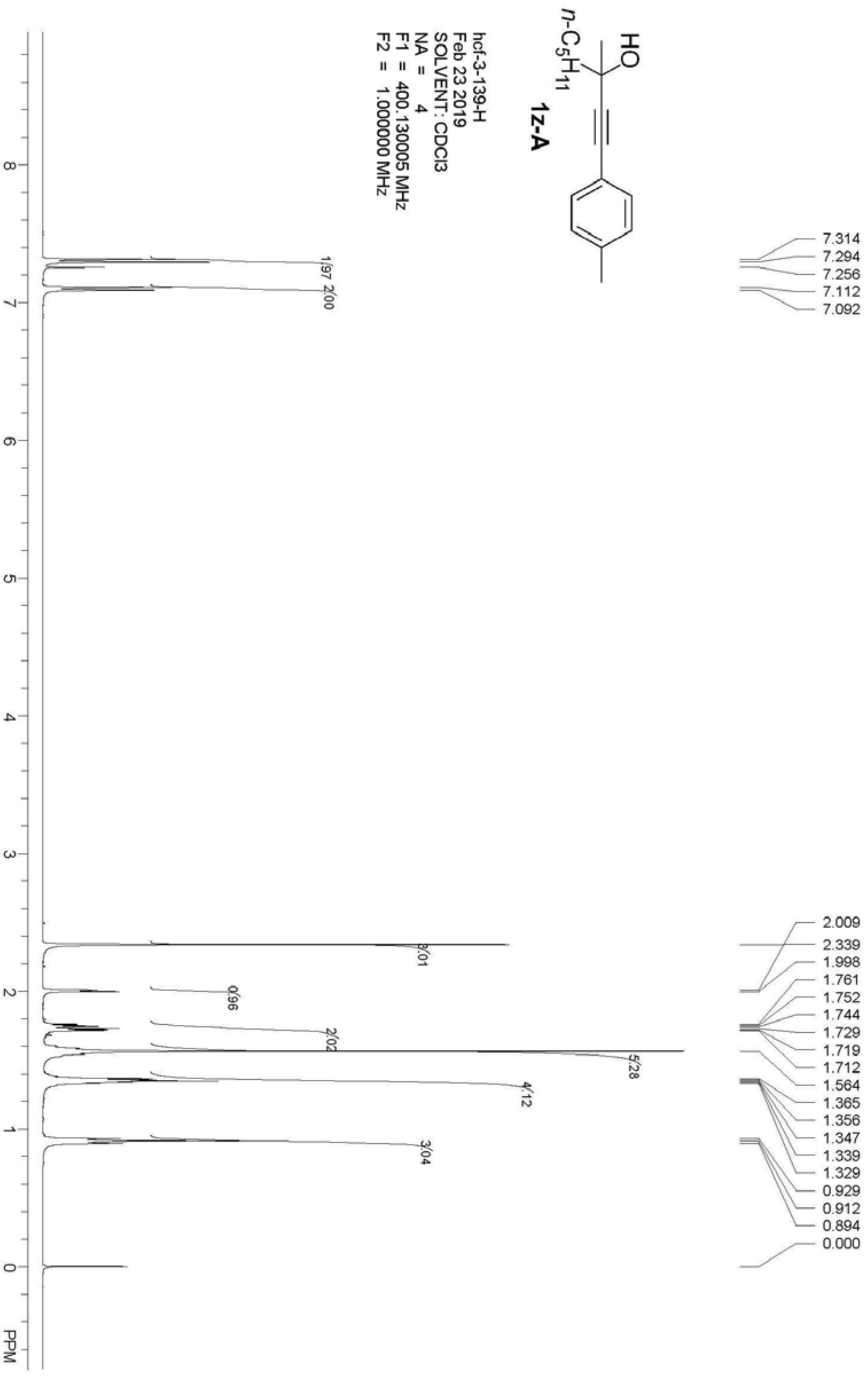

1z-A
HO
n-C5H11
hcf-3-139-H
Feb 23 2019
SOLVENT: CDCl3
NA = 4
F1 = 400.130005 MHz
F2 = 1.000000 MHz
7.314
7.294
7.256
7.112
7.092
2.009
2.339
1.998
1.761
1.752
1.744
1.729
1.719
1.712
1.564
1.365
1.356
1.347
1.339
1.329
0.929
0.912
0.894
0.000
1.97
2.00
3.01
0.96
2.02
5.28
4.12
3.04
PPM

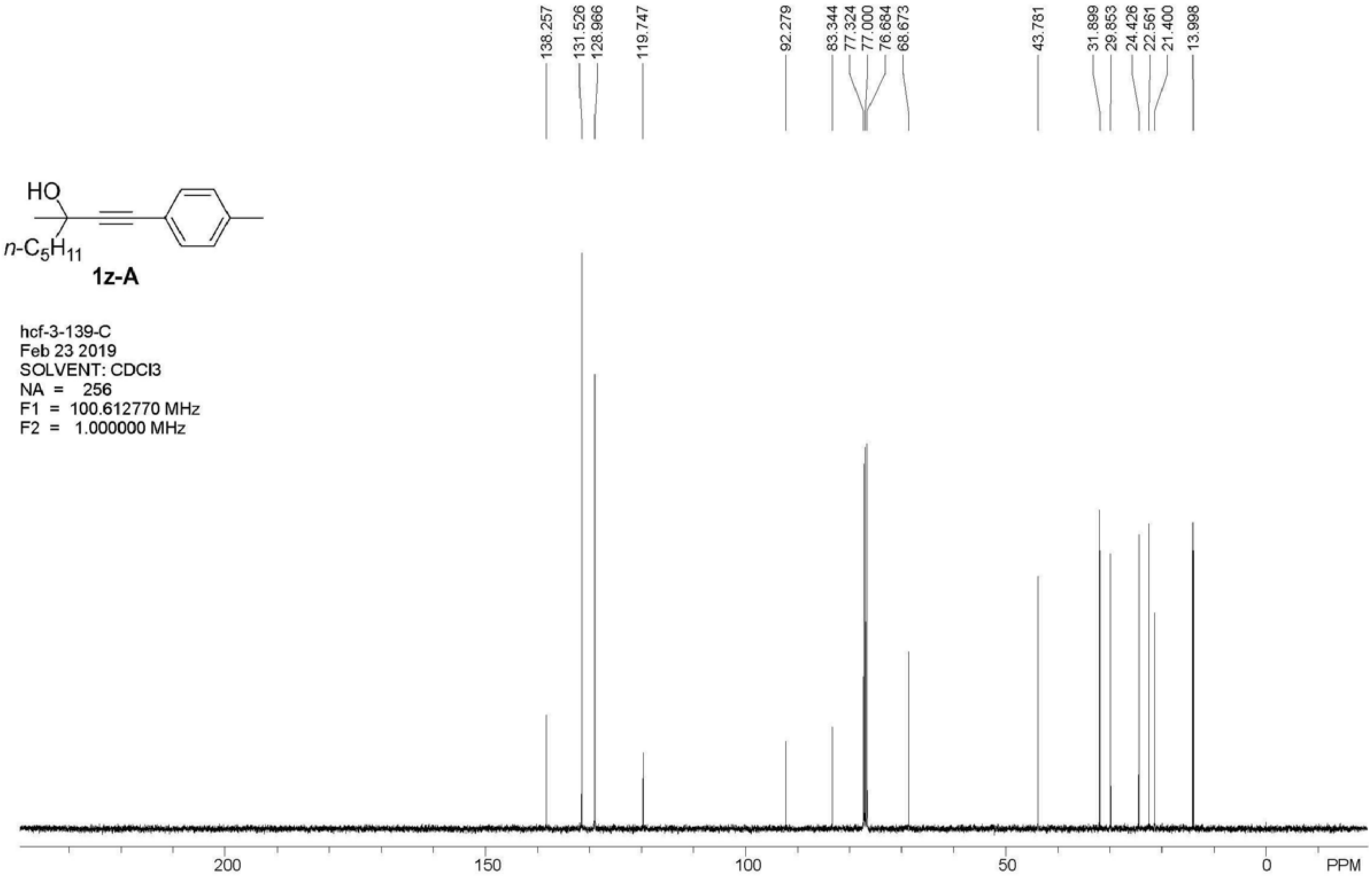
HO
n-C5H11
1z-A
hcf-3-139-C
Feb 23 2019
SOLVENT: CDCl3
NA = 256
F1 = 100.612770 MHz
F2 = 1.000000 MHz
138.257
131.526
128.966
119.747
92.279
83.344
77.324
77.000
76.684
68.673
43.781
31.899
29.853
24.426
22.561
21.400
13.998
200
150
100
50
0
PPM

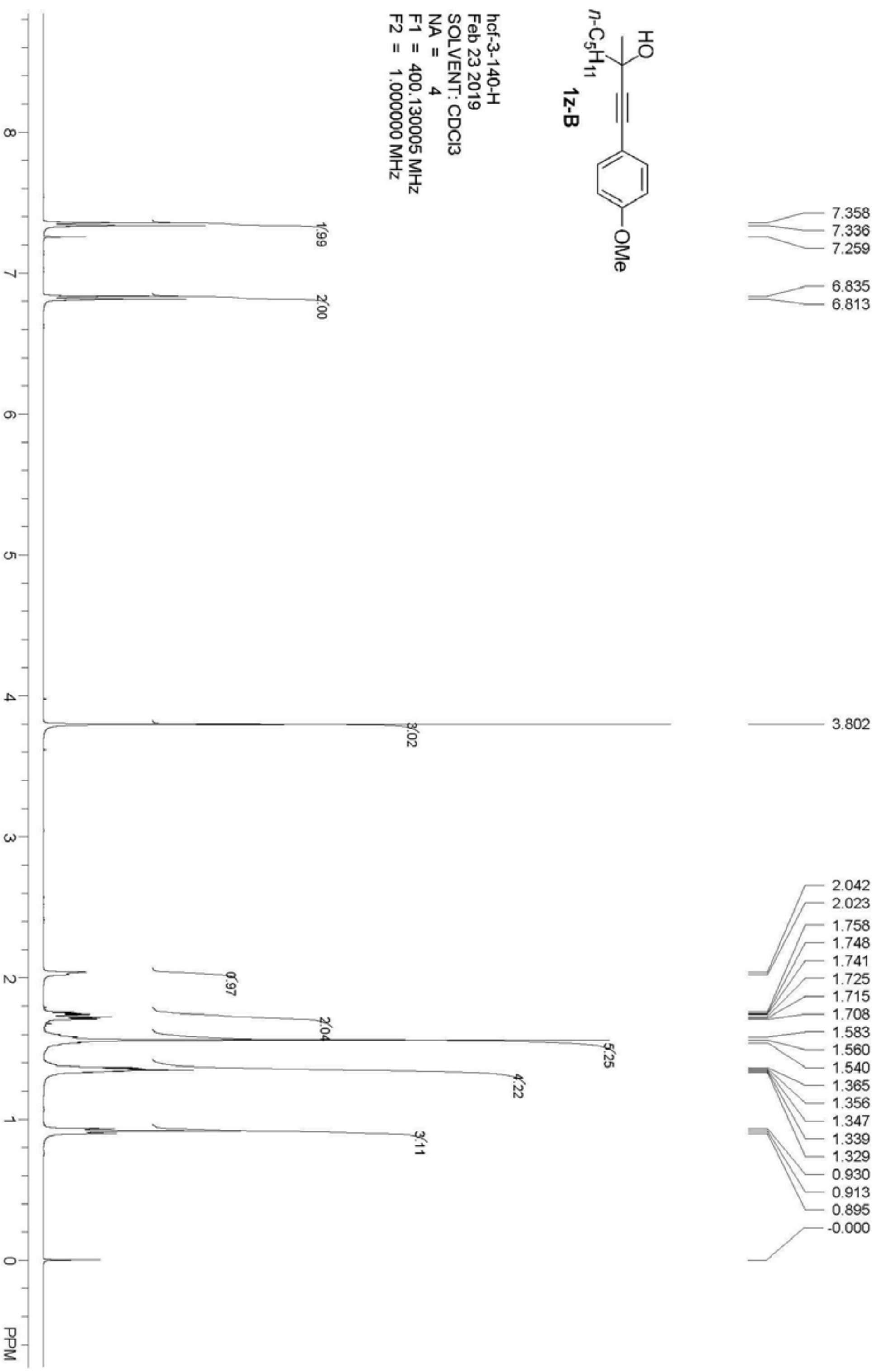
1z-B
OMe
hcf-3-140-H
Feb 23 2019
SOLVENT: CDCl3
NA = 4
F1 = 400.130005 MHz
F2 = 1.000000 MHz
7.358
7.336
7.259
6.835
6.813
3.802
2.042
2.023
1.758
1.748
1.741
1.725
1.715
1.708
1.583
1.560
1.540
1.365
1.356
1.347
1.339
1.329
0.930
0.913
0.895
-0.000
1.99
2.00
3.02
0.97
2.04
5.25
4.22
3.11
PPM

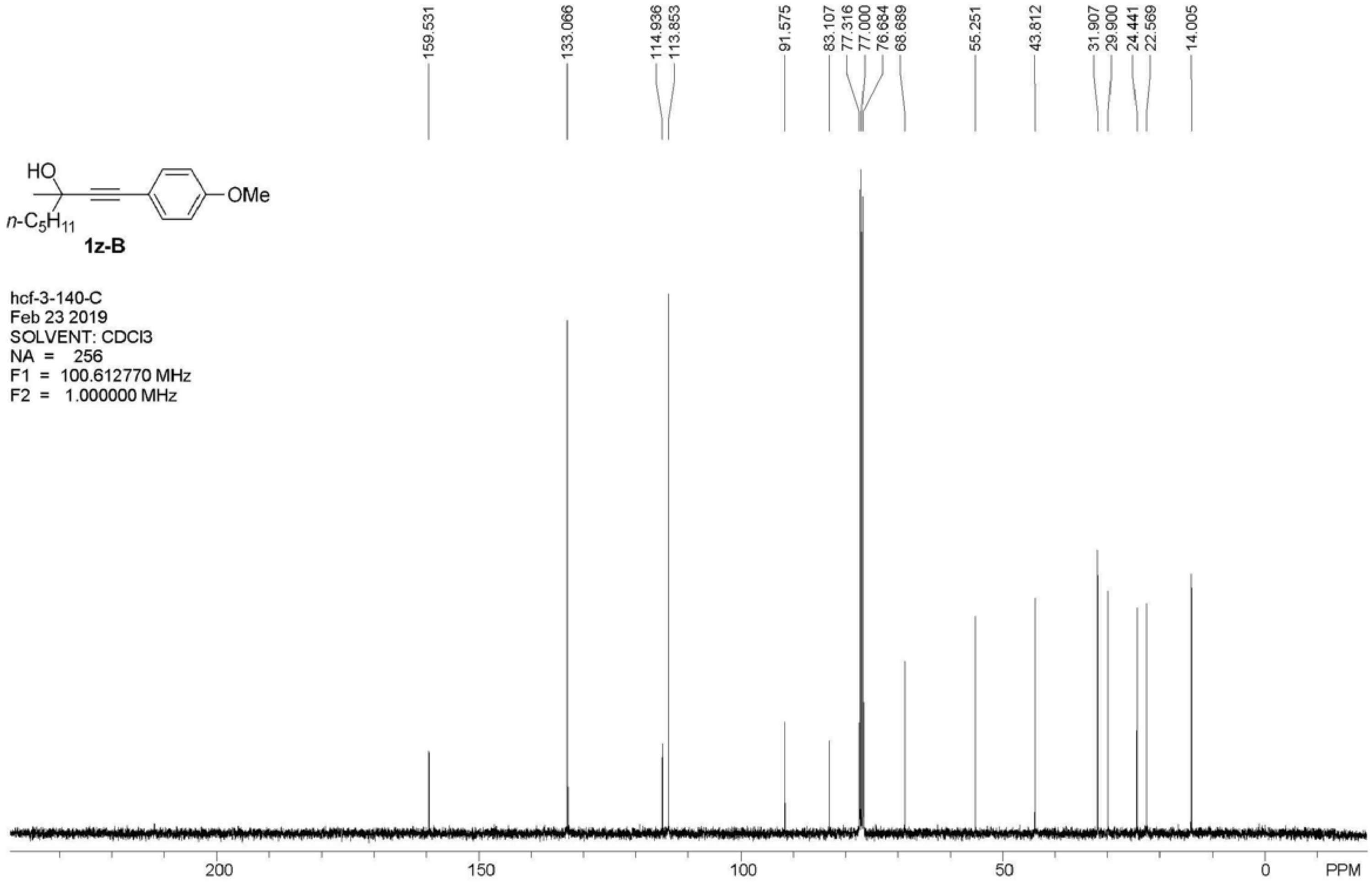

1z-B

hcf-3-140-C
Feb 23 2019
SOLVENT: CDCl3
NA = 256
F1 = 100.612770 MHz
F2 = 1.000000 MHz

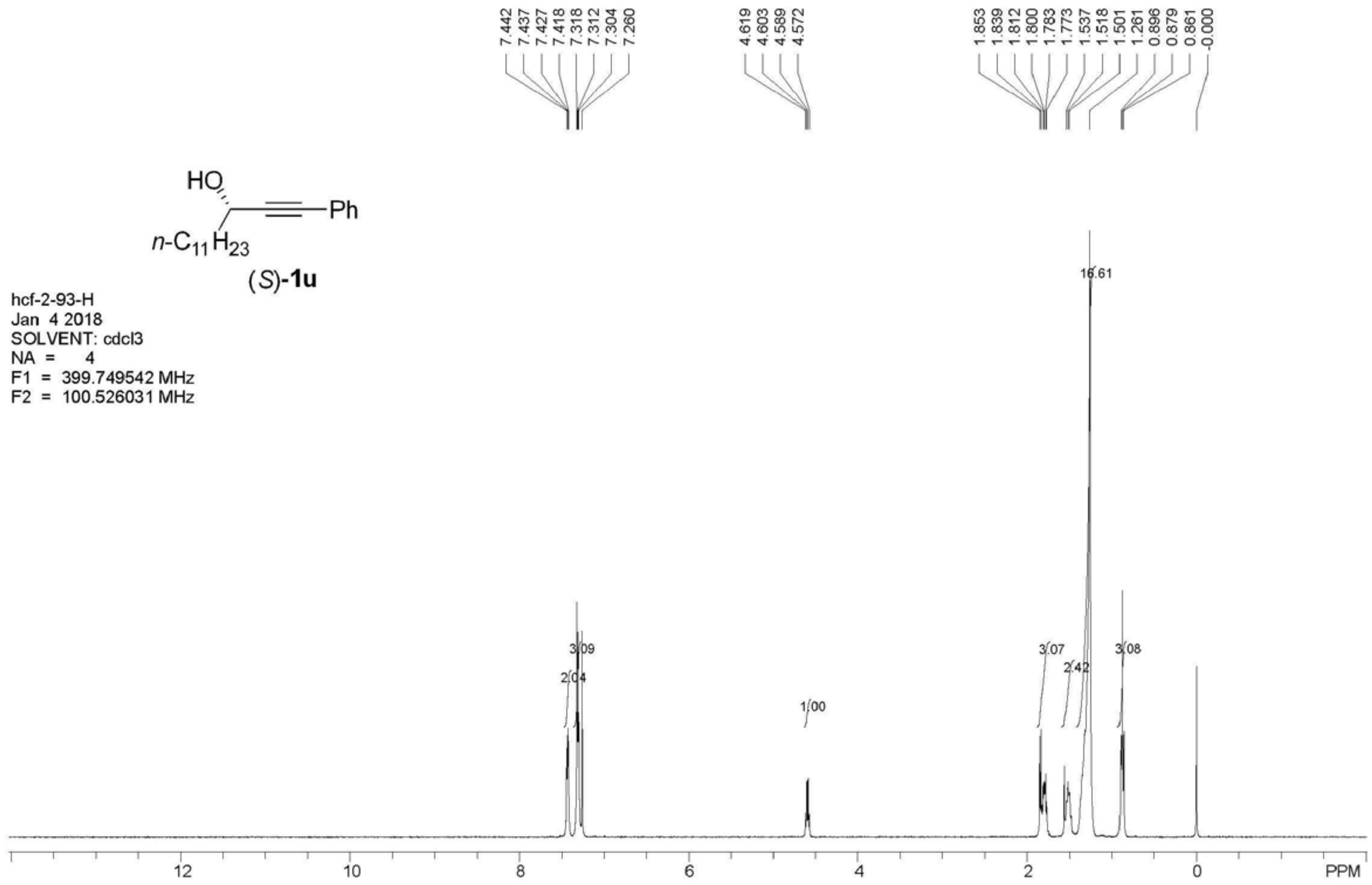
HO
Ph
n-C11H23
(S)-1u
hcf-2-93-H
Jan 4 2018
SOLVENT: cdcl3
NA = 4
F1 = 399.749542 MHz
F2 = 100.526031 MHz
7.442
7.437
7.427
7.418
7.318
7.312
7.304
7.260
4.619
4.603
4.589
4.572
1.853
1.839
1.812
1.800
1.783
1.773
1.537
1.518
1.501
1.261
0.896
0.879
0.861
-0.000
2.04
3.09
1.00
3.07
2.42
16.61
3.08
12
10
8
6
4
2
0
PPM

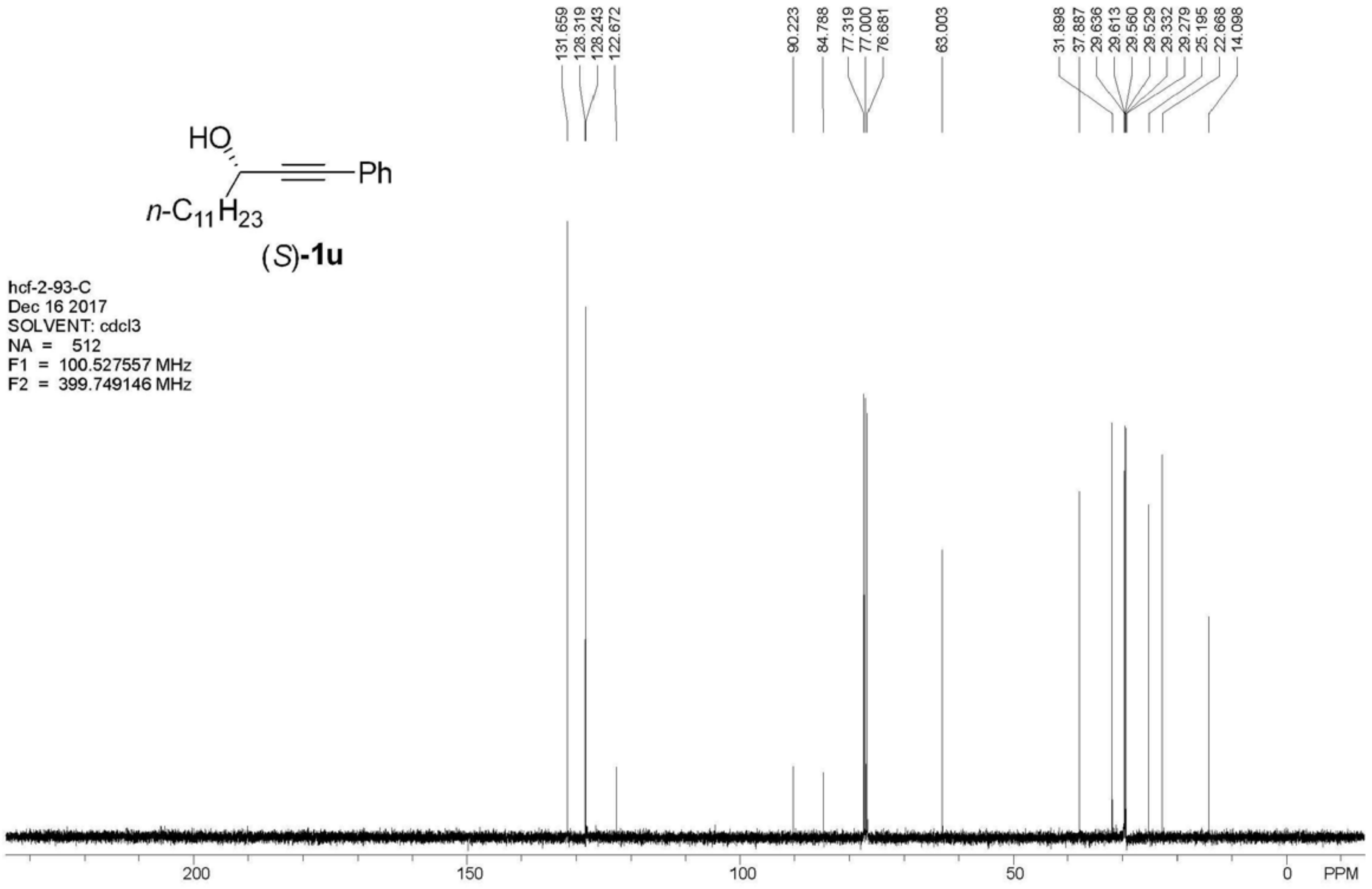
HO
Ph
n-C11H23
(S)-1u
hcf-2-93-C
Dec 16 2017
SOLVENT: cdcl3
NA = 512
F1 = 100.527557 MHz
F2 = 399.749146 MHz
131.659
128.319
128.243
122.672
90.223
84.788
77.319
77.000
76.681
63.003
31.898
37.887
29.636
29.613
29.560
29.529
29.332
29.279
25.195
22.668
14.098
200
150
100
50
0
PPM

hcf-2-71-pa-2-95-5-0.7-214

实验时间：2017-12-21, 16:55:54　　　　报告时间：2017-12-21, 18:24:23
谱图文件:D:\zhuguangjiong\hcf\20171221\hcf-2-71-pa-2-95-5-0.7-214.org

实验内容简介：

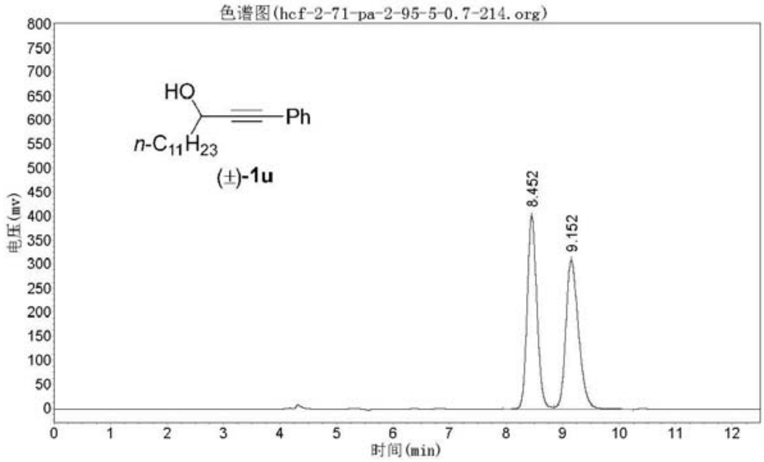

分析结果表

峰号	峰名	保留时间	峰高	峰面积	含量
1		8.452	401822.000	4787561.500	49.8038
2		9.152	309477.500	4825286.500	50.1962
**总计**			711299.500	9612848.000	100.0000

hcf-2-93-pa-2-95-5-0.7-214

实验时间：2017-12-21, 17:13:49　　　　报告时间：2017-12-21, 18:25:50
谱图文件:D:\zhuguangjiong\hcf\20171221\hcf-2-93-pa-2-95-5-0.7-214.org

实验内容简介：

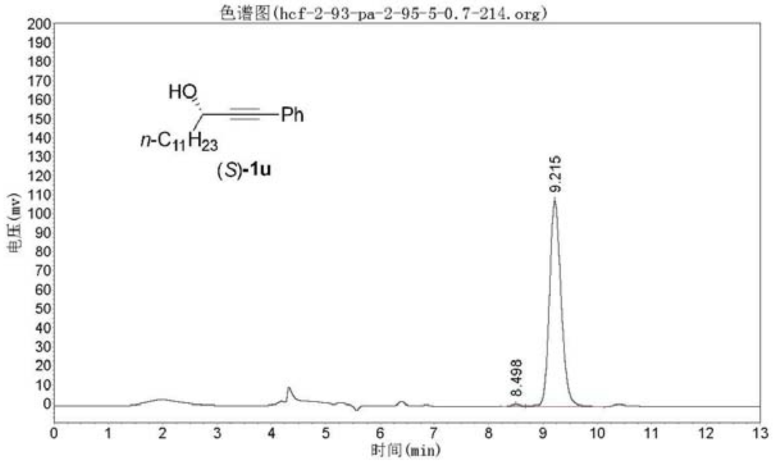

分析结果表

峰号	峰名	保留时间	峰高	峰面积	含量
1		8.498	1178.903	14763.397	0.9050
2		9.215	108352.688	1616500.625	99.0950
**总计**			109531.590	1631264.022	100.0000

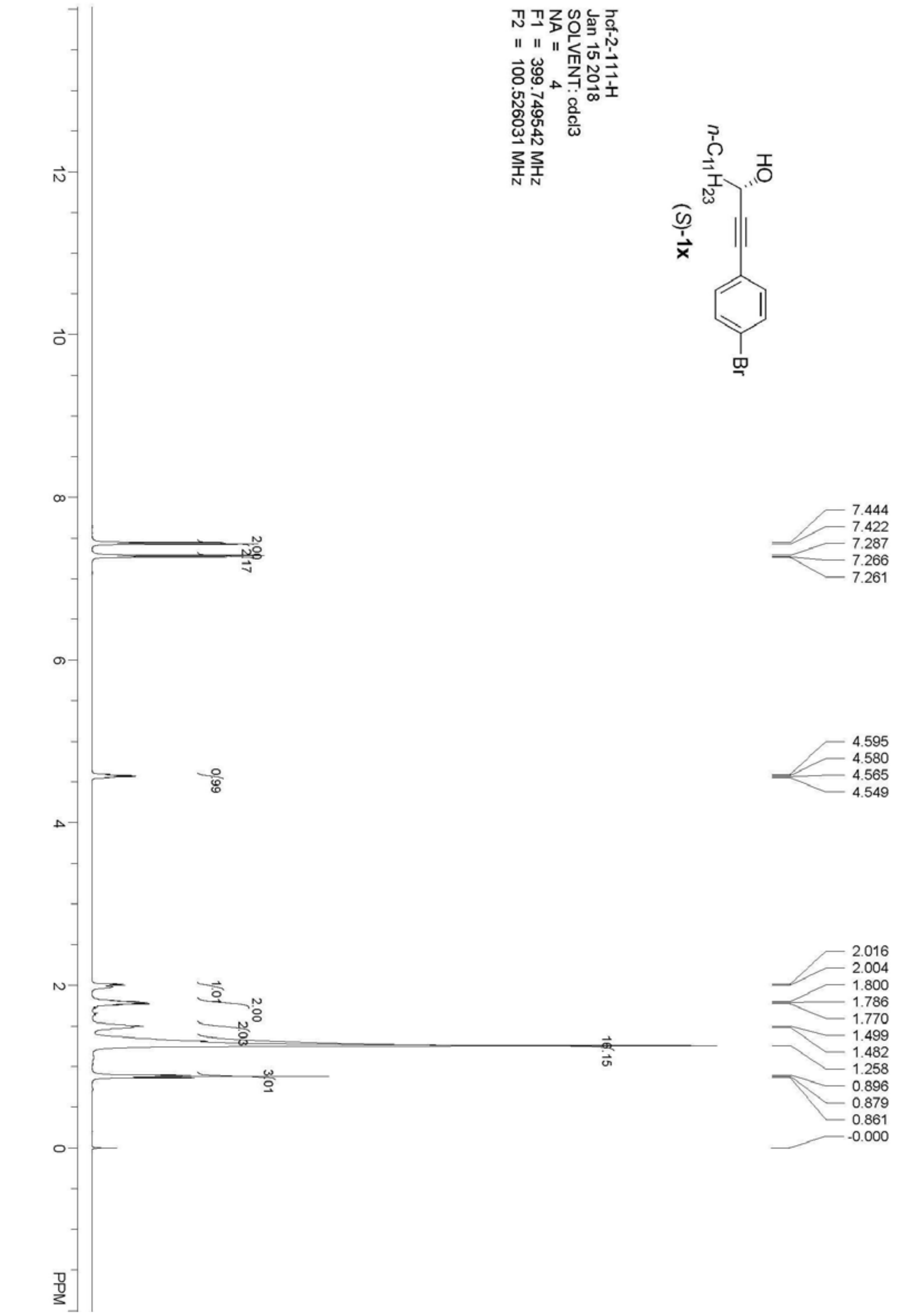
hcf-2-111-H
Jan 15 2018
SOLVENT: cdcl3
NA = 4
F1 = 399.749542 MHz
F2 = 100.526031 MHz
HO
n-C11H23
(S)-1x
Br
7.444
7.422
7.287
7.266
7.261
4.595
4.580
4.565
4.549
2.016
2.004
1.800
1.786
1.770
1.499
1.482
1.258
0.896
0.879
0.861
-0.000
2.00
2.17
0.99
1.01
2.00
2.03
16.15
3.01
PPM

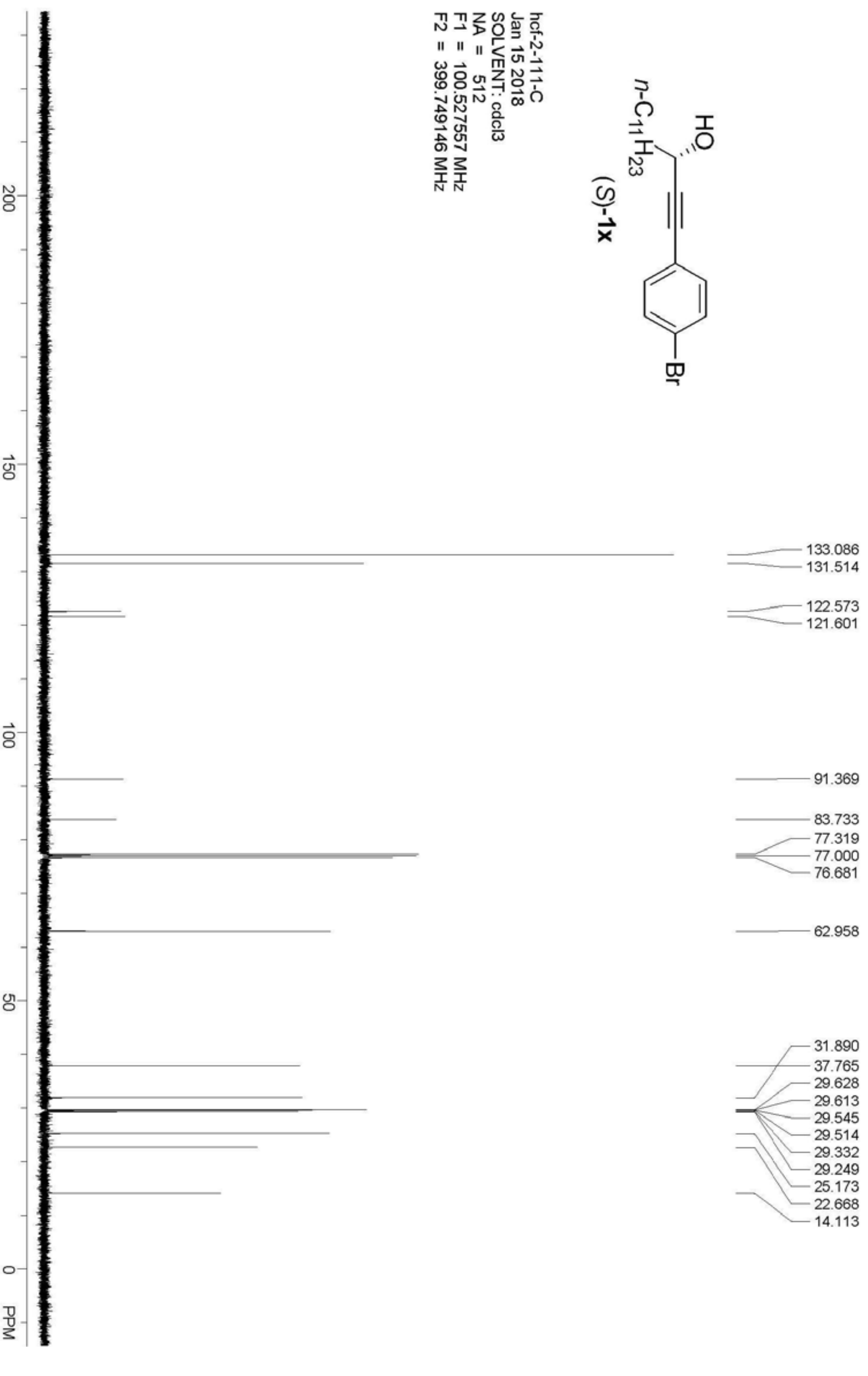
hcf-2-111-C
Jan 15 2018
SOLVENT: cdcl3
NA = 512
F1 = 100.527557 MHz
F2 = 399.749146 MHz
HO
n-C11H23
Br
(S)-1x
133.086
131.514
122.573
121.601
91.369
83.733
77.319
77.000
76.681
62.958
31.890
37.765
29.628
29.613
29.545
29.514
29.332
29.249
25.173
22.668
14.113
200
150
100
50
0
PPM

hcf-2-109-pa-2-100-1-0.7-214

实验时间：2018-01-31, 15:44:41　　　　报告时间：2018-01-31, 16:49:52
谱图文件:D:\zhuguangjiong\hcf\20180131\hcf-2-109-pa-2-100-1-0.7-214.org

实验内容简介：

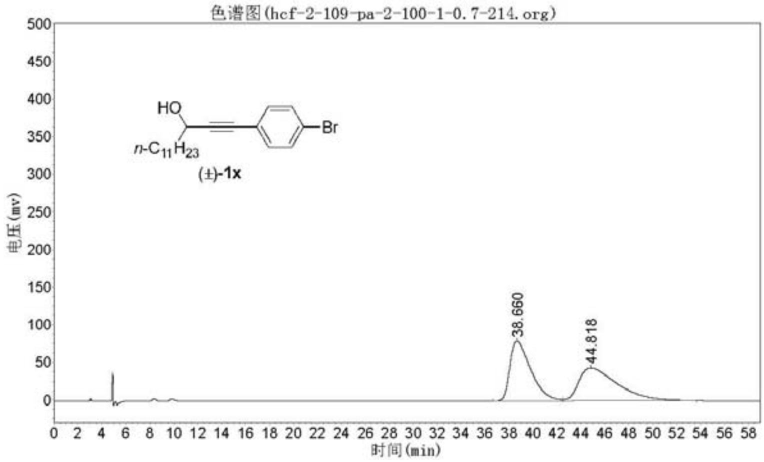

分析结果表

峰号	峰名	保留时间	峰高	峰面积	含量
1		38.660	79432.055	9596410.000	49.7081
2		44.818	43692.844	9709120.000	50.2919
**总计**			123124.898	19305530.000	100.0000

hcf-2-111-pa-2-100-1-0.7-214

实验时间：2018-01-31, 14:40:55　　　　报告时间：2018-01-31, 16:51:26
谱图文件:D:\zhuguangjiong\hcf\20180131\hcf-2-111-pa-2-100-1-0.7-214.org

实验内容简介：

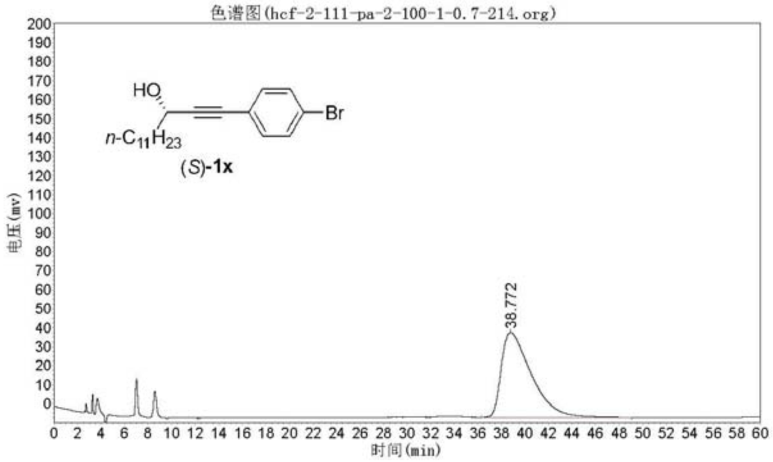

分析结果表

峰号	峰名	保留时间	峰高	峰面积	含量
1		38.772	44773.922	7916097.000	100.0000
总计			44773.922	7916097.000	100.0000

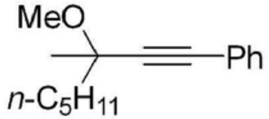

3

hcf-2-148-H
Mar 7 2018
SOLVENT: cdcl3
NA = 4
F1 = 399.749542 MHz
F2 = 100.526031 MHz

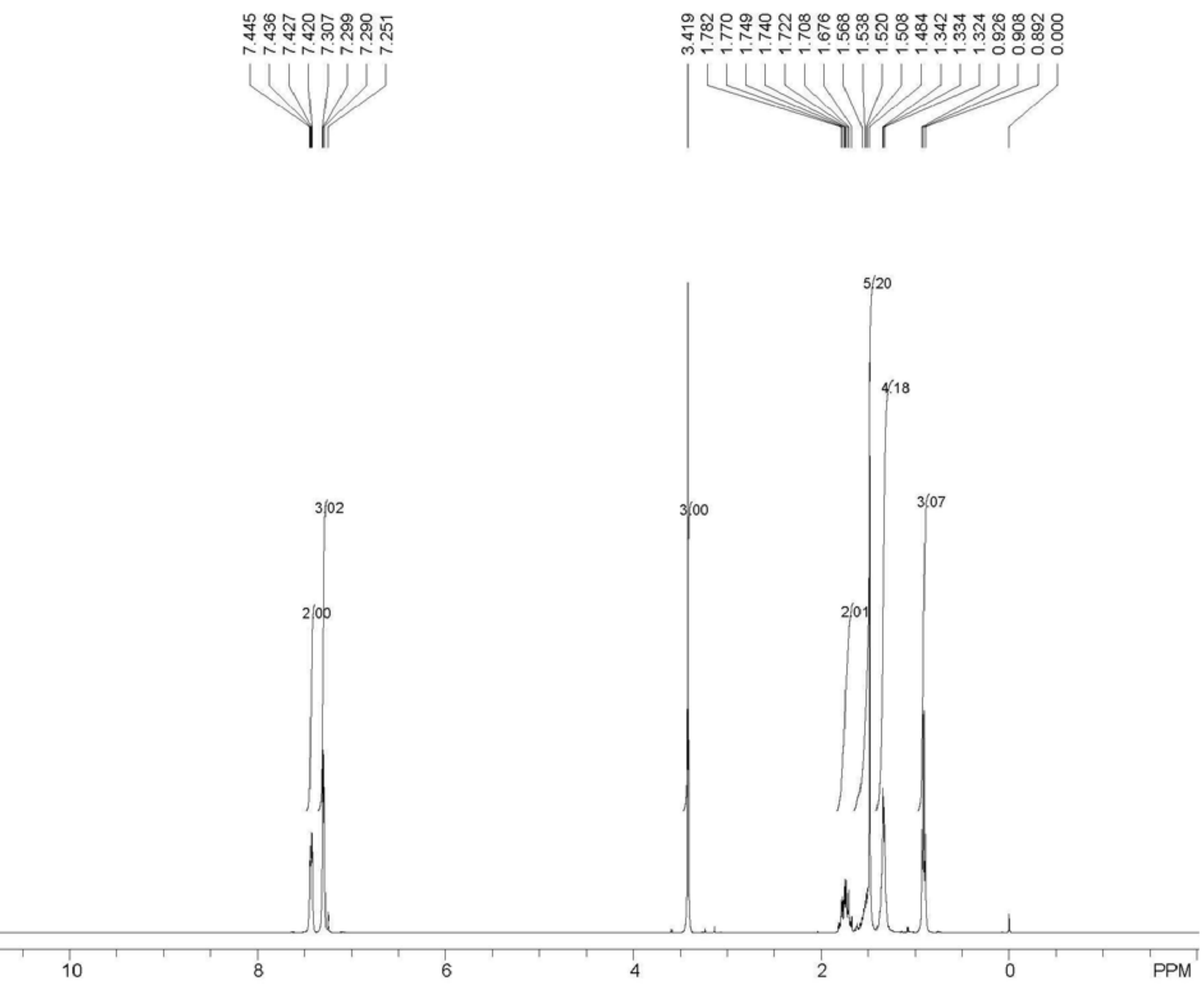

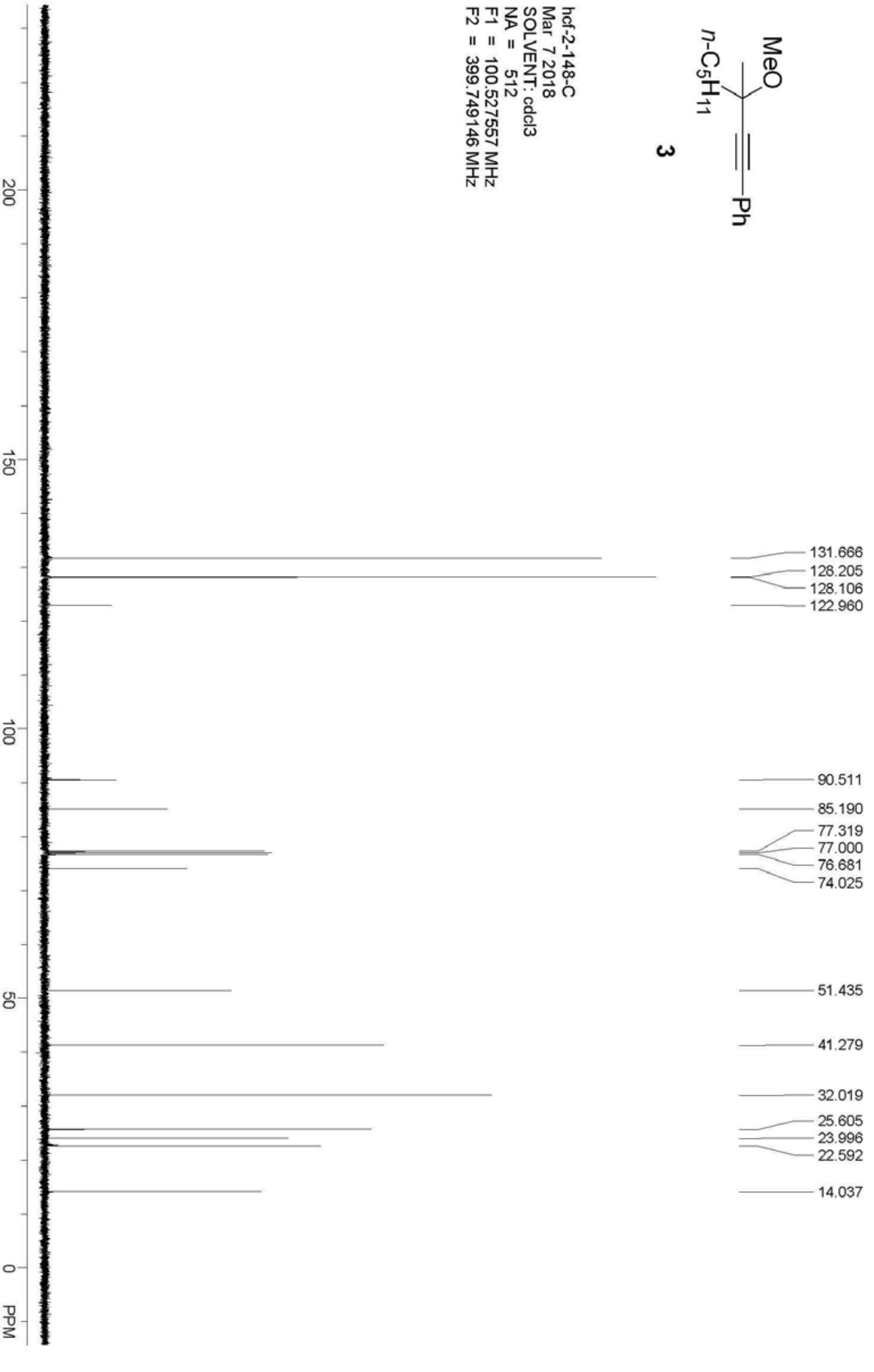
MeO
n-C5H11
Ph
3
hcf-2-148-C
Mar 7 2018
SOLVENT: cdcl3
NA = 512
F1 = 100.527557 MHz
F2 = 399.749146 MHz
131.666
128.205
128.106
122.960
90.511
85.190
77.319
77.000
76.681
74.025
51.435
41.279
32.019
25.605
23.996
22.592
14.037
200
150
100
50
0
PPM

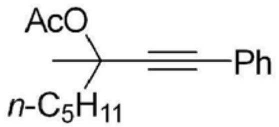

4

hcf-2-146-H
Mar 7 2018
SOLVENT: cdcl3
NA = 4
F1 = 399.749542 MHz
F2 = 100.526031 MHz

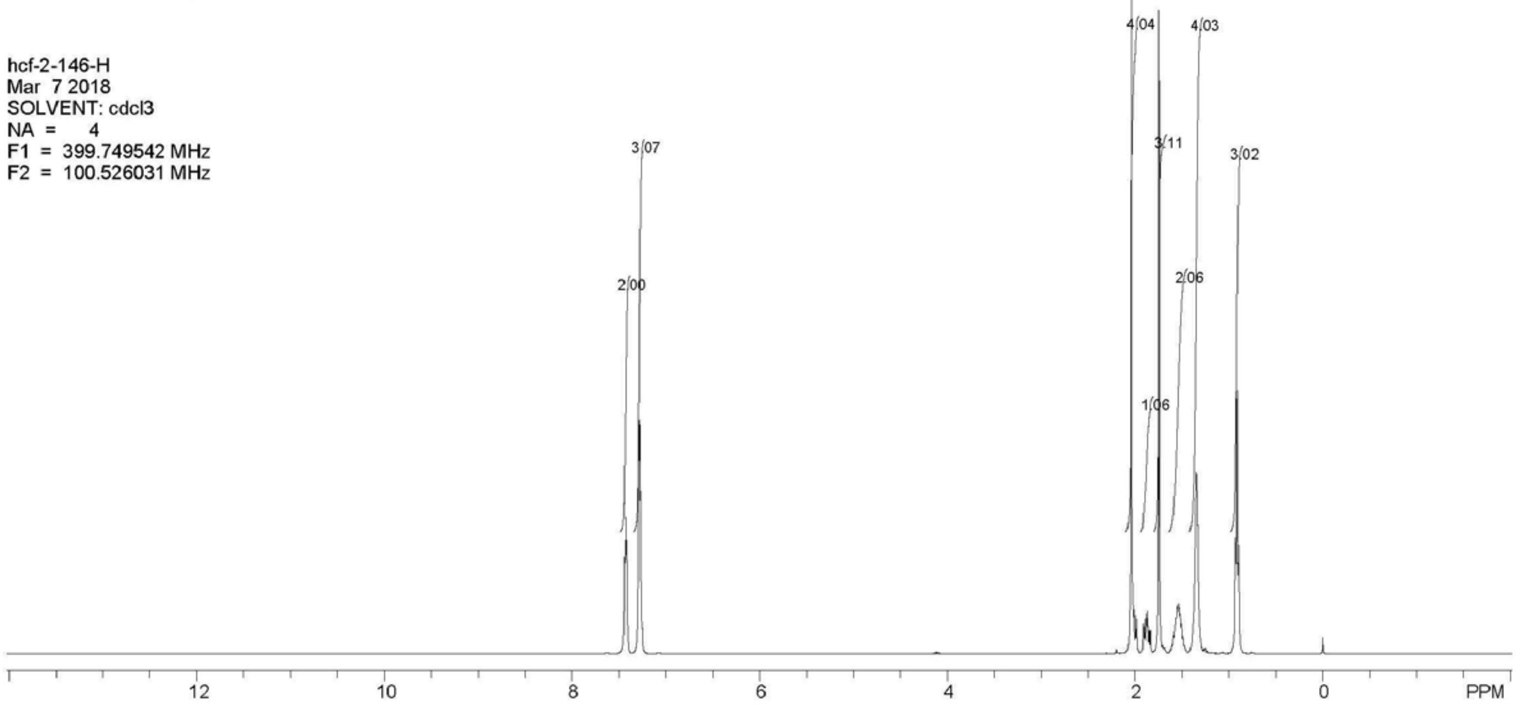

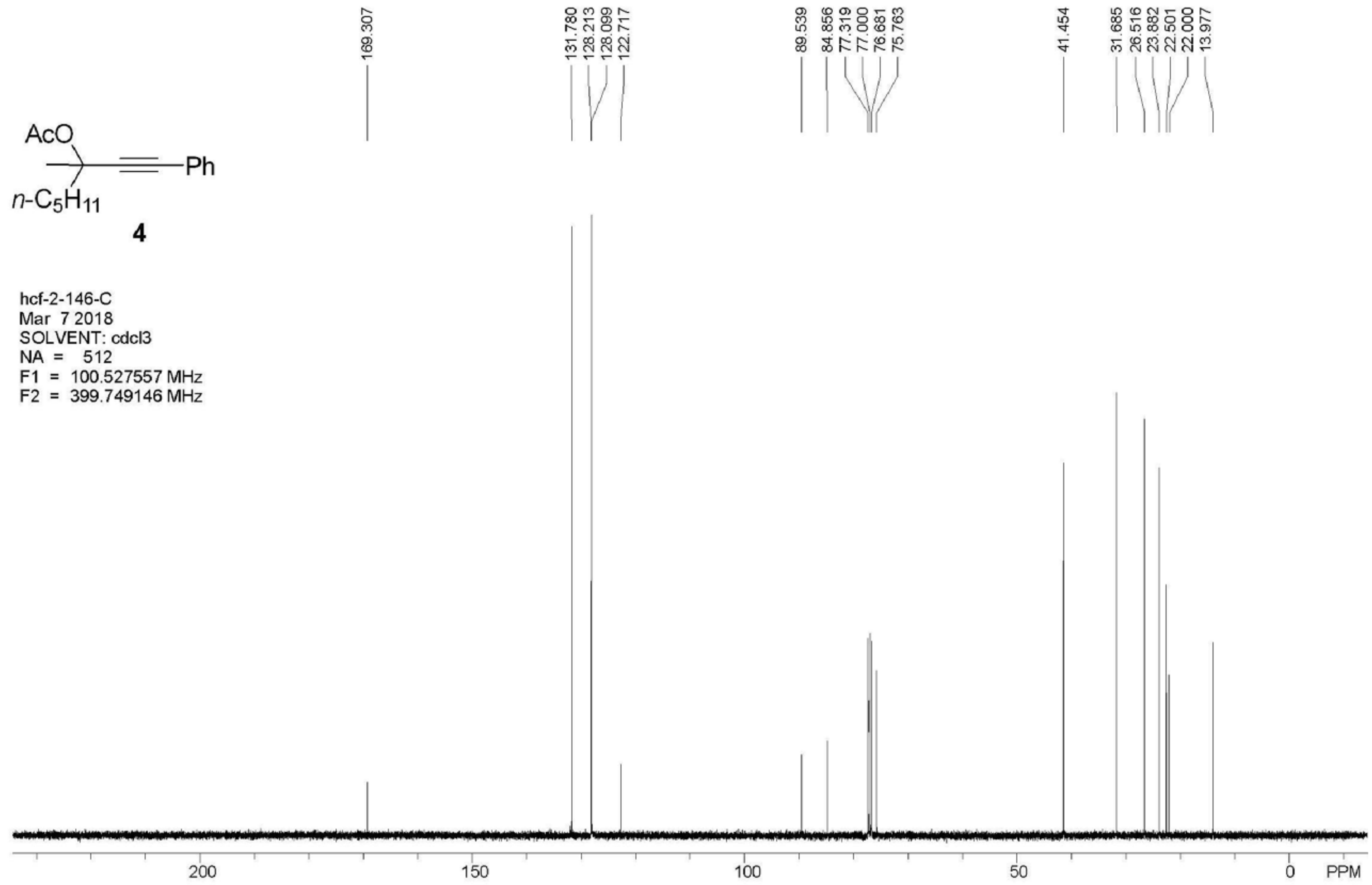

4

hcf-2-146-C
Mar 7 2018
SOLVENT: cdcl3
NA = 512
F1 = 100.527557 MHz
F2 = 399.749146 MHz

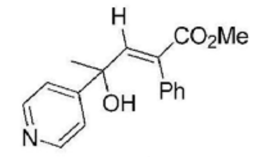
H
CO_2Me
OH
Ph
N

(*E*)-**2a**

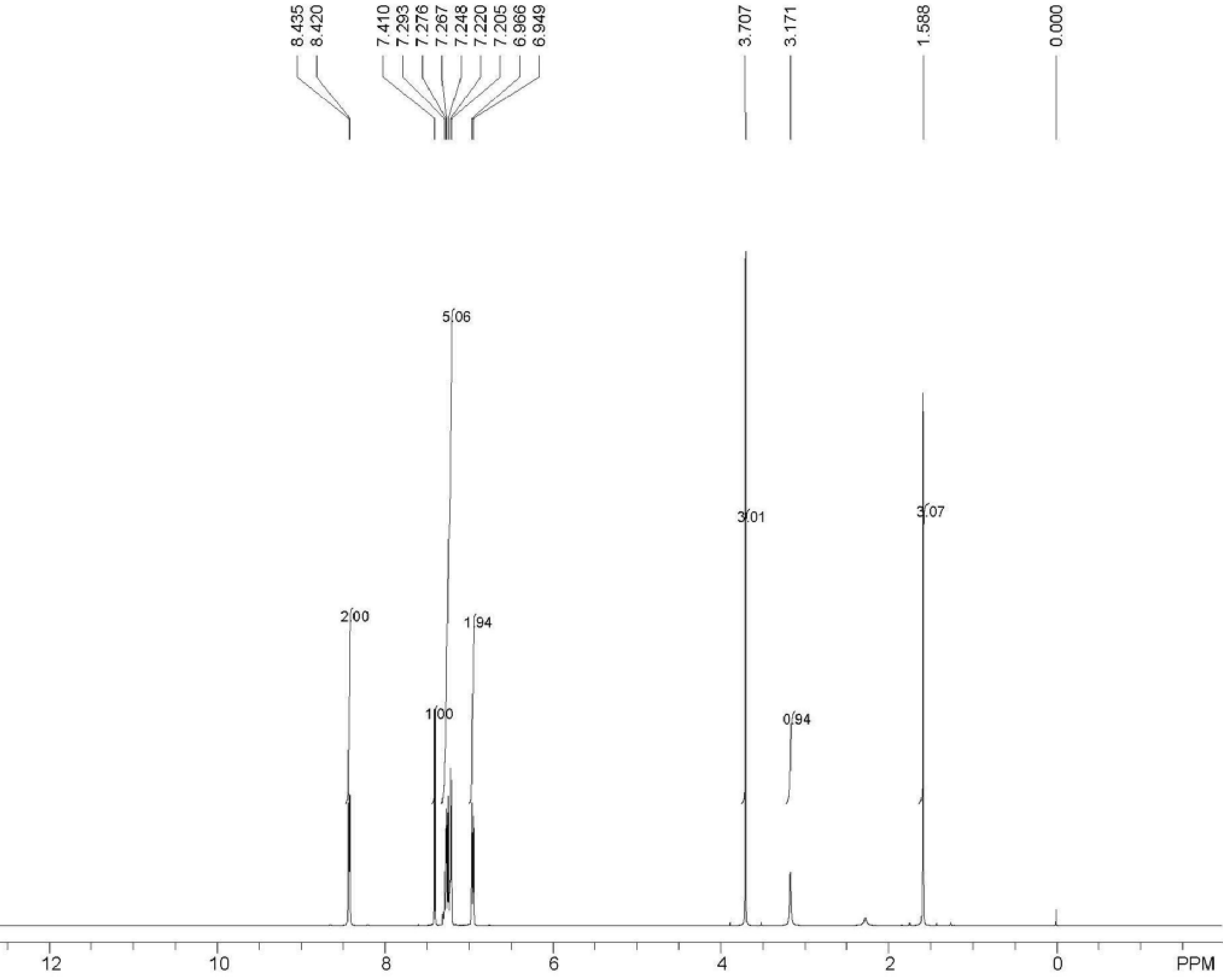
hcf-1-197-H
Oct 2 2017
SOLVENT: CDCl3
NA = 24
F1 = 399.722809 MHz
F2 = 100.519203 MHz
8.435
8.420
7.410
7.293
7.276
7.267
7.248
7.220
7.205
6.966
6.949
3.707
3.171
1.588
0.000
2.00
1.00
5.06
1.94
3.01
0.94
3.07
14
12
10
8
6
4
2
0
PPM

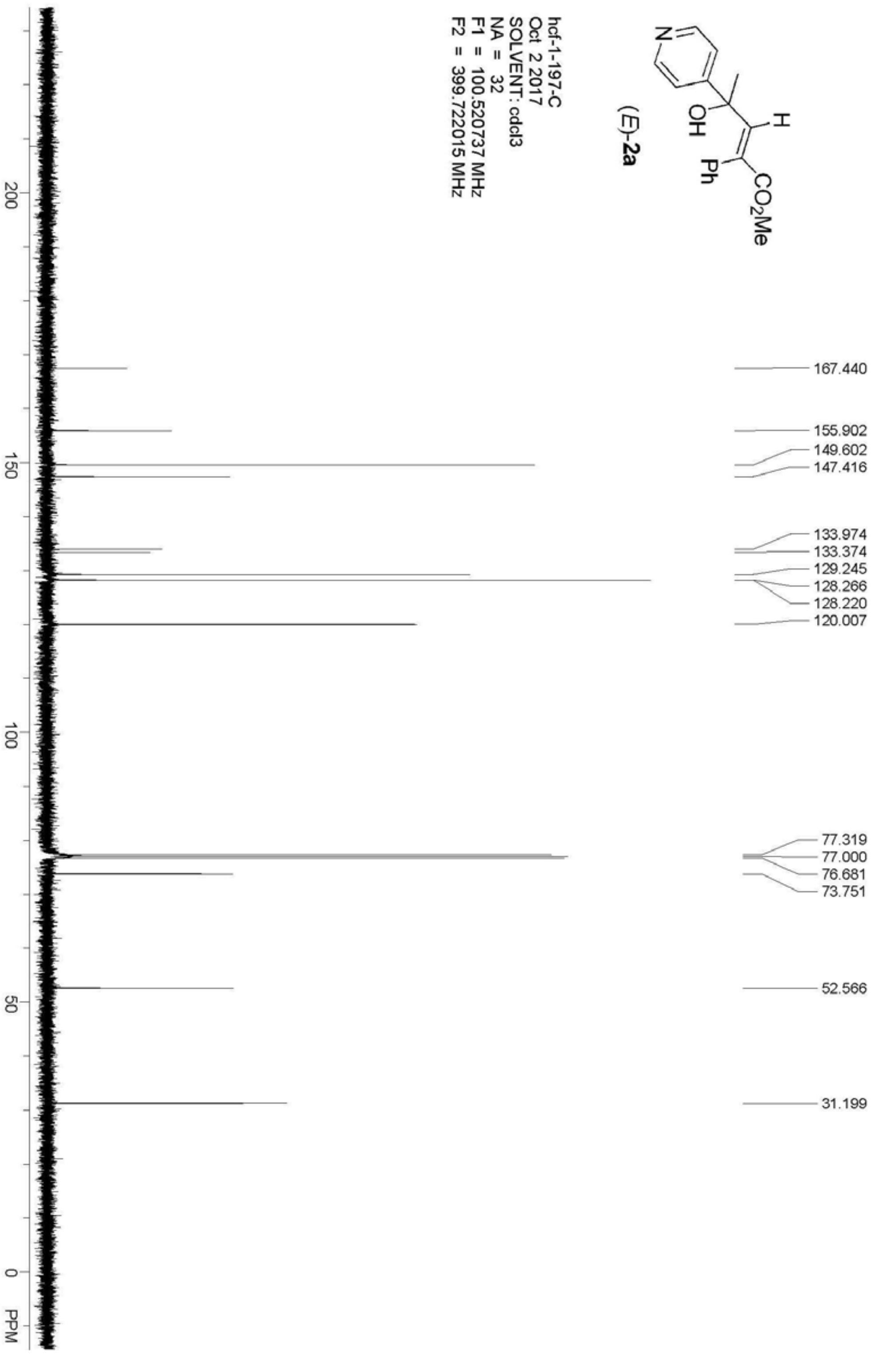
(E)-2a
CO2Me
Ph
OH
H
N
hcf-1-197-C
Oct 2 2017
SOLVENT: cdcl3
NA = 32
F1 = 100.520737 MHz
F2 = 399.722015 MHz
167.440
155.902
149.602
147.416
133.974
133.374
129.245
128.266
128.220
120.007
77.319
77.000
76.681
73.751
52.566
31.199
200
150
100
50
0
PPM

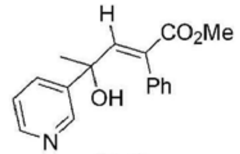
H
CO_2Me
OH
Ph
N

(*E*)-**2b**

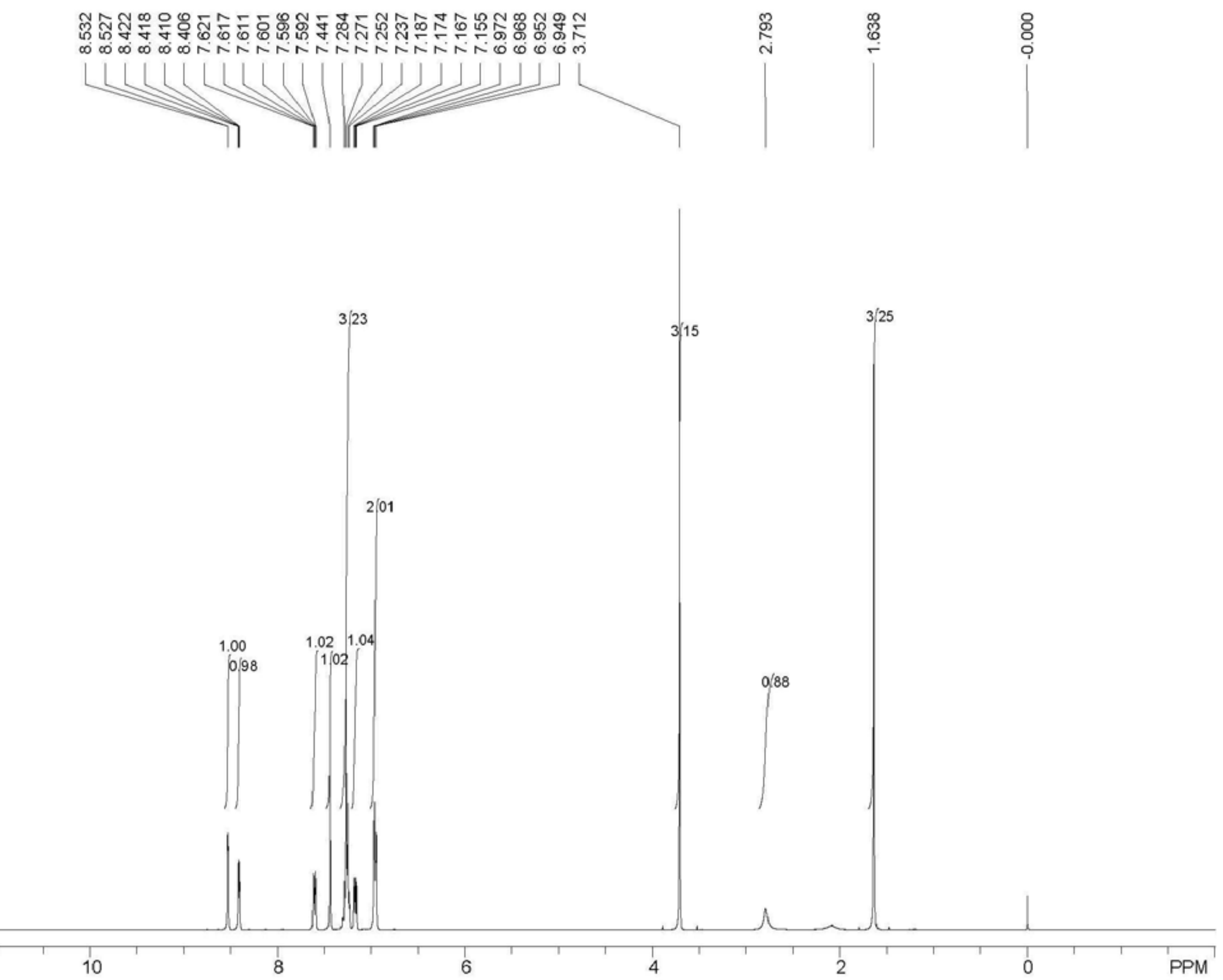
hcf-1-198-H
Sep 22 2017
SOLVENT: cdcl3
NA = 8
F1 = 399.749542 MHz
F2 = 100.526031 MHz
8.532
8.527
8.422
8.418
8.410
8.406
7.621
7.617
7.611
7.601
7.596
7.592
7.441
7.284
7.271
7.252
7.237
7.187
7.174
7.167
7.155
6.972
6.968
6.952
6.949
3.712
2.793
1.638
-0.000
1.00
0.98
1.02
1.02
1.04
3.23
2.01
3.15
0.88
3.25
12
10
8
6
4
2
0
PPM

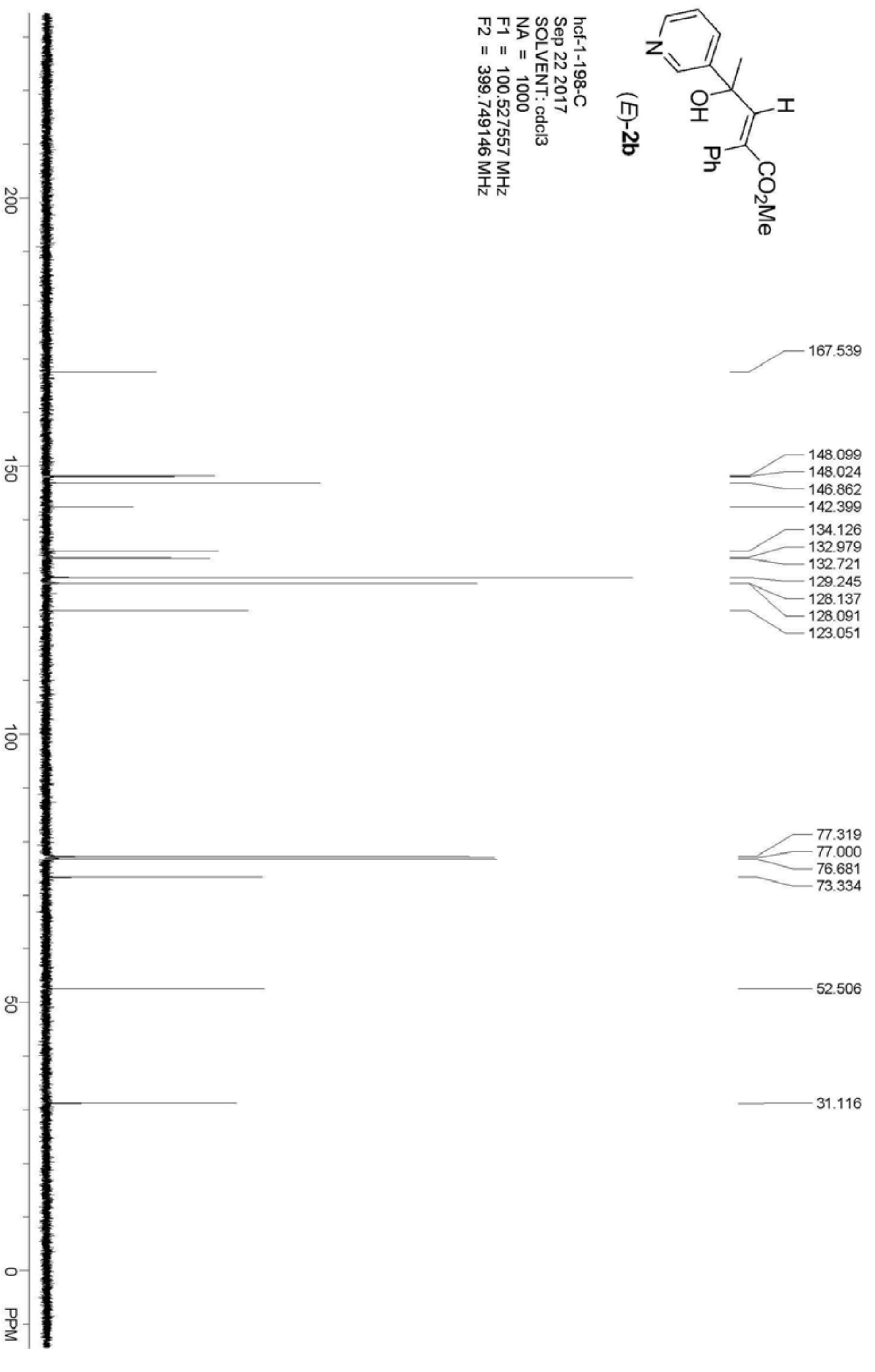

(E)-2b
hcf-1-198-C
Sep 22 2017
SOLVENT: cdcl3
NA = 1000
F1 = 100.527557 MHz
F2 = 399.749146 MHz
167.539
148.099
148.024
146.862
142.399
134.126
132.979
132.721
129.245
128.137
128.091
123.051
77.319
77.000
76.681
73.334
52.506
31.116
PPM

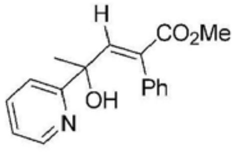

(*E*)-**2c**

hcf-2-2-H
Oct 18 2017
SOLVENT: cdcl3
NA = 8
F1 = 399.749542 MHz
F2 = 100.526031 MHz

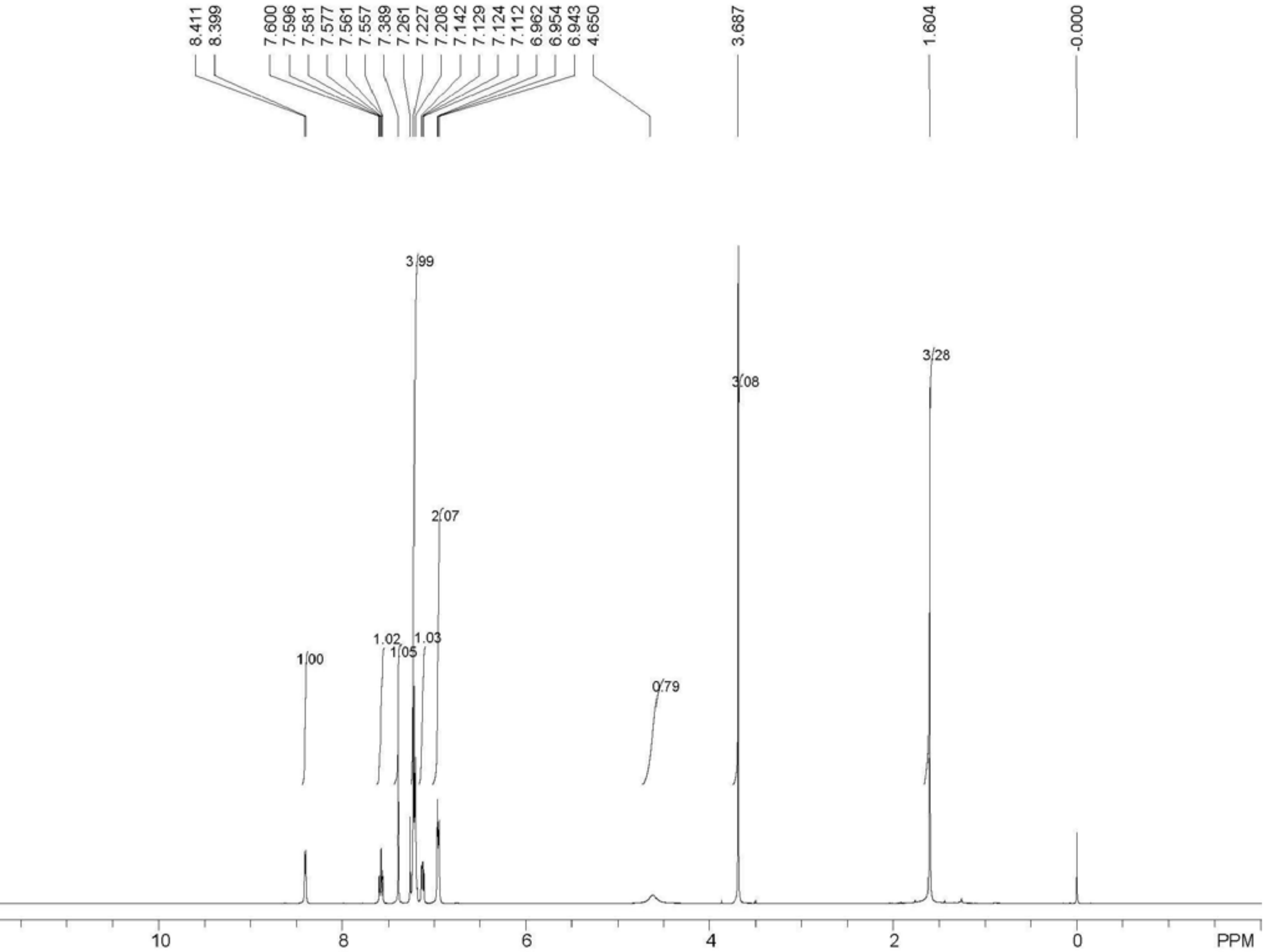

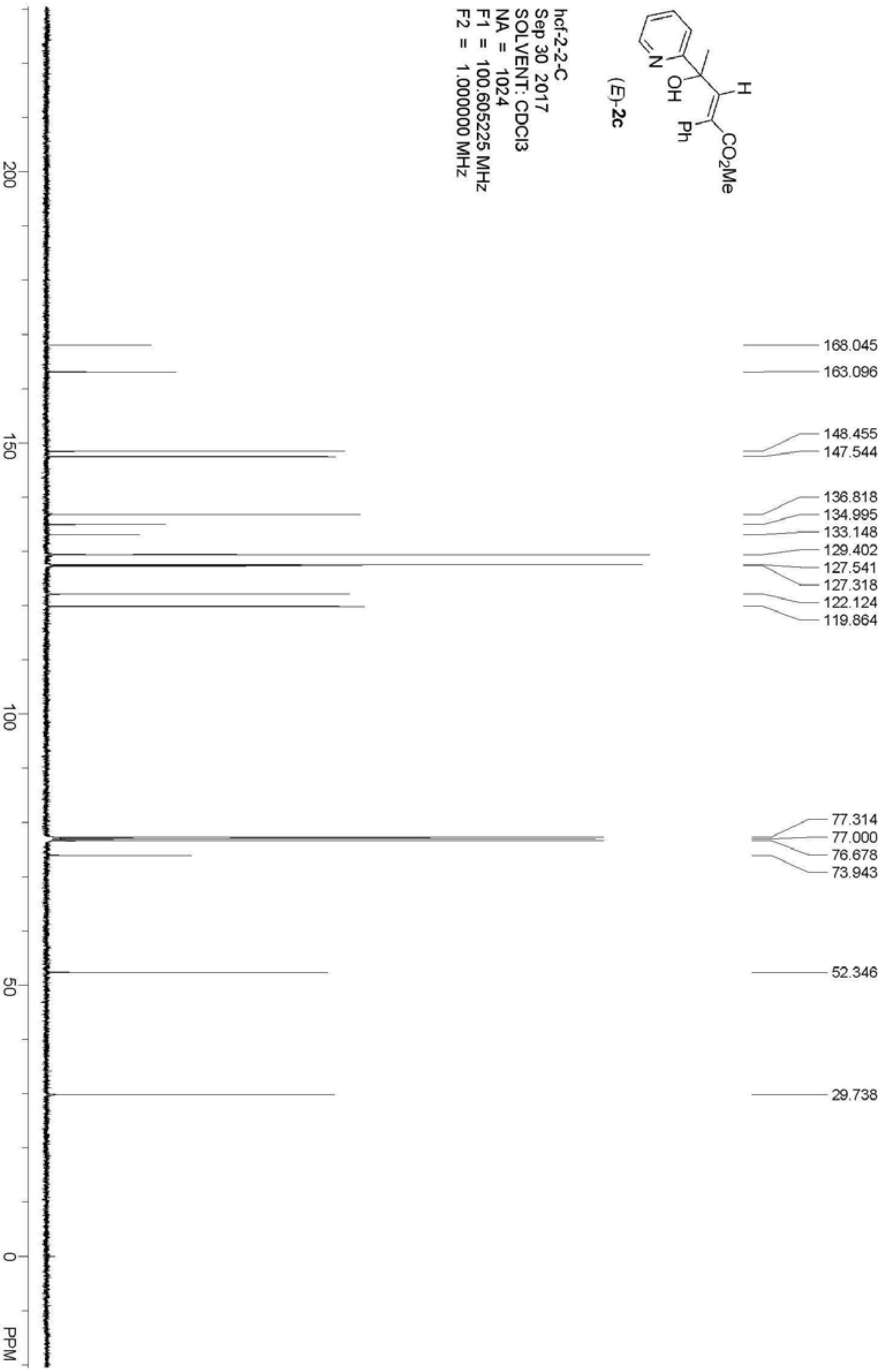

(E)-2c
hcf-2-2-C
Sep 30 2017
SOLVENT: CDCl3
NA = 1024
F1 = 100.605225 MHz
F2 = 1.000000 MHz
168.045
163.096
148.455
147.544
136.818
134.995
133.148
129.402
127.541
127.318
122.124
119.864
77.314
77.000
76.678
73.943
52.346
29.738
200
150
100
50
0
PPM

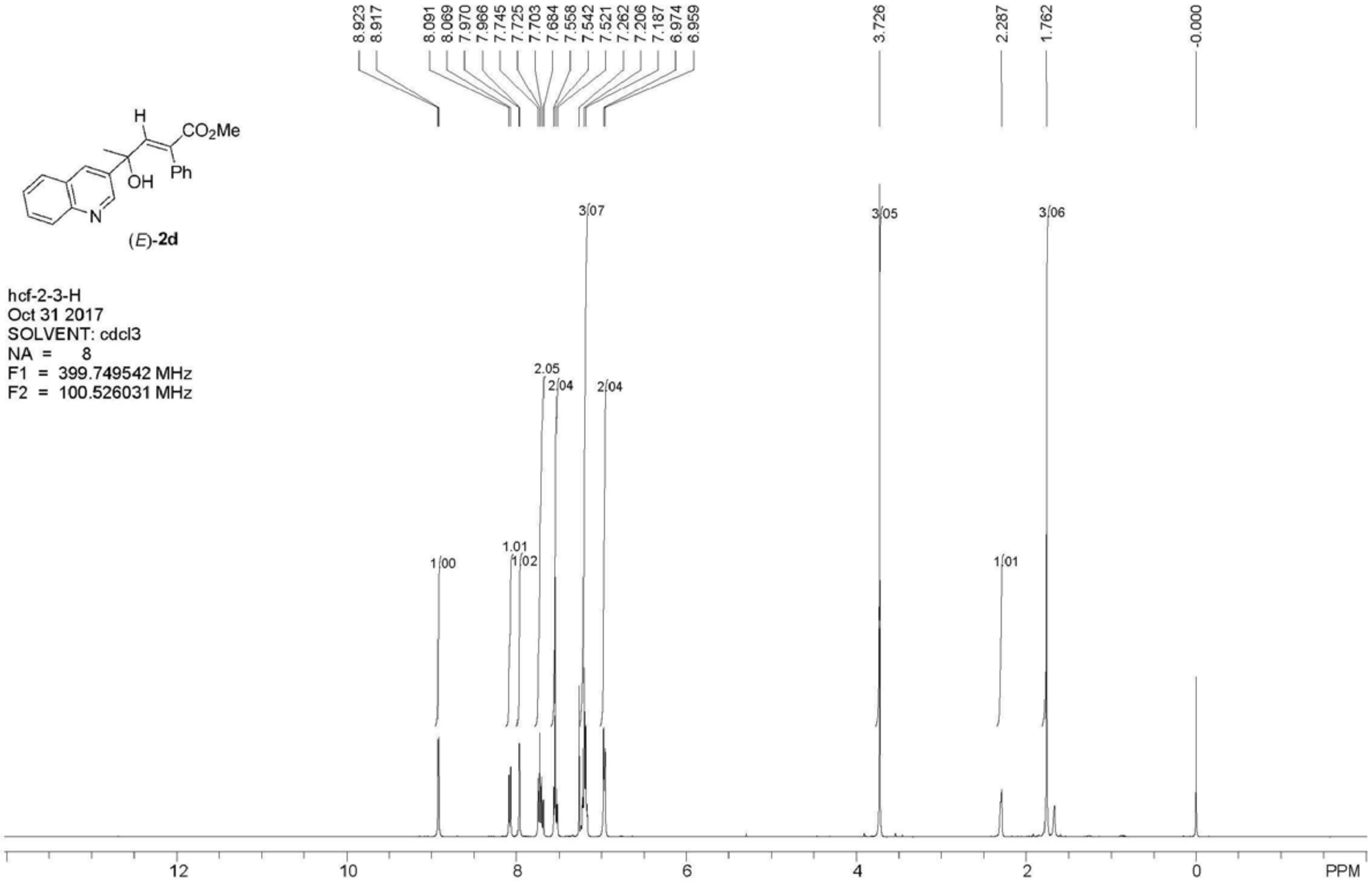

(*E*)-**2d**

hcf-2-3-H
Oct 31 2017
SOLVENT: cdcl3
NA = 8
F1 = 399.749542 MHz
F2 = 100.526031 MHz

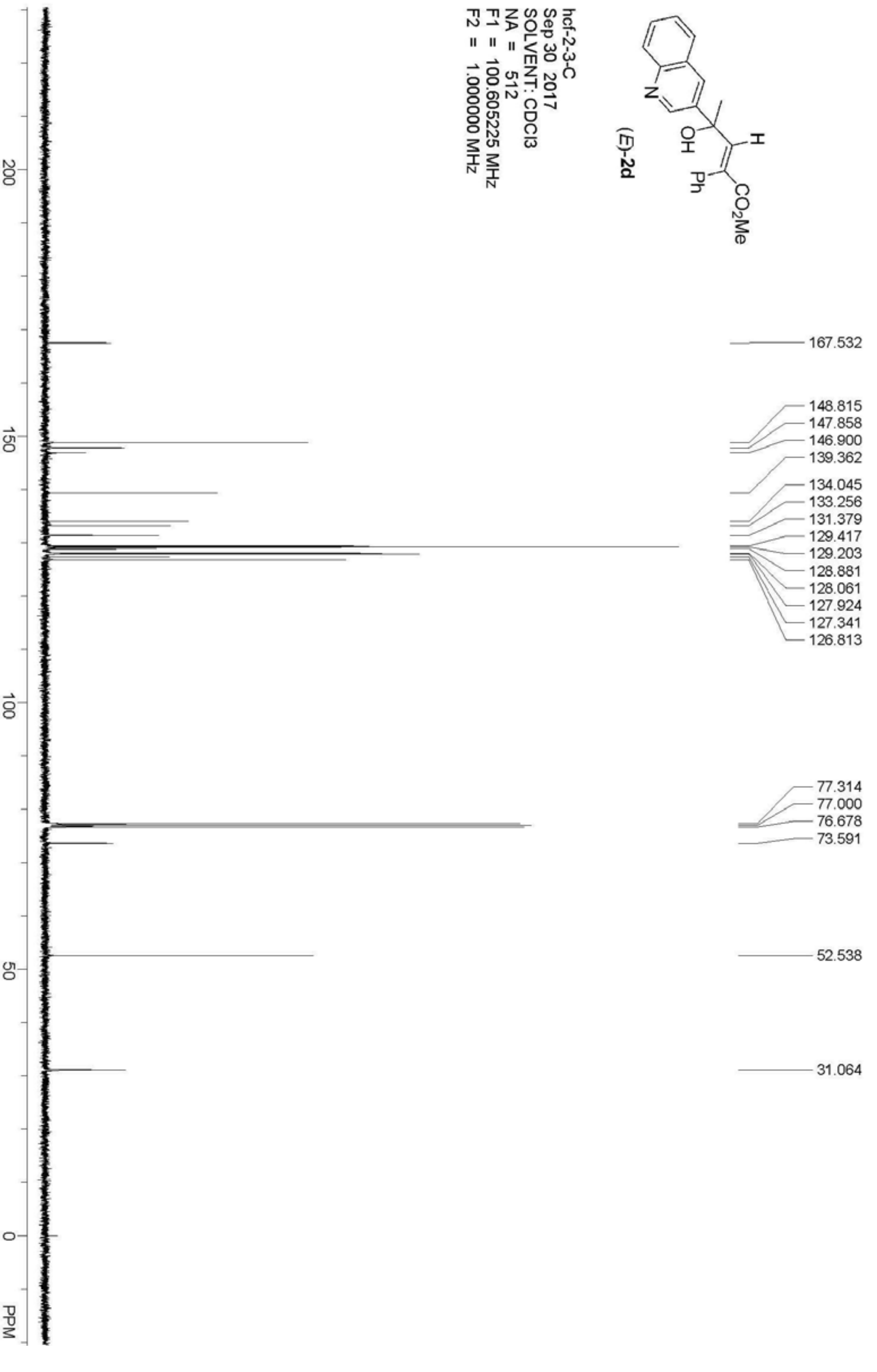
hcf-2-3-C
Sep 30 2017
SOLVENT: CDCl3
NA = 512
F1 = 100.605225 MHz
F2 = 1.000000 MHz
(E)-2d
OH
Ph
H
CO2Me
N
167.532
148.815
147.858
146.900
139.362
134.045
133.256
131.379
129.417
129.203
128.881
128.061
127.924
127.341
126.813
77.314
77.000
76.678
73.591
52.538
31.064
200
150
100
50
0
PPM

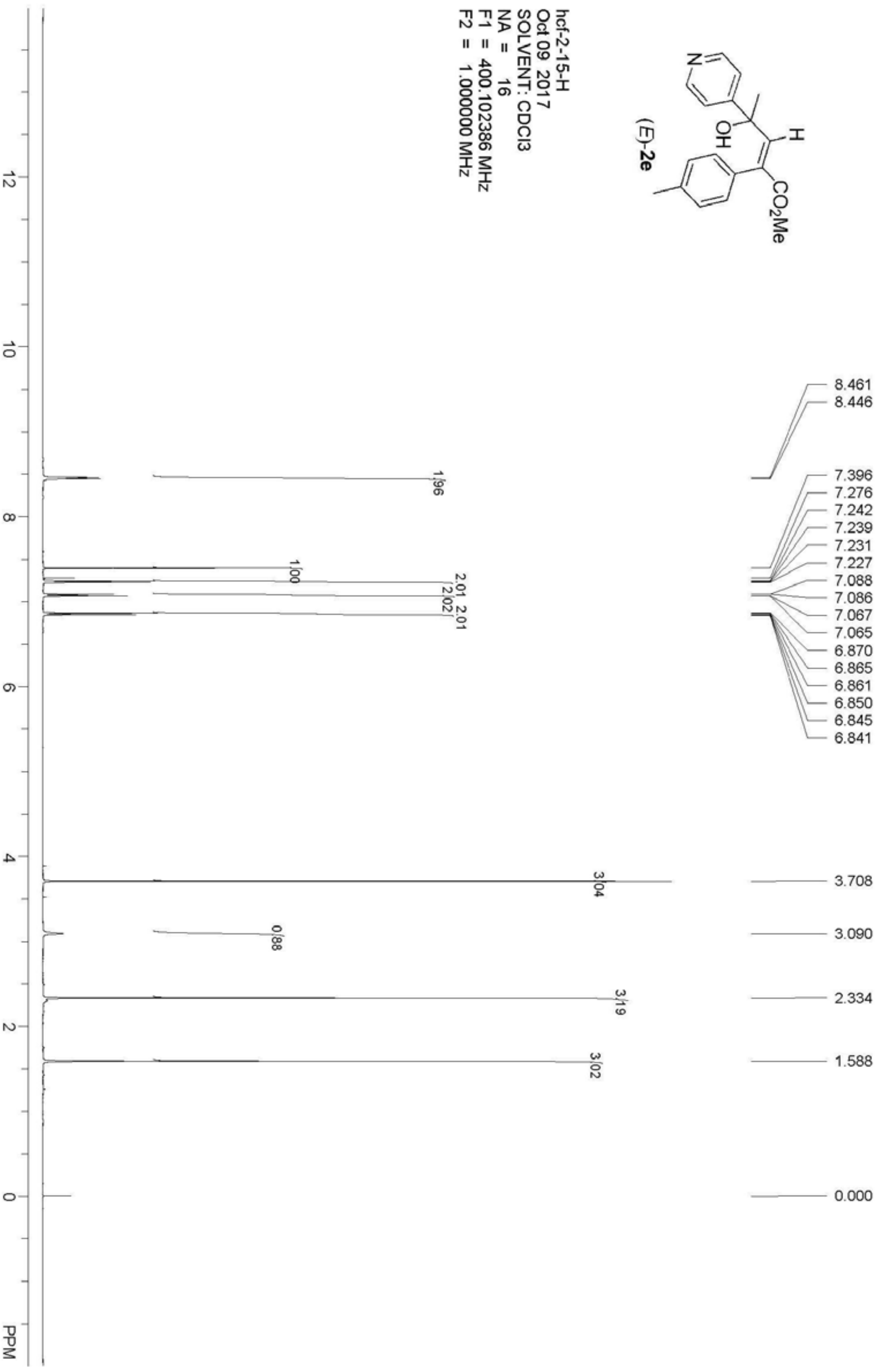
(E)-2e
CO2Me
OH
H
N
hcf-2-15-H
Oct 09 2017
SOLVENT: CDCl3
NA = 16
F1 = 400.102386 MHz
F2 = 1.000000 MHz
8.461
8.446
7.396
7.276
7.242
7.239
7.231
7.227
7.088
7.086
7.067
7.065
6.870
6.865
6.861
6.850
6.845
6.841
3.708
3.090
2.334
1.588
0.000
1.96
1.00
2.01
2.02
2.01
3.04
0.88
3.19
3.02
12
10
8
6
4
2
0
PPM

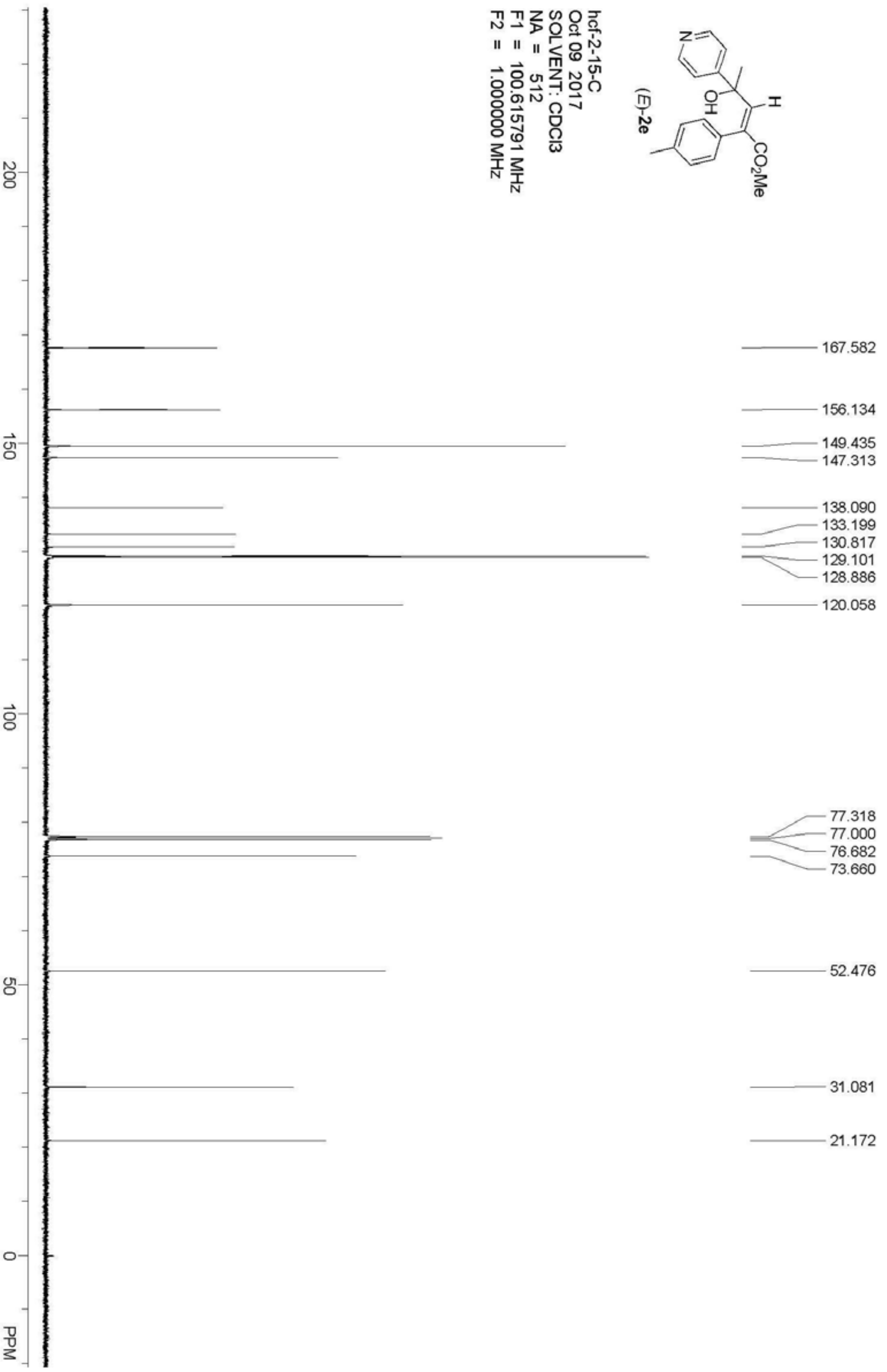
hcf-2-15-C
Oct 09 2017
SOLVENT: CDCl3
NA = 512
F1 = 100.615791 MHz
F2 = 1.000000 MHz
(E)-2e
167.582
156.134
149.435
147.313
138.090
133.199
130.817
129.101
128.886
120.058
77.318
77.000
76.682
73.660
52.476
31.081
21.172
200
150
100
50
0
PPM

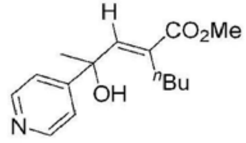

(*E*)-**2f**

hcf-2-4-H
Oct 31 2017
SOLVENT: cdcl3
NA = 8
F1 = 399.749542 MHz
F2 = 100.526031 MHz

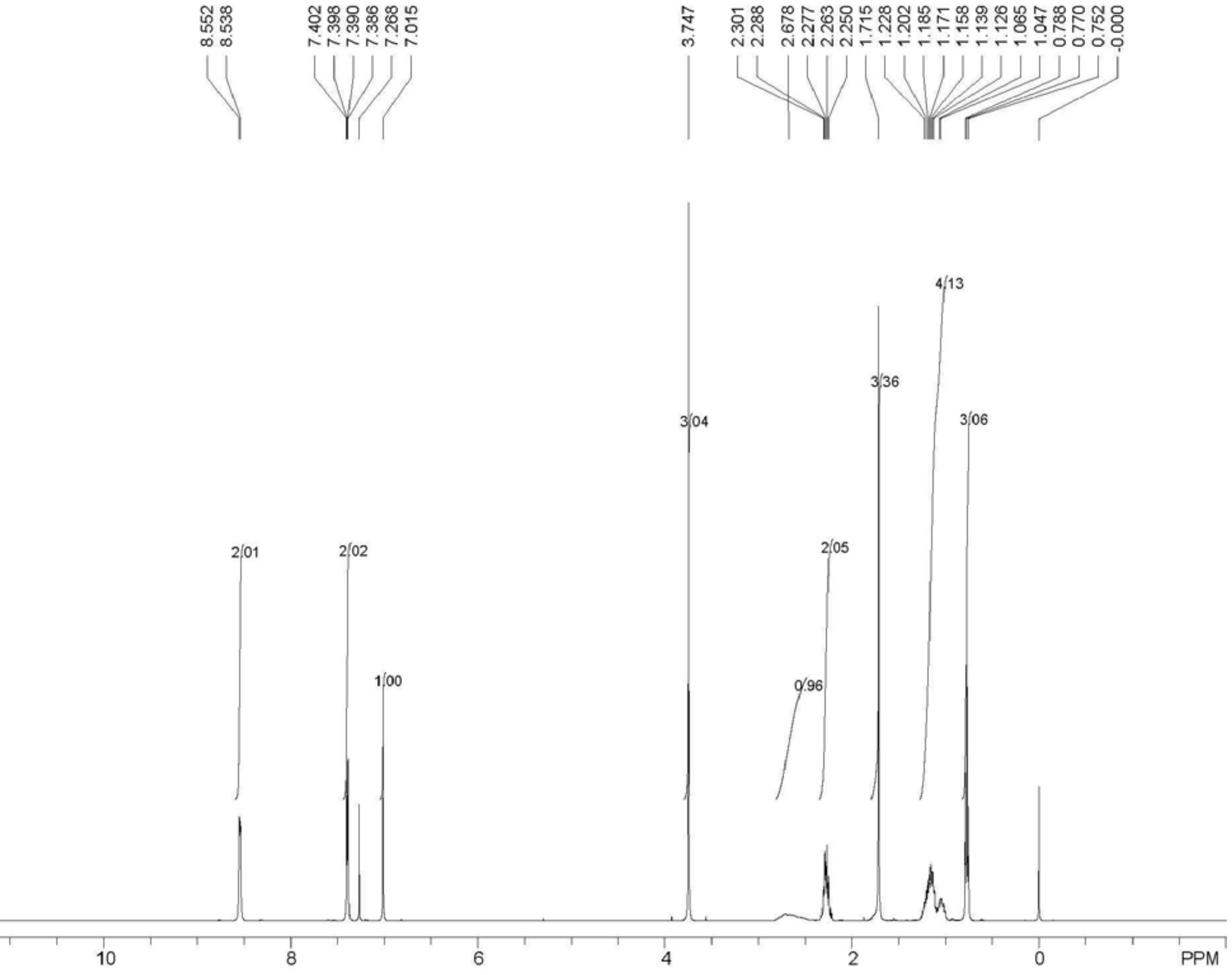

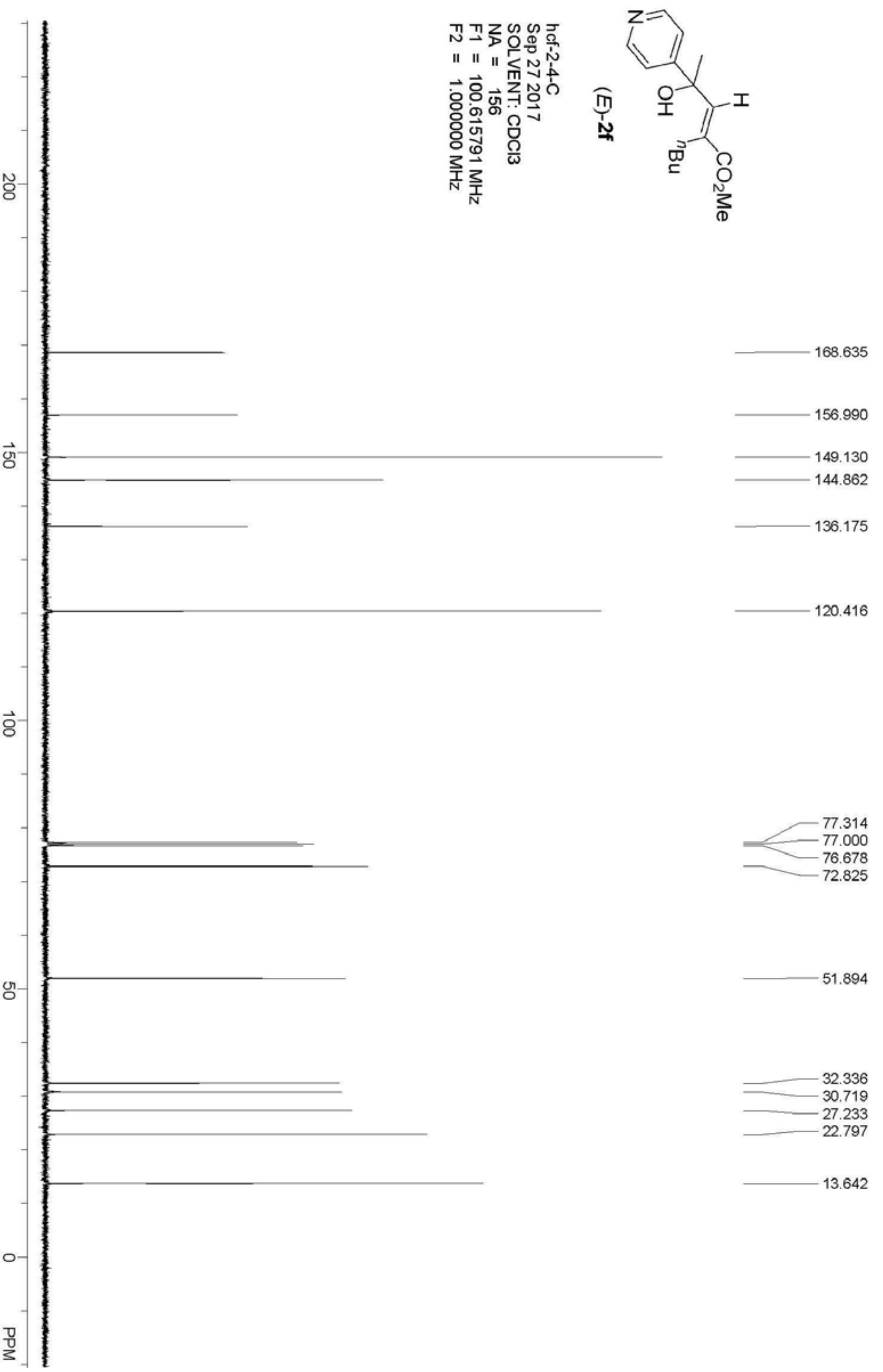

(E)-2f
hcf-2-4-C
Sep 27 2017
SOLVENT: CDCl3
NA = 156
F1 = 100.615791 MHz
F2 = 1.000000 MHz
168.635
156.990
149.130
144.862
136.175
120.416
77.314
77.000
76.678
72.825
51.894
32.336
30.719
27.233
22.797
13.642
200
150
100
50
0
PPM

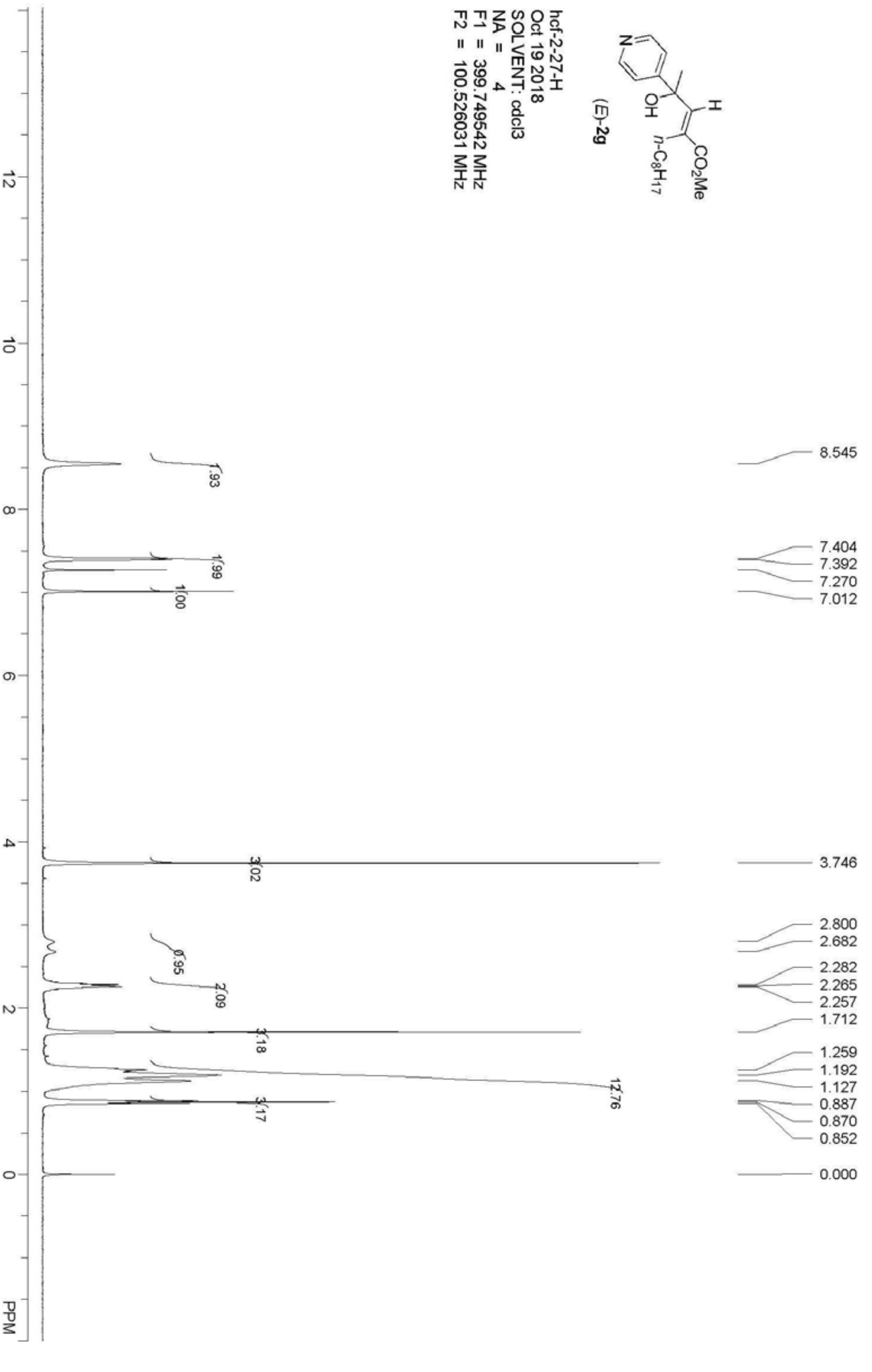
hcf-2-27-H
Oct 19 2018
SOLVENT: cdcl3
NA = 4
F1 = 399.749542 MHz
F2 = 100.526031 MHz
(E)-2g
8.545
7.404
7.392
7.270
7.012
3.746
2.800
2.682
2.282
2.265
2.257
1.712
1.259
1.192
1.127
0.887
0.870
0.852
0.000
PPM

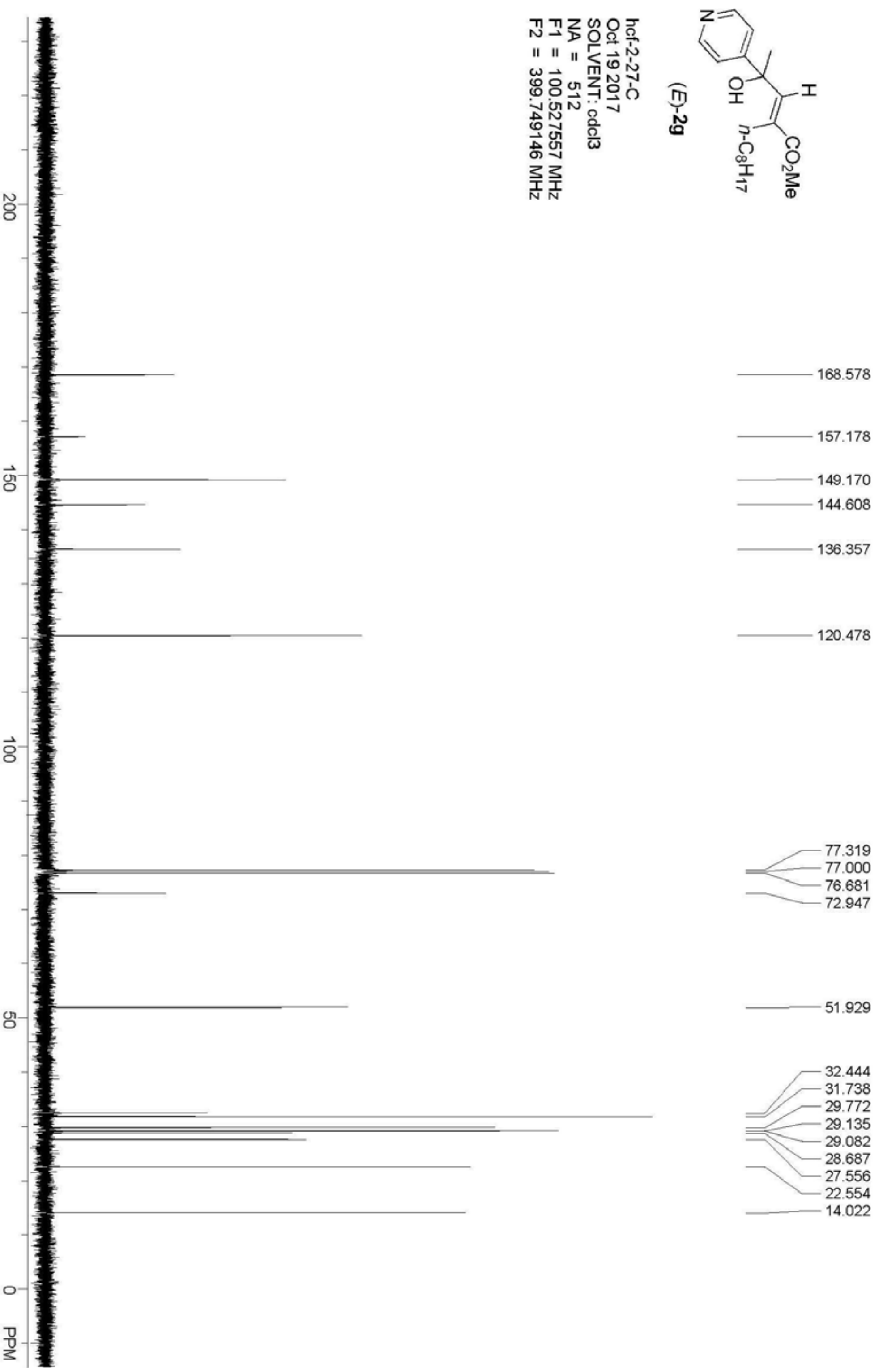
(E)-2g
hcf-2-27-C
Oct 19 2017
SOLVENT: cdcl3
NA = 512
F1 = 100.527557 MHz
F2 = 399.749146 MHz
168.578
157.178
149.170
144.608
136.357
120.478
77.319
77.000
76.681
72.947
51.929
32.444
31.738
29.772
29.135
29.082
28.687
27.556
22.554
14.022
200
150
100
50
0
PPM

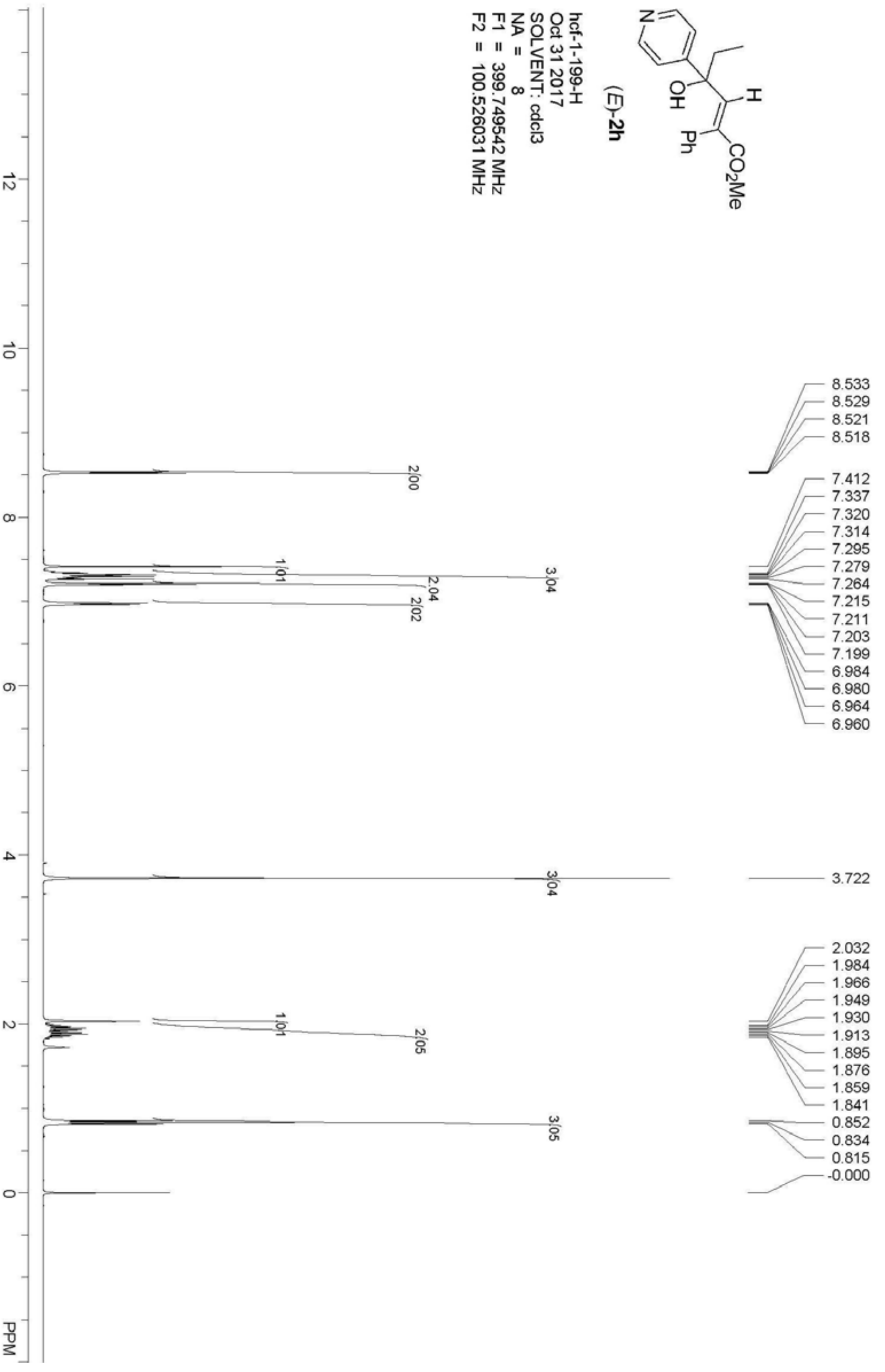
(E)-2h
H
CO2Me
Ph
OH
N
hcf-1-199-H
Oct 31 2017
SOLVENT: cdcl3
NA = 8
F1 = 399.749542 MHz
F2 = 100.526031 MHz
8.533
8.529
8.521
8.518
7.412
7.337
7.320
7.314
7.295
7.279
7.264
7.215
7.211
7.203
7.199
6.984
6.980
6.964
6.960
3.722
2.032
1.984
1.966
1.949
1.930
1.913
1.895
1.876
1.859
1.841
0.852
0.834
0.815
-0.000
2.00
1.01
3.04
2.04
2.02
3.04
1.01
2.05
3.05
12
10
8
6
4
2
0
PPM

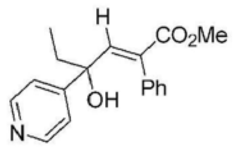
H
CO2Me
OH
Ph
N

(*E*)-**2h**

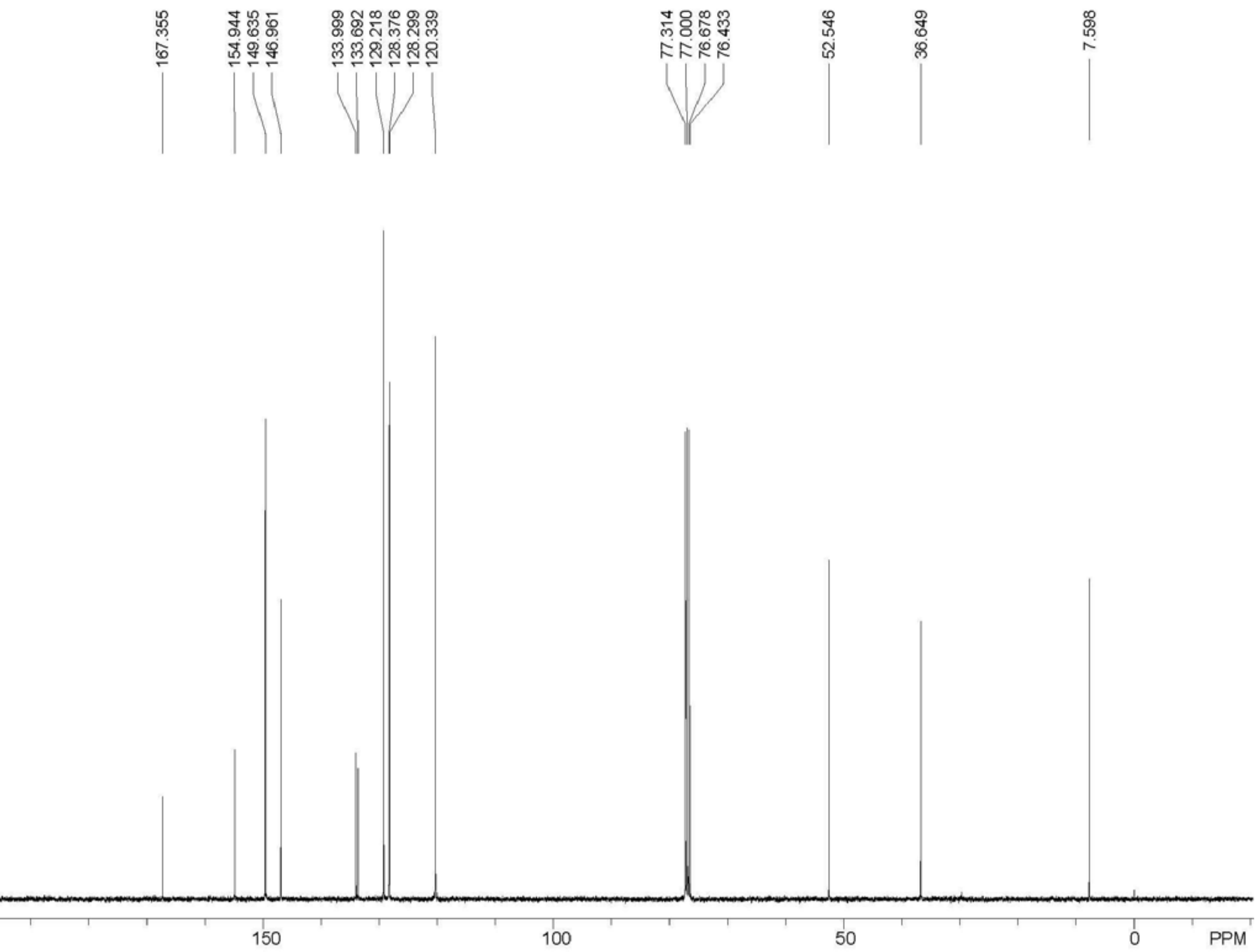
hcf-1-199-C
Sep 30 2017
SOLVENT: CDCl3
NA = 1024
F1 = 100.605225 MHz
F2 = 1.000000 MHz
167.355
154.944
149.635
146.961
133.999
133.692
129.218
128.376
128.299
120.339
77.314
77.000
76.678
76.433
52.546
36.649
7.598
200
150
100
50
0
PPM

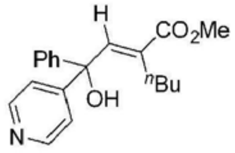

(*E*)-**2i**

hcf-2-70-H
Jan 8 2018
SOLVENT: cdcl3
NA = 4
F1 = 399.749542 MHz
F2 = 100.526031 MHz

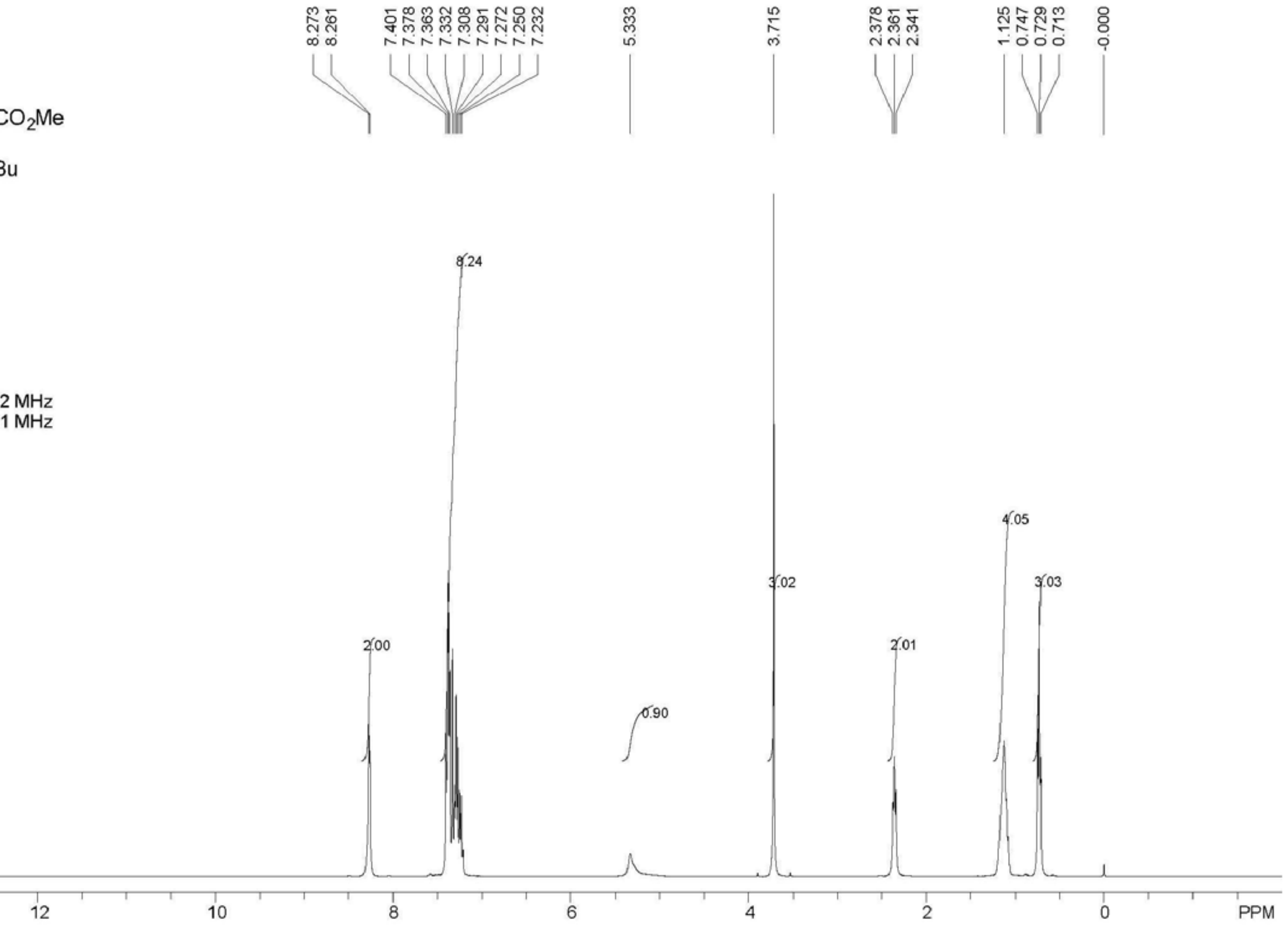

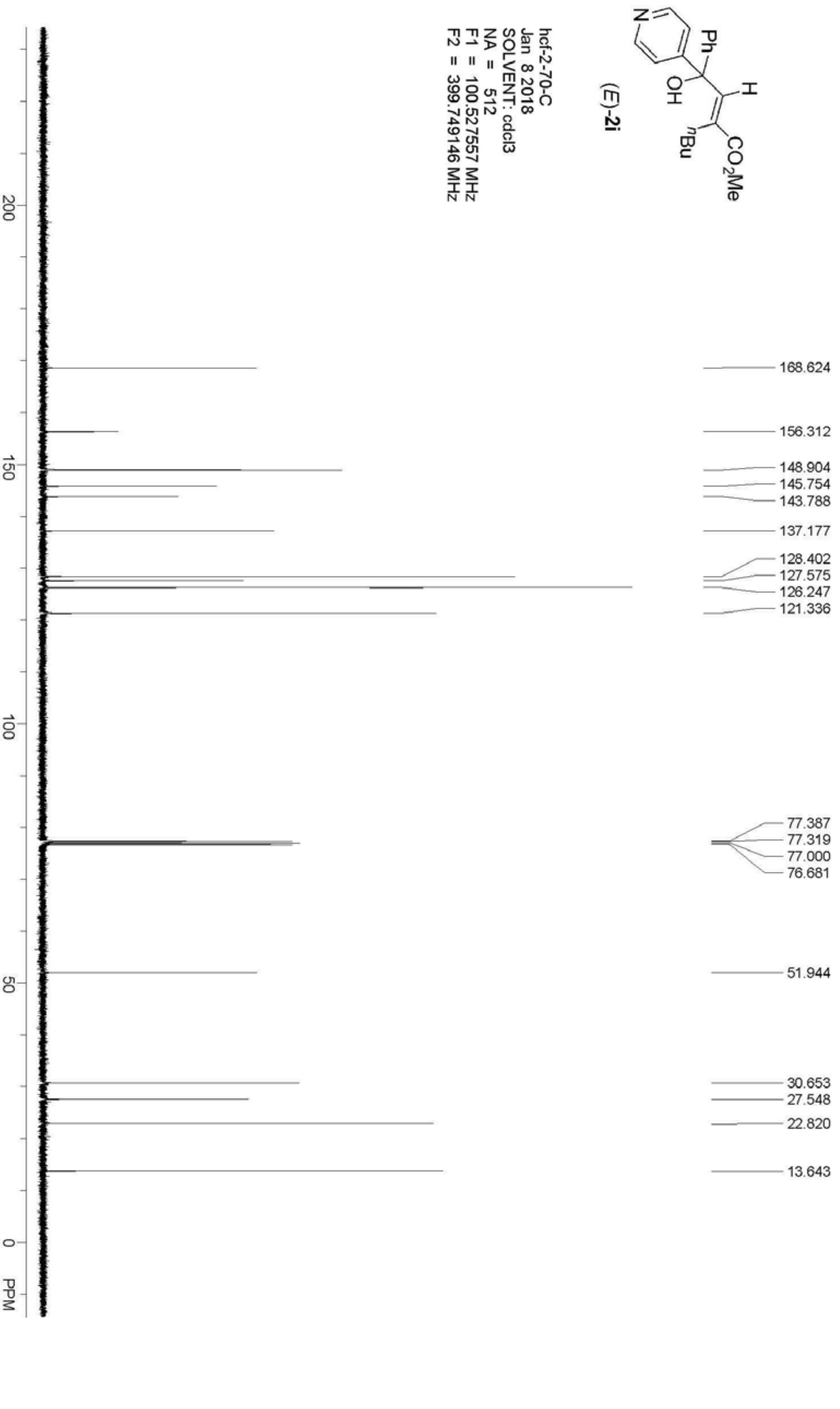

Ph
H
CO2Me
N
OH
nBu
(E)-2i
hcf-2-70-C
Jan 8 2018
SOLVENT: cdcl3
NA = 512
F1 = 100.527557 MHz
F2 = 399.749146 MHz
168.624
156.312
148.904
145.754
143.788
137.177
128.402
127.575
126.247
121.336
77.387
77.319
77.000
76.681
51.944
30.653
27.548
22.820
13.643
200
150
100
50
0
PPM

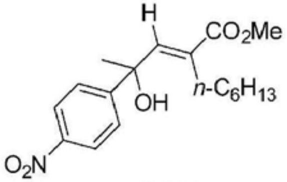

(*E*)-**2j**

hcf-2-82-H
Dec 7 2017
SOLVENT: cdcl3
NA = 8
F1 = 399.749542 MHz
F2 = 100.526031 MHz

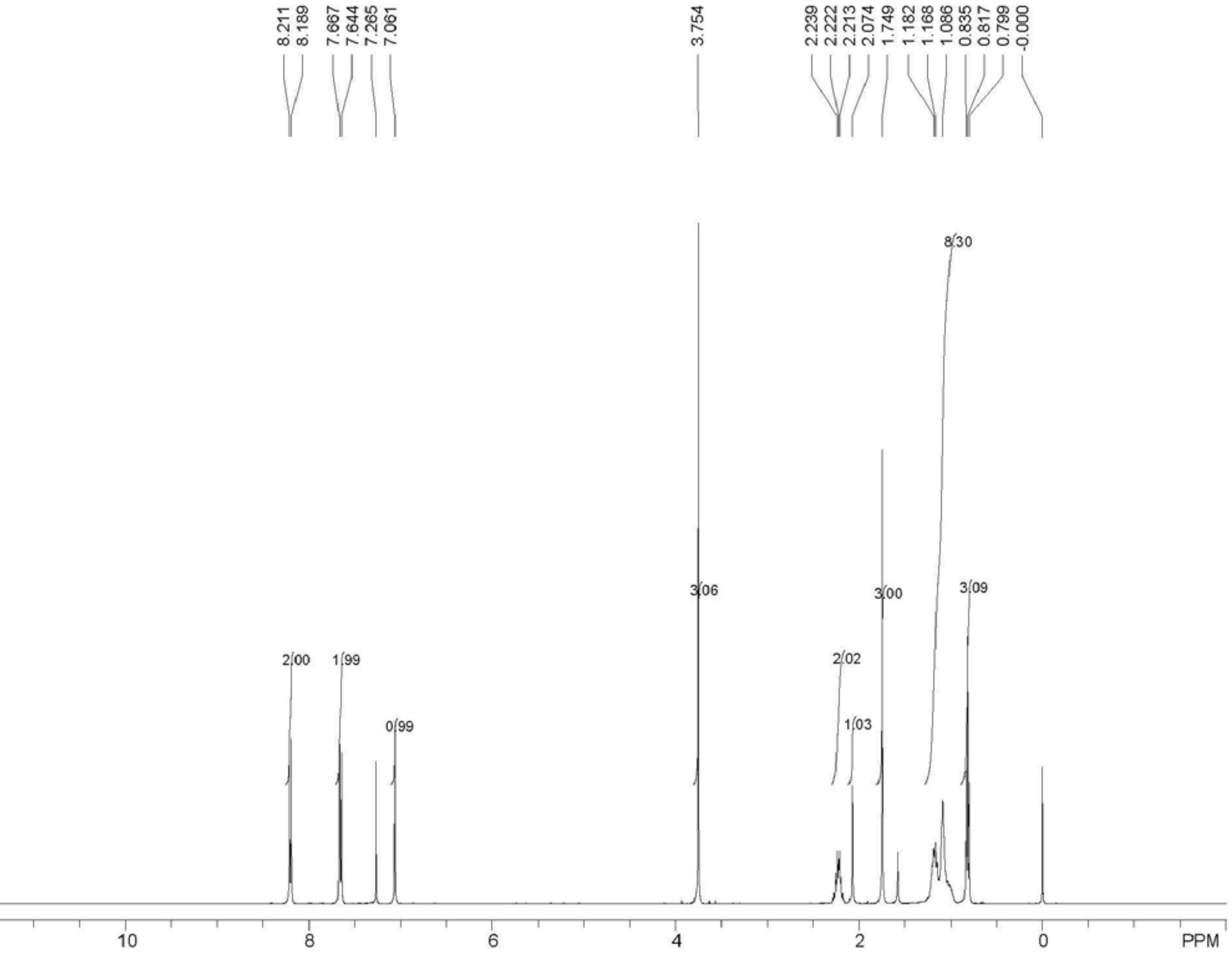

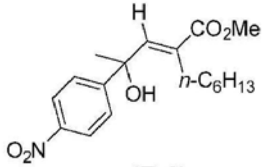

(*E*)-**2j**

hcf-2-82-C
Dec 7 2017
SOLVENT: cdcl3
NA = 1000
F1 = 100.527557 MHz
F2 = 399.749146 MHz

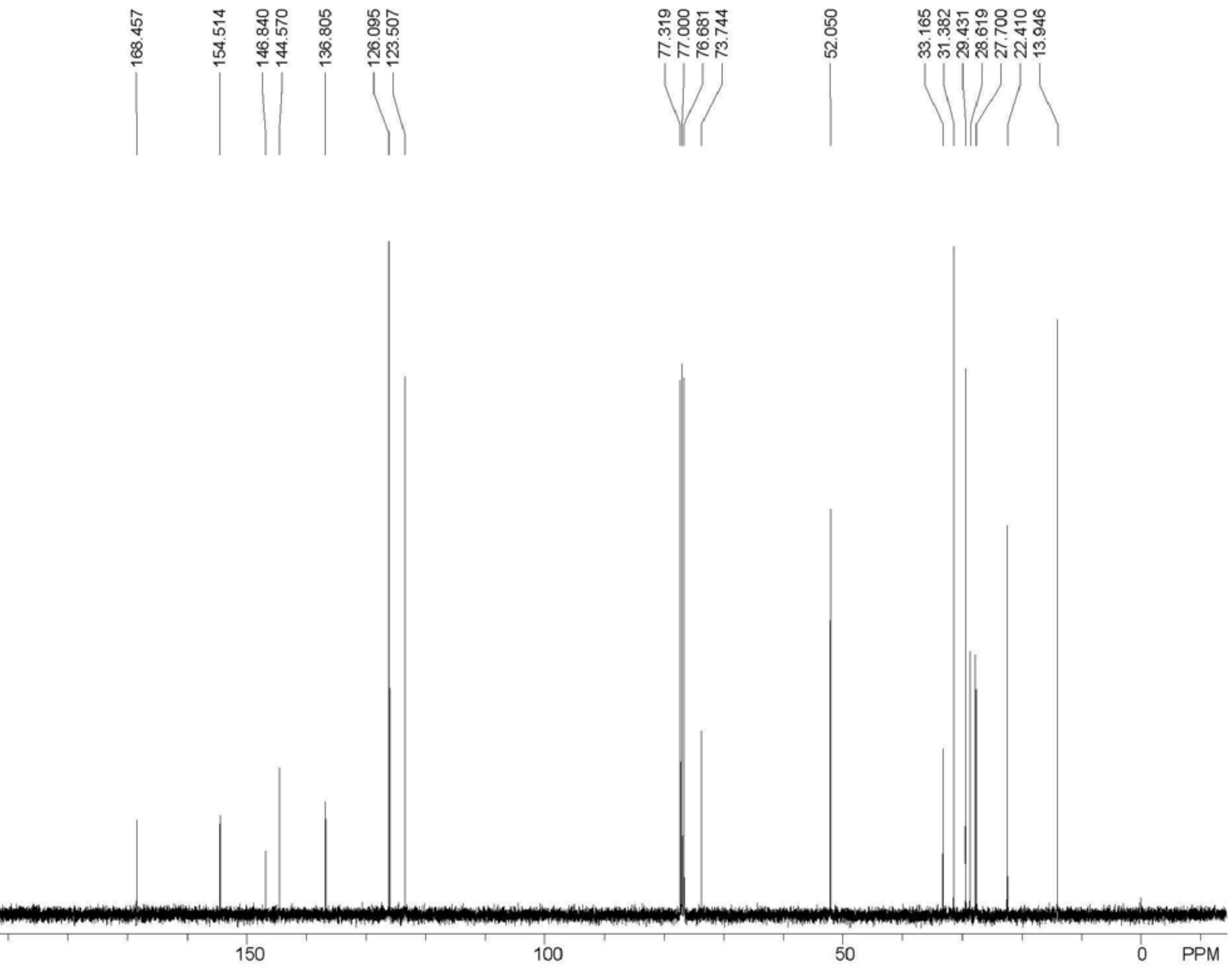

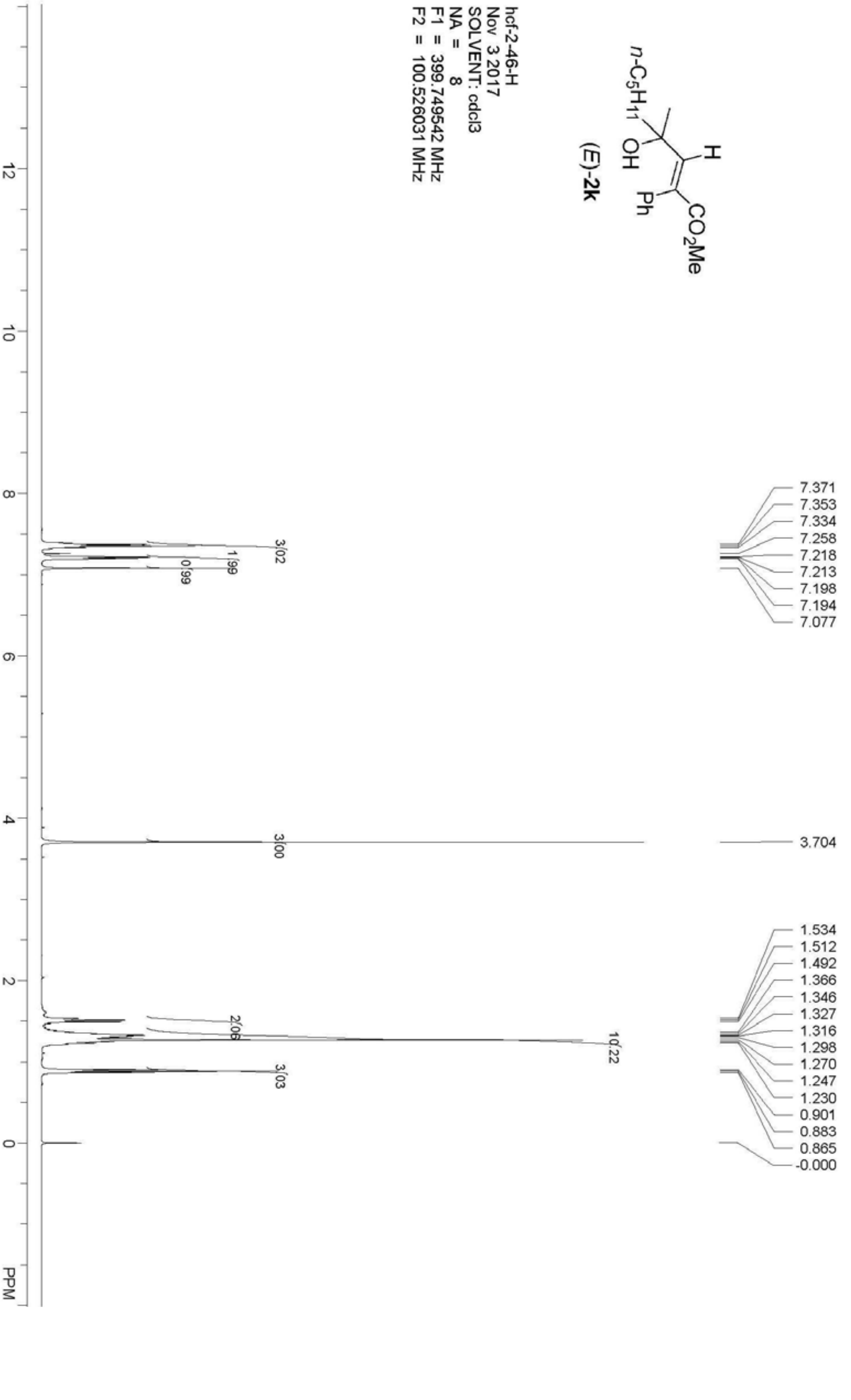
hcf-2-46-H
Nov 3 2017
SOLVENT: cdcl3
NA = 8
F1 = 399.749542 MHz
F2 = 100.526031 MHz
(E)-2k
H
CO2Me
Ph
OH
n-C5H11
7.371
7.353
7.334
7.258
7.218
7.213
7.198
7.194
7.077
3.704
1.534
1.512
1.492
1.366
1.346
1.327
1.316
1.298
1.270
1.247
1.230
0.901
0.883
0.865
-0.000
3.02
1.99
0.99
3.00
2.06
10.22
3.03
12
10
8
6
4
2
0
PPM

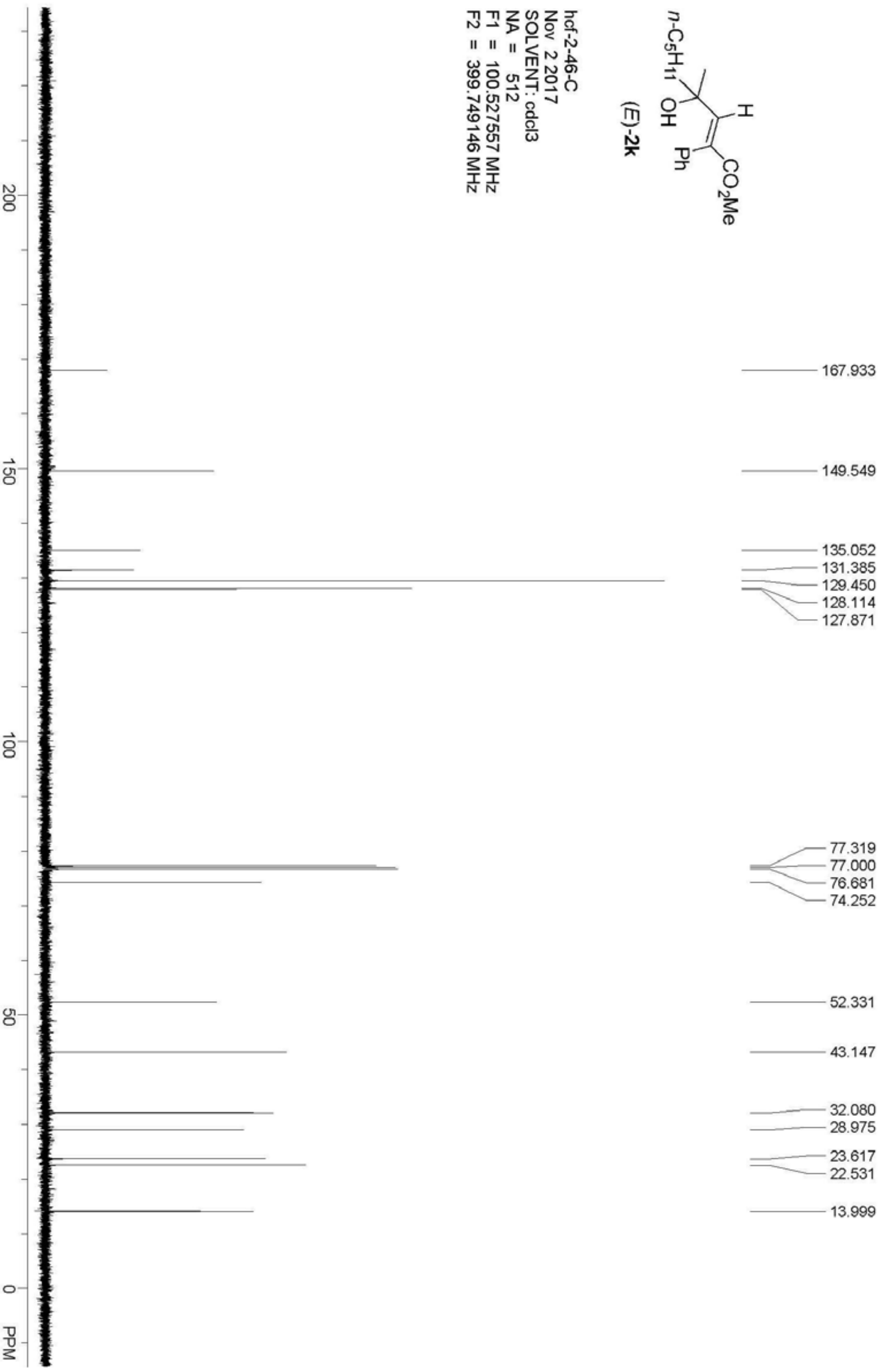

(E)-2k
hcf-2-46-C
Nov 2 2017
SOLVENT: cdcl3
NA = 512
F1 = 100.527557 MHz
F2 = 399.749146 MHz
167.933
149.549
135.052
131.385
129.450
128.114
127.871
77.319
77.000
76.681
74.252
52.331
43.147
32.080
28.975
23.617
22.531
13.999
200
150
100
50
0
PPM

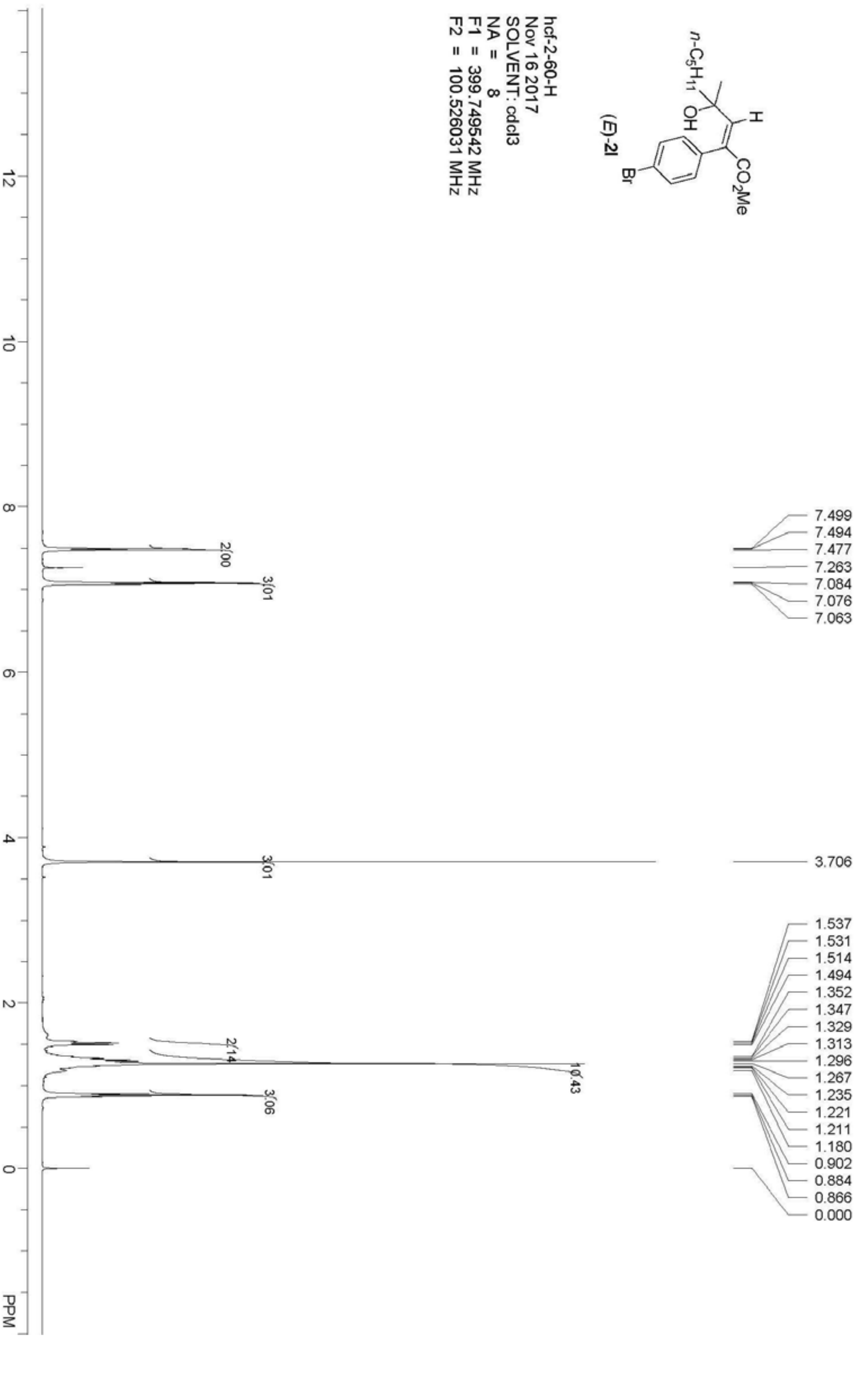
(E)-2l
hcf-2-60-H
Nov 16 2017
SOLVENT: cdcl3
NA = 8
F1 = 399.749542 MHz
F2 = 100.526031 MHz
7.499
7.494
7.477
7.263
7.084
7.076
7.063
3.706
1.537
1.531
1.514
1.494
1.352
1.347
1.329
1.313
1.296
1.267
1.235
1.221
1.211
1.180
0.902
0.884
0.866
0.000
2.00
3.01
3.01
2.14
10.43
3.06
PPM

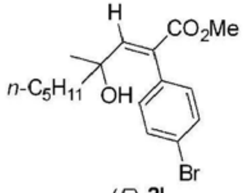

(*E*)-**2l**

hcf-2-60-C
Nov 16 2017
SOLVENT: cdcl3
NA = 512
F1 = 100.527557 MHz
F2 = 399.749146 MHz

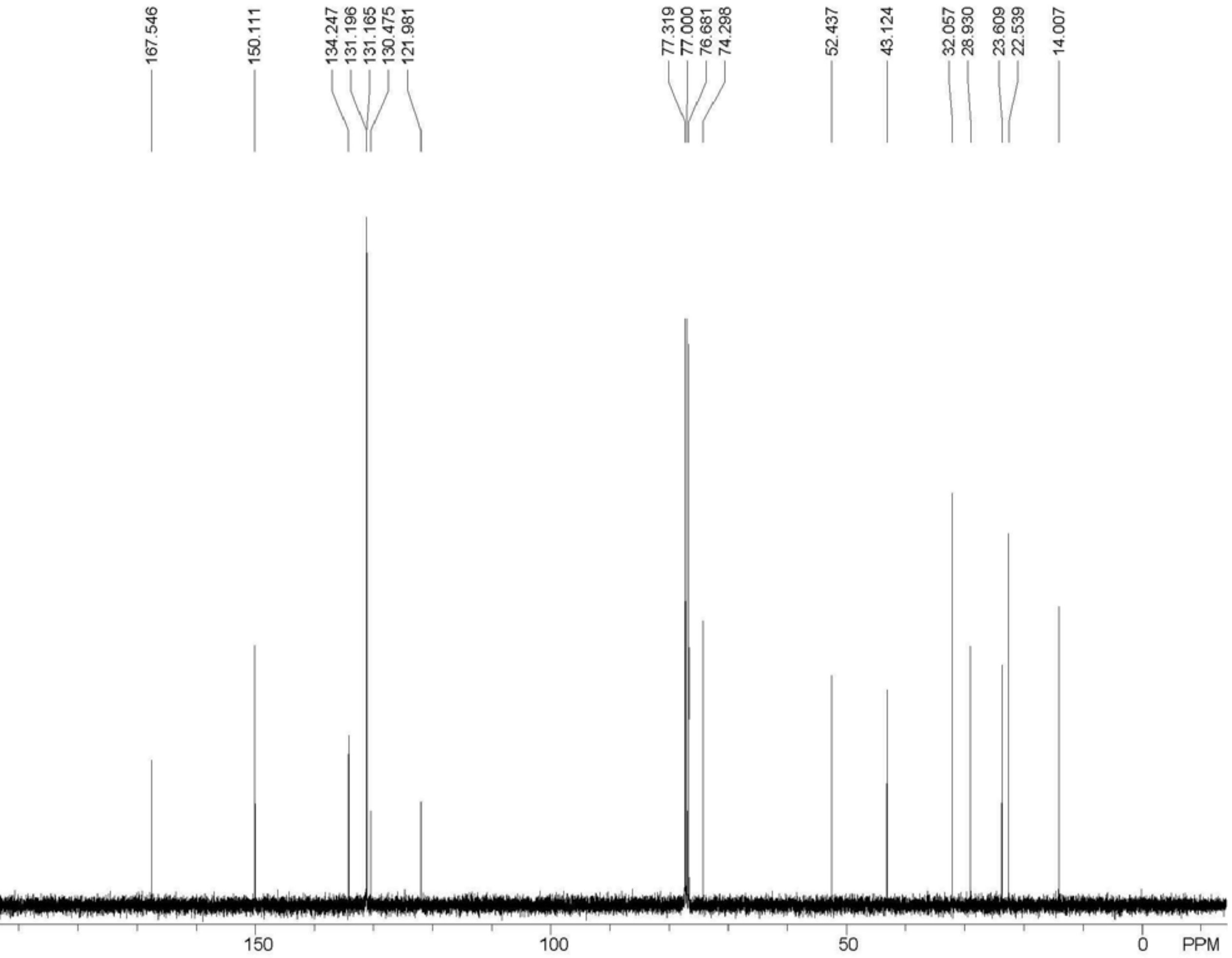

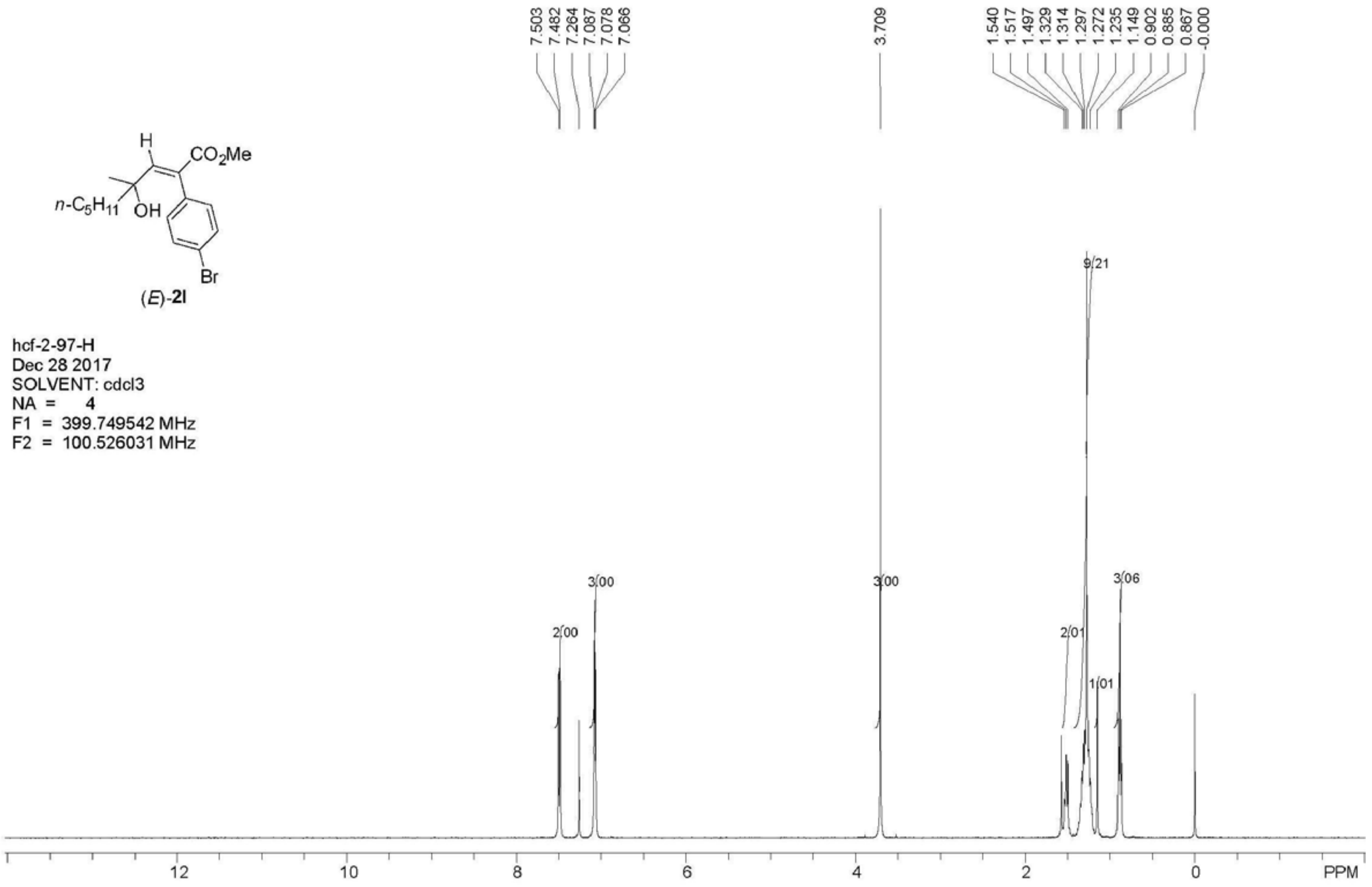

(*E*)-**2l**

hcf-2-97-H
Dec 28 2017
SOLVENT: cdcl3
NA = 4
F1 = 399.749542 MHz
F2 = 100.526031 MHz

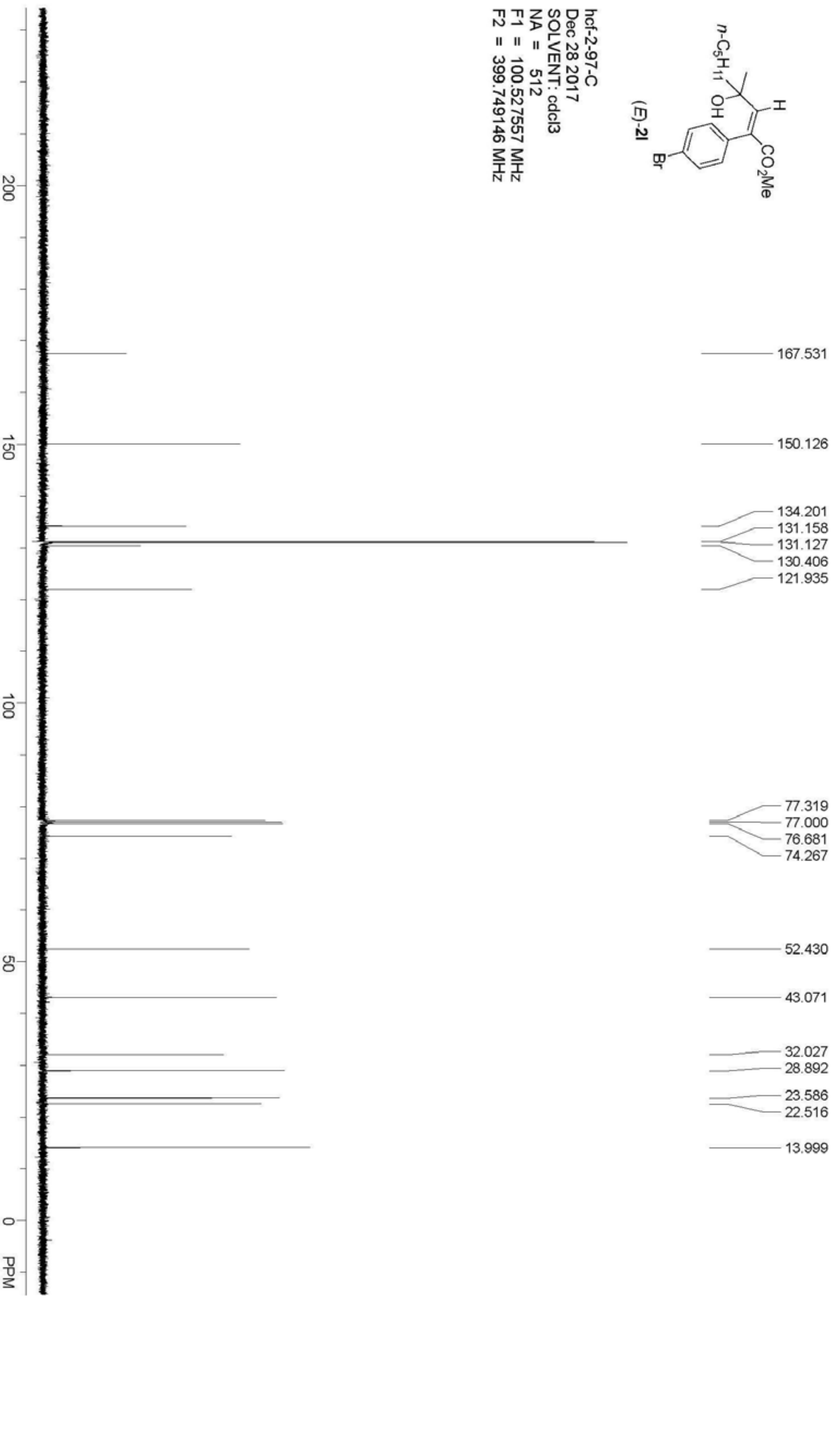

(E)-2l
hcf-2-97-C
Dec 28 2017
SOLVENT: cdcl3
NA = 512
F1 = 100.527557 MHz
F2 = 399.749146 MHz
167.531
150.126
134.201
131.158
131.127
130.406
121.935
77.319
77.000
76.681
74.267
52.430
43.071
32.027
28.892
23.586
22.516
13.999
PPM

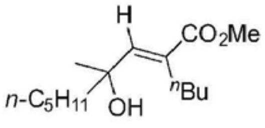

(*E*)-**2m**

hcf-2-92-H
Dec 14 2017
SOLVENT: cdcl3
NA = 4
F1 = 399.749542 MHz
F2 = 100.526031 MHz

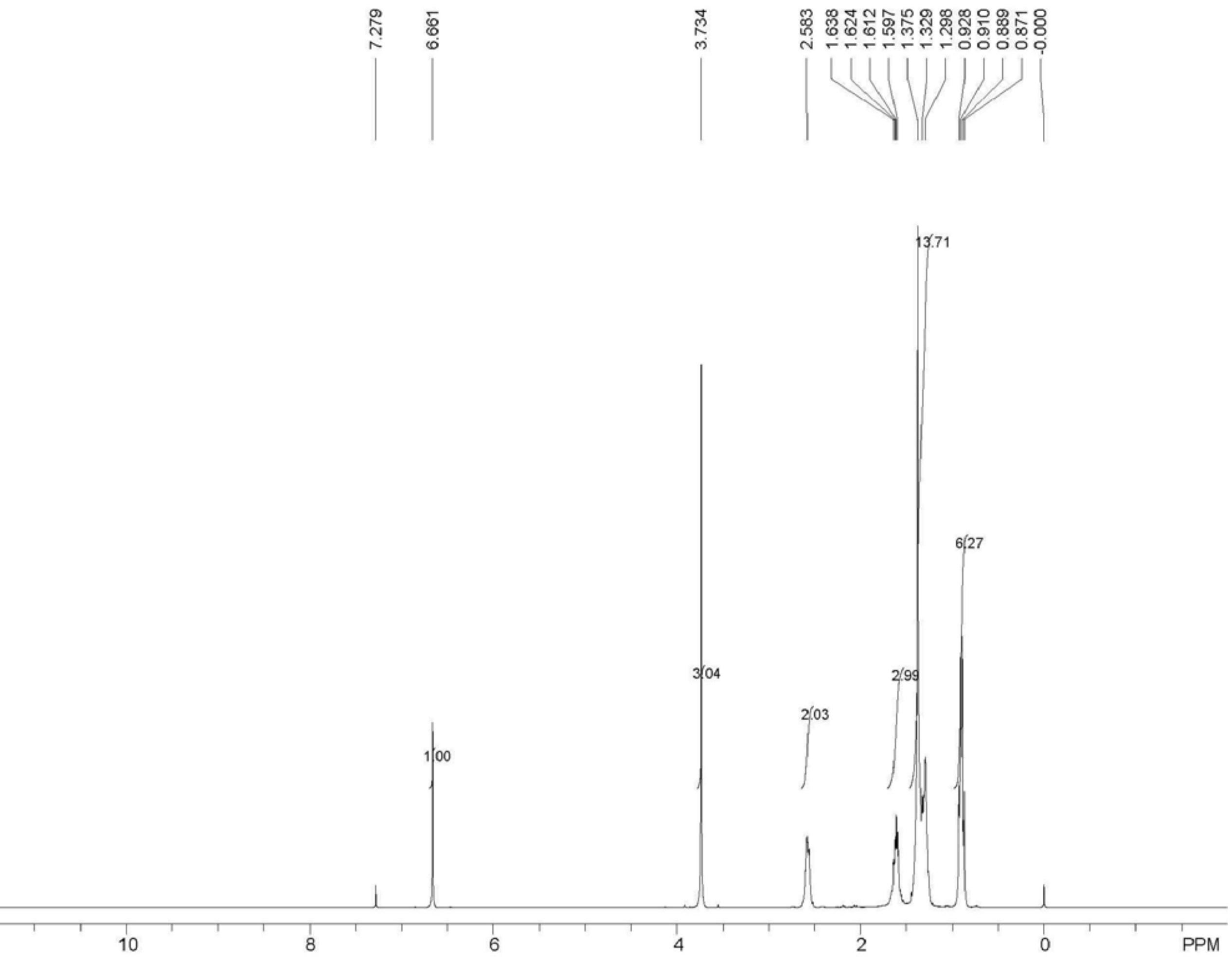

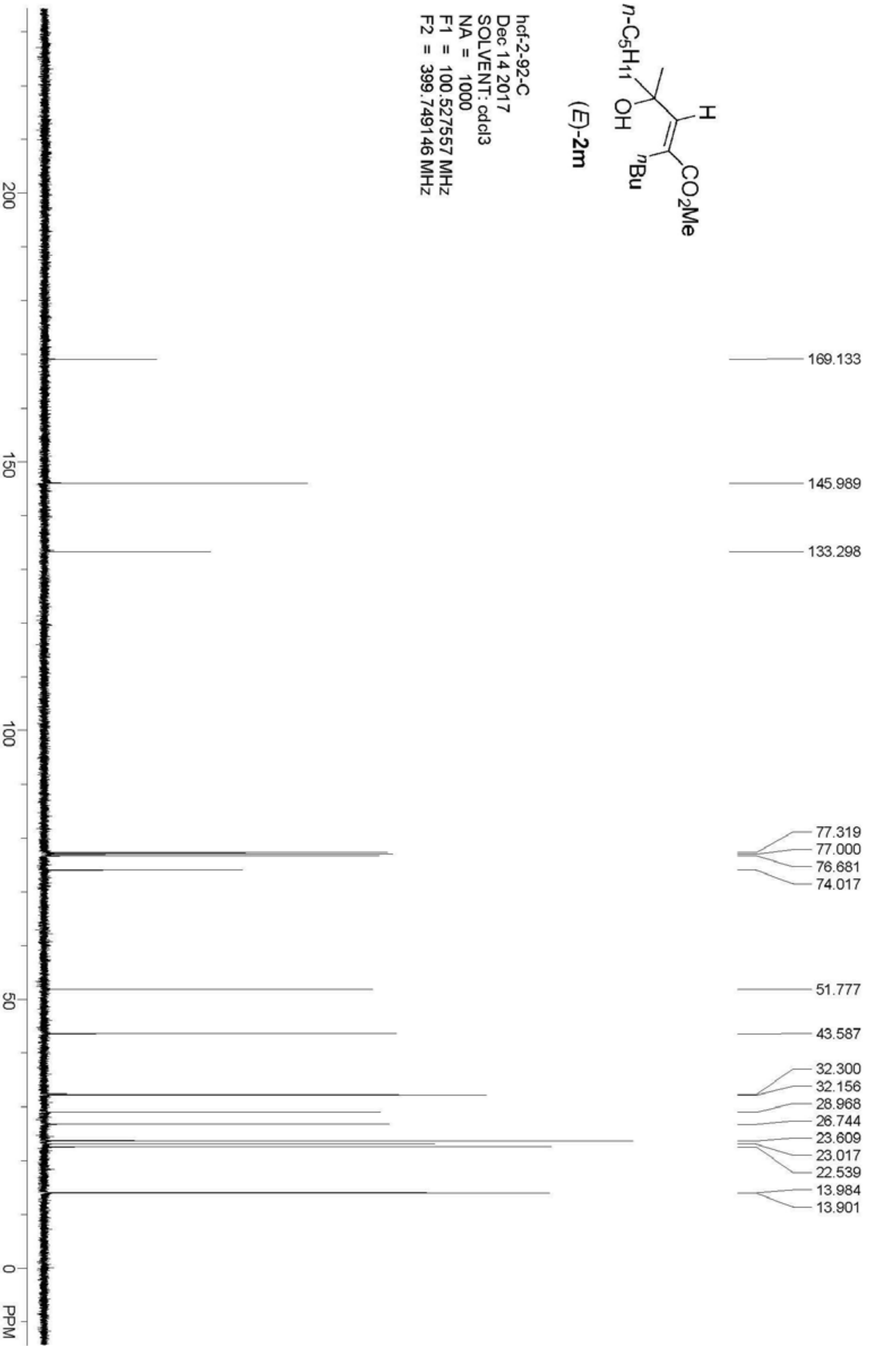
(E)-2m
hcf-2-92-C
Dec 14 2017
SOLVENT: cdcl3
NA = 1000
F1 = 100.527557 MHz
F2 = 399.749146 MHz
169.133
145.989
133.298
77.319
77.000
76.681
74.017
51.777
43.587
32.300
32.156
28.968
26.744
23.609
23.017
22.539
13.984
13.901
200
150
100
50
0
PPM

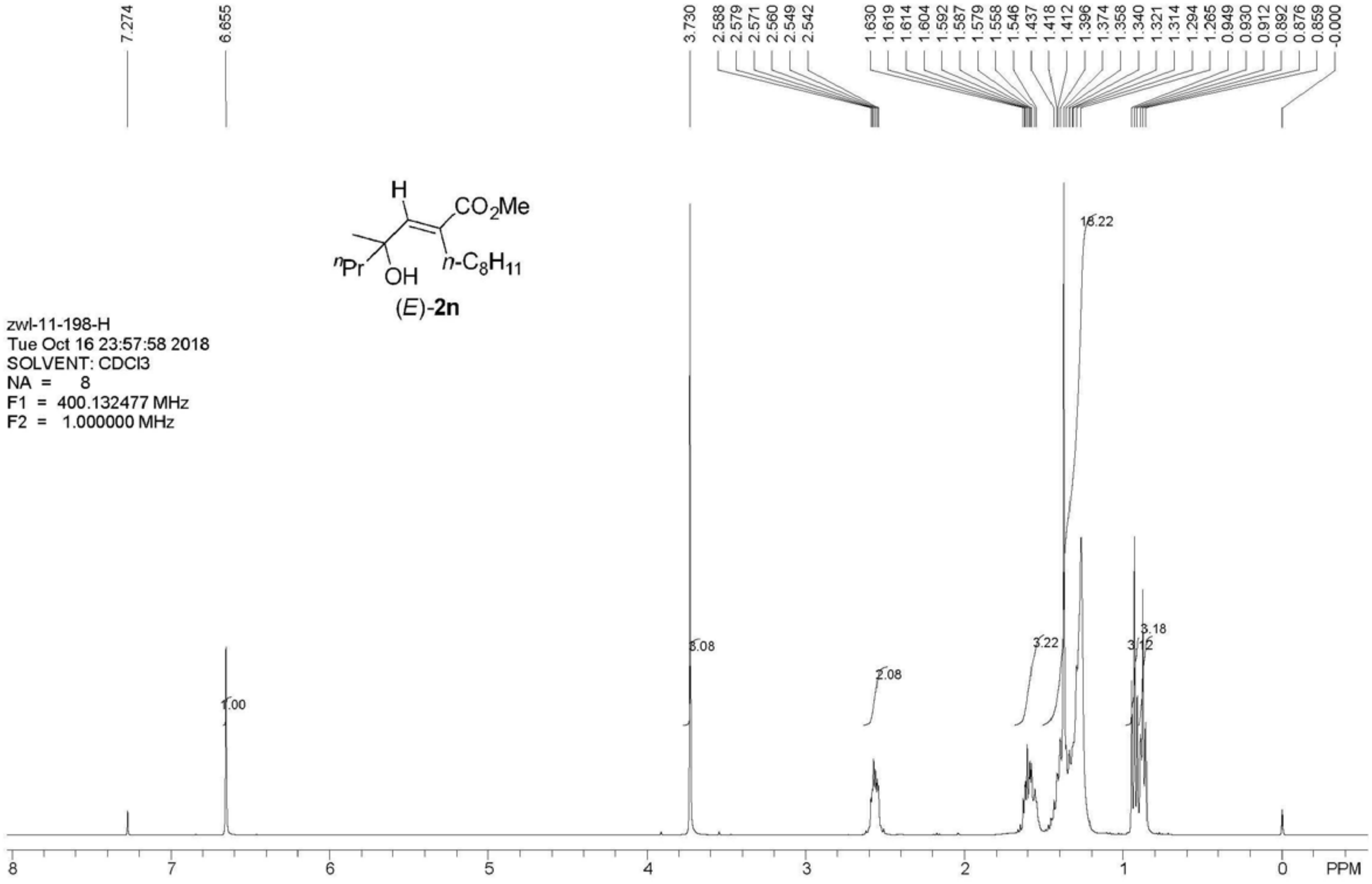
H
CO_2Me
^{n}Pr
OH
n-C_8H_{11}
(*E*)-**2n**
zwl-11-198-H
Tue Oct 16 23:57:58 2018
SOLVENT: CDCl3
NA = 8
F1 = 400.132477 MHz
F2 = 1.000000 MHz
7.274
6.655
3.730
2.588
2.579
2.571
2.560
2.549
2.542
1.630
1.619
1.614
1.604
1.592
1.587
1.579
1.558
1.546
1.437
1.418
1.412
1.396
1.374
1.358
1.340
1.321
1.314
1.294
1.265
0.949
0.930
0.912
0.892
0.876
0.859
-0.000
1.00
3.08
2.08
3.22
16.22
3.12
3.18
8
7
6
5
4
3
2
1
0
PPM

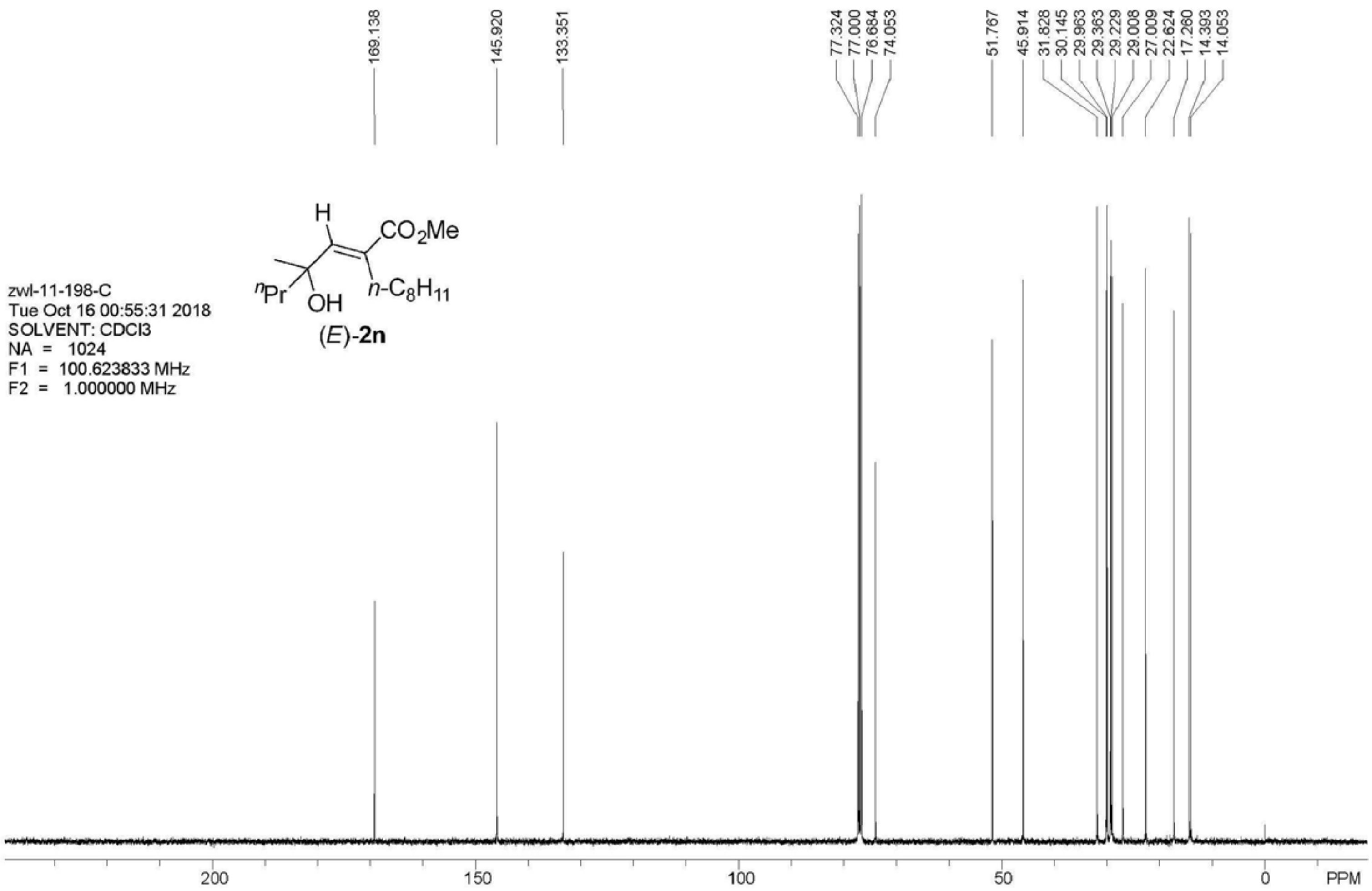

zwl-11-198-C
Tue Oct 16 00:55:31 2018
SOLVENT: CDCl3
NA = 1024
F1 = 100.623833 MHz
F2 = 1.000000 MHz
H
CO_2Me
^nPr
OH
n-C_8H_{11}
(E)-**2n**
169.138
145.920
133.351
77.324
77.000
76.684
74.053
51.767
45.914
31.828
30.145
29.963
29.363
29.229
29.008
27.009
22.624
17.260
14.393
14.053
200
150
100
50
0
PPM

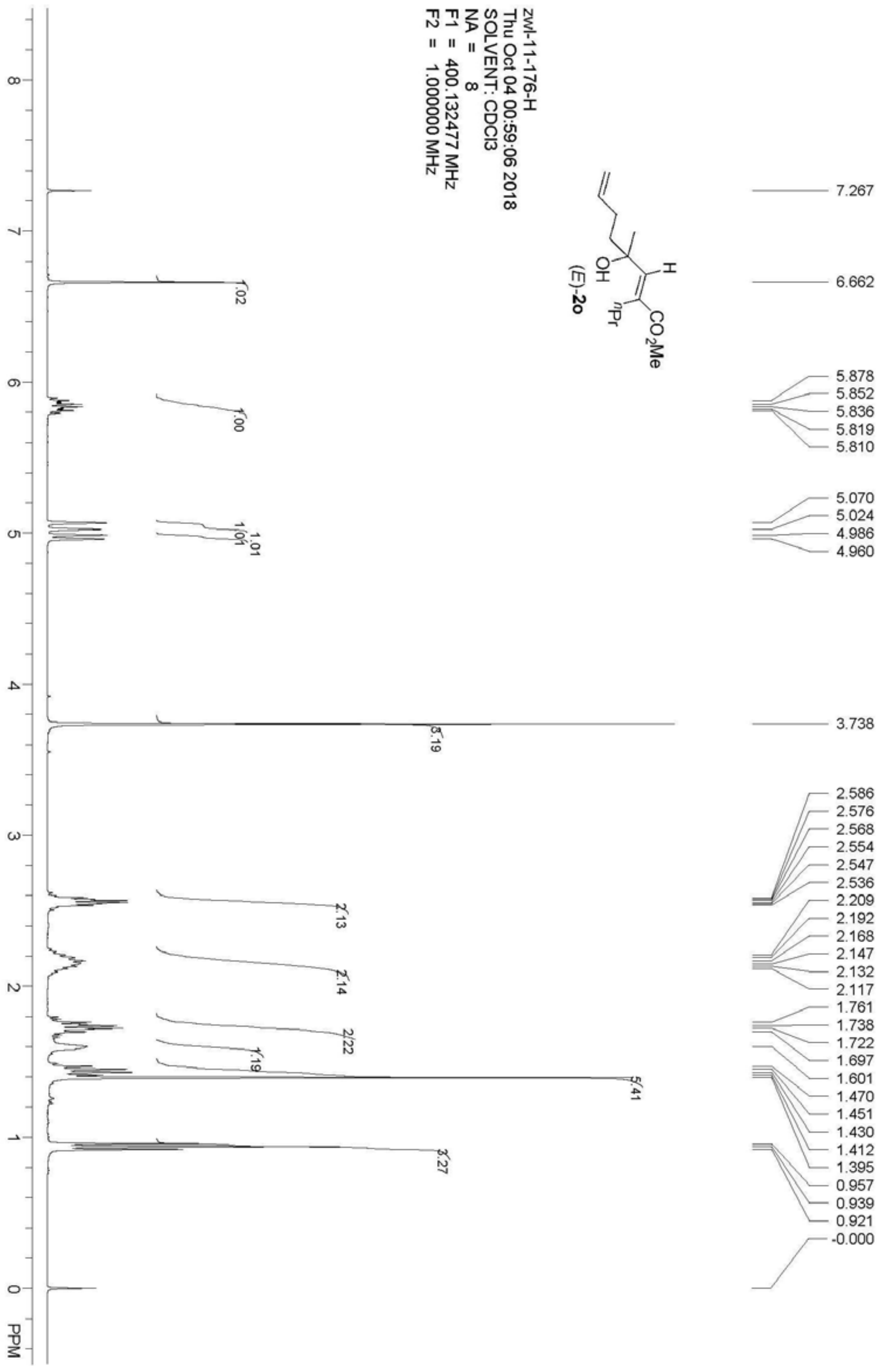
zwl-11-176-H
Thu Oct 04 00:59:06 2018
SOLVENT: CDCl3
NA = 8
F1 = 400.132477 MHz
F2 = 1.000000 MHz
H
CO2Me
nPr
OH
(E)-2o
7.267
6.662
5.878
5.852
5.836
5.819
5.810
5.070
5.024
4.986
4.960
3.738
2.586
2.576
2.568
2.554
2.547
2.536
2.209
2.192
2.168
2.147
2.132
2.117
1.761
1.738
1.722
1.697
1.601
1.470
1.451
1.430
1.412
1.395
0.957
0.939
0.921
-0.000
1.02
1.00
1.01
1.01
3.19
2.13
2.14
2.22
1.19
5.41
3.27
8
7
6
5
4
3
2
1
0
PPM

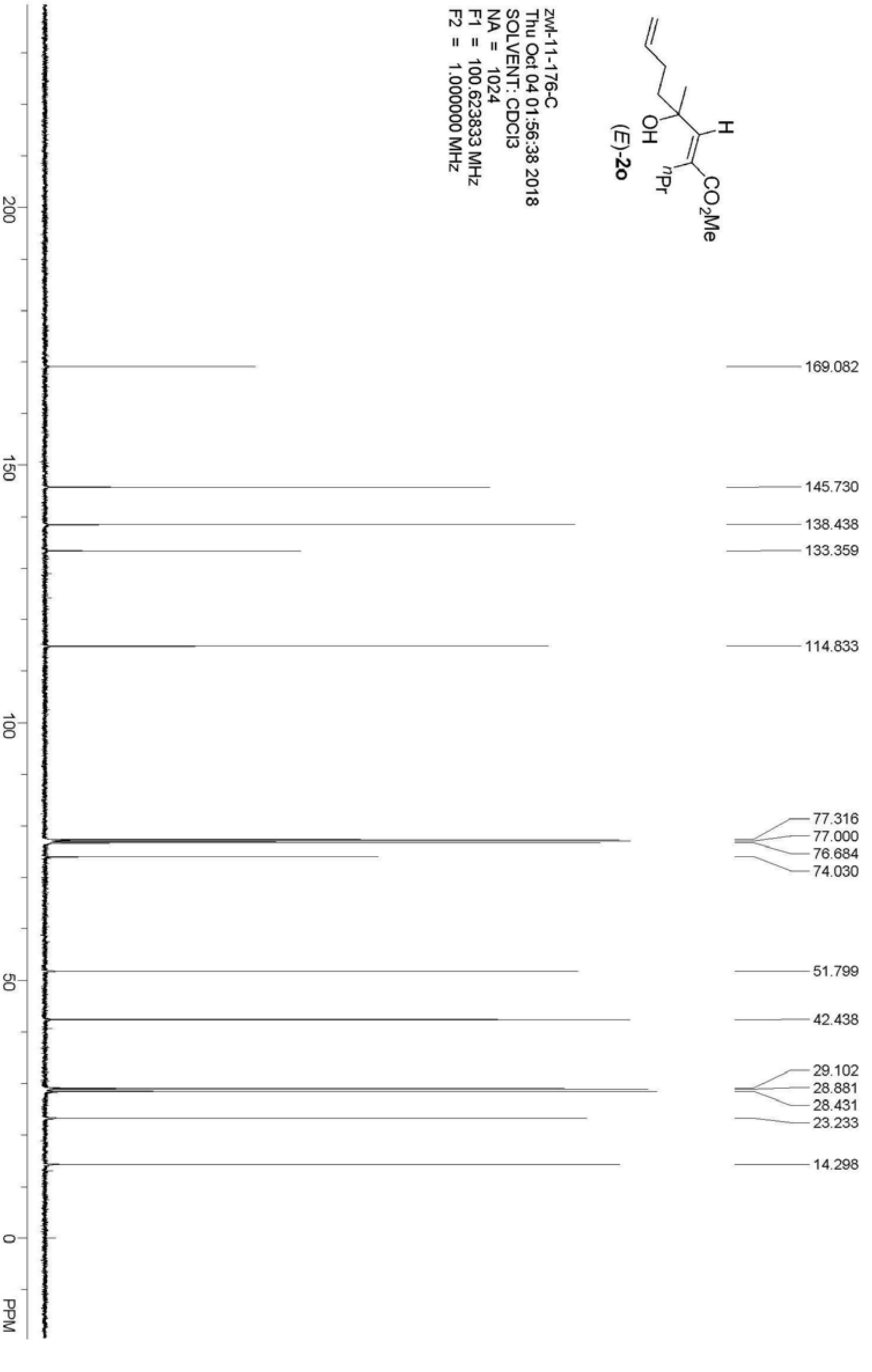

zwl-11-176-C
Thu Oct 04 01:56:38 2018
SOLVENT: CDCl3
NA = 1024
F1 = 100.623833 MHz
F2 = 1.000000 MHz
H
CO_2Me
nPr
OH
(E)-2o
169.082
145.730
138.438
133.359
114.833
77.316
77.000
76.684
74.030
51.799
42.438
29.102
28.881
28.431
23.233
14.298
200
150
100
50
0
PPM

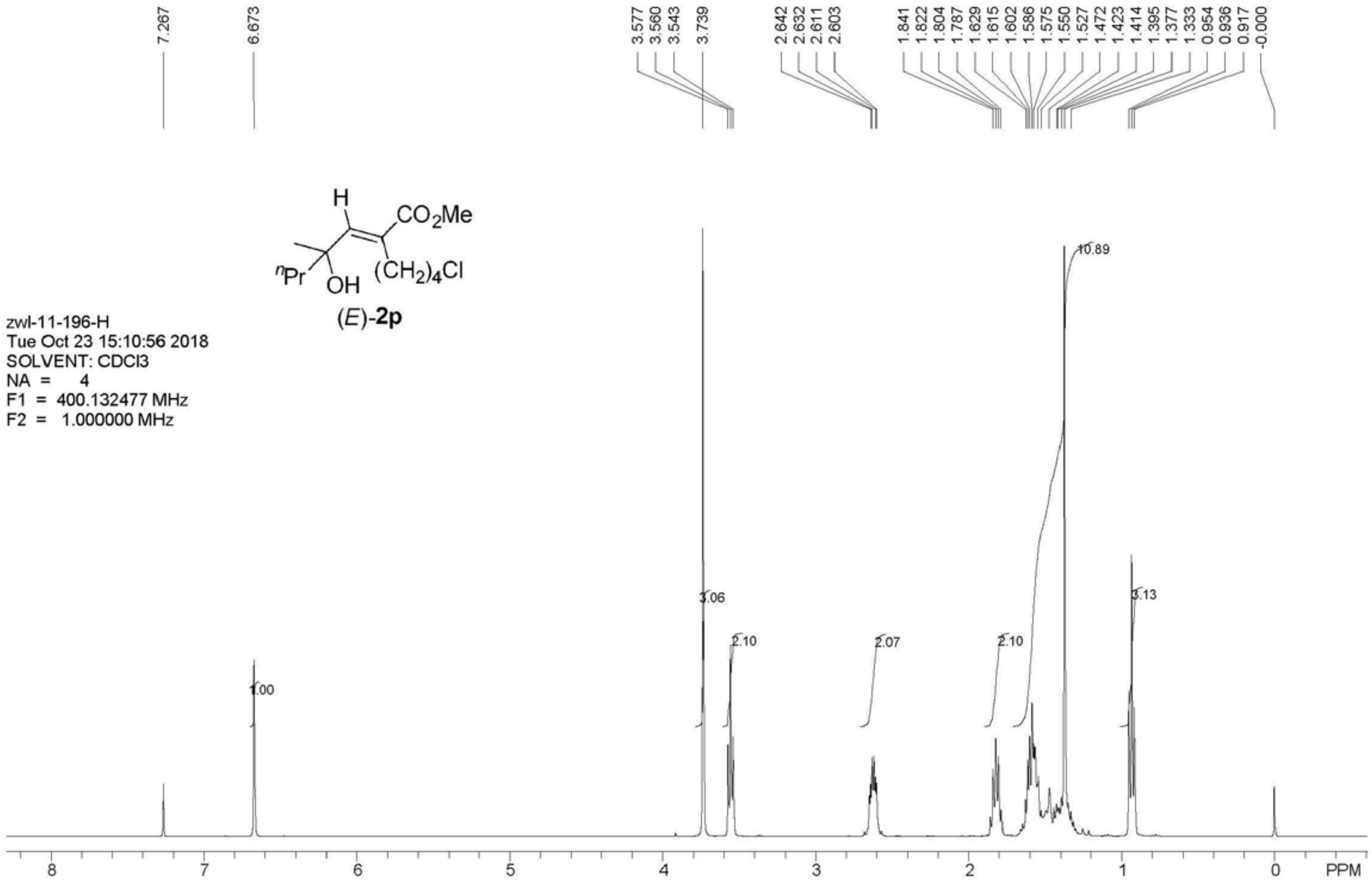
7.267
6.673
3.577
3.560
3.543
3.739
2.642
2.632
2.611
2.603
1.841
1.822
1.804
1.787
1.629
1.615
1.602
1.586
1.575
1.550
1.527
1.472
1.423
1.414
1.395
1.377
1.333
0.954
0.936
0.917
-0.000
H
CO_2Me
^{n}Pr
OH
$(CH_2)_4Cl$
(*E*)-**2p**
zwl-11-196-H
Tue Oct 23 15:10:56 2018
SOLVENT: CDCl3
NA = 4
F1 = 400.132477 MHz
F2 = 1.000000 MHz
1.00
3.06
2.10
2.07
2.10
10.89
3.13
8
7
6
5
4
3
2
1
0
PPM

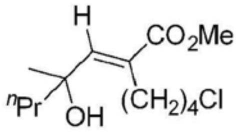

(*E*)-**2p**

zwl-11-196-C
Tue Oct 23 23:42:51 2018
SOLVENT: CDCl3
NA = 256
F1 = 100.623833 MHz
F2 = 1.000000 MHz

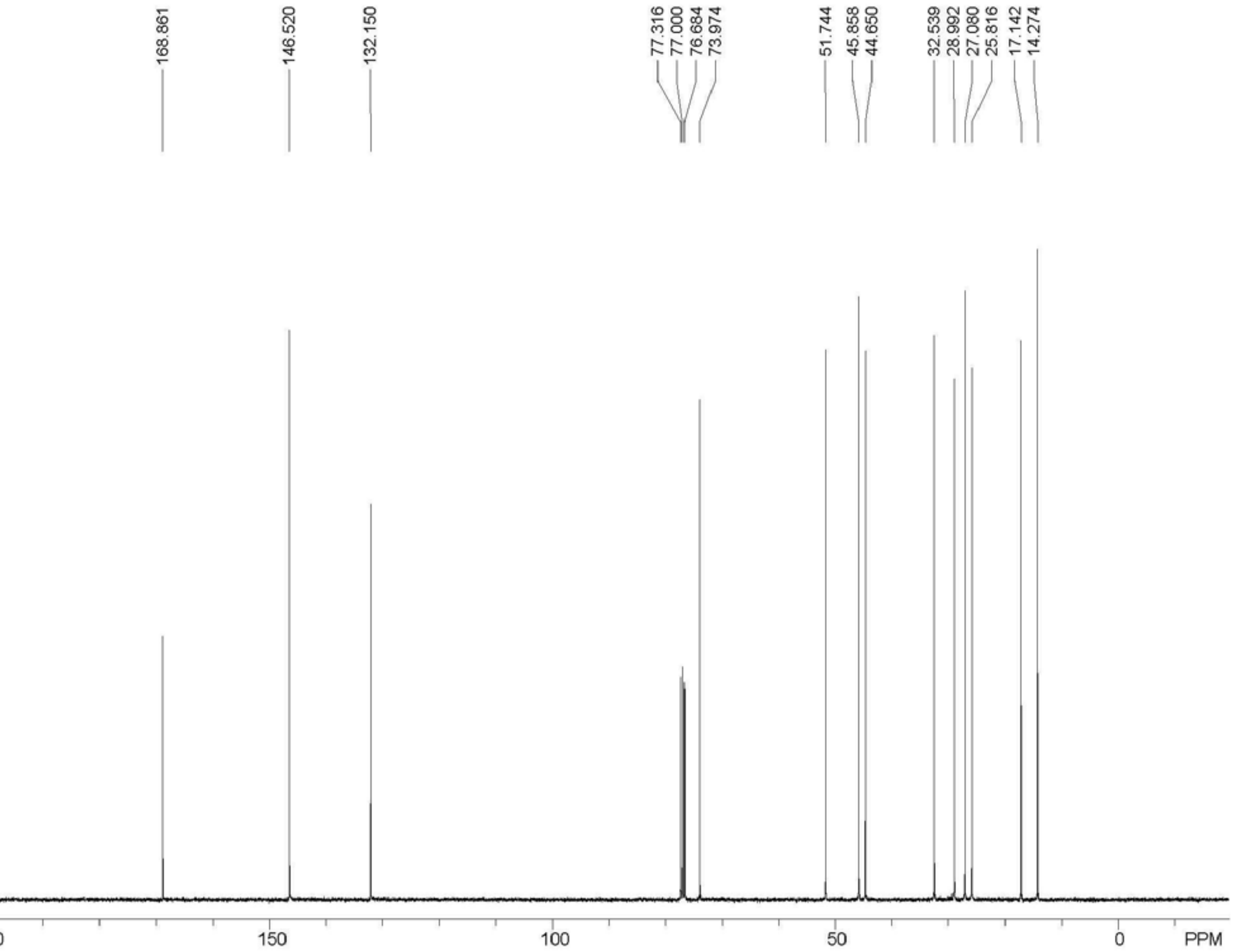

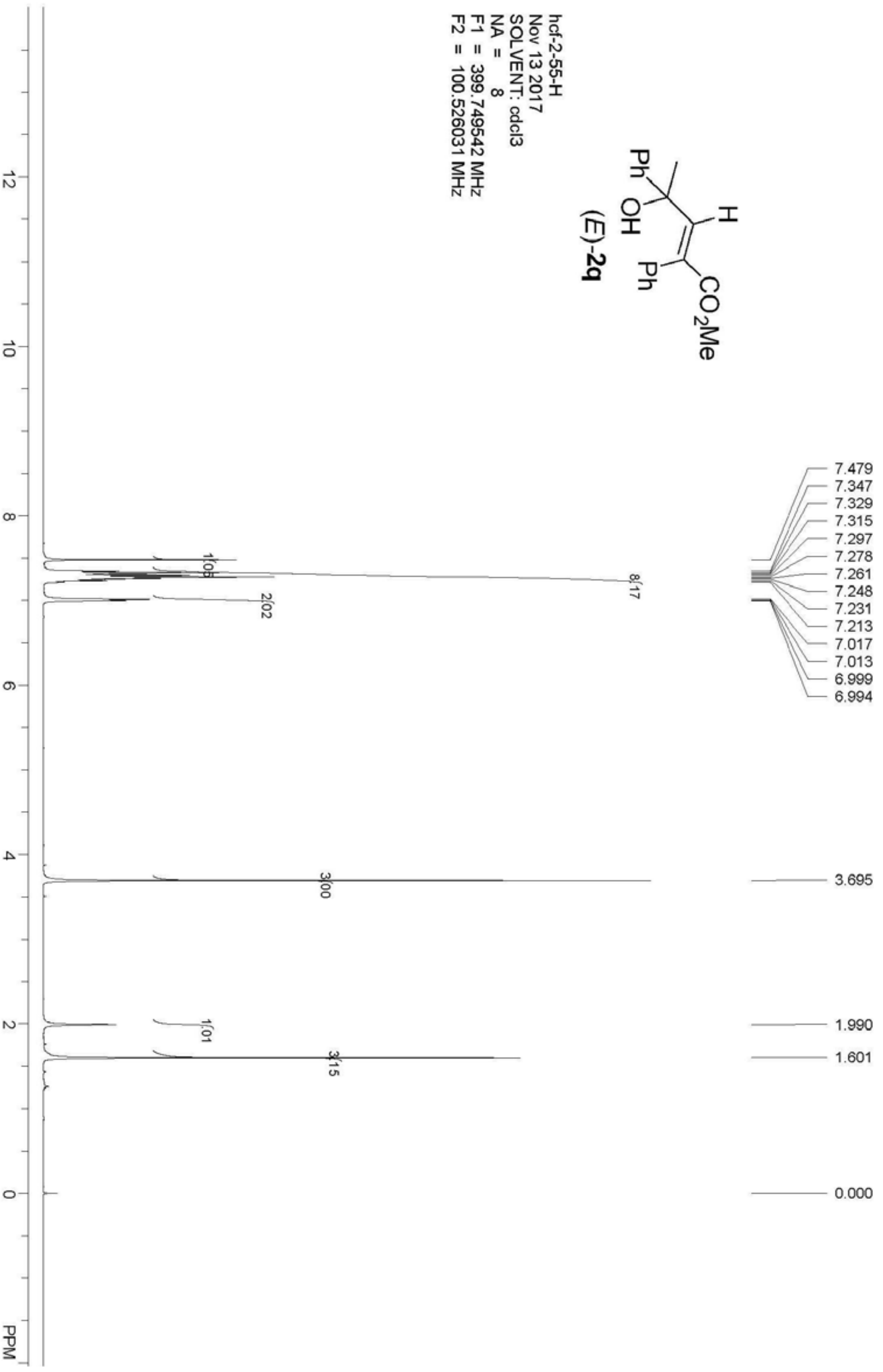
hcf-2-55-H
Nov 13 2017
SOLVENT: cdcl3
NA = 8
F1 = 399.749542 MHz
F2 = 100.526031 MHz
(E)-2q
7.479
7.347
7.329
7.315
7.297
7.278
7.261
7.248
7.231
7.213
7.017
7.013
6.999
6.994
3.695
1.990
1.601
0.000
PPM

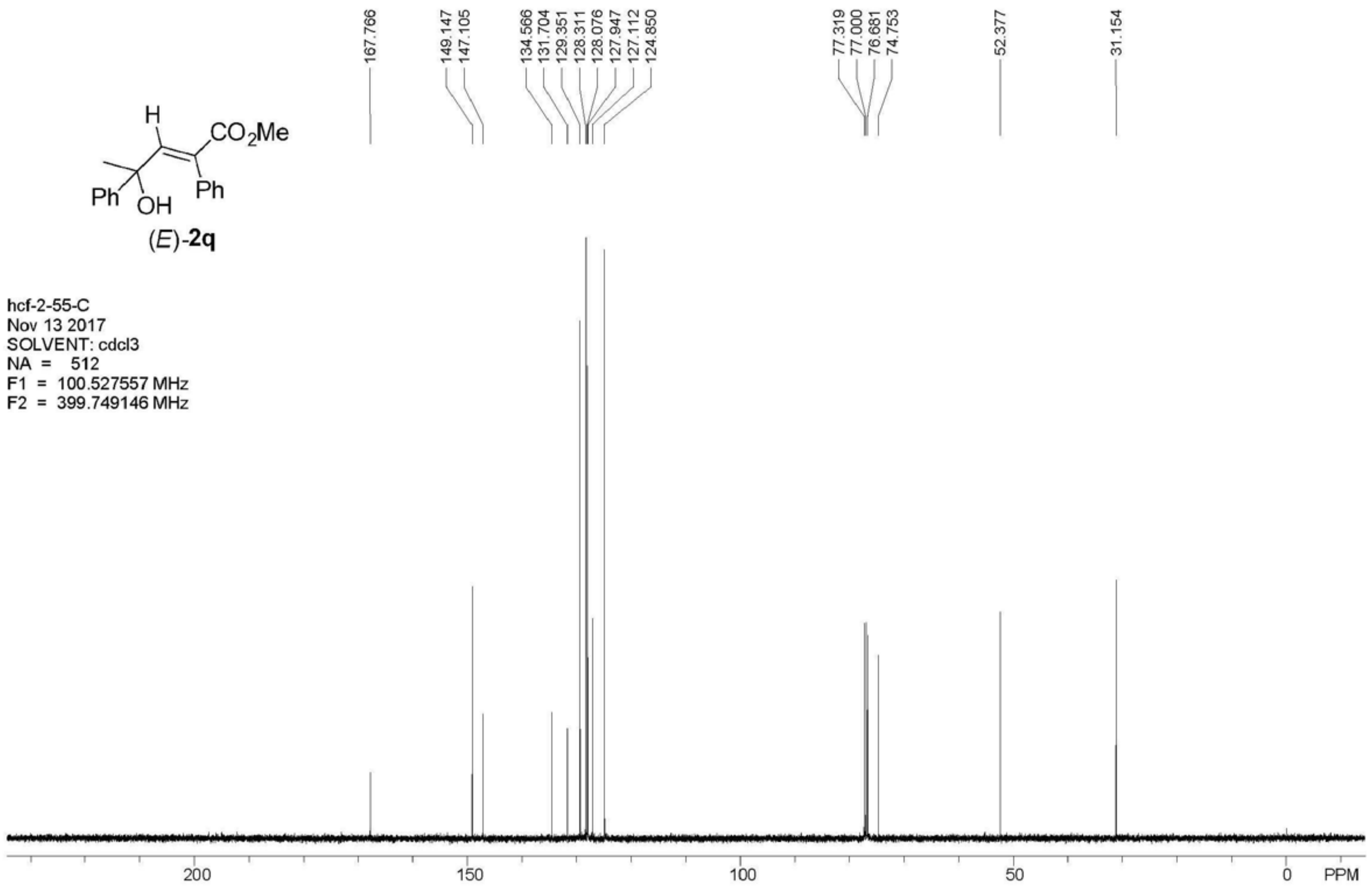
CO2Me
H
Ph
Ph
OH
(E)-2q
hcf-2-55-C
Nov 13 2017
SOLVENT: cdcl3
NA = 512
F1 = 100.527557 MHz
F2 = 399.749146 MHz
167.766
149.147
147.105
134.566
131.704
129.351
128.311
128.076
127.947
127.112
124.850
77.319
77.000
76.681
74.753
52.377
31.154
200
150
100
50
0
PPM

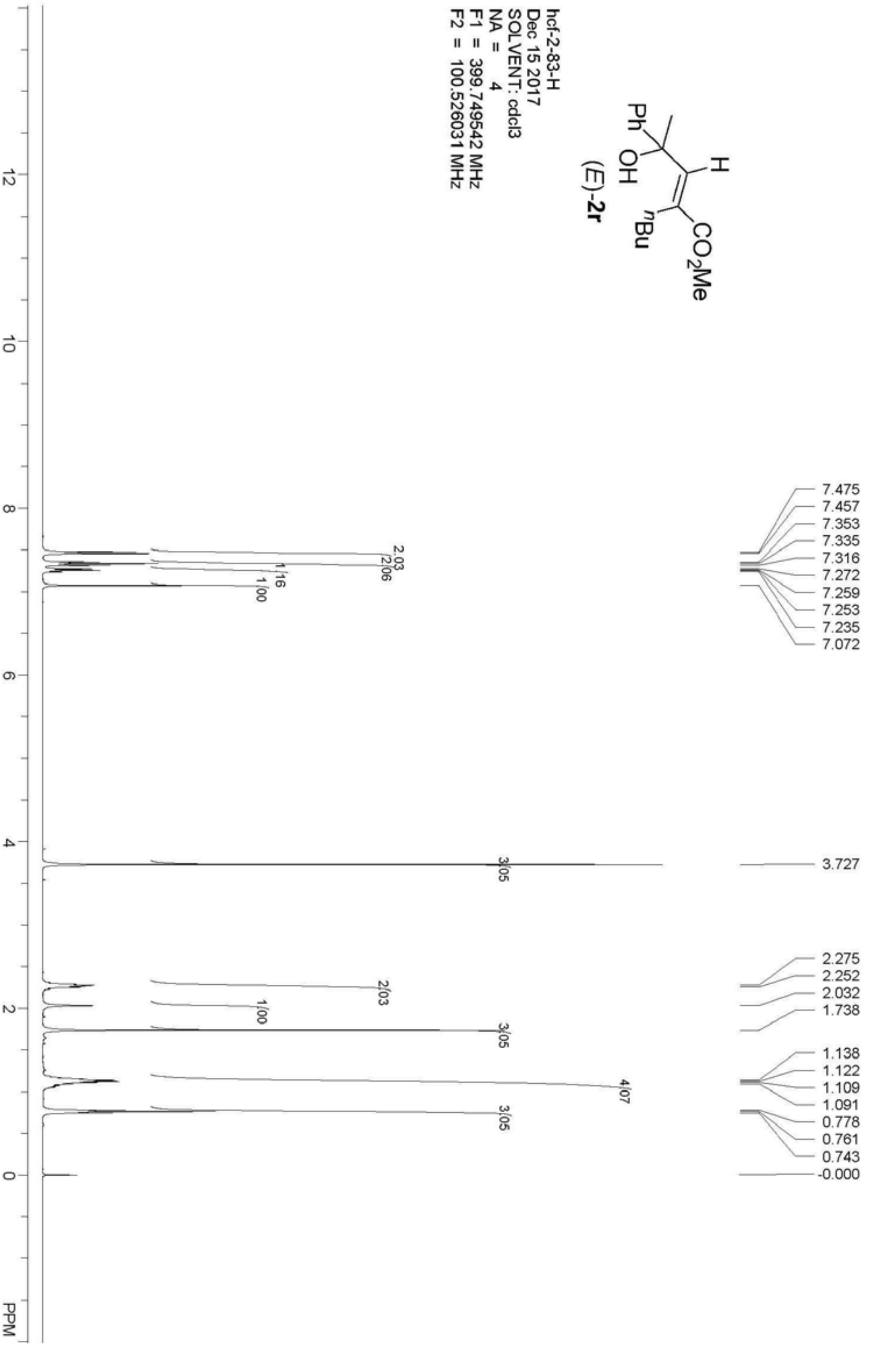
hcf-2-83-H
Dec 15 2017
SOLVENT: cdcl3
NA = 4
F1 = 399.749542 MHz
F2 = 100.526031 MHz
(E)-2r
CO2Me
nBu
H
Ph
OH
7.475
7.457
7.353
7.335
7.316
7.272
7.259
7.253
7.235
7.072
3.727
2.275
2.252
2.032
1.738
1.138
1.122
1.109
1.091
0.778
0.761
0.743
-0.000
2.03
2.06
1.16
1.00
3.05
2.03
1.00
3.05
4.07
3.05
12
10
8
6
4
2
0
PPM

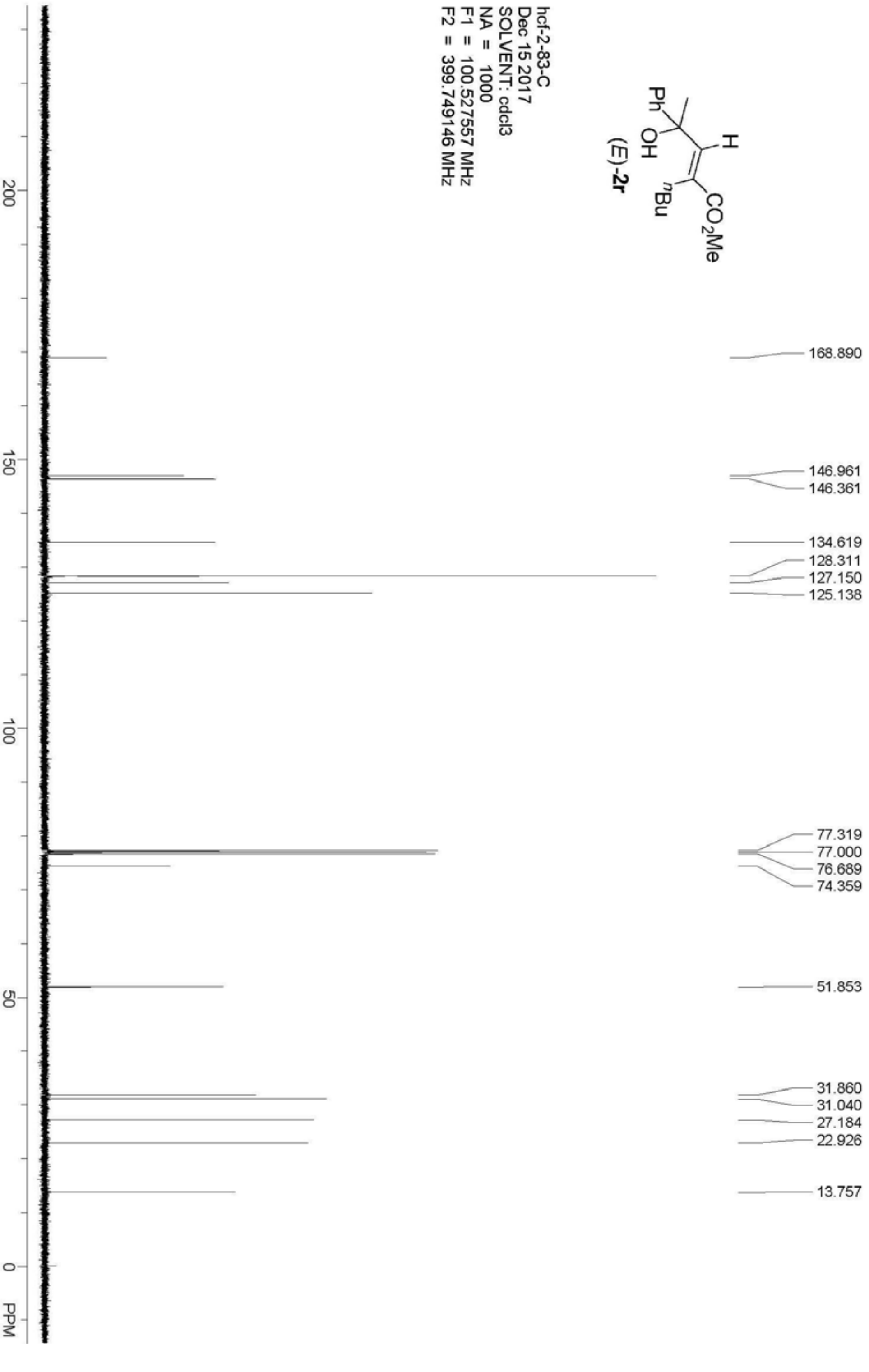

hcf-2-83-C
Dec 15 2017
SOLVENT: cdcl3
NA = 1000
F1 = 100.527557 MHz
F2 = 399.749146 MHz
H
CO2Me
Ph
OH
nBu
(E)-2r
168.890
146.961
146.361
134.619
128.311
127.150
125.138
77.319
77.000
76.689
74.359
51.853
31.860
31.040
27.184
22.926
13.757
200
150
100
50
0
PPM

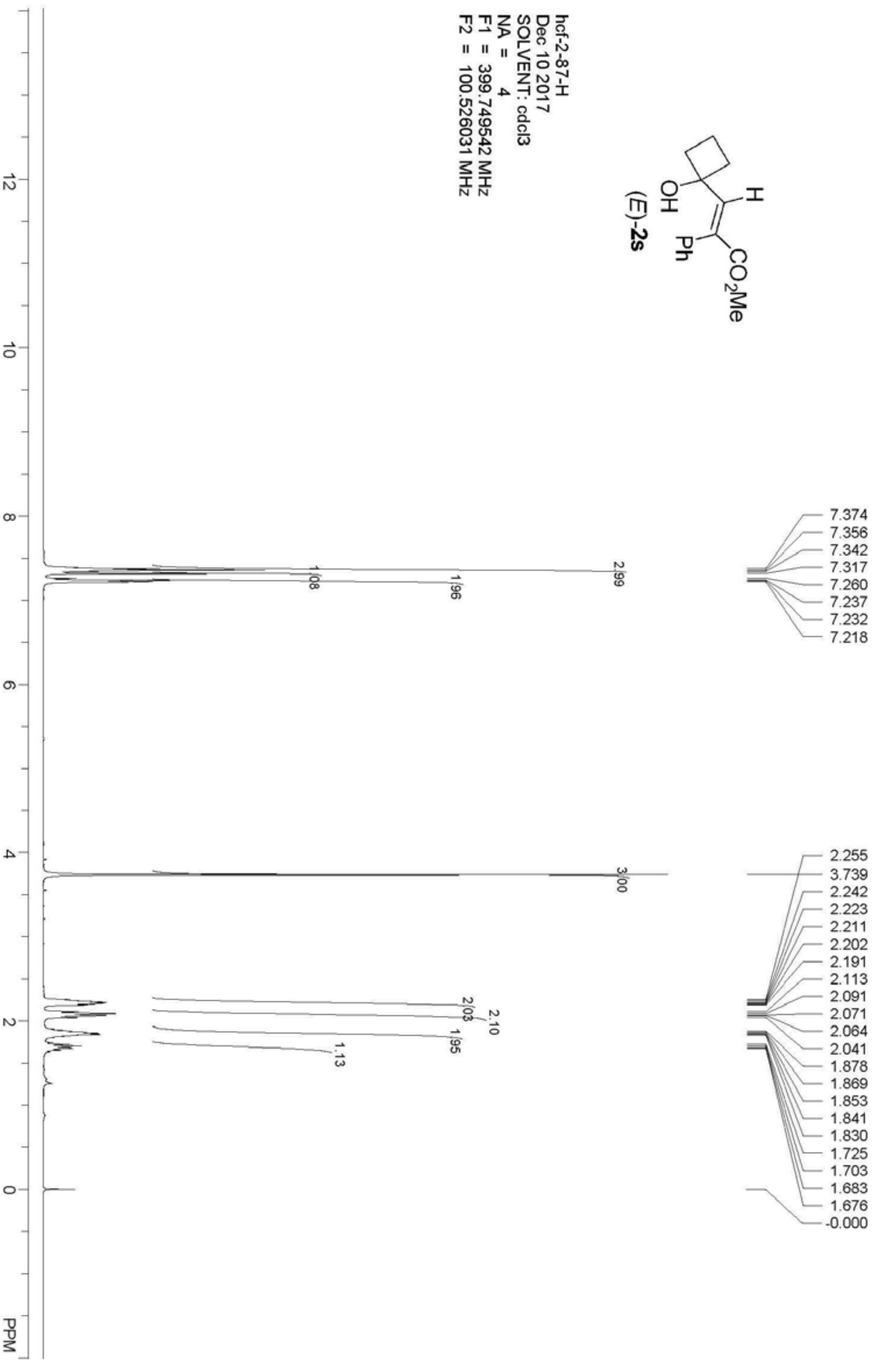

hcf-2-87-H
Dec 10 2017
SOLVENT: cdcl3
NA = 4
F1 = 399.749542 MHz
F2 = 100.526031 MHz
(E)-2s
7.374
7.356
7.342
7.317
7.260
7.237
7.232
7.218
2.255
3.739
2.242
2.223
2.211
2.202
2.191
2.113
2.091
2.071
2.064
2.041
1.878
1.869
1.853
1.841
1.830
1.725
1.703
1.683
1.676
-0.000
2.99
1.08
1.96
3.00
2.03
2.10
1.95
1.13
PPM

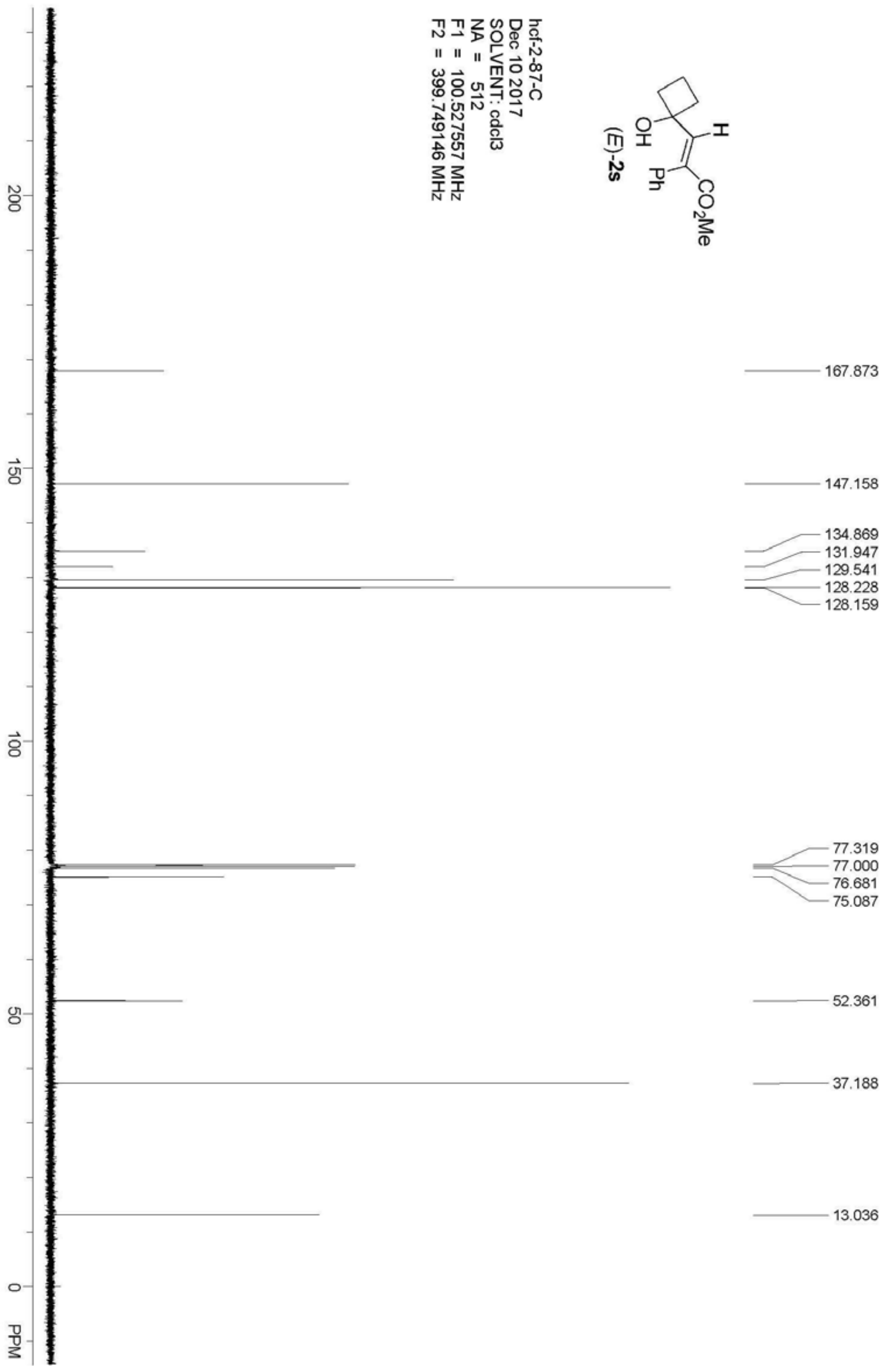
hcf-2-87-C
Dec 10 2017
SOLVENT: cdcl3
NA = 512
F1 = 100.527557 MHz
F2 = 399.749146 MHz
H
CO2Me
OH
Ph
(E)-2s
167.873
147.158
134.869
131.947
129.541
128.228
128.159
77.319
77.000
76.681
75.087
52.361
37.188
13.036
200
150
100
50
0
PPM

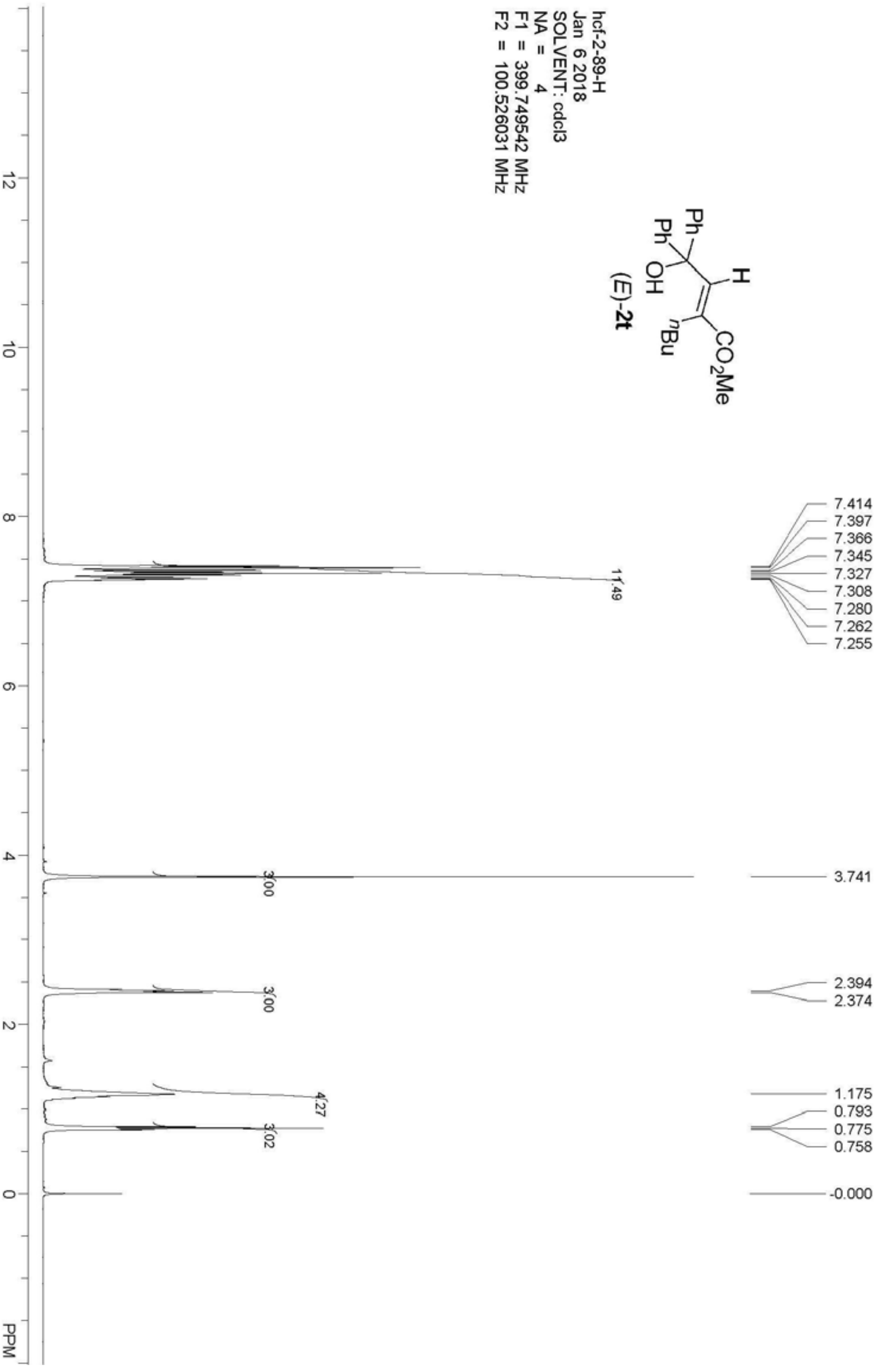
hcf-2-89-H
Jan 6 2018
SOLVENT: cdcl3
NA = 4
F1 = 399.749542 MHz
F2 = 100.526031 MHz
Ph
Ph
OH
H
CO2Me
nBu
(E)-2t
7.414
7.397
7.366
7.345
7.327
7.308
7.280
7.262
7.255
3.741
2.394
2.374
1.175
0.793
0.775
0.758
-0.000
11.49
3.00
3.00
4.27
3.02
12
10
8
6
4
2
0
PPM

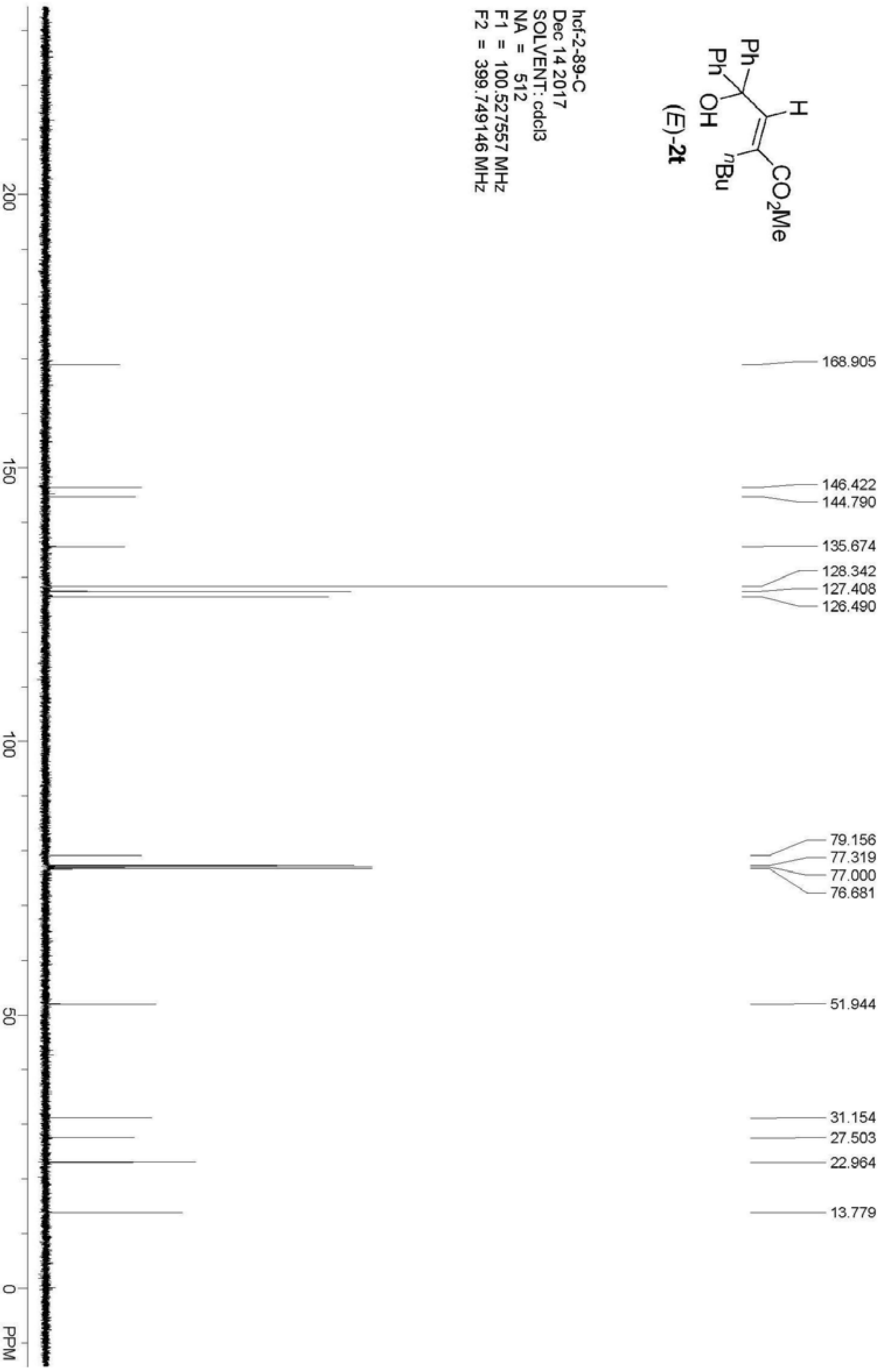
Ph
Ph OH
H
CO2Me
nBu
(E)-2t
hcf-2-89-C
Dec 14 2017
SOLVENT: cdcl3
NA = 512
F1 = 100.527557 MHz
F2 = 399.749146 MHz
168.905
146.422
144.790
135.674
128.342
127.408
126.490
79.156
77.319
77.000
76.681
51.944
31.154
27.503
22.964
13.779
200
150
100
50
0
PPM

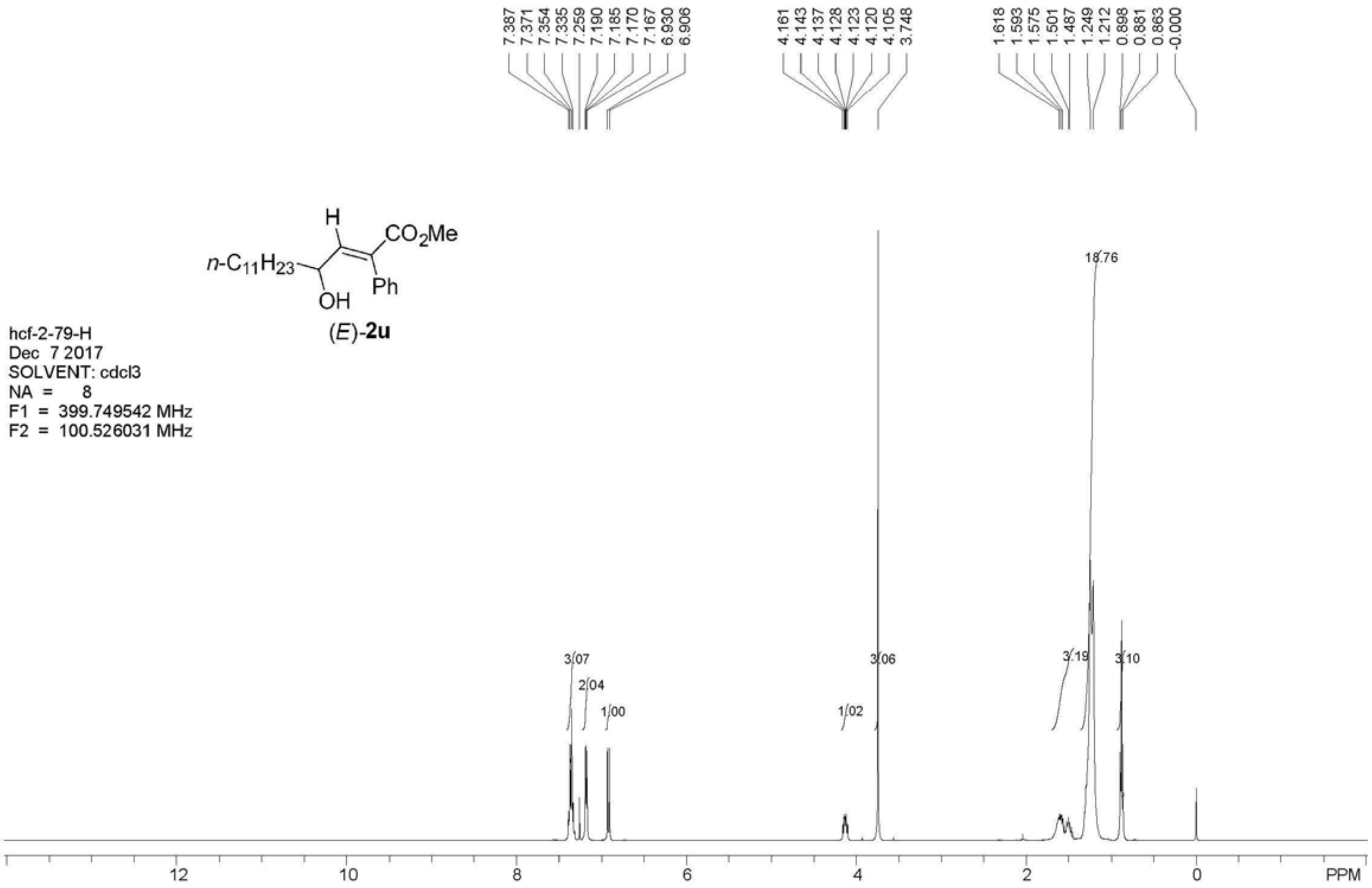

7.387
7.371
7.354
7.335
7.259
7.190
7.185
7.170
7.167
6.930
6.906
4.161
4.143
4.137
4.128
4.123
4.120
4.105
3.748
1.618
1.593
1.575
1.501
1.487
1.249
1.212
0.898
0.881
0.863
-0.000
H
CO2Me
n-C11H23
OH
Ph
(E)-2u
hcf-2-79-H
Dec 7 2017
SOLVENT: cdcl3
NA = 8
F1 = 399.749542 MHz
F2 = 100.526031 MHz
18.76
3.07
2.04
1.00
1.02
3.06
3.19
3.10
12
10
8
6
4
2
0
PPM

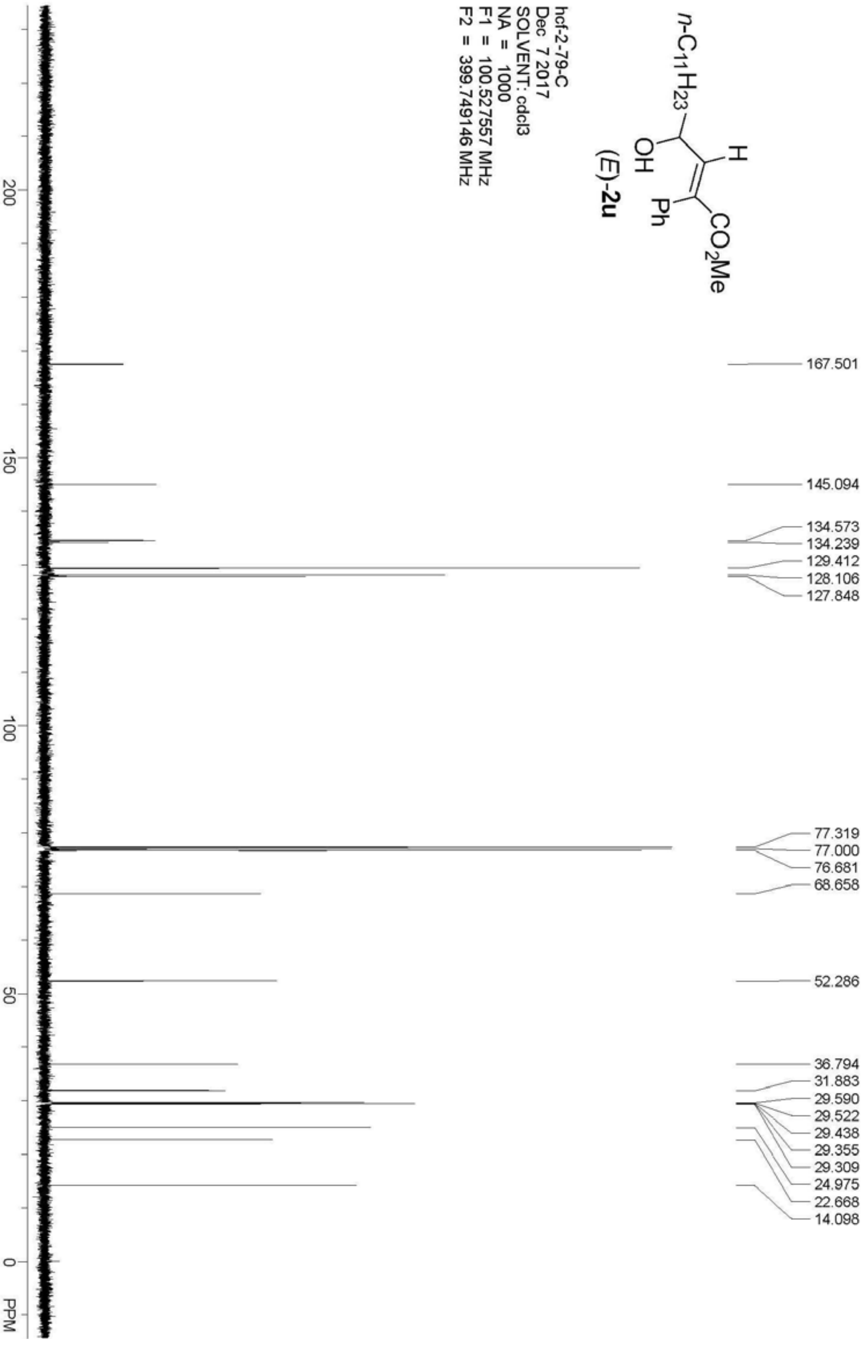

n-C11H23
H
OH
Ph
CO2Me
(E)-2u
hcf-2-79-C
Dec 7 2017
SOLVENT: cdcl3
NA = 1000
F1 = 100.527557 MHz
F2 = 399.749146 MHz
167.501
145.094
134.573
134.239
129.412
128.106
127.848
77.319
77.000
76.681
68.658
52.286
36.794
31.883
29.590
29.522
29.438
29.355
29.309
24.975
22.668
14.098
200
150
100
50
0
PPM

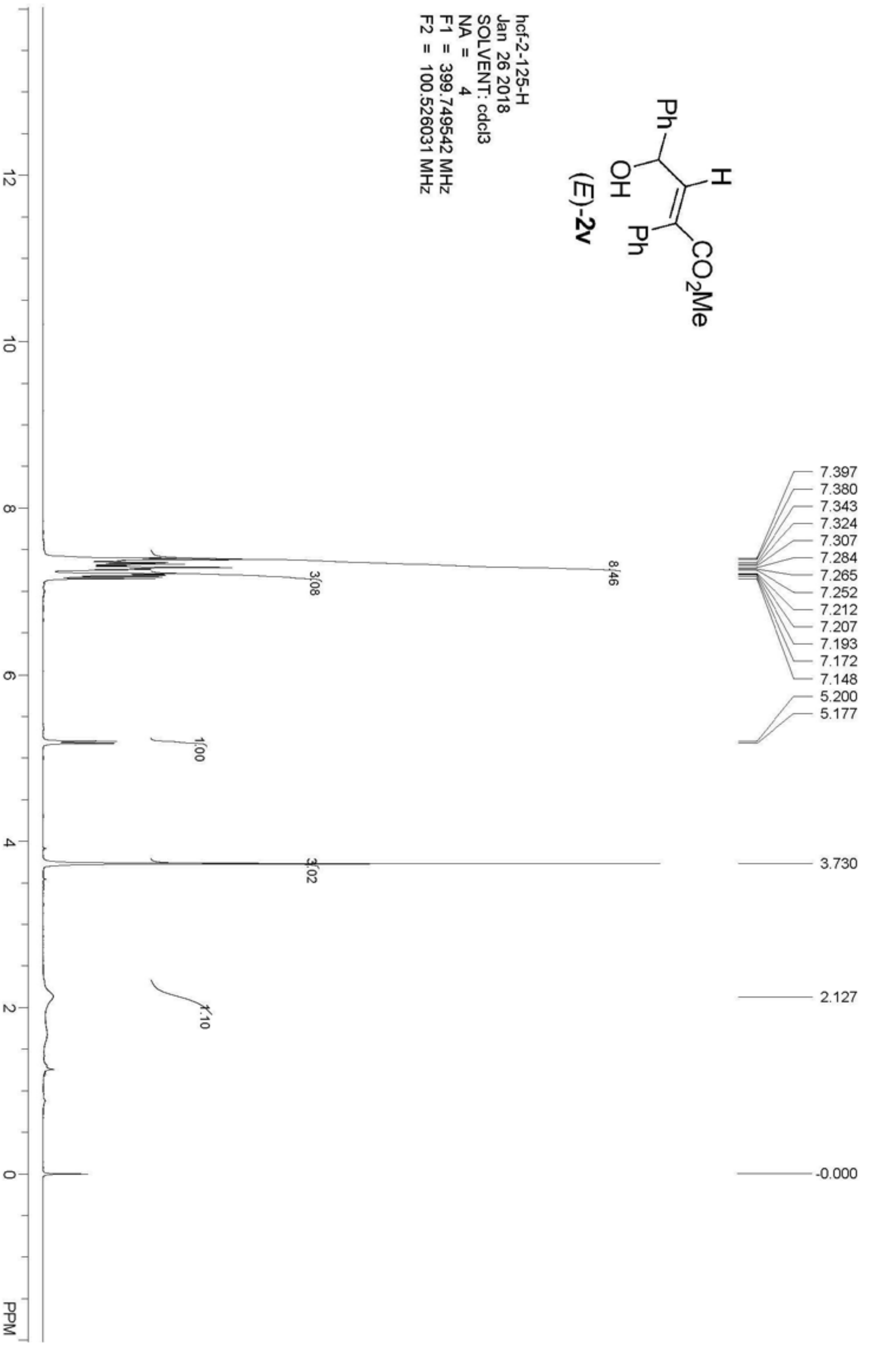
(E)-2v
hcf-2-125-H
Jan 26 2018
SOLVENT: cdcl3
NA = 4
F1 = 399.749542 MHz
F2 = 100.526031 MHz
7.397
7.380
7.343
7.324
7.307
7.284
7.265
7.252
7.212
7.207
7.193
7.172
7.148
5.200
5.177
3.730
2.127
-0.000
8.46
3.08
1.00
3.02
1.10
PPM

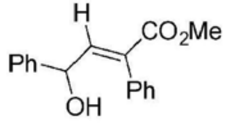

(*E*)-**2v**

hcf-2-125-C
Jan 26 2018
SOLVENT: cdcl3
NA = 512
F1 = 100.527557 MHz
F2 = 399.749146 MHz

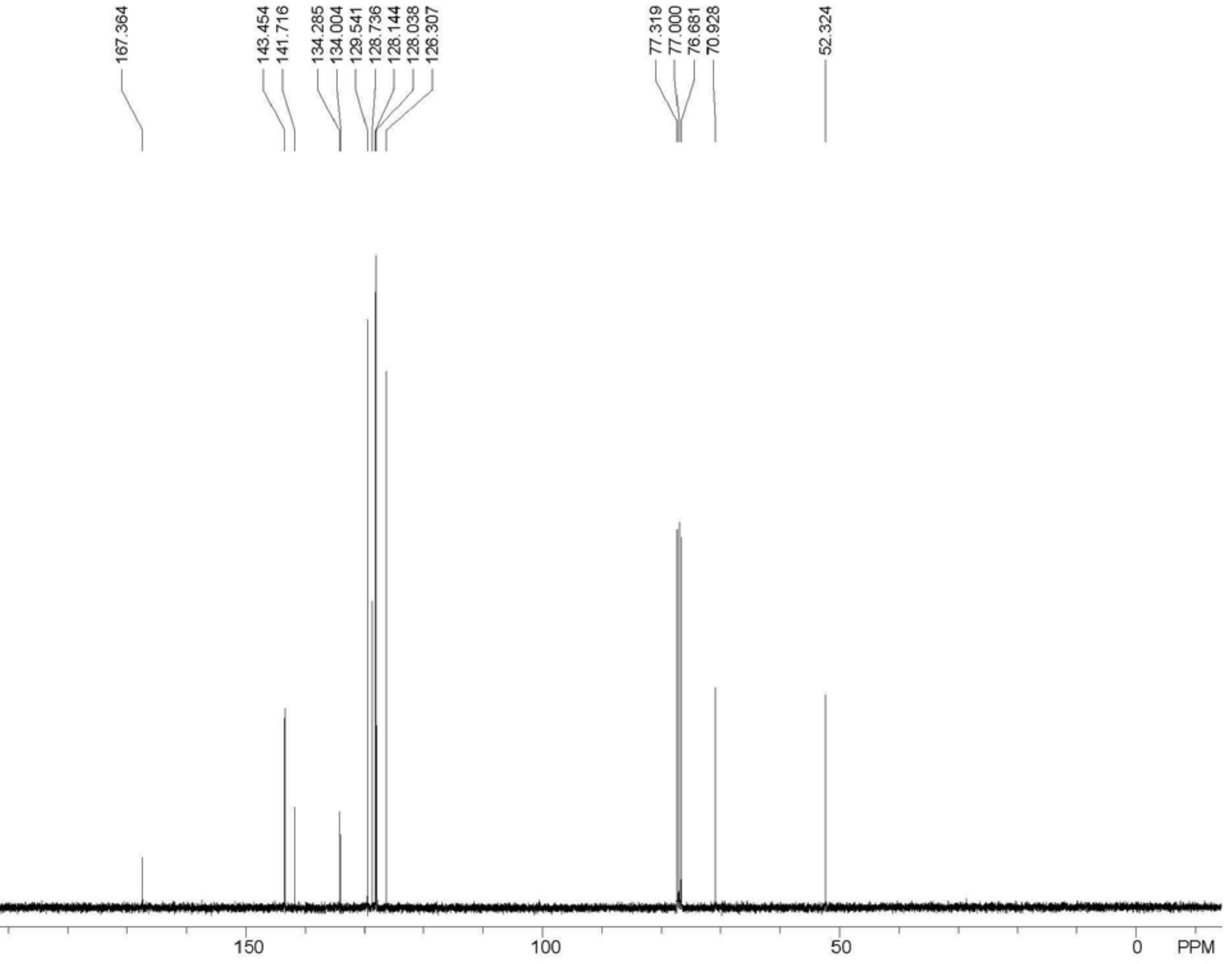

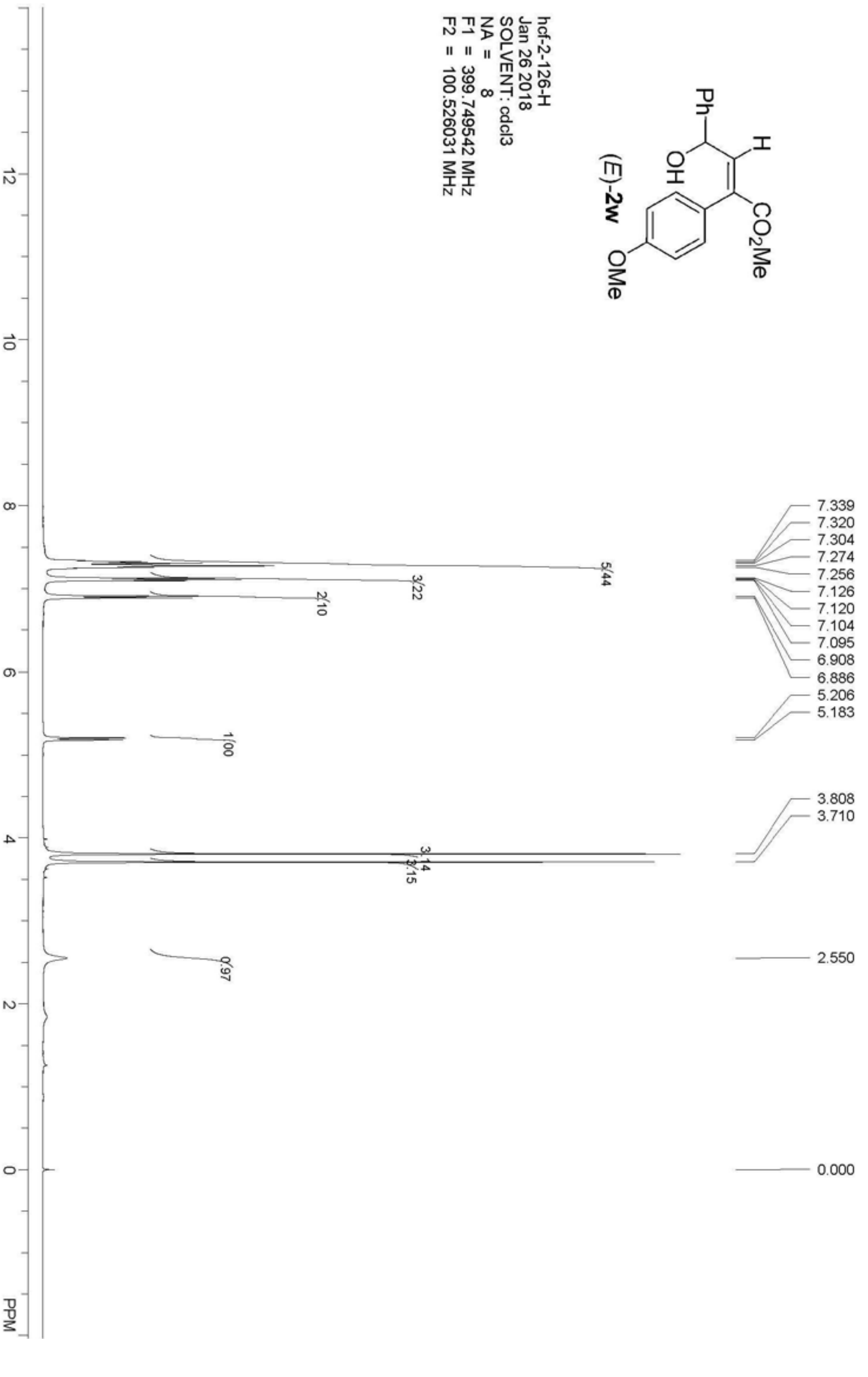

(E)-2w
Ph
OH
H
CO2Me
OMe
hcf-2-126-H
Jan 26 2018
SOLVENT: cdcl3
NA = 8
F1 = 399.749542 MHz
F2 = 100.526031 MHz
7.339
7.320
7.304
7.274
7.256
7.126
7.120
7.104
7.095
6.908
6.886
5.206
5.183
3.808
3.710
2.550
0.000
5.44
3.22
2.10
1.00
3.14
3.15
0.97
PPM

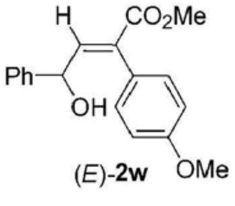

(*E*)-**2w**

hcf-2-126-C
Jan 29 2018
SOLVENT: cdcl3
NA = 512
F1 = 100.527557 MHz
F2 = 399.749146 MHz

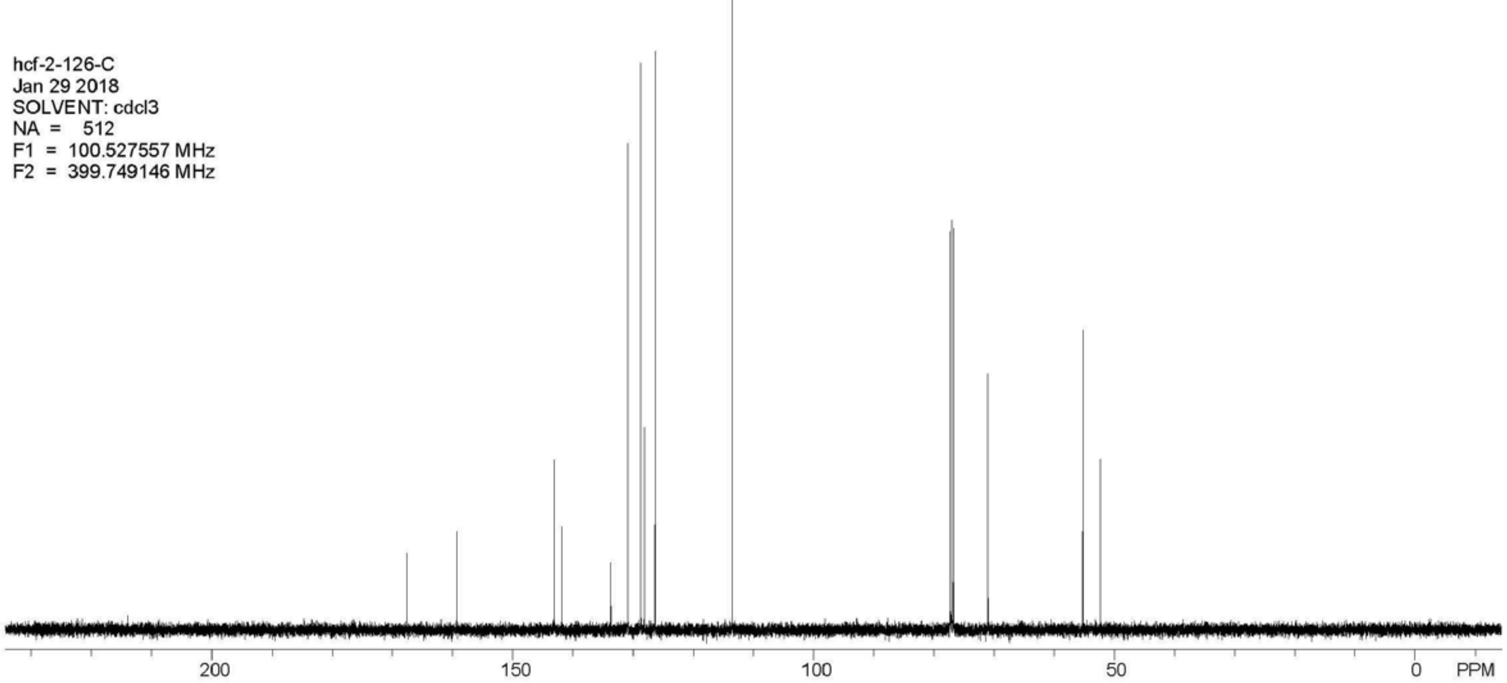

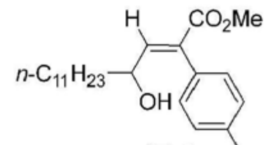
n-C11H23
OH
H
CO2Me
Br

(*E*)-**2x**

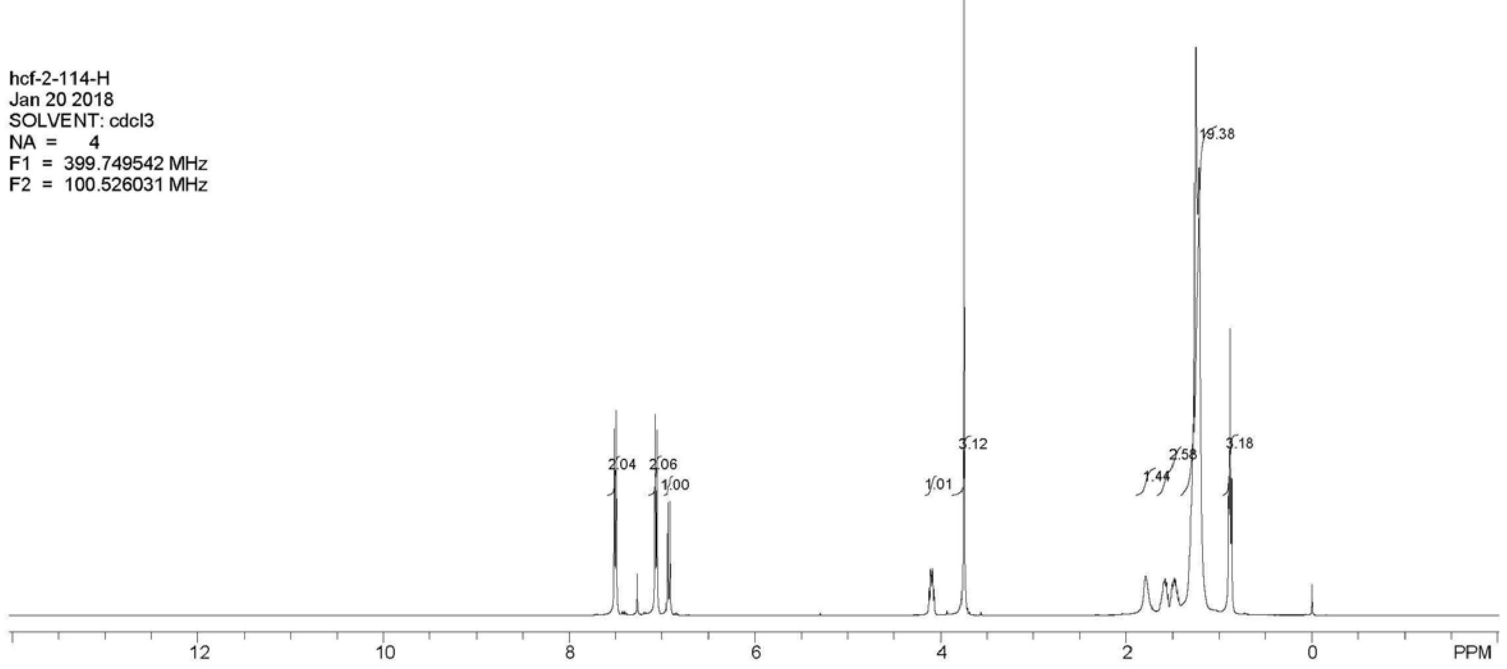
hcf-2-114-H
Jan 20 2018
SOLVENT: cdcl3
NA = 4
F1 = 399.749542 MHz
F2 = 100.526031 MHz
7.511
7.491
7.265
7.074
7.053
6.940
6.916
4.123
4.105
4.085
4.067
3.749
1.582
1.566
1.547
1.790
1.506
1.485
1.471
1.450
1.435
1.253
1.218
0.897
0.881
0.863
-0.000
2.04
2.06
1.00
1.01
3.12
1.44
2.58
19.38
3.18
12
10
8
6
4
2
0
PPM

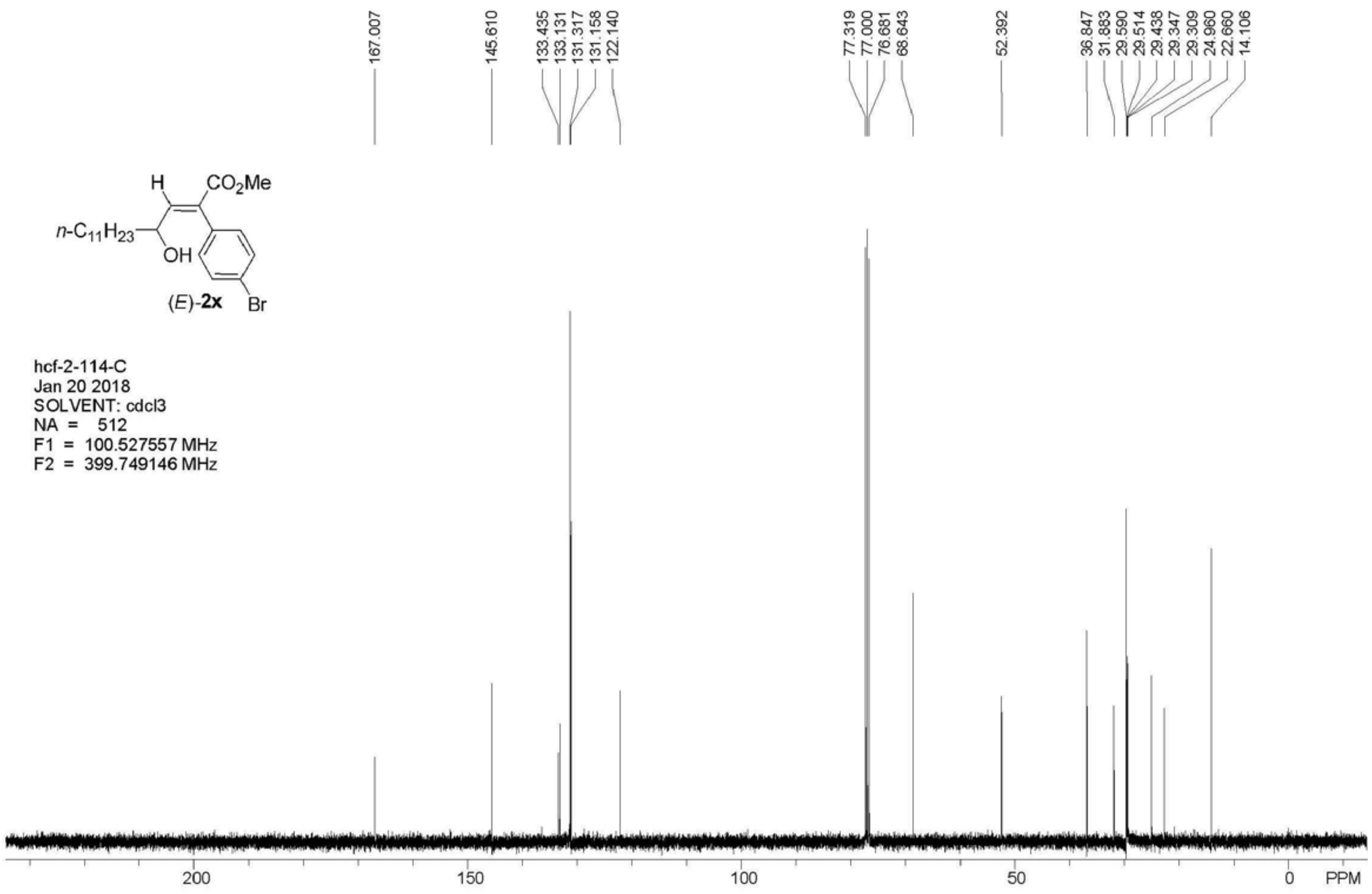
CO2Me
H
n-C11H23
OH
Br
(E)-2x
hcf-2-114-C
Jan 20 2018
SOLVENT: cdcl3
NA = 512
F1 = 100.527557 MHz
F2 = 399.749146 MHz
167.007
145.610
133.435
133.131
131.317
131.158
122.140
77.319
77.000
76.681
68.643
52.392
36.847
31.883
29.590
29.514
29.438
29.347
29.309
24.960
22.660
14.106
200
150
100
50
0
PPM

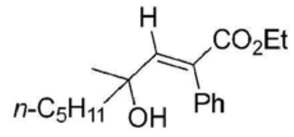
H
CO_2Et
n-C_5H_{11}
OH
Ph

(E)-**2ka**

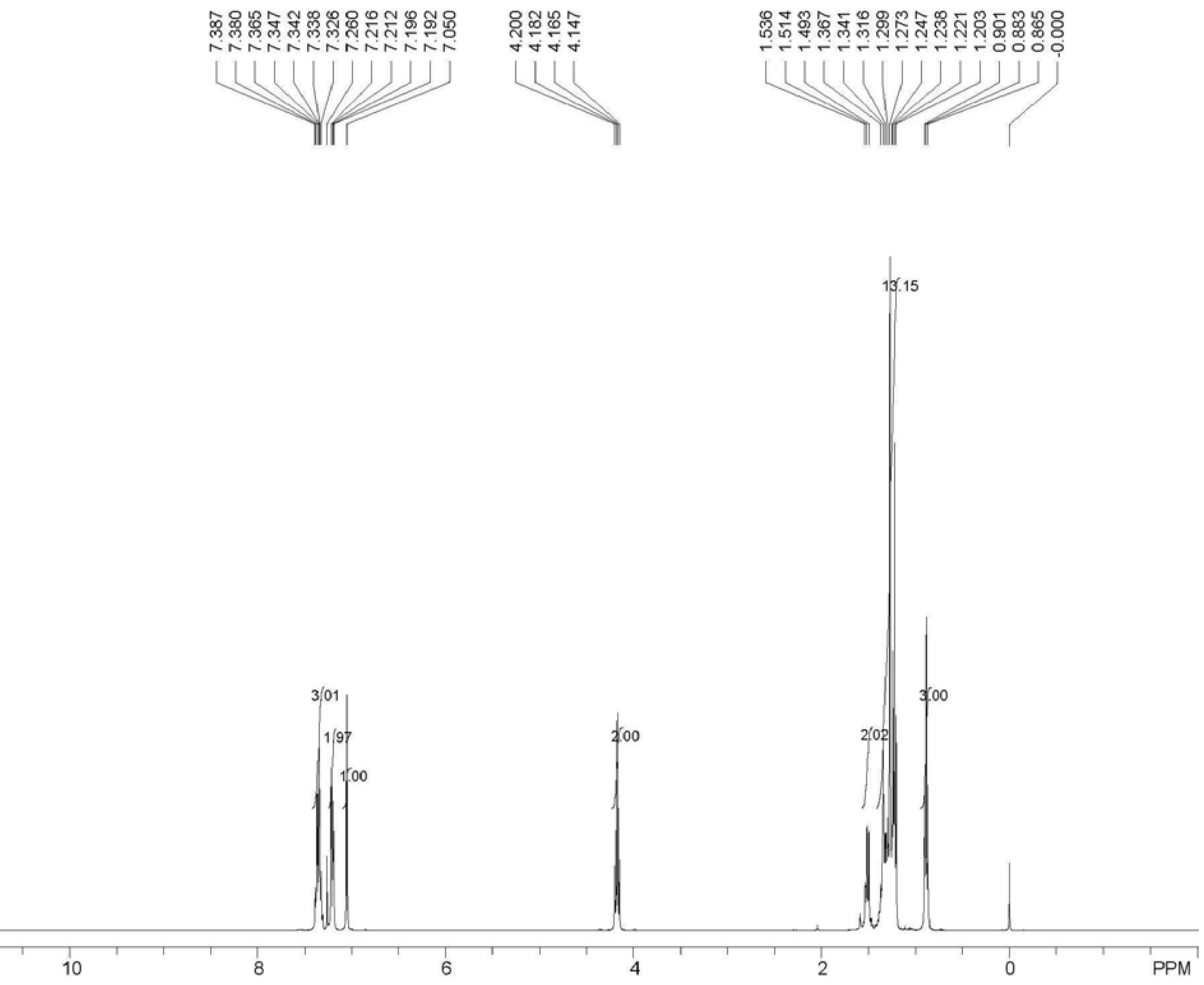
hcf-2-76-H
Dec 5 2017
SOLVENT: cdcl3
NA = 4
F1 = 399.749542 MHz
F2 = 100.526031 MHz
7.387
7.380
7.365
7.347
7.342
7.338
7.326
7.260
7.216
7.212
7.196
7.192
7.050
4.200
4.182
4.165
4.147
1.536
1.514
1.493
1.367
1.341
1.316
1.299
1.273
1.247
1.238
1.221
1.203
0.901
0.883
0.865
-0.000
3.01
1.97
1.00
2.00
2.02
13.15
3.00
12
10
8
6
4
2
0
PPM

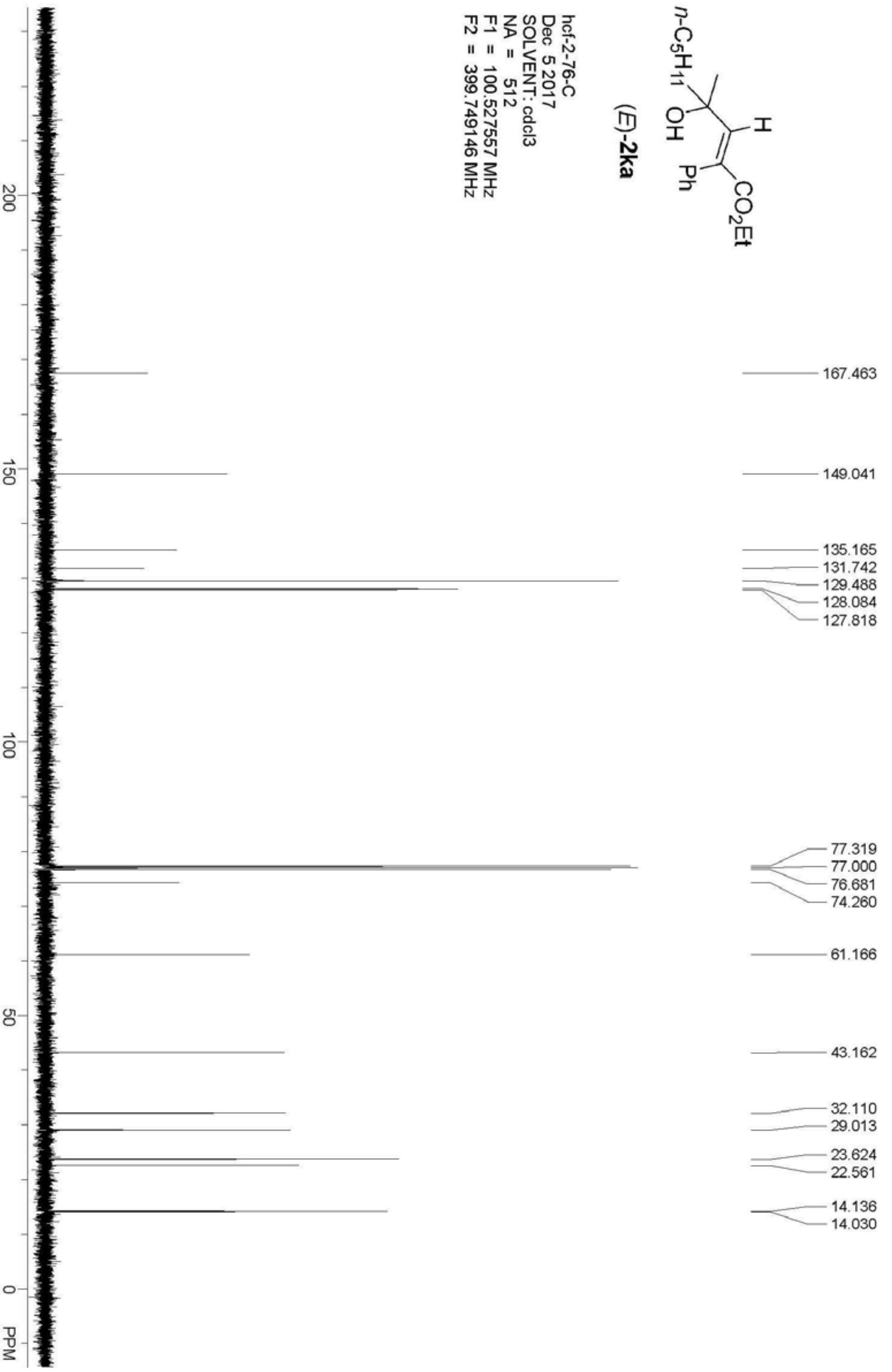
n-C5H11
OH
H
Ph
CO2Et
(E)-2ka
hcf-2-76-C
Dec 5 2017
SOLVENT: cdcl3
NA = 512
F1 = 100.527557 MHz
F2 = 399.749146 MHz
167.463
149.041
135.165
131.742
129.488
128.084
127.818
77.319
77.000
76.681
74.260
61.166
43.162
32.110
29.013
23.624
22.561
14.136
14.030
200
150
100
50
0
PPM

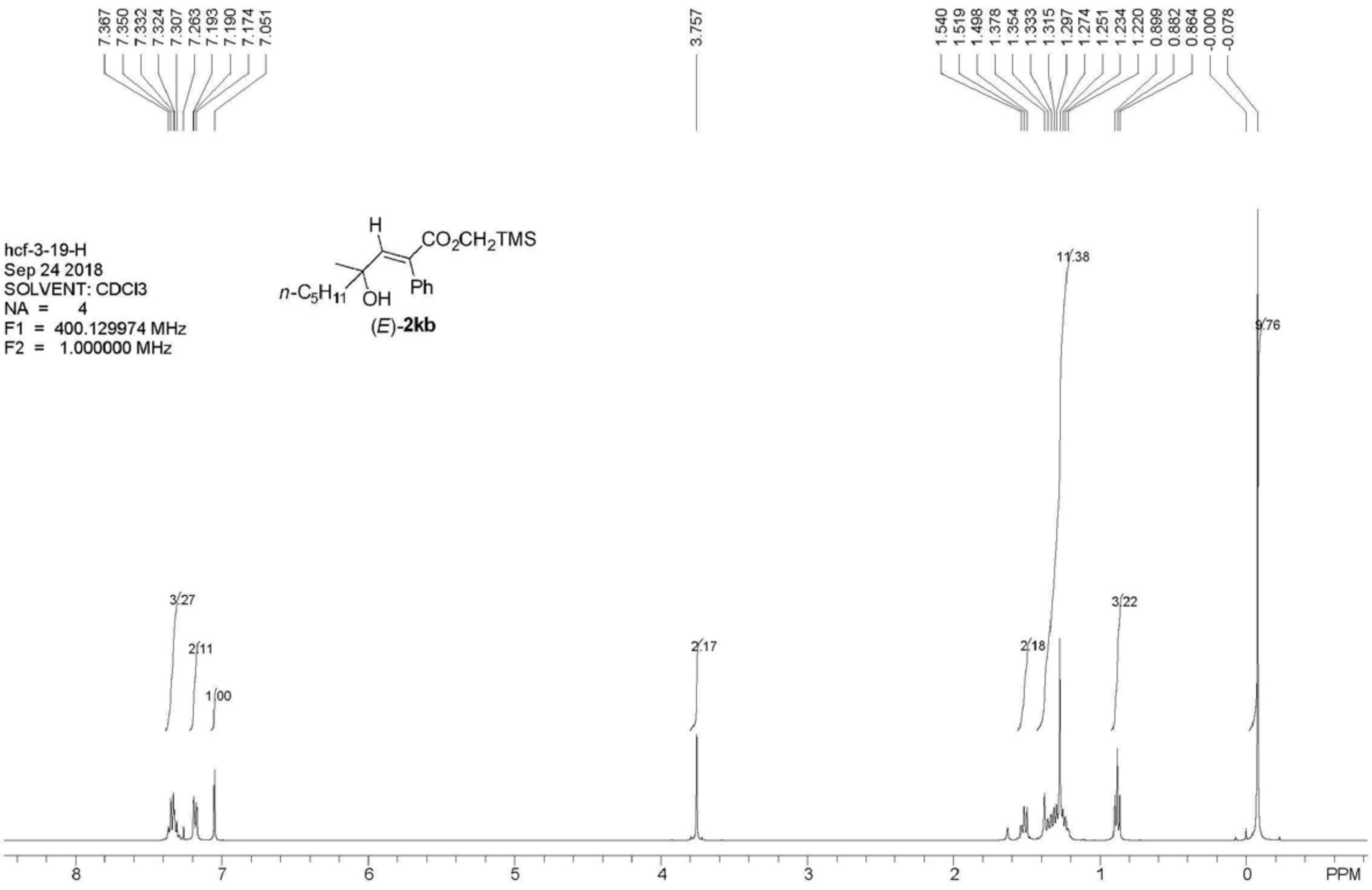
7.367
7.350
7.332
7.324
7.307
7.263
7.193
7.190
7.174
7.051
3.757
1.540
1.519
1.498
1.378
1.354
1.333
1.315
1.297
1.274
1.251
1.234
1.220
0.899
0.882
0.864
-0.000
-0.078
hcf-3-19-H
Sep 24 2018
SOLVENT: CDCl3
NA = 4
F1 = 400.129974 MHz
F2 = 1.000000 MHz
3.27
2.11
1.00
2.17
2.18
11.38
3.22
9.76
8
7
6
5
4
3
2
1
0
PPM
H
CO_2CH_2TMS
n-C_5H_{11}
OH
Ph

(E)-**2kb**

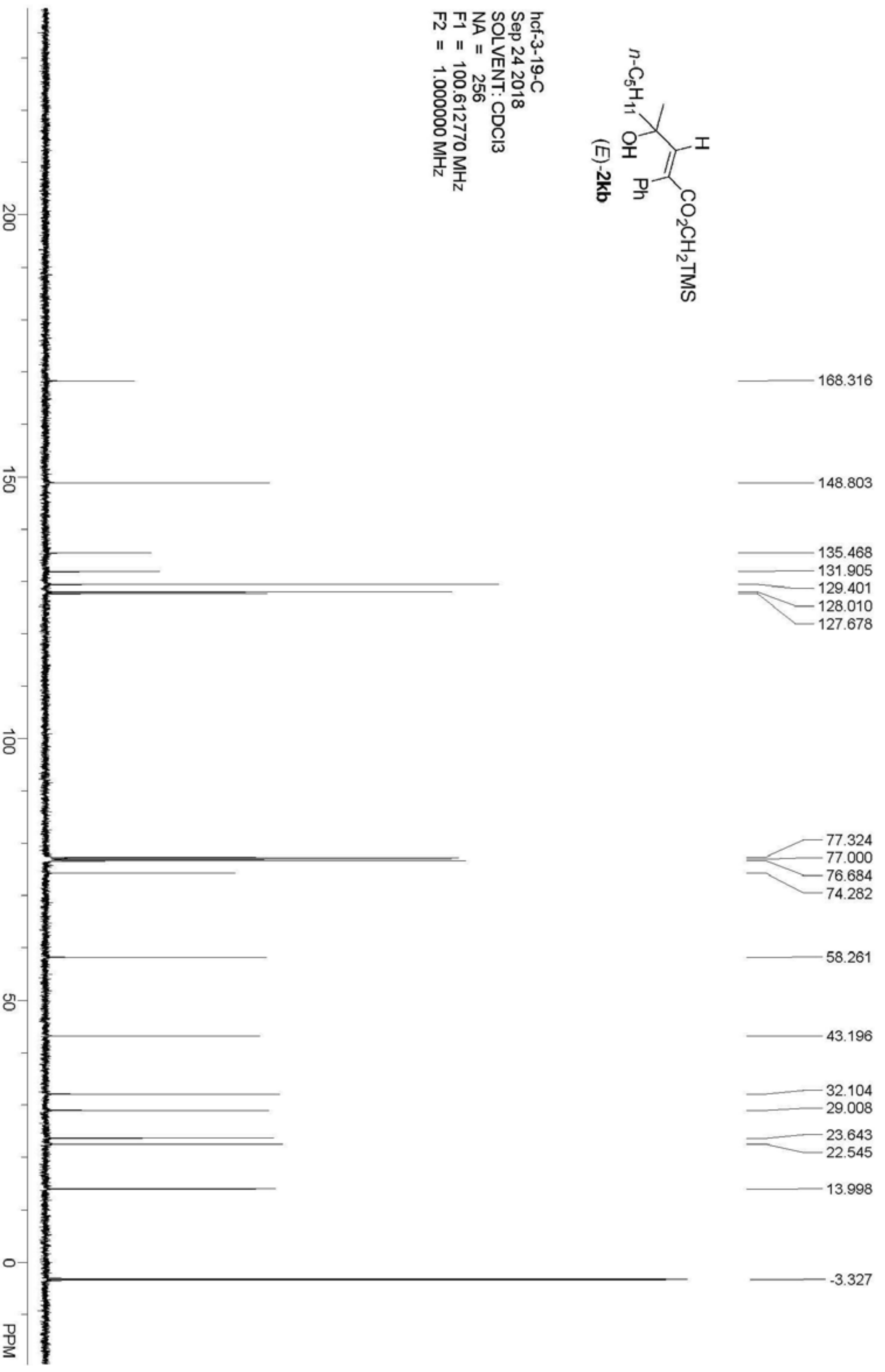
hcf-3-19-C
Sep 24 2018
SOLVENT: CDCl3
NA = 256
F1 = 100.612770 MHz
F2 = 1.000000 MHz
H
CO2CH2TMS
n-C5H11
OH
Ph
(E)-2kb
168.316
148.803
135.468
131.905
129.401
128.010
127.678
77.324
77.000
76.684
74.282
58.261
43.196
32.104
29.008
23.643
22.545
13.998
-3.327
200
150
100
50
0
PPM

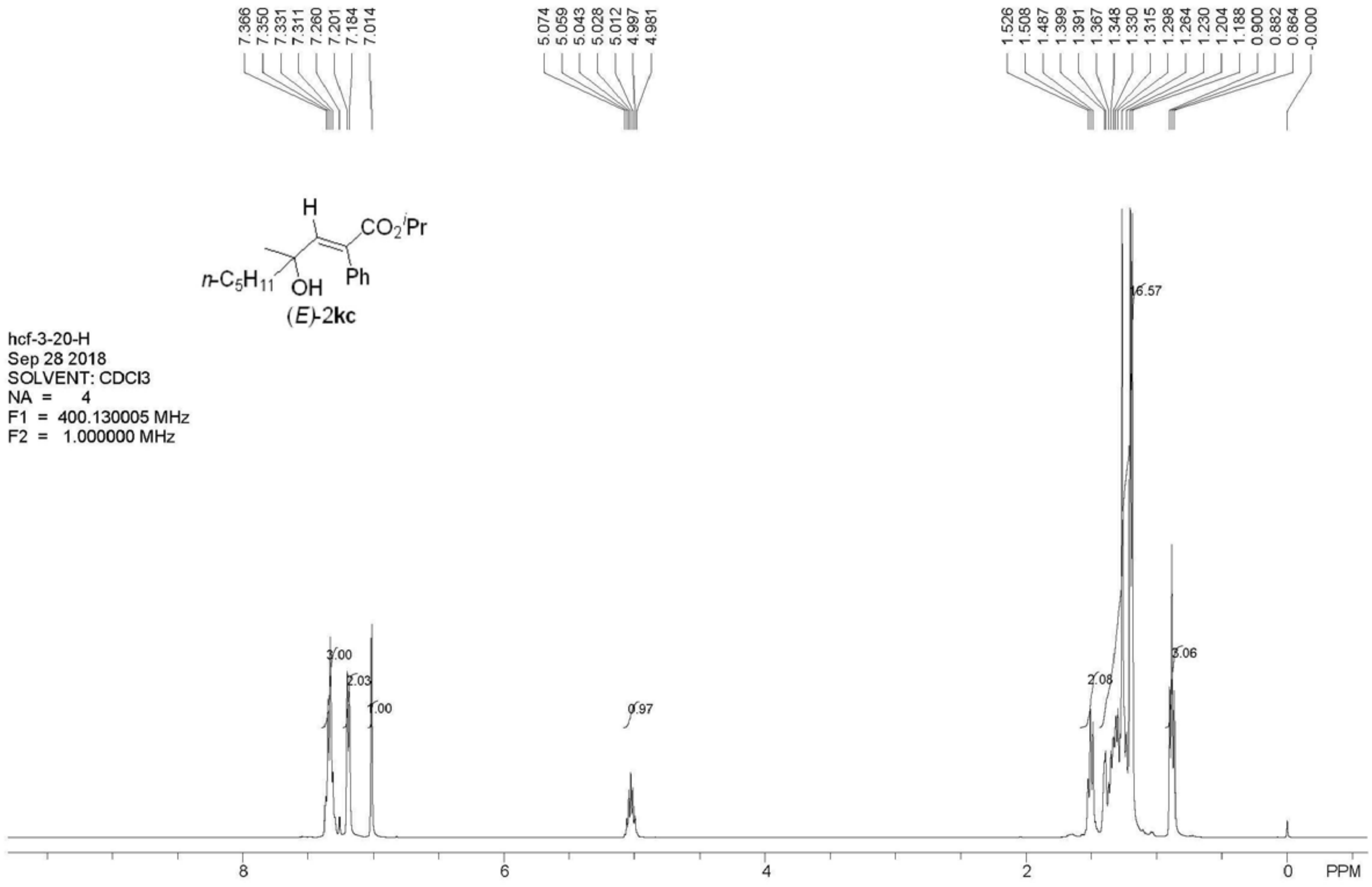
7.366
7.350
7.331
7.311
7.260
7.201
7.184
7.014
5.074
5.059
5.043
5.028
5.012
4.997
4.981
1.526
1.508
1.487
1.399
1.391
1.367
1.348
1.330
1.315
1.298
1.264
1.230
1.204
1.188
0.900
0.882
0.864
-0.000
H
$CO_2{}^iPr$
n-C_5H_{11}
OH
Ph
(E)-2**kc**
hcf-3-20-H
Sep 28 2018
SOLVENT: CDCl3
NA = 4
F1 = 400.130005 MHz
F2 = 1.000000 MHz
3.00
2.03
1.00
0.97
2.08
16.57
3.06
8
6
4
2
0
PPM

(E)-2**kc**

hcf-3-20-C
Sep 28 2018
SOLVENT: CDCl3
NA = 128
F1 = 100.612770 MHz
F2 = 1.000000 MHz

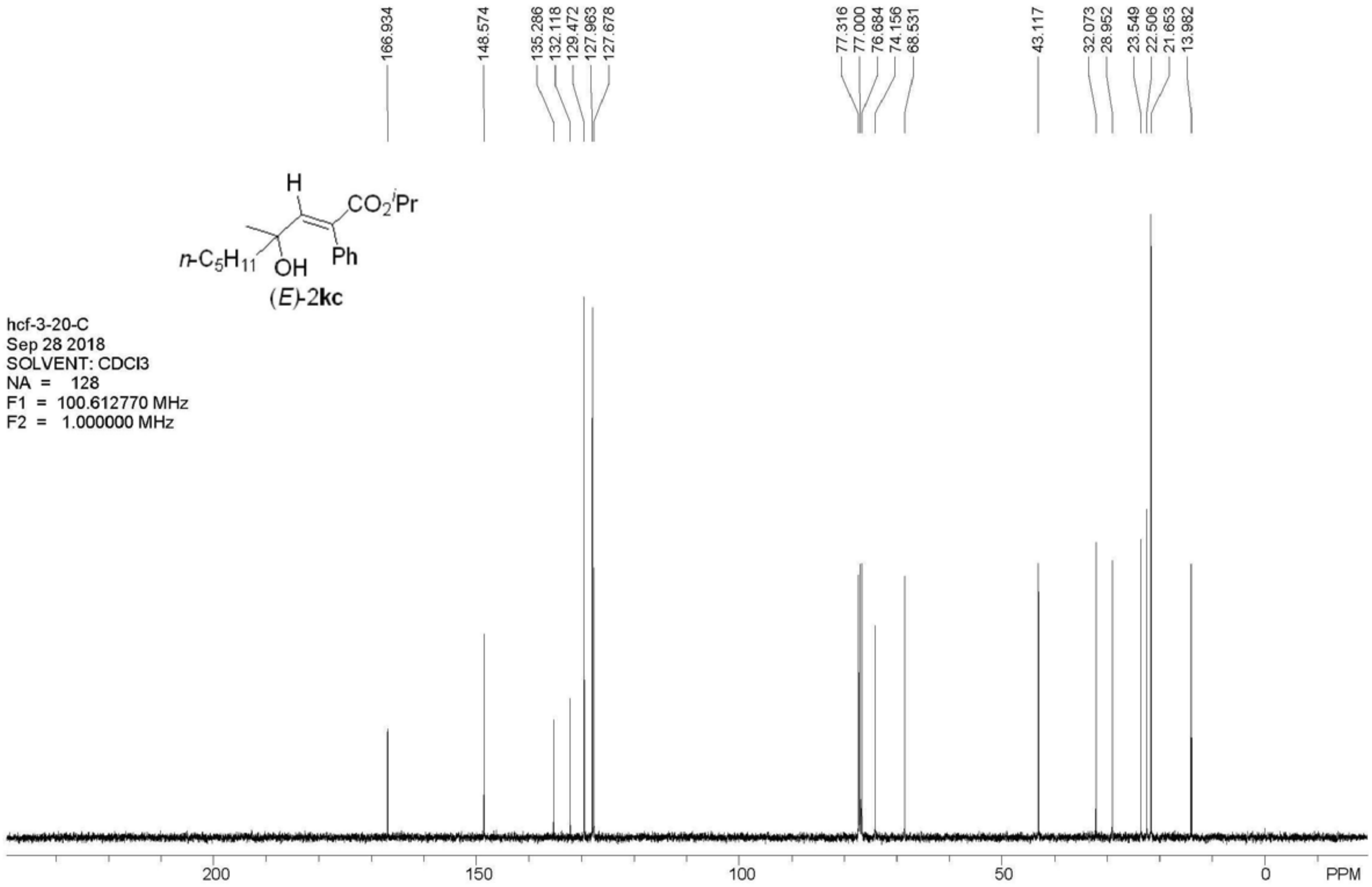

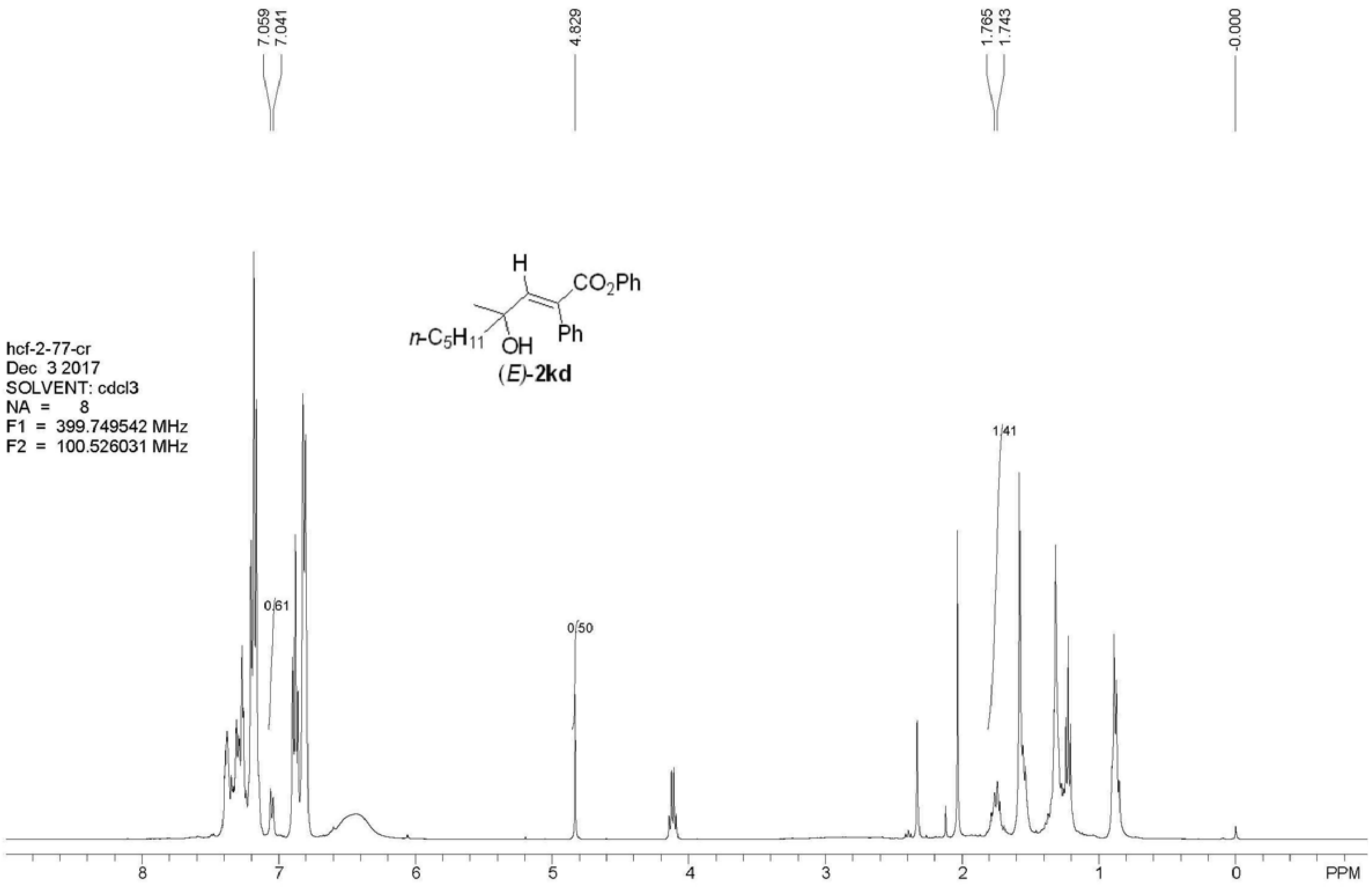

7.059
7.041
4.829
1.765
1.743
-0.000
hcf-2-77-cr
Dec 3 2017
SOLVENT: cdcl3
NA = 8
F1 = 399.749542 MHz
F2 = 100.526031 MHz
H
CO_2Ph
n-C_5H_{11}
OH
Ph
(*E*)-**2kd**
0.61
0.50
1.41
8
7
6
5
4
3
2
1
0
PPM

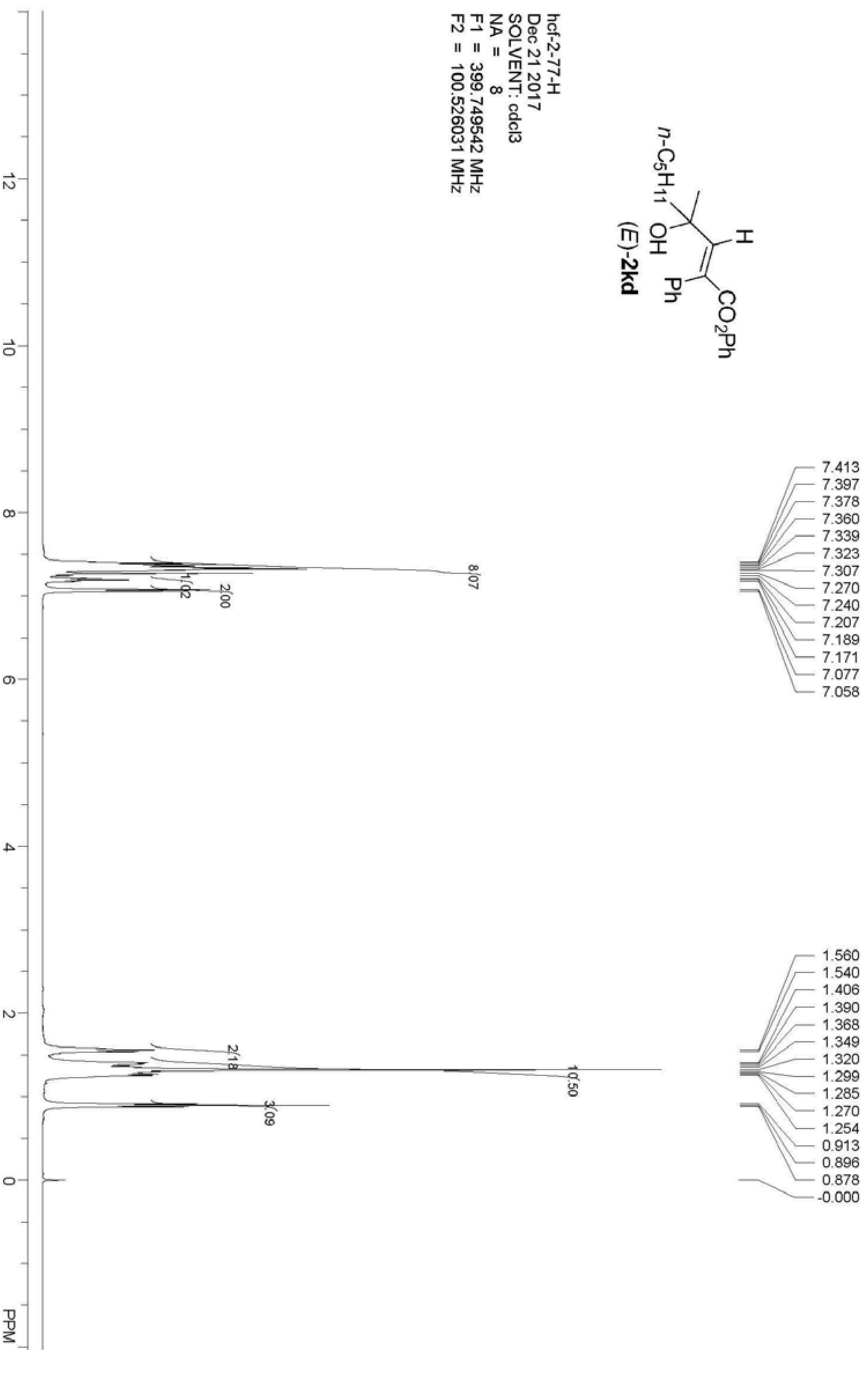
hcf-2-77-H
Dec 21 2017
SOLVENT: cdcl3
NA = 8
F1 = 399.749542 MHz
F2 = 100.526031 MHz
n-C5H11
OH
H
Ph
CO2Ph
(E)-2kd
7.413
7.397
7.378
7.360
7.339
7.323
7.307
7.270
7.240
7.207
7.189
7.171
7.077
7.058
1.560
1.540
1.406
1.390
1.368
1.349
1.320
1.299
1.285
1.270
1.254
0.913
0.896
0.878
-0.000
8.07
1.02
2.00
2.18
10.50
3.09
PPM

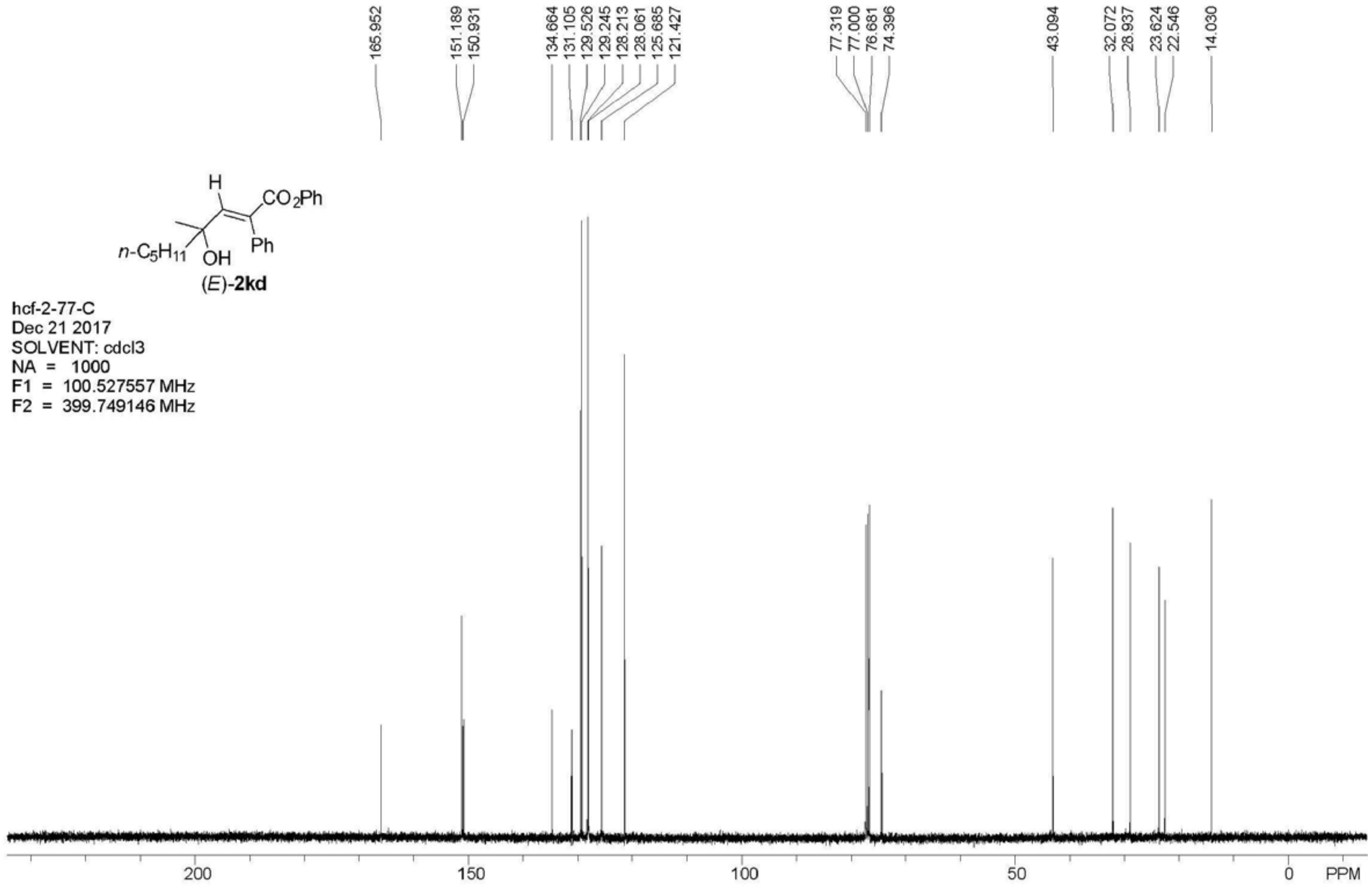
H
CO_2Ph
Ph
n-C_5H_{11}
OH
(E)-**2kd**
hcf-2-77-C
Dec 21 2017
SOLVENT: cdcl3
NA = 1000
F1 = 100.527557 MHz
F2 = 399.749146 MHz
165.952
151.189
150.931
134.664
131.105
129.526
129.245
128.213
128.061
125.685
121.427
77.319
77.000
76.681
74.396
43.094
32.072
28.937
23.624
22.546
14.030
200
150
100
50
0
PPM

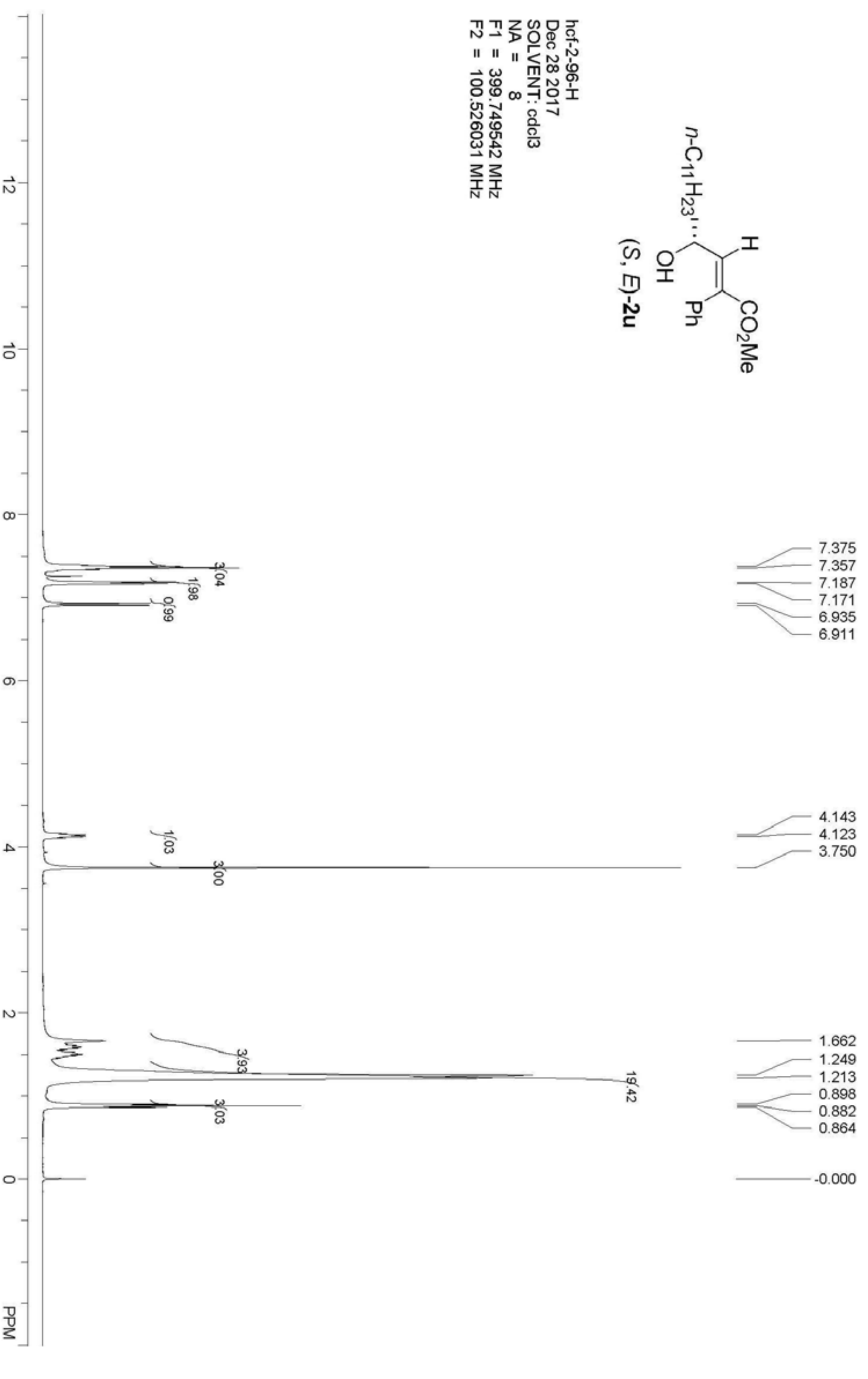

hcf-2-96-H
Dec 28 2017
SOLVENT: cdcl3
NA = 8
F1 = 399.749542 MHz
F2 = 100.526031 MHz
n-C11H23
H
CO2Me
OH
Ph
(S, E)-2u
7.375
7.357
7.187
7.171
6.935
6.911
4.143
4.123
3.750
1.662
1.249
1.213
0.898
0.882
0.864
-0.000
3.04
1.98
0.99
1.03
3.00
3.93
19.42
3.03
PPM

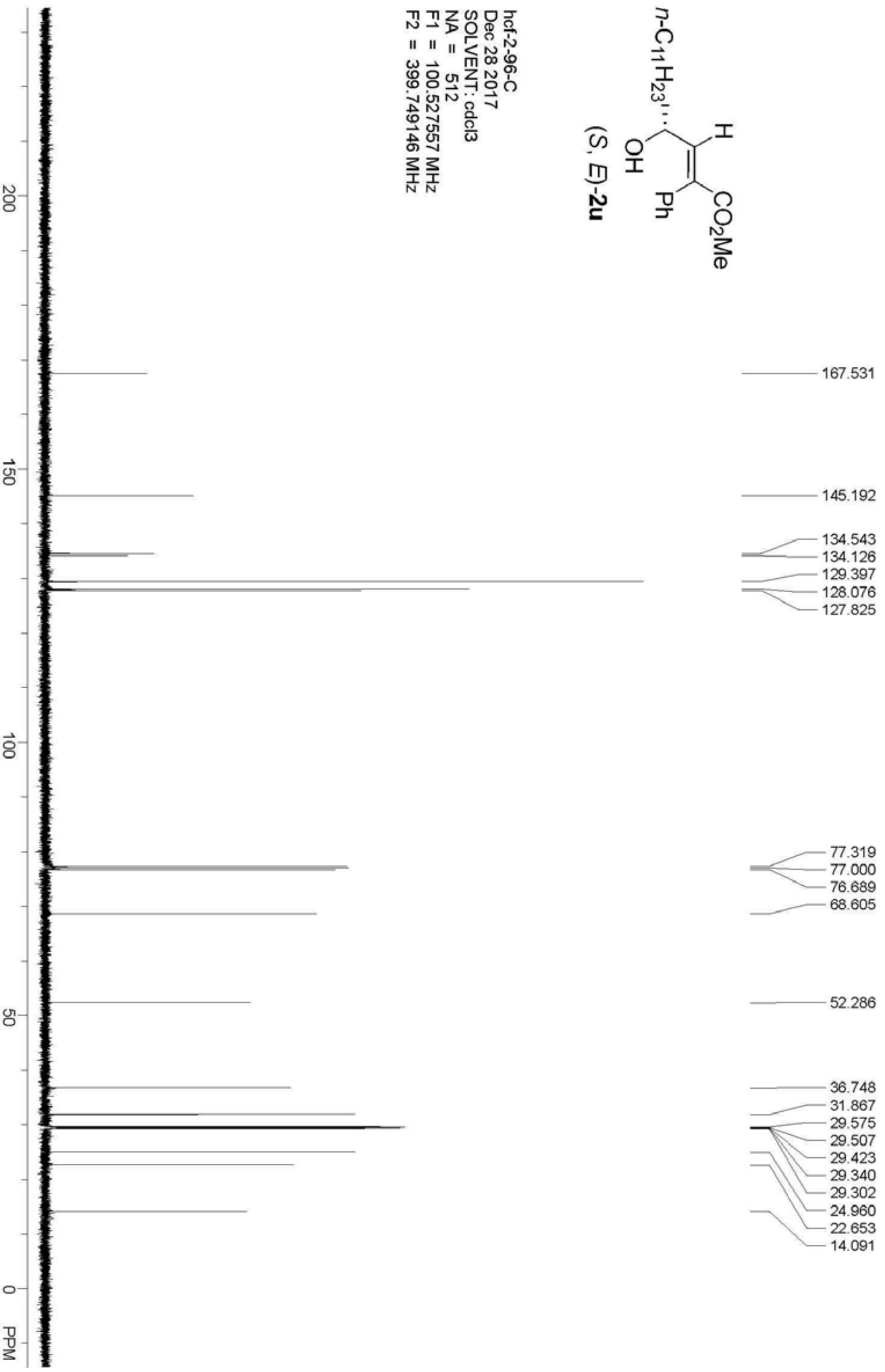

hcf-2-96-C
Dec 28 2017
SOLVENT: cdcl3
NA = 512
F1 = 100.527557 MHz
F2 = 399.749146 MHz
(S, E)-**2u**
167.531
145.192
134.543
134.126
129.397
128.076
127.825
77.319
77.000
76.689
68.605
52.286
36.748
31.867
29.575
29.507
29.423
29.340
29.302
24.960
22.653
14.091
PPM

hcf-2-79-pa-2-95-5-1-214

实验时间：2018-01-04, 17:07:27　　　　报告时间：2018-01-04, 17:48:59
谱图文件:D:\zhuguangjiong\hcf\20180104\hcf-2-79-pa-2-95-5-1-214.org

实验内容简介：

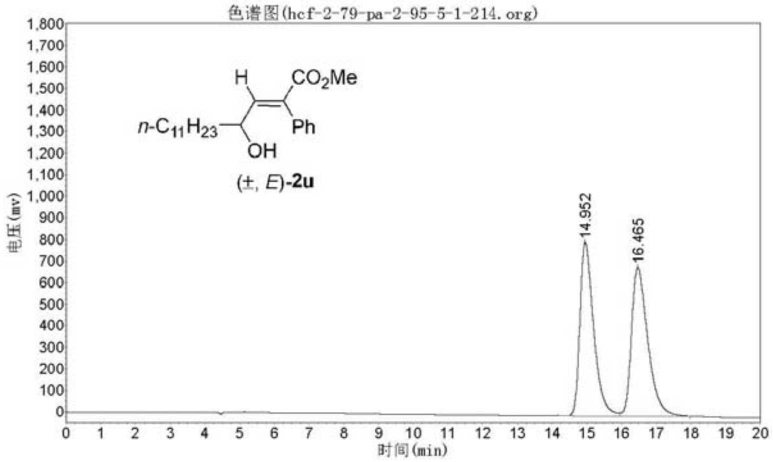

分析结果表

峰号	峰名	保留时间	峰高	峰面积	含量
1		14.952	806185.750	22177016.000	49.5640
2		16.465	689829.250	22567178.000	50.4360
总计			1496015.000	44744194.000	100.0000

hcf-2-96-pa-2-95-5-1-214

实验时间：2018-01-04, 17:28:11　　　　报告时间：2018-01-04, 17:50:52
谱图文件:D:\zhuguangjiong\hcf\20180104\hcf-2-96-pa-2-95-5-1-214.org

实验内容简介：

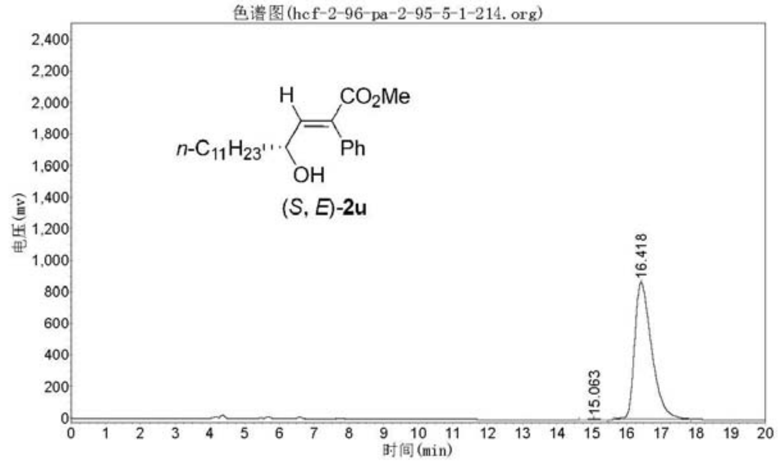

分析结果表

峰号	峰名	保留时间	峰高	峰面积	含量
1		15.063	3885.598	95790.781	0.3263
2		16.418	864572.563	29259652.000	99.6737
总计			868458.160	29355442.781	100.0000

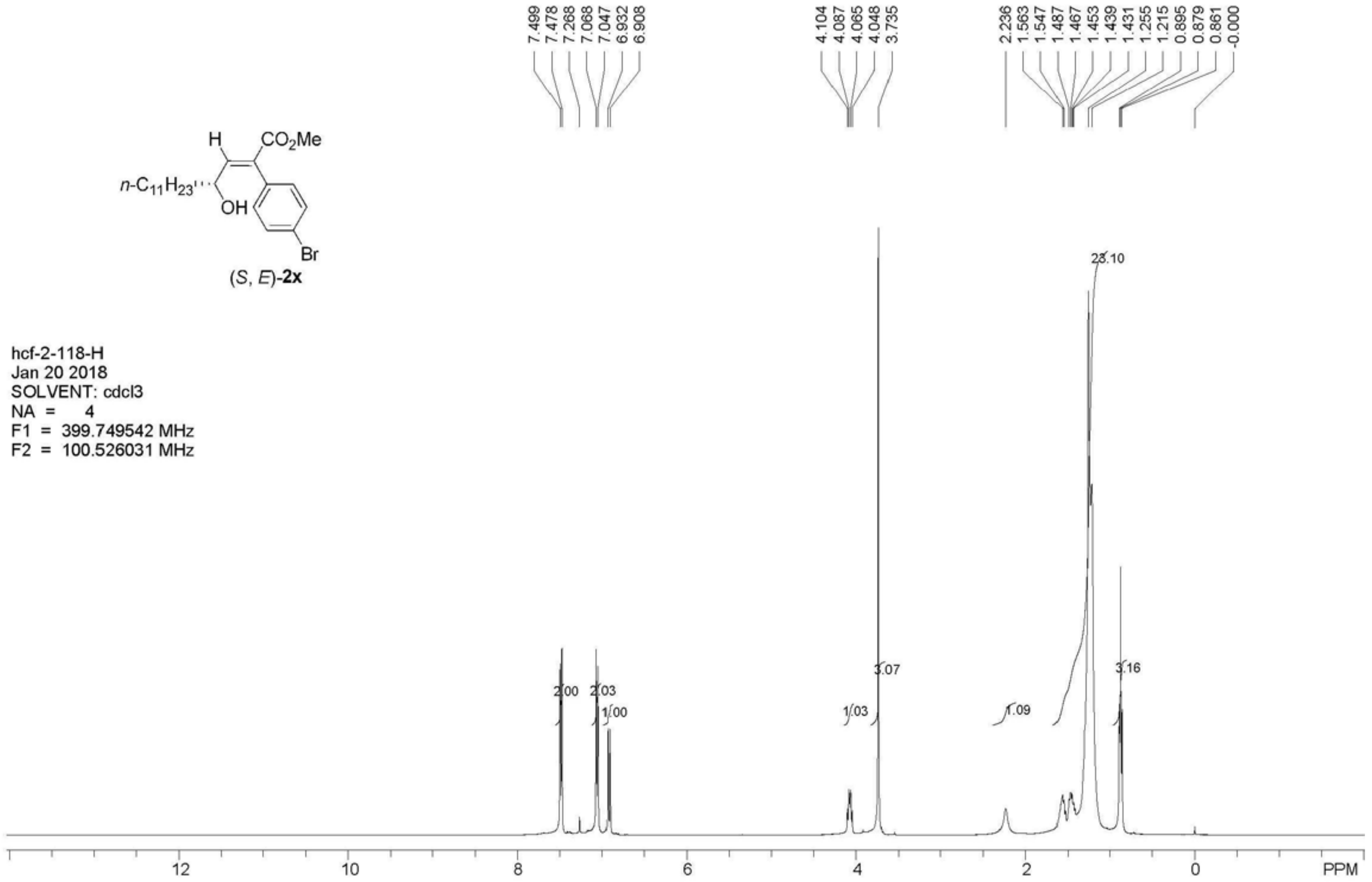

n-$C_{11}H_{23}$
OH
H
CO_2Me
Br
(S, E)-2x
hcf-2-118-H
Jan 20 2018
SOLVENT: cdcl3
NA = 4
F1 = 399.749542 MHz
F2 = 100.526031 MHz
7.499
7.478
7.268
7.068
7.047
6.932
6.908
4.104
4.087
4.065
4.048
3.735
2.236
1.563
1.547
1.487
1.467
1.453
1.439
1.431
1.255
1.215
0.895
0.879
0.861
-0.000
2.00
2.03
1.00
1.03
3.07
1.09
23.10
3.16
12
10
8
6
4
2
0
PPM

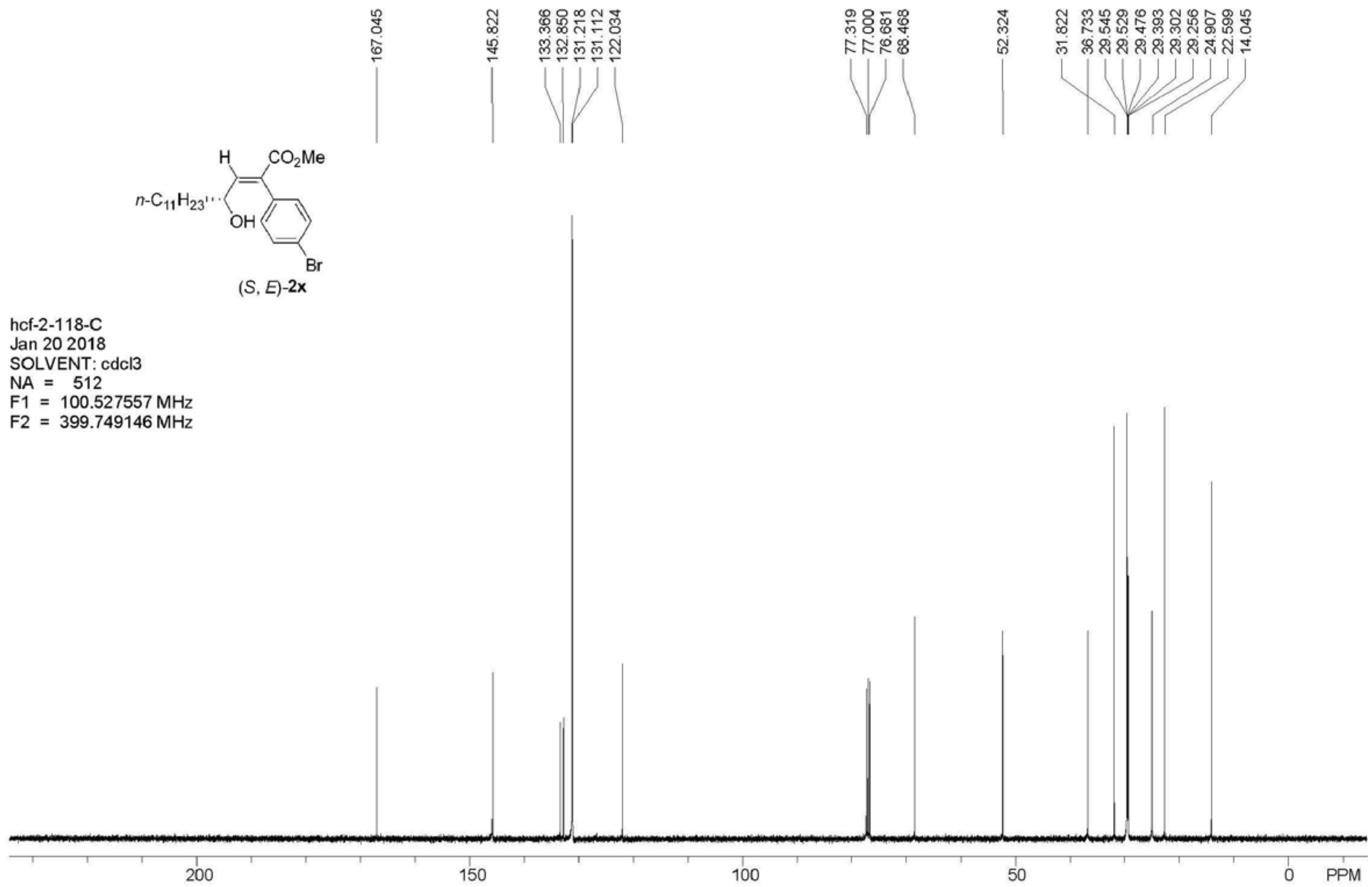
167.045
145.822
133.366
132.850
131.218
131.112
122.034
77.319
77.000
76.681
68.468
52.324
31.822
36.733
29.545
29.529
29.476
29.393
29.302
29.256
24.907
22.599
14.045
hcf-2-118-C
Jan 20 2018
SOLVENT: cdcl3
NA = 512
F1 = 100.527557 MHz
F2 = 399.749146 MHz
200
150
100
50
0
PPM

(*S*, *E*)-**2x**

中国科学院上海有机化学研究所

Project Name: defaults for copy

Reported by User: Breeze user (Breeze)

Breeze 2 HPLC System

SAMPLE INFORMATION

Sample Name:	hcy-2-114-as-h-97-3-1-214	Acquired By:	Breeze
Sample Type:	未知	Date Acquired:	2018/1/24 10:55:38 CST
Vial:	999	Acq. Method:	zgj97
Injection #:	285	Date Processed:	2018/1/24 15:23:10 CST
Injection Volume:	10.00 ul	Channel Name:	W2489 ChA
Run Time:	20.00 Minutes	Channel Desc.:	W2489 ChA 214nm
Column Type:		Sample Set Name:	

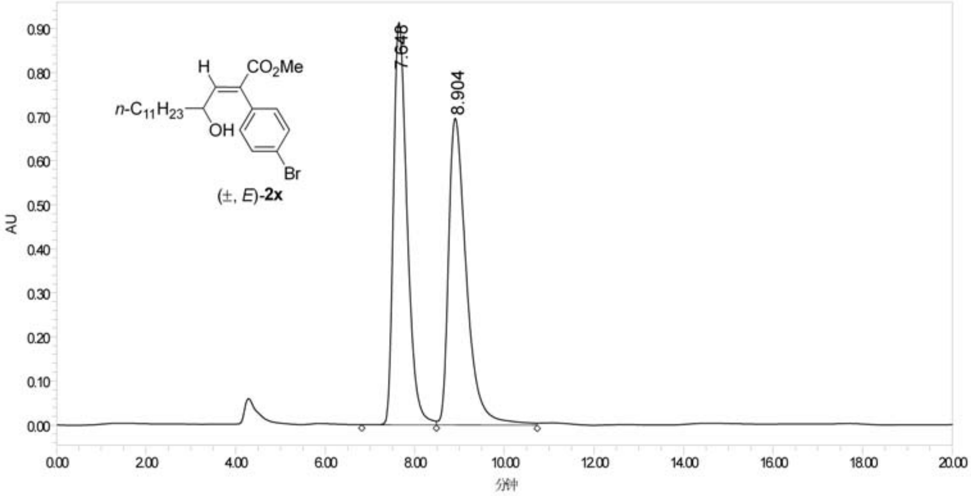

	RT (min)	Area (微伏*sec)	% Area	Height (微伏)	% Height
1	7.648	1932806C	50.0C	912718	56.75
2	8.904	1933113	50.0C	695578	43.25

Report Method: Individual Report ASC

Printed: 2018/1/24 15:26:10 PRC

Page: 1 (共计 1)

中国科学院上海有机化学研究所

Project Name: defaults for copy

Reported by User: Breeze user (Breeze)

Breeze 2 HPLC System

SAMPLE INFORMATION

Sample Name:	hcy-2-118-as-h-97-3-1-214	Acquired By:	Breeze
Sample Type:	未知	Date Acquired:	2018/1/24 12:30:05 CST
Vial:	999	Acq. Method:	zgj97
Injection #:	288	Date Processed:	2018/1/24 15:25:06 CST
Injection Volume:	10.00 ul	Channel Name:	W2489 ChA
Run Time:	20.00 Minutes	Channel Desc.:	W2489 ChA 214nm
Column Type:		Sample Set Name:	

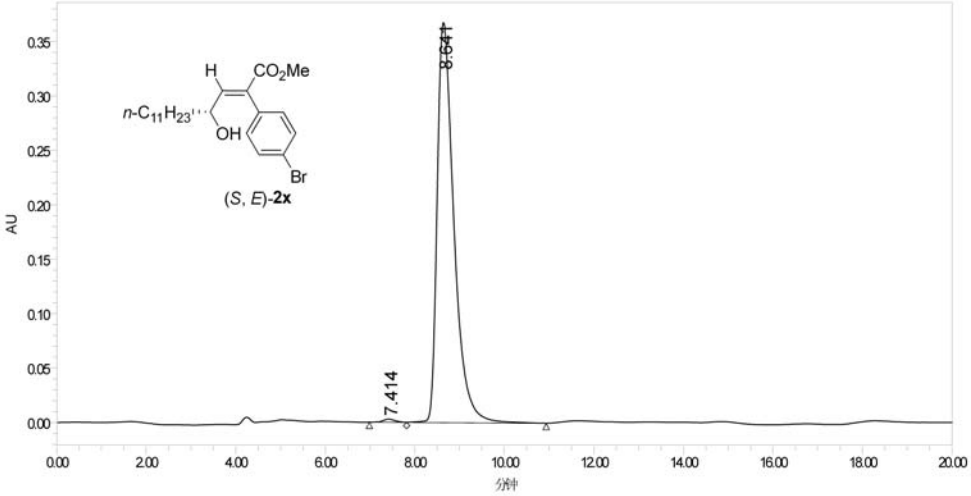

	RT (min)	Area ([illegible]*sec)	% Area	Height ([illegible])	% Height
1	7.414	55389	0.58	2874	0.78
2	8.641	9537059	99.42	367908	99.22

Report Method: Individual Report ASC

Page: 1 (共计 1)

Printed: 2018/1/24 15:26:56 PRC

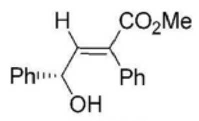

(*R*, *E*)-**2v**

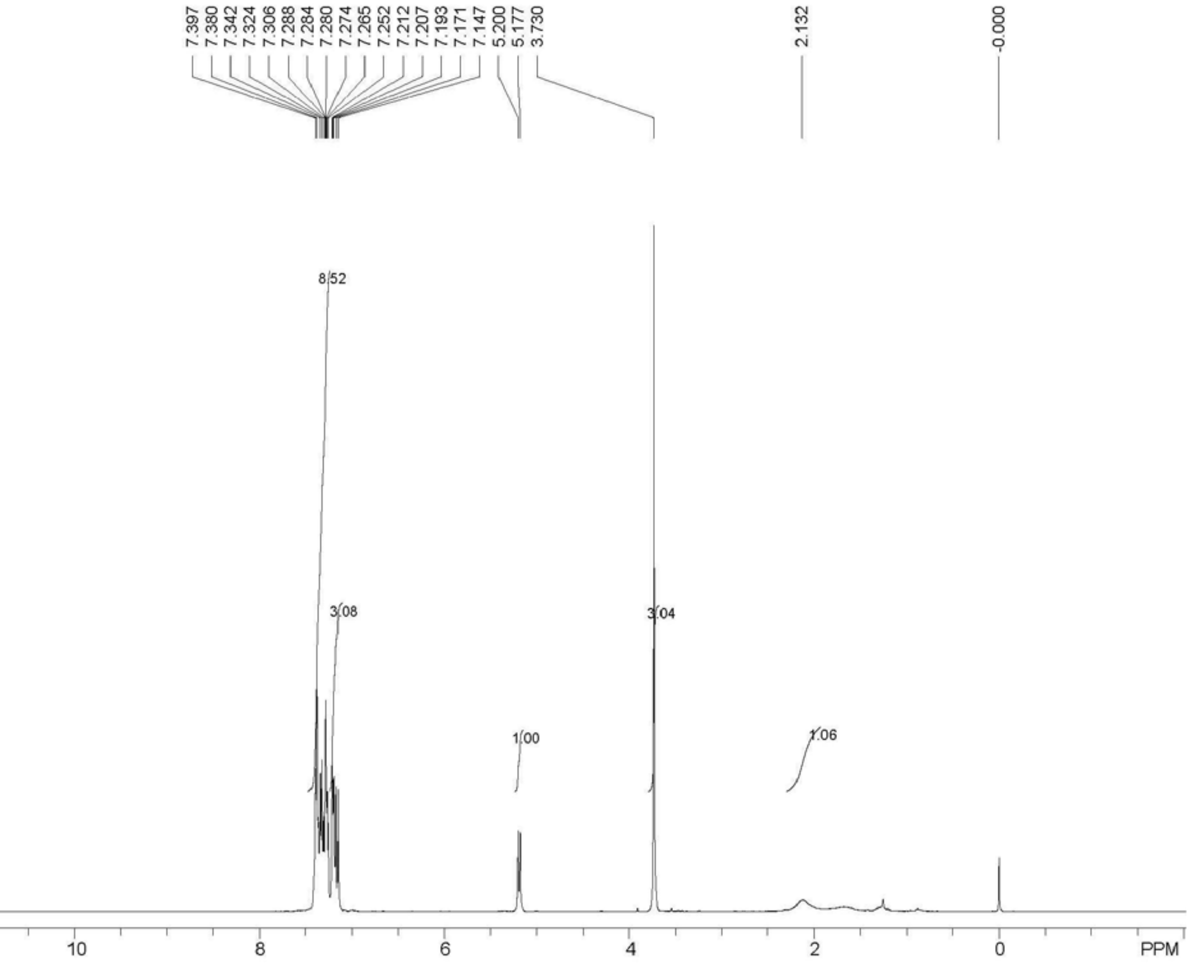

7.397
7.380
7.342
7.324
7.306
7.288
7.284
7.280
7.274
7.265
7.252
7.212
7.207
7.193
7.171
7.147
5.200
5.177
3.730
2.132
-0.000
hcf-2-132-H
Jan 29 2018
SOLVENT: cdcl3
NA = 8
F1 = 399.749542 MHz
F2 = 100.526031 MHz
8.52
3.08
3.04
1.00
1.06
12
10
8
6
4
2
0
PPM

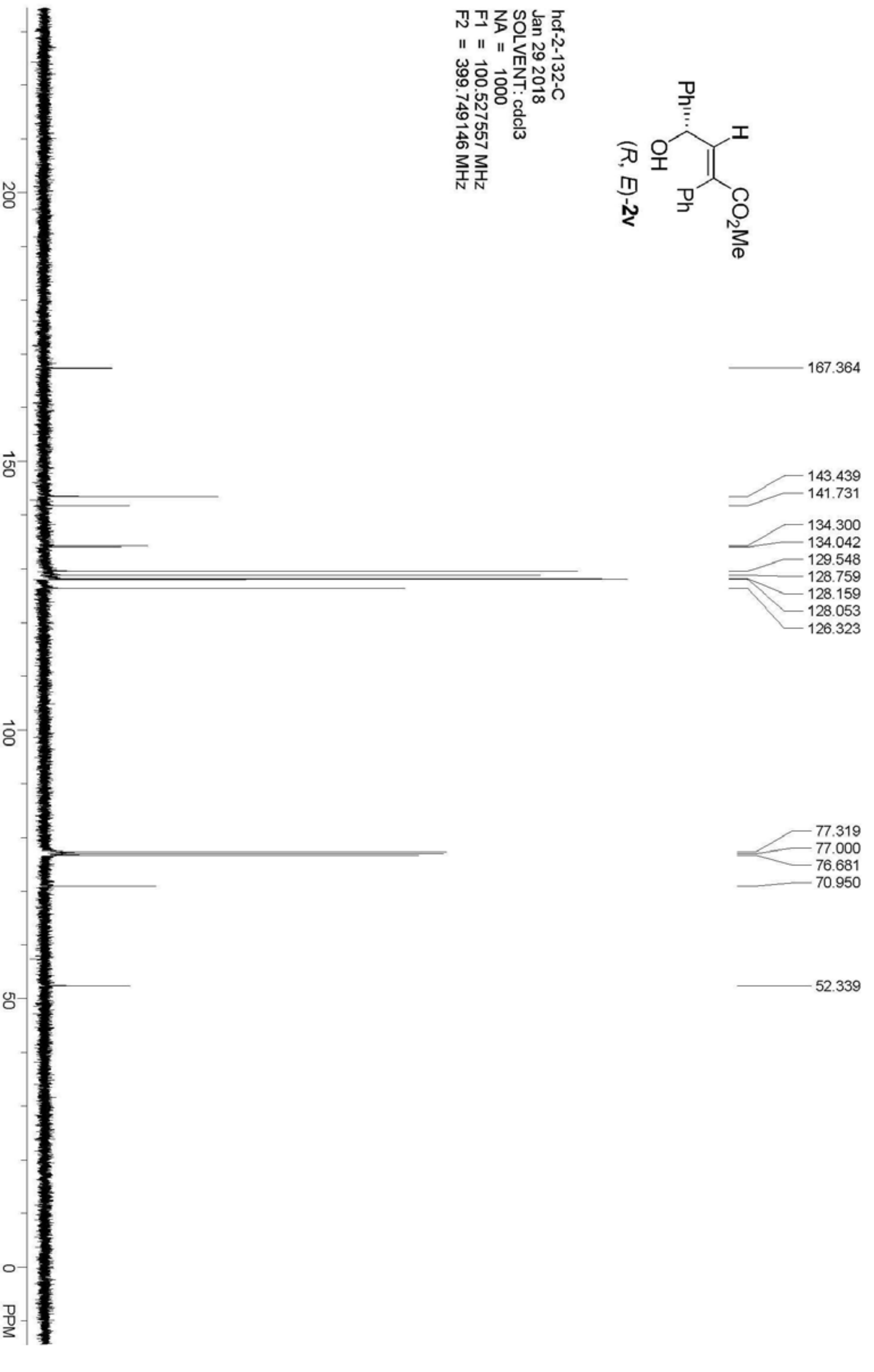
hcf-2-132-C
Jan 29 2018
SOLVENT: cdcl3
NA = 1000
F1 = 100.527557 MHz
F2 = 399.749146 MHz
(R, E)-2v
167.364
143.439
141.731
134.300
134.042
129.548
128.759
128.159
128.053
126.323
77.319
77.000
76.681
70.950
52.339
200
150
100
50
0
PPM

hcf-2-125-pa-2-80-20-0.7-214

实验时间：2018-02-02, 15:21:17　　　　报告时间：2018-02-02, 17:16:54

谱图文件:D:\zhuguangjiong\hcf\20180131\hcf-2-125-pa-2-80-20-0.7-214.org

实验内容简介：

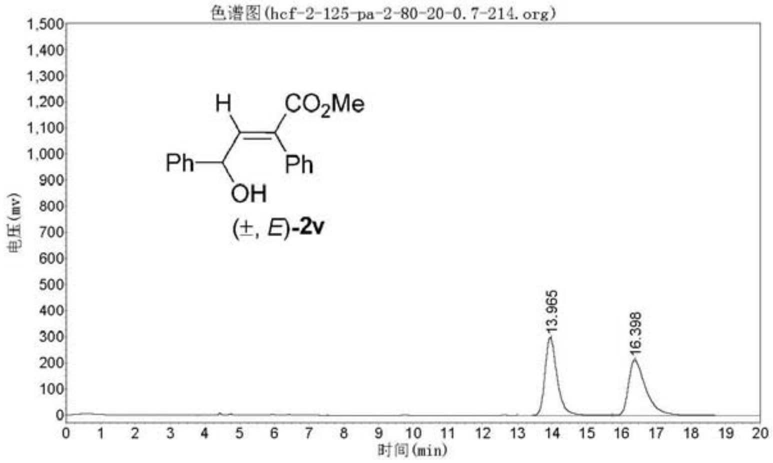

分析结果表

峰号	峰名	保留时间	峰高	峰面积	含量
1		13.965	298364.969	7329453.500	49.5159
2		16.398	212095.734	7472764.000	50.4841
**总计**			510460.703	14802217.500	100.0000

hcf-2-132-pa-2-80-20-0.7-214

实验时间：2018-02-02,16:06:47　　　　报告时间：2018-02-02,17:18:24
谱图文件:D:\zhuguangjiong\hcf\20180131\hcf-2-132-pa-2-80-20-0.7-214..org

实验内容简介：

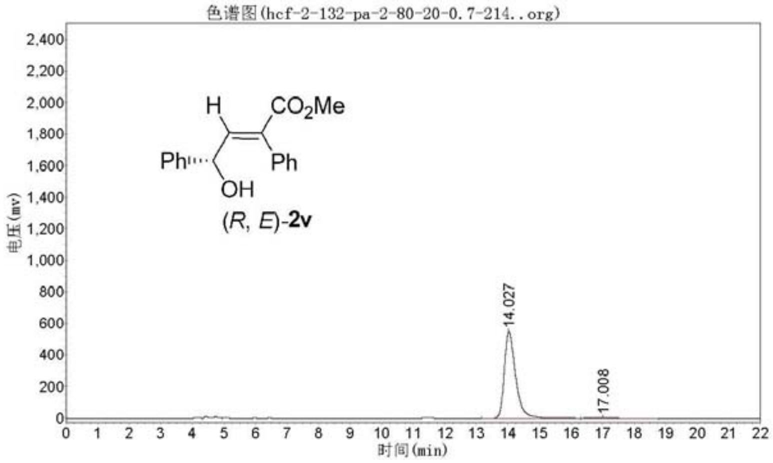

分析结果表

峰号	峰名	保留时间	峰高	峰面积	含量
1		14.027	546686.625	13619557.000	99.4113
2		17.008	1243.959	80652.625	0.5887
总计			547930.584	13700209.625	100.0000

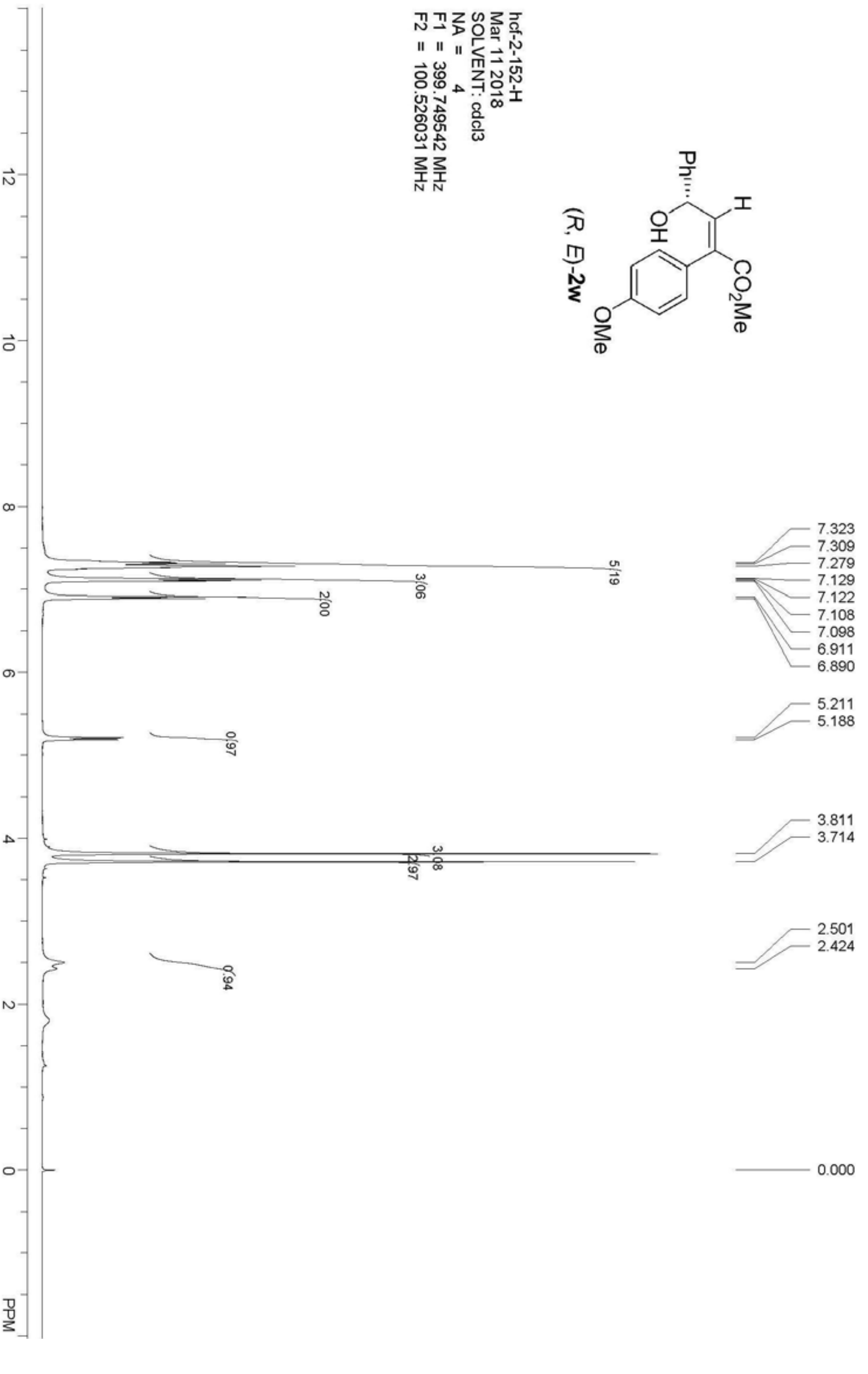
hcf-2-152-H
Mar 11 2018
SOLVENT: cdcl3
NA = 4
F1 = 399.749542 MHz
F2 = 100.526031 MHz
Ph
OH
H
CO2Me
OMe
(R, E)-2w
7.323
7.309
7.279
7.129
7.122
7.108
7.098
6.911
6.890
5.211
5.188
3.811
3.714
2.501
2.424
0.000
5.19
3.06
2.00
0.97
3.08
2.97
0.94
12
10
8
6
4
2
0
PPM

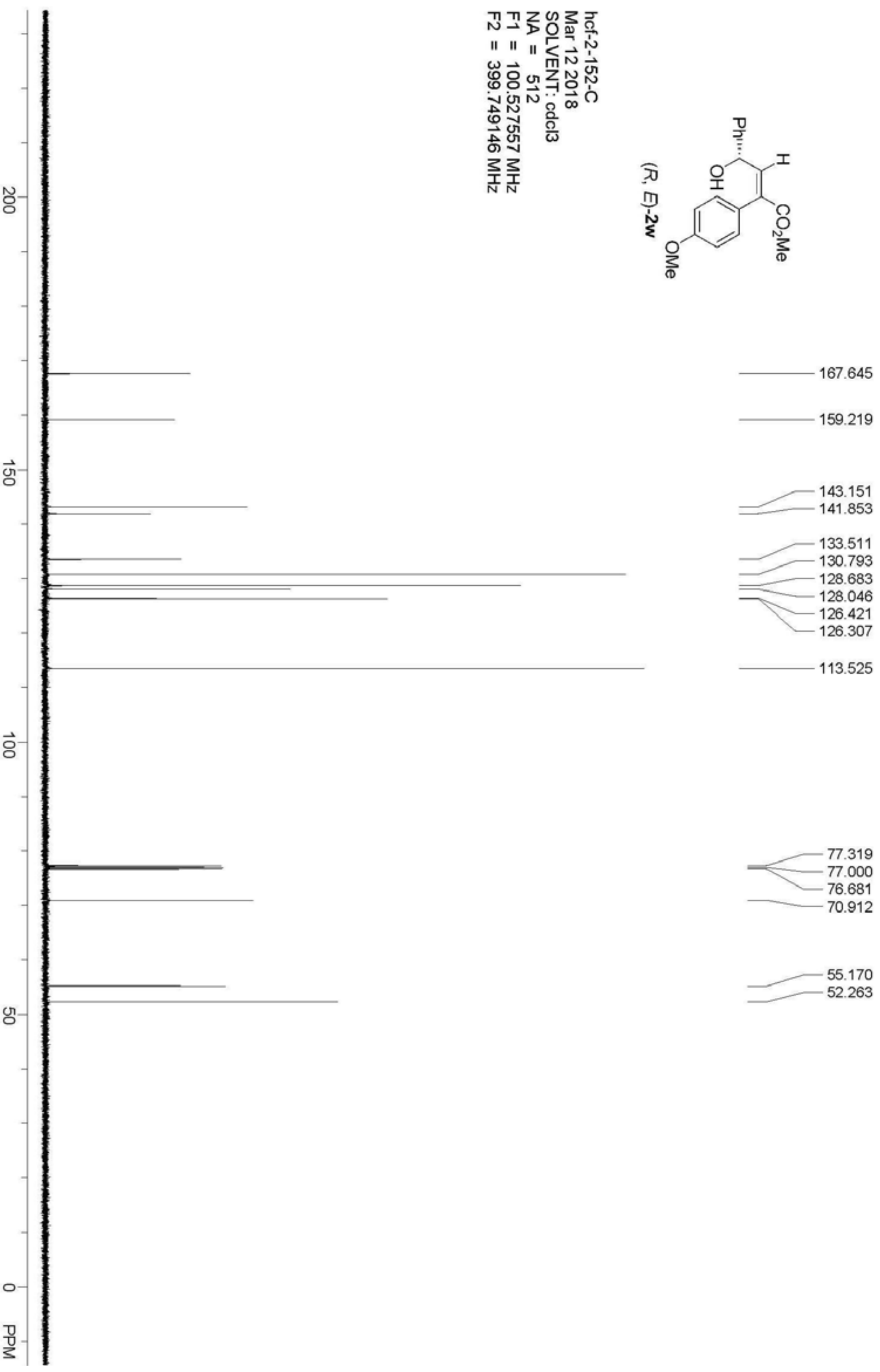
hcf-2-152-C
Mar 12 2018
SOLVENT: cdcl3
NA = 512
F1 = 100.527557 MHz
F2 = 399.749146 MHz
(R, E)-2w
Ph
OH
H
CO2Me
OMe
167.645
159.219
143.151
141.853
133.511
130.793
128.683
128.046
126.421
126.307
113.525
77.319
77.000
76.681
70.912
55.170
52.263
200
150
100
50
0
PPM

hcf-2-126-pc-2-50-50-0.8-214

实验时间：2018-02-06, 10:26:57　　　　　　报告时间：2018-02-06, 12:29:43
谱图文件:D:\zhuguangjiong\hcf\20180205\hcf-2-126-pc-2-50-50-0.8-214...org

实验内容简介：

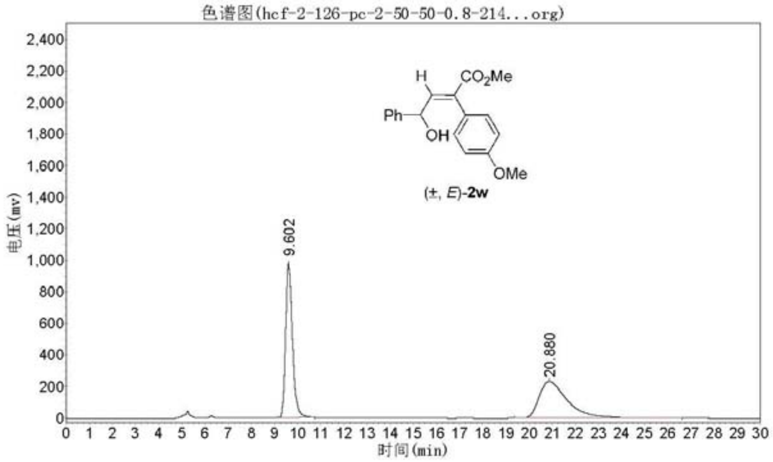

分析结果表

峰号	峰名	保留时间	峰高	峰面积	含量
1		9.602	973350.625	19930722.000	50.1094
2		20.880	232116.125	19843714.000	49.8906
总计			1205466.750	39774436.000	100.0000

中国科学院上海有机化学研究所

Project Name: defaults for copy

Reported by User: Breeze user (Breeze)

Breeze 2 HPLC System

SAMPLE INFORMATION

Sample Name:	hcf-2-152-pc-2-50-50-0.8-214	Acquired By:	Breeze
Sample Type:	未知	Date Acquired:	2018/3/13 18:23:01 CST
Vial:	999	Acq. Method:	zgj50
Injection #:	17	Date Processed:	2018/3/13 18:47:07 CST
Injection Volume:	10.00 ul	Channel Name:	W2489 ChA
Run Time:	20.00 Minutes	Channel Desc.:	W2489 ChA 214nm
Column Type:		Sample Set Name:	

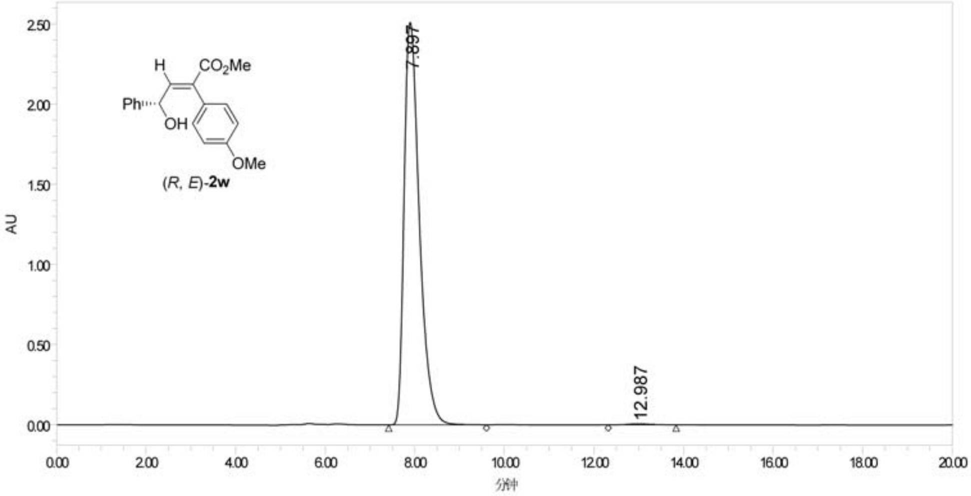

	RT (min)	Area ([illegible]*sec)	% Area	Height ([illegible])	% Height
1	7.897	59259699	99.65	2510851	99.78
2	12.987	210680	0.35	5624	0.22

Report Method: Individual Report ASC

Printed: 2018/3/13 18:47:21 PRC

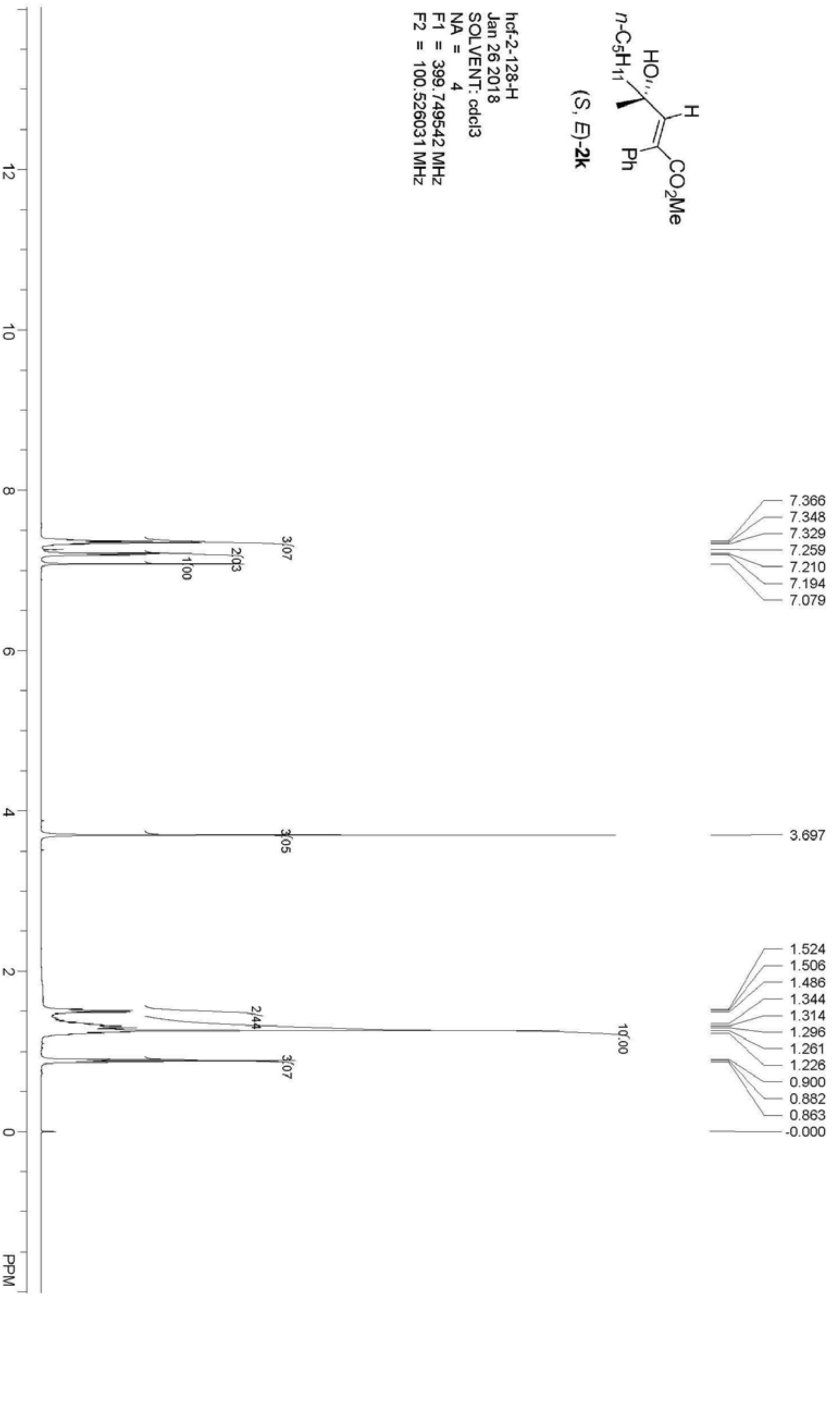

H
HO,,
CO_2Me
n-C_5H_{11}
Ph
(S, E)-**2k**
hcf-2-128-H
Jan 26 2018
SOLVENT: cdcl3
NA = 4
F1 = 399.749542 MHz
F2 = 100.526031 MHz
7.366
7.348
7.329
7.259
7.210
7.194
7.079
3.697
1.524
1.506
1.486
1.344
1.314
1.296
1.261
1.226
0.900
0.882
0.863
-0.000
3.07
2.03
1.00
3.05
2.44
10.00
3.07
12
10
8
6
4
2
0
PPM

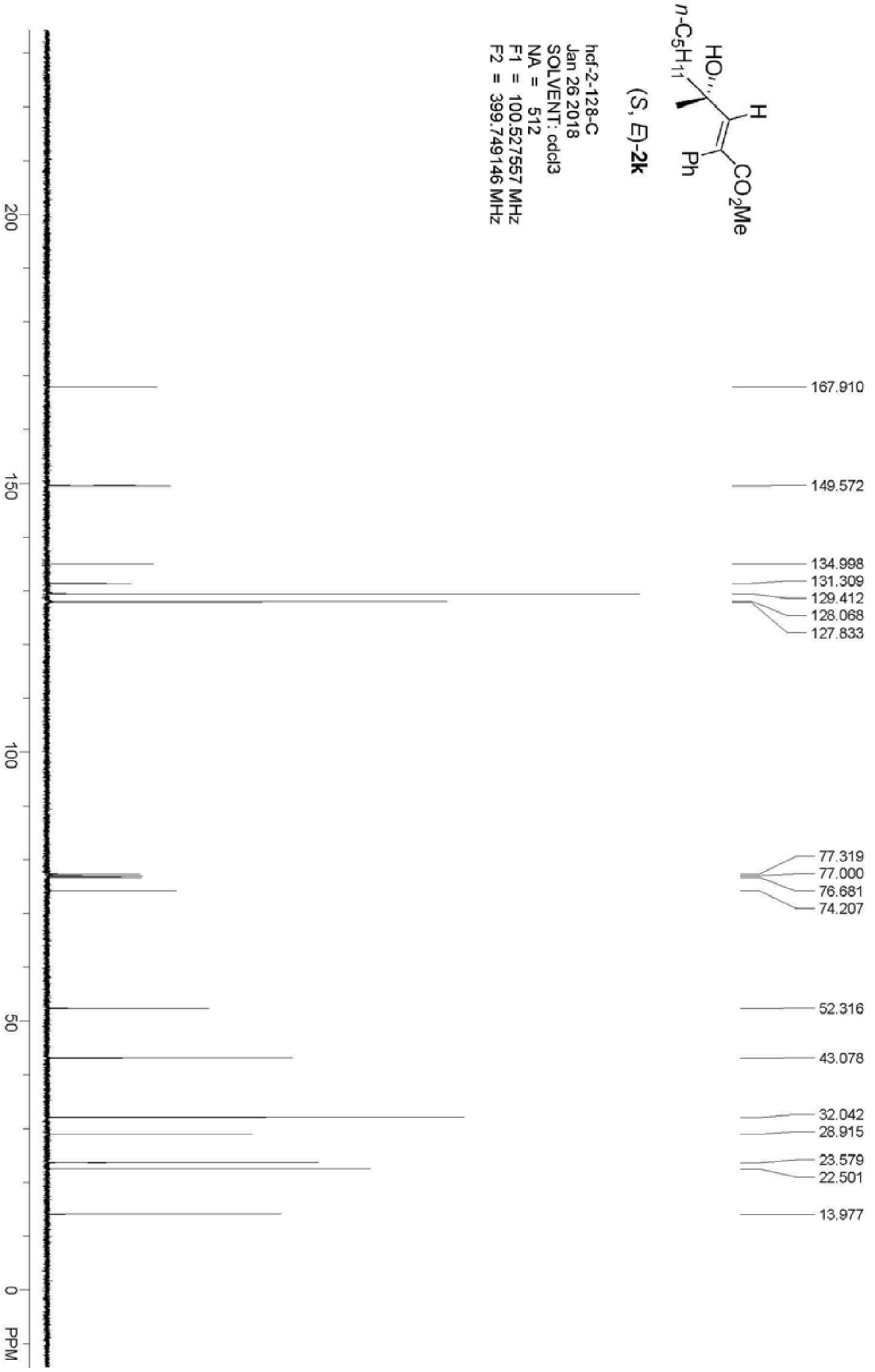
(S, E)-2k
hcf-2-128-C
Jan 26 2018
SOLVENT: cdcl3
NA = 512
F1 = 100.527557 MHz
F2 = 399.749146 MHz
167.910
149.572
134.998
131.309
129.412
128.068
127.833
77.319
77.000
76.681
74.207
52.316
43.078
32.042
28.915
23.579
22.501
13.977
PPM

55

Project Name: defaults for copy
Reported by User: Breeze user (Breeze)

Breeze 2 HPLC System

SAMPLE INFORMATION

Sample Name:	hcf-2-46-pa-2-95-5-1-214	Acquired By:	Breeze
Sample Type:	未知	Date Acquired:	2018/1/26 14:14:49 CST
Vial:	999	Acq. Method:	zgj95
Injection #:	291	Date Processed:	2018/1/26 18:37:35 CST
Injection Volume:	10.00 ul	Channel Name:	W2489 ChA
Run Time:	60.00 Minutes	Channel Desc.:	W2489 ChA 214nm
Column Type:		Sample Set Name:	

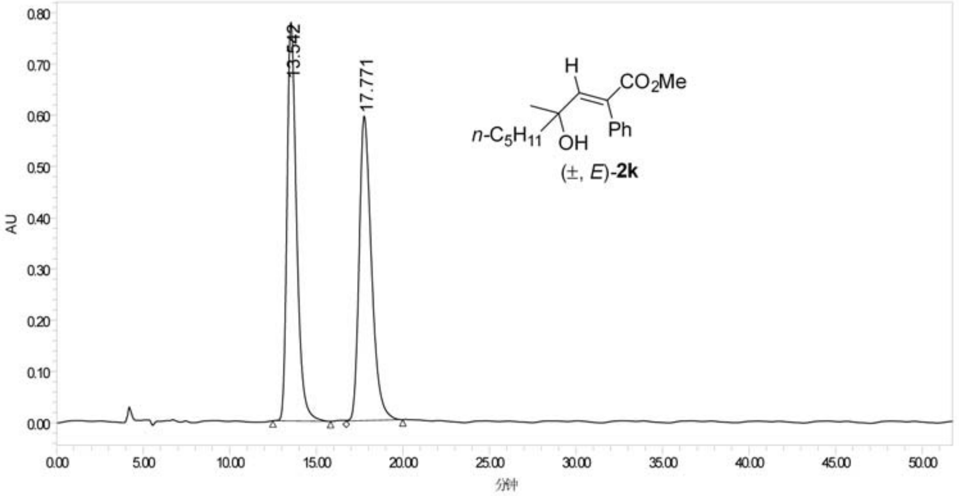

	RT (min)	Area ([illegible]*sec)	% Area	Height ([illegible])	% Height
1	13.542	29738986	50.36	777925	56.71
2	17.771	29318203	49.64	593792	43.29

Report Method: Individual Report ASC
Page: 1 (共计 1)

Printed: 2018/1/26
18:40:30 PRC

SAMPLE INFORMATION

Sample Name:	hcf-2-128-pa-2-95-5-1-214	Acquired By:	Breeze
Sample Type:	未知	Date Acquired:	2018/1/26 15:33:53 CST
Vial:	999	Acq. Method:	zgj95
Injection #:	293	Date Processed:	2018/1/26 18:40:14 CST
Injection Volume:	10.00 ul	Channel Name:	W2489 ChA
Run Time:	30.00 Minutes	Channel Desc.:	W2489 ChA 214nm
Column Type:		Sample Set Name:	

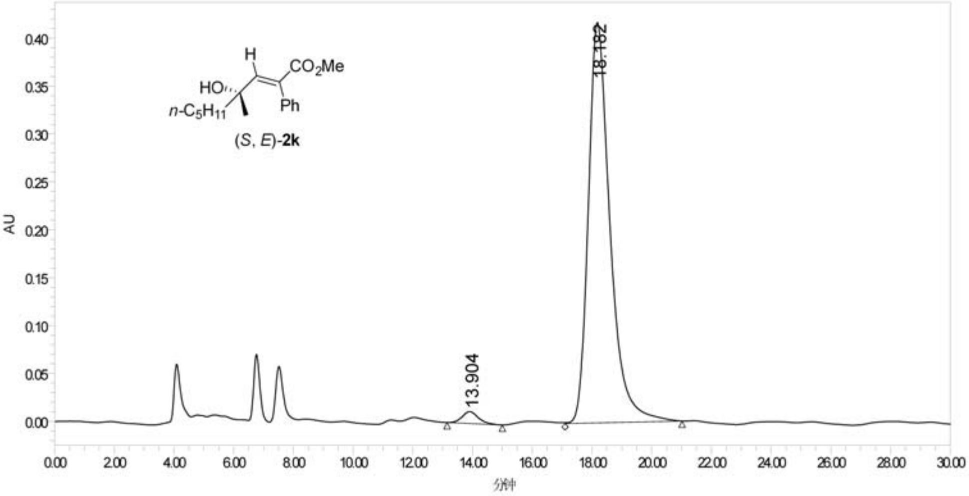

	RT (min)	Area ([illegible]*sec)	% Area	Height ([illegible])	% Height
1	13.904	470903	2.19	12428	2.89
2	18.182	21049959	97.81	417943	97.11

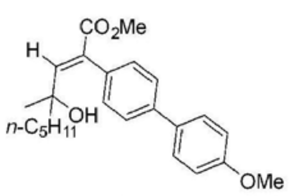

(*E*)-**7**

hcf-2-98-H
Jan 4 2018
SOLVENT: cdcl3
NA = 4
F1 = 399.749542 MHz
F2 = 100.526031 MHz

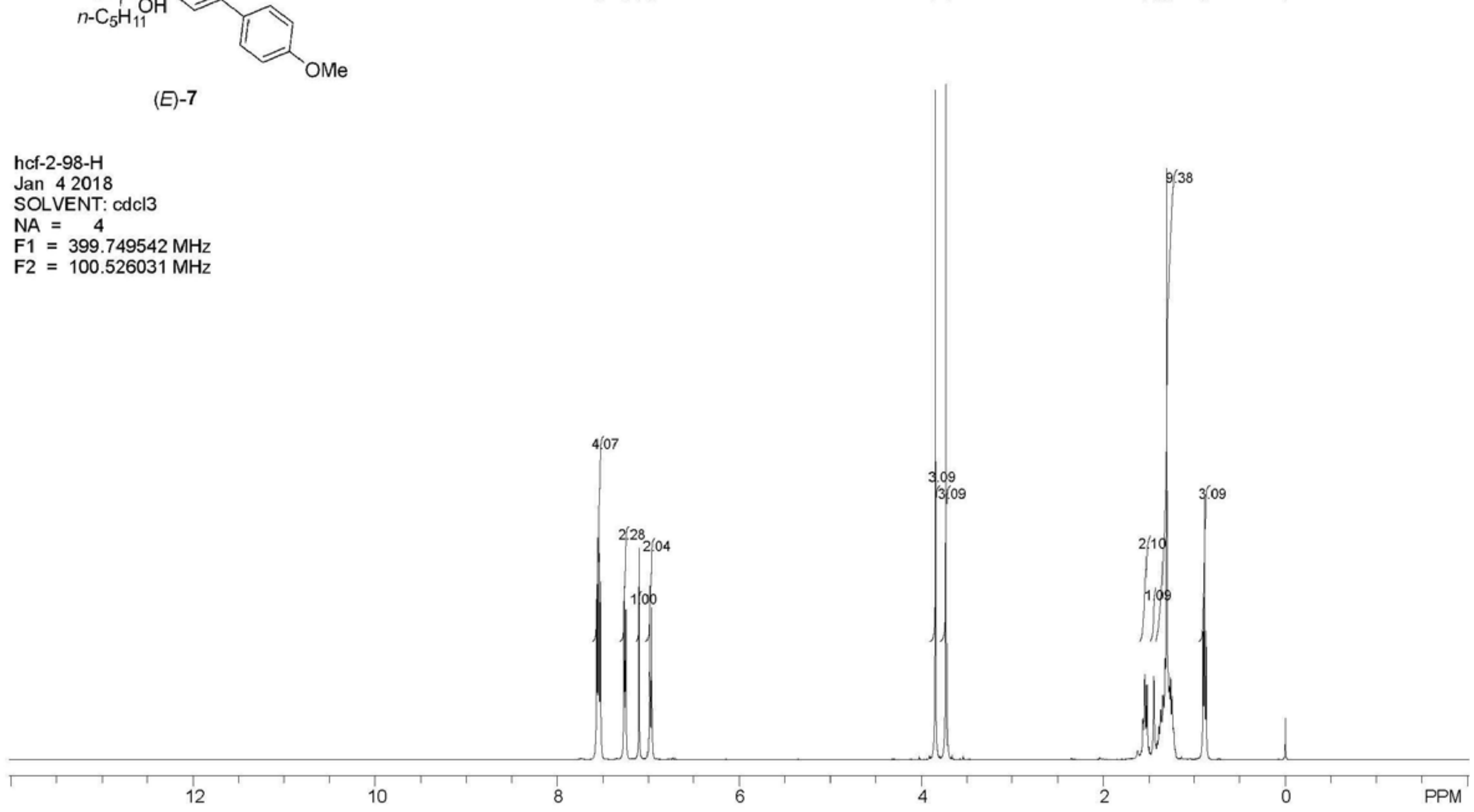

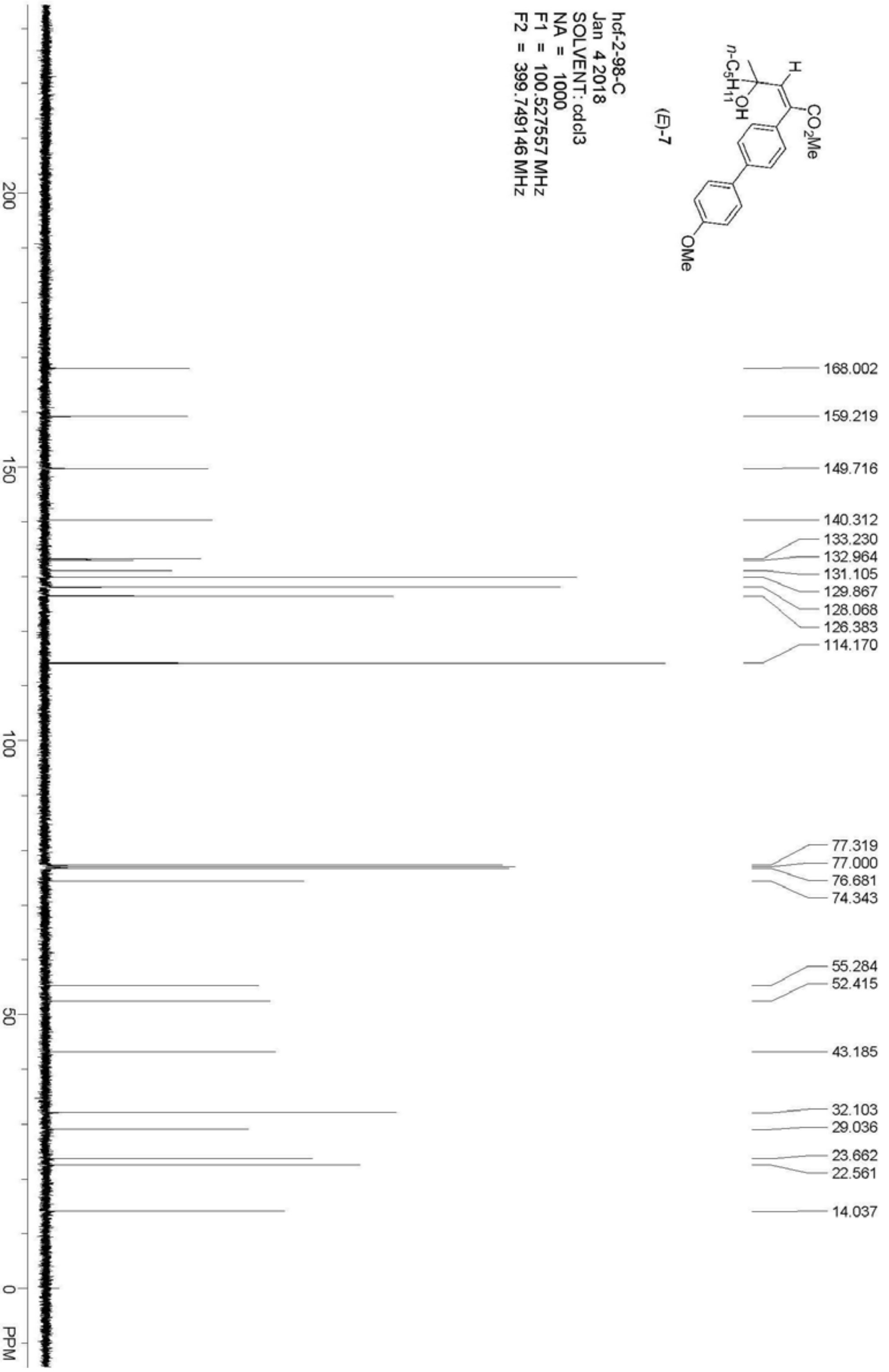

hcf-2-98-C
Jan 4 2018
SOLVENT: cdcl3
NA = 1000
F1 = 100.527557 MHz
F2 = 399.749146 MHz
CO2Me
H
OH
n-C5H11
OMe
(E)-7
168.002
159.219
149.716
140.312
133.230
132.964
131.105
129.867
128.068
126.383
114.170
77.319
77.000
76.681
74.343
55.284
52.415
43.185
32.103
29.036
23.662
22.561
14.037
200
150
100
50
0
PPM

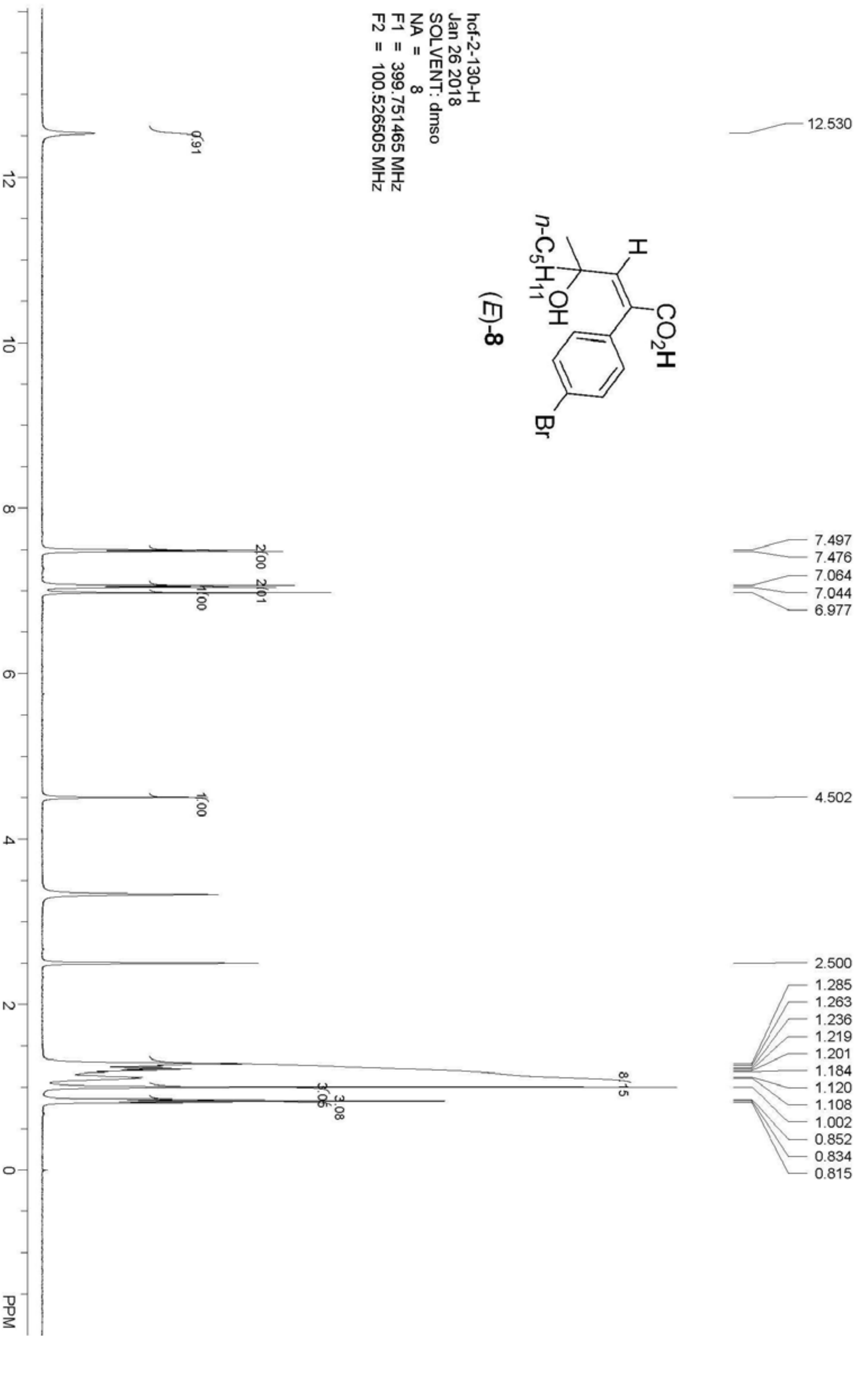
hcf-2-130-H
Jan 26 2018
SOLVENT: dmso
NA = 8
F1 = 399.751465 MHz
F2 = 100.526505 MHz
CO2H
H
n-C5H11
OH
Br
(E)-8
12.530
7.497
7.476
7.064
7.044
6.977
4.502
2.500
1.285
1.263
1.236
1.219
1.201
1.184
1.120
1.108
1.002
0.852
0.834
0.815
0.91
2.00
2.01
1.00
1.00
8.15
3.06
3.08
PPM

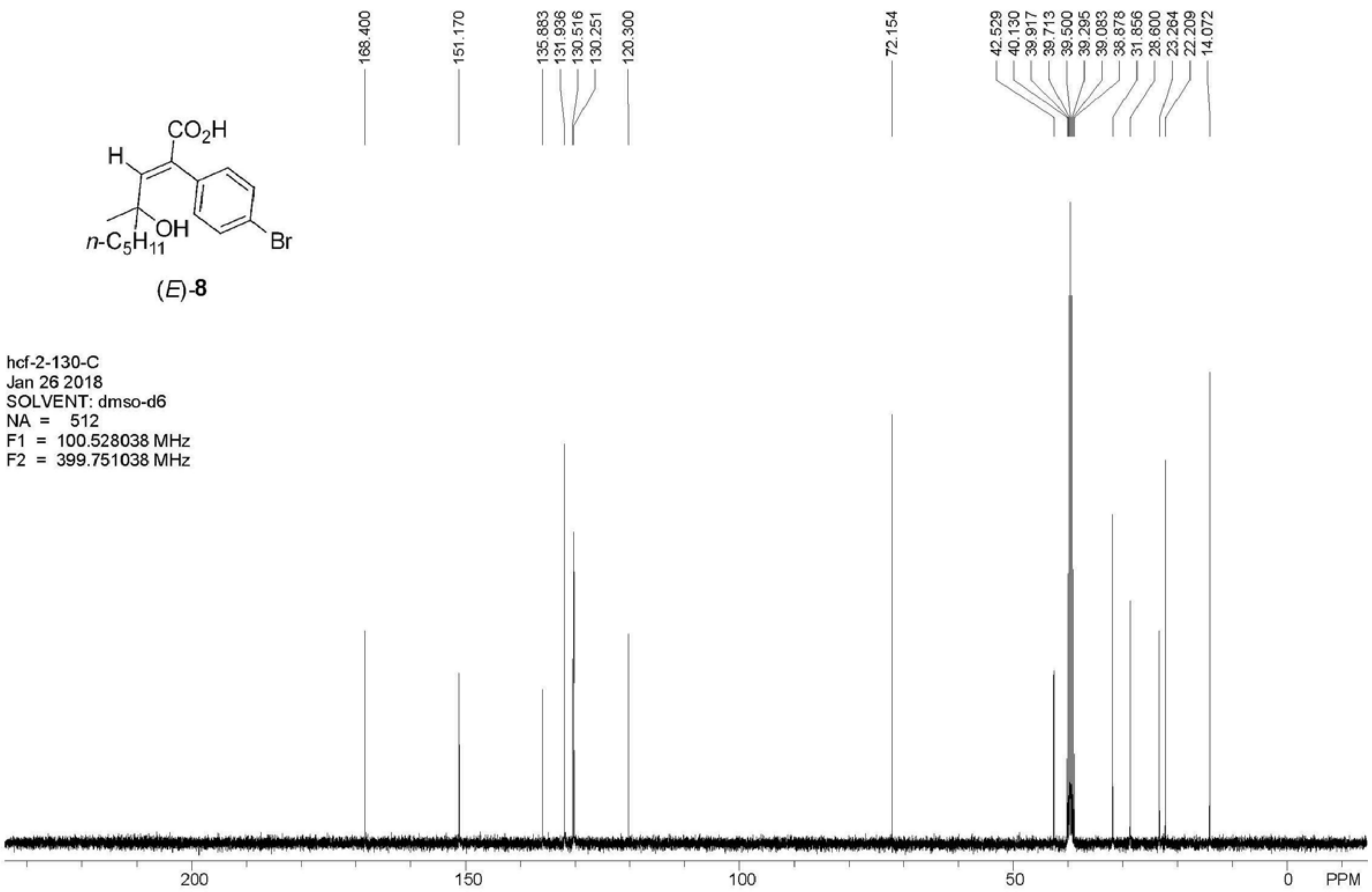
CO2H
H
n-C5H11 OH
Br
(E)-8
hcf-2-130-C
Jan 26 2018
SOLVENT: dmso-d6
NA = 512
F1 = 100.528038 MHz
F2 = 399.751038 MHz
168.400
151.170
135.883
131.936
130.516
130.251
120.300
72.154
42.529
40.130
39.917
39.713
39.500
39.295
39.083
38.878
31.856
28.600
23.264
22.209
14.072
200
150
100
50
0
PPM

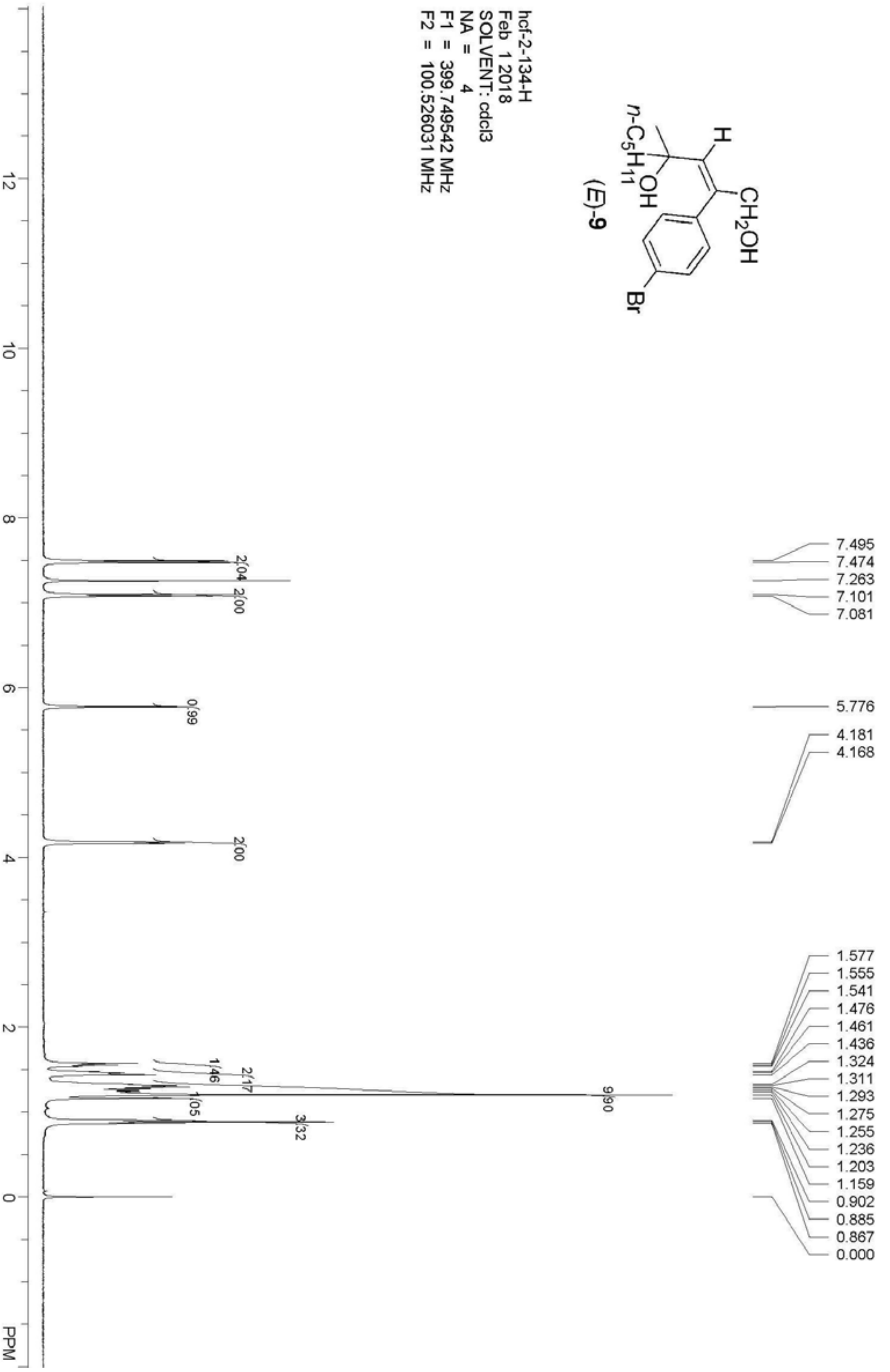
hcf-2-134-H
Feb 1 2018
SOLVENT: cdcl3
NA = 4
F1 = 399.749542 MHz
F2 = 100.526031 MHz
CH2OH
H
Br
n-C5H11
OH
(E)-9
7.495
7.474
7.263
7.101
7.081
5.776
4.181
4.168
1.577
1.555
1.541
1.476
1.461
1.436
1.324
1.311
1.293
1.275
1.255
1.236
1.203
1.159
0.902
0.885
0.867
0.000
2.04
2.00
0.99
2.00
1.46
2.17
9.90
1.05
3.32
12
10
8
6
4
2
0
PPM

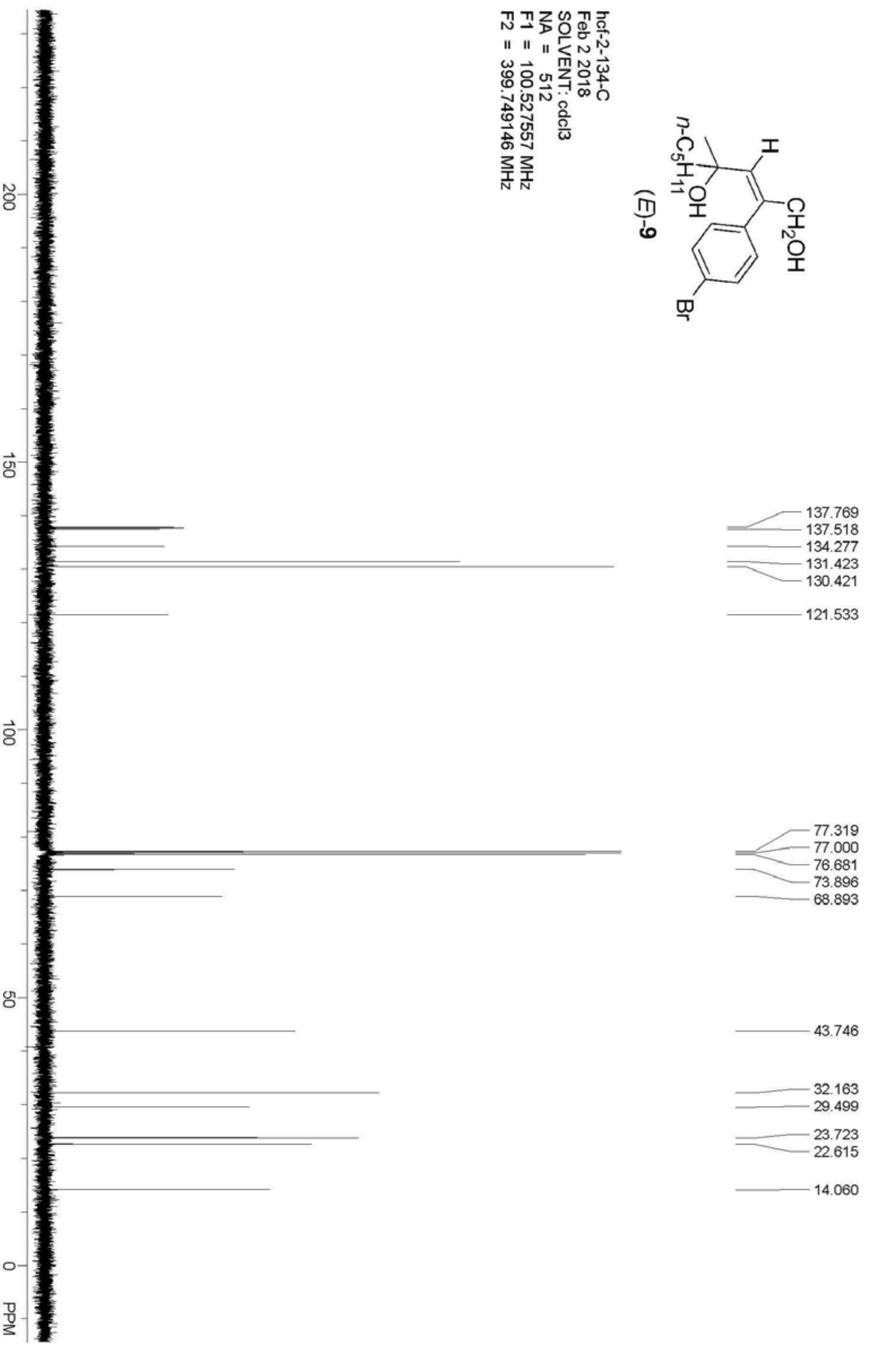

hcf-2-134-C
Feb 2 2018
SOLVENT: cdcl3
NA = 512
F1 = 100.527557 MHz
F2 = 399.749146 MHz
H
CH2OH
n-C5H11
OH
Br
(E)-9
137.769
137.518
134.277
131.423
130.421
121.533
77.319
77.000
76.681
73.896
68.893
43.746
32.163
29.499
23.723
22.615
14.060
200
150
100
50
0
PPM

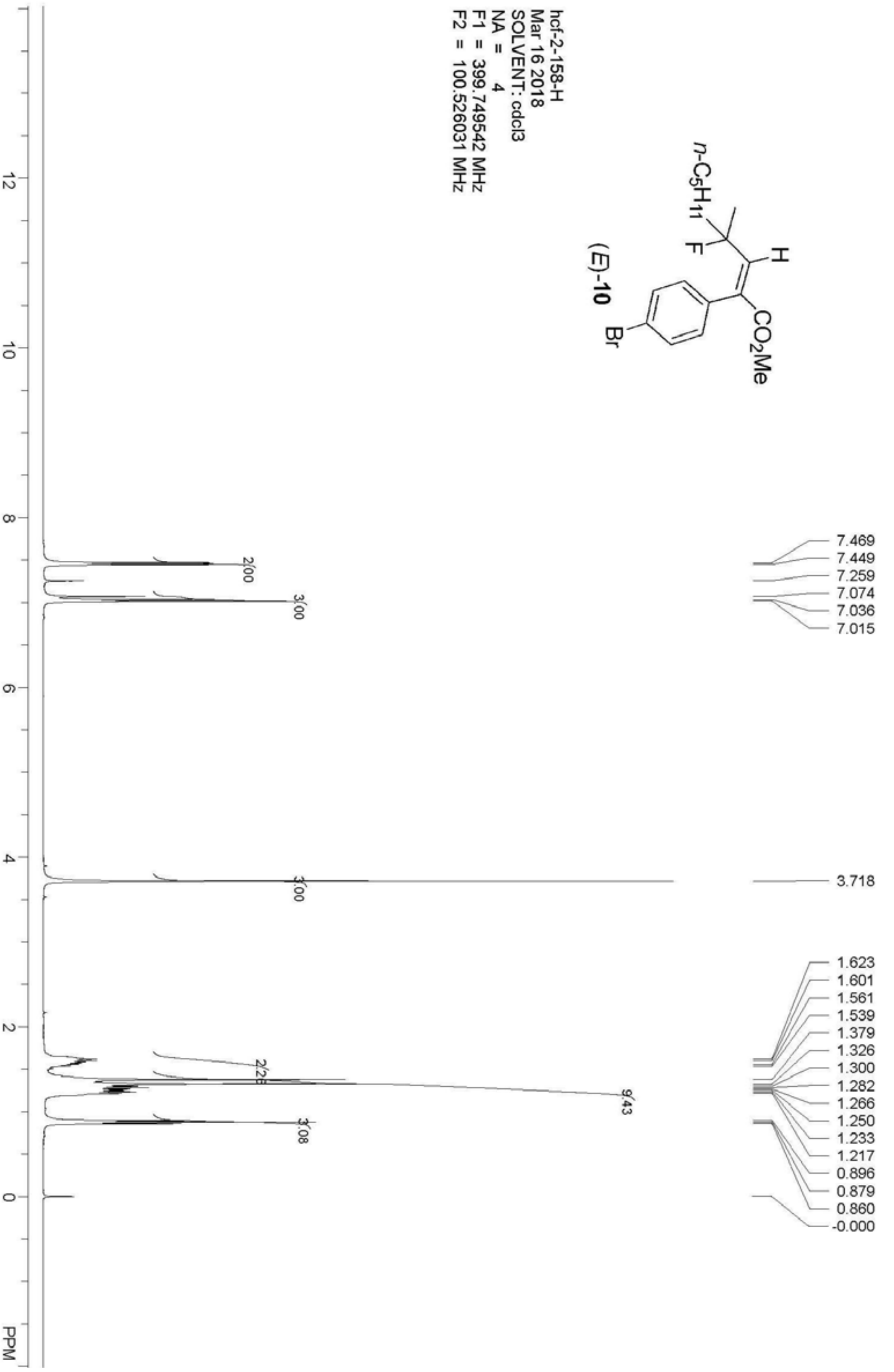
hcf-2-158-H
Mar 16 2018
SOLVENT: cdcl3
NA = 4
F1 = 399.749542 MHz
F2 = 100.526031 MHz
n-C5H11
F
H
CO2Me
Br
(E)-10
7.469
7.449
7.259
7.074
7.036
7.015
3.718
1.623
1.601
1.561
1.539
1.379
1.326
1.300
1.282
1.266
1.250
1.233
1.217
0.896
0.879
0.860
-0.000
2.00
3.00
3.00
2.26
9.43
3.08
12
10
8
6
4
2
0
PPM

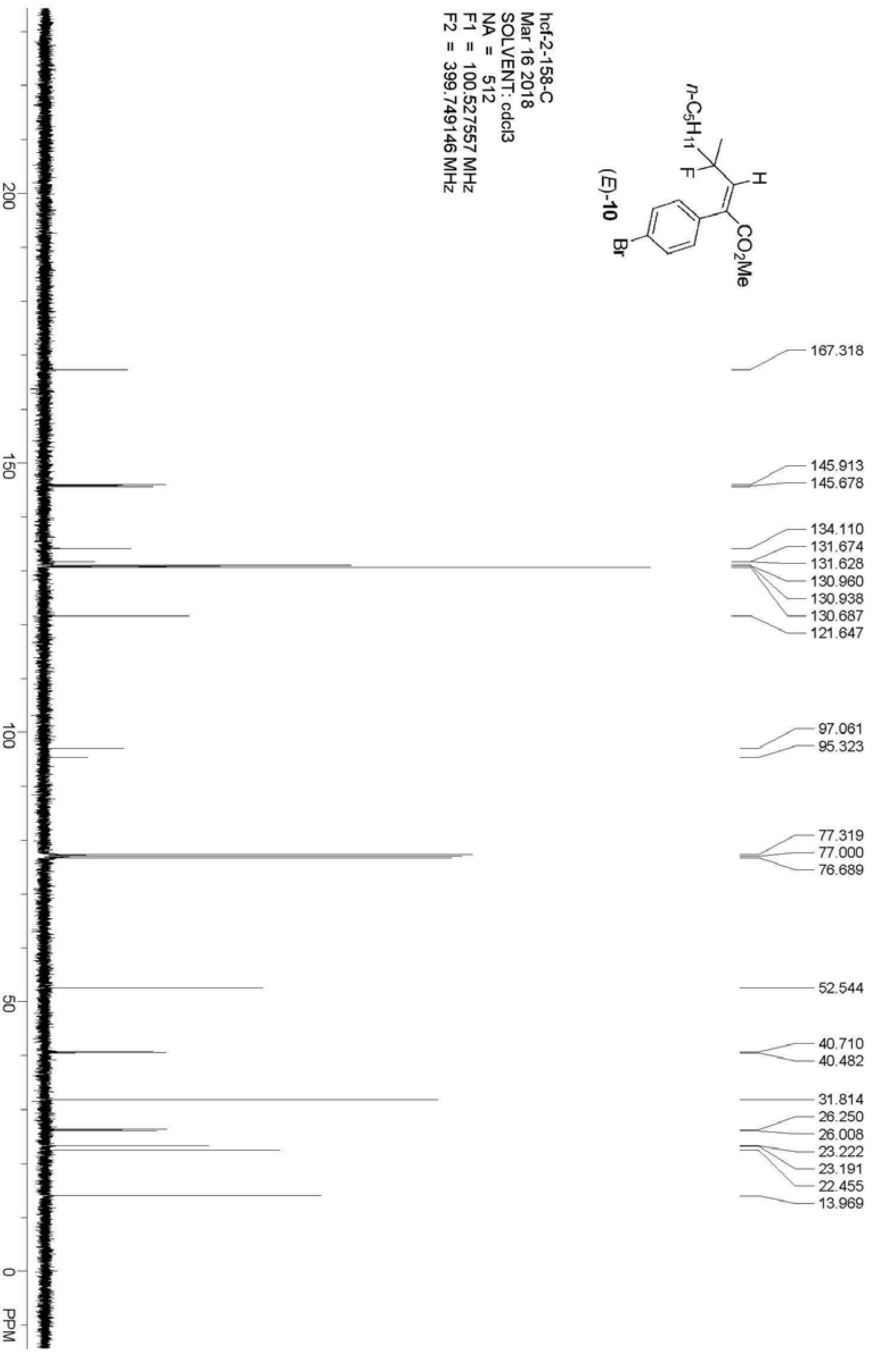

hcf-2-158-C
Mar 16 2018
SOLVENT: cdcl3
NA = 512
F1 = 100.527557 MHz
F2 = 399.749146 MHz
n-C5H11
F
H
CO2Me
Br
(E)-10
167.318
145.913
145.678
134.110
131.674
131.628
130.960
130.938
130.687
121.647
97.061
95.323
77.319
77.000
76.689
52.544
40.710
40.482
31.814
26.250
26.008
23.222
23.191
22.455
13.969
200
150
100
50
0
PPM

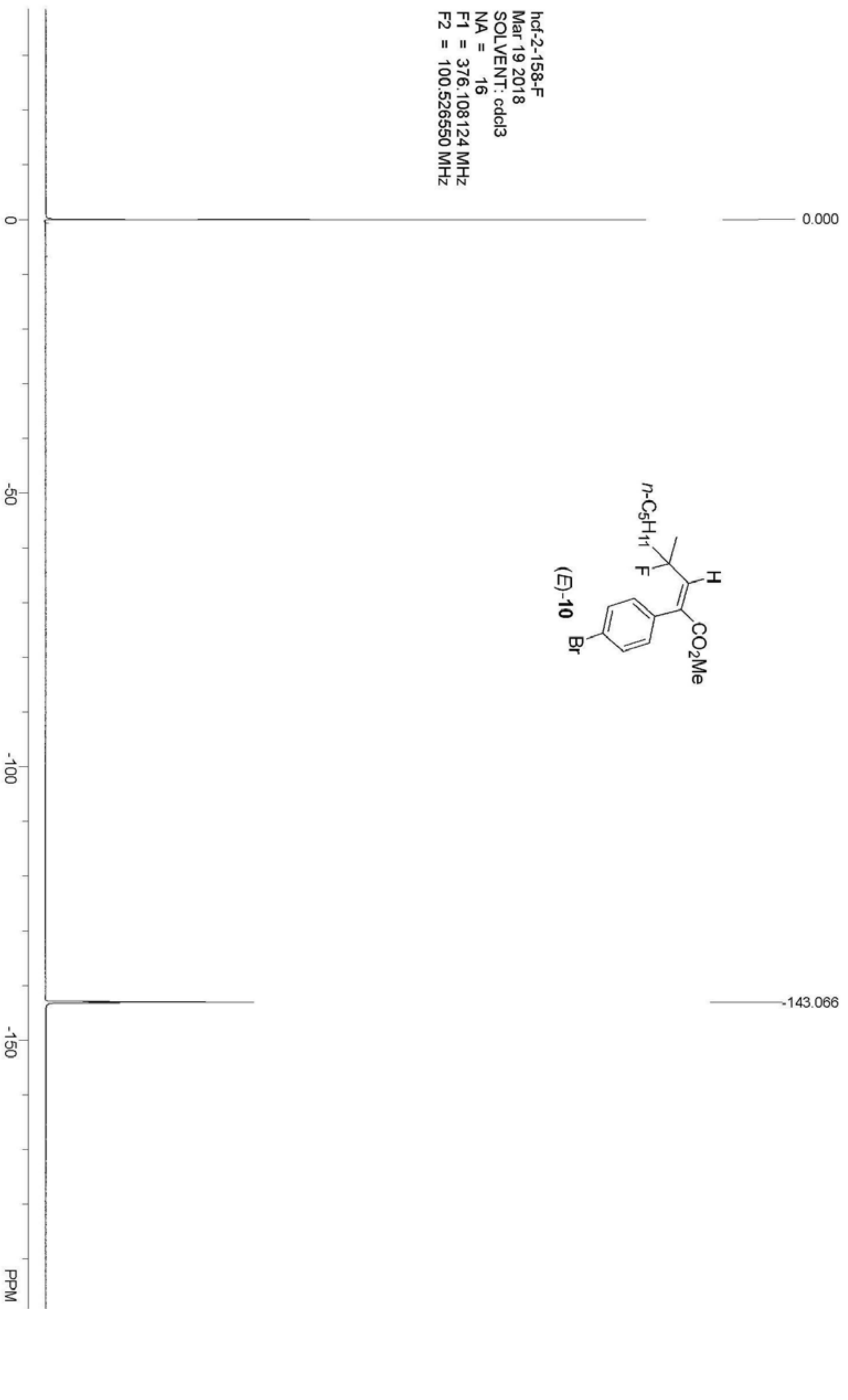
hcf-2-158-F
Mar 19 2018
SOLVENT: cdcl3
NA = 16
F1 = 376.108124 MHz
F2 = 100.526550 MHz
0.000
-143.066
n-C5H11
F
H
CO2Me
Br
(E)-10
0
-50
-100
-150
PPM

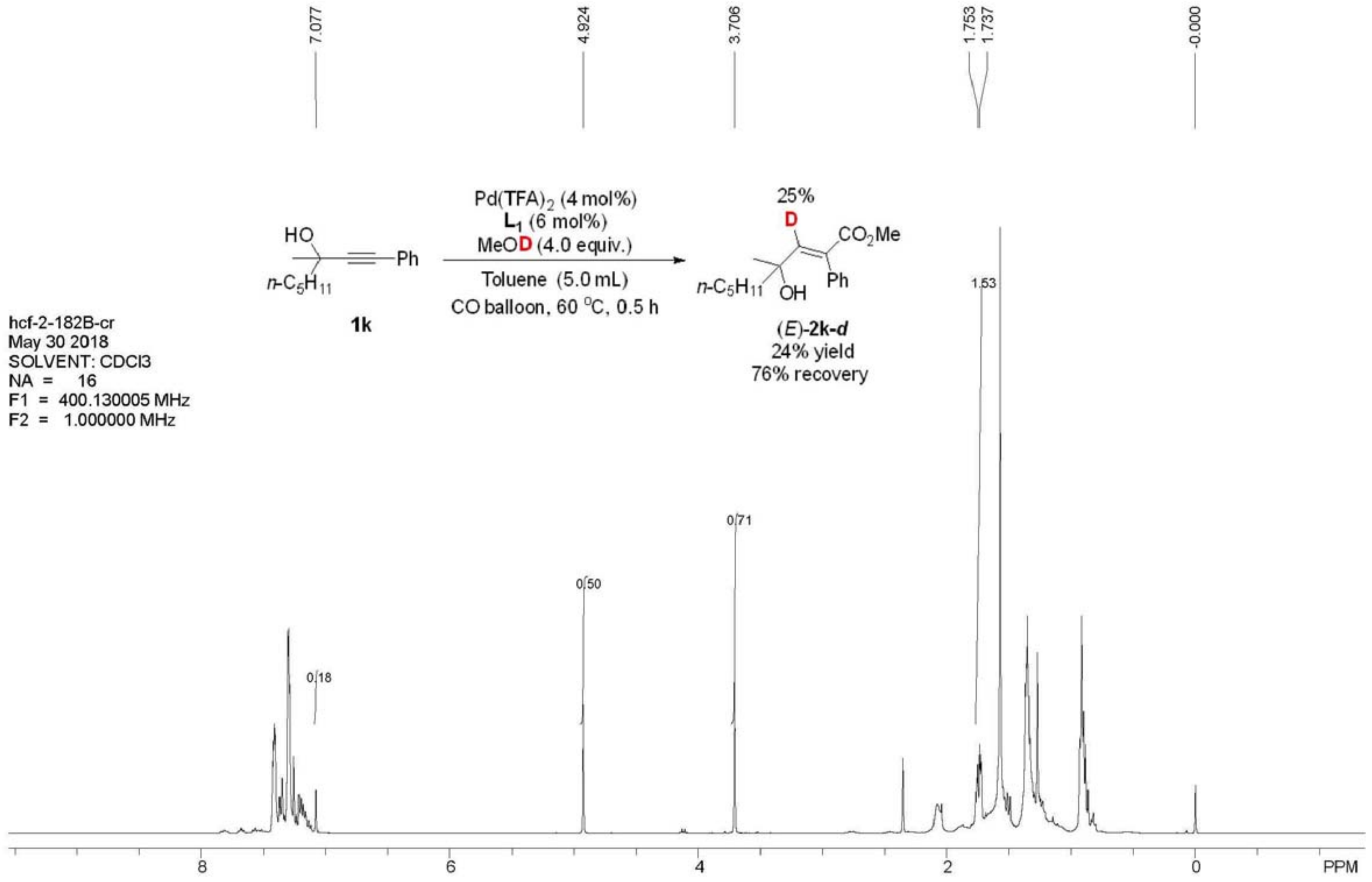
7.077
4.924
3.706
1.753
1.737
-0.000
HO
Ph
n-C5H11
1k
Pd(TFA)2 (4 mol%)
L1 (6 mol%)
MeOD (4.0 equiv.)
Toluene (5.0 mL)
CO balloon, 60 °C, 0.5 h
25%
D
CO2Me
n-C5H11
OH
Ph
(E)-2k-d
24% yield
76% recovery
hcf-2-182B-cr
May 30 2018
SOLVENT: CDCl3
NA = 16
F1 = 400.130005 MHz
F2 = 1.000000 MHz
0.18
0.50
0.71
1.53
8
6
4
2
0
PPM

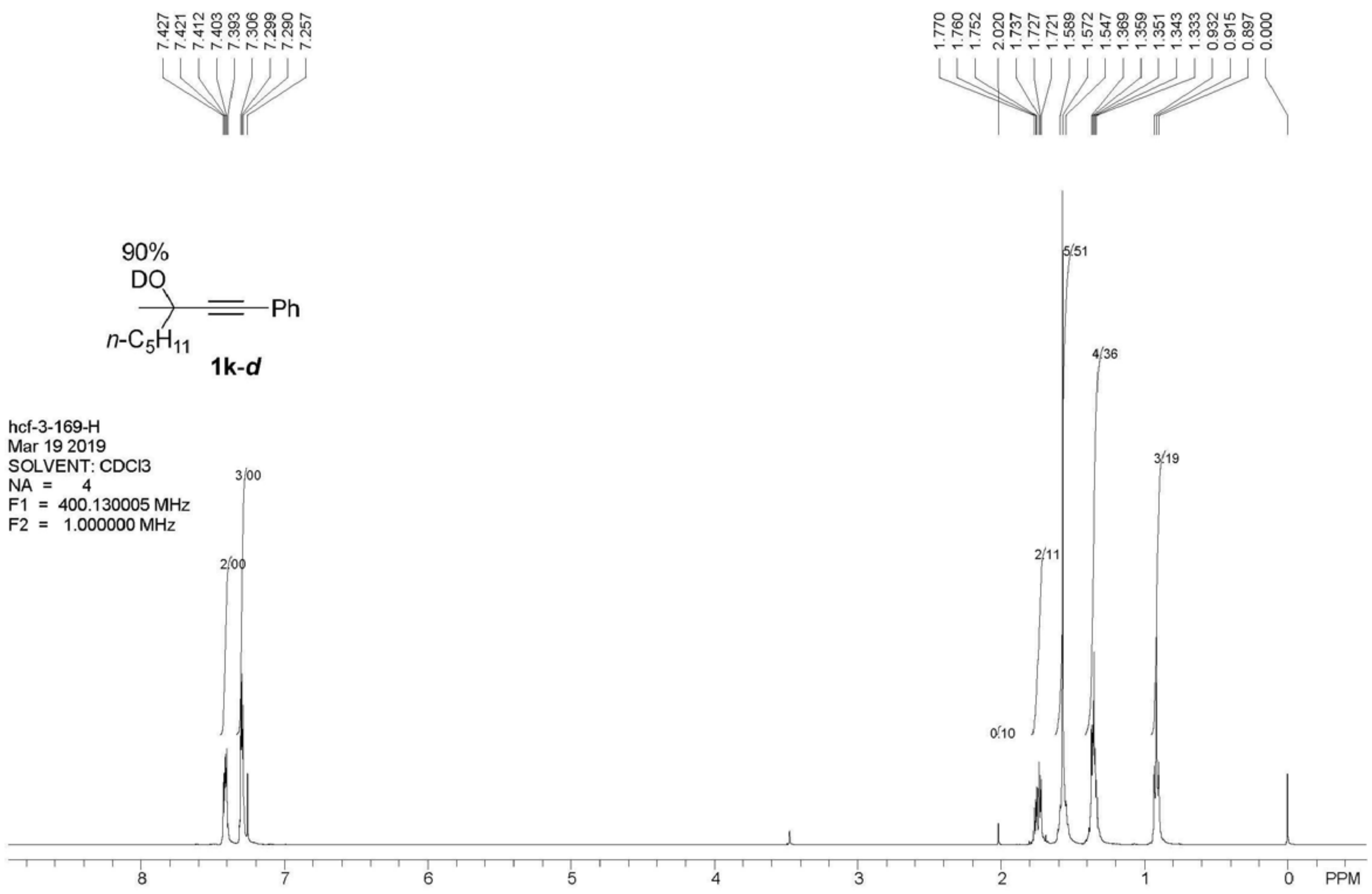
7.427
7.421
7.412
7.403
7.393
7.306
7.299
7.290
7.257
1.770
1.760
1.752
2.020
1.737
1.727
1.721
1.589
1.572
1.547
1.369
1.359
1.351
1.343
1.333
0.932
0.915
0.897
0.000
90%
DO
Ph
n-C5H11
1k-d
hcf-3-169-H
Mar 19 2019
SOLVENT: CDCl3
NA = 4
F1 = 400.130005 MHz
F2 = 1.000000 MHz
2.00
3.00
0.10
2.11
5.51
4.36
3.19
8
7
6
5
4
3
2
1
0
PPM

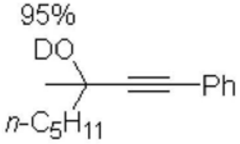
95%
DO
Ph
n-C5H11

1k-*d*

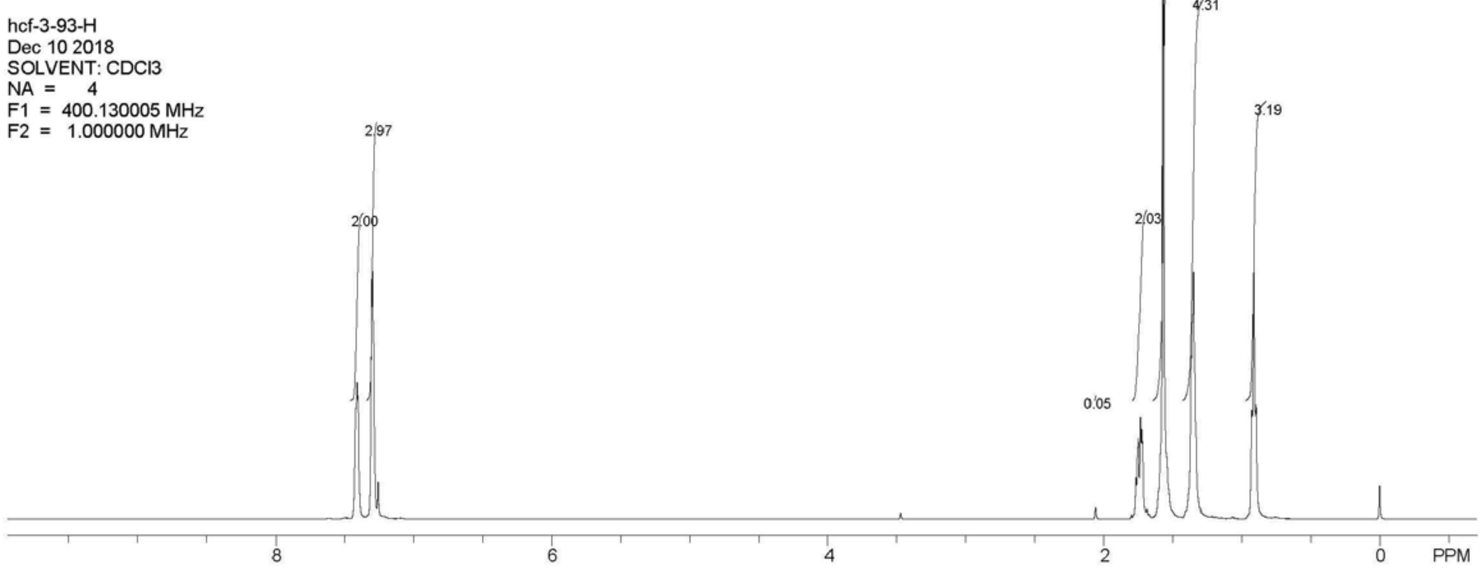
hcf-3-93-H
Dec 10 2018
SOLVENT: CDCl3
NA = 4
F1 = 400.130005 MHz
F2 = 1.000000 MHz
7.420
7.412
7.404
7.303
7.297
7.256
2.063
1.769
1.752
1.736
1.727
1.721
1.571
1.366
1.349
0.930
0.914
0.898
0.000
2.00
2.97
0.05
2.03
5.38
4.31
3.19
8
6
4
2
0
PPM

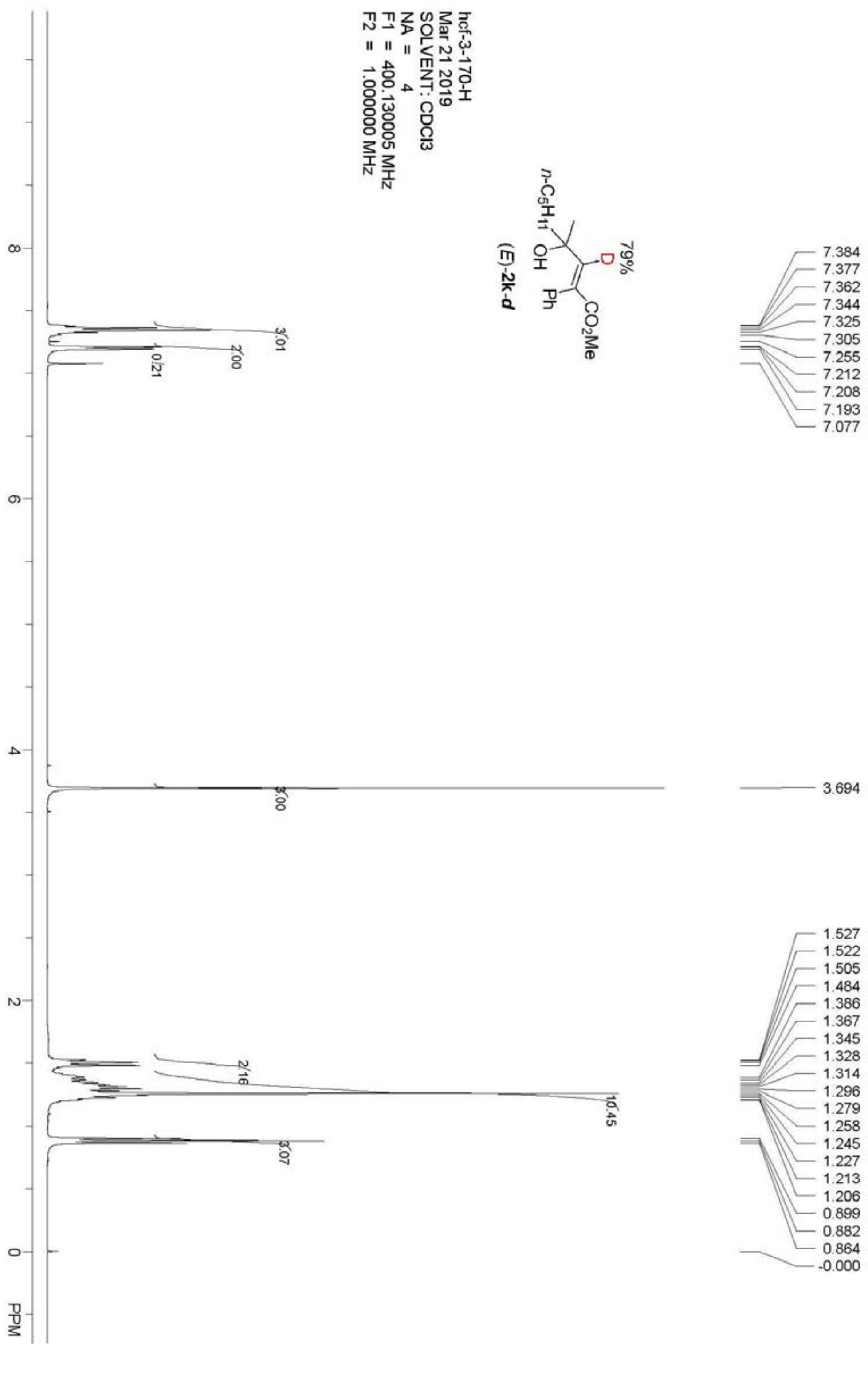
hcf-3-170-H
Mar 21 2019
SOLVENT: CDCl3
NA = 4
F1 = 400.130005 MHz
F2 = 1.000000 MHz
79%
D
CO2Me
n-C5H11
OH
Ph
(E)-2k-d
7.384
7.377
7.362
7.344
7.325
7.305
7.255
7.212
7.208
7.193
7.077
3.694
1.527
1.522
1.505
1.484
1.386
1.367
1.345
1.328
1.314
1.296
1.279
1.258
1.245
1.227
1.213
1.206
0.899
0.882
0.864
-0.000
3.01
2.00
0.21
3.00
2.16
10.45
3.07
8
6
4
2
0
PPM

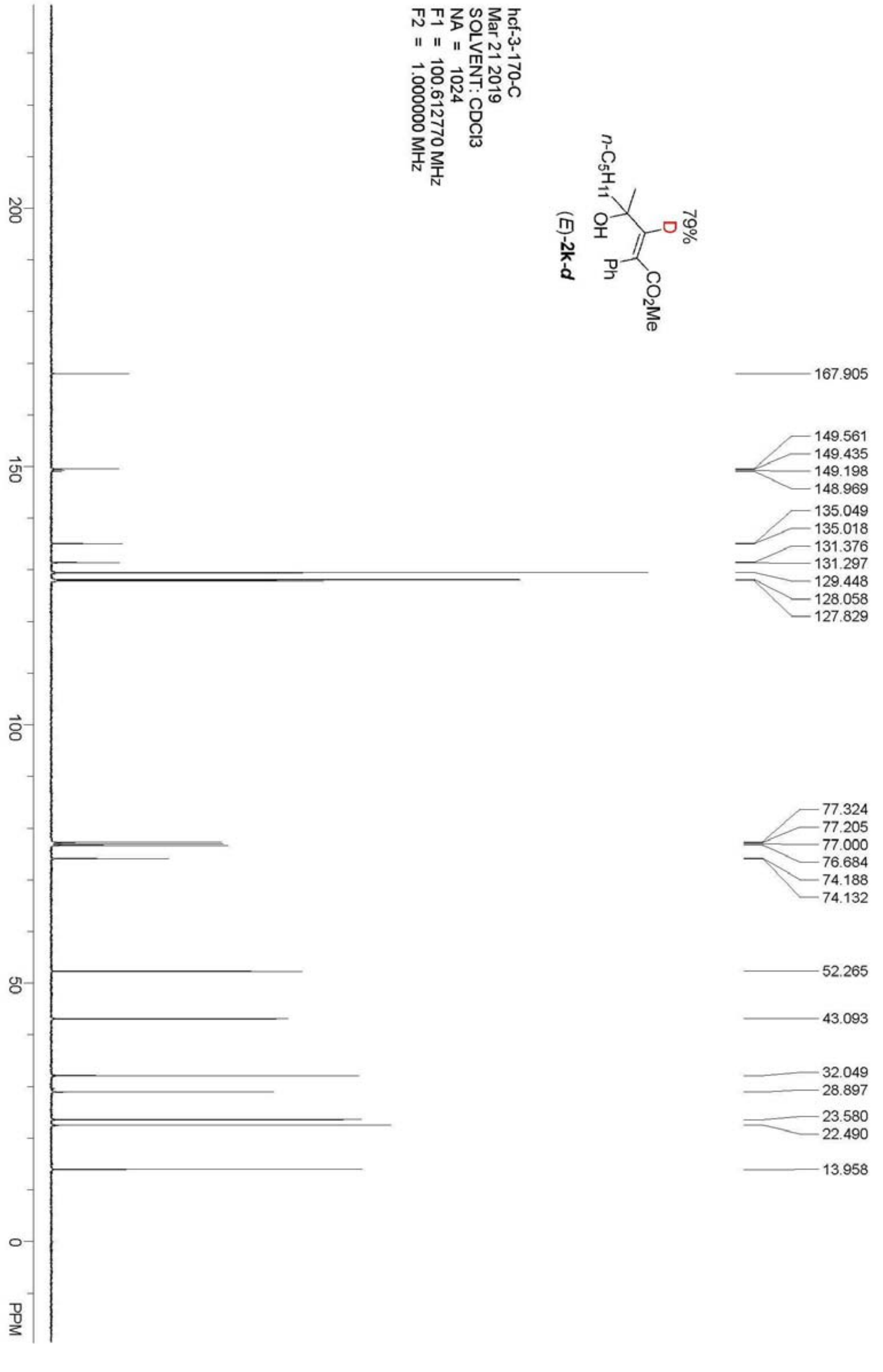
hcf-3-170-C
Mar 21 2019
SOLVENT: CDCl3
NA = 1024
F1 = 100.612770 MHz
F2 = 1.000000 MHz
79%
D
CO2Me
Ph
OH
n-C5H11
(E)-2k-d
167.905
149.561
149.435
149.198
148.969
135.049
135.018
131.376
131.297
129.448
128.058
127.829
77.324
77.205
77.000
76.684
74.188
74.132
52.265
43.093
32.049
28.897
23.580
22.490
13.958
200
150
100
50
0
PPM

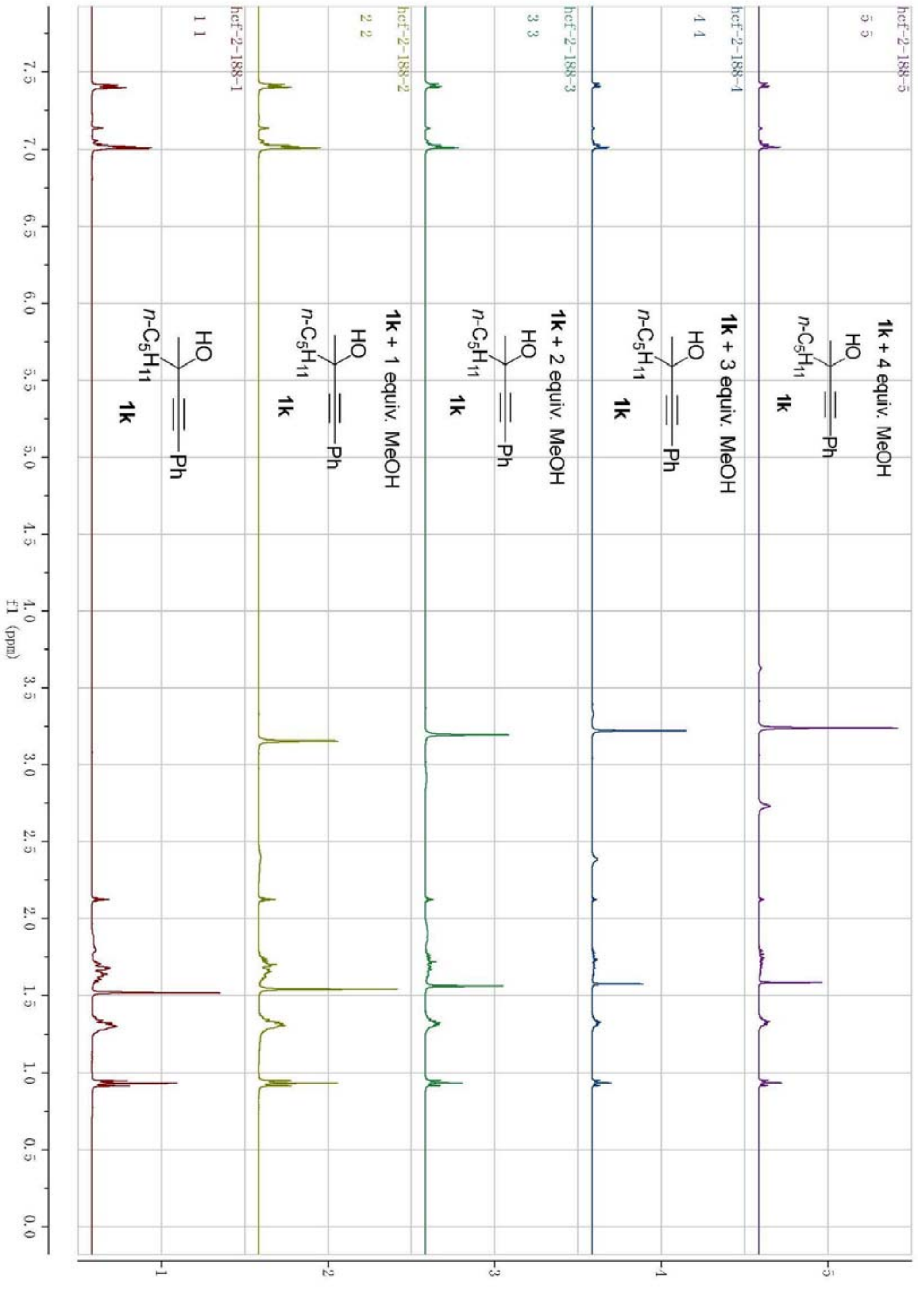

1k + 4 equiv. MeOH
1k + 3 equiv. MeOH
1k + 2 equiv. MeOH
1k + 1 equiv. MeOH
HO
n-C5H11
Ph
1k
hcf-2-188-5
hcf-2-188-4
hcf-2-188-3
hcf-2-188-2
hcf-2-188-1
f1 (ppm)

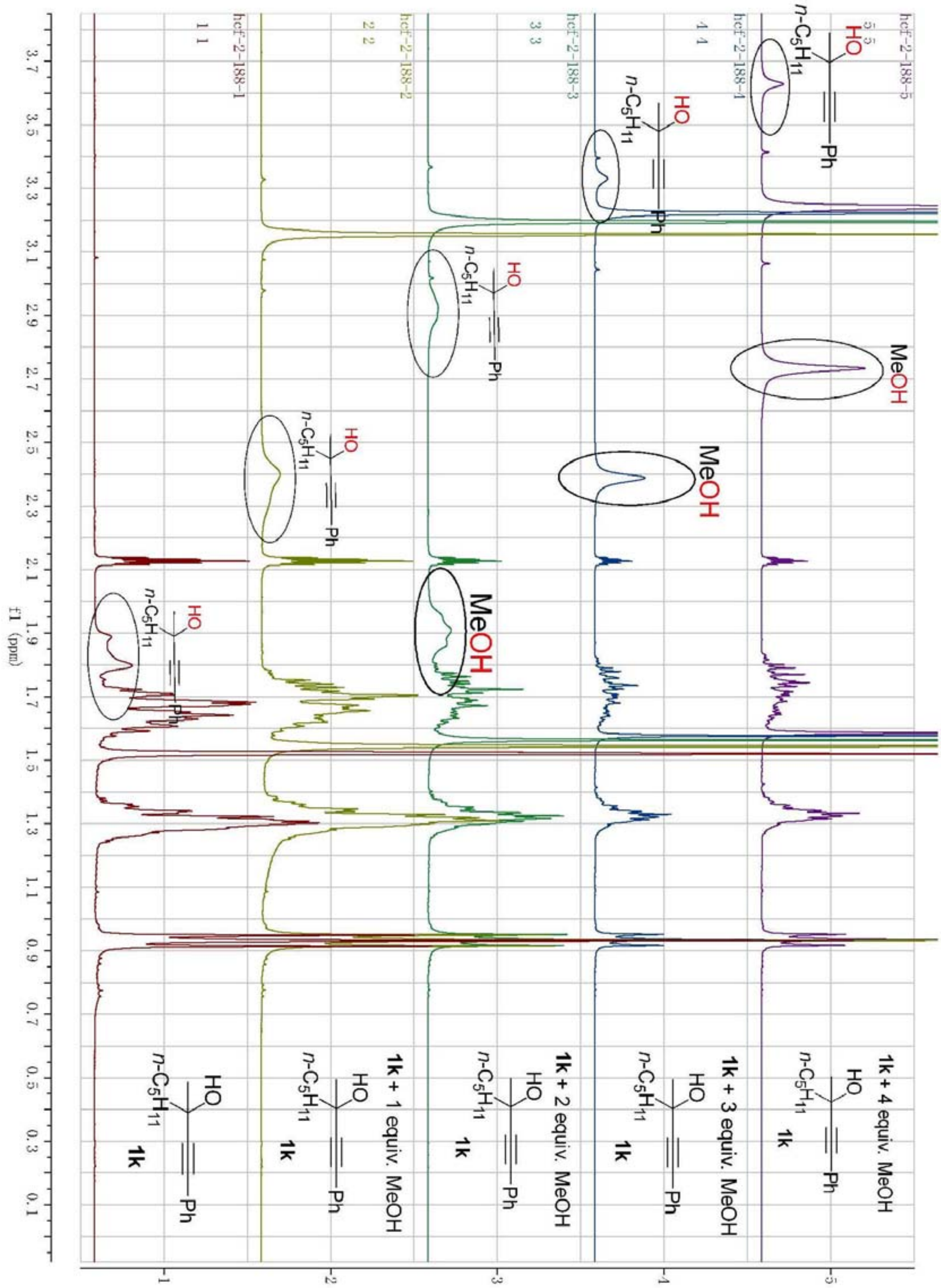
1k + 4 equiv. MeOH
1k + 3 equiv. MeOH
1k + 2 equiv. MeOH
1k + 1 equiv. MeOH
1k
MeOH
MeOH
MeOH
f1 (ppm)

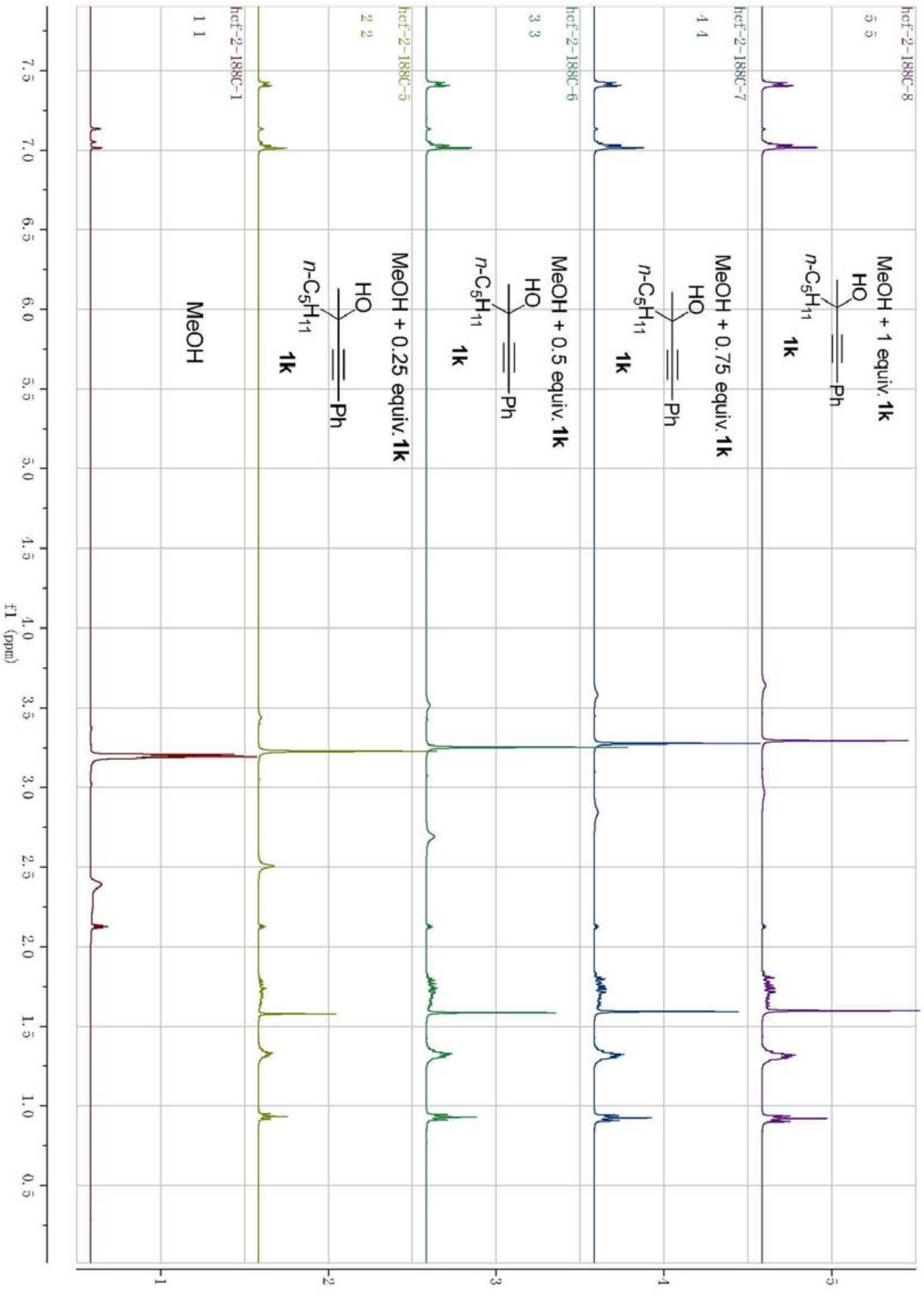
MeOH + 1 equiv. 1k
MeOH + 0.75 equiv. 1k
MeOH + 0.5 equiv. 1k
MeOH + 0.25 equiv. 1k
MeOH
1k
f1 (ppm)

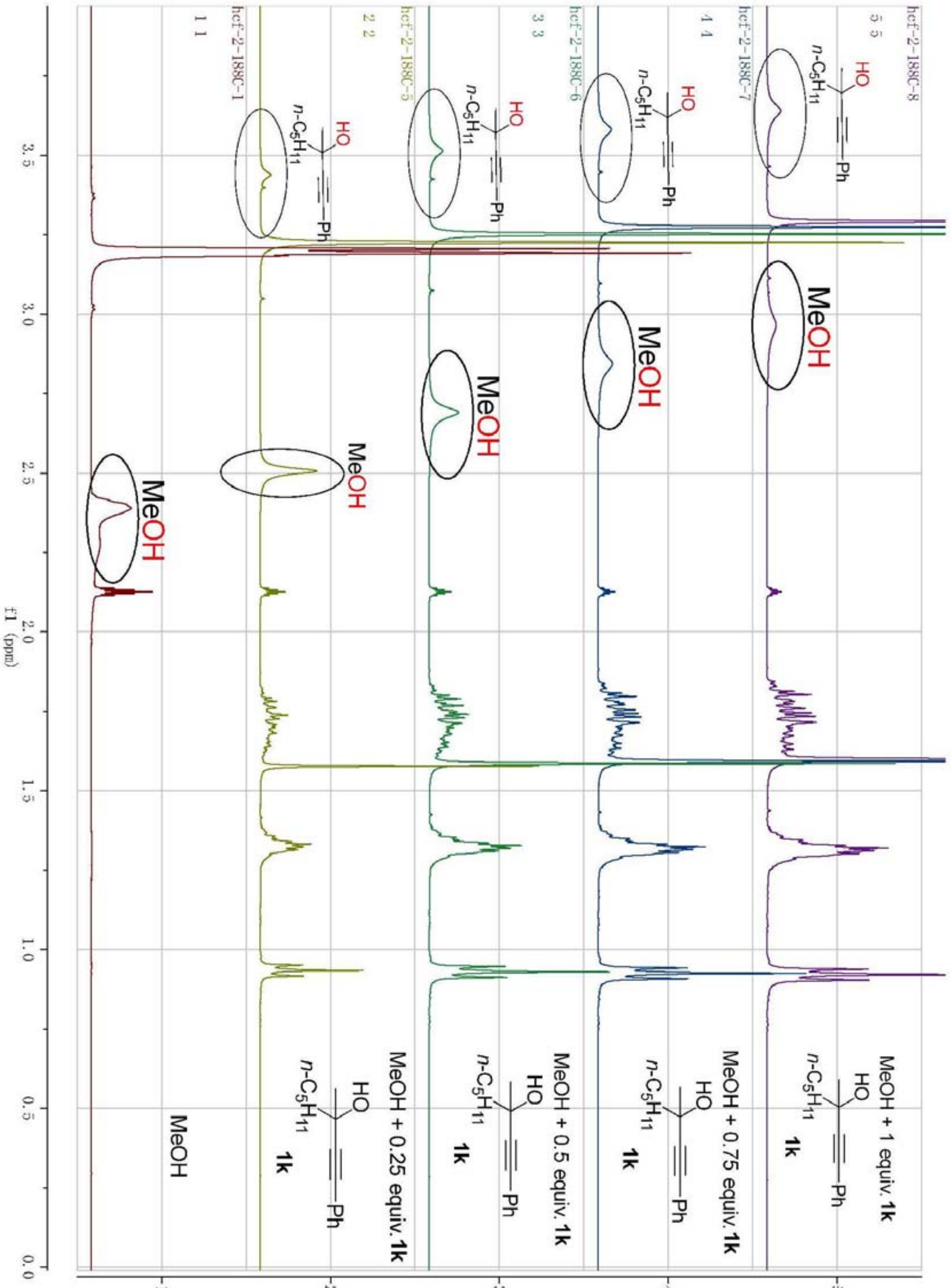
MeOH + 1 equiv. 1k
MeOH + 0.75 equiv. 1k
MeOH + 0.5 equiv. 1k
MeOH + 0.25 equiv. 1k
MeOH
hcf-2-188C-8
hcf-2-188C-7
hcf-2-188C-6
hcf-2-188C-5
hcf-2-188C-1
f1 (ppm)

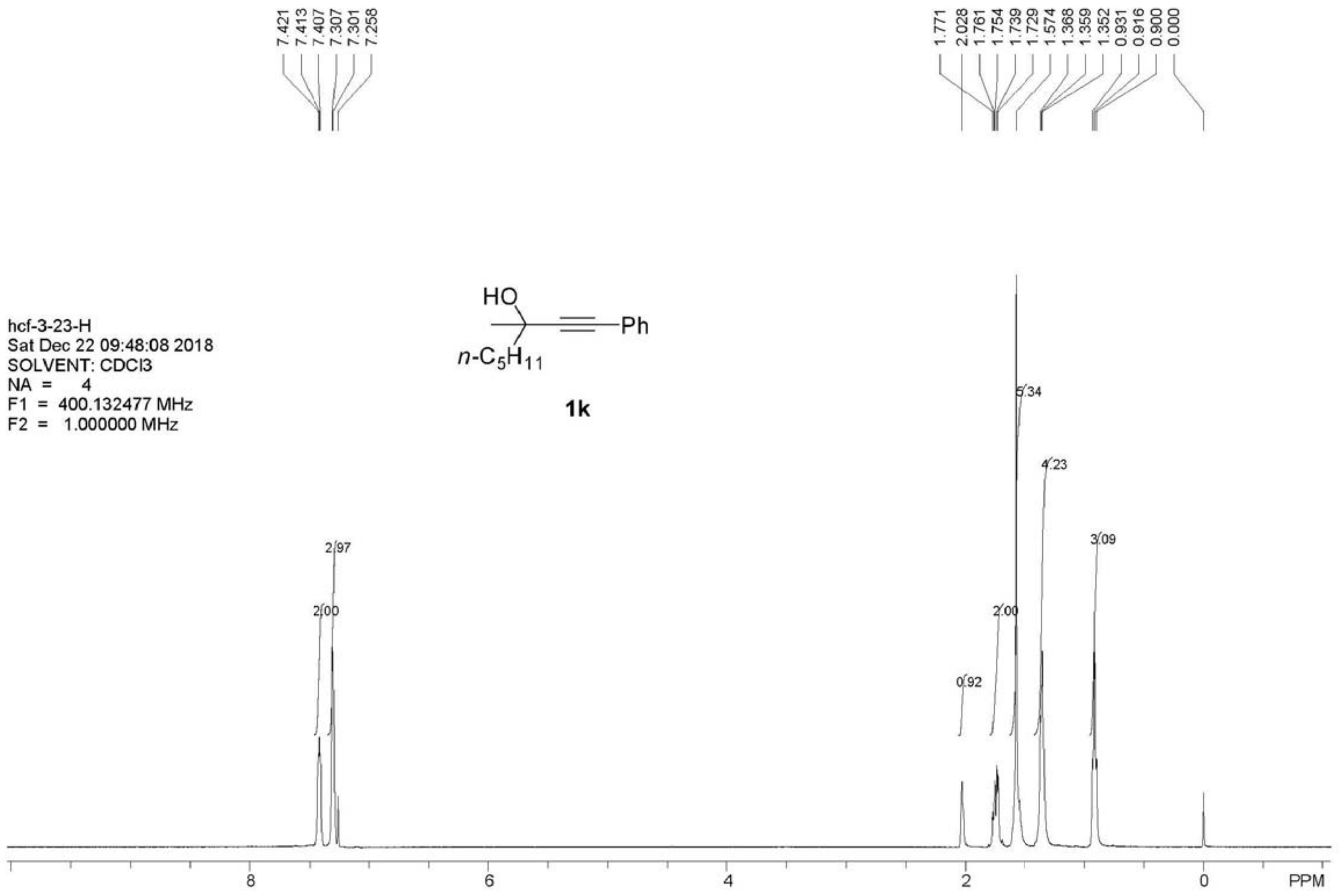
hcf-3-23-H
Sat Dec 22 09:48:08 2018
SOLVENT: CDCl3
NA = 4
F1 = 400.132477 MHz
F2 = 1.000000 MHz
HO
Ph
n-C5H11
1k
7.421
7.413
7.407
7.307
7.301
7.258
1.771
2.028
1.761
1.754
1.739
1.729
1.574
1.368
1.359
1.352
0.931
0.916
0.900
0.000
2.00
2.97
0.92
2.00
5.34
4.23
3.09
8
6
4
2
0
PPM

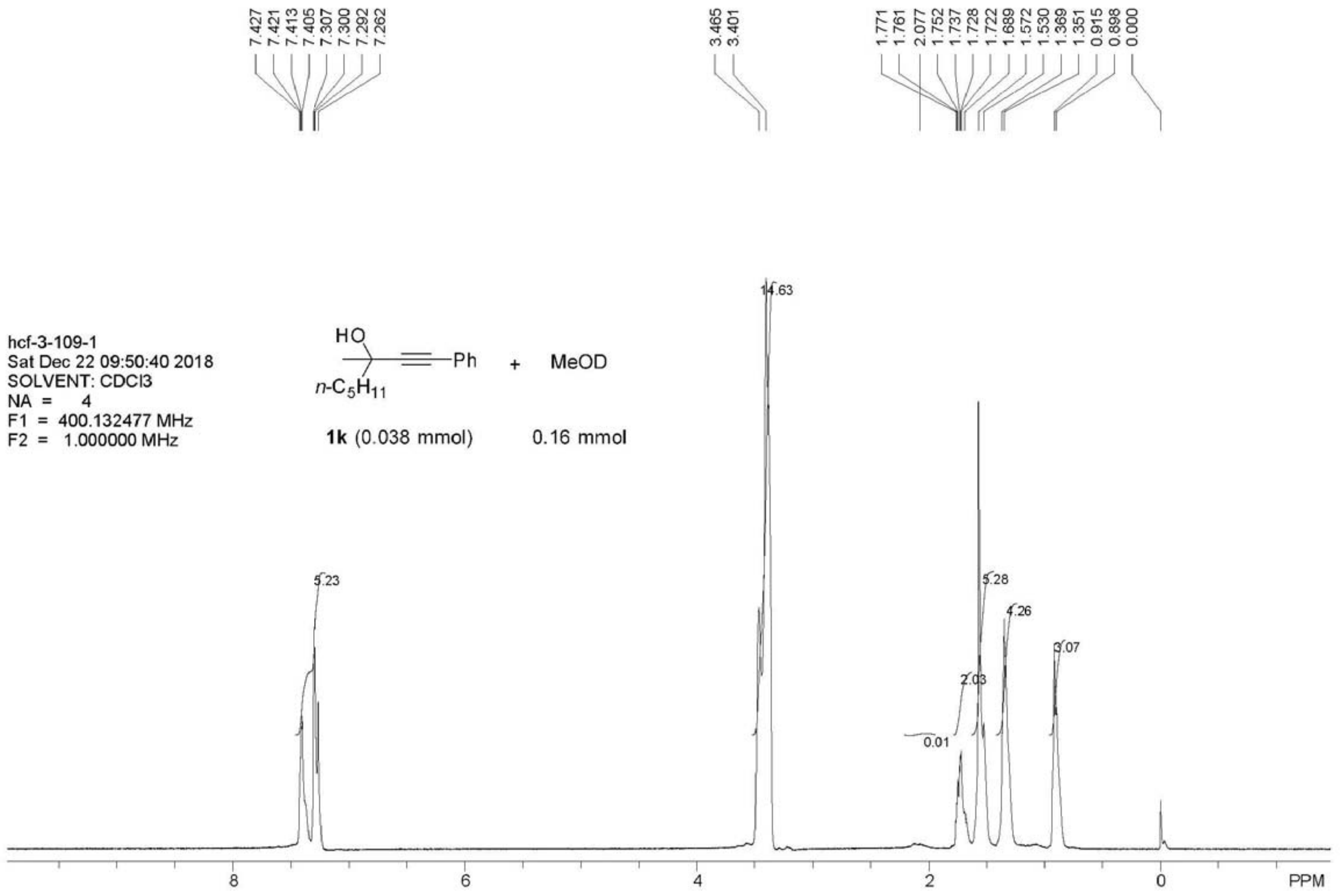
7.427
7.421
7.413
7.405
7.307
7.300
7.292
7.262
3.465
3.401
1.771
1.761
2.077
1.752
1.737
1.728
1.722
1.689
1.572
1.530
1.369
1.351
0.915
0.898
0.000
hcf-3-109-1
Sat Dec 22 09:50:40 2018
SOLVENT: CDCl3
NA = 4
F1 = 400.132477 MHz
F2 = 1.000000 MHz
HO
Ph
n-C5H11
+ MeOD
1k (0.038 mmol)
0.16 mmol
14.63
5.23
5.28
4.26
3.07
2.03
0.01
8
6
4
2
0
PPM

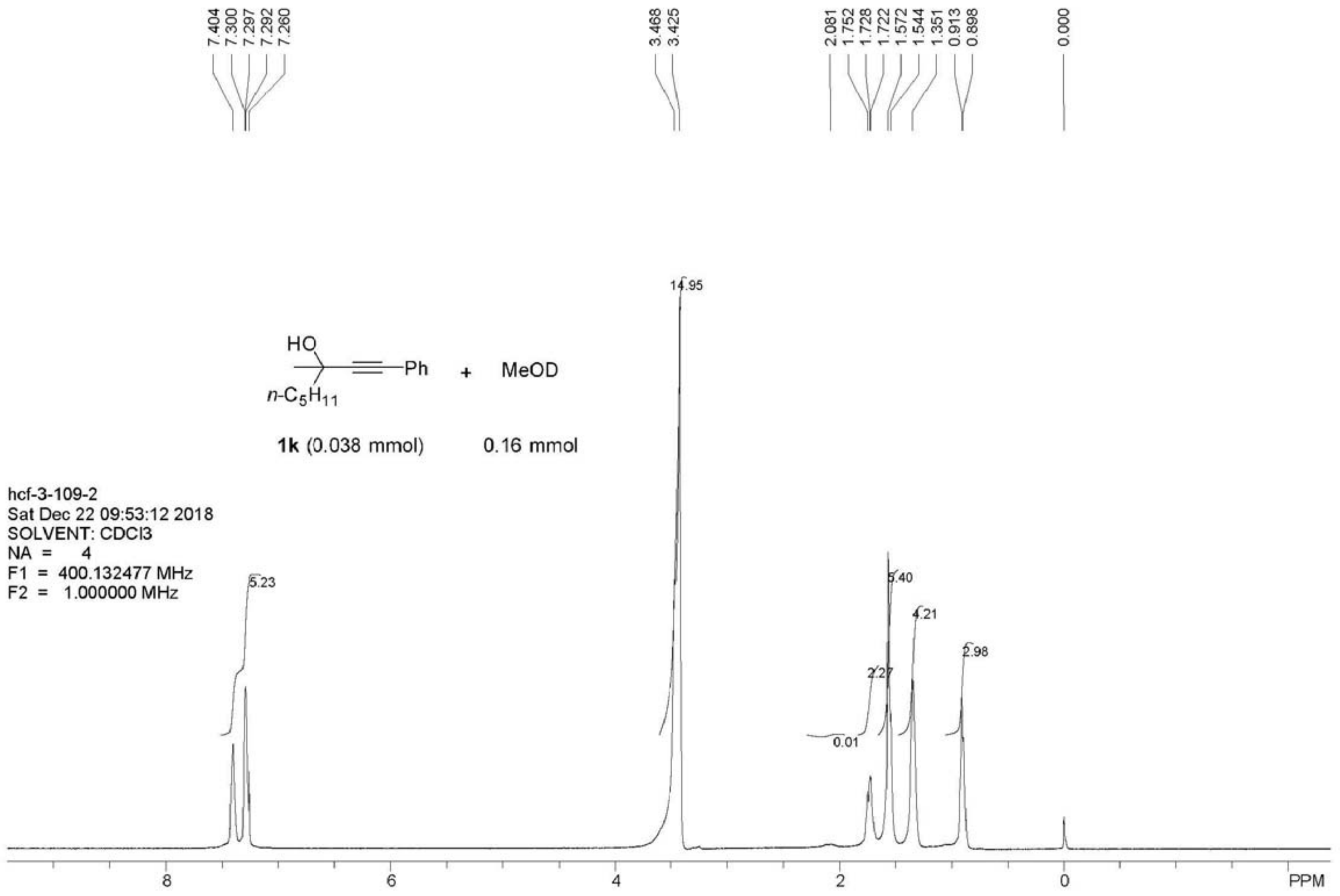
7.404
7.300
7.297
7.292
7.260
3.468
3.425
2.081
1.752
1.728
1.722
1.572
1.544
1.351
0.913
0.898
0.000
14.95
HO
Ph
+
MeOD
n-C_5H_{11}
1k (0.038 mmol)
0.16 mmol
hcf-3-109-2
Sat Dec 22 09:53:12 2018
SOLVENT: CDCl3
NA = 4
F1 = 400.132477 MHz
F2 = 1.000000 MHz
5.23
5.40
4.21
2.27
2.98
0.01
8
6
4
2
0
PPM

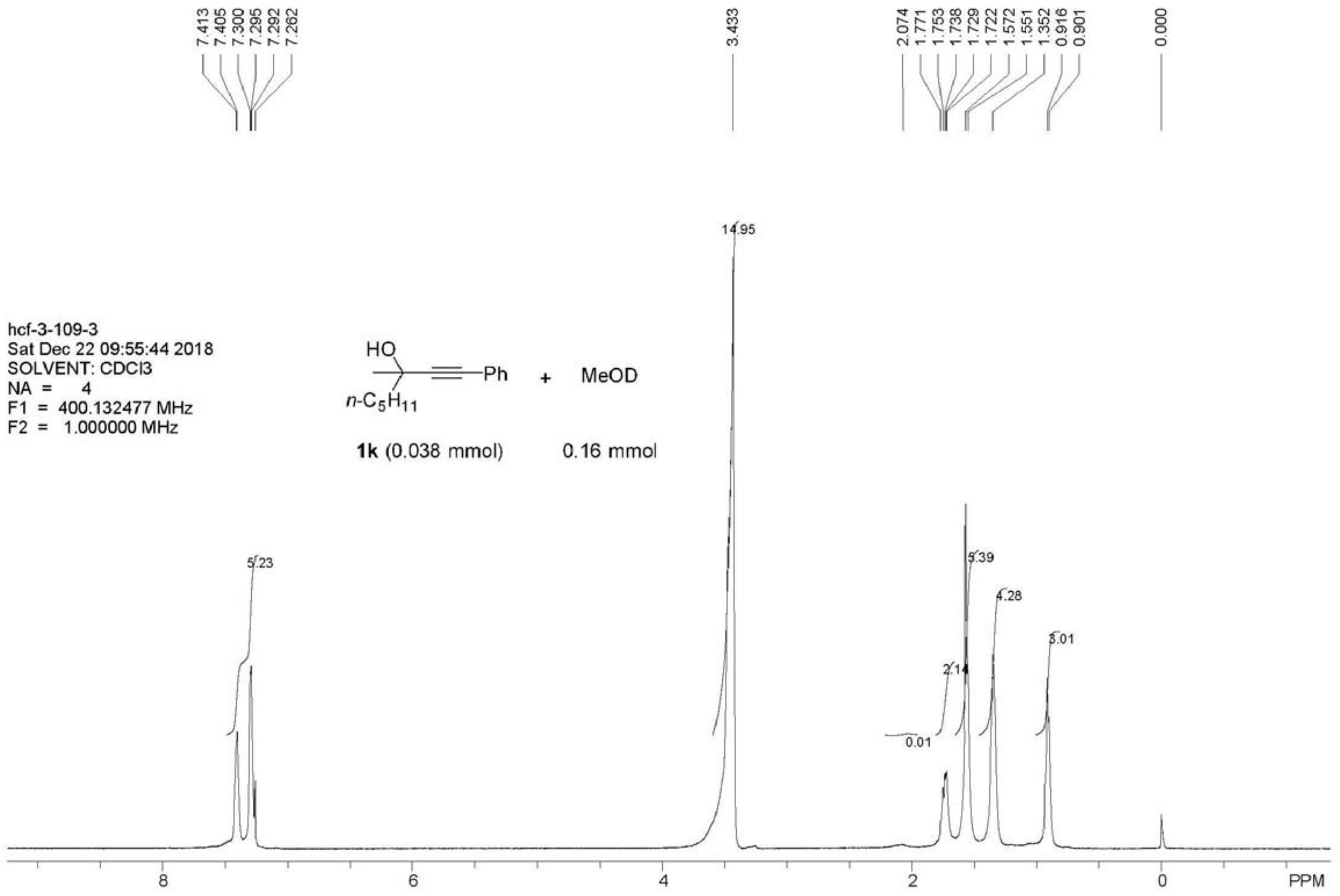
7.413
7.405
7.300
7.295
7.292
7.262
3.433
2.074
1.771
1.753
1.738
1.729
1.722
1.572
1.551
1.352
0.916
0.901
0.000
hcf-3-109-3
Sat Dec 22 09:55:44 2018
SOLVENT: CDCl3
NA = 4
F1 = 400.132477 MHz
F2 = 1.000000 MHz
HO
Ph
n-C5H11
+ MeOD
1k (0.038 mmol)
0.16 mmol
14.95
5.23
5.39
4.28
3.01
2.14
0.01
8
6
4
2
0
PPM

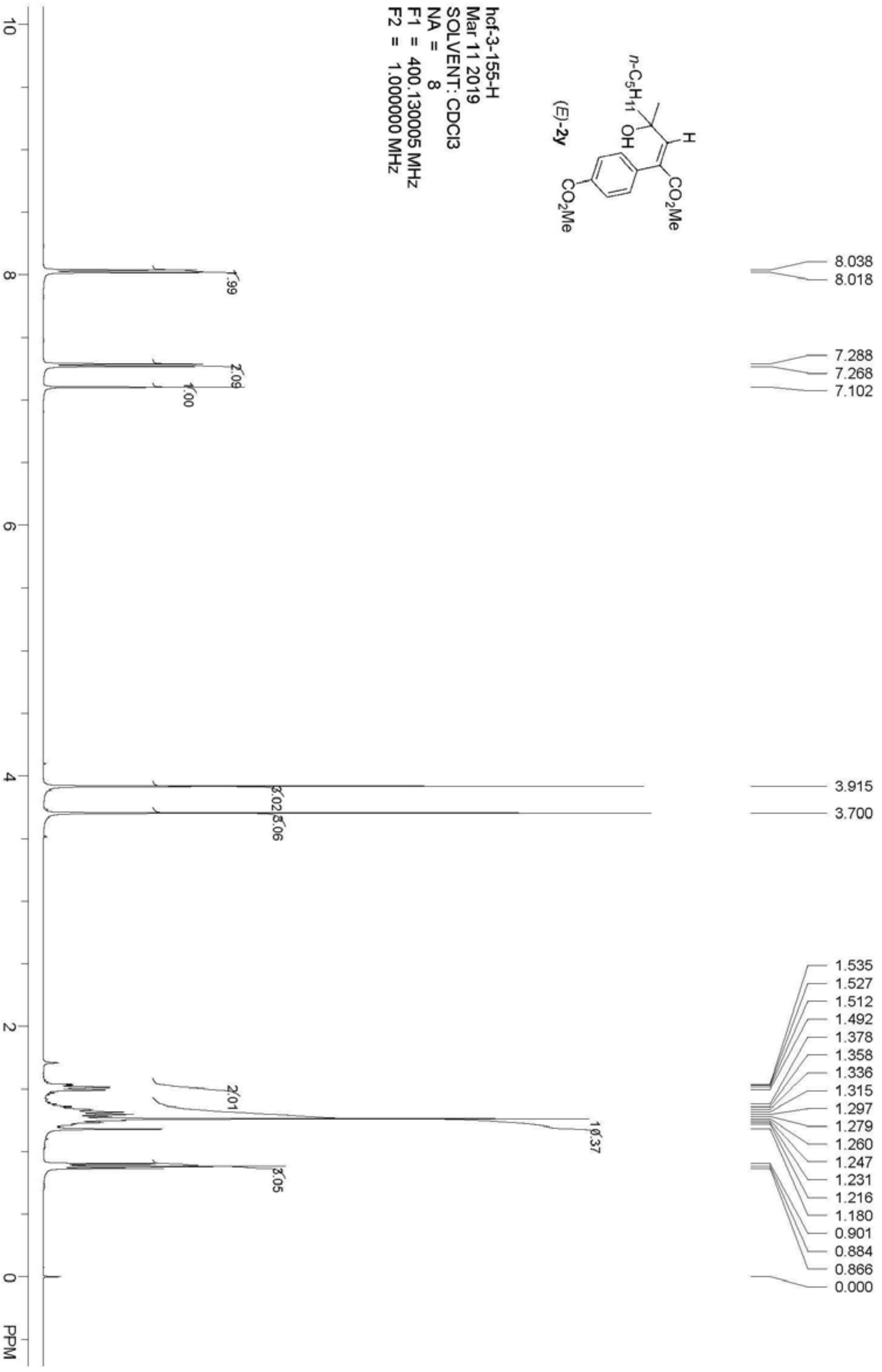

hcf-3-155-H
Mar 11 2019
SOLVENT: CDCl3
NA = 8
F1 = 400.130005 MHz
F2 = 1.000000 MHz
(E)-2y
CO2Me
n-C5H11
OH
H
8.038
8.018
7.288
7.268
7.102
3.915
3.700
1.535
1.527
1.512
1.492
1.378
1.358
1.336
1.315
1.297
1.279
1.260
1.247
1.231
1.216
1.180
0.901
0.884
0.866
0.000
1.99
2.09
1.00
3.02
3.06
2.01
10.37
3.05
10
8
6
4
2
0
PPM

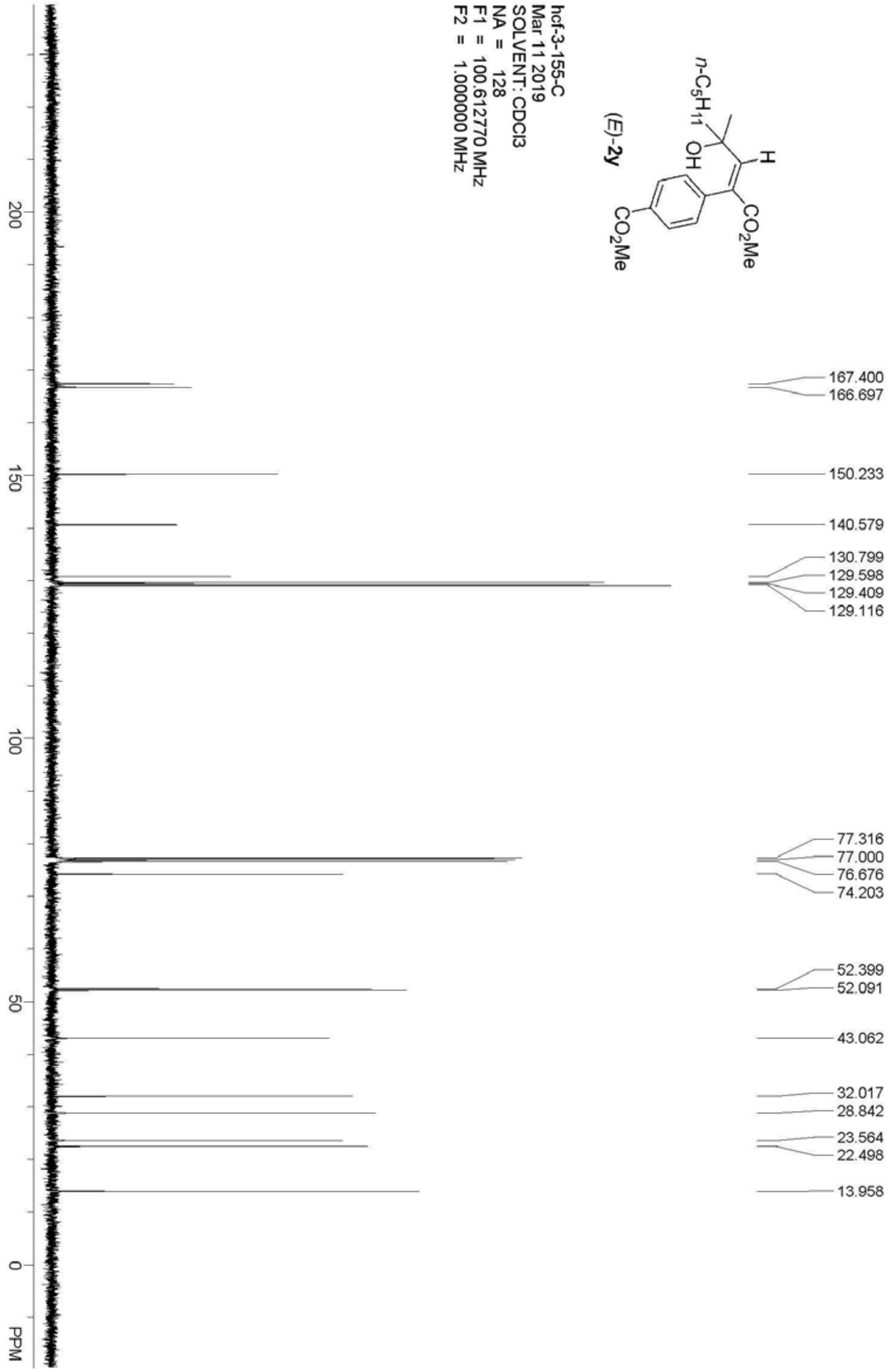
hcf-3-155-C
Mar 11 2019
SOLVENT: CDCl3
NA = 128
F1 = 100.612770 MHz
F2 = 1.000000 MHz
(E)-2y
n-C5H11
OH
H
CO2Me
CO2Me
167.400
166.697
150.233
140.579
130.799
129.598
129.409
129.116
77.316
77.000
76.676
74.203
52.399
52.091
43.062
32.017
28.842
23.564
22.498
13.958
200
150
100
50
0
PPM

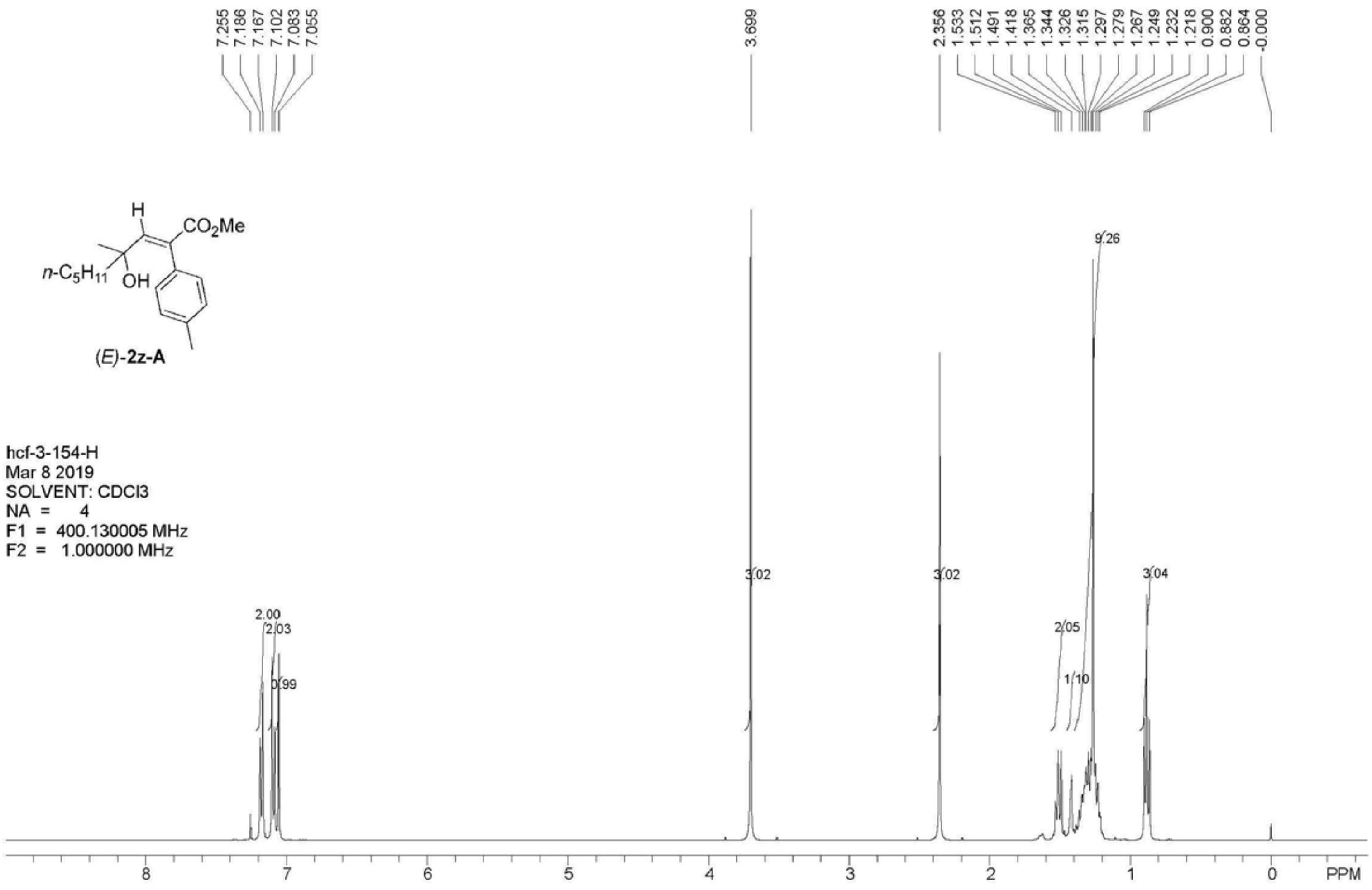
7.255
7.186
7.167
7.102
7.083
7.055
3.699
2.356
1.533
1.512
1.491
1.418
1.365
1.344
1.326
1.315
1.297
1.279
1.267
1.249
1.232
1.218
0.900
0.882
0.864
-0.000
H
CO_2Me
n-C_5H_{11}
OH
(E)-**2z-A**
hcf-3-154-H
Mar 8 2019
SOLVENT: CDCl3
NA = 4
F1 = 400.130005 MHz
F2 = 1.000000 MHz
2.00
2.03
0.99
3.02
3.02
2.05
1.10
9.26
3.04
8
7
6
5
4
3
2
1
0
PPM

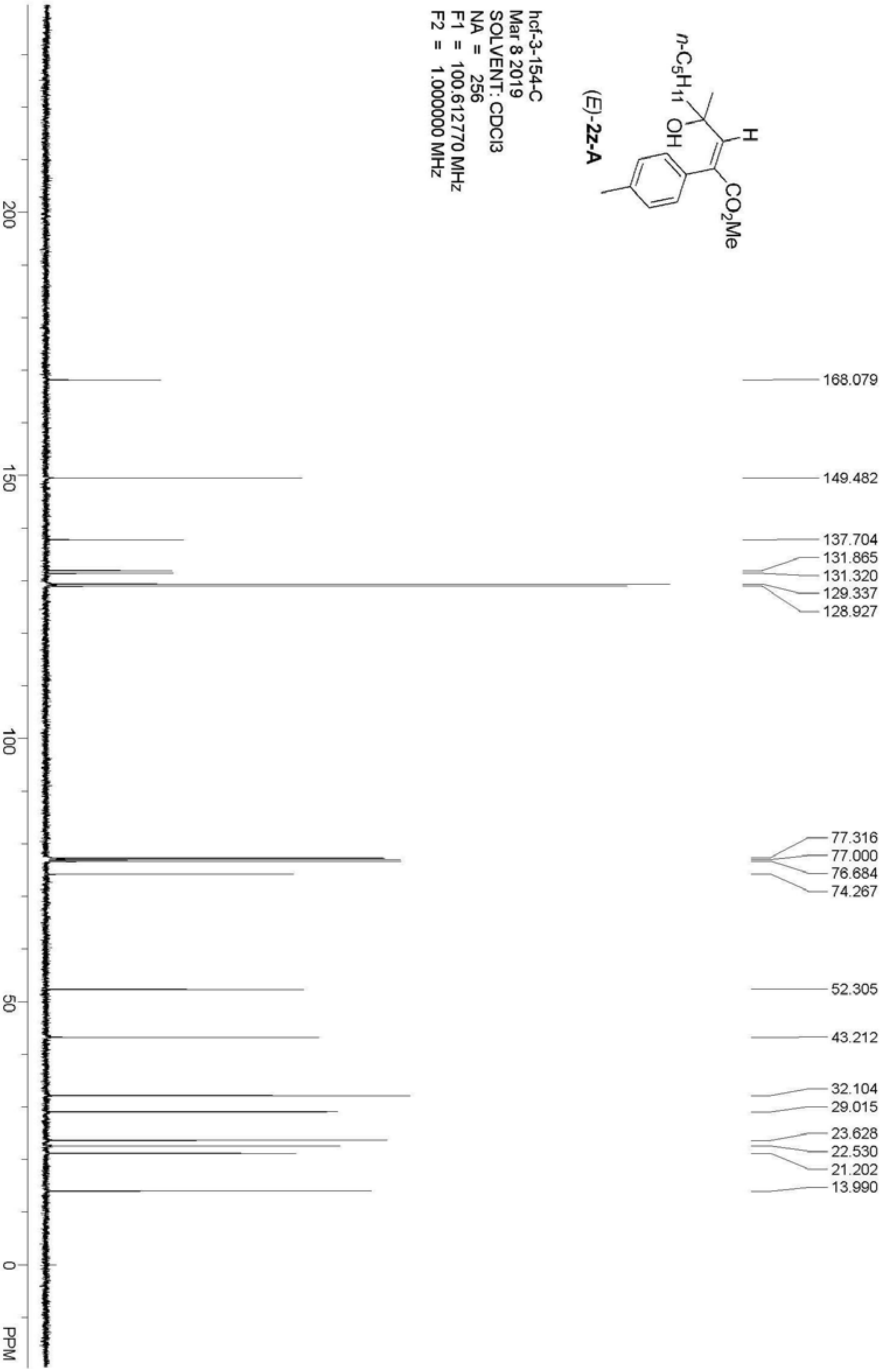

(E)-2z-A
n-C5H11
OH
H
CO2Me
hcf-3-154-C
Mar 8 2019
SOLVENT: CDCl3
NA = 256
F1 = 100.612770 MHz
F2 = 1.000000 MHz
168.079
149.482
137.704
131.865
131.320
129.337
128.927
77.316
77.000
76.684
74.267
52.305
43.212
32.104
29.015
23.628
22.530
21.202
13.990
200
150
100
50
0
PPM

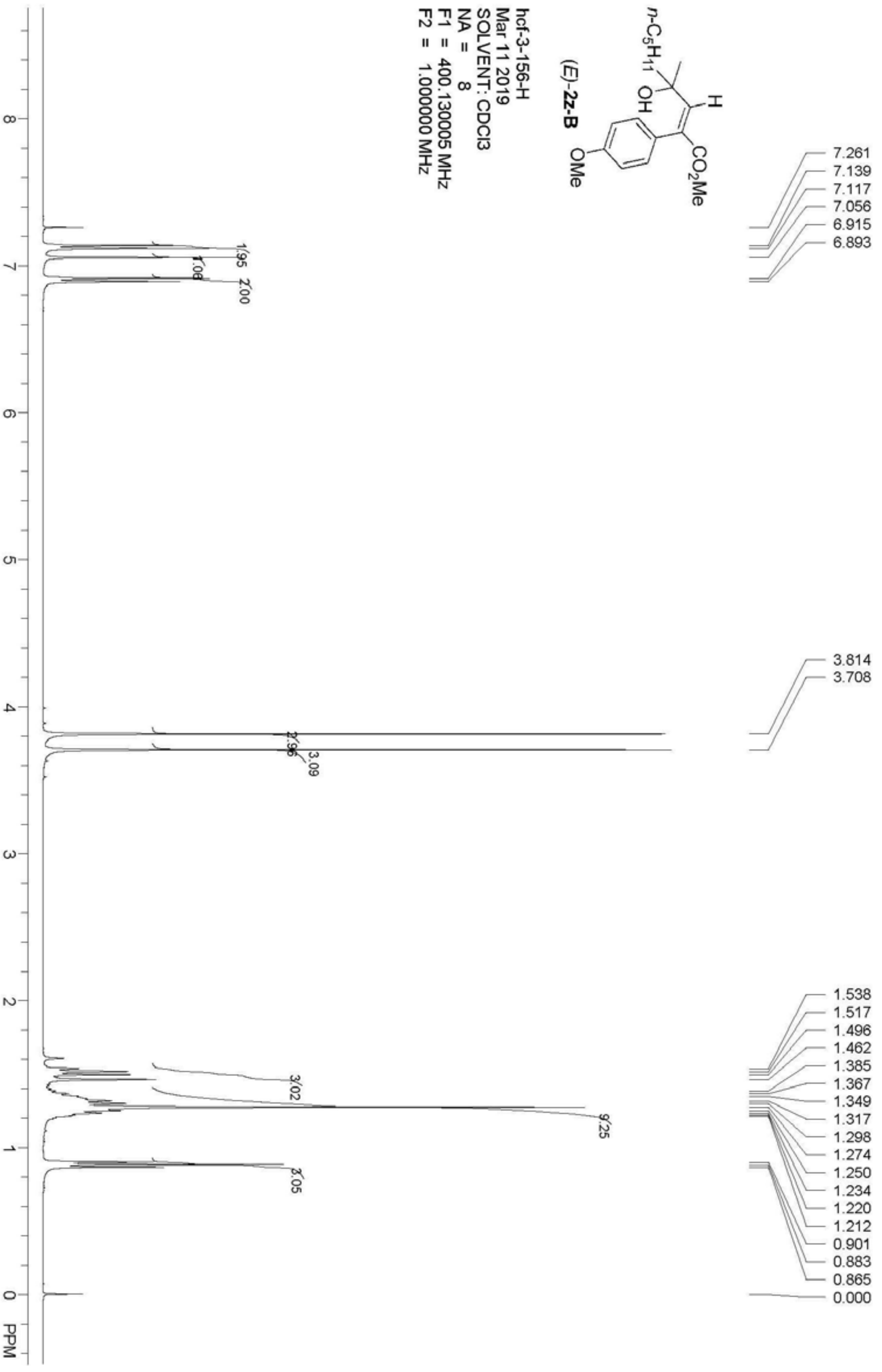

(E)-2z-B
hcf-3-156-H
Mar 11 2019
SOLVENT: CDCl3
NA = 8
F1 = 400.130005 MHz
F2 = 1.000000 MHz
7.261
7.139
7.117
7.056
6.915
6.893
3.814
3.708
1.538
1.517
1.496
1.462
1.385
1.367
1.349
1.317
1.298
1.274
1.250
1.234
1.220
1.212
0.901
0.883
0.865
0.000
1.95
1.06
2.00
2.96
3.09
3.02
9.25
3.05
PPM

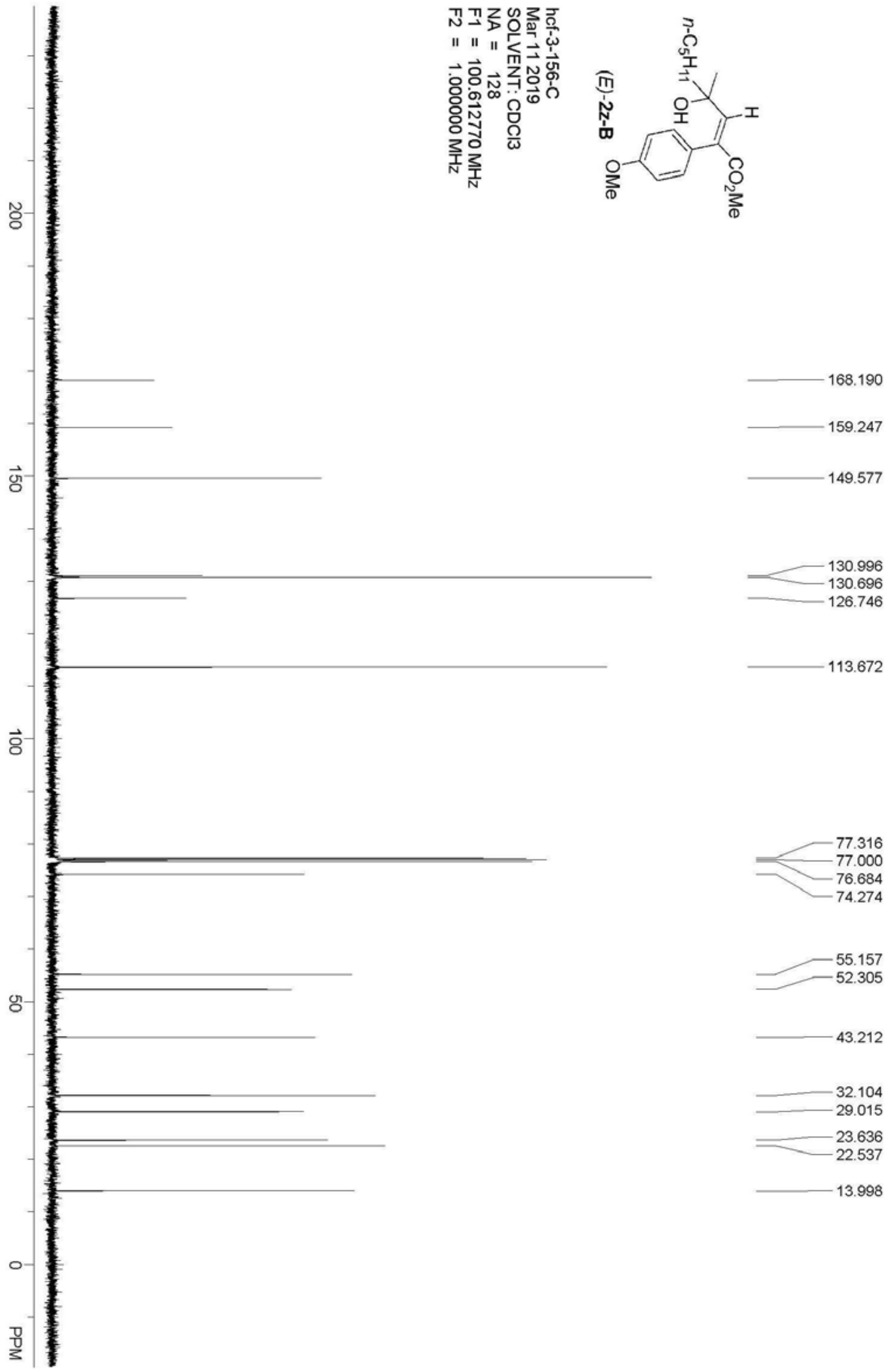

(E)-2z-B
hcf-3-156-C
Mar 11 2019
SOLVENT: CDCl3
NA = 128
F1 = 100.612770 MHz
F2 = 1.000000 MHz
168.190
159.247
149.577
130.996
130.696
126.746
113.672
77.316
77.000
76.684
74.274
55.157
52.305
43.212
32.104
29.015
23.636
22.537
13.998
PPM

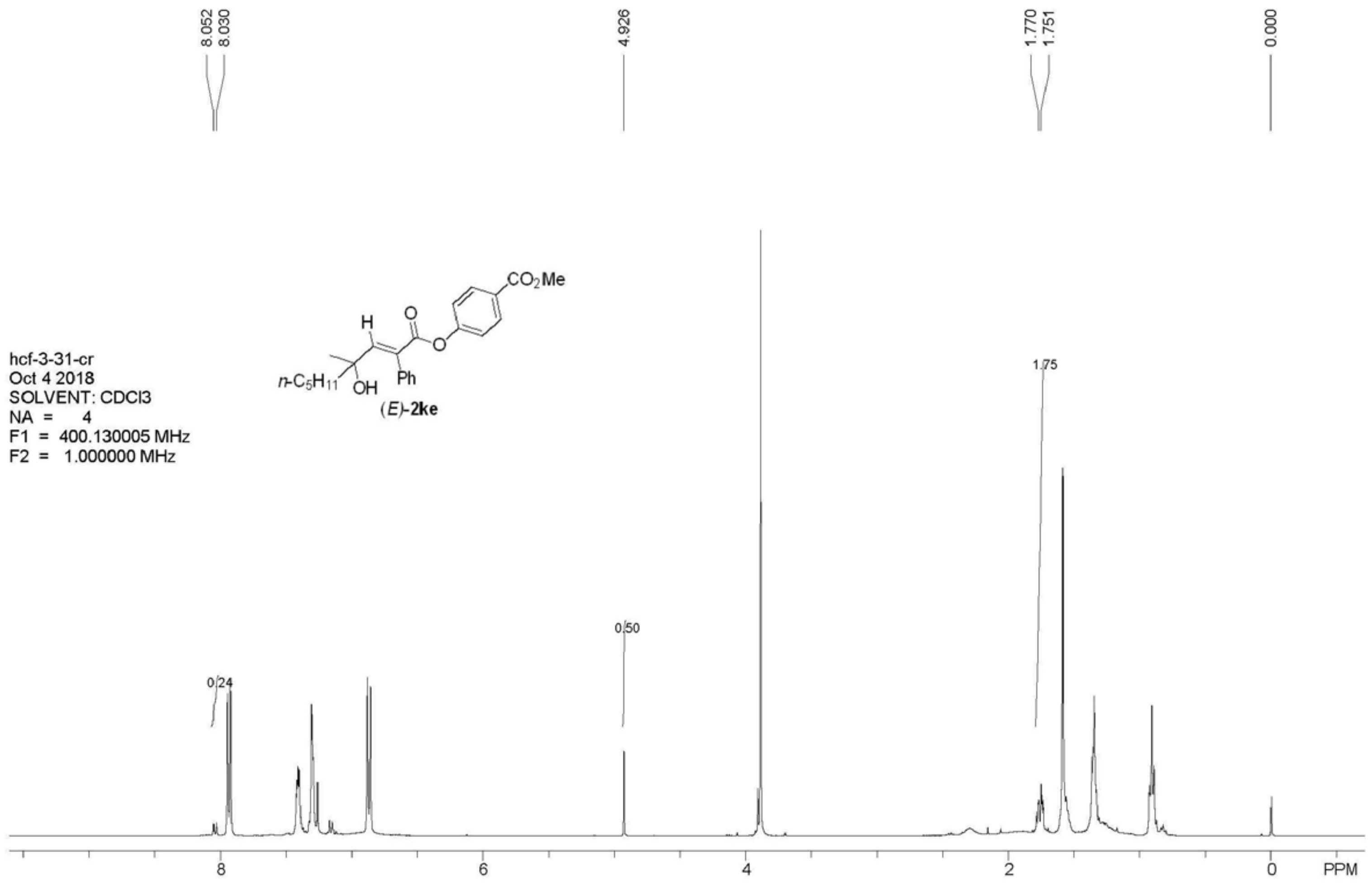
hcf-3-31-cr
Oct 4 2018
SOLVENT: CDCl3
NA = 4
F1 = 400.130005 MHz
F2 = 1.000000 MHz
(E)-2ke
8.052
8.030
4.926
1.770
1.751
0.000
0.24
0.50
1.75
PPM

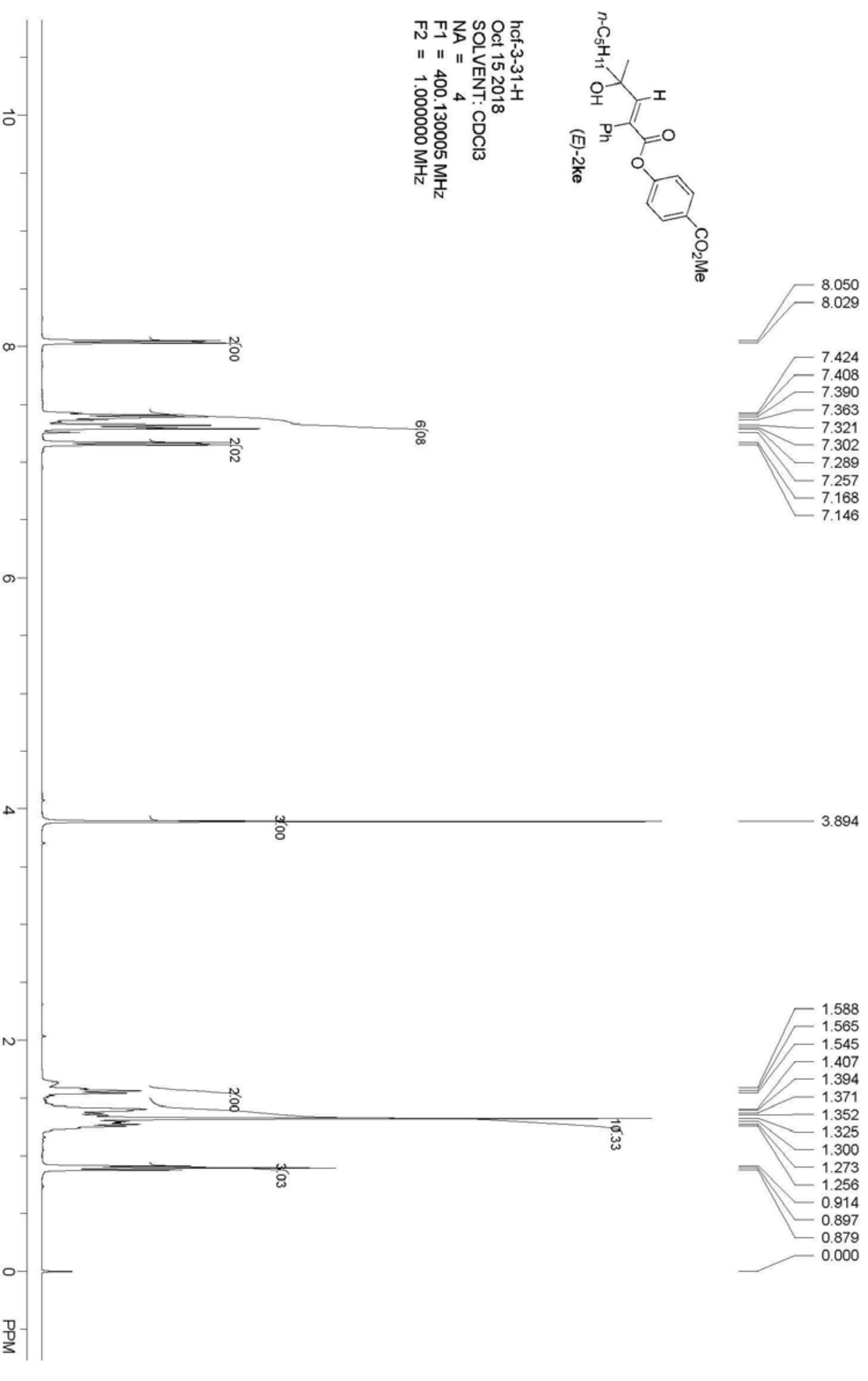
hcf-3-31-H
Oct 15 2018
SOLVENT: CDCl3
NA = 4
F1 = 400.130005 MHz
F2 = 1.000000 MHz
(E)-2ke
n-C5H11
OH
Ph
H
O
CO2Me
8.050
8.029
7.424
7.408
7.390
7.363
7.321
7.302
7.289
7.257
7.168
7.146
3.894
1.588
1.565
1.545
1.407
1.394
1.371
1.352
1.325
1.300
1.273
1.256
0.914
0.897
0.879
0.000
2.00
6.08
2.02
3.00
2.00
10.33
3.03
10
8
6
4
2
0
PPM

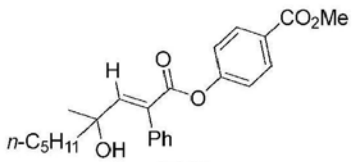

(*E*)-2**ke**

hcf-3-31-C
Oct 15 2018
SOLVENT: CDCl3
NA = 256
F1 = 100.612770 MHz
F2 = 1.000000 MHz

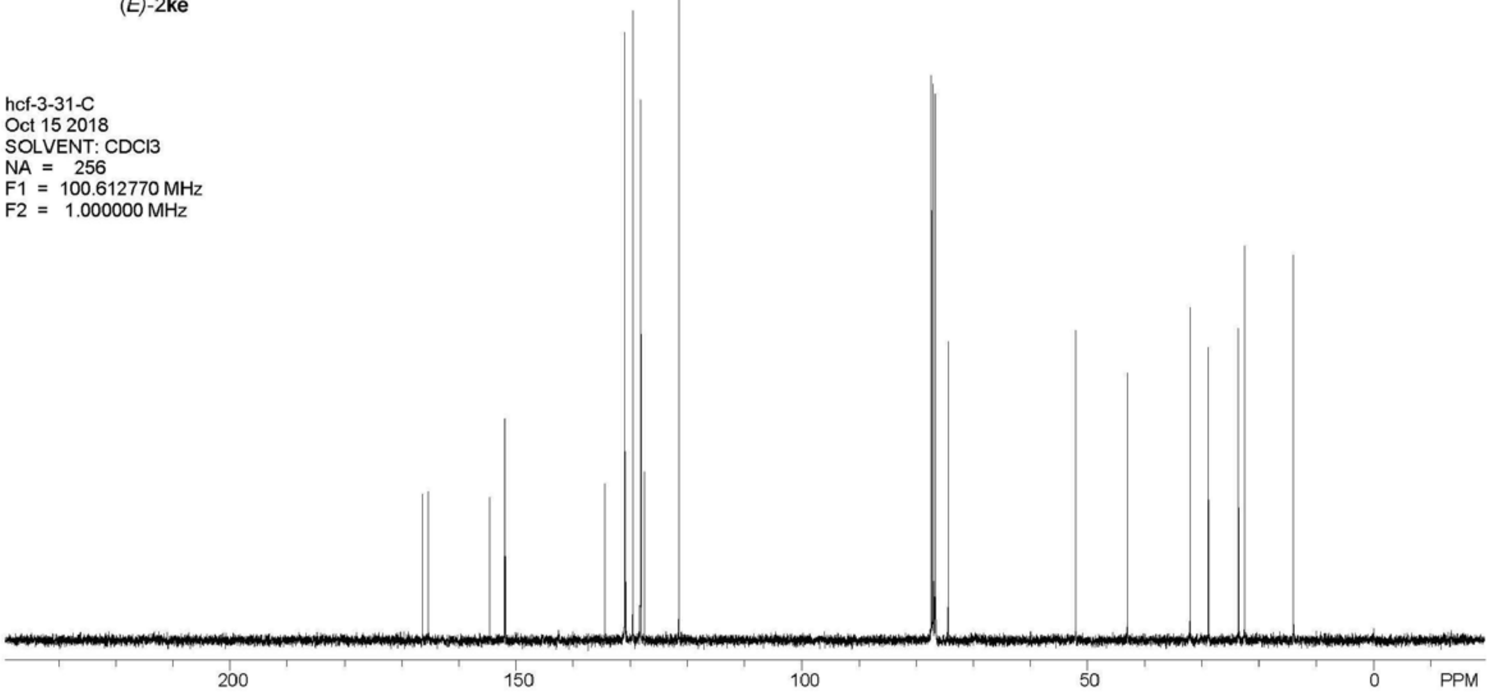

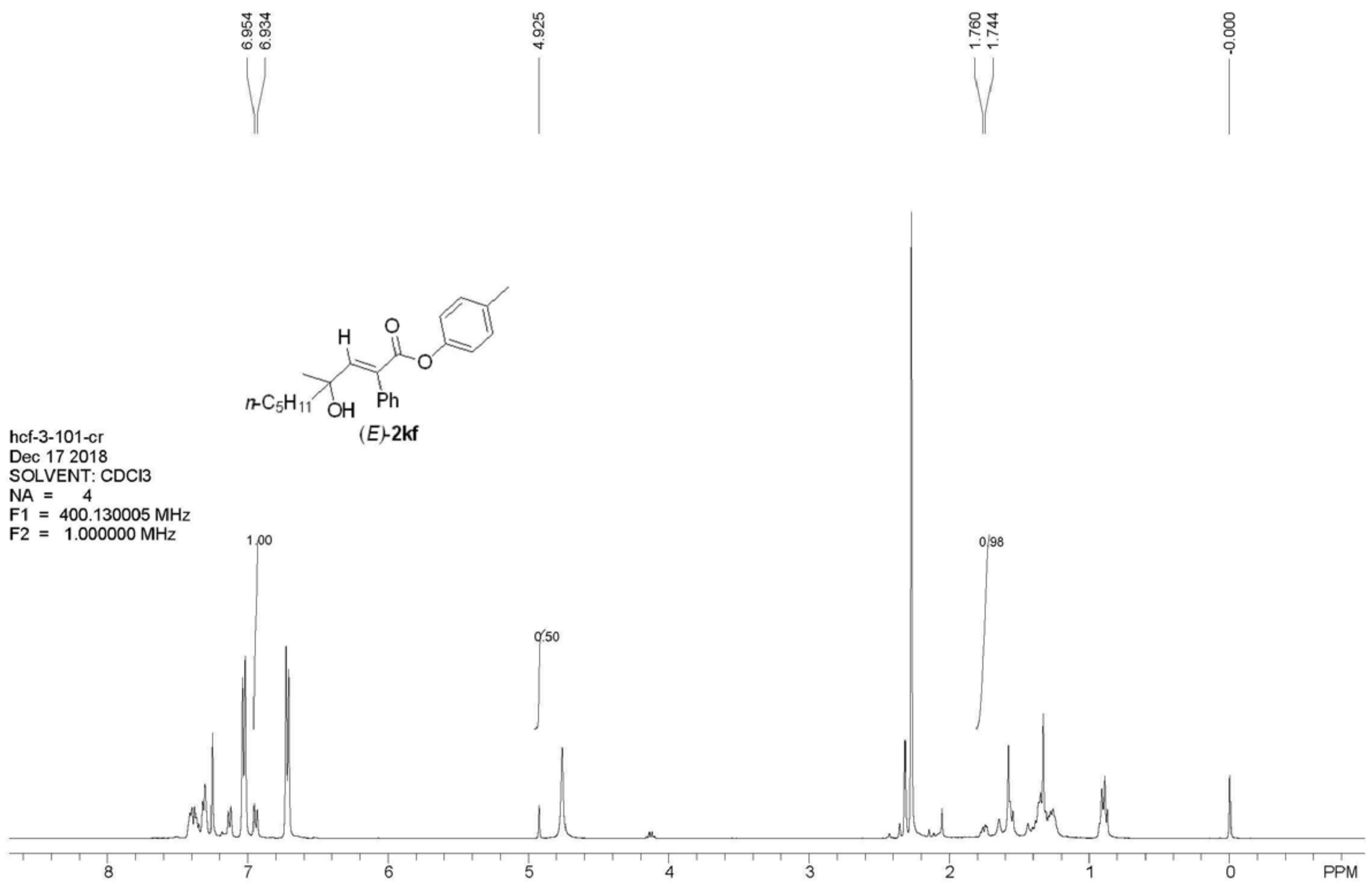
6.954
6.934
4.925
1.760
1.744
-0.000
n-C5H11
OH
Ph
H
O
O
(*E*)-**2kf**
hcf-3-101-cr
Dec 17 2018
SOLVENT: CDCl3
NA = 4
F1 = 400.130005 MHz
F2 = 1.000000 MHz
1.00
0.50
0.98
8
7
6
5
4
3
2
1
0
PPM

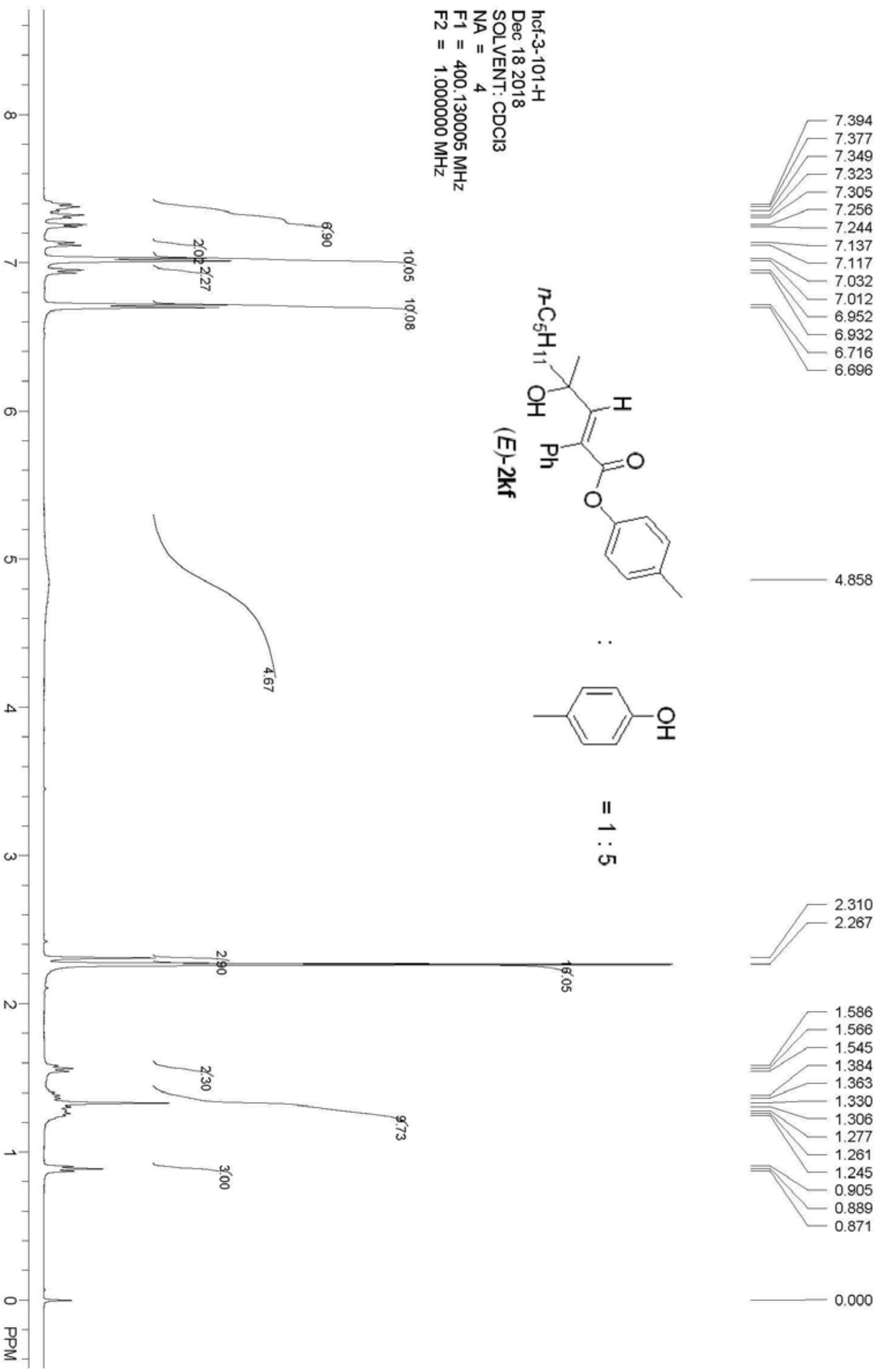
hcf-3-101-H
Dec 18 2018
SOLVENT: CDCl3
NA = 4
F1 = 400.130005 MHz
F2 = 1.000000 MHz
7.394
7.377
7.349
7.323
7.305
7.256
7.244
7.137
7.117
7.032
7.012
6.952
6.932
6.716
6.696
4.858
2.310
2.267
1.586
1.566
1.545
1.384
1.363
1.330
1.306
1.277
1.261
1.245
0.905
0.889
0.871
0.000
6.90
10.05
2.02
2.27
10.08
4.67
2.90
16.05
2.30
9.73
3.00
n-C5H11
OH
H
Ph
O
O
(E)-2kf
:
OH
= 1 : 5
8
7
6
5
4
3
2
1
0
PPM

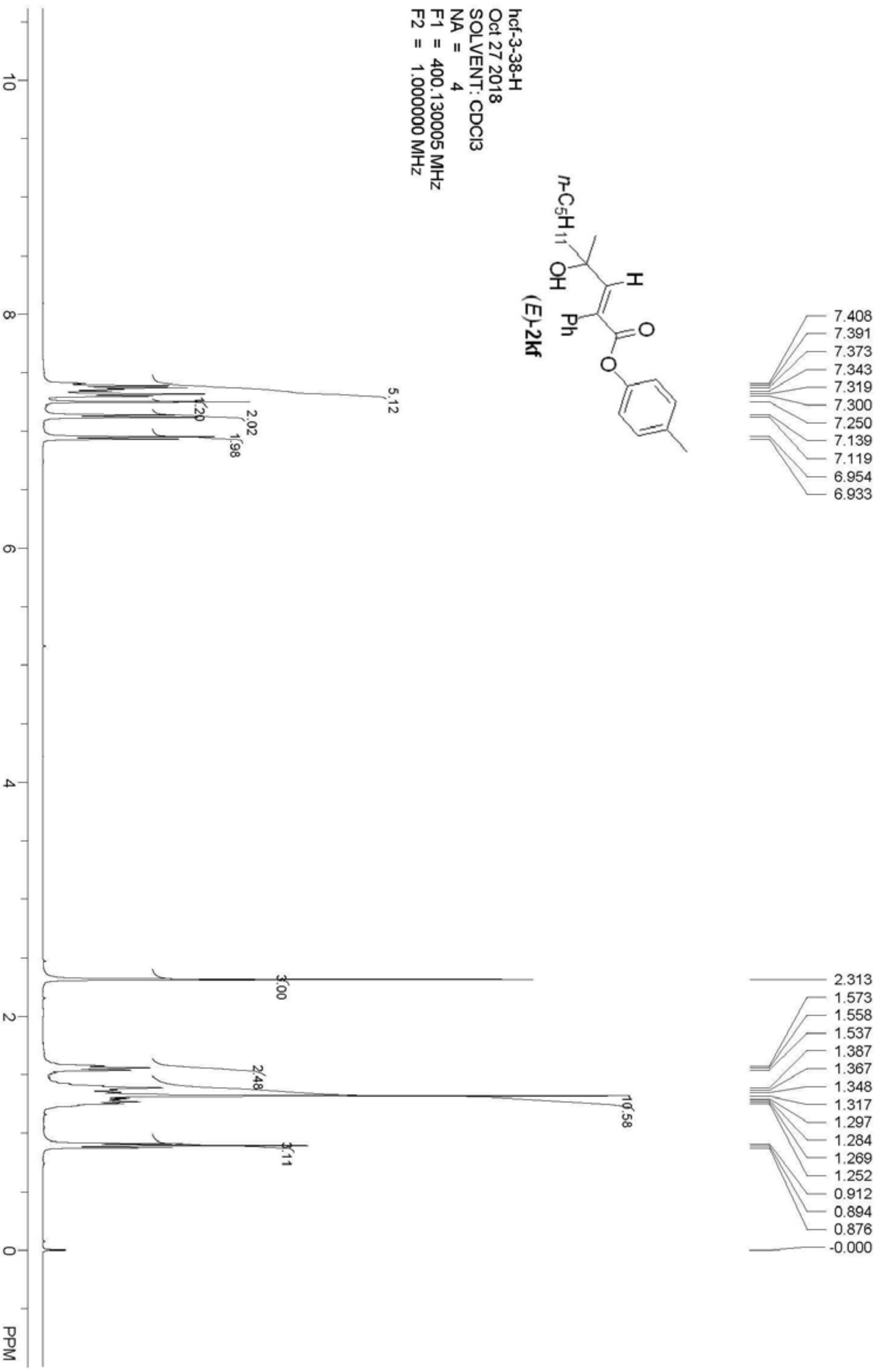
hcf-3-38-H
Oct 27 2018
SOLVENT: CDCl3
NA = 4
F1 = 400.130005 MHz
F2 = 1.000000 MHz
n-C5H11
OH
H
Ph
O
(E)-2kf
7.408
7.391
7.373
7.343
7.319
7.300
7.250
7.139
7.119
6.954
6.933
2.313
1.573
1.558
1.537
1.387
1.367
1.348
1.317
1.297
1.284
1.269
1.252
0.912
0.894
0.876
-0.000
5.12
1.20
2.02
1.98
3.00
2.48
10.58
3.11
10
8
6
4
2
0
PPM

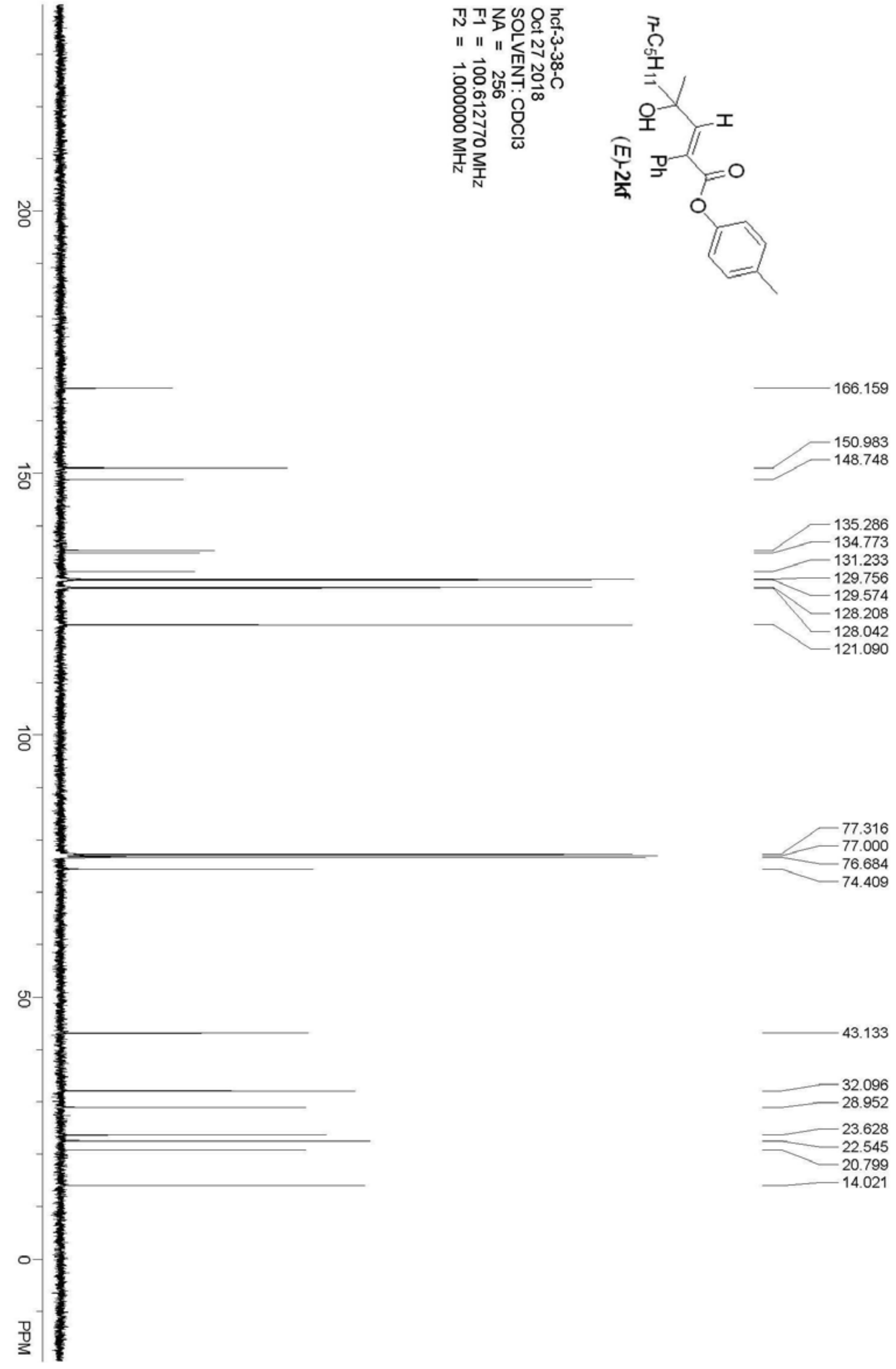
(E)-2kf
n-C5H11
OH
Ph
H
O
hcf-3-38-C
Oct 27 2018
SOLVENT: CDCl3
NA = 256
F1 = 100.612770 MHz
F2 = 1.000000 MHz
166.159
150.983
148.748
135.286
134.773
131.233
129.756
129.574
128.208
128.042
121.090
77.316
77.000
76.684
74.409
43.133
32.096
28.952
23.628
22.545
20.799
14.021
200
150
100
50
0
PPM

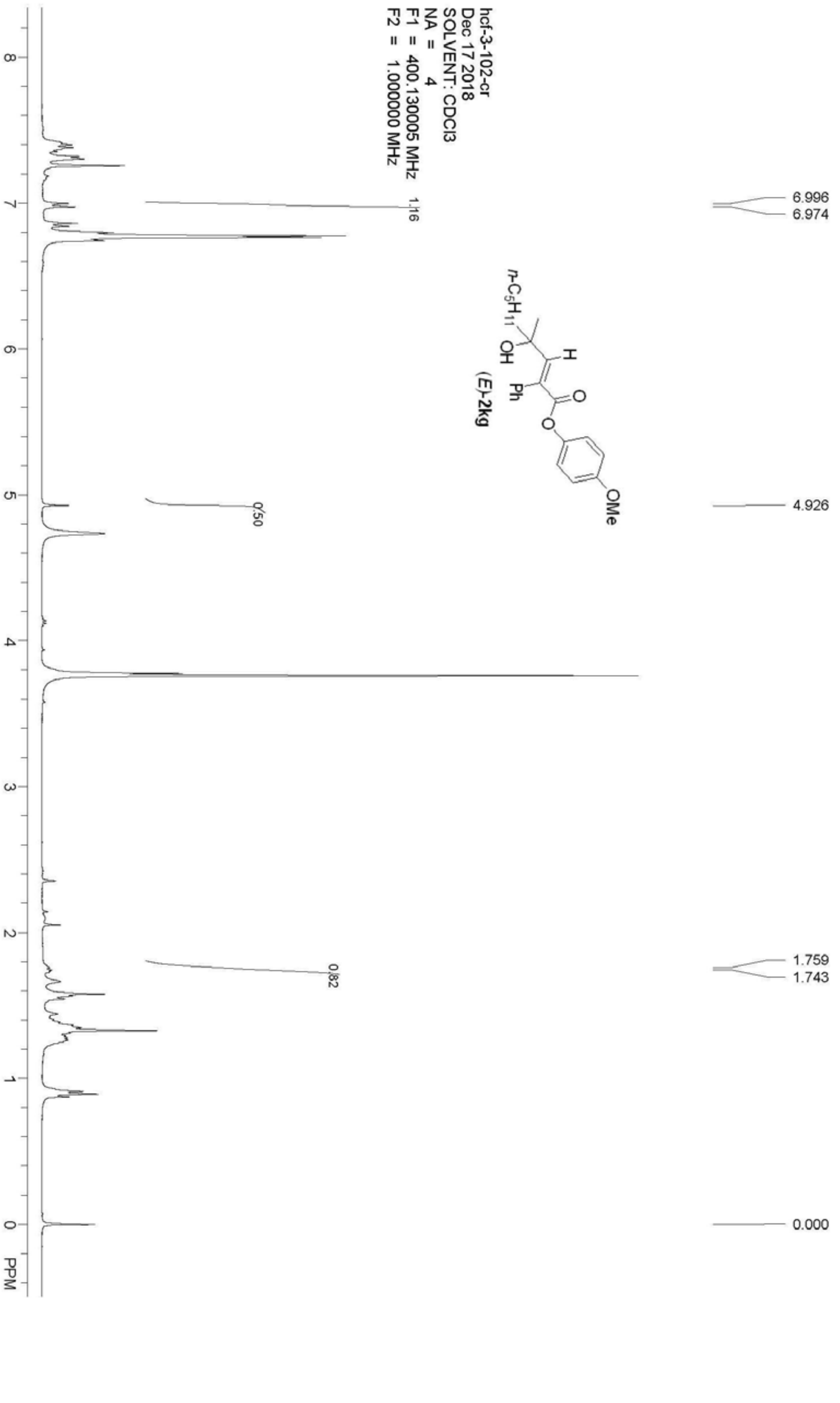
hcf-3-102-cr
Dec 17 2018
SOLVENT: CDCl3
NA = 4
F1 = 400.130005 MHz
F2 = 1.000000 MHz
1.16
0.50
0.82
6.996
6.974
4.926
1.759
1.743
0.000
n-C5H11
OH
H
Ph
O
O
OMe
(E)-2kg
PPM

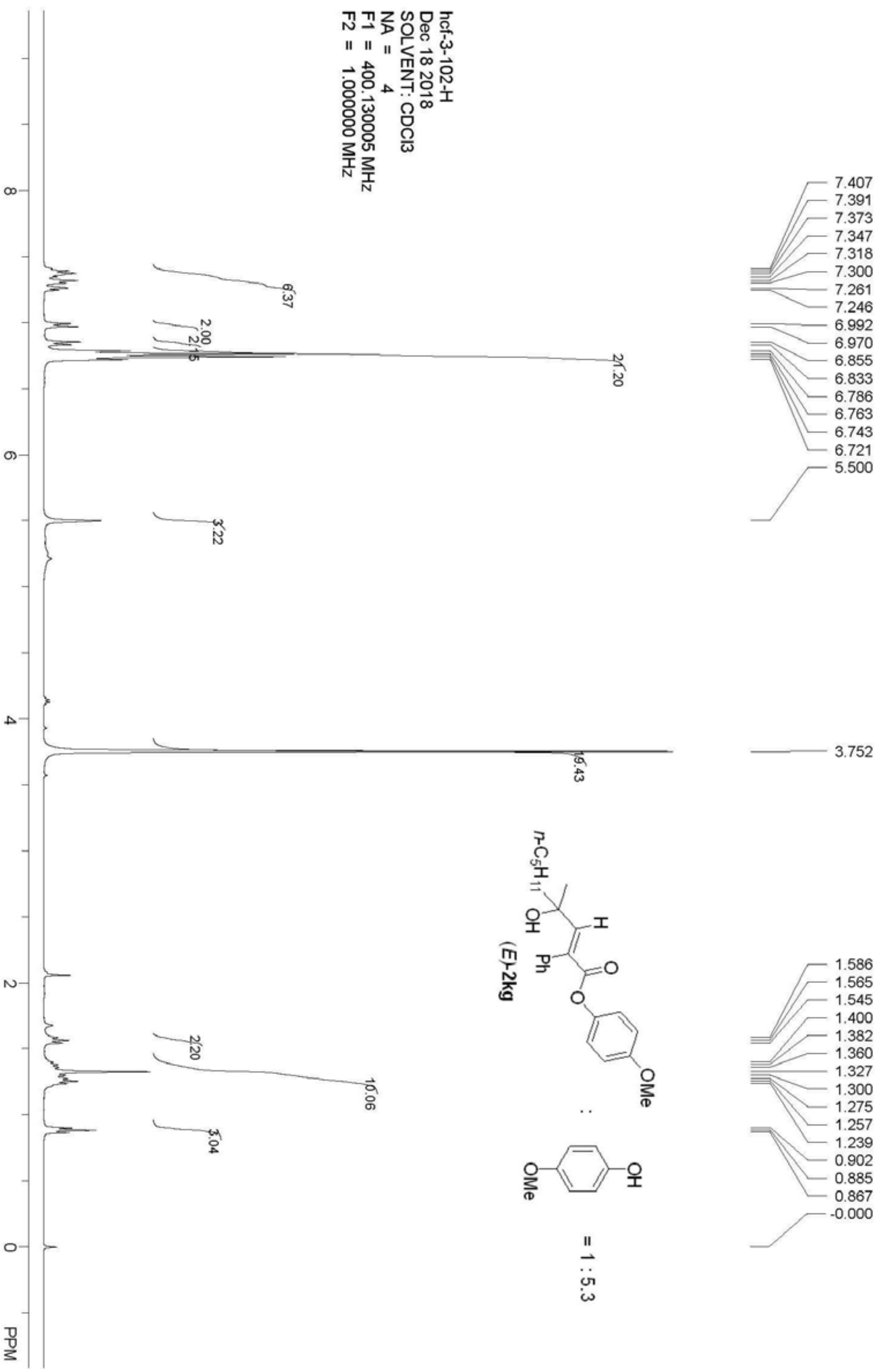
hcf-3-102-H
Dec 18 2018
SOLVENT: CDCl3
NA = 4
F1 = 400.130005 MHz
F2 = 1.000000 MHz
7.407
7.391
7.373
7.347
7.318
7.300
7.261
7.246
6.992
6.970
6.855
6.833
6.786
6.763
6.743
6.721
5.500
3.752
1.586
1.565
1.545
1.400
1.382
1.360
1.327
1.300
1.275
1.257
1.239
0.902
0.885
0.867
-0.000
6.37
2.00
2.15
21.20
3.22
19.43
2.20
10.06
3.04
8
6
4
2
0
PPM
H
O
Ph
OH
OMe
(E)-2kg
:
= 1 : 5.3

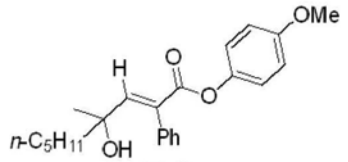

(E)-2kg

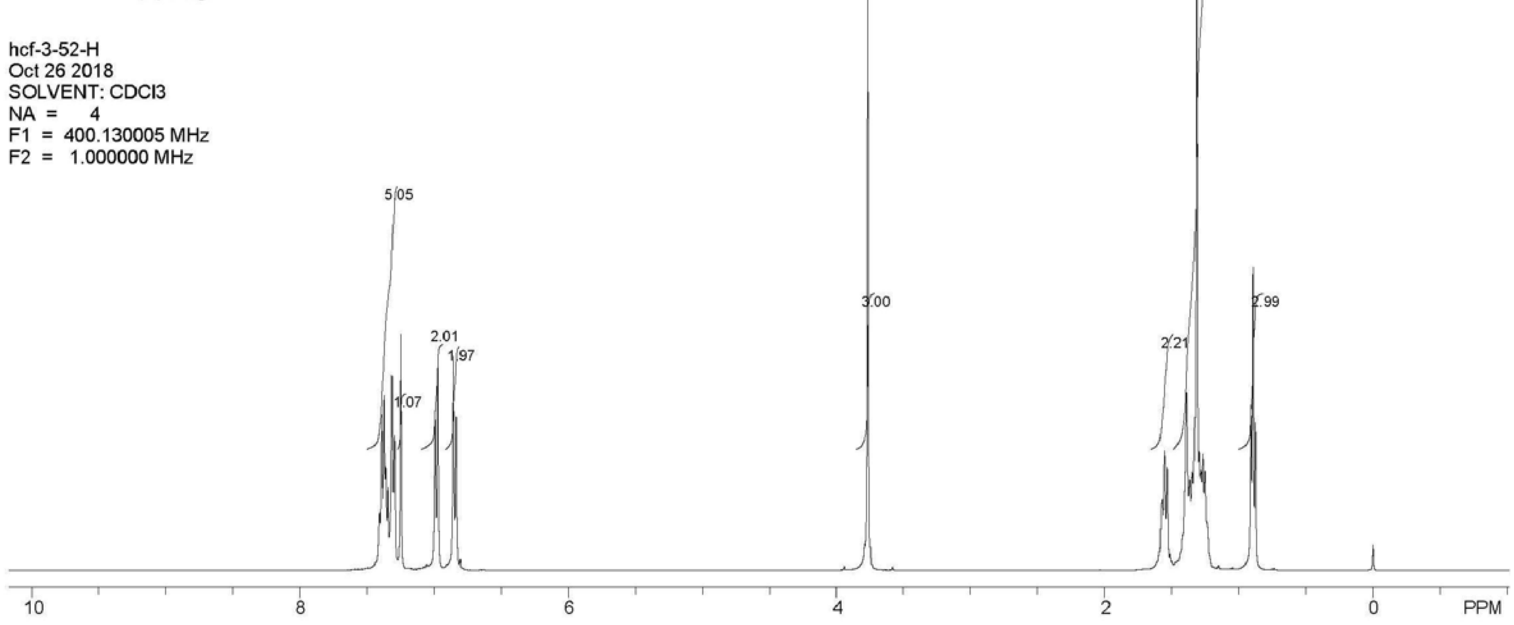
hcf-3-52-H
Oct 26 2018
SOLVENT: CDCl3
NA = 4
F1 = 400.130005 MHz
F2 = 1.000000 MHz
7.408
7.391
7.373
7.360
7.344
7.315
7.296
7.248
6.994
6.972
6.856
6.834
3.765
1.577
1.556
1.535
1.391
1.366
1.347
1.315
1.268
1.251
0.911
0.894
0.876
-0.000
5.05
1.07
2.01
1.97
3.00
2.21
10.15
2.99
10
8
6
4
2
0
PPM

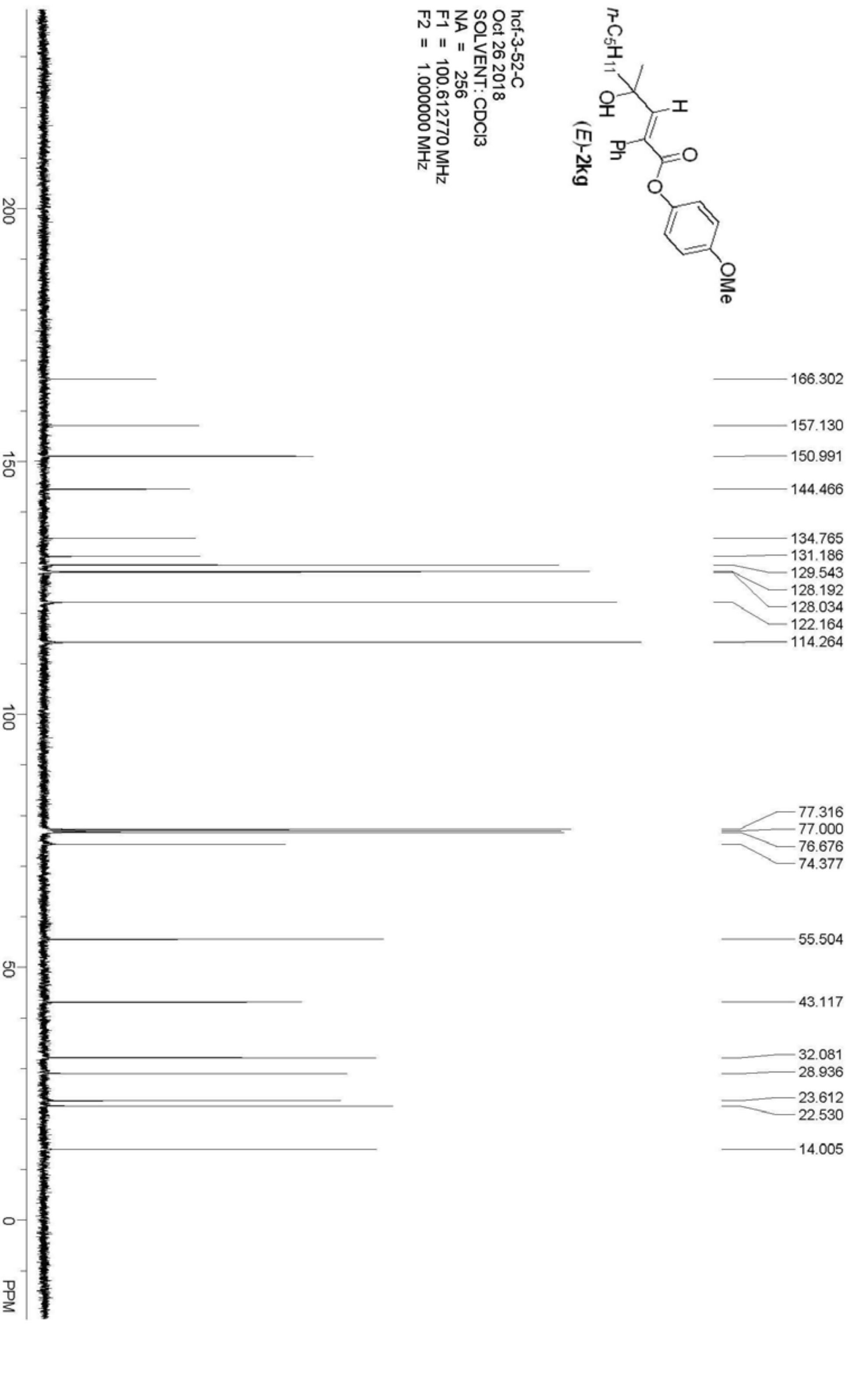
hcf-3-52-C
Oct 26 2018
SOLVENT: CDCl3
NA = 256
F1 = 100.612770 MHz
F2 = 1.000000 MHz
(E)-2kg
166.302
157.130
150.991
144.466
134.765
131.186
129.543
128.192
128.034
122.164
114.264
77.316
77.000
76.676
74.377
55.504
43.117
32.081
28.936
23.612
22.530
14.005
PPM

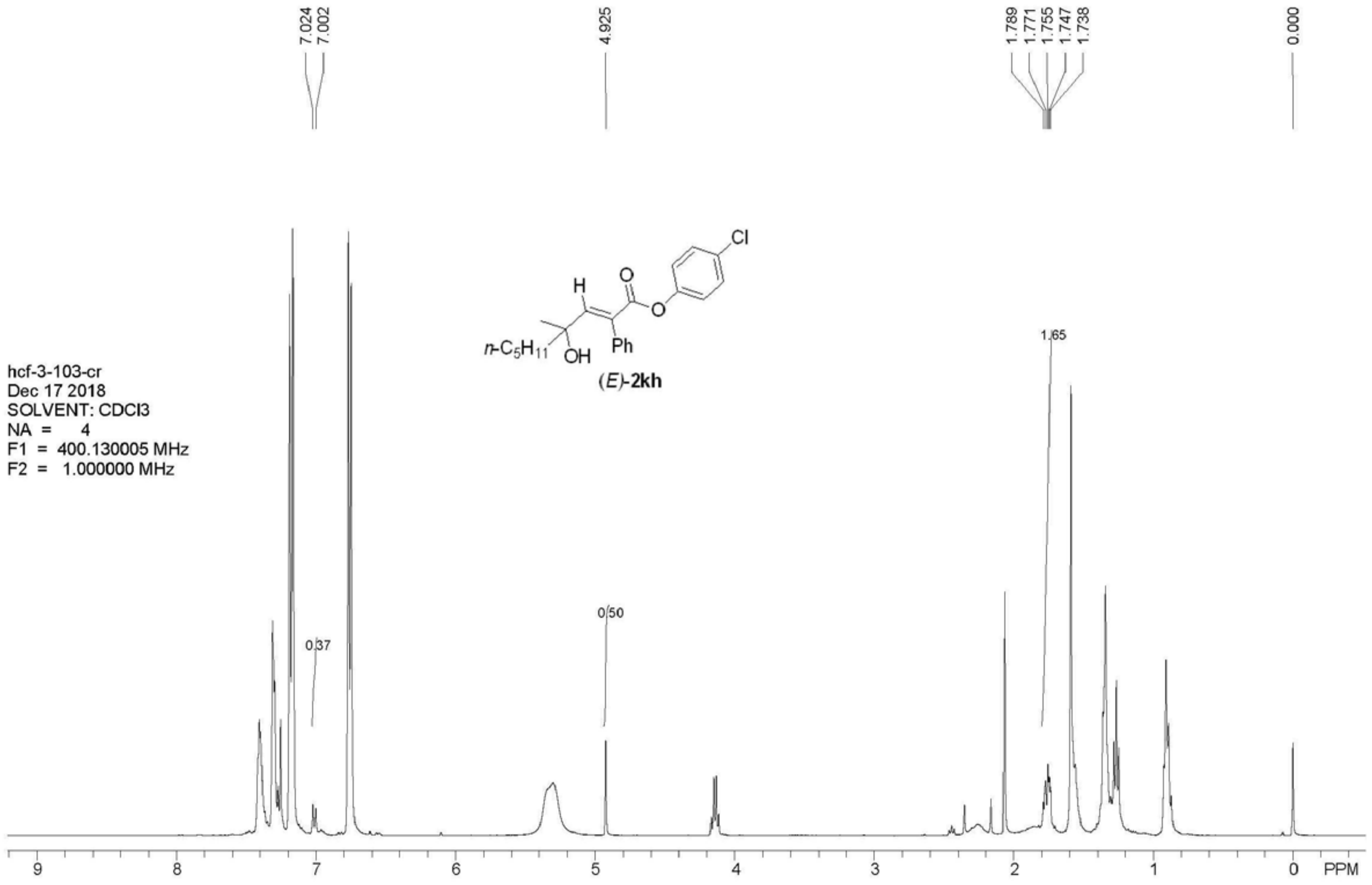
7.024
7.002
4.925
1.789
1.771
1.755
1.747
1.738
0.000
hcf-3-103-cr
Dec 17 2018
SOLVENT: CDCl3
NA = 4
F1 = 400.130005 MHz
F2 = 1.000000 MHz
n-C5H11
OH
H
O
O
Ph
Cl
(E)-2kh
0.37
0.50
1.65
9
8
7
6
5
4
3
2
1
0
PPM

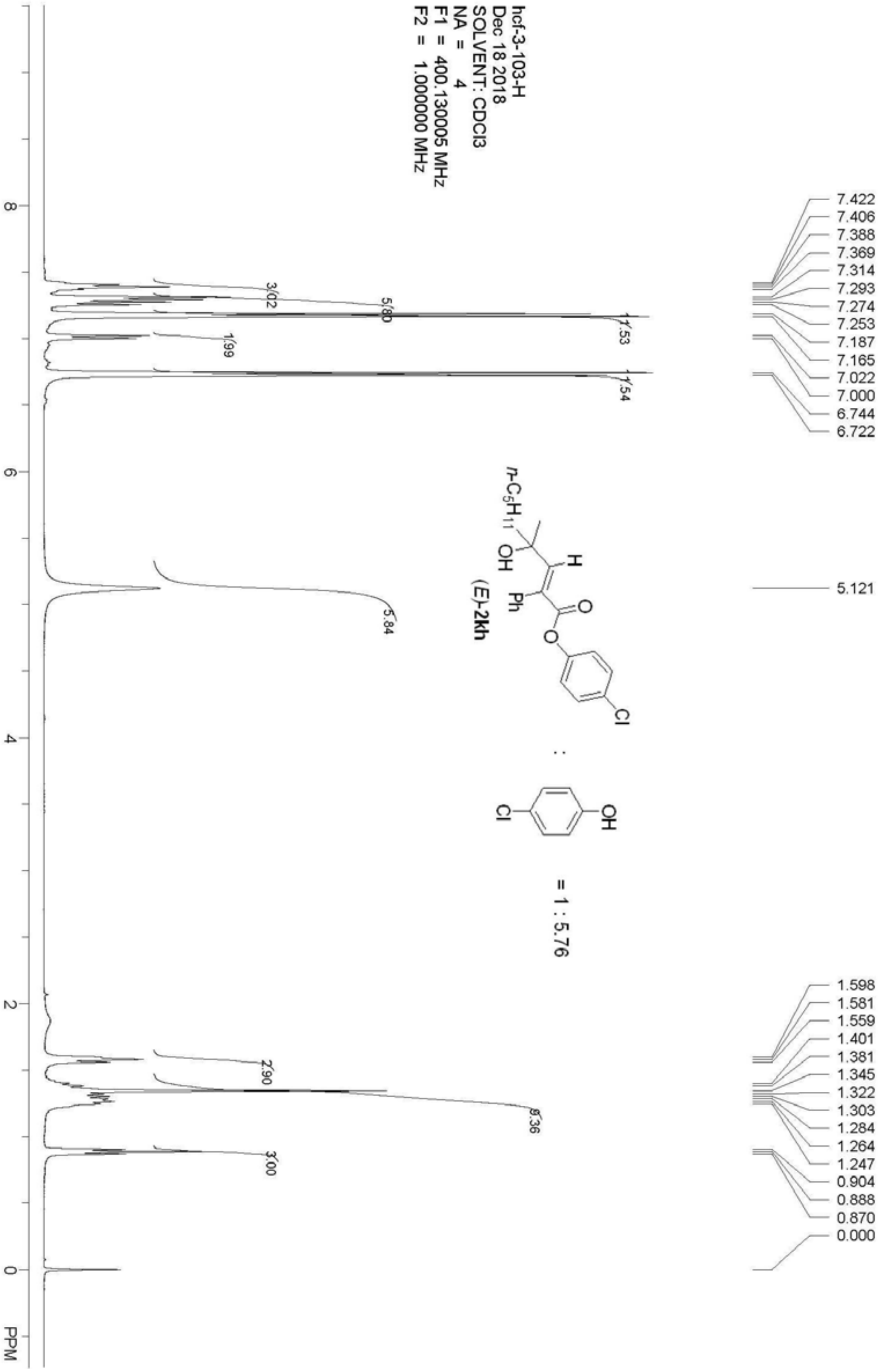
hcf-3-103-H
Dec 18 2018
SOLVENT: CDCl3
NA = 4
F1 = 400.130005 MHz
F2 = 1.000000 MHz
(E)-2Kh
= 1 : 5.76
PPM

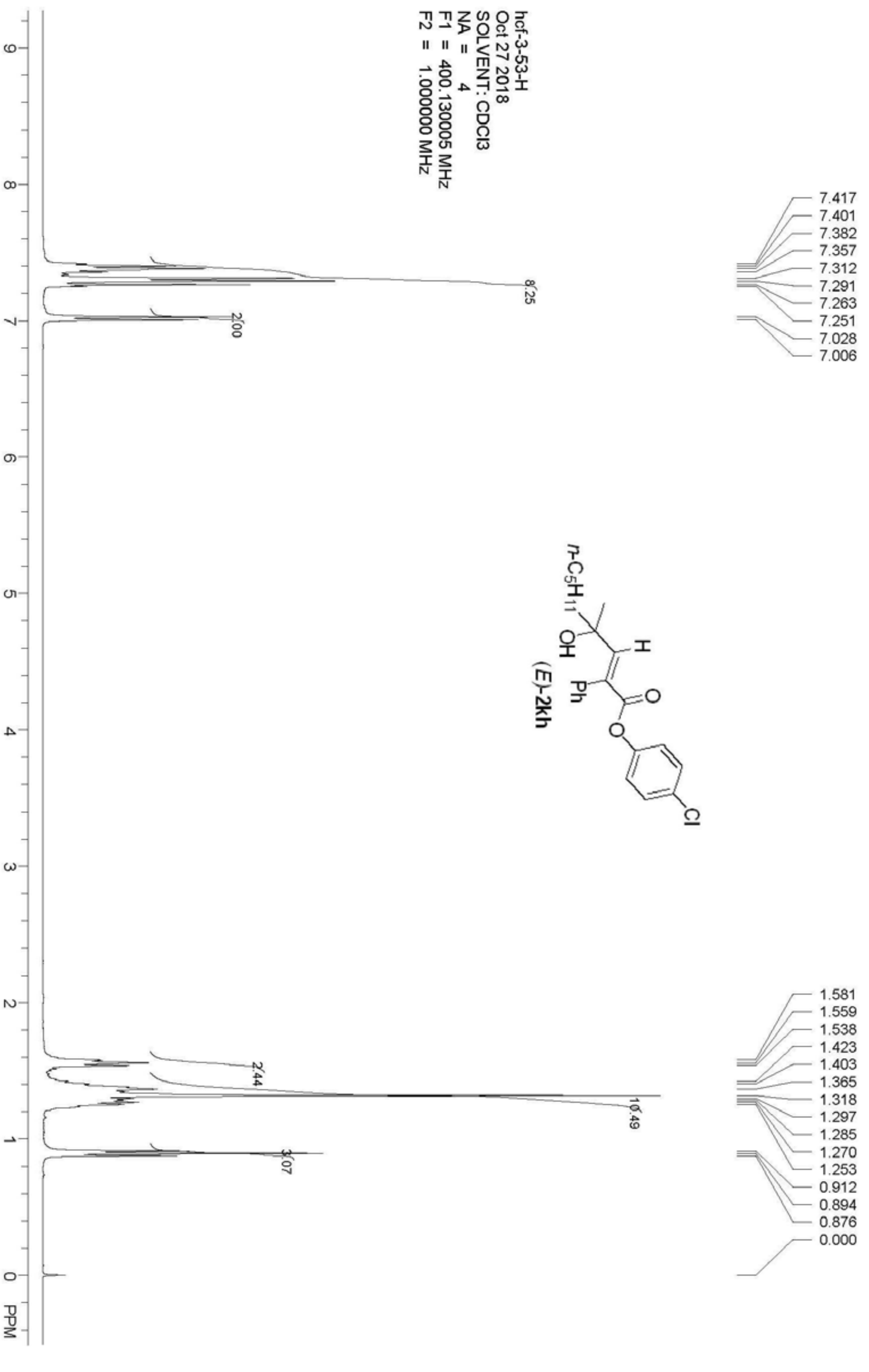
hcf-3-53-H
Oct 27 2018
SOLVENT: CDCl3
NA = 4
F1 = 400.130005 MHz
F2 = 1.000000 MHz
7.417
7.401
7.382
7.357
7.312
7.291
7.263
7.251
7.028
7.006
1.581
1.559
1.538
1.423
1.403
1.365
1.318
1.297
1.285
1.270
1.253
0.912
0.894
0.876
0.000
8.25
2.00
2.44
10.49
3.07
n-C5H11
OH
H
Ph
O
Cl
(E)-2kh
PPM

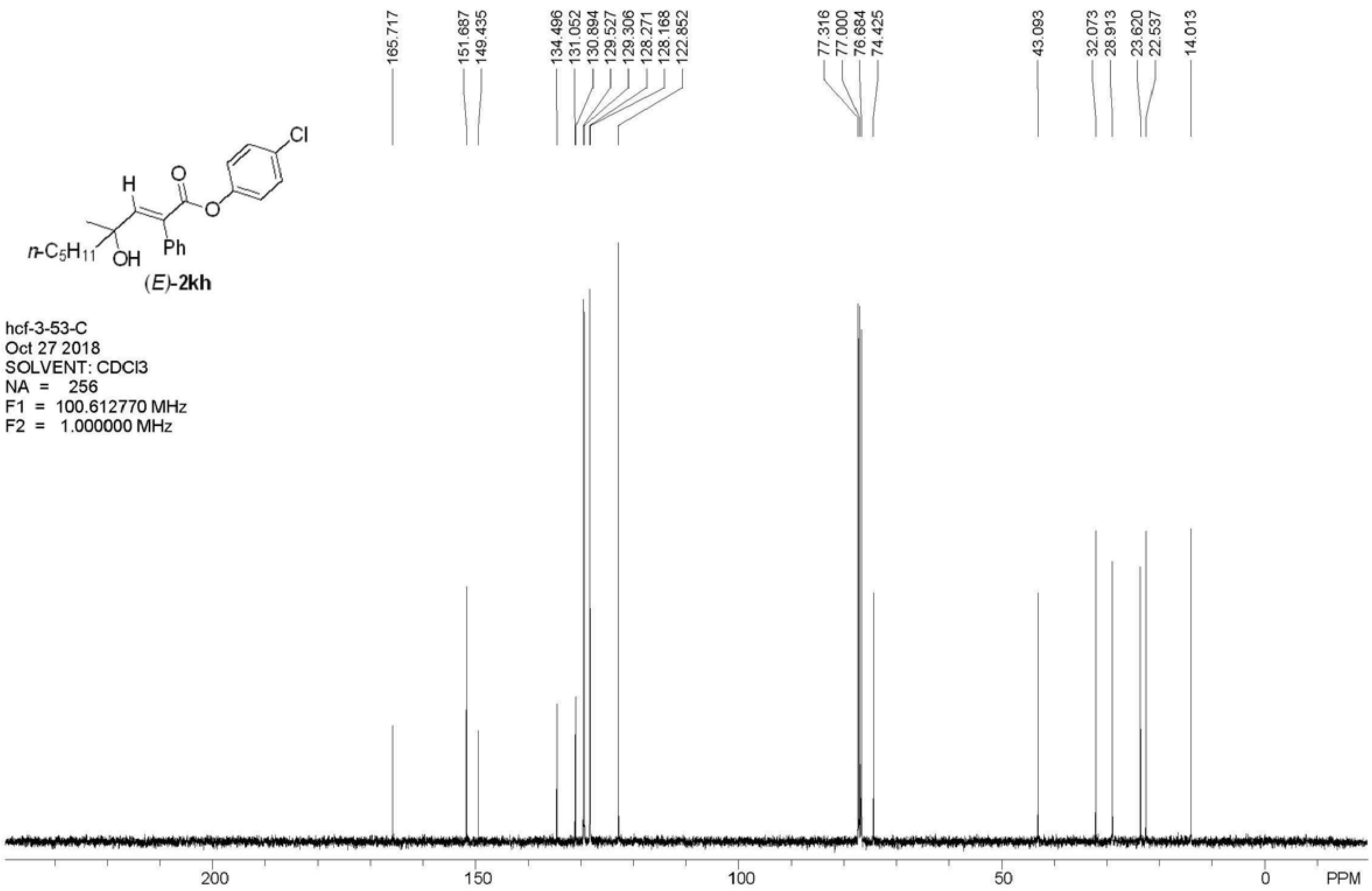

n-C5H11
OH
H
O
Ph
Cl
hcf-3-53-C
Oct 27 2018
SOLVENT: CDCl3
NA = 256
F1 = 100.612770 MHz
F2 = 1.000000 MHz
165.717
151.687
149.435
134.496
131.052
130.894
129.527
129.306
128.271
128.168
122.852
77.316
77.000
76.684
74.425
43.093
32.073
28.913
23.620
22.537
14.013
200
150
100
50
0
PPM

(*E*)-**2kh**
